# Fangorn Forest (F2): a machine learning approach to classify genes and genera in the family *Geminiviridae*

**DOI:** 10.1186/s12859-017-1839-x

**Published:** 2017-09-30

**Authors:** José Cleydson F. Silva, Thales F. M. Carvalho, Elizabeth P. B. Fontes, Fabio R. Cerqueira

**Affiliations:** 1 Department of Informatics, Universidade Federal de Viçosa, Viçosa, Minas Gerais 36570-900 Brazil; 2 National Institute of Science and Technology in Plant-Pest Interactions/BIOAGRO, Campus Universitário, Viçosa, Minas Gerais 36570-900 Brazil; 3 Department of Biochemistry and Molecular Biology, Universidade Federal de Viçosa, Campus Universitário, Viçosa, Minas Gerais 36570-900 Brazil; 4 Department of Production Engineering, Universidade Federal Fluminense, Rua Domingos Silvério, s/n, Bairro Quitandinha, Petrópolis, Rio de Janeiro 25650-050 Brazil

**Keywords:** Geminivirus; machine learning, Gene classification, Genus classification, Random Forest, Multilayer perceptron, Support vector machines

## Abstract

**Background:**

Geminiviruses infect a broad range of cultivated and non-cultivated plants, causing significant economic losses worldwide. The studies of the diversity of species, taxonomy, mechanisms of evolution, geographic distribution, and mechanisms of interaction of these pathogens with the host have greatly increased in recent years. Furthermore, the use of rolling circle amplification (RCA) and advanced metagenomics approaches have enabled the elucidation of viromes and the identification of many viral agents in a large number of plant species. As a result, determining the nomenclature and taxonomically classifying geminiviruses turned into complex tasks. In addition, the gene responsible for viral replication (particularly, the viruses belonging to the genus *Mastrevirus*) may be spliced due to the use of the transcriptional/splicing machinery in the host cells. However, the current tools have limitations concerning the identification of *introns*.

**Results:**

This study proposes a new method, designated Fangorn Forest (F2), based on machine learning approaches to classify genera using an *ab initio* approach, i.e., using only the genomic sequence, as well as to predict and classify genes in the family *Geminiviridae*. In this investigation, nine genera of the family *Geminiviridae* and their related satellite DNAs were selected. We obtained two training sets, one for genus classification, containing attributes extracted from the complete genome of geminiviruses, while the other was made up to classify geminivirus genes, containing attributes extracted from ORFs taken from the complete genomes cited above. Three ML algorithms were applied on those datasets to build the predictive models: support vector machines, using the sequential minimal optimization training approach, random forest (RF), and multilayer perceptron. RF demonstrated a very high predictive power, achieving 0.966, 0.964, and 0.995 of precision, recall, and area under the curve (AUC), respectively, for genus classification. For gene classification, RF could reach 0.983, 0.983, and 0.998 of precision, recall, and AUC, respectively.

**Conclusions:**

Therefore, Fangorn Forest is proven to be an efficient method for classifying genera of the family *Geminiviridae* with high precision and effective gene prediction and classification. The method is freely accessible at www.geminivirus.org:8080/geminivirusdw/discoveryGeminivirus.jsp.

**Electronic supplementary material:**

The online version of this article (10.1186/s12859-017-1839-x) contains supplementary material, which is available to authorized users.

## Background

*Geminiviridae* is one of the largest and most successfully plant virus families. This family comprises viruses with single-strand DNA genome encapsulated in twinned icosahedral particles. Geminiviruses infect several species of cultivated and ornamental plants as well as weeds, causing significant economic losses in agriculture and food safety worldwide [1]. The family *Geminiviridae* comprises nine genera: *Begomovirus*, *Mastrevirus*, *Becurtovirus, Curtovirus, Turncurtovirus, Eragrovirus, Topocuvirus, Capulavirus*, and *Graglovirus* [2–4]. Geminivirus genomes are comprised of a genomic component called DNA-A. Viruses of the *Begomovirus* genus are exceptions. Their genomes can present only the component DNA-A (monopartite), similarly to other geminiviruses, or two components: DNA-Aand DNA-B (bipartite). The component DNA-A may be transmitted by the silverleaf whitefly (*Bemisia tabaci* of biotypes A or B), particularly for begomoviruses; by leafhoppers (mastreviruses, becurtoviruses, and curtoviruses), and by treehoppers (topocuviruses) [1, 2, 5, 6]. The genera *Eragrovirus* and *Turncurtovirus* have no known vector yet. The genomes of bipartite *Begomovirus* are mostly found in the New World, while monopartite ones (made up of only DNA-A) are commonly found in the Old World [7–9].

Recent studies report the first occurrence of monopartite geminivirus (begomoviruses) infecting tomatoes in Peru and Ecuador [10]. Conversely, bipartite begomoviruses have been identified in the Old World (Madagascar) infecting *Asystasia gangetica* and associated with mosaic disease in *Coccinia grandis* in India [11–13]. Overall, diseases caused by geminiviruses have had economic and social impacts in several continents. For example, in Europe, tomato plants have been infected by the tomato yellow leaf curl virus disease (TYLCD) and wheat has been severely inflicted by the wheat dwarf virus disease (WDVD) [14–16]. In Africa, the cassava mosaic disease (CMD) and the maize streak disease (MSD) have been reported [17, 18]. There have also been occurrences of the cotton leaf curl disease (CLCuD) and the chickpea chlorotic dwarf disease in Asia, as well as the bean golden mosaic disease (BGMD) in the Americas [19–21].

The genomic organization of geminiviruses is highly conserved. However, the species are genetically divergent, encoding two to seven genes, with long and short intergenic regions and a common region between DNA-A and DNA-B [2]. DNA-A encodes CP (capsid proteins), Rep (a protein associated with replication), TrAP (transcriptional activator protein and gene silencing suppressor), REn (replication enhancer protein), Reg (gene regulator), Sd (or AC4, symptom determinant and gene silencing suppressor), and AC5 (recently studied and functionally described as a determinant of pathogenicity that suppresses antiviral defenses based on RNA silencing) [2, 22]. Furthermore, monopartite geminiviruses in the Old World contain a pre-coat protein (V2) related to movement and transport of viral genome in the plant.

DNA-B (reported for begomovirus) is responsible for the transport and movement of viral DNA in the plant and codes two proteins, MP (movement protein) and NSP (nuclear transport protein). NSP facilitates the intracellular transport of viral DNA from the nucleus to the cytoplasm and acts in concert with MP to move the viral DNA to the adjacent, uninfected cells [23]. In some cases, geminiviruses may be associated with beta satellite (DNA-Beta) or alpha satellite DNA (DNA-Alpha) [24]. Beta satellites are DNA molecules with approximately 1.35 kb, and code a single ORF betaC1 (pathogenicity determinant protein), which acts in the development of symptoms, modulation of virus host range, and host defense response [25–27]. In contrast, alpha satellites are capable of autonomous replication but are dependent on geminiviruses for systemic infection and vector transmission [28, 29]. The genome of alpha satellites contains approximately 1.37 kb and codes a single Rep protein.

Recent researches have shown the high diversity of geminivirus species, multiple hosts, and geographic distribution in various regions of the Old and New Worlds [2, 30–32]. Currently, high-throughput sequencing methods, advanced metagenomics approaches, and different bioinformatics tools have enabled elucidating viromes and identifying many viral agents in a large number of plant species. In addition, using the rolling circle amplification (RCA) approach [33], thousands of sequences or complete genomes have been amplified, sequenced, and made available in public databases (GenBank NCBI, geminivirus.org). Currently, geminiviruses are classified based on the type of insect vector, host range, phylogenetic reconstruction, and genomic organization [2]. Therefore, geminivirus classification requires knowledge of taxonomy and bioinformatics since different computational tools and algorithms can be used. For example, the algorithms Muscle, MAFFT, ClustalW, and BLAST are often used for alignment of sequences [34–37]. Methods, including neighbor-joining, maximum parsimony, maximum likelihood, and Bayesian inference, are also used to obtain phylogenetic reconstruction [3, 4, 38]. Other approaches using pairwise sequence comparisons are also widely employed. Those comparisons are used by the software SDT [39] and analyzed according to the taxonomic criterion of each genus. Several previous works have applied those computational tools to provide taxonomic reviews [2–4, 30–32, 40]. Guidelines and protocols have been proposed to demarcate and classify species for *Becurtovirus, Eragroviru*s, and *Turncurtovirus* [2]. Similarly, criteria have also been proposed for begomoviruses and mastreviruses [30, 31]. In order to evaluate the genomic organization, the Open Reading Frames (ORFs) and their respective positions in the genome must be first obtained. In this step, ORFs are predicted by the ORF finder tool (https://www.ncbi.nlm.nih.gov/orffinder/), which, although widely used, has limitations in identifying introns of this family. Other consolidated tools, such as AUGUSTUS (http://augustus.gobics.de/), Geneid (http://genome.crg.es/software/geneid/index.html) and Prodigal (https://github.com/hyattpd/Prodigal), are still limited to identify all ORFs that are encoded by the geminivirus genomes. Even though the computer programs cited above are robust and help taxonomic classification, they are of general purpose, i.e., they were not designed taking the peculiarities of geminivirus genomes into account. Furthermore, they often use databases with non-standardized, non-curated sequences with frequent annotation errors. Still, in general, the required methods are not integrated. Such integration would facilitate automating the data analysis process and decision-making.

We hereby present an in silico prediction approach, called Fangorn Forest (F2), capable of classifying genera and genes in the *Geminiviridae* family based on machine learning (ML) methods. F2 uses only genomic characteristics common to any viral genome to build classification models. In this research, all genera (nine) of the family *Geminiviridae* and their related satellite DNAs were considered. The proposed method is proven to be highly accurate, as the machine learning models used yielded very high values of precision, recall, and area under the ROC curve (AUC) for the classification tasks. F2 integrates the set of computational tools of the data warehouse www.geminivirus.org:8080/geminivirusdw/discoveryGeminivirus.jsp [41].

## Methods

### Data source

Initially, genome sequences of plant viruses were retrieved from the GenBank database for composing the negative class (non-geminiviruses) of the training set for family classification.The non-geminivirus class is composed by DNA sequence of different families of plant viruses. This class consists of double-stranded DNA sequences (*Caulimovidae*), double-stranded RNA viruses (*Amalgaviridae, Fijiviridae, Oryzaviridae*), single-stranded DNA (*Nanoviridae*), negative sense single-stranded RNA viruses (Ophioviridae) and positive sense single-chain RNA viruses (*Benyviridae, Bromoviridae, Closteroviridae, Luteoviridae, Potyviridae, Tombusviridae, Virgaviridae*) (http://viralzone.expasy.org/). This class was intended to distinguish genomic sequences of geminiviruses from other plant viruses.

Complete genome sequences of species from eight genera in the *Geminivividae* family as well as satellite DNAs were used to create the positive class of the training set instances for *Geminiviridae* family classification (mentioned before) and genus classification. All sequences were obtained from the geminivirus.org curated repository [40]. The sequences of *Begomovirus*, *Mastrevirus*, *Becurtovirus, Curtovirus, Turncurtovirus, Eragrovirus, Capulavirus*, and *Graglovirus* were defined according to taxonomic reviews [2–4, 30–32, 41, 42]. Additionally, the complete genomes of betasatellites were chosen in conformity with the study of Briddon et al. [31], while sequences of alphasatellites and DNA-B were randomly selected from the geminivirus.org repository. The genus *Topocuvirus* was not selected because has only one sequence deposited in GenBank database.

A family test set was also created using sequences of GenBank database. These sequences. Which were not present in the training set, were used only for the negative class. The sequences used in the positive class were retrieved from geminivirus.org. Also a genus test set was also created using sequences of geminivirus.org, which were not present in the training set. Therefore, four datasets were created. Two datasets (for training and test) comprised of instances of two classes (geminiviruses and non-geminiviruses) and two resultant datasets (for training and test) were comprised of instances of ten classes: begomoviruses/DNA-B, mastreviruses, becurtoviruses, curtoviruses, turncurtoviruses, eragroviruses, capulaviruses, grabloviruses, alphasatellites, and betasatellites.

After creating datasets related to genus classification, we also built training and test sets for gene (ORF) classification. To make up the ORF training set, we selected ORFs contained in the genomes and used in the aforementioned genus training set. In the same way, the ORF test set was composed of ORFs extracted from the same sequences considered to build the genus test set mentioned above. The instance classes of the resultant datasets related to ORF classification are: betaC1, alphaRep, Rep, TrAP, REn, Sd/p.sd, AC5, CP, pre-coat, Reg, MP, and NSP.

**Fig. 1:**
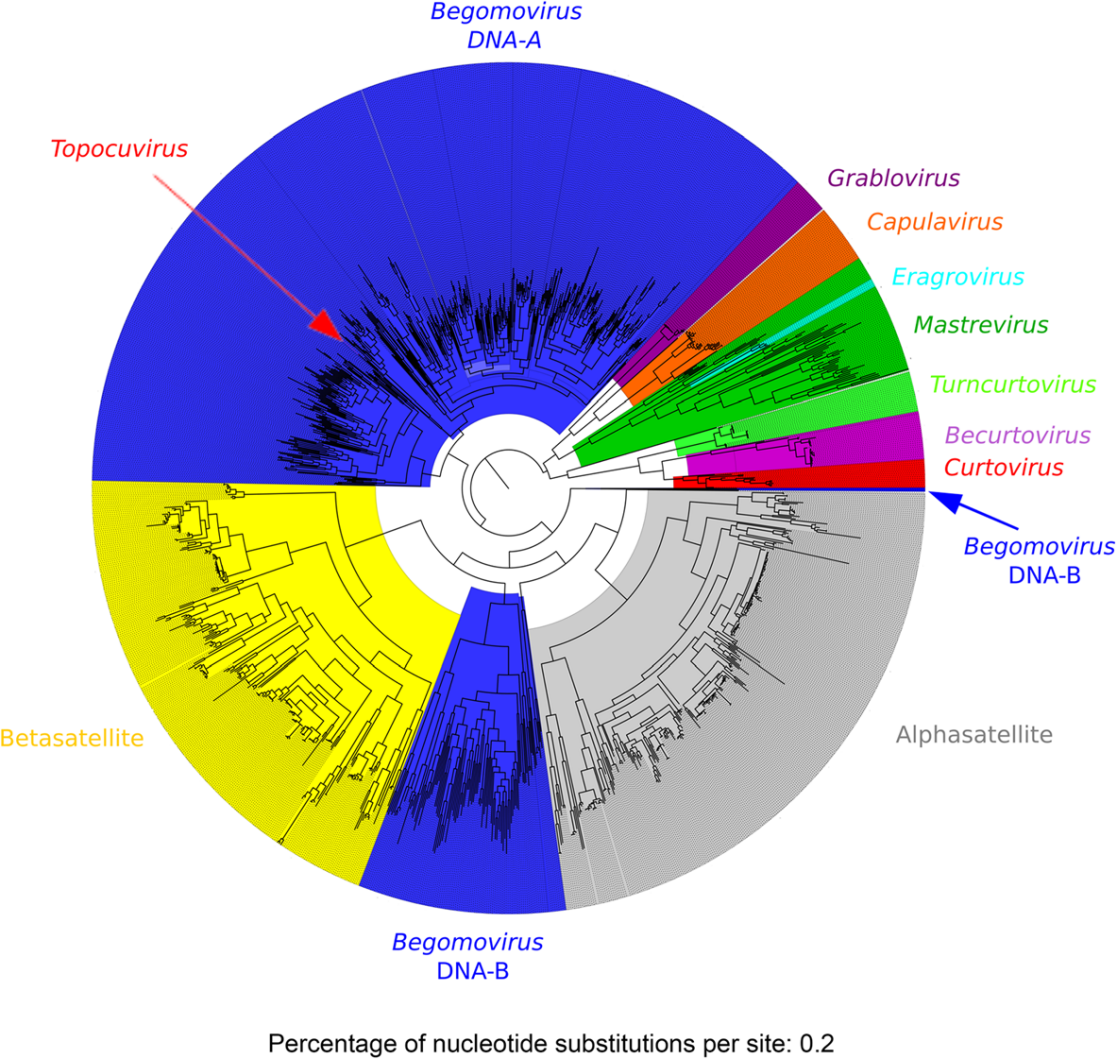
Phylogenetic reconstruction of the *Geminiviridae* family and satellite DNAs. To perform the phylogenetic reconstruction of geminiviruses, all genomic sequences belonging to the genus training set were used. Sequences were aligned using the MAFFT algorithm. The phylogenetic reconstruction was obtained through the program FastTree version 2.1.7. The phylogenetic tree was visualized and edited using the program FigTree v1.4.2

As could be noted, we perform a multi-class classification in both genus and ORF classification. Figure 1 shows a phylogenetic tree built with the genomic sequences used in the training sets. Notice that DNA-A and DNA-B are from the genus *Begomovirus*, i.e., both DNA-A and DNA-B sequences give rise to instances from this genus. The number of instances in each class, composing the training/test sets for family, genus and ORF classification, is shown in Additional file 1: Table S1. Additional file 2 shows the accession numbers of the complete genomes used to create the datasets.

### Data quality

The data available in public databases may contain non-standardized, non-curated sequences, with possible annotation errors, and, consequently, may be inappropriate to build training sets. The sequences used for the training and test sets should fit into the following criteria, which were established and implemented in www.geminivirus.org:

(i) The genomic sequences must start with the conserved 5′ end nucleotides (AC) of the Rep cleavage site;
(ii) the last seven nucleotides have to be the conserved sequence TAATATT that corresponds to the initial nucleotides of the replication origin TAATATTAC [43]. Notice that we standardized all genome sequences, which are circular, cutting them between TAATATT and AC;
(iii) the sequence length must be a value within an interval predefined for each genus (Table 1);
(iv) the ORFs must contain a start codon as well as a stop codon, and must not be truncated (no additional stop codon in between);
(v) ORF annotation errors, including wrong acronym as well as start and end positions, are corrected.

**Table 1:** Minimum and maximum sizes of each genus

Genus	Minimum size	Maximum size
*Begomovirus*	2411	2959
*Mastrevirus*	2425	2982
*Eragrovirus*	2845	2854
*Turncurtovirus*	3044	3081
*Curtovirus*	3011	3180
*Becurtovirus*	2939	2960
*Capulavirus*	2550	2872
*Grablovirus*	*3105*	*3205*
*Unclassified*	*2483*	*3308*
*Betasatellites*	731	1552
Alphasatellites	955	1579

In particular, the quality and reliability of the training instances generated from the already-mentioned taxonomic reviews have a high level of confidence, because they are manually curated by a specialized team. Such confidence is fundamental to create good datasets.

### Attribute extraction

The family *Geminiviridae* comprises plant virus species distributed across nine genera. Interestingly, the genomic organization is highly conserved among those genera. For example, the genes Rep (coded in the virion-complementary strand) and CP (coded in the virion-sense strand) are common to all genera, and their coordinates in different genomes are approximately equivalent regarding their replication origin [2]. Despite the high conservation of the genomic structure and particularities of the family *Geminiviridae*, we selected attributes common to any viral genome so that our considerations could be possibly used in other studies with different species involving the same kind of classification tasks.

**Fig. 2:**
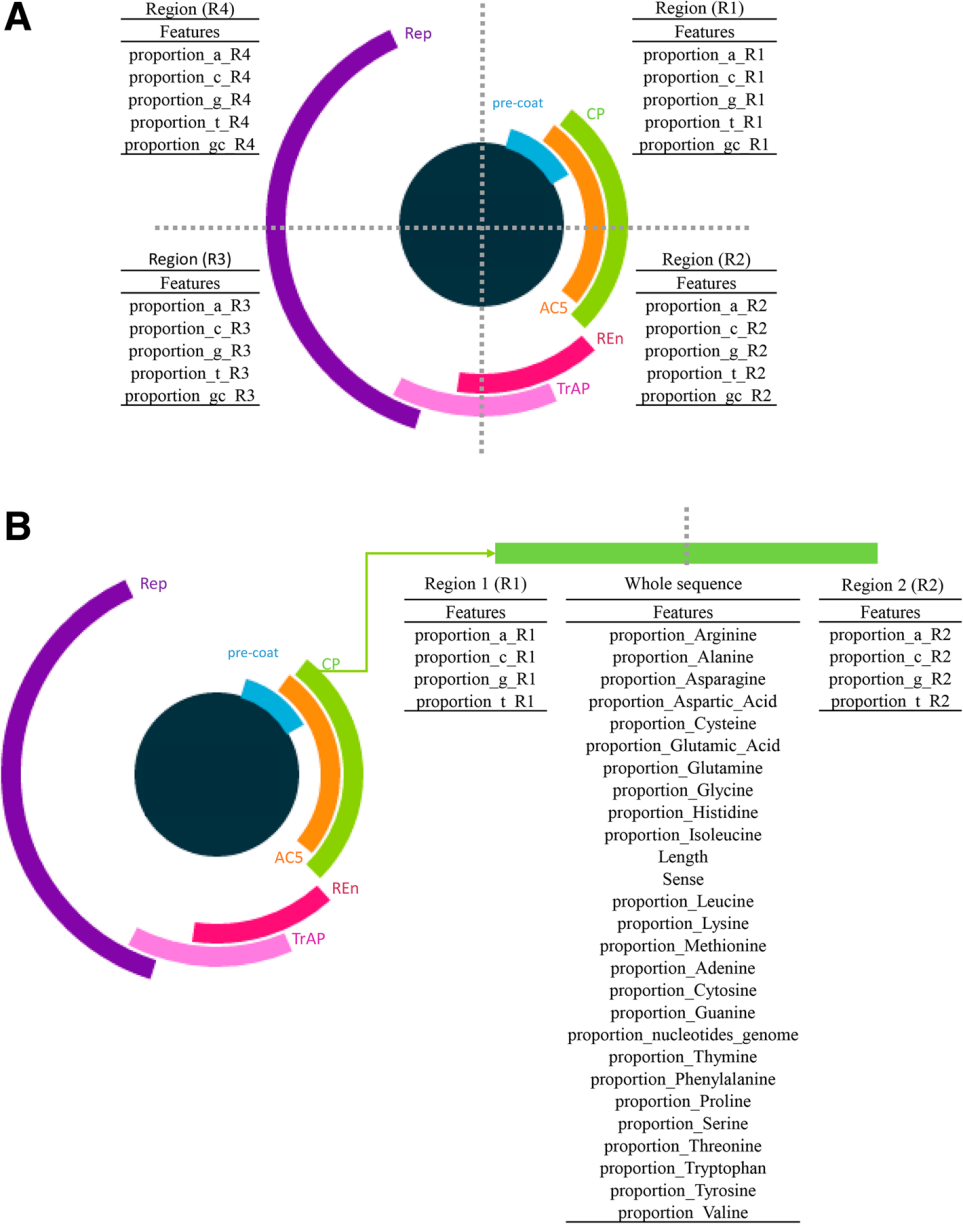
Attributes used for the classification tasks. **a** The circular genome is divided into four genomic regions of the same (or nearly same) size. For each region, the following attributes are extracted: proportion of adenine, thymine, cytosine, guanine, and GC content. **b** Each ORF contained in the genome is divided into two regions of equal (or nearly equal) size. Then, a series of attributes concerning the constituent nucleotides and amino acids of the translated sequence are considered in these regions and the whole ORF sequence

The attributes selected to build the family and genus classification models include the proportions of deoxynucleotides. Inspecting the complete genomic sequence, the proportions of adenine (A), thymine (T), cytosine (C), and guanine (G) are calculated. Next, the genomic sequence is split into four equal (or nearly equal) regions (R1, R2, R3, and R4) and, for each one, the proportions of A, T, C, and G as well as the GC content are calculated (Fig. 2a). As a result, we consider 24 attributes for classifying family and, genus, which are presented in Additional file 3: Table S2 and Additional file 4: Table S3, respectively.

To build the gene classification models, the attributes were obtained from each coding DNA sequence (CDS) and its respective amino acid sequence. First, attributes such as ORF orientation in the genome (forward/complement), CDS length, and proportion of nucleotides of the CDS in relation to the complete genome (CDS length/genome length) are extracted. Also, the A, T, C, and G proportions of the CDS itself are calculated. Moreover, the CDS is split into two equal (or nearly equal) regions and, for each of these regions, the proportions of A, T, C, and G are also considered. In addition to those attributes, the proportion of each of the 20 primary amino acids is obtained from the CDS translated sequence (Fig. 2b). Consequently, 35 attributes (see Additional file 5: Table S4) are taken into account.

### Attribute evaluation

Evaluating the attributes extracted from genomic sequences enables identifying which ones help differentiate one genus from another in the classification process. In the same way, measuring the relevance of ORF attributes enables verifying how such attributes contribute to the classification of genes.

Thus, in order to evaluate the importance of each attribute in the training sets, two ranking methods were used: information gain (IG) and RELIEFF [44, 45]. The IG method is based on the shannon entropy and is largely used in many bioinformatics studies [46, 47]. This method assesses the attributes by measuring the information gain they provide in relation to the class attribute. The IG method is defined by IG(*Attribute*) = *Entropy*(*Class*) - *Entropy*(*Class*|*Attribute*), where the entropy is given by -∑ *p*_*i*_ *log*_2_ *p*_*i*_, and *p*_*i*_ is the probability of class *i*.

RELIEFF is an extension of RELIEF [48]. RELIEF was coined for binary classification and builds a weight vector (*W*) of length *p* (the number of attributes) to represent the relevance of the attributes. This vector starts with zeros and is updated considering the attribute vector (*X*) of a random instance as well as the attribute vectors *H* and *M*, representing the closest instance of the same class (hit) and the closest instance of the other class (miss), respectively, using the following update formula:

$$ {w}_i={w}_i{\left({x}_i-{h}_i\right)}^2+{\left({x}_i-{m}_I\right)}^2 $$

Therefore, differences between *X* and *H* contribute to diminish the relevance of the attributes, while differences between *X* and *M* contribute to augment the weight of attributes. This process is repeated *m* times (for *m* sampled instances), and the final values in *W* are the average of all iterations (at the end, the values in *W* are divided by *m*). Kononenko proposed RELIEFF to overcome some issues of RELIEF [48]. The main improvements were that the update step is made for all instances, not for a sample; instead of taking only one neighbor of each class, *k* neighbors of each class are taken into account and their contribution is averaged; the algorithm adapts the calculation of *W* for multiple classes.

To complement the attribute analysis, descriptive statistics and exploratory data analysis were performed. Boxplots, histograms and density plots were created to visualize the distribution of attribute values in each class (Additional file 6).

### Defining candidate ORFs

**Fig. 3:**
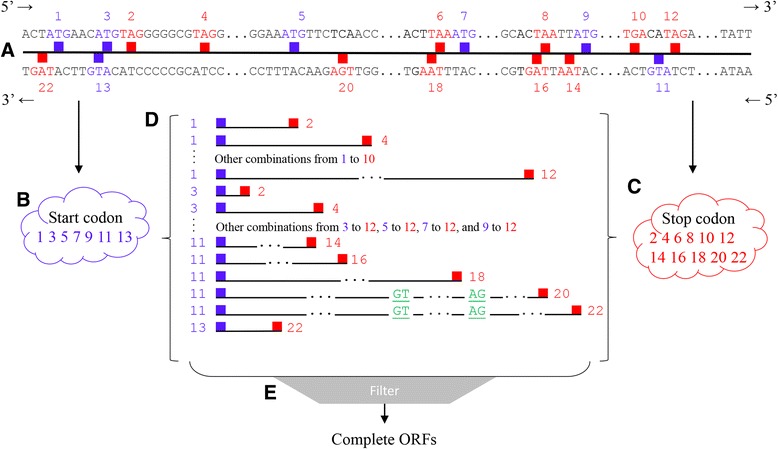
Schematic representation of the VM Algorithm. Initially, the user submits a putative genomic sequence (**a**). Then, the algorithm scans the full-length sequence identifying all initiation codons [ATG (5′ → 3′) and CAT (3′ → 5′)], which are highlighted in blue boxes and odd numbers, and stop codons [TAA, TAG, TGA (5′ → 3′) and TTA, CTA, TCA (3′ → 5′)], denoted in red and identified by even numbers. The initiation and stop codons are clustered separately and organized according to their numbering scheme (**b**, **e**, **c**). Each initiation codon is tested with all stop codons to verify whether each pair can form a full-length ORF (**d**). All possible splicing sites GT and AG are located in the ORF (highlighted in green). Filters are applied to evaluate the consistency of candidate ORFs and to certify that they are not truncated (**e**)

To predict genes using ML algorithms, we need first to extract candidate ORFs from the input sequence. To this end, we developed an algorithm based on a greedy approach implemented as part of the F2 method, hereby designated Viaduc de Millau (VM) (Fig. 3). Initially, the algorithm identifies all start codons [ATG (5′ → 3′) and CAT (3′ → 5′)] and the reading phase in the sense or anti-sense sequence. In the same way, all stop codons [TAA, TAG, TGA (5′ → 3′) and TTA, CTA, TCA (3′ → 5′)] are located. In addition, our procedure determines the coordinates where the start codon and stop codon are located in the genome. Each start codon of the sequence in a given sense is paired with stop codons in the same sense. Next, two steps are performed to check some requirements concerning the consistency of each possible ORF (in 5′ → 3′ or 3′ → 5′): (i) whether the sequence is in frame; and (ii) whether the translated amino acid sequence is not truncated, and has size greater or equal to 33 amino acids.

However, genes that code different splicing forms in the 3′ → 5′ orientation of genomic sequences of maize streak virus (MSV) have been reported in the family *Geminiviridae* [49]. In order to find such genes, an algorithm different from previously proposed procedures was performed. To find these ORFs, basic rules of the biological process of mRNA excision were employed in order to precisely identify splicing regions [50]. In this approach, the start and stop codons may or may not be in the same reading phase in the 3′ → 5′ sense. After obtaining sequences of possible ORFs in 3′ → 5′ containing start and stop codons in equal or different sense, the following steps are applied to check some basic requirements as well as typical characteristics of ORFs with introns in genimiviruses: (i) all stop codons in the 3′ → 5′ sense are inspected to verify whether their positions are greater than the position of the respective start codons; (ii) the existence of excision sites (CT and AC) is checked; (iii) each candidate CT excision site is paired with all possible AC sites; (iv) the sizes of the two exons (exon 1: minimum 204 nt and exon 2: minimum 148 nt) and the intron (minimum 67 nt, maximum 102 nt) are checked; (v) it is inspected whether the amount of pyrimidines is greater than the amount of purines at 50 pb upstream of the AC excision sites; (vi) the minimum length (1000 nt) of the ORF is verified and whether the sequences are in the correct reading phase; (vii) the reverse complement of the sequence is obtained, the candidate CDS is translated, and it is verified if it is not truncated. The restrictions to exon, intron, and sequence sizes were determined in view of the structure of the genes of this family, particularly *Mastrevirus*, which has an intron in the gene C1:C2 [49].

### Choosing the machine learning algorithm

The Fangorn Forest method embeds two ML models built with the previously described training sets. The genus model classifies complete genomes of the nine genera in the family *Geminiviridae* and related satellite DNAs, using 24 attributes. The ORF model was trained to classify genes of all the above types of genomes, using 35 attributes.

In this study, three ML algorithms were tested in order to select the one that suits the classification tasks: Sequential Minimal Optimization (SMO), Random Forest (RF), and Multilayer Perceptron (MLP). Those algorithms are implemented in the suite Weka v3.8.1 [51], whose API is used in our system. The experiments performed with those methods employed the Weka API using programs in the Java programming language.

The SMO algorithm is a largely used method to solve the quadratic programming problem upon which the SVM approach is based to find the maximum-margin hyperplane for separating two classes [52]. The RF algorithm is a classification method based on decision trees, which is able to perform regression and classification. The classification of a new instance occurs by the classification of multiple trees, resulting in a consensus of those classifications through a voting procedure (ensemble) [53].

The MLP algorithm is a type of neural network that is widely used for its high predictive power in non-linear systems. Several studies report the benefits of neural networks compared to traditional statistical modeling techniques [54]. MLP features three types of artificial neuron layers: an input layer, one or more hidden (or intermediate) layers, and an output layer. Each neuron in a layer may only connect to neurons in the subsequent layer (feedforward connections). Those connections have weights (calculated in the training procedure) that define how the input data values will be processed to generate the final output. Backpropagation is the most common learning (weight adjustment) method of MLPs [54].

Those ML algorithms were run with the Weka default parameters. The generality of the resulting models was evaluated using three different techniques: (i) the use of a completely independent test set, (ii) 10-fold cross validation, and (iii) leave-one-out (which is an *n*-fold cross validation, where *n* is the number of instances in the training set) [55, 56]. For each test, the following measures were obtained for evaluating the model performance: accuracy, precision, recall, *F*-measure, MCC (Matthews correlation coefficient) [57] (Additional file 7: Equation S1), and AUC [58]. After performing all tests, the *F*-measure (harmonic mean of precision and recall), MCC and AUC were analyzed to support our choice for the ML algorithm to be included in our system.

### Fangorn Forest method

**Fig. 4:**
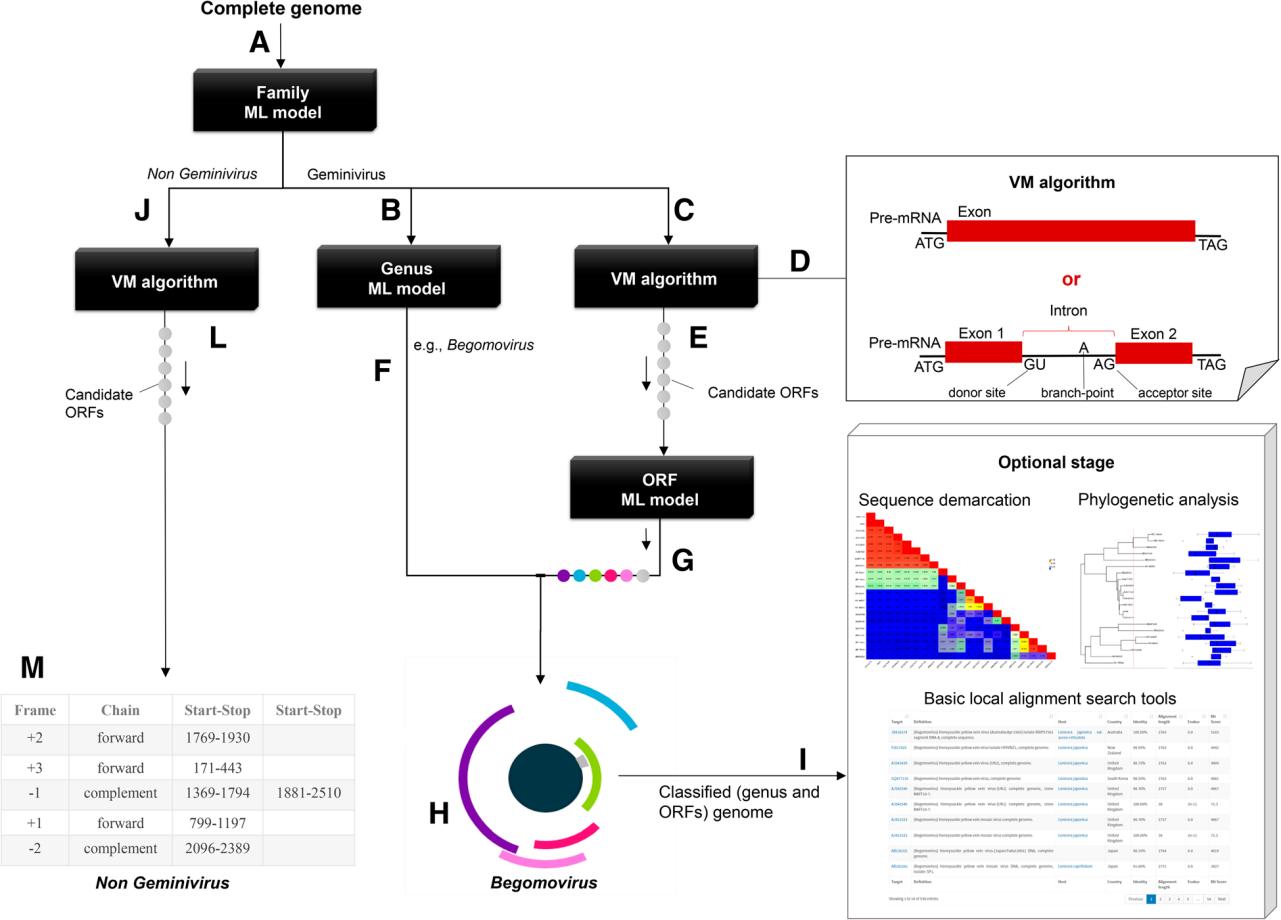
Flowchart of the Fangorn Forest method. First, the complete genome is given as input to the family classification model (**a**). If it is classified as a geminivirus the sequence is given as input for the genus classification model (**b**) and to the VM algorithm (**c**). This algorithm selects putative genes (ORFs) (**d**). These candidates are then given as input to the ORF classification model (**e**). Finally, the output of the genus model (**f**) and the output of the ORF model (**g**) are combined so that the virus genomic organization can be visualized (**h**). Additional analysis may be optionally performed (**i**). Based on the class determined by the genus model, a BLAST search with specific sequences may be performed. Furthermore, species demarcation analyses (SDT) and phylogenetic analyses may be carried out. If in the step A, the sequence is classified as non-geminivirus or if the replication origin is missing, the genomic sequence is given as input for the VM (**j**) algorithm. The result of the prediction (**l**) is presented in a table (**m**)

The Fangorn Forest method is composed of four fundamental parts: the family ML model, genus ML model, the VM algorithm, and the ORF ML model, as illustrated in Fig. 4. The family model classifies a complete genome as belonging to the Geminiviridae family (Fig. 4a). The genus model classifies a complete genome among eight genera of the family Geminiviridae as well as related satellite DNAs (alpha or beta satellite) (Fig. 4b). For gene prediction, the VM algorithm is first used to select candidate ORFs contained in the input genome, and, next, the ORF model classifies them within one of the classes: pre-coat, Reg, CP, AC5, REn, TrAP, Rep, Sd/p.sd, NSP, MP, alphaRep, and betaC1. Once those classifiers are executed, their results are combined to provide an interactive visualization of the genomic organization, similarly to the structures suggested by Varsani et al. [2]. Notice that the VM algorithm is not infallible, i.e., a spurious ORF might be given as input to the ORF model. F2 detects such cases by analyzing the probability distribution, across the twelve classes, yielded by the ORF model. If all probabilities are low (less than a predefined threshold – default: 0.8), then the putative ORF is marked as unknown (gray circle in Fig. 4f and gray piece in Fig. 4g). DNA sequence classified as belonging to the family *Geminiviridae* is verified by a filter for the existence of the replication origin of geminivirus, before being fed to the second model composed of 10 classes (Fig. 4b). If the origin of replication is not found, the sequence is not submitted to the genus and gene classification model but is submitted to the VM algorithm to predict ORFs and other analysis tools (Fig. 4j). The same procedures are taken for a genomic sequence classified as a non-geminivirus sequence in the first model (Fig. 4j). If a totally unraleted genome is submitted to the method, it will be classified as non-geminivirus.

Optionally, F2 allows additional analyses using the complete genomic sequence: (i) BLASTn with e-value 1*.*0E10^−5^, aiming to identify the closest species; (ii) phylogenetic reconstruction (BLASTn with e-value 1*.*0E10^−5^, sequence alignment with Muscle, tree building with FastTree [59], and phytools package for tree visualization [60]); and (iii) species demarcation using the SDT software.

## Results and discussion

The number of scientific studies on the family *Geminiviridae* has significantly increased in the last ten years (geminivirus.org:8080/geminivirusdw/statistics.jsp). The broad diversity of species, the large number of complete sequences, and the discovery of new geminiviruses have increased the complexity in determining the nomenclature and providing the taxonomic classification of geminiviruses [3, 30–32, 61–63]. Another issue in the family *Geminiviridae* concerns some particular genes in some species of the genus *Mastrevirus*, post-transcriptional changes may occur in primary gene transcripts, such as for MSV, whose genome holds gene C1:C2 [49]. Post-transcriptional processing of genes is common in eukaryotes and rare in prokaryotes. It occurs through a series of reactions catalyzed by the host spliceosome or self-splicing mechanisms [64]. The traditional tools to predict ORFs, such as ORF Finder, have not been adapted for the possibility of splicing. Other consolidated tools, such as AUGUSTUS, Geneid (both adapted for Eukaryote) and Prodigal (adapted for Prokaryotes), are still limited to identify all ORFs encoded by a given genome sequence of geminivirus species. These tools consider common features for organisms that have larger genomes with more complex promoters.

To mitigate all these issues, the present study developed the family and genus classification model along with the VM algorithm, for ORF extraction, associated with an ORF classification model so that a geminivirus genome sequence could be classified into one of genera in the *Geminiviridae* family, and the genes in this sequence could be easily identified. The results to validate our method are presented below. Notice that we do not provide here a comparison between methods, as, to our knowledge, there is no known approach, with similar intent, proposed specifically to geminiviruses, and that works in an ab initio manner (i.e., only the input sequence itself is analyzed). Thus, no homology analysis procedure, which is the usual approach in general, is used in our case.

### Attribute analysis results

Additional file 3: Tables S2, Additional file 4: Table S3 and Additional file 5: Table S4 show the results of the attribute analysis using IG and RELIEFF. Both methods agreed on the relevance of some top and low-ranked attributes, although the evaluation of many others attributes presented highly dissimilar rank positions comparing the outputs of those algorithms. Most importantly, none of the attributes presented null relevance in both ranks. In fact, we tried to remove some low-ranked attributes for all processes, family, genus and ORF model training. It turns out that all attempts to eliminate any of the attributes caused a decrease in performance of the resultant models

**Fig. 5:**
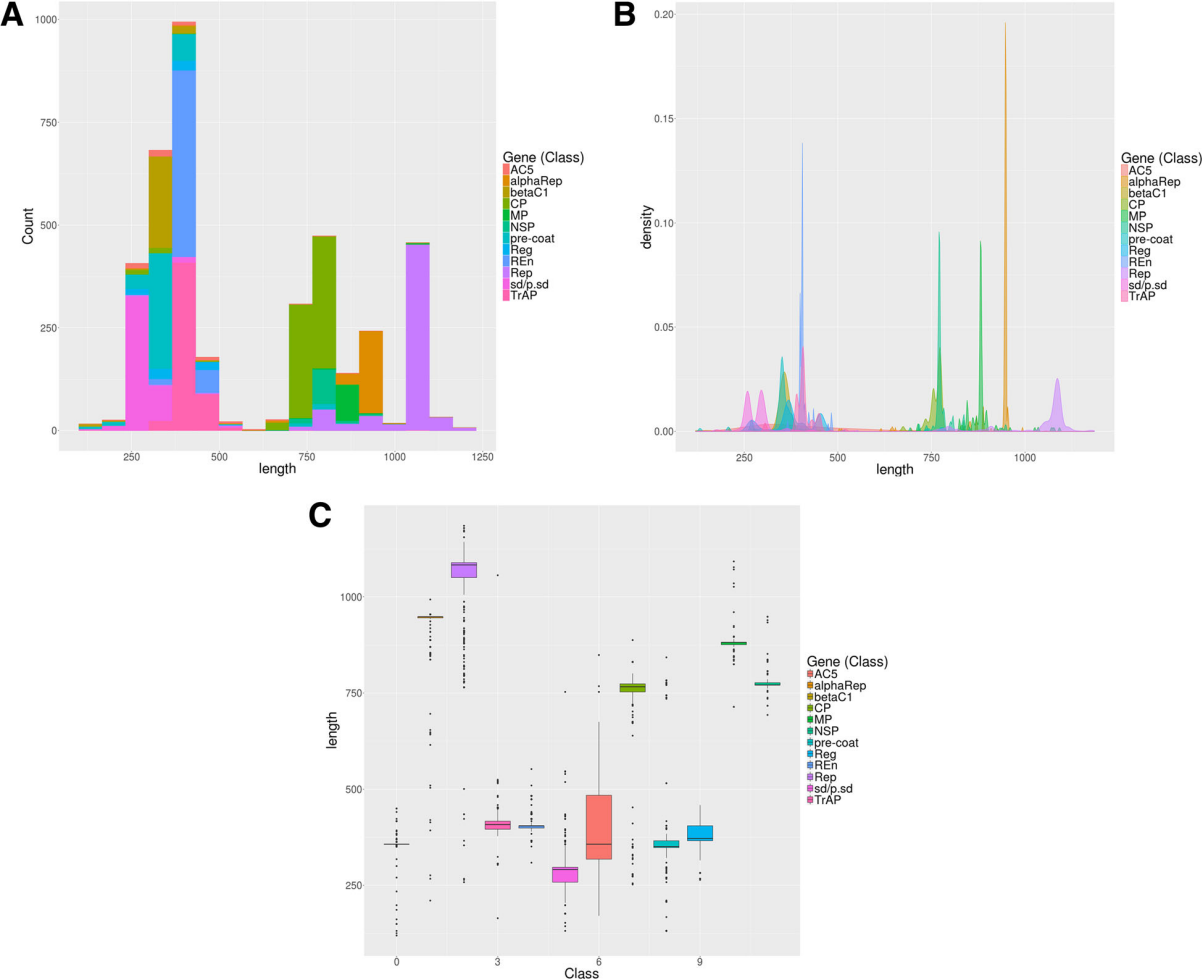
Exploratory analysis of the sequence length attribute. **a**) Histogram. **b** Density plot. **c** Boxplot

The relevance of all proposed attributes for building both models was corroborated by histograms, density plots and boxplots. An example is provided in Fig. 5 for the attribute ‘length’ used in ORF classification. The histogram and density plot demonstrate diverse distributions of that attribute across the classes. Additionally, the boxplot shows very distinct means and standard deviations of the same attribute when the classes are compared. Additional file 6 shows these plots for all attributes in both training sets (genus and ORF). The same conclusions about the distribution diversity across the classes can be drawn for the other attributes in both classification tasks. Based on these analyses, we decided to keep all proposed attributes in the training sets used to construct the F2 models.

### Performance of the ML models

Tables 2, 3 and 4 show the performance of the models for family, genus and ORF classification, which were built with MLP, SMO, and RF, using the default parameters of Weka (see Additional file 8: Table S5 for more details). It can be seen that MLP and RF are superior than SMO for genus classification. For ORF classification, on the other hand, all methods performed well. Inspecting the *F*-measure, it is difficult to choose between MLP and RF. MLP was slightly better for genus classification, while RF presented slightly superior values for ORF classification. However, based on the results shown in Tables 2, 3 and 4, we chose RF as the classifier for both genus and ORF for two reasons: (i) RF presented the greatest AUC value in all tests for both classification tasks, which means more coherent output probabilities; and (ii) the training time for RF is much shorter compared with the other methods.

**Table 2:** Performance of the family classification model using default parameters of Weka

Type of evaluation	ML algorithm	Weighted average among all classes					
		Accuracy	Precision	Recall	F-Measure	MCC	AUC
Using a test set	MLP	0.9444	0.946	0.944	0.944	0.891	0.969
	SMO	0.8107	0.815	0.811	0.810	0.625	0.810
	RF	0.9542	0.955	0.954	0.954	0.909	0.988
10-fold cross validation	MLP	0.9369	0.937	0.937	0.937	0.871	0.972
	SMO	0.8568	0.861	0.857	0.855	0.709	0.844
	RF	0.9601	0.960	0.960	0.960	0.919	0.992
Leave-one-out	MLP	0.944	0.944	0.944	0.944	0.886	0.975
	SMO	85.597	0.860	0.856	0.854	0.707	0.843
	RF	96.228	0.963	0.962	0.962	0.923	0.992
Mean performance	MLP	0.9420	0.9430	0.9417	0.9417	0.8843	0.9700
	SMO	0.8411	0.8433	0.8413	0.8339	0.6803	0.8323
	RF	0.9588	0.9533	0.9586	0.9586	0.917	0.9906

**Table 3:** Performance of the genus classification model using default parameters of Weka

Type of evaluation	ML algorithm	Weighted average among all classes					
		Accuracy	Precision	Recall	F-Measure	MCC	AUC
Using a test set	MLP	0.941	0.963	0.941	0.951	0.8940	0.971
	SMO	0.835	0.865	0.835	0.795	0.6340	0.816
	RF	0.934	0.941	0.934	0.936	0.8750	0.988
10-fold cross validation	MLP	0.970	0.970	0.971	0.970	0.9610	0.991
	SMO	0.920	0.901	0.920	0.906	0.8850	0.962
	RF	0.966	0.966	0.966	0.965	0.9510	0.997
Leave-one-out	MLP	0,971	0,971	0,972	0,960	0.9920	0.995
	SMO	0.944	0.938	0.945	0.939	0.8810	0.946
	RF	0.991	0.991	0.991	0.991	0.9550	0.999
Mean performance	MLP	0.966	0.974	0.967	0.970	0.9490	0.986
	SMO	0.900	0.901	0.900	0.880	0.8800	0.908
	RF	0.964	0.966	0.964	0.964	0.9238	0.995

**Table 4:** Performance of the gene classification model using default parameters of Weka

Type of evaluation	ML algorithm	Weighted average among all classes					
		Accuracy	Precision	Recall	F-Measure	MCC	AUC
Using a test set	MLP	0.972	0.973	0.973	0.972	0.968	0.985
	SMO	0.976	0.977	0.976	0.976	0.973	0.995
	RF	0.981	0.982	0.982	0.982	0.979	0.998
10-fold cross validation	MLP	0.970	0.971	0.971	0.971	0.967	0.994
	SMO	0.972	0.973	0.973	0.973	0.969	0.994
	RF	0.976	0.977	0.977	0.977	0.974	0.997
Leave-one-out	MLP	0.970	0.970	0.970	0.970	0.966	0.994
	SMO	0.9727	0.973	0.973	0.973	0.969	0.994
	RF	0.9759	0.976	0.976	0.976	0.973	0.997
Mean performance	MLP	0.9707	0.9713	0.9713	0.9710	0.9670	0.9910
	SMO	0.9736	0.9743	0.9740	0.9740	0.9703	0.9943
	RF	0.9776	0.9783	0.9783	0.9783	0.9753	0.9973

Most importantly, RF demonstrated a very high prediction power. For the classification model of the family *Geminividae*, the RF algorithm achieves mean performance of 0.9588. 0.9533, 0.9586, 0.9586, 0.917, 0.9906 of accuracy, precision, recall, F-measure, MCC and AUC, respectively. The mean performance of RF for genus classification was 0.964, 0.966. 0.964, 0.964, 0.923, 0.995 of accuracy, precision, recall, F-measure, MCC and AUC, respectively. For ORF classification, RF achieved the mean values 0.977, 0.978, 0.978, 0.978, 0.975, 0.997 of accuracy, precision, recall, F-measure, MCC and AUC, respectively.

To evaluate the overall pipeline, a set of sequences of plant viruses, sequences from the *Circoviridae* Family (circular single-stranded DNA animal virus) and artificially generated sequences were submitted manually to the web interface of the pipeline method. The method was adjusted with the threshold of 0.5 (default) for family and ORF classifications. In this test, F2 achieved accuracy, precision, recall and F-Measure of 0.9343, 0.9343, 0.9343, and 0.9343, respectively, for the correct identification of genomes of non-geminiviruses or geminiviruses and their genus (Additional file 9). In addition, a partial begomovirus sequence (EF591125-begomovirus), which does not encode a protein, was not classified as geminivirus. Likewise, a defective KT099181 sequence of betasatellites was not cataloged as a geminivirus-related DNA satellite. These examples demonstrated that defective begomovirus genomes, which did not display the genomic structure of geminivirus were not recognized as geminiviruses.

Some geminivirus genomes exhibit considerable similarity with the genomic structure of different families of ssDNA viruses (i.e. circoviruses and parvoviruses) (Additional file 9). Thus, genomic sequences of Family Circovidae and Parvoviridae were confronted to F2 and three of 20 sequences were classified as geminiviruses with low probability. Furthermore, the predicted ORFs were not classified as geminivirus ORFs within the established limit as default. Random sequences with geminivirus origin of replication were created and compared against the F2 method. Neither of these sequences were classified as geminiviruses nor the predicted ORFs were classified as geminiviral ORFs.

In addition to predicting family and gender, the F2 method can predict ORFs and classify sequences of geminivirus-specific ORFs (genes). Some species encode two to seven genes only in the component A. Most sequences are short and important to complete the infectious cycle of the virus. Like the ORF finder, some other tools can identify ORFs; however, they did not identify introns and hence they fail to annotate some genes. The AUGUSTUS tool is widely consolidated and widely used in genome projects to perform a prediction of eukaryotic genes. We confronted AUGUSTUS and ORF finder by performing a gene prediction for the most common begomovirus sequences (AF416742, AF448058, AF241479, AF126406, DQ026296). For each of these sequences, the AUGUSTUS algorithm only identified two ORFs, whereas these genomes encode six to seven genes. Mastervirus sequences (KY618115, KF806701, KJ187748, KC172663, HQ113104) were also used, however, few genes and no introns were identified. The ORF finder identified almost all geminivirus genes, except the ones with introns. The methodology proposed by the F2 method can complement these tools, as it is efficient to annotate all geminivirus genes.

## Conclusion

*Geminiviridae* is an important plant virus family, as it represents a serious threat to agriculture and food security. Identifying genera of this family requires caution and has become a challenge due to a large number of sequences available in databases. Moreover, advanced knowledge in taxonomy and bioinformatics analyses is currently required.

As a result of this research, a new method based on machine learning techniques, called Fangorn Forest, is proposed to automatize the identification of genera and genes of the family *Geminiviridae*. This method is composed of four fundamental parts. The family and genus classification module is able to classify a complete genome within one of eight genera of the family *Geminiviridae* or associated satellite DNAs (alpha or beta satellite). Another important component is the algorithm for ORF identification, called here Viaduc de Millau (VM), created for the specific peculiarities of the family *Geminiviridae*, which are not covered by other general-purpose ORF predictors, such as ORF finder. VM is used in conjunction with the third important part of our system. This is the ORF classification procedure that classifies the ORFs extracted by VM according to the typical gene types encountered in geminiviruses. Both classifiers, for genus and ORF, are highly accurate, as could be seen in the presented results.

It is also worth mentioning the additional stages that can be performed with the input sequence. Our system may optionally use the SDT tool for species-demarcation, and perform phylogenetic analyses, which greatly facilitate the study under consideration. To this purpose, F2 is adapted to act autonomously based on the genus classification, whose result redirects the analysis to specific databases for the identified genus, so that an appropriate set of sequences can be used to perform the analyses.

We stress the importance of automatizing genus and ORF classification, with high accuracy, with a special focus on geminiviruses, resulting in a powerful customized system for this type of virus that causes expressive economic impacts. The method is freely accessible at http://geminivirus.org:8080/geminivirusdw/discoveryGeminivirus.jsp.

## Additional files

Additional file 1: Table S1. Number of instances (sequences) of each family, genus, and respective ORFs, contained in the datasets. (DOC 78 kb)

Additional file 2: This file presents the accession numbers of the complete genomic sequences that make up the training and test sets. (XLS 1650 kb)

Additional file 3: Table S2. The IG, RELIEFF ranks of attributes in the family training set. Attributes are sorted by the IG rank. (DOC 53 kb)

Additional file 4: Table S3. The IG, RELIEFF ranks of attributes in the genus training set. Attributes are sorted by the IG rank. (DOC 52 kb)

Additional file 5: Table S4. The IG, RELIEFF ranks of attributes in the ORF training set. Attributes are sorted by the IG rank. (DOC 62 kb)

Additional file 6: This file shows plots (histogram, density and boxplot) related to the attributes of the ORF and genus training sets. (PDF 8536 kb)

Additional file 7: Equations S1. Model assessment measures. (DOCX 16 kb)

Additional file 8: Table S5. Performance results of the tests (performed with the Weka) for the models of family, genus and gene classification. (DOC 147 kb)

Additional file 9: This file presents the pipeline evaluation with geminiviruses sequences, plant viruses, circoviruses and fake sequences. (XLS 44 kb)

## Abbreviations

BGMD: Bean golden mosaic disease
CDS: Coding DNA sequence
CLCuD: Cotton leaf curl disease
CMD: Cassava mosaic disease
F2: Fangorn forest
IG: Information gain
ML: Machine learning
MLP: Multilayer perceptron
MP: Movement protein
MSD: Maize streak disease
MSV: Maize streak virus
NSP: Nuclear transport protein
ORFs: Open reading frames
RCA: Rolling circle amplification
RF: Random forest
SDT: Species demarcation analyses
SMO: Sequential minimal optimization
SVM: Support vector machine
TYLCD: Tomato yellow leaf curl virus
UTR: Untranslated region
VM: *Viaduc de Millau*
WDVD: Wheat dwarf virus disease
