# Supplementary material for: Fangorn Forest (F2): a machine learning approach to classify genes and genera in the family Geminiviridae
Source: BMC Bioinformatics. 2017 Sep 30;18:431. doi: 10.1186/s12859-017-1839-x (PMC5622471; doi:10.1186/s12859-017-1839-x)
Supplement: Supplementary file 1 — Number of instances (sequences) of each family, genus, and respective ORFs, contained in the datasets. (DOC 78 kb) [file 12859_2017_1839_MOESM1_ESM.doc]

**Supplementary Table S1. Number of instances/sequences of each family, genus, and respective ORFs, contained in the datasets.**

| **Dataset** | **Class** | **Number of instances** |  |
| --- | --- | --- | --- |
| Family training set | Non-geminivirus | 1000 |  |
|  | Geminivirus | 1333 |  |
|  | **Total** | **2333** |  |
| Family test set | Non-geminivirus | 9000 |  |
|  | Geminivirus | 9188 |  |
|  | **Total** | **18188** |  |
| Genus training set | Betasatellites | 258 |  |
|  | alphasatellites | 302 |  |
|  | Becurtovirus | 23 |  |
|  | Curtoviruses | 18 |  |
|  | Eragroviruses | 4 |  |
|  | Mastreviruses | 57 |  |
|  | Topocuviruses | 1 |  |
|  | Turncurtoviruses | 20 |  |
|  | Begomovirus (DNA-A /DNA-B) | 602 |  |
|  | Capulaviruses | 17 |  |
|  | Grabloviruses | 31 |  |
|  | **Total** | **1333** |  |
| Genus test set | Betasatellites | 986 |  |
|  | alphasatellites | 303 |  |
|  | Becurtovirus | 10 |  |
|  | Curtoviruses | 95 |  |
|  | Eragroviruses | 2 |  |
|  | Mastreviruses | 1524 |  |
|  | Topocuviruses | 0 |  |
|  | Turncurtoviruses | 27 |  |
|  | Begomovirus (DNA-A /DNA-B) | 6216 |  |
|  | Capulaviruses | 9 |  |
|  | Grabloviruses | 16 |  |
|  | **Total** | **9188** |  |
| ORF training set | betaC1 | 251 |  |
|  | alphaRep | 240 |  |
|  | Rep | 631 |  |
|  | TrAP | 531 |  |
|  | REn | 525 |  |
|  | sd/p.sd | 447 |  |
|  | AC5 | 54 |  |
|  | CP | 644 |  |
|  | pre-coat | 421 |  |
|  | Reg | 84 |  |
|  | MP | 101 |  |
|  | NSP | 106 |  |
|  | **Total** | **4035** |  |
| ORF test set | betaC1 | 872 |  |
|  | alphaRep | 249 |  |
|  | Rep | 6435 |  |
|  | TrAP | 4428 |  |
|  | REn | 4491 |  |
|  | sd/p.sd | 4377 |  |
|  | AC5 | 724 |  |
|  | CP | 6330 |  |
|  | pre-coat | 3840 |  |
|  | Reg | 171 |  |
|  | MP | 1002 |  |
|  | NSP | 999 |  |
|  | **Total** | **33918** |  |
|  |  |  |  |
