# Supplementary material for: Fangorn Forest (F2): a machine learning approach to classify genes and genera in the family Geminiviridae
Source: BMC Bioinformatics. 2017 Sep 30;18:431. doi: 10.1186/s12859-017-1839-x (PMC5622471; doi:10.1186/s12859-017-1839-x)
Supplement: Supplementary file 3 — The IG, RELIEFF ranks of attributes in the family training set. Attributes are sorted by the IG rank. (DOC 53 kb) [file 12859_2017_1839_MOESM3_ESM.doc]

**Supplementary Table S2. IG, RELIEFF ranks of attributes in the family training set. Attributes are sorted by the IG rank.**

| **Attribute** | **IG  value/rank** | **RELIEFF  value/rank** |
| --- | --- | --- |
| Proportion of thymine in the genome | 0.4868 (1°) | 0.0389 (3°) |
| Proportion of thymine in region 4 | 0.292 (2°) | 0.0288 (8°) |
| Proportion of thymine in region 1 | 0.2615 (3°) | 0.0293 (5°) |
| Proportion of thymine in region 2 | 0.2615 (4°) | 0.0293 (7°) |
| Proportion of thymine in region 3 | 0.2615 (5°) | 0.0293 (6°) |
| Proportion of adenine in the genome | 0.2575 (6°) | 0.0703 (1°) |
| Proportion of adenine in region 4 | 0.2433 (7°) | 0.0247 (9°) |
| Proportion of adenine in region 2 | 0.2384 (8°) | 0.022 (12°) |
| Proportion of adenine in region 1 | 0.2384 (9°) | 0.022 (11°) |
| Proportion of adenine in region 3 | 0.2384 (10°) | 0.022 (10°) |
| Proportion of cytosine in the genome | 0.1946 (11°) | 0.0437 (2°) |
| Proportion of guanine in the genome | 0.1615 (12°) | 0.0309 (4°) |
| Proportion of guanine in region 4 | 0.1411 (13°) | 0.0199 (16°) |
| Proportion of guanine in region 1 | 0.1357 (14°) | 0.0215 (13°) |
| Proportion of guanine in region 2 | 0.1357 (15°) | 0.0215 (14°) |
| Proportion of guanine in region 3 | 0.1357 (16°) | 0.0215 (15°) |
| Proportion of cytosine in region 4 | 0.0874 (17°) | 0.0188 (17°) |
| Proportion of cytosine in region 3 | 0.0817 (18°) | 0.0153 (20°) |
| Proportion of cytosine in region 2 | 0.0817 (19°) | 0.0153 (18°) |
| Proportion of cytosine in region 1 | 0.0817 (20°) | 0.0153 (19°) |
| Proportion of guanine and cytosine in region 4 | 0.0509 (21°) | 0.0114 (24°) |
| Proportion of guanine and cytosine in region 2 | 0.0505 (22°) | 0.0115 (21°) |
| Proportion of guanine and cytosine in region 3 | 0.0505 (23°) | 0.0115 (23°) |
| Proportion of guanine and cytosine in region 1 | 0.0505 (24°) | 0.0115 (22°) |
