# Supplementary material for: Fangorn Forest (F2): a machine learning approach to classify genes and genera in the family Geminiviridae
Source: BMC Bioinformatics. 2017 Sep 30;18:431. doi: 10.1186/s12859-017-1839-x (PMC5622471; doi:10.1186/s12859-017-1839-x)
Supplement: Supplementary file 4 — The IG, RELIEFF ranks of attributes in the genus training set. Attributes are sorted by the IG rank. (DOC 52 kb) [file 12859_2017_1839_MOESM4_ESM.doc]

**Supplementary Table S3. IG, RELIEFF ranks of attributes in the genus training set. Attributes are sorted by the IG rank.**

| **Attribute** | **IG  value/rank** | **RELIEFF  value/rank** |
| --- | --- | --- |
| Proportion of adenine in the genome | 1.249 (1°) | 0.1586 (1°) |
| Proportion of cytosine in region 2 | 1.137 (2°) | 0.1203 (4°) |
| Proportion of cytosine in region 1 | 1.137 (3°) | 0.1203 (3°) |
| Proportion of cytosine in region 3 | 1.137 (4°) | 0.1203 (5°) |
| Proportion of cytosine in region 4 | 1.112 (5°) | 0.1201 (6°) |
| Proportion of guanine and cytosine in region 4 | 0.981 (6°) | 0.0760 (20°) |
| Proportion of guanine and cytosine in region 2 | 0.964 (7°) | 0.0763 (18°) |
| Proportion of guanine and cytosine in region 1 | 0.964 (8°) | 0.0763 (17°) |
| Proportion of guanine and cytosine in region 3 | 0.964 (9°) | 0.0763 (19°) |
| Proportion of cytosine in the genome | 0.887 (10°) | 0.1248 (2°) |
| Proportion of adenine in region 4 | 0.787 (11°) | 0.0800 (15°) |
| Proportion of adenine in region 1 | 0.753 (12°) | 0.0810 (13°) |
| Proportion of adenine in region 2 | 0.753 (13°) | 0.0810 (14°) |
| Proportion of adenine in region 3 | 0.753 (14°) | 0.0810 (12°) |
| Proportion of guanine in the genome | 0.699 (15°) | 0.0789 (16°) |
| Proportion of thymine in region 3 | 0.656 (16°) | 0.0841 (7°) |
| Proportion of thymine in region 2 | 0.656 (17°) | 0.0841 (9°) |
| Proportion of thymine in region 1 | 0.656 (18°) | 0.0841 (10°) |
| Proportion of thymine in region 4 | 0.629 (19°) | 0.0835 (11°) |
| Proportion of guanine in region 1 | 0.588 (20°) | 0.0754 (23°) |
| Proportion of guanine in region 3 | 0.588 (21°) | 0.0754 (22°) |
| Proportion of guanine in region 2 | 0.588 (22°) | 0.0754 (24°) |
| Proportion of guanine in region 4 | 0.587 (23°) | 0.0755 (21°) |
| Proportion of thymine in the genome | 0.472 (24°) | 0.1034 (6°) |
