# Supplementary material for: Fangorn Forest (F2): a machine learning approach to classify genes and genera in the family Geminiviridae
Source: BMC Bioinformatics. 2017 Sep 30;18:431. doi: 10.1186/s12859-017-1839-x (PMC5622471; doi:10.1186/s12859-017-1839-x)
Supplement: Supplementary file 5 — The IG, RELIEFF ranks of attributes in the ORF training set. Attributes are sorted by the IG rank. (DOC 62 kb) [file 12859_2017_1839_MOESM5_ESM.doc]

**Supplementary Table S4. IG, RELIEFF ranks of attributes in the ORF training set. Attributes are sorted by the IG rank.**

| **Attribute** | **IG  value/rank** | **RELIEFF**  **value/rank** |
| --- | --- | --- |
| Length | 2.53571 (1°) | 0.2539 (2°) |
| Proportion of nucleotides regarding the genome (ORF_size / genome_size) | 2.38483 (2°) | 0.1695 (3°) |
| Proportion of Phenylalanine | 2.35723 (3°) | 0.1072 (7°) |
| Proportion of Tryptophan | 2.35522 (4°) | 0.1098 (5°) |
| Proportion of Tyrosine | 2.35098 (5°) | 0.1035 (9°) |
| Proportion of Cysteine | 2.35064 (6°) | 0.1066 (8°) |
| Proportion of Histidine | 2.34051 (7°) | 0.0767 (22°) |
| Proportion of Proline | 2.25308 (8°) | 0.1020 (10°) |
| Proportion of Glutamic_acid | 2.20959 (9°) | 0.1015 (11°) |
| Proportion of Isoleucine | 2.19040 (10°) | 0.0855 (14°) |
| Proportion of Aspartic_acid | 2.17441 (11°) | 0.0914 (13°) |
| Proportion of Valine | 2.11748 (12°) | 0.1177 (4°) |
| Proportion of Glutamine | 2.11366 (13°) | 0.0807 (18°) |
| Proportion of Lysine | 2.11276 (14°) | 0.0804 (19°) |
| Proportion of Leucine | 2.09200 (15°) | 0.0492 (30°) |
| Proportion of Alanine | 2.09200 (16°) | 0.0492 (32°) |
| Proportion of Methionine | 2.09200 (17°) | 0.0492 (31°) |
| Proportion of Asparagine | 2.02454 (18°) | 0.0776 (21°) |
| Proportion of Glycine | 2.00749 (19°) | 0.0606 (27°) |
| Proportion of Threonine | 1.98814 (20°) | 0.0847 (15°) |
| Proportion of Arginine | 1.89853 (21°) | 0.0944 (12°) |
| Proportion of Serine | 1.65532 (22°) | 0.1078 (6°) |
| Proportion of thymine in region 2 | 1.14179 (23°) | 0.0599 (28°) |
| Proportion of thymine in region 1 | 1.11190 (24°) | 0.0609 (26°) |
| Sense | 0.93206 (25°) | 0.5158 (1°) |
| Proportion of guanine in region 2 | 0.93082 (26°) | 0.0837 (17°) |
| Proportion of guanine in region 1 | 0.89886 (27°) | 0.0839 (16°) |
| Proportion of thymine | 0.89410 (28°) | 0.0552 (29°) |
| Proportion of cytosine in region 2 | 0.89249 (29°) | 0.0634 (24°) |
| Proportion of cytosine | 0.85924 (30°) | 0.0784 (20°) |
| Proportion of adenine in region 1 | 0.80036 (31°) | 0.0433 (34°) |
| Proportion of cytosine in region 1 | 0.80017 (32°) | 0.0634 (25°) |
| Proportion of adenine | 0.77133 (33°) | 0.0398 (35°) |
| Proportion of adenine in region 2 | 0.74682 (34°) | 0.0437 (33°) |
| Proportion of guanine | 0.72526 (35°) | 0.0659 (23°) |
