# Supplementary material for: Fangorn Forest (F2): a machine learning approach to classify genes and genera in the family Geminiviridae
Source: BMC Bioinformatics. 2017 Sep 30;18:431. doi: 10.1186/s12859-017-1839-x (PMC5622471; doi:10.1186/s12859-017-1839-x)
Supplement: Supplementary file 6 — This file shows plots (histogram, density and boxplot) related to the attributes of the ORF and genus training sets. (PDF 8536 kb) [file 12859_2017_1839_MOESM6_ESM.pdf]

Charts of genus and ORF attributes

- Family attributes
  - Proportion of Guanine in the genome
  - Proportion of Guanine in region 1
  - Proportion of Guanine in region 2
  - Proportion of Guanine in region 3
  - Proportion of Guanine in region 4
  - Proportion of Adenine in the genome
  - Proportion of Adenine in region 1
  - Proportion of Adenine in region 2
  - Proportion of Adenine in region 3
  - Proportion of Adenine in region 4
  - Proportion of Thymine in the genome
  - Proportion of Thymine in region 1
  - Proportion of Thymine in region 2
  - Proportion of Thymine in region 3
  - Proportion of Thymine in region 4
  - Proportion of Cytosine in the genome
  - Proportion of Cytosine in region 1
  - Proportion of Cytosine in region 2
  - Proportion of Cytosine in region 3
  - Proportion of Cytosine in region 4
  - Proportion of Guanine and Cytosine in region 1
  - Proportion of Guanine and Cytosine in region 2
  - Proportion of Guanine and Cytosine in region 3
  - Proportion of Guanine and Cytosine in region 4
- Genus attributes
  - Proportion of Guanine in the genome
  - Proportion of Guanine in region 1
  - Proportion of Guanine in region 2
  - Proportion of Guanine in region 3
  - Proportion of Guanine in region 4
  - Proportion of Adenine in the genome
  - Proportion of Adenine in region 1
  - Proportion of Adenine in region 2
  - Proportion of Adenine in region 3
  - Proportion of Adenine in region 4
  - Proportion of Thymine in the genome
  - Proportion of Thymine in region 1
  - Proportion of Thymine in region 2
  - Proportion of Thymine in region 3
  - Proportion of Thymine in region 4
  - Proportion of Cytosine in the genome
  - Proportion of Cytosine in region 1
  - Proportion of Cytosine in region 2
  - Proportion of Cytosine in region 3
  - Proportion of Cytosine in region 4
  - Proportion of Guanine and Cytosine in region 1
  - Proportion of Guanine and Cytosine in region 2
  - Proportion of Guanine and Cytosine in region 3
  - Proportion of Guanine and Cytosine in region 4
- ORF attributes
  - Proportion of Arginine
  - Proportion of Alanine
  - Proportion of Asparagine
  - Proportion of Aspartic Acid
  - Proportion of Cysteine
  - Proportion of Glutamic Acid
  - Proportion of Glutamine
  - Proportion of Glycine
  - Proportion of Histidine

- Proportion of Isoleucine
- Length
- Sense
- Proportion of Leucine
- Proportion of Lysine
- Proportion of Methionine
- Proportion of Adenine
- Proportion of Adenine in region 1
- Proportion of Adenine in region 2
- Proportion of Cytosine
- Proportion of Cytosine in region 1
- Proportion of Cytosine in region 2
- Proportion of Guanine
- Proportion of Guanine in region 1
- Proportion of Guanine in region 2
- Proportion of nucleotides regarding the genome (ORF_size / genome_size)
- Proportion of Thymine
- Proportion of Thymine in region 1
- Proportion of Thymine in region 2
- Proportion of Phenylalanine
- Proportion of Proline
- Proportion of Serine
- Proportion of Threonine
- Proportion of Tryptophan
- Proportion of Tyrosine
- Proportion of Valine

Charts of Family attributes

Proportion of Guanine in the genome

Histogram

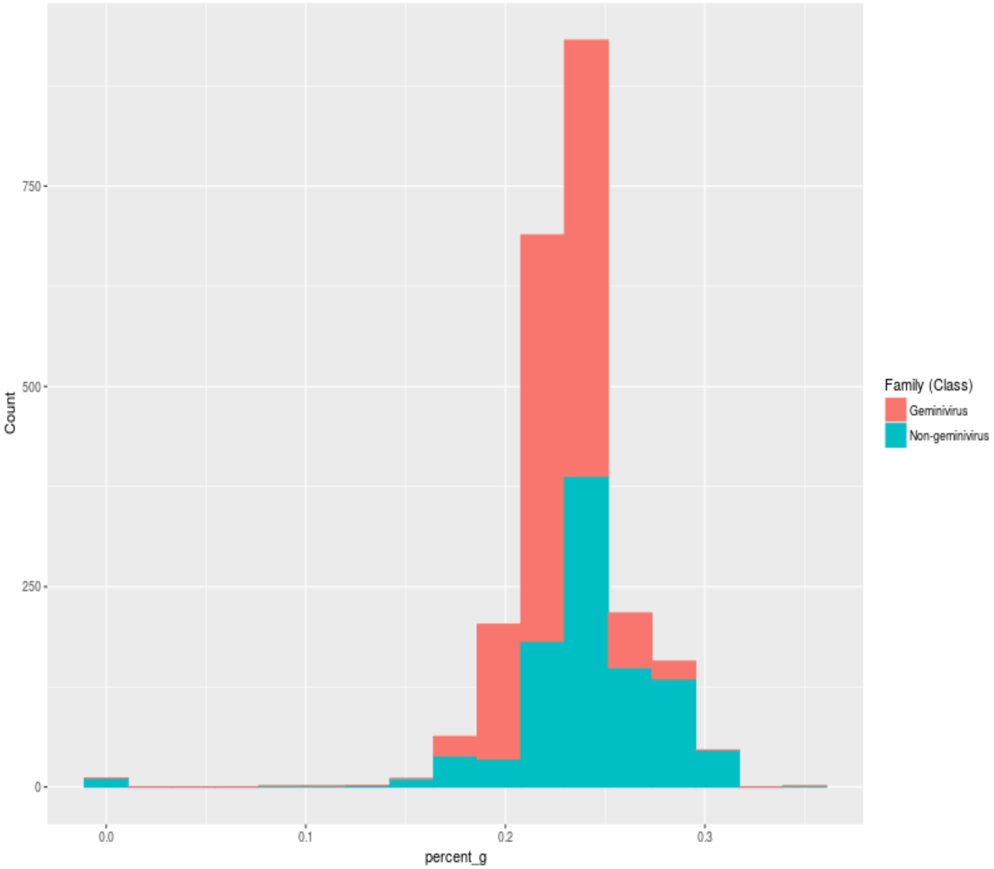

Density

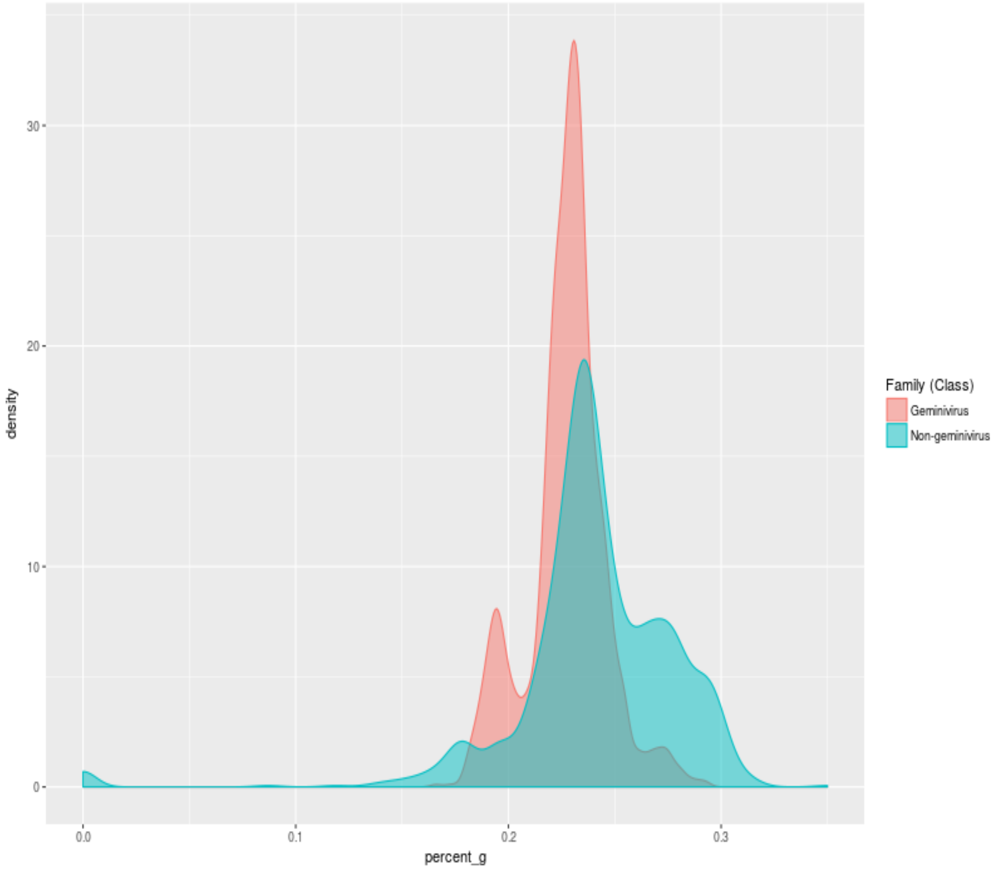

Boxplots

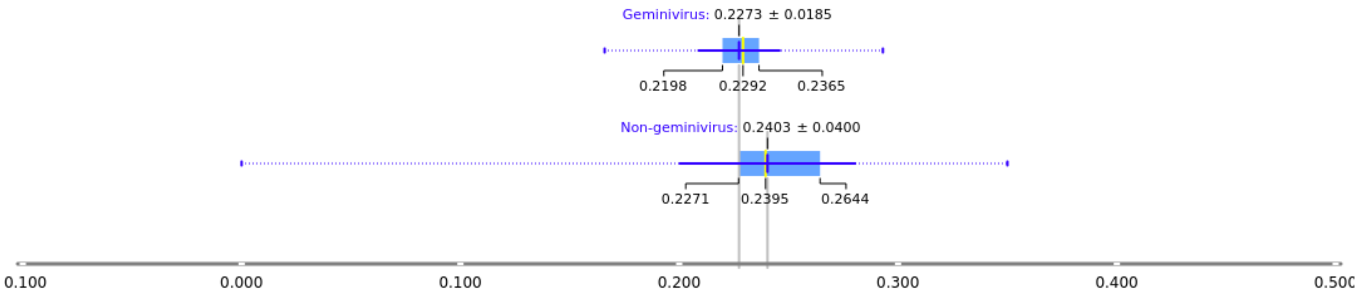

Geminivirus: 0.2273 ± 0.0185
0.2198
0.2292
0.2365
Non-geminivirus: 0.2403 ± 0.0400
0.2271
0.2395
0.2644
0.100
0.000
0.100
0.200
0.300
0.400
0.500

Proportion of Guanine in region 1

Histogram

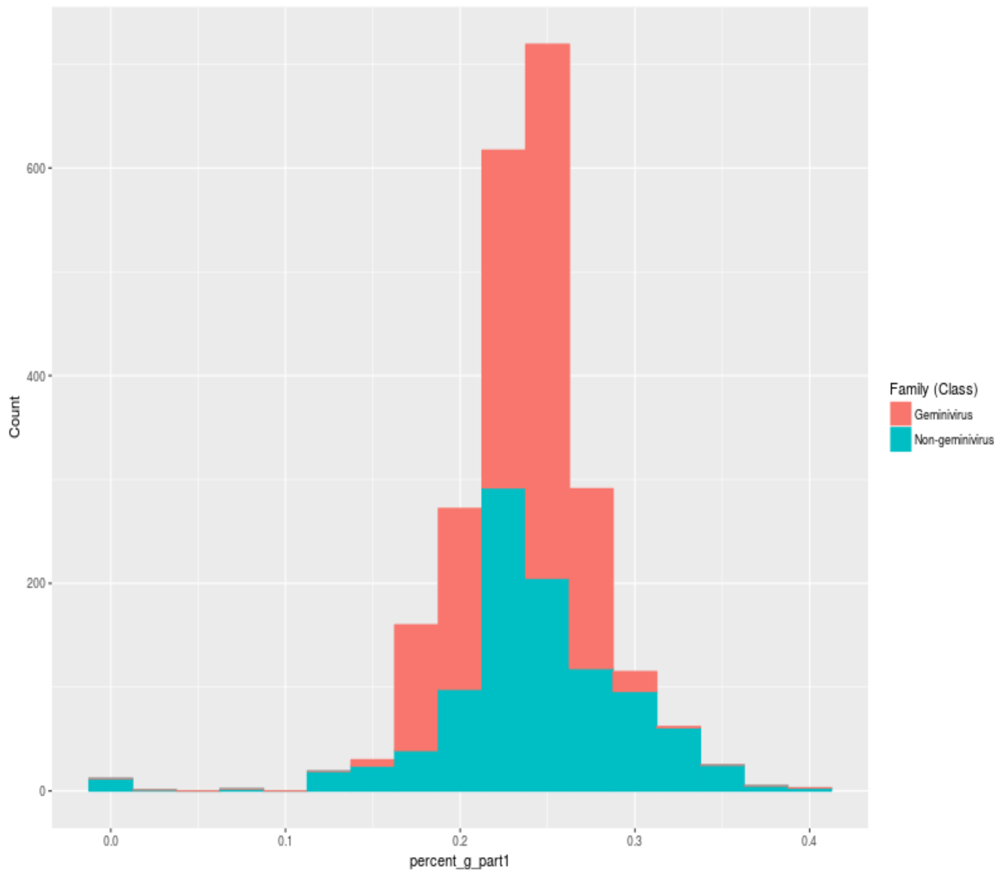

Density

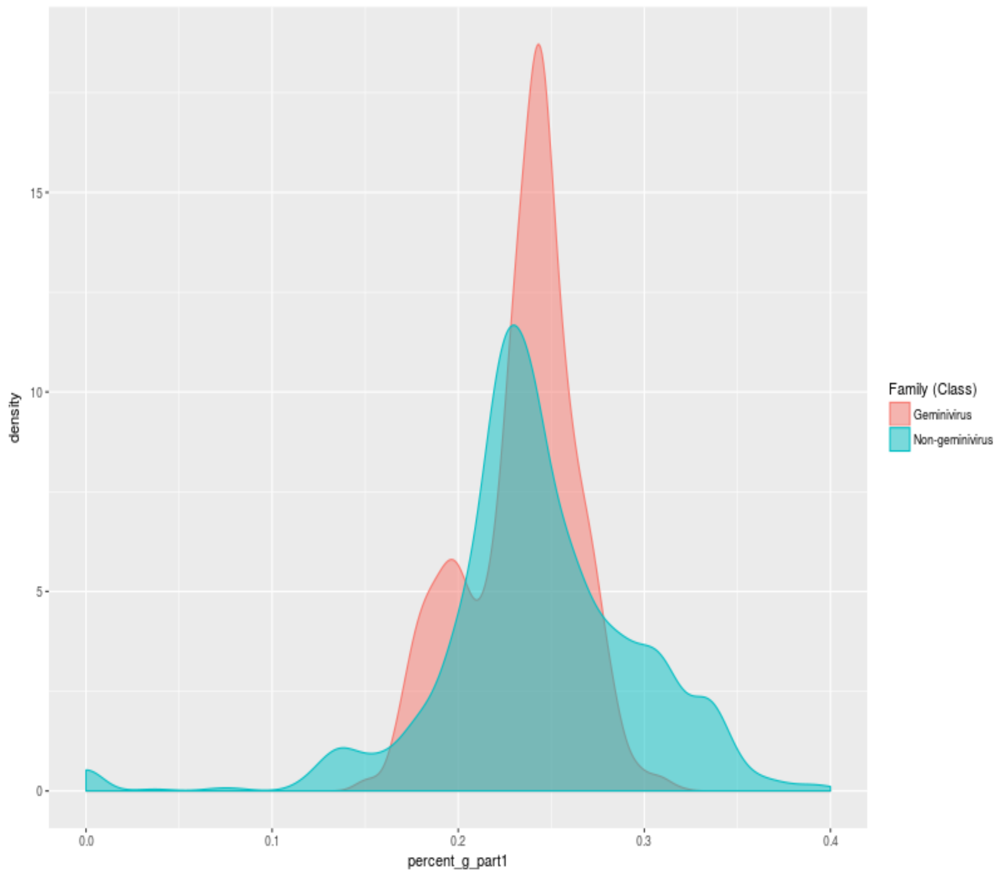

Boxplots

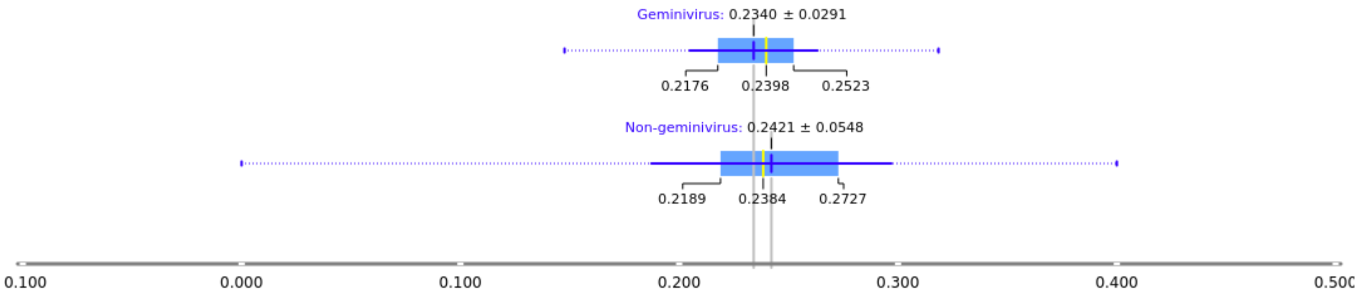

Geminivirus: 0.2340 ± 0.0291
0.2176
0.2398
0.2523
Non-geminivirus: 0.2421 ± 0.0548
0.2189
0.2384
0.2727
0.100
0.000
0.100
0.200
0.300
0.400
0.500

Proportion of Guanine in region 2

Histogram

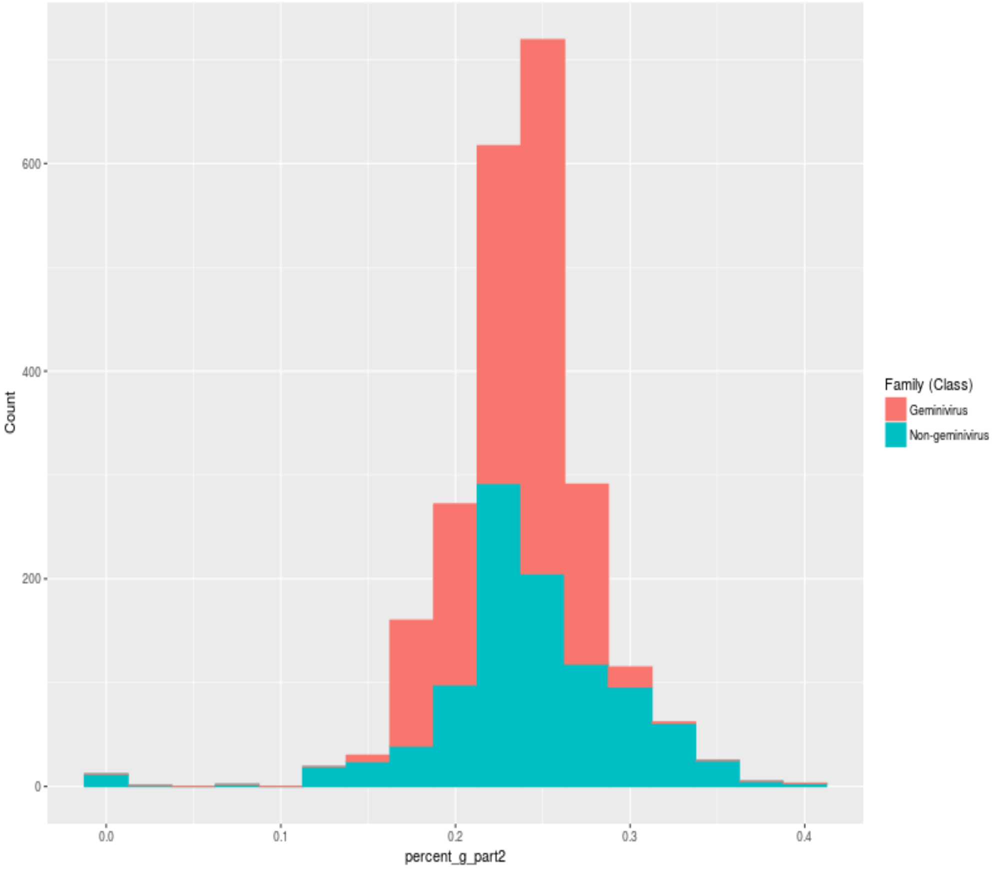

Density

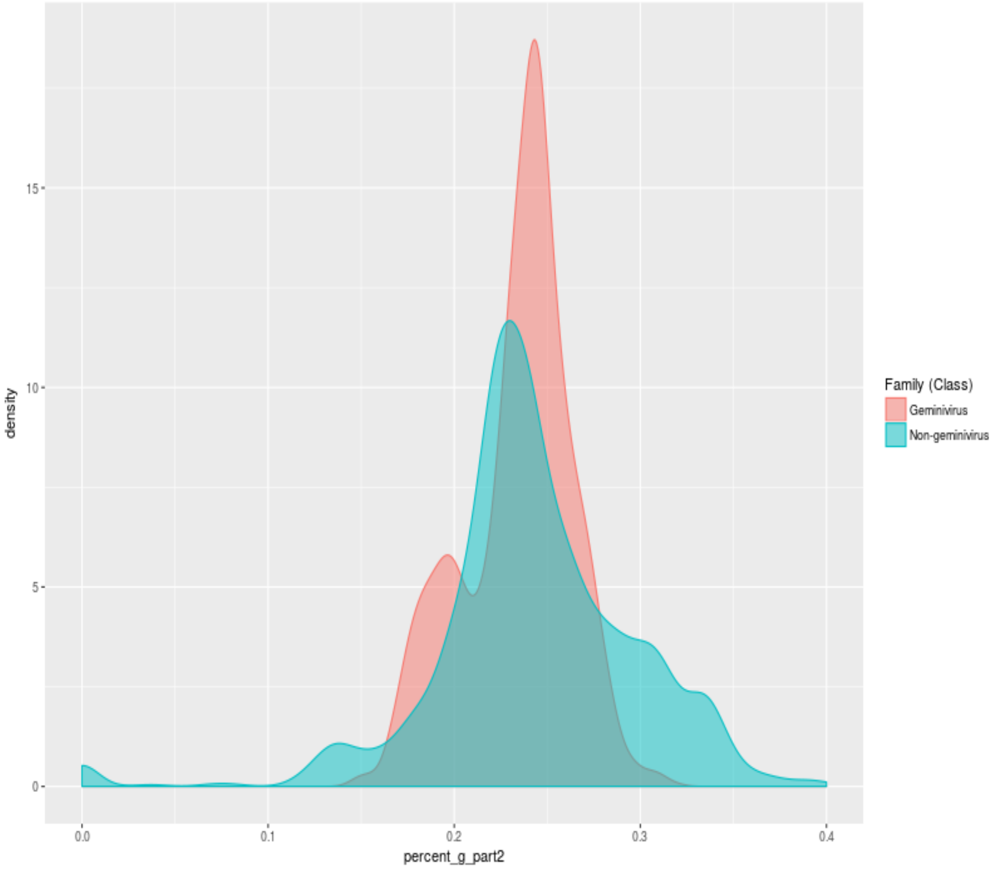

Boxplots

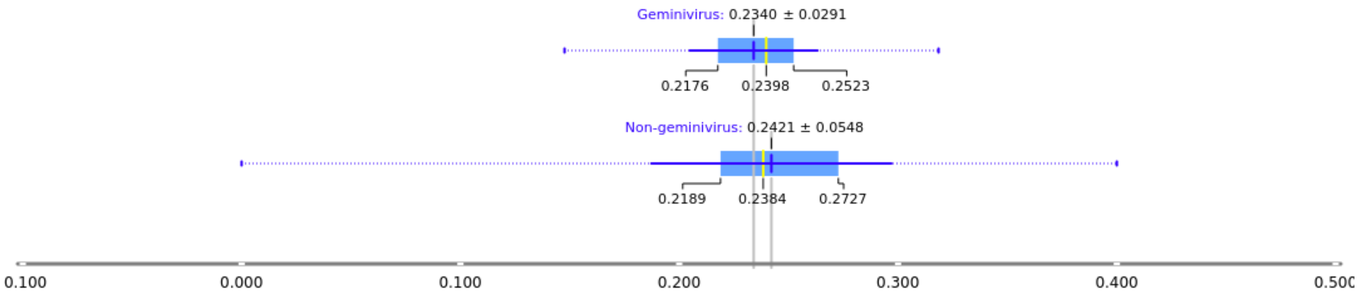

Geminivirus: 0.2340 ± 0.0291
0.2176
0.2398
0.2523
Non-geminivirus: 0.2421 ± 0.0548
0.2189
0.2384
0.2727
0.100
0.000
0.100
0.200
0.300
0.400
0.500

Proportion of Guanine in region 3

Histogram

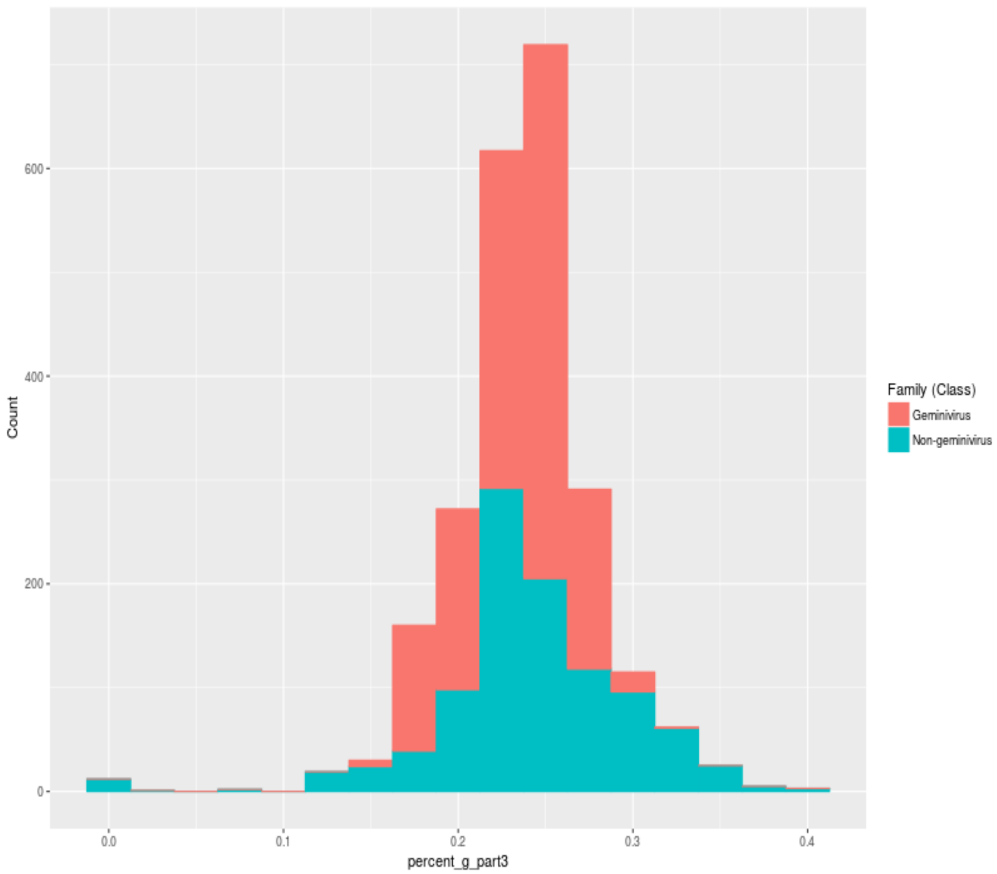

Density

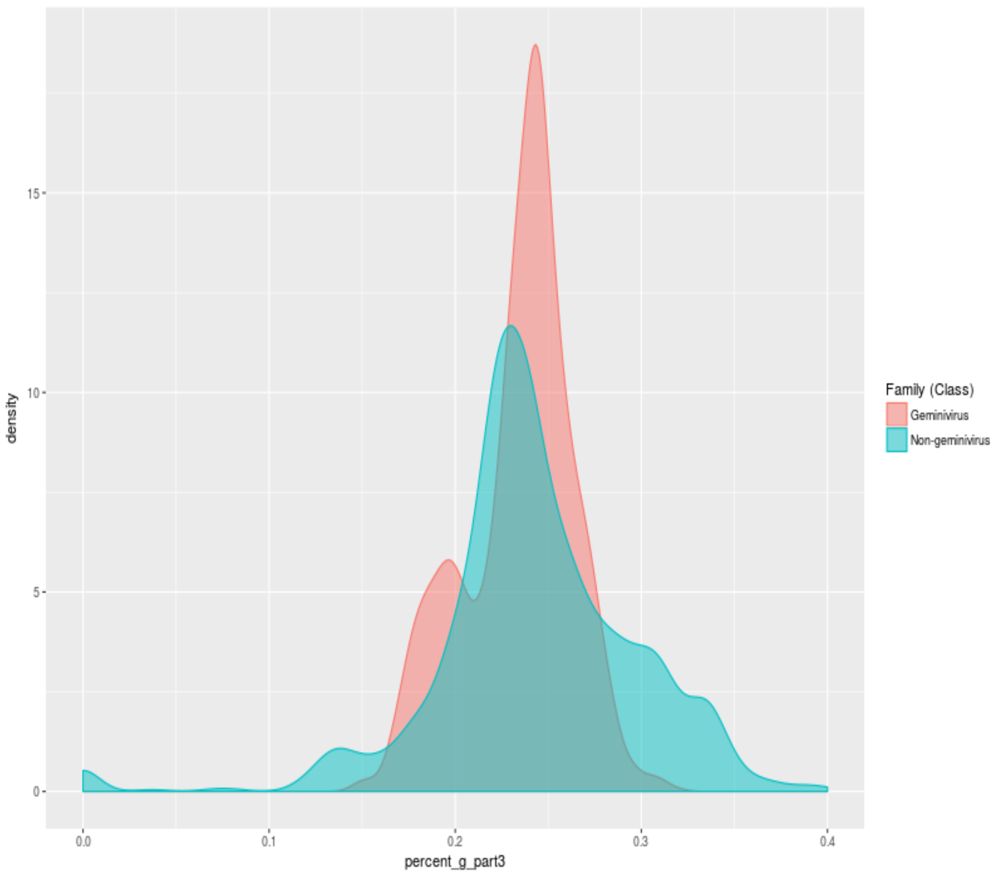

Boxplots

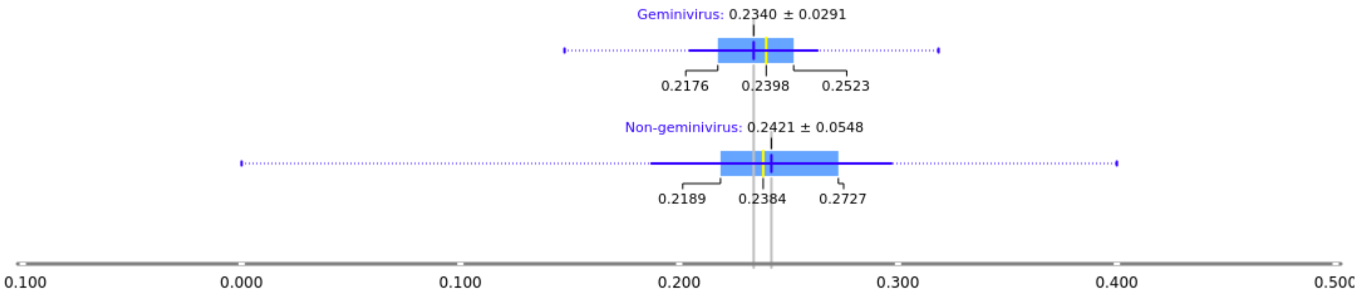
Geminivirus: 0.2340 ± 0.0291
0.2176
0.2398
0.2523
Non-geminivirus: 0.2421 ± 0.0548
0.2189
0.2384
0.2727
0.100
0.000
0.100
0.200
0.300
0.400
0.500

Proportion of Guanine in region 4

Histogram

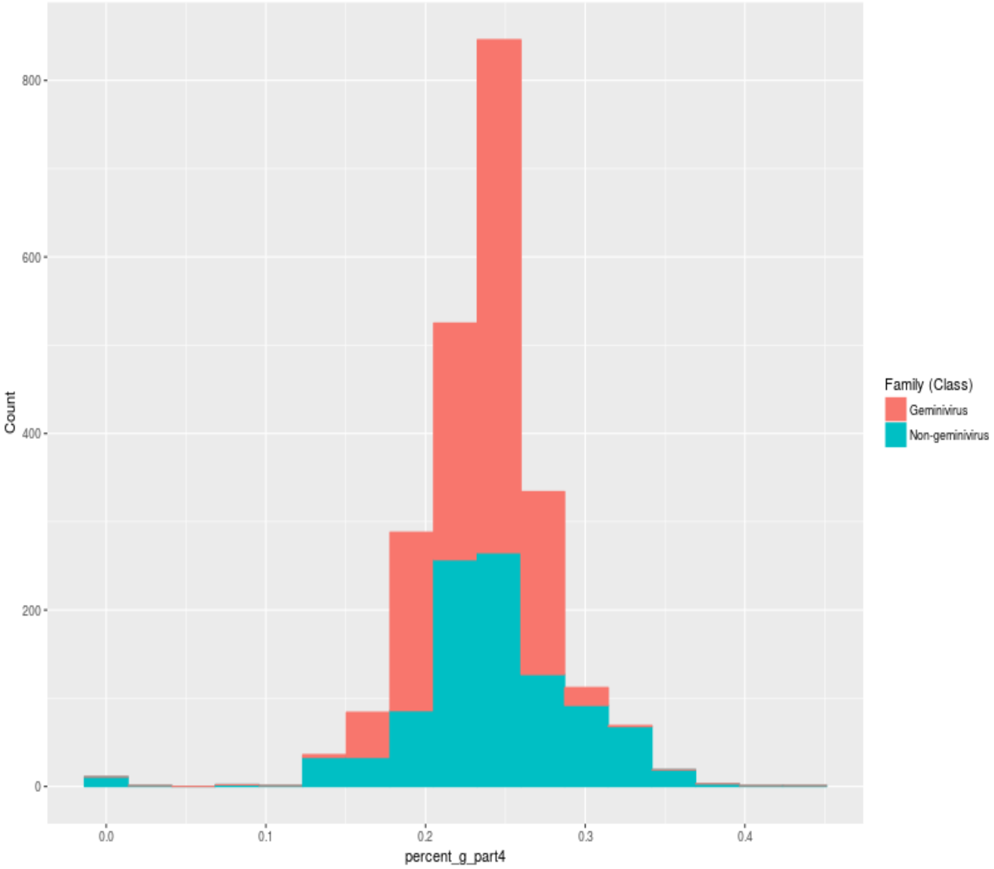

Density

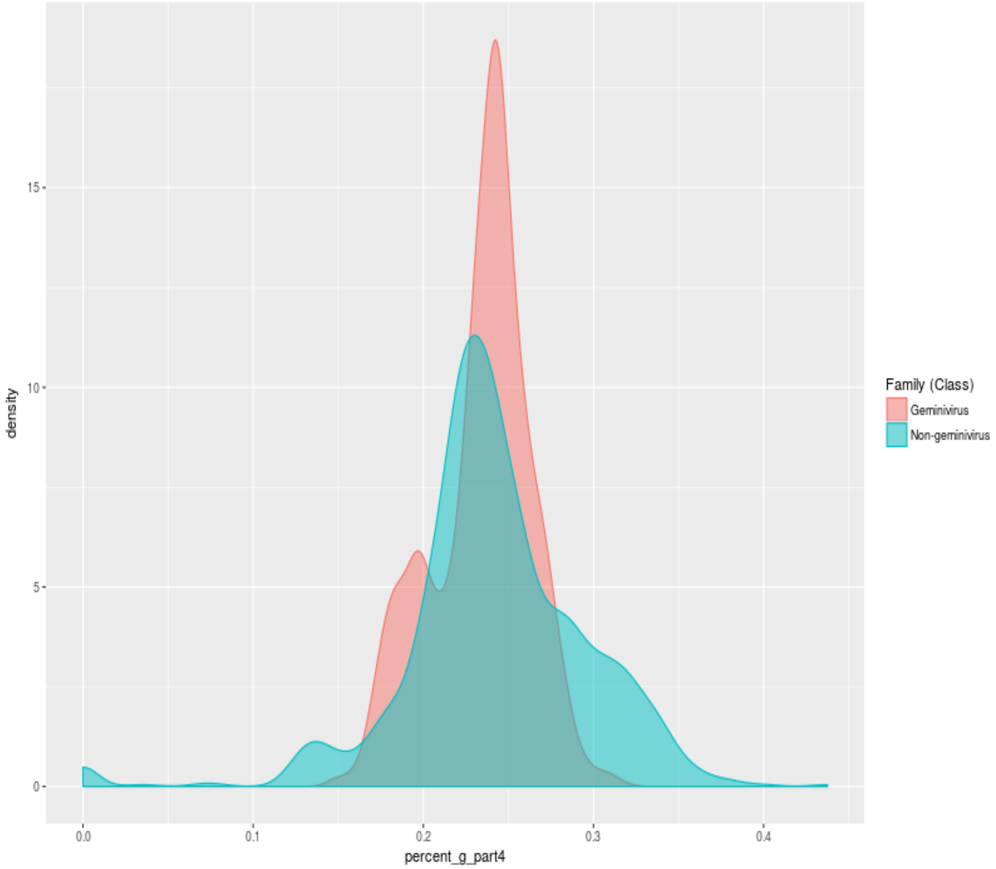

Boxplots

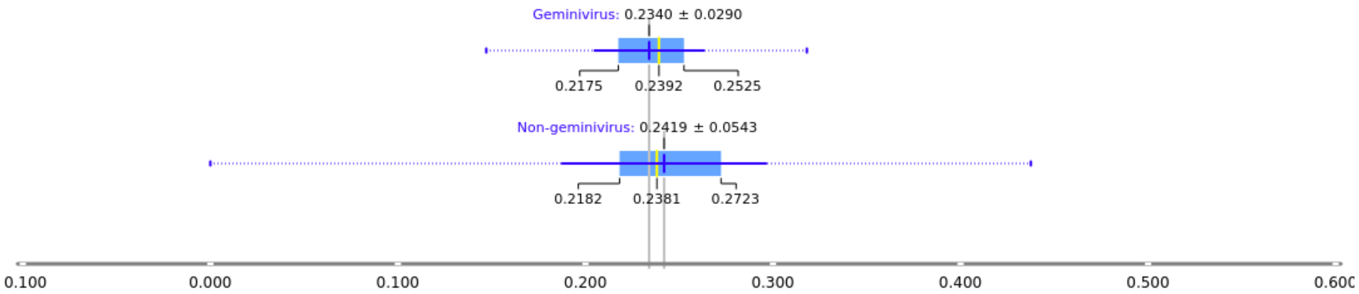

Geminivirus: 0.2340 ± 0.0290
0.2175
0.2392
0.2525
Non-geminivirus: 0.2419 ± 0.0543
0.2182
0.2381
0.2723
0.100
0.000
0.100
0.200
0.300
0.400
0.500
0.600

Proportion of Adenine in the genome

Histogram

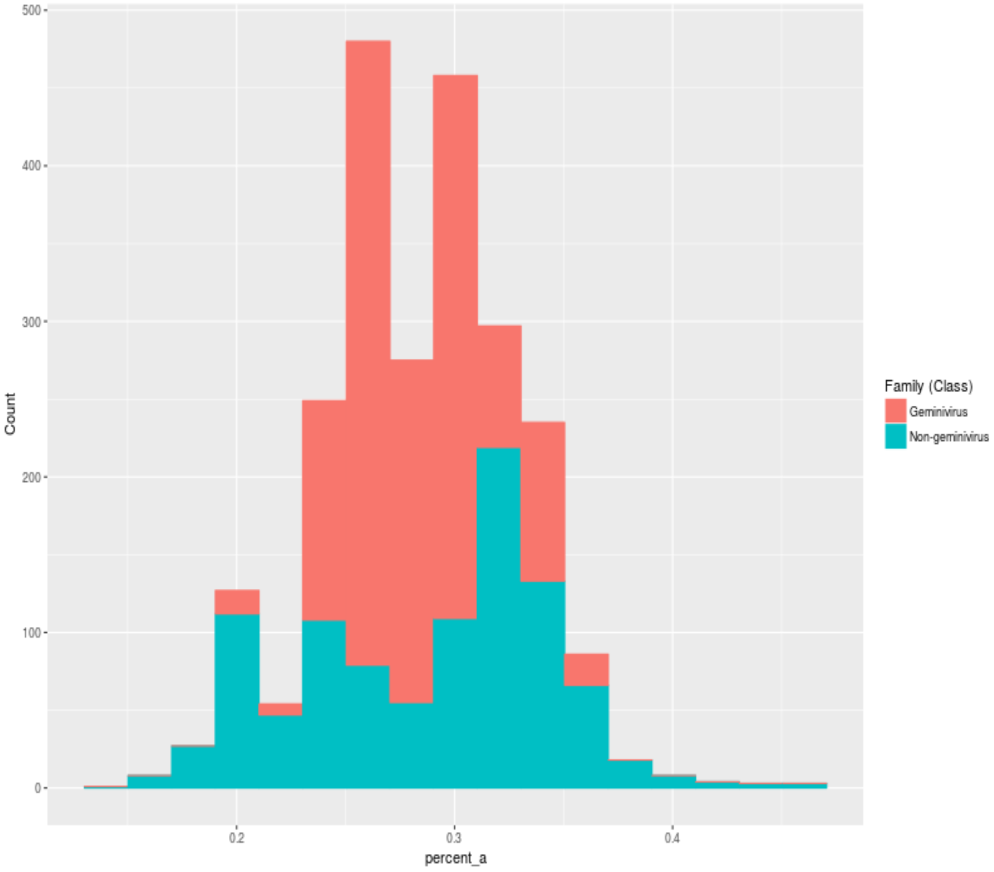

Density

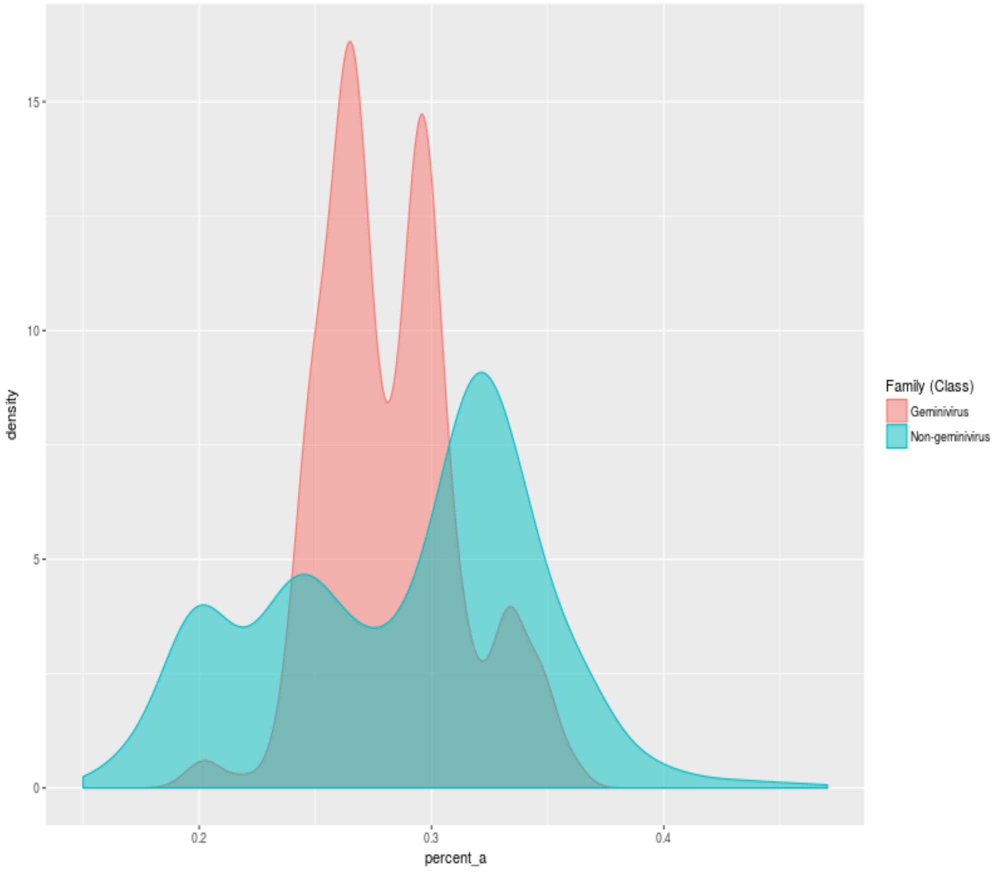

Boxplots

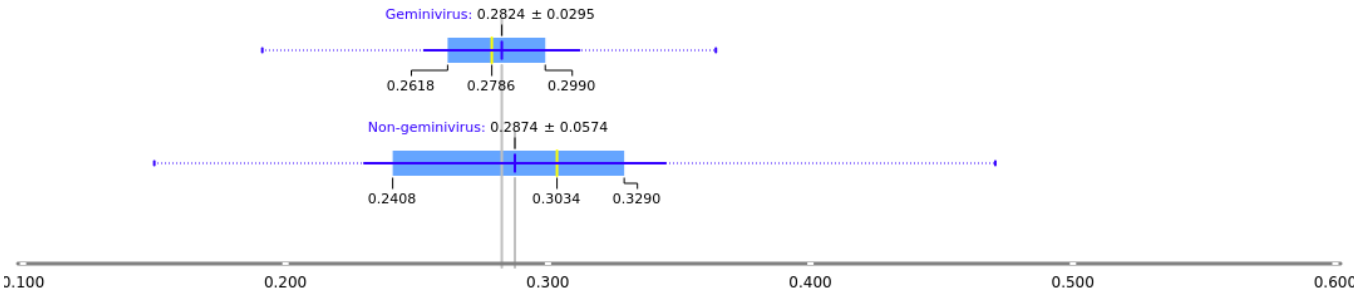

Geminivirus: 0.2824 ± 0.0295
0.2618
0.2786
0.2990
Non-geminivirus: 0.2874 ± 0.0574
0.2408
0.3034
0.3290
0.100
0.200
0.300
0.400
0.500
0.600

Proportion of Adenine in region 1

Histogram

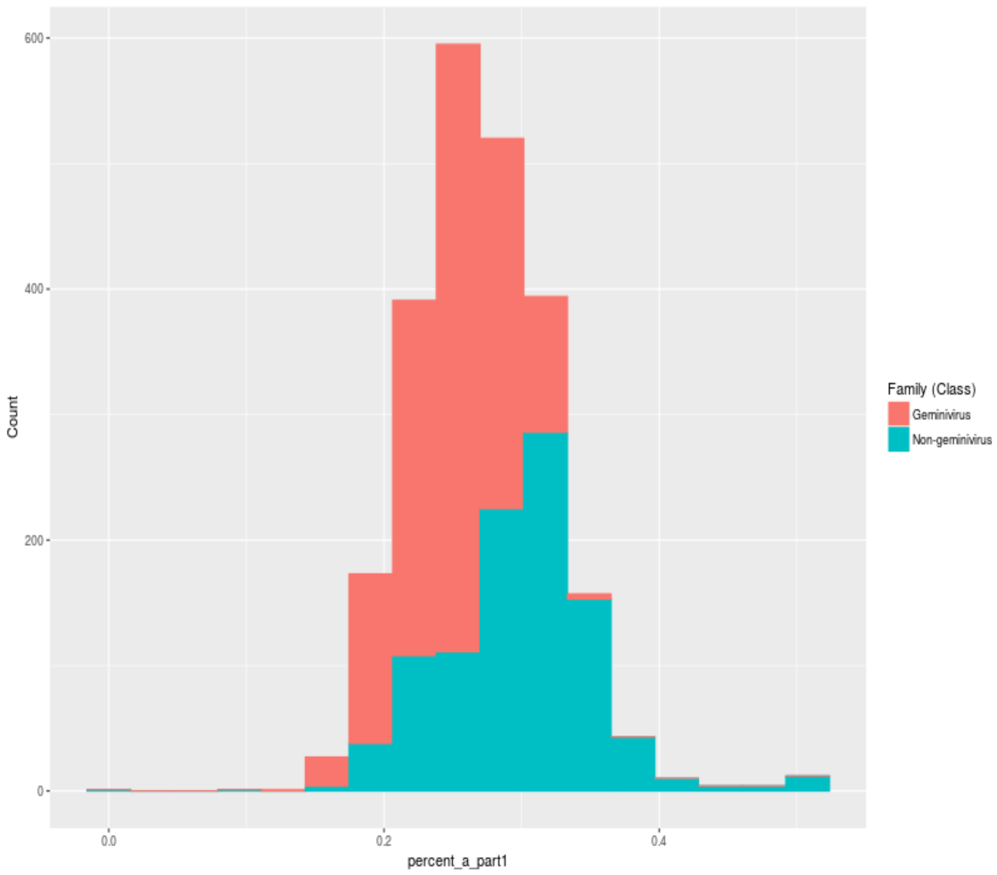

Density

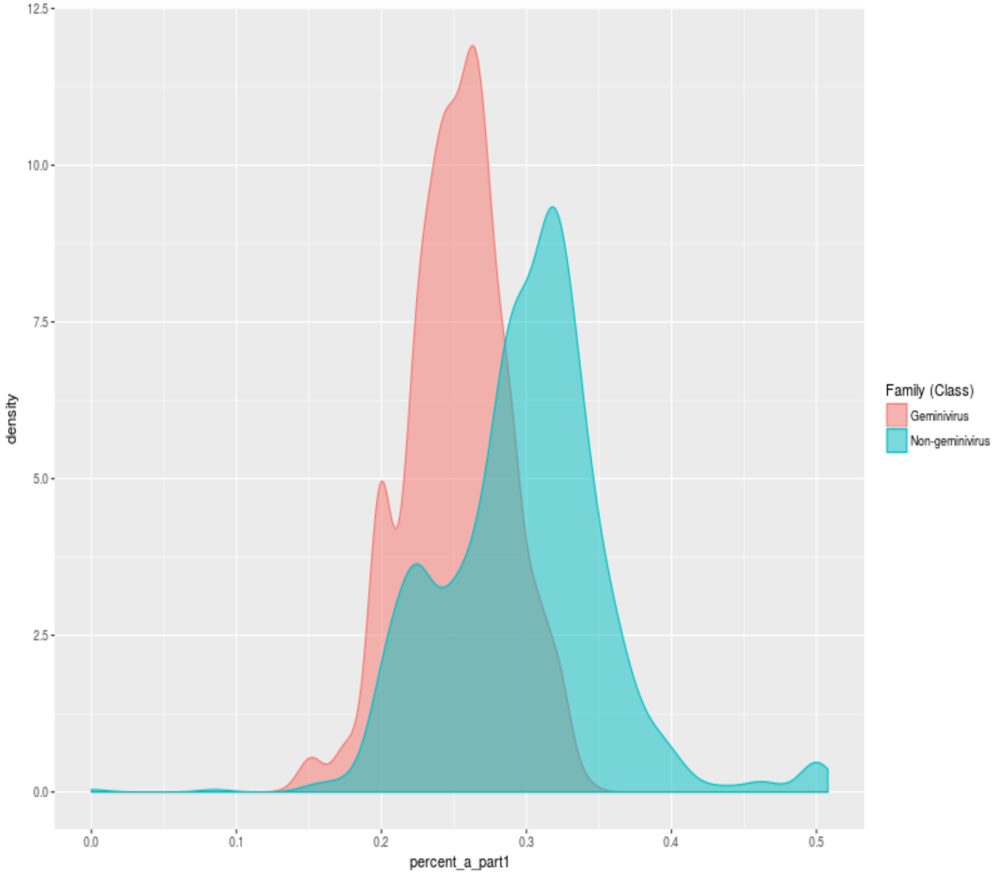

Boxplots

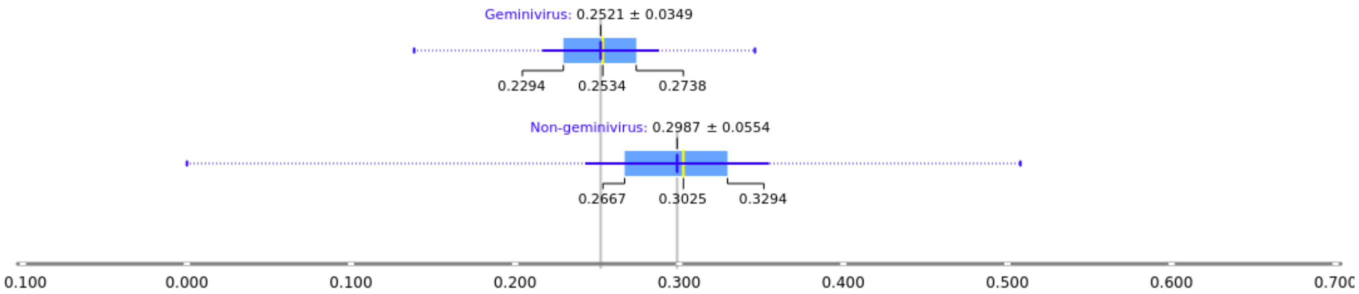

Geminivirus: 0.2521 ± 0.0349
0.2294
0.2534
0.2738
Non-geminivirus: 0.2987 ± 0.0554
0.2667
0.3025
0.3294
0.100
0.000
0.100
0.200
0.300
0.400
0.500
0.600
0.700

Proportion of Adenine in region 2

Histogram

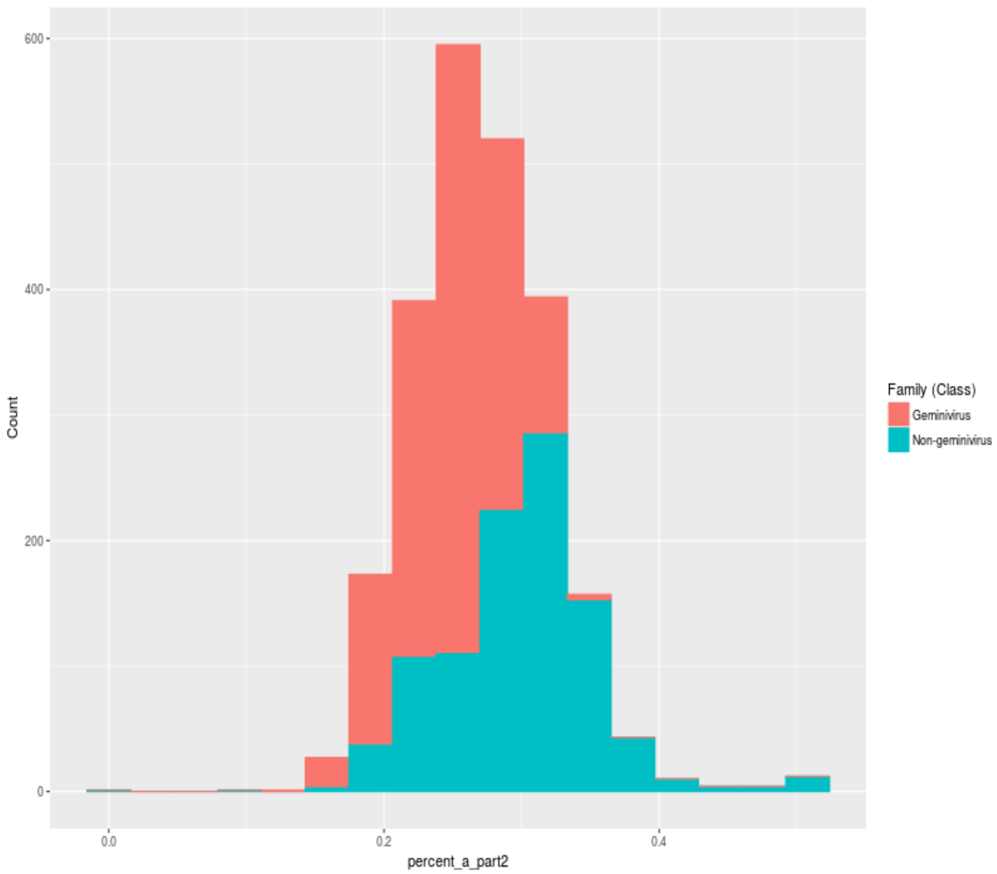

Density

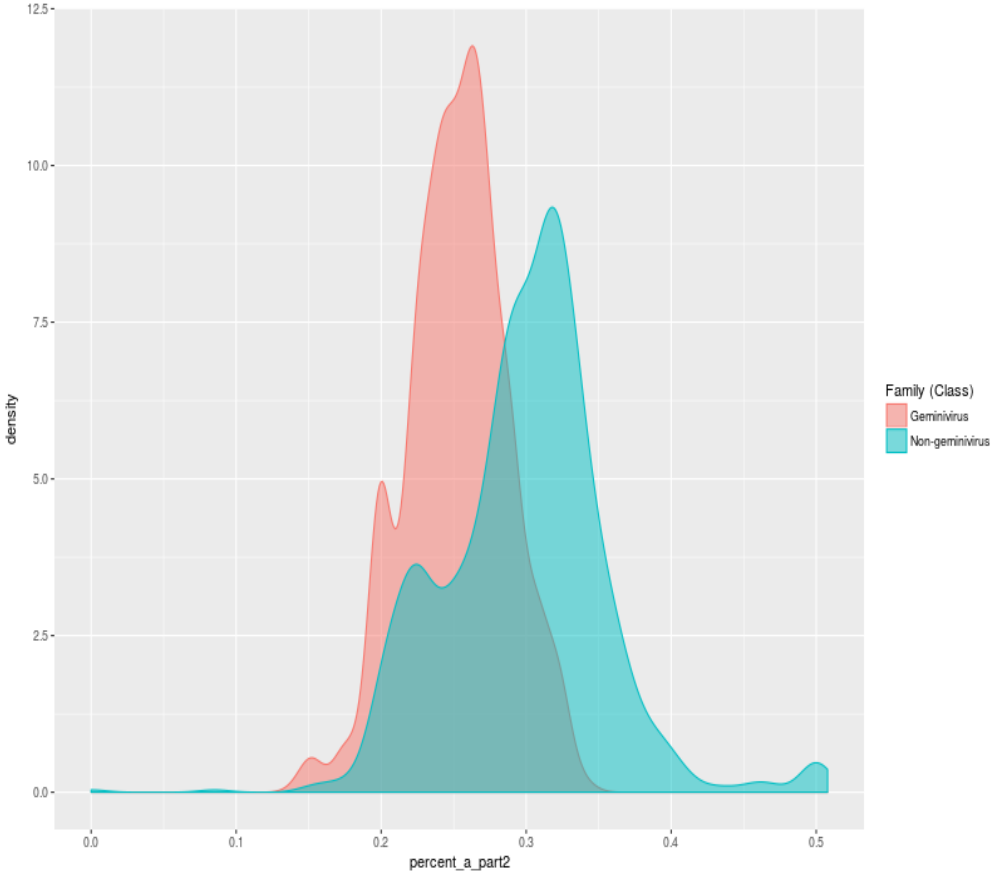

Boxplots

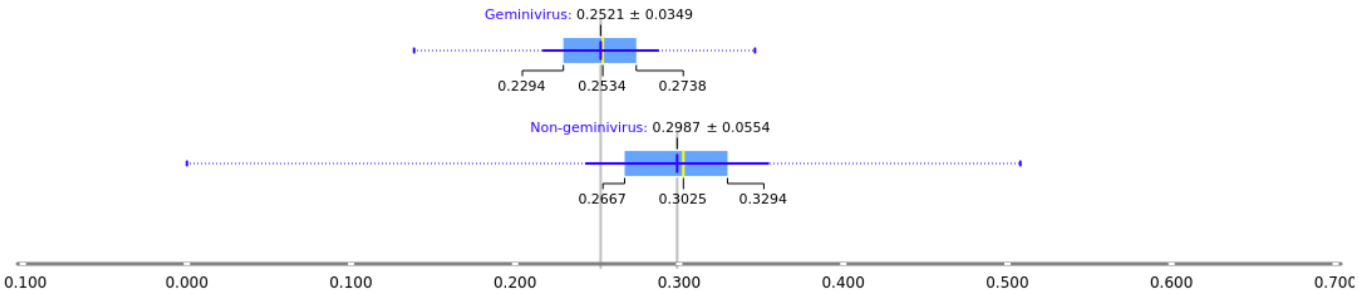

Geminivirus: 0.2521 ± 0.0349
0.2294
0.2534
0.2738
Non-geminivirus: 0.2987 ± 0.0554
0.2667
0.3025
0.3294
0.100
0.000
0.100
0.200
0.300
0.400
0.500
0.600
0.700

Proportion of Adenine in region 3

Histogram

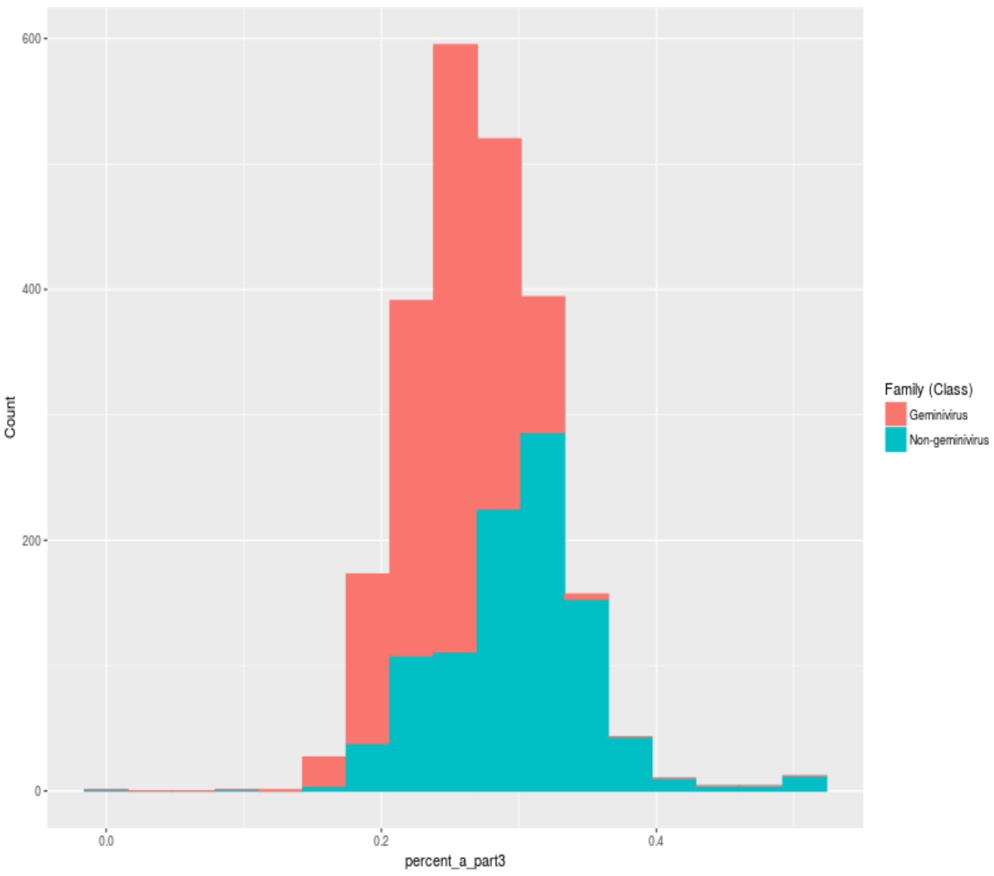

Density

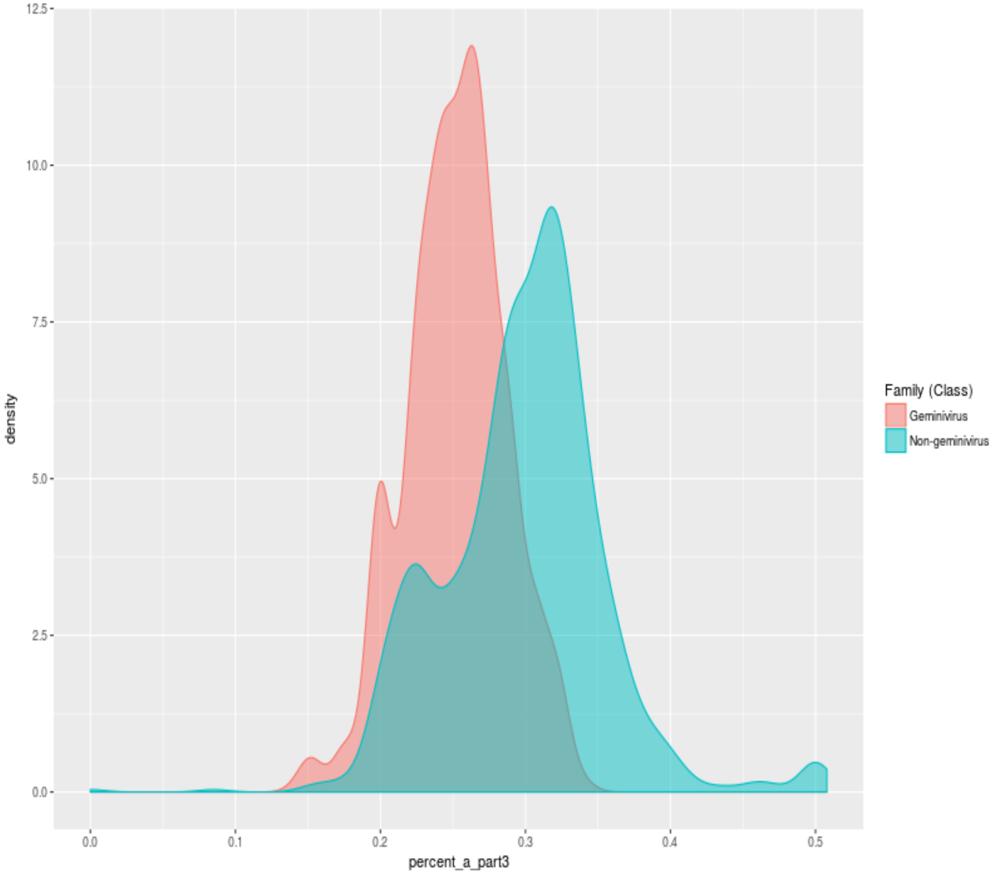

Boxplots

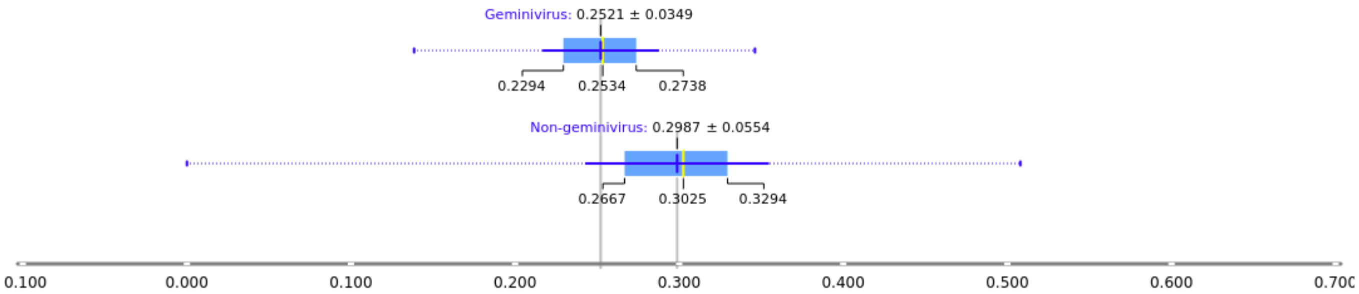

Geminivirus: 0.2521 ± 0.0349
0.2294
0.2534
0.2738
Non-geminivirus: 0.2987 ± 0.0554
0.2667
0.3025
0.3294
0.100
0.000
0.100
0.200
0.300
0.400
0.500
0.600
0.700

Proportion of Adenine in region 4

Histogram

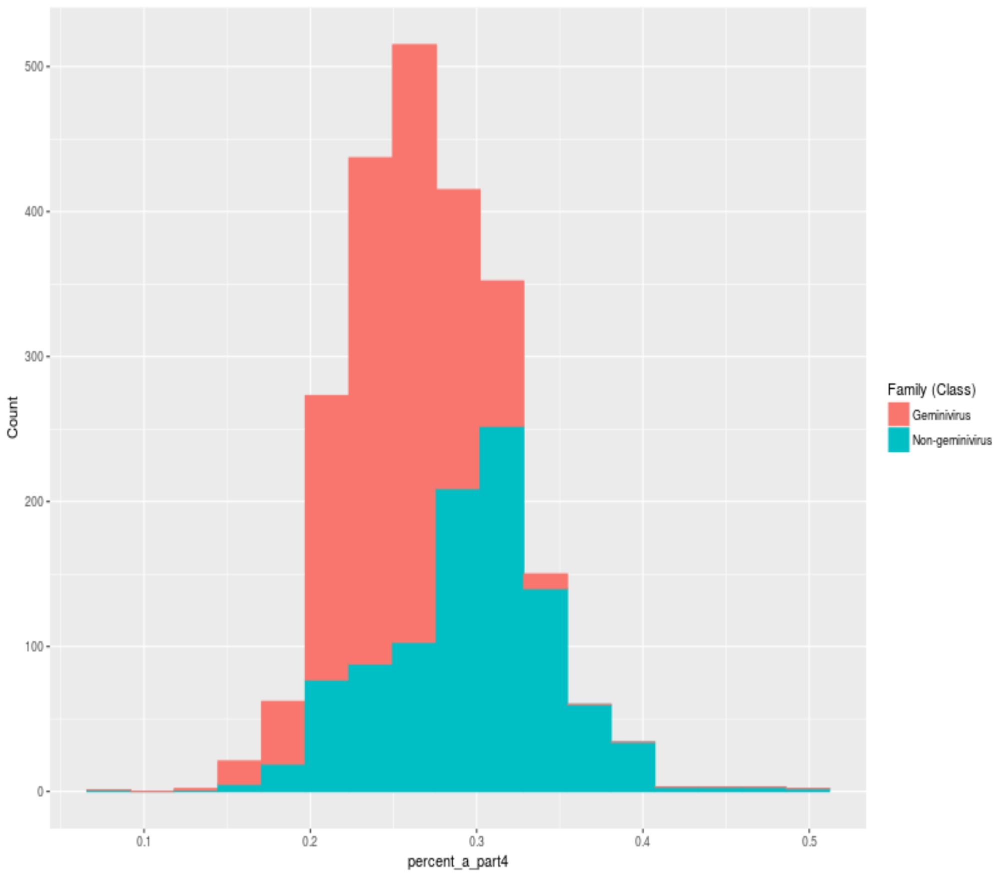

Density

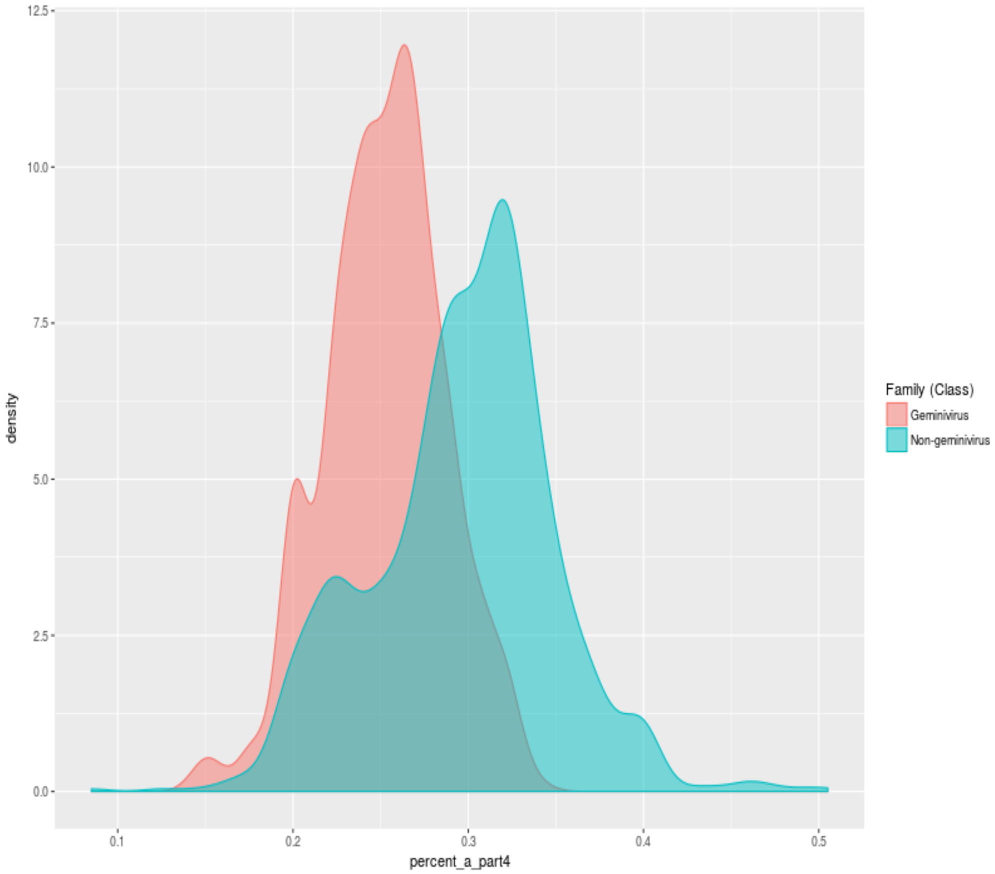

Boxplots

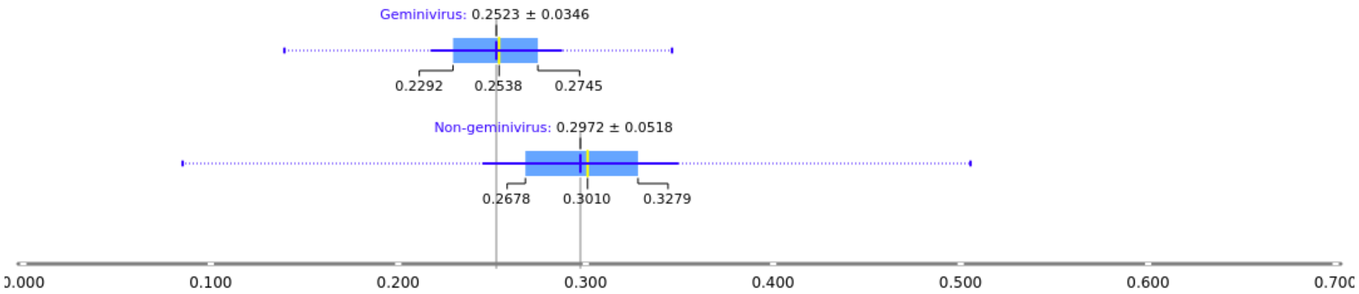

Geminivirus: 0.2523 ± 0.0346
0.2292
0.2538
0.2745
Non-geminivirus: 0.2972 ± 0.0518
0.2678
0.3010
0.3279
0.000
0.100
0.200
0.300
0.400
0.500
0.600
0.700

Proportion of Thymine in the genome

Histogram

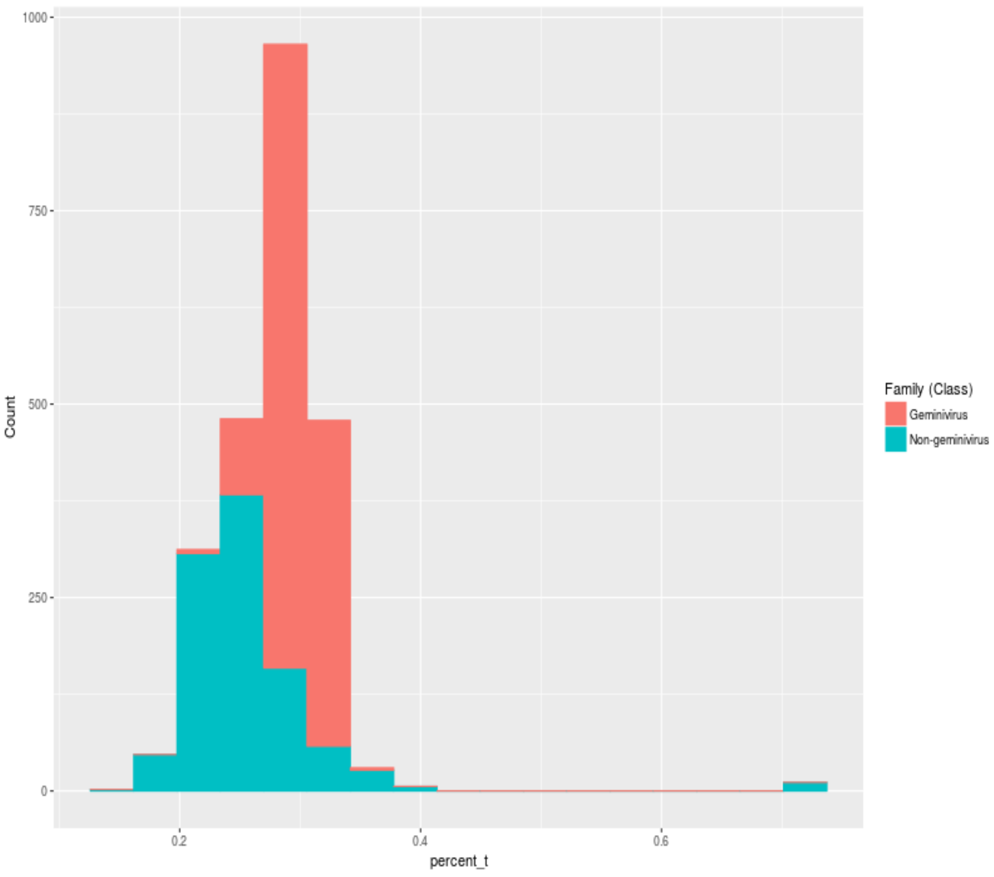

Density

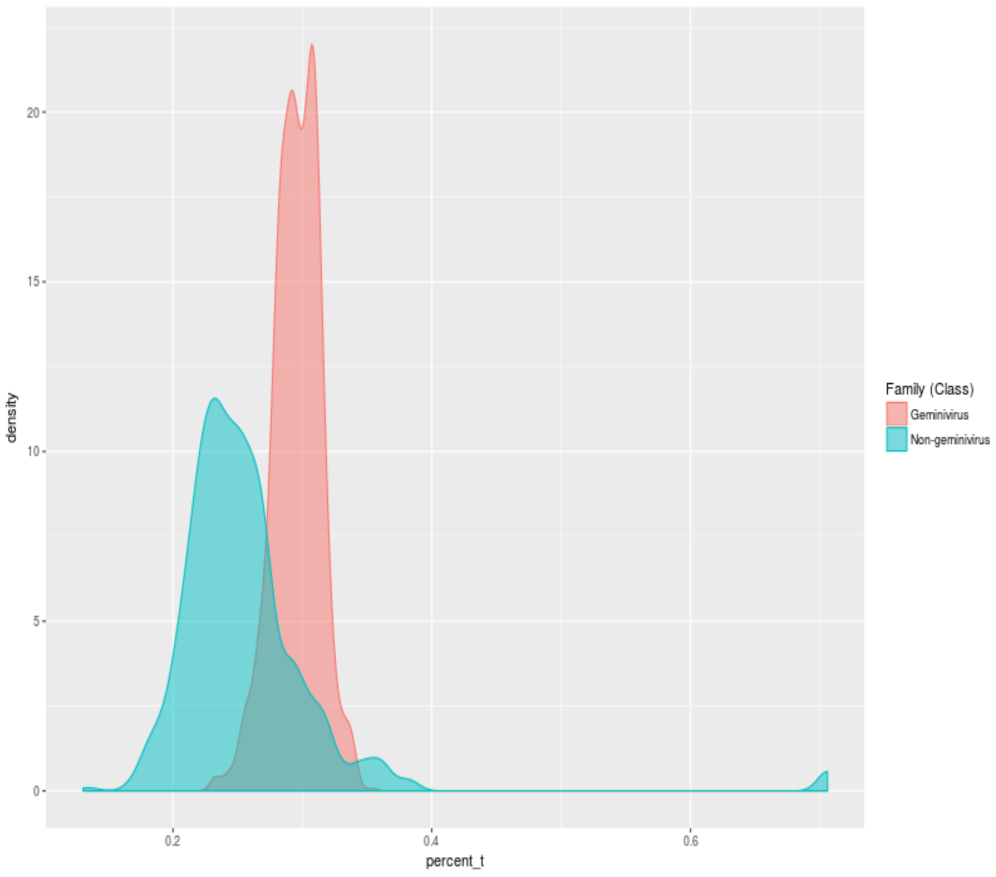

Boxplots

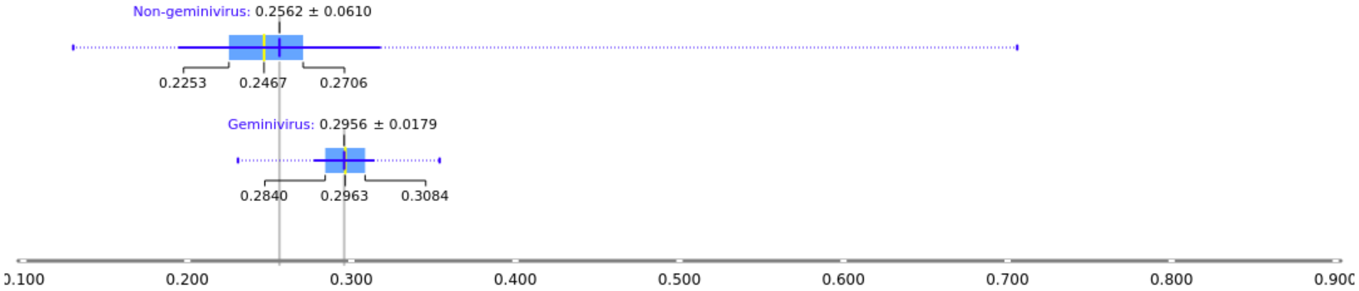

Non-geminivirus: 0.2562 ± 0.0610
0.2253
0.2467
0.2706
Geminivirus: 0.2956 ± 0.0179
0.2840
0.2963
0.3084
0.100
0.200
0.300
0.400
0.500
0.600
0.700
0.800
0.900

Proportion of Thymine in region 1

Histogram

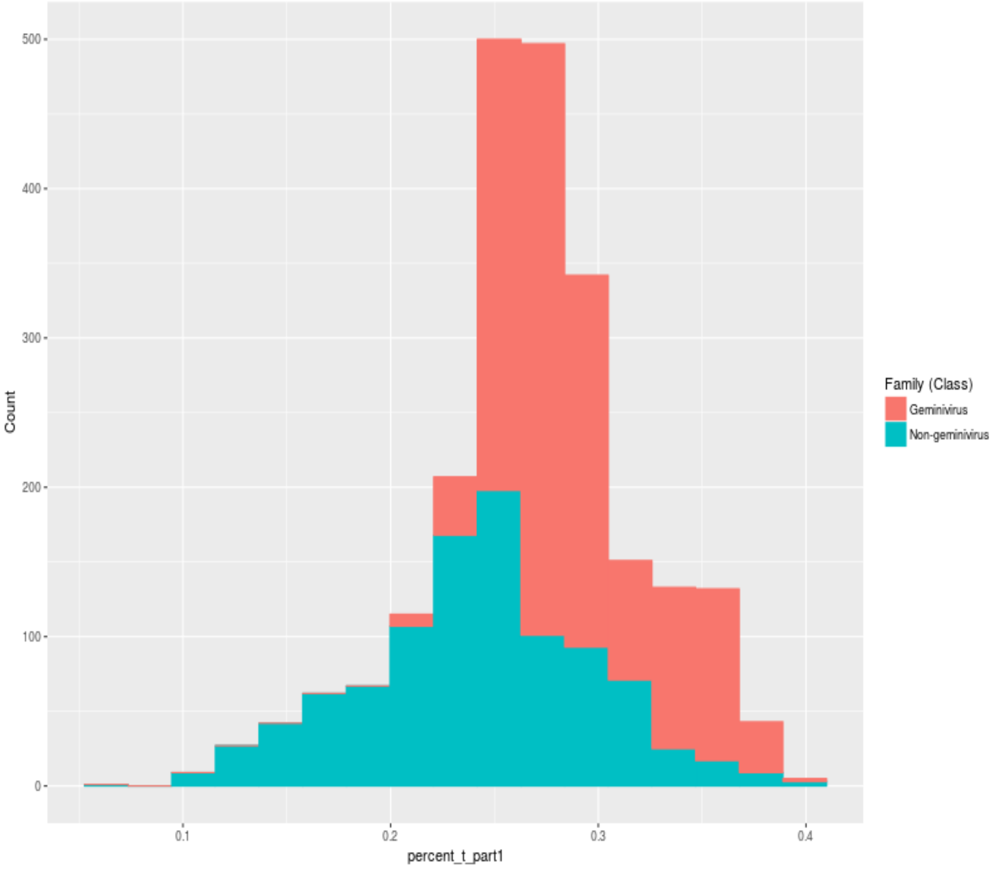

Density

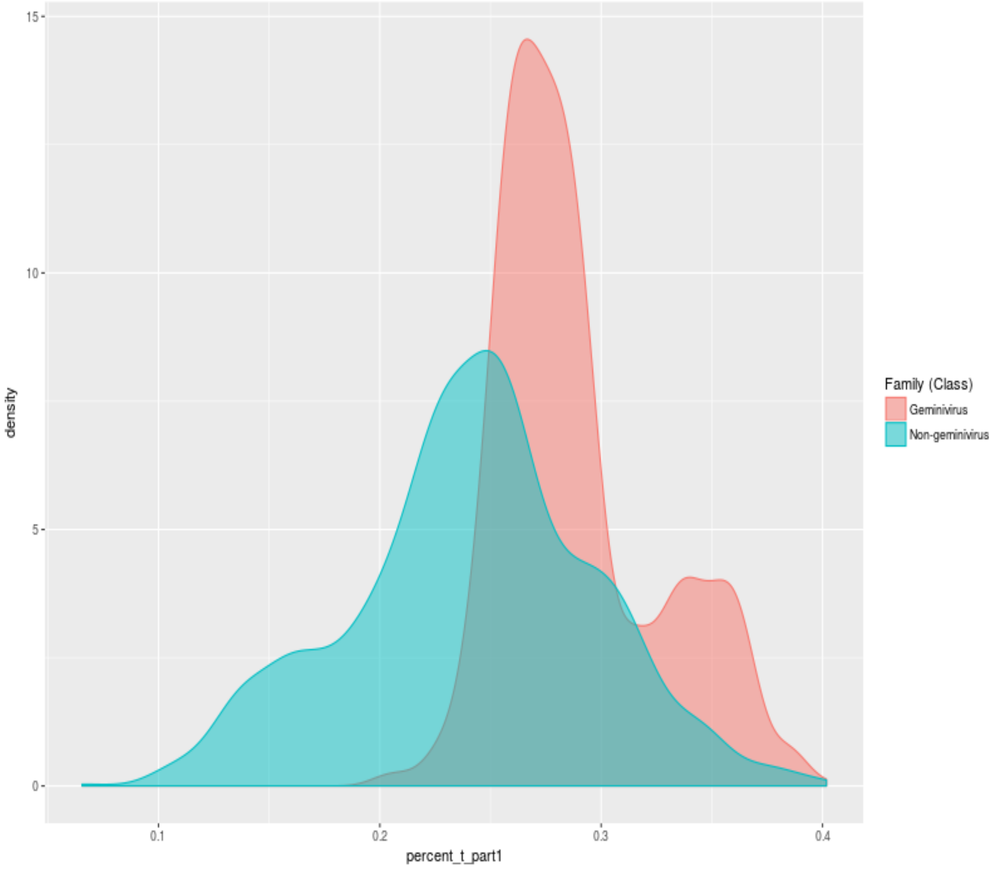

Boxplots

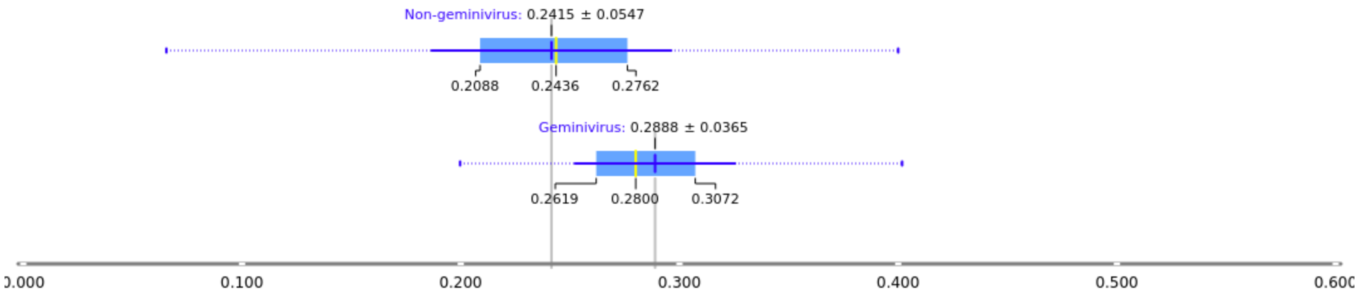

Non-geminivirus: 0.2415 ± 0.0547
0.2088
0.2436
0.2762
Geminivirus: 0.2888 ± 0.0365
0.2619
0.2800
0.3072
0.000
0.100
0.200
0.300
0.400
0.500
0.600

Proportion of Thymine in region 2

Histogram

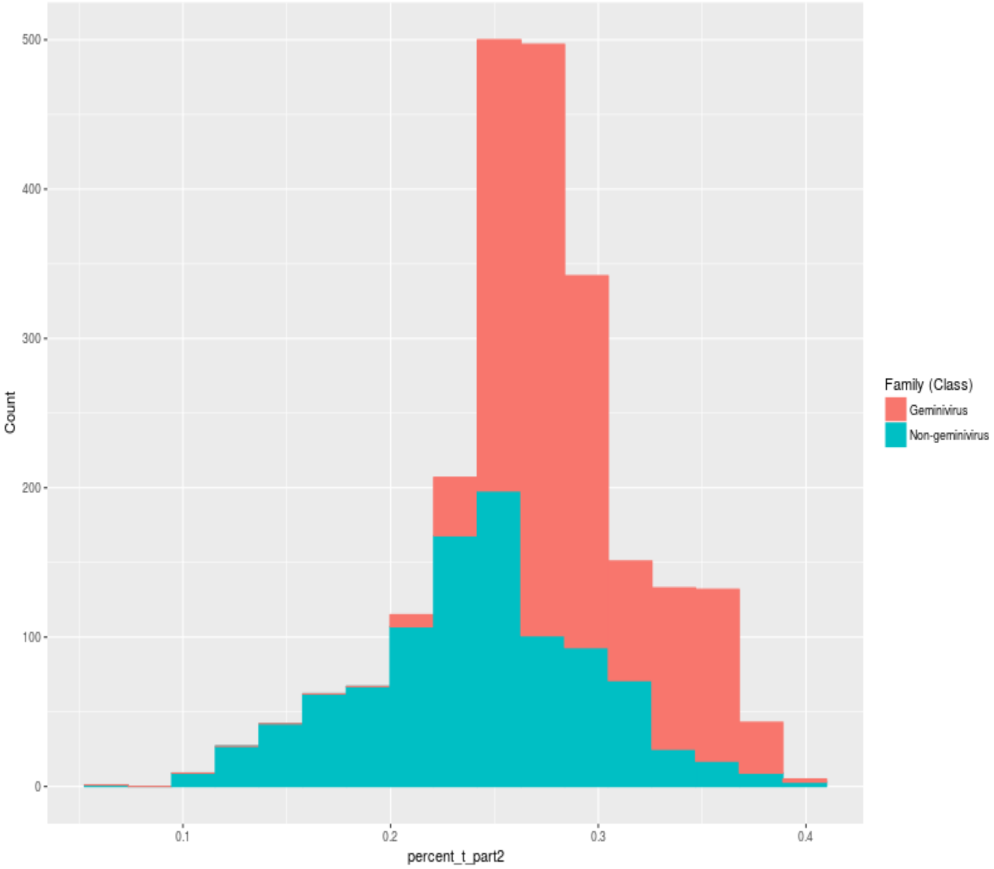

Density

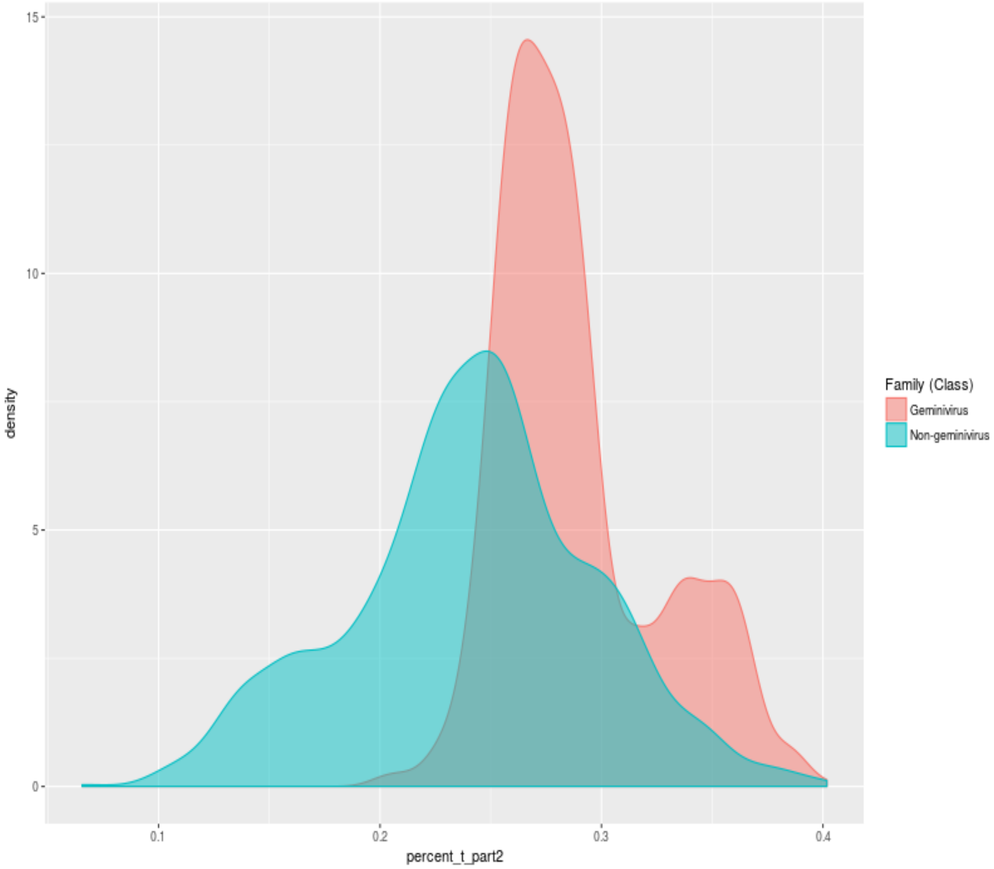

Boxplots

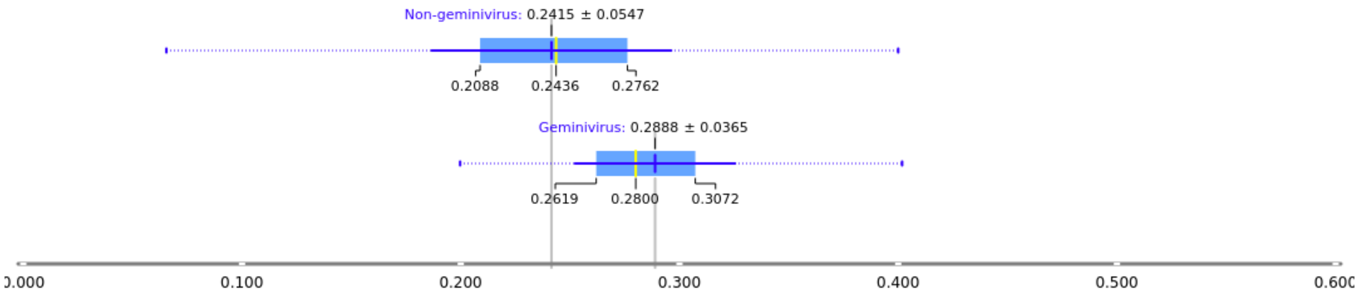

Non-geminivirus: 0.2415 ± 0.0547
0.2088
0.2436
0.2762
Geminivirus: 0.2888 ± 0.0365
0.2619
0.2800
0.3072
0.000
0.100
0.200
0.300
0.400
0.500
0.600

Proportion of Thymine in region 3

Histogram

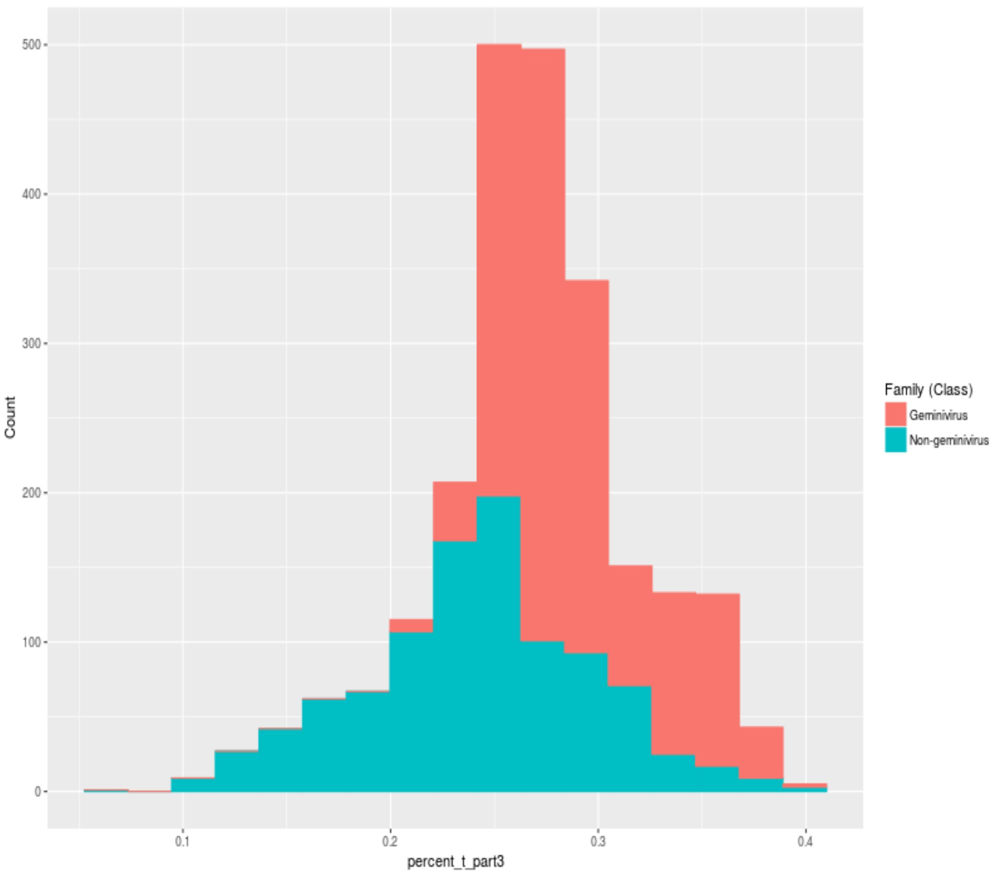

Density

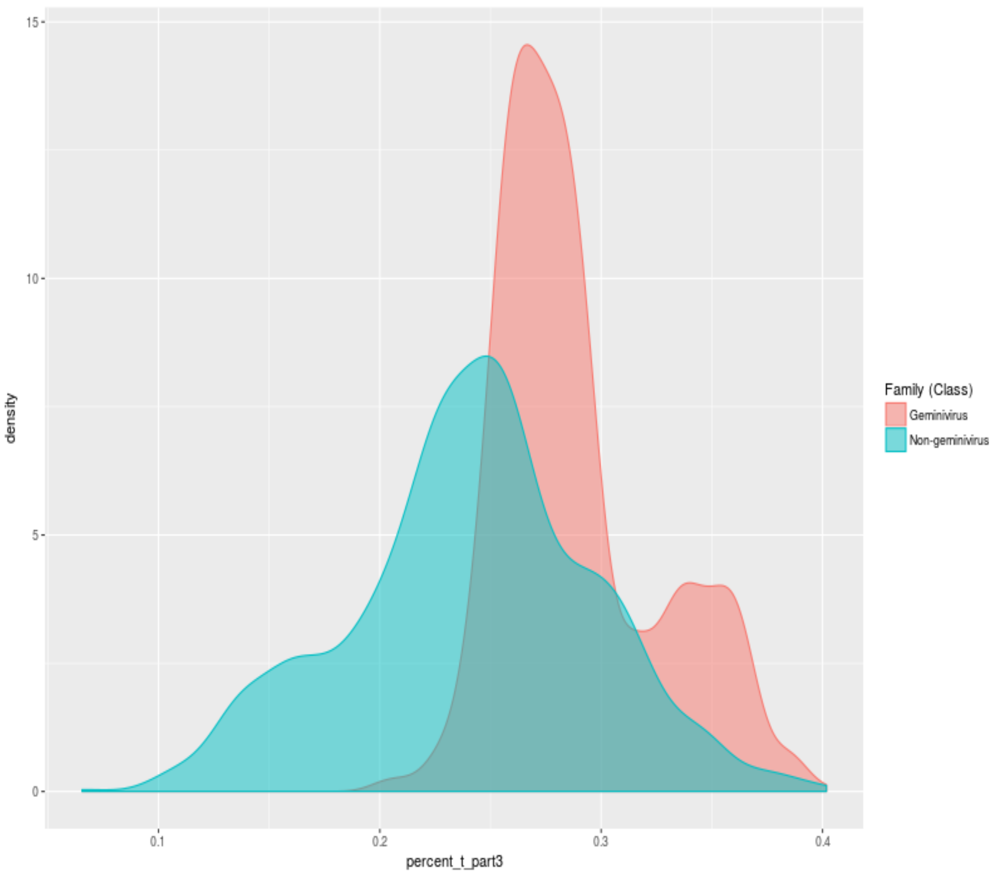

Boxplots

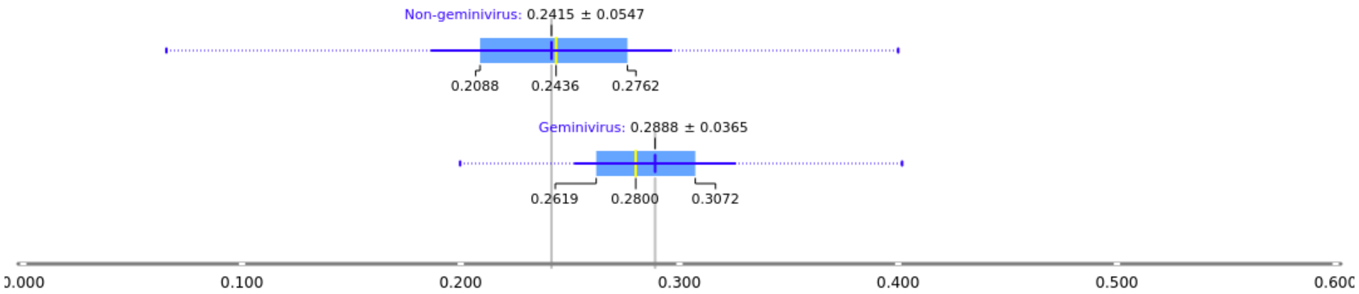

Non-geminivirus: 0.2415 ± 0.0547
0.2088
0.2436
0.2762
Geminivirus: 0.2888 ± 0.0365
0.2619
0.2800
0.3072
0.000
0.100
0.200
0.300
0.400
0.500
0.600

Proportion of Thymine in region 4

Histogram

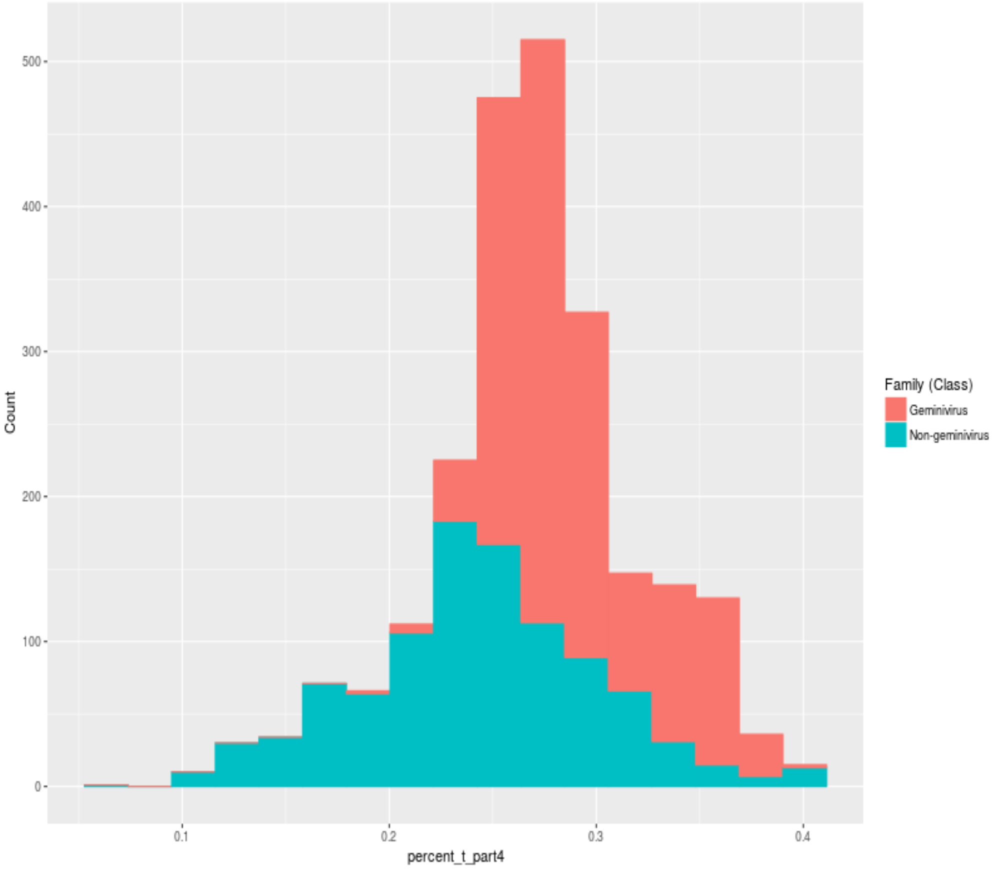

Density

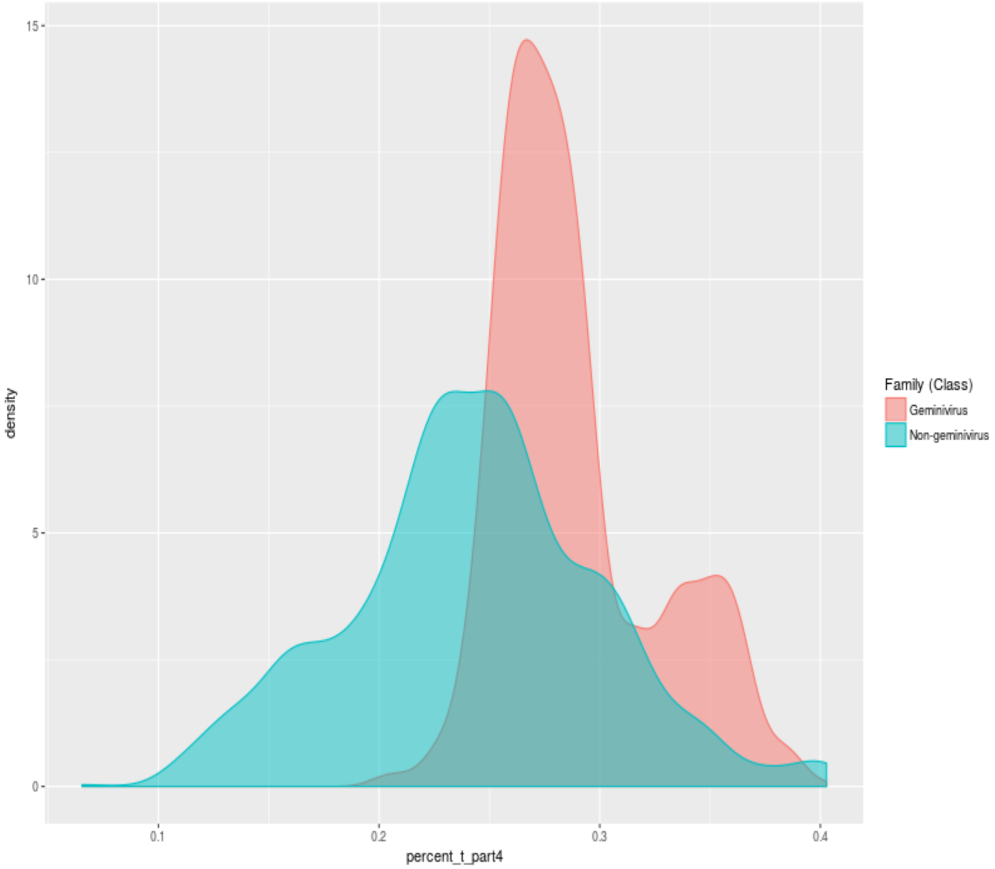

Boxplots

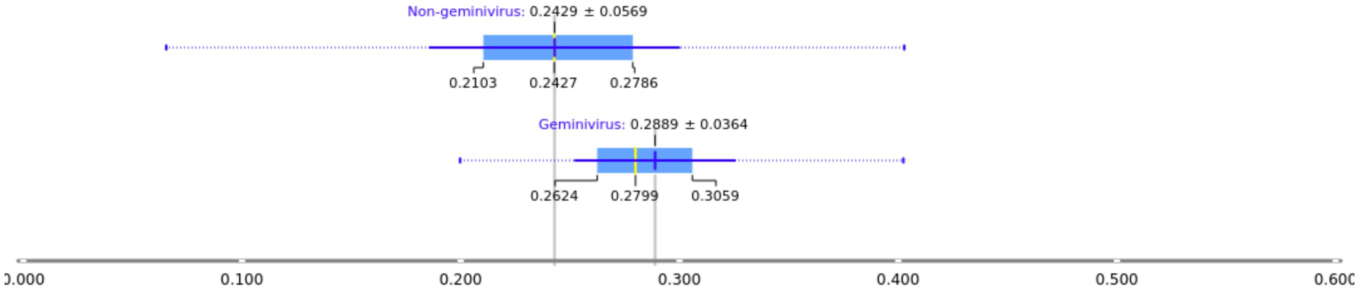
Non-geminivirus: 0.2429 ± 0.0569
0.2103
0.2427
0.2786
Geminivirus: 0.2889 ± 0.0364
0.2624
0.2799
0.3059
0.000
0.100
0.200
0.300
0.400
0.500
0.600

Proportion of Cytosine in the genome

Histogram

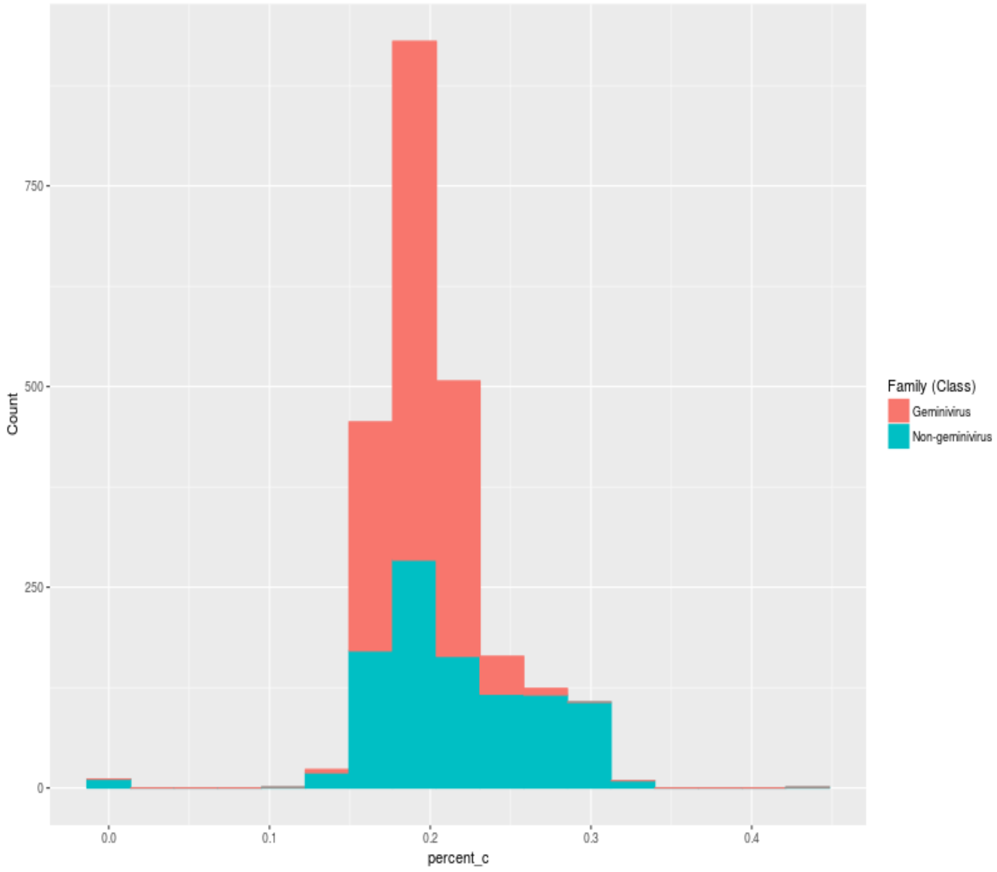

Density

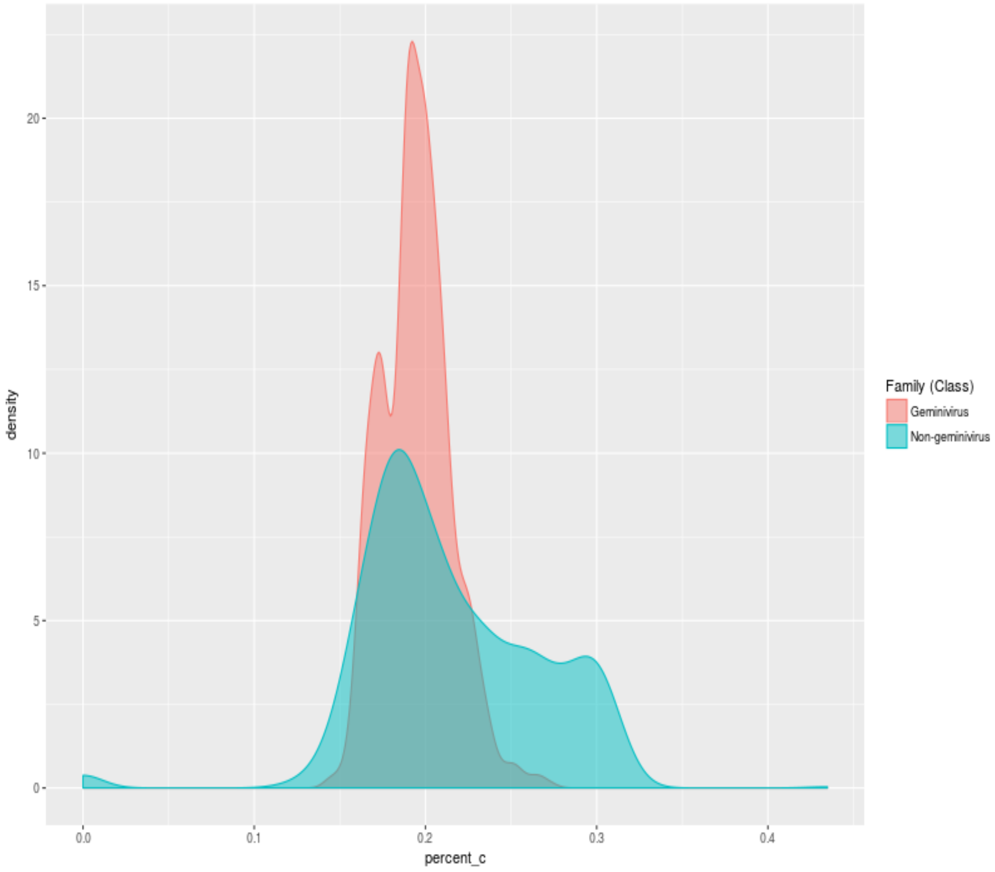

Boxplots

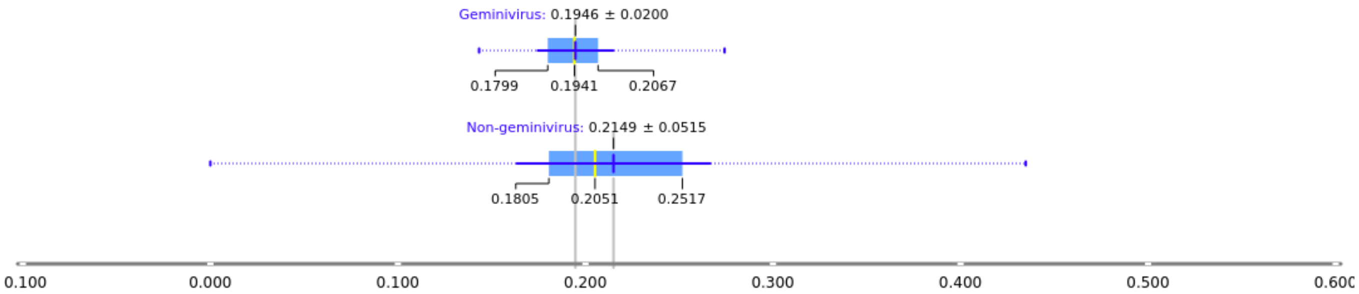

Geminivirus: 0.1946 ± 0.0200
0.1799
0.1941
0.2067
Non-geminivirus: 0.2149 ± 0.0515
0.1805
0.2051
0.2517
0.100
0.000
0.100
0.200
0.300
0.400
0.500
0.600

Proportion of Cytosine in region 1

Histogram

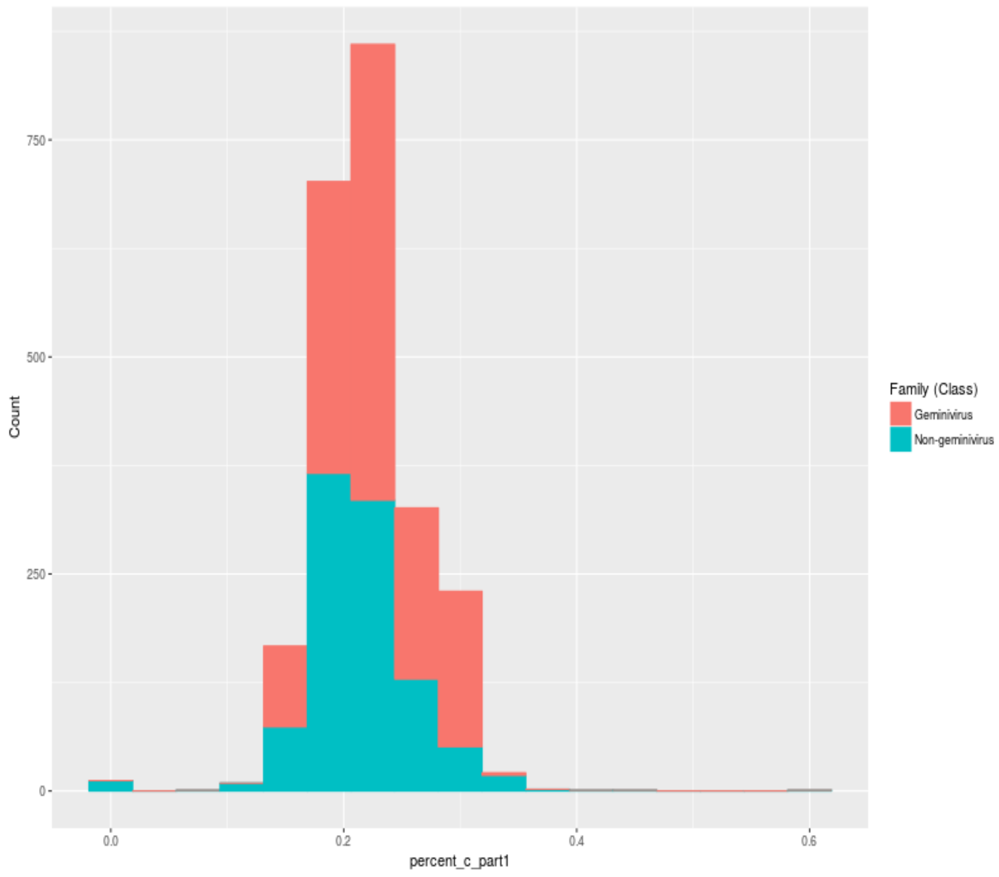

Density

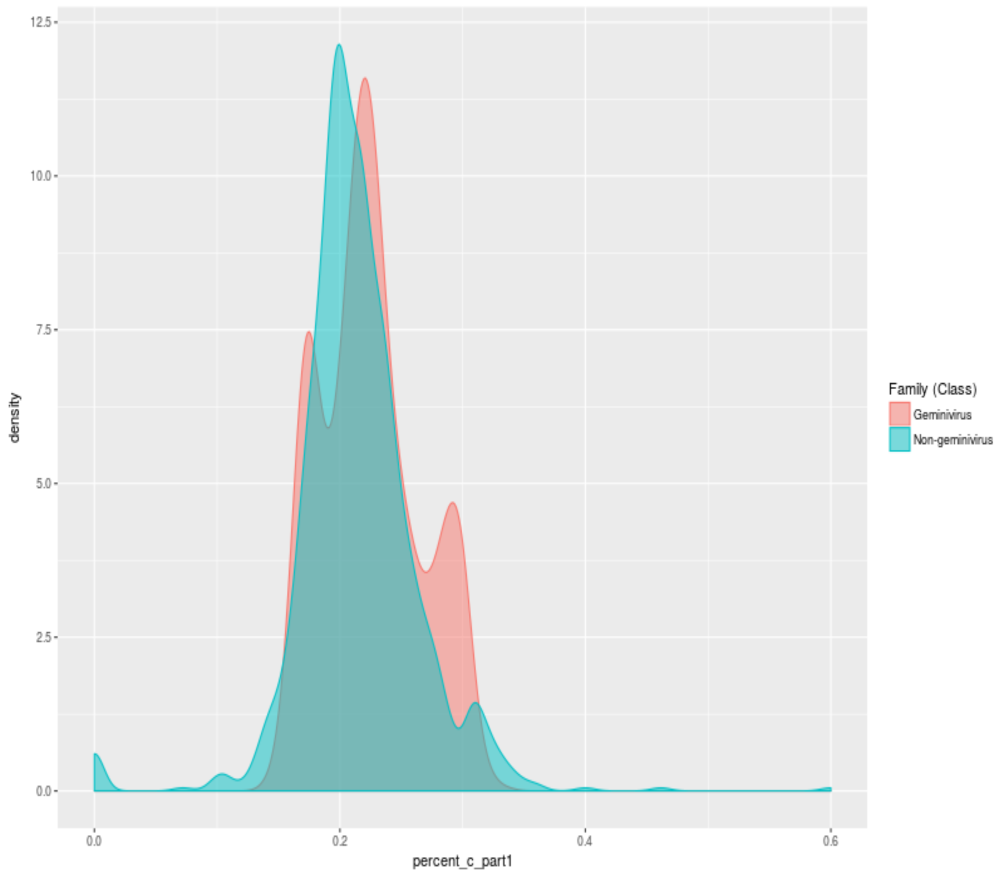

Boxplots

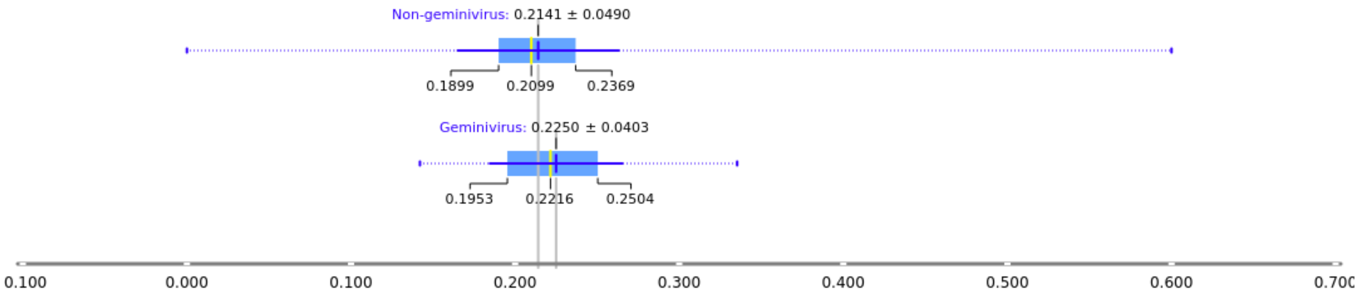

Non-geminivirus: 0.2141 ± 0.0490
0.1899
0.2099
0.2369
Geminivirus: 0.2250 ± 0.0403
0.1953
0.2216
0.2504
0.100
0.000
0.100
0.200
0.300
0.400
0.500
0.600
0.700

Proportion of Cytosine in region 2

Histogram

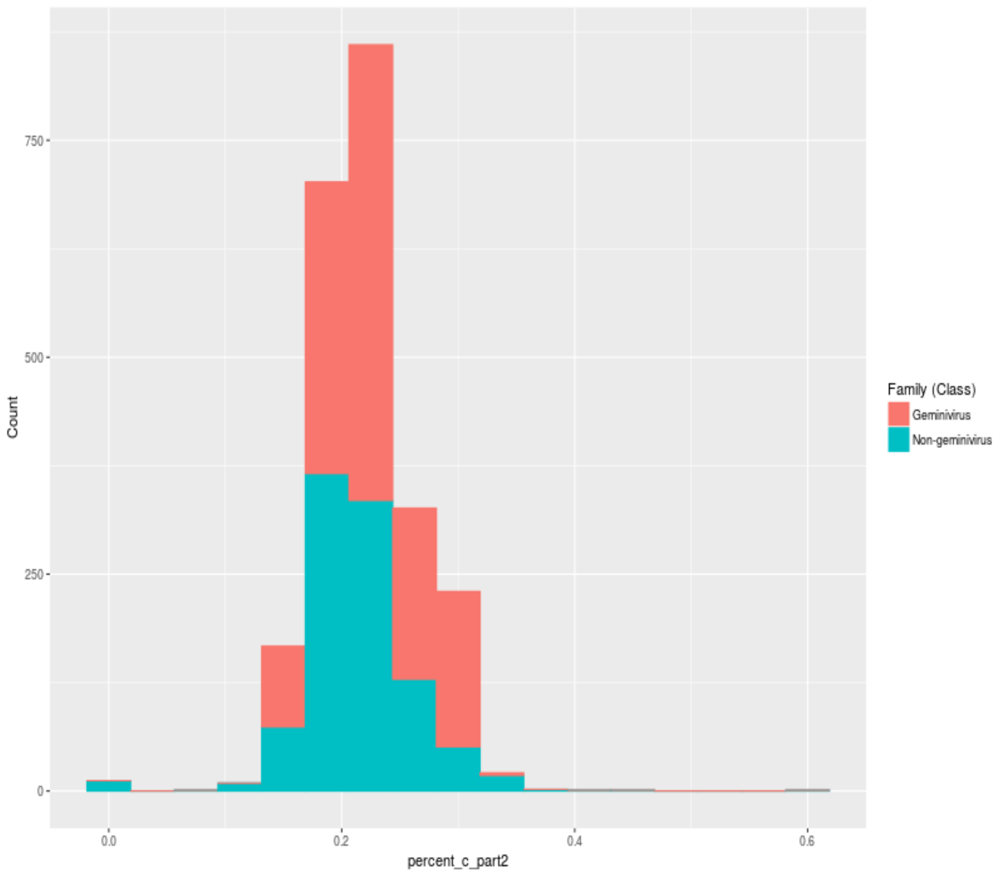

Density

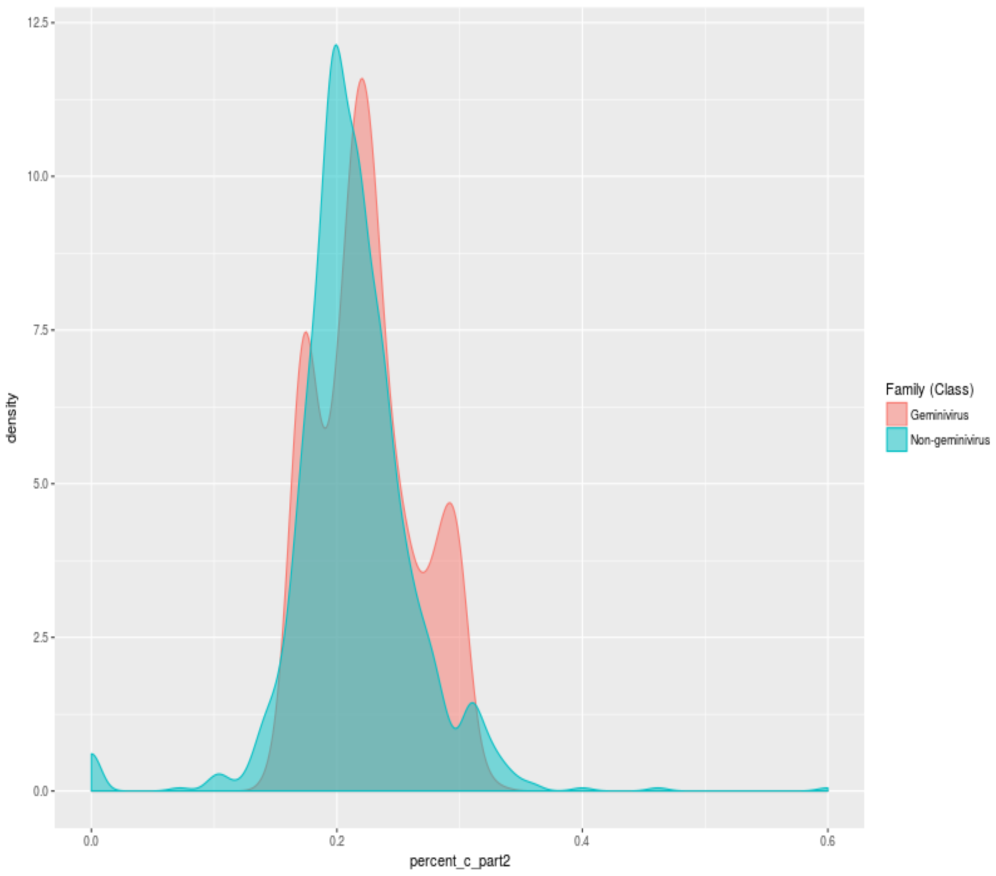

Boxplots

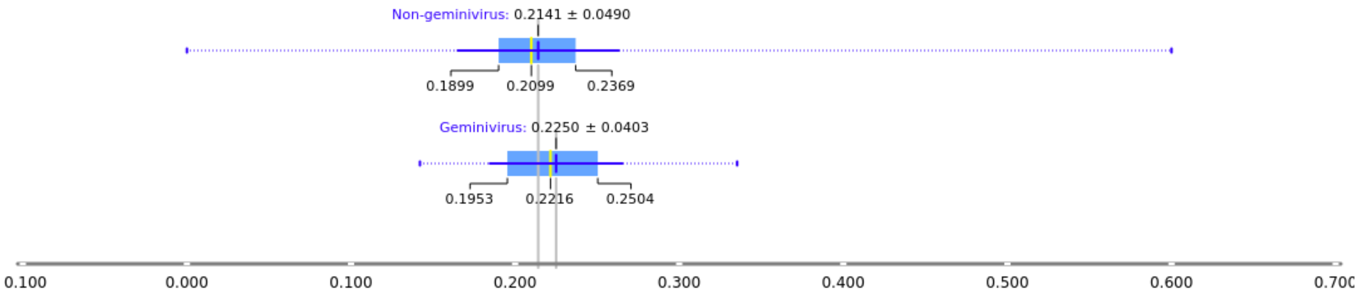

Non-geminivirus: 0.2141 ± 0.0490
0.1899
0.2099
0.2369
Geminivirus: 0.2250 ± 0.0403
0.1953
0.2216
0.2504
0.100
0.000
0.100
0.200
0.300
0.400
0.500
0.600
0.700

Proportion of Cytosine in region 3

Histogram

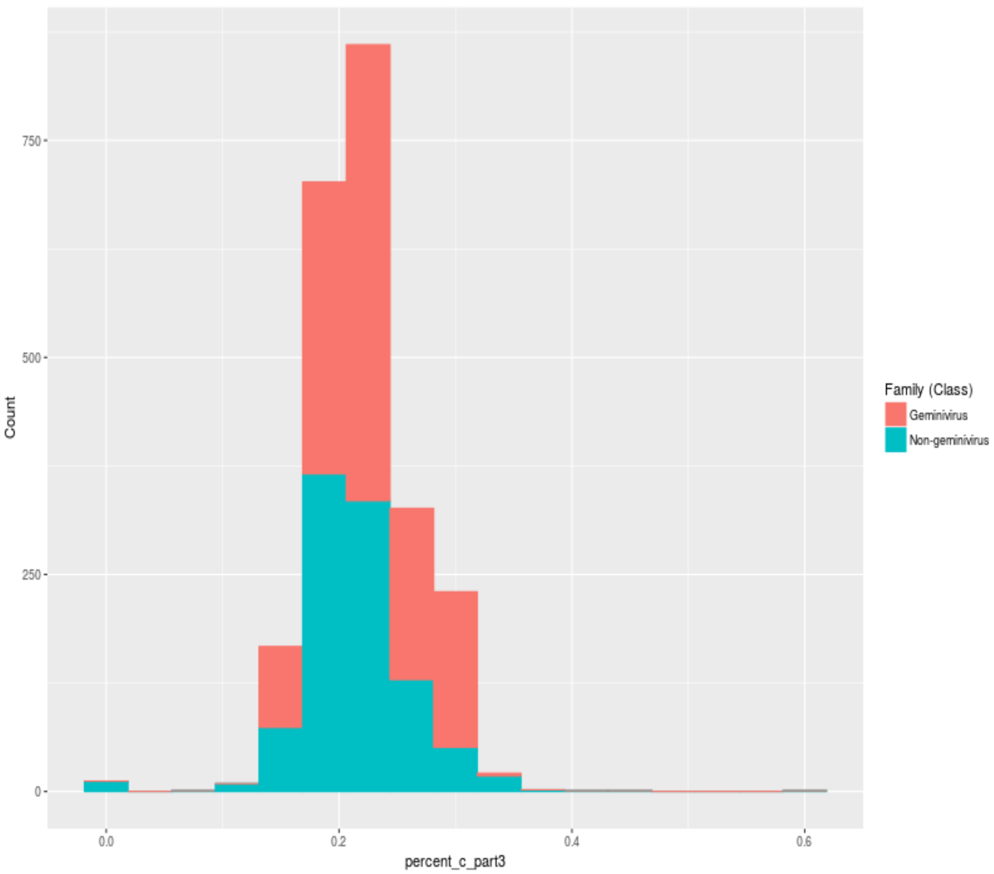

Density

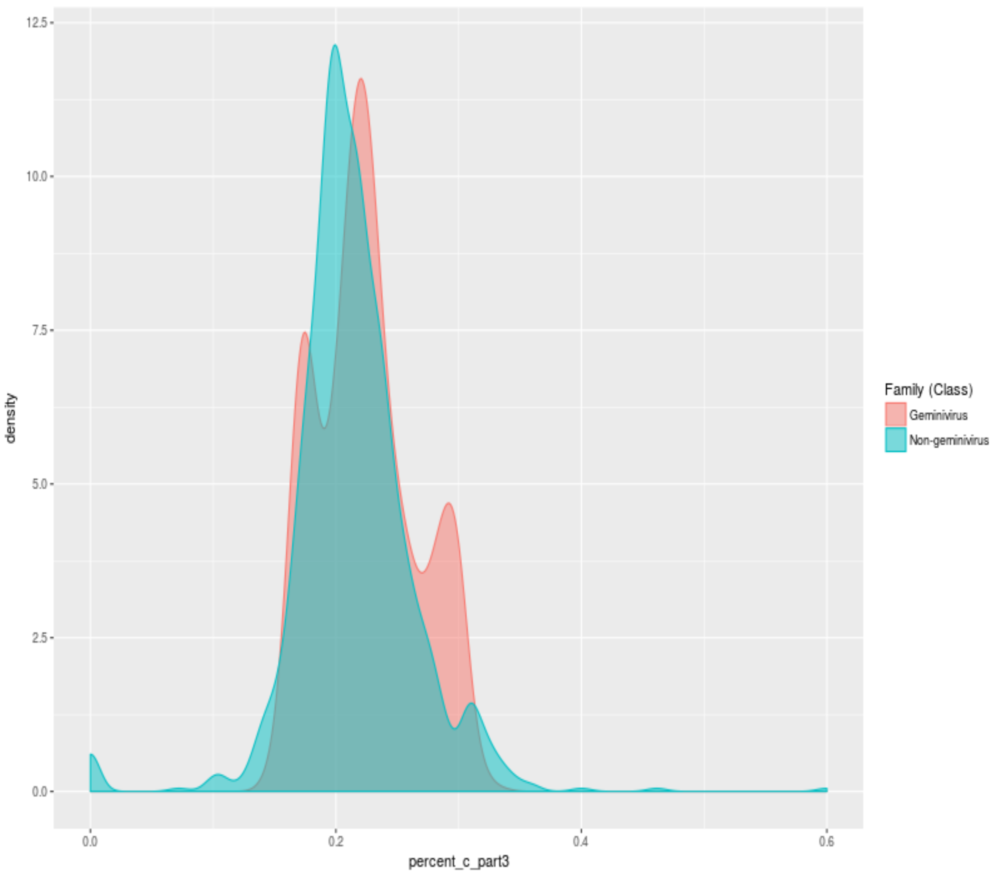

Boxplots

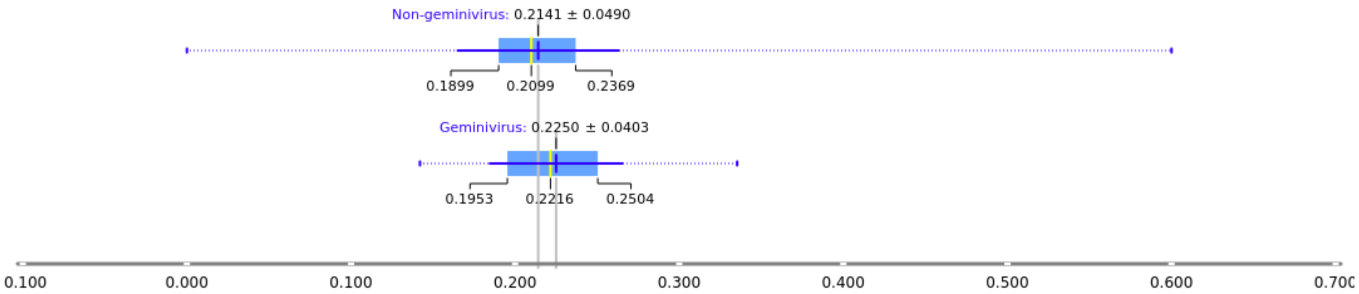
Non-geminivirus: 0.2141 ± 0.0490
0.1899
0.2099
0.2369
Geminivirus: 0.2250 ± 0.0403
0.1953
0.2216
0.2504
0.100
0.000
0.100
0.200
0.300
0.400
0.500
0.600
0.700

Proportion of Cytosine in region 4

Histogram

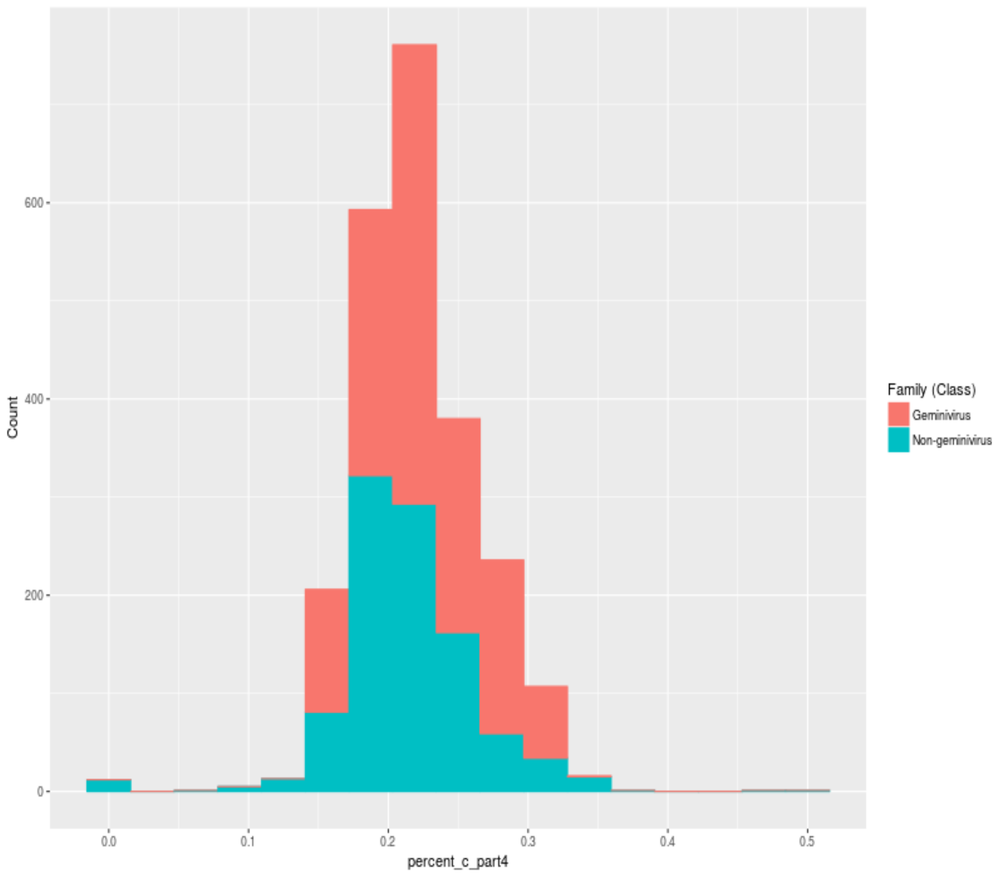

Density

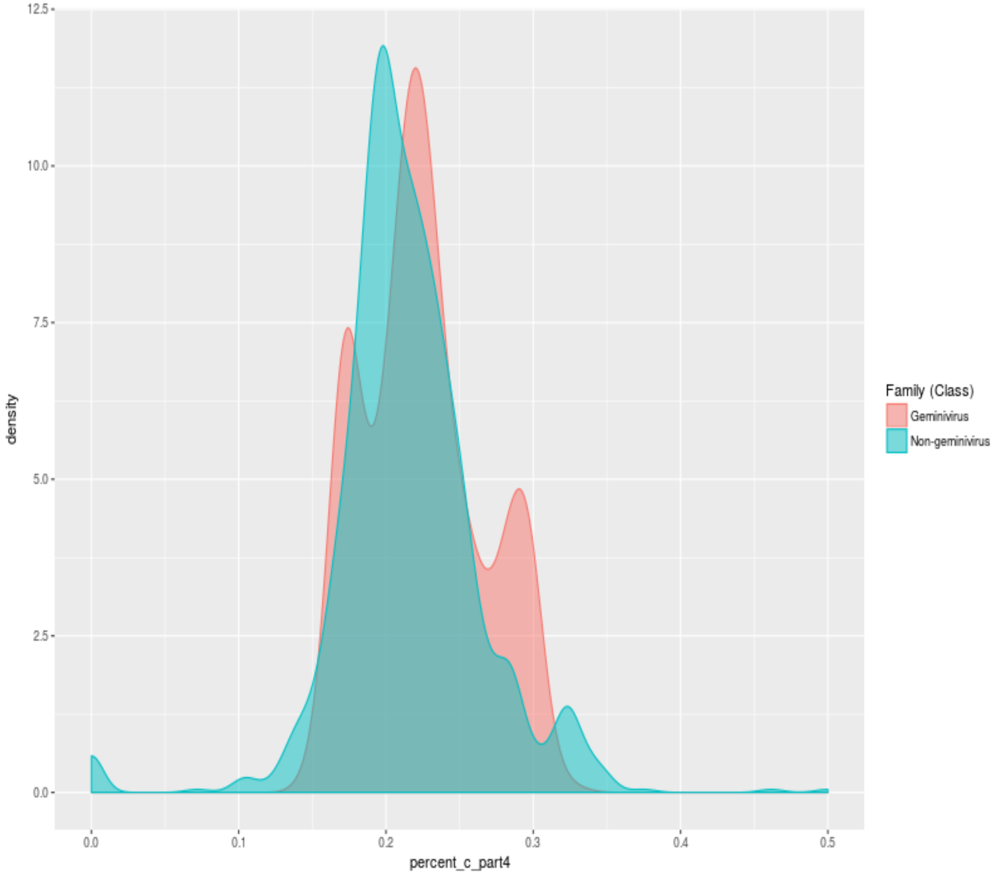

Boxplots

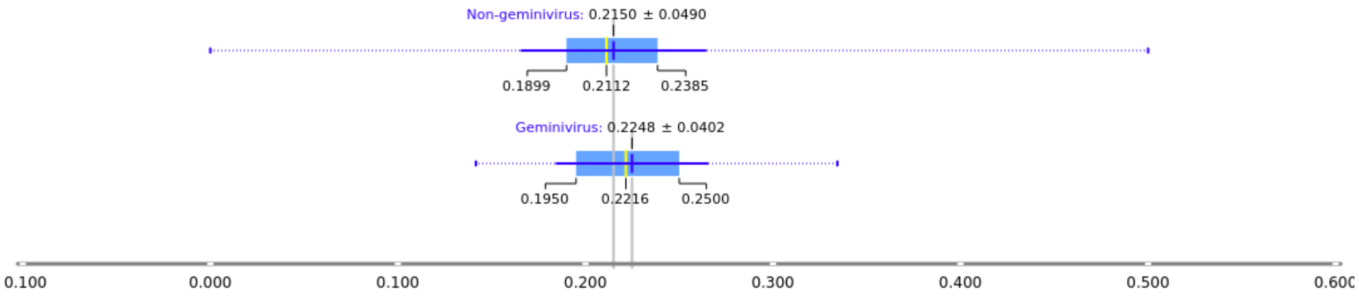

Non-geminivirus: 0.2150 ± 0.0490
0.1899
0.2112
0.2385
Geminivirus: 0.2248 ± 0.0402
0.1950
0.2216
0.2500
0.100
0.000
0.100
0.200
0.300
0.400
0.500
0.600

Proportion of Guanine and Cytosine in region 1

Histogram

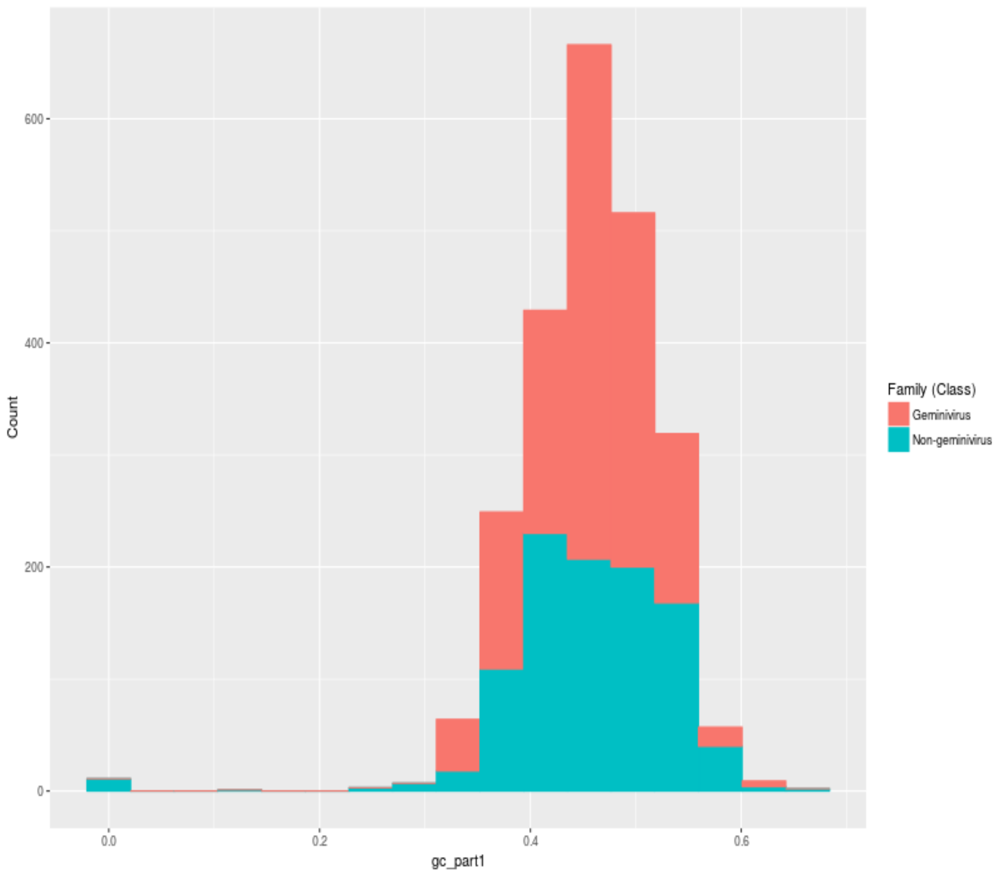

Density

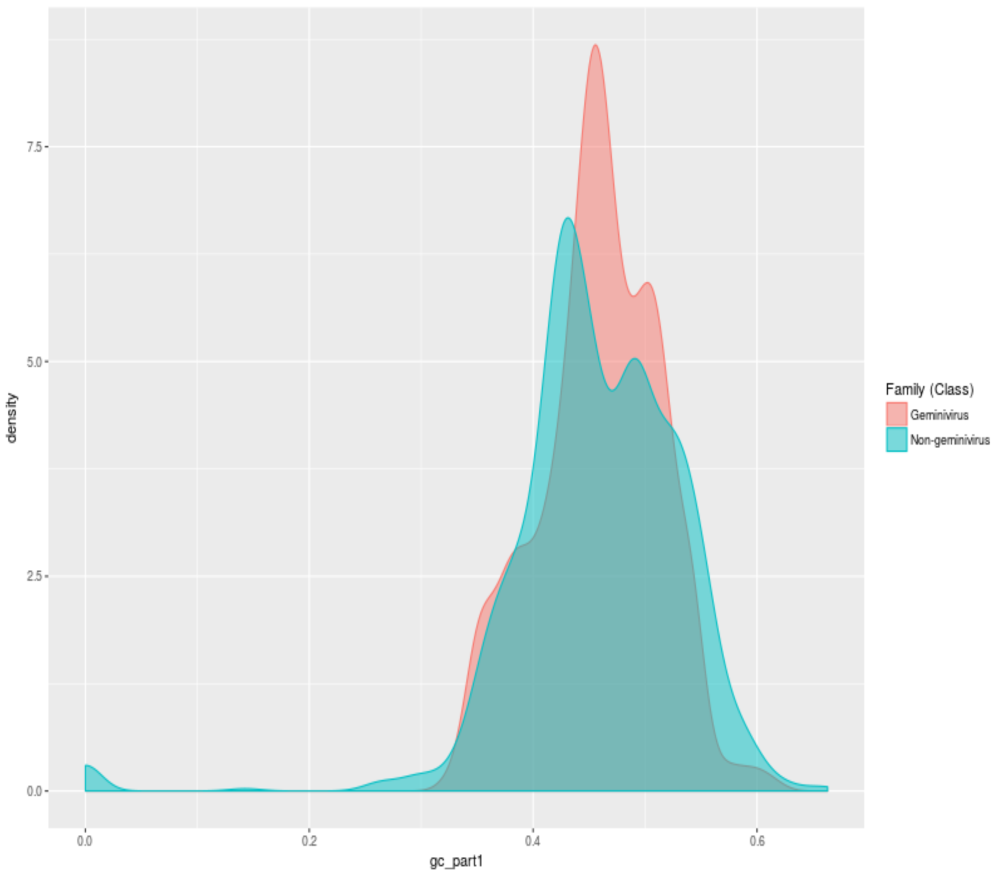

Boxplots

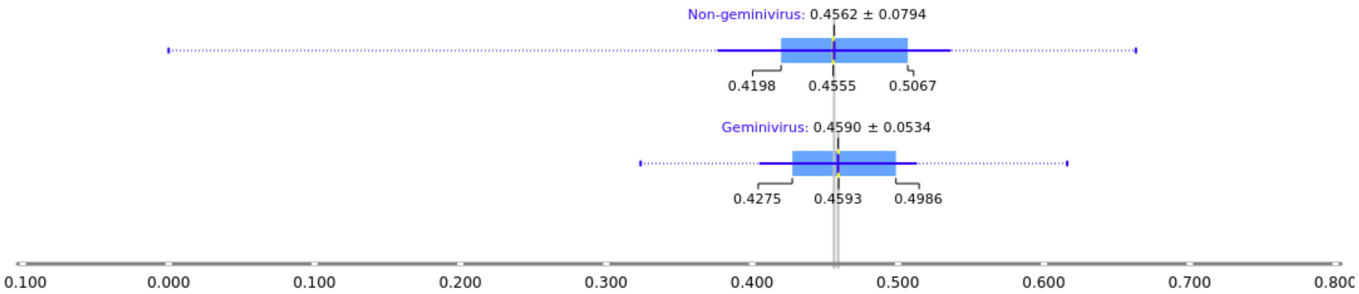

Non-geminivirus: 0.4562 ± 0.0794
0.4198
0.4555
0.5067
Geminivirus: 0.4590 ± 0.0534
0.4275
0.4593
0.4986
0.100
0.000
0.100
0.200
0.300
0.400
0.500
0.600
0.700
0.800

Proportion of Guanine and Cytosine in region 2

Histogram

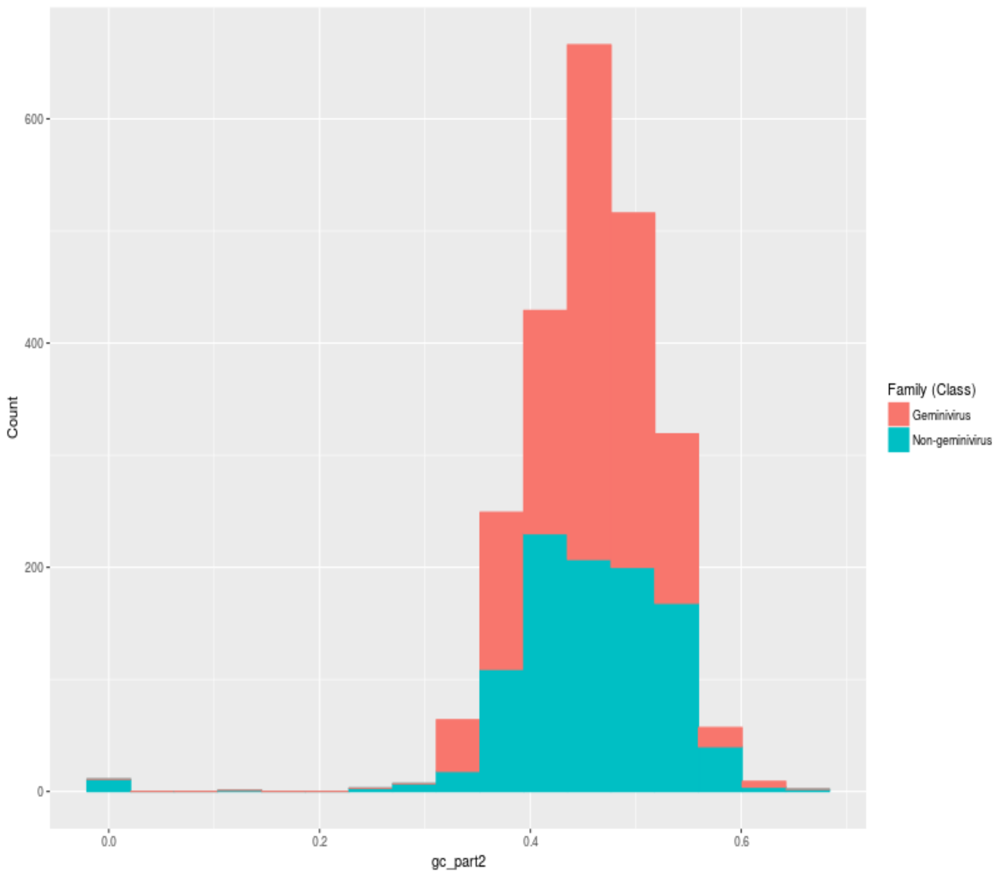

Density

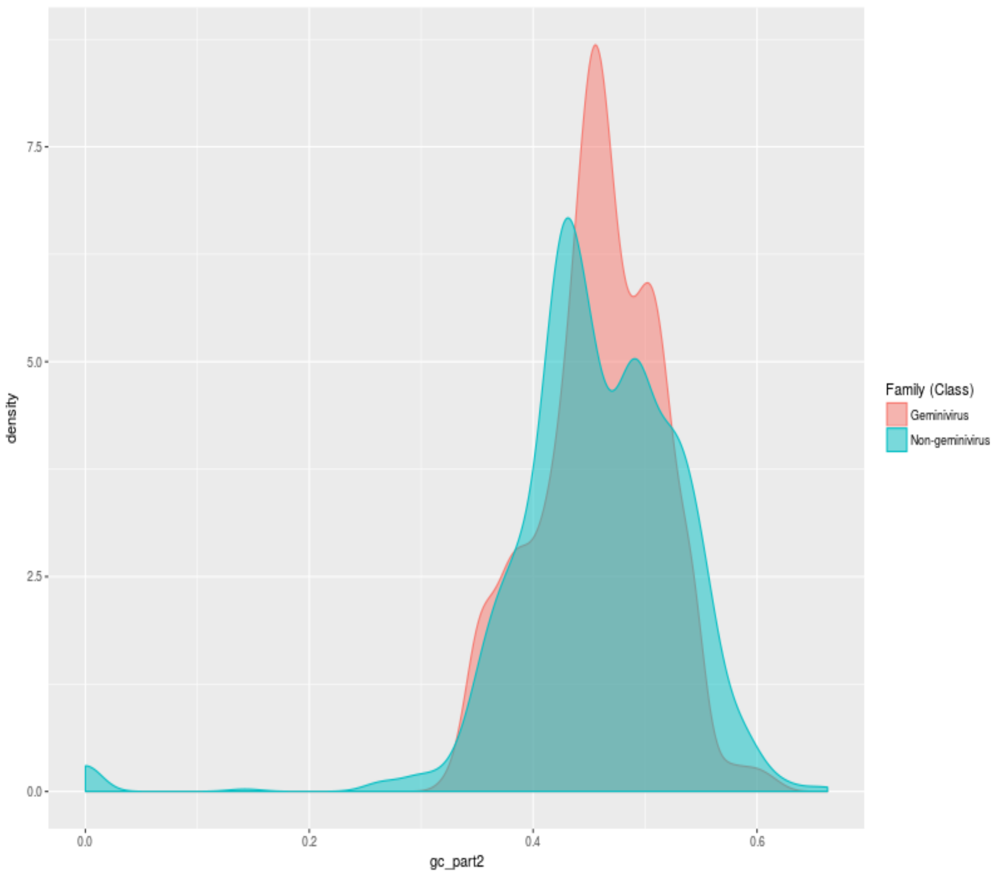

Boxplots

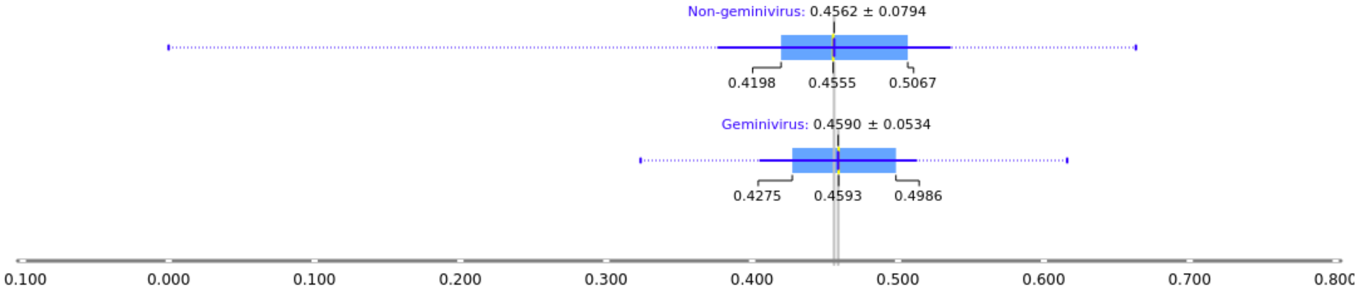

Non-geminivirus: 0.4562 ± 0.0794
0.4198
0.4555
0.5067
Geminivirus: 0.4590 ± 0.0534
0.4275
0.4593
0.4986
0.100
0.000
0.100
0.200
0.300
0.400
0.500
0.600
0.700
0.800

Proportion of Guanine and Cytosine in region 3

Histogram

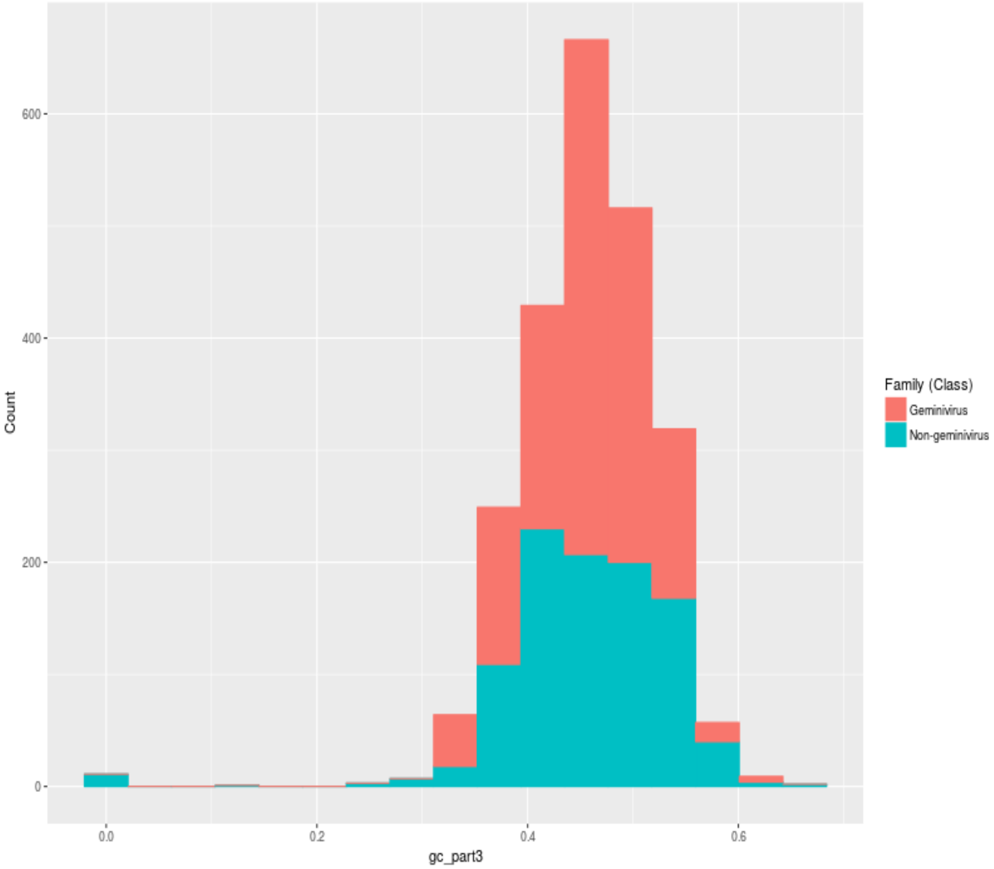

Density

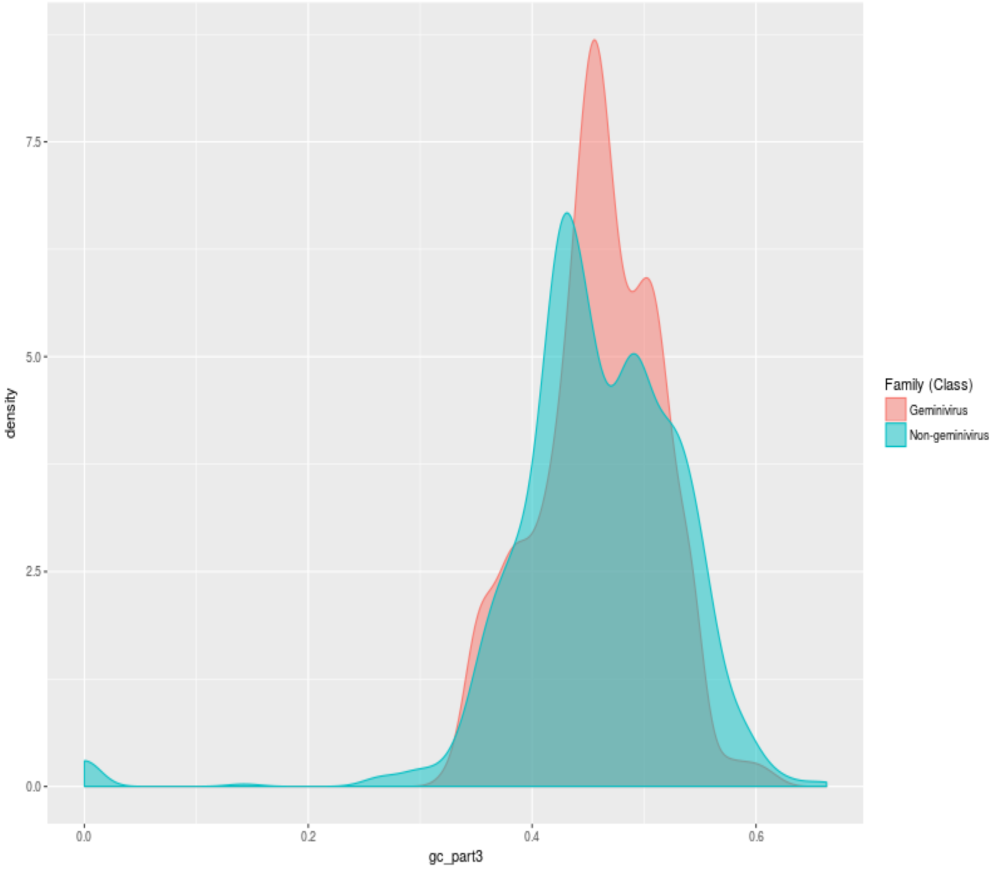

Boxplots

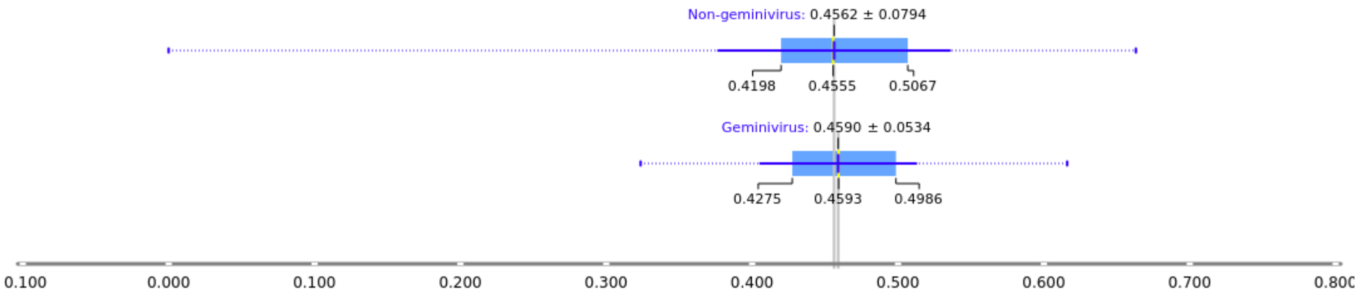
Non-geminivirus: 0.4562 ± 0.0794
0.4198
0.4555
0.5067
Geminivirus: 0.4590 ± 0.0534
0.4275
0.4593
0.4986
0.100
0.000
0.100
0.200
0.300
0.400
0.500
0.600
0.700
0.800

Proportion of Guanine and Cytosine in region 4

Histogram

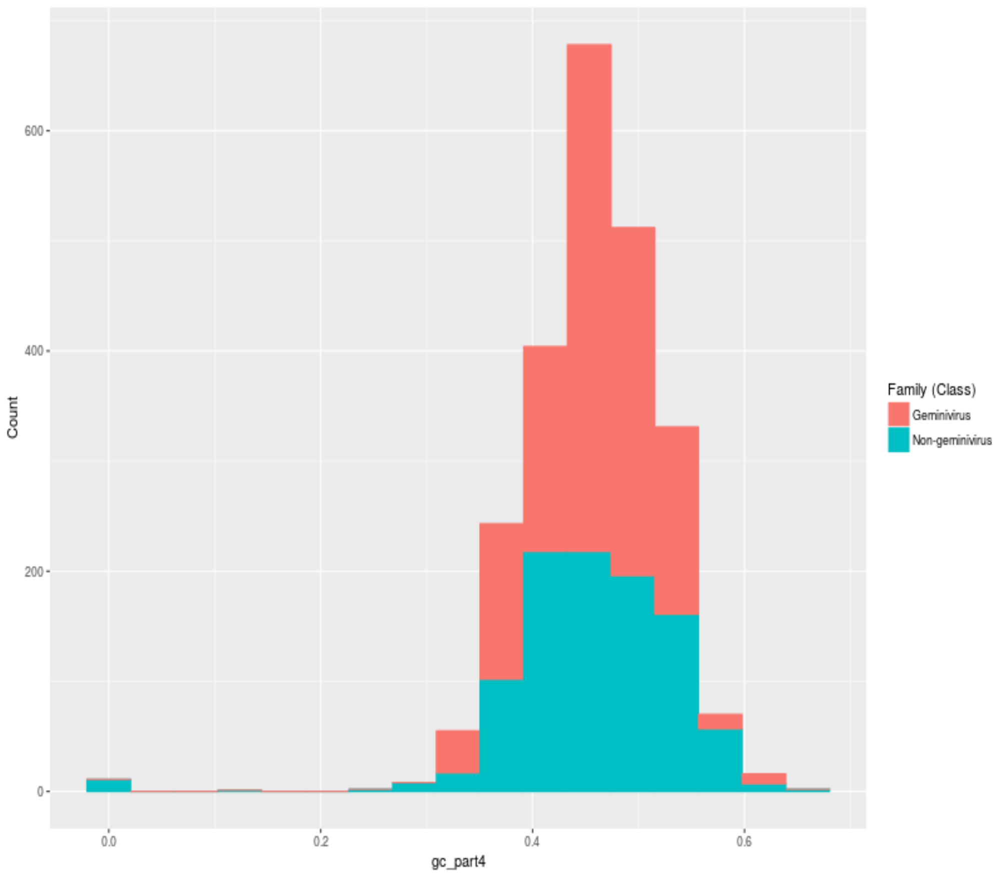

Density

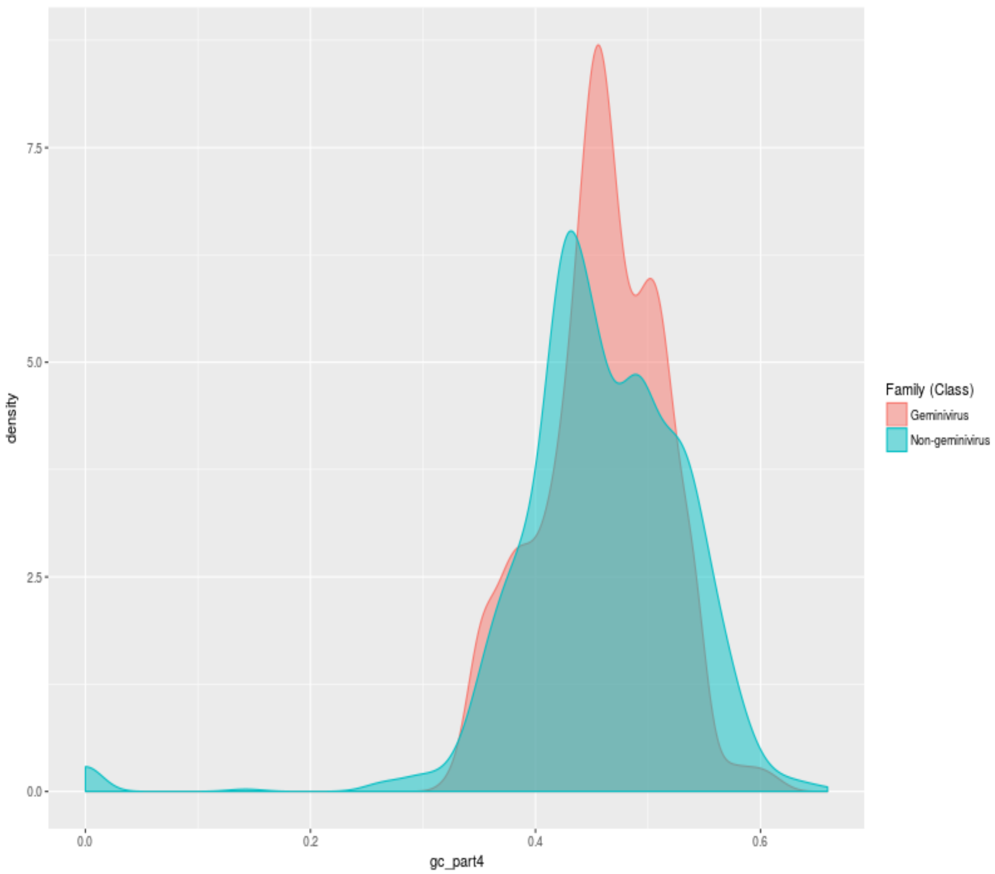

Boxplots

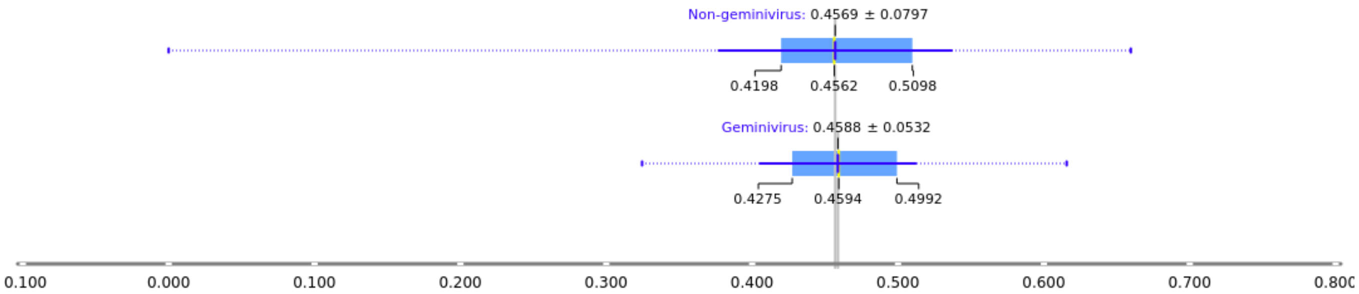

Non-geminivirus: 0.4569 ± 0.0797
0.4198
0.4562
0.5098
Geminivirus: 0.4588 ± 0.0532
0.4275
0.4594
0.4992
0.100
0.000
0.100
0.200
0.300
0.400
0.500
0.600
0.700
0.800

Charts of genus attributes

Proportion of Guanine in the genome

Histogram

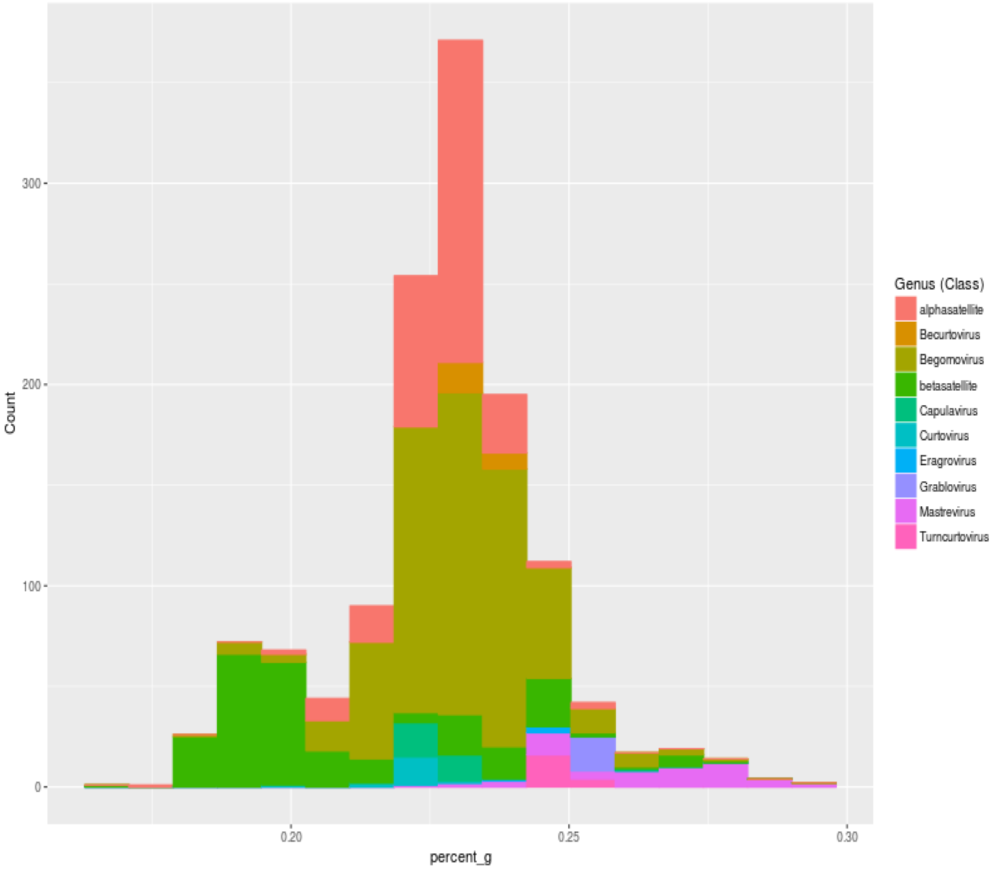

Density

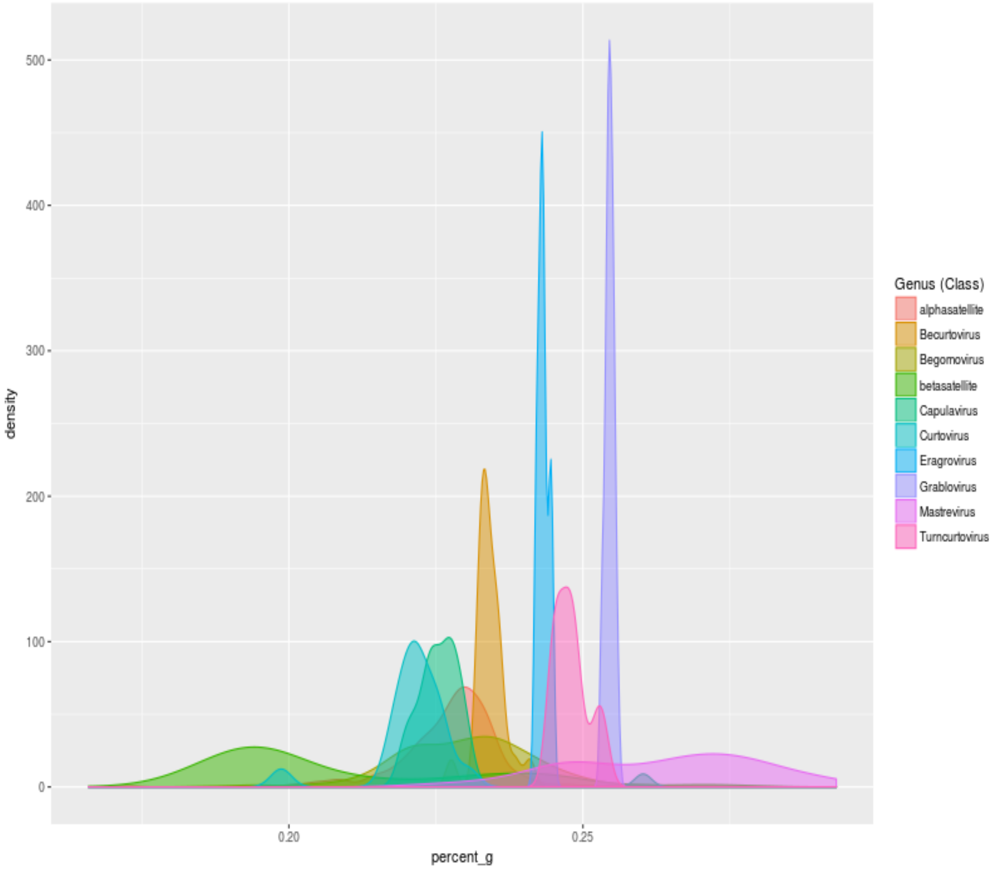

Boxplots

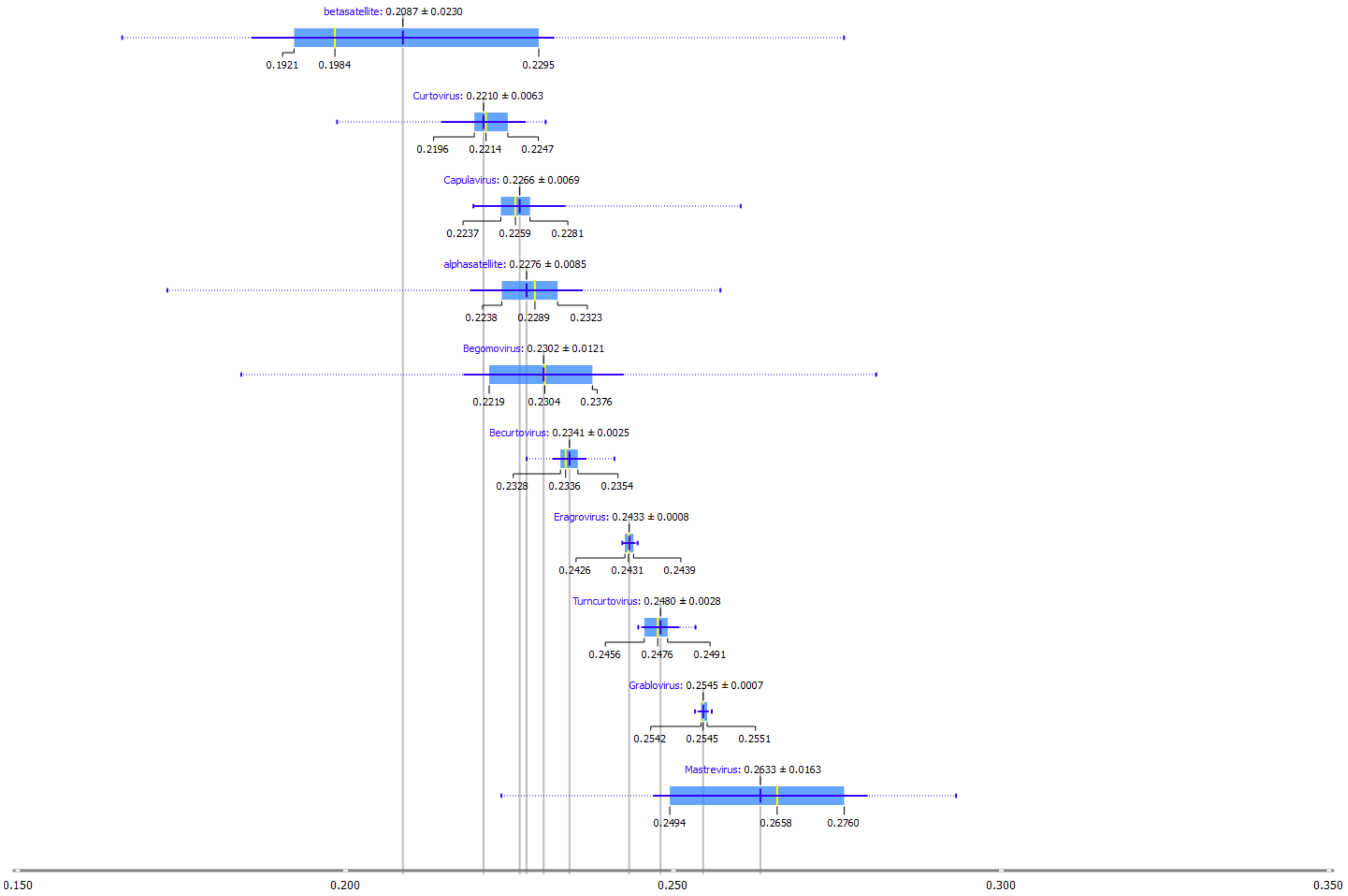

betasatellite: 0.2087 ± 0.0230
0.1921
0.1984
0.2295
Curtovirus: 0.2210 ± 0.0063
0.2196
0.2214
0.2247
Capulavirus: 0.2266 ± 0.0069
0.2237
0.2259
0.2281
alphasatellite: 0.2276 ± 0.0085
0.2238
0.2289
0.2323
Begomovirus: 0.2302 ± 0.0121
0.2219
0.2304
0.2376
Becurtovirus: 0.2341 ± 0.0025
0.2328
0.2336
0.2354
Eragrovirus: 0.2433 ± 0.0008
0.2426
0.2431
0.2439
Turncurtovirus: 0.2480 ± 0.0028
0.2456
0.2476
0.2491
Grablovirus: 0.2545 ± 0.0007
0.2542
0.2545
0.2551
Mastrevirus: 0.2633 ± 0.0163
0.2494
0.2658
0.2760
0.150
0.200
0.250
0.300
0.350

Proportion of Guanine in region 1

Histogram

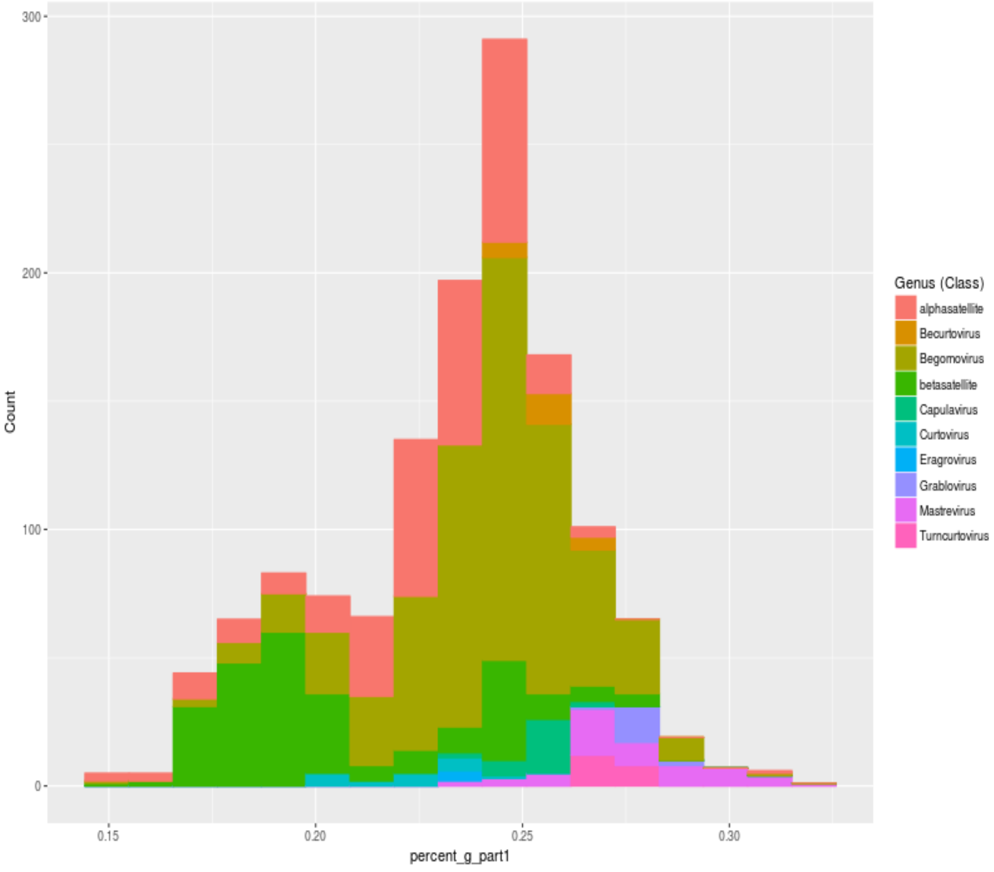

Density

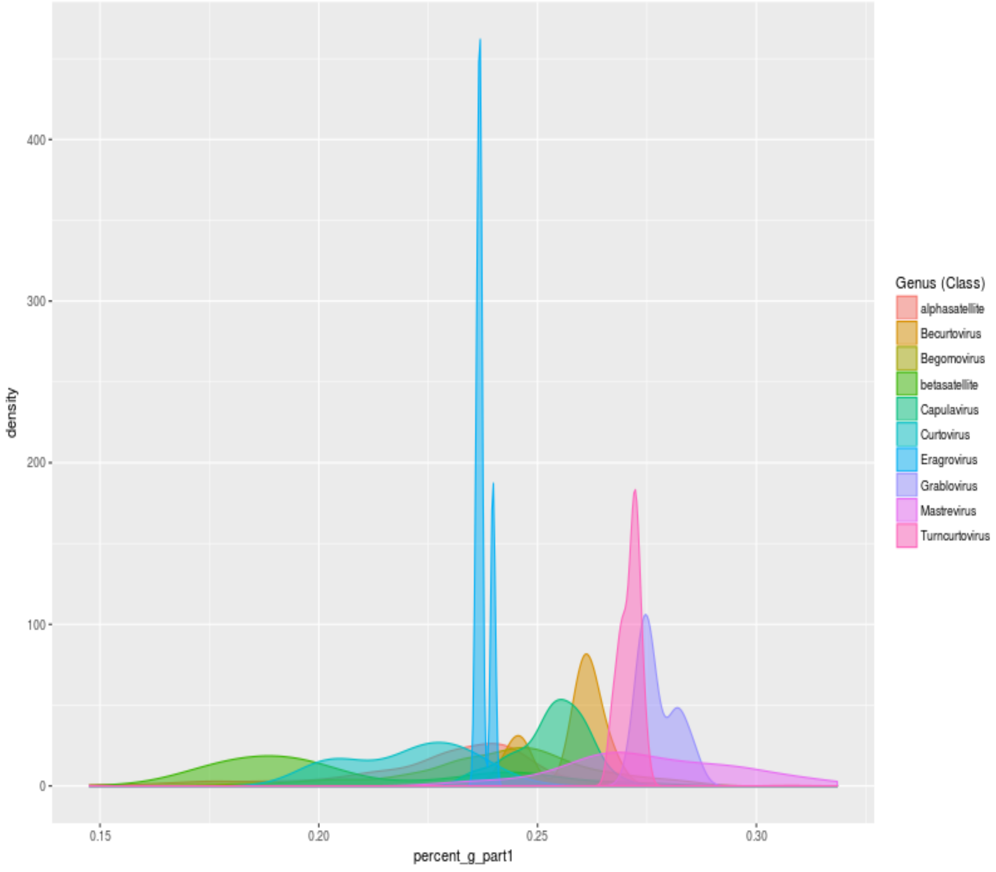

Boxplots

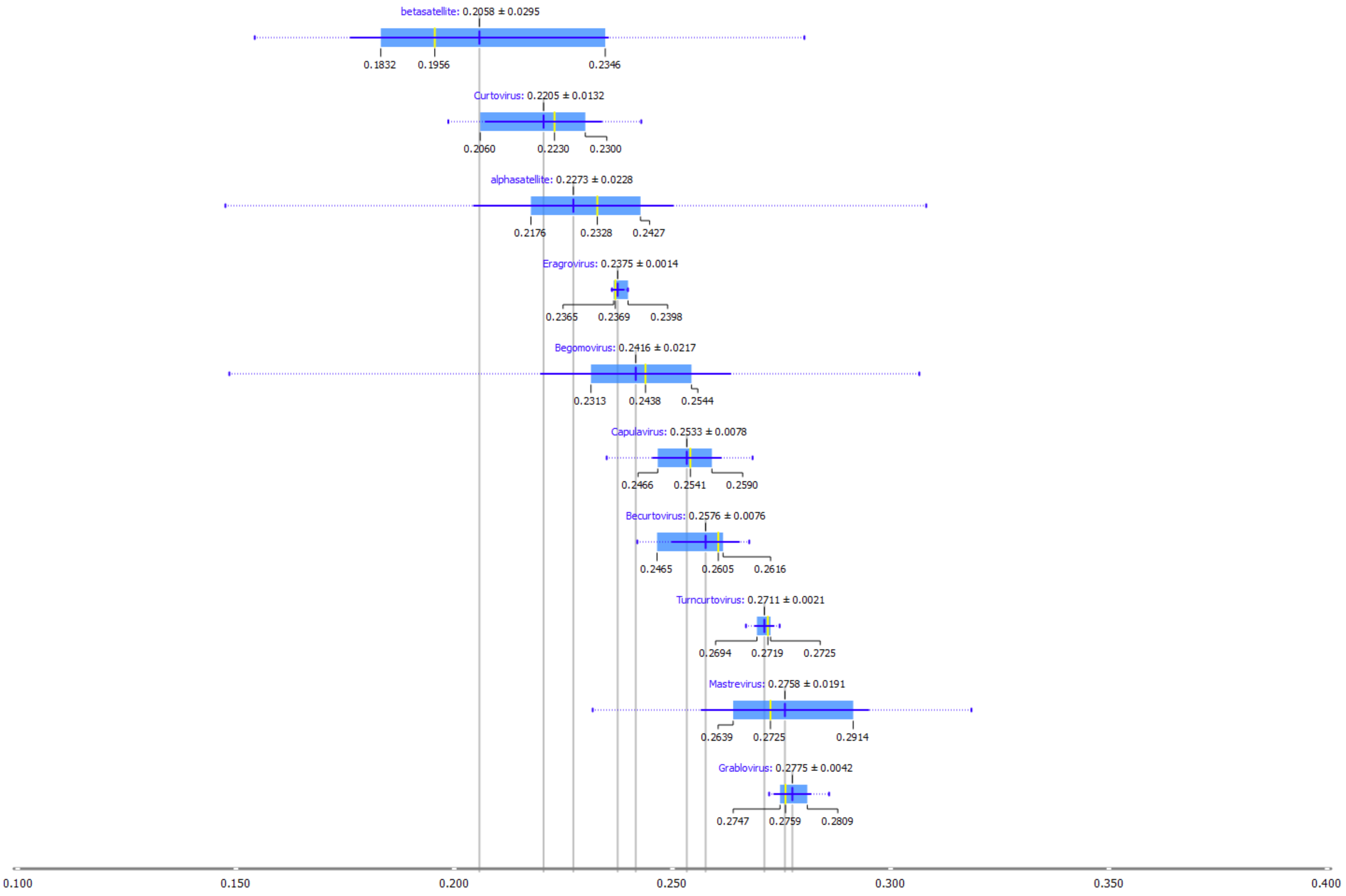

betasatellite: 0.2058 ± 0.0295
0.1832
0.1956
0.2346
Curtovirus: 0.2205 ± 0.0132
0.2060
0.2230
0.2300
alphasatellite: 0.2273 ± 0.0228
0.2176
0.2328
0.2427
Eragrovirus: 0.2375 ± 0.0014
0.2365
0.2369
0.2398
Begomovirus: 0.2416 ± 0.0217
0.2313
0.2438
0.2544
Capulavirus: 0.2533 ± 0.0078
0.2466
0.2541
0.2590
Becurtovirus: 0.2576 ± 0.0076
0.2465
0.2605
0.2616
Turncurtovirus: 0.2711 ± 0.0021
0.2694
0.2719
0.2725
Mastrevirus: 0.2758 ± 0.0191
0.2639
0.2725
0.2914
Grablovirus: 0.2775 ± 0.0042
0.2747
0.2759
0.2809
0.100
0.150
0.200
0.250
0.300
0.350
0.400

Proportion of Guanine in region 2

Histogram

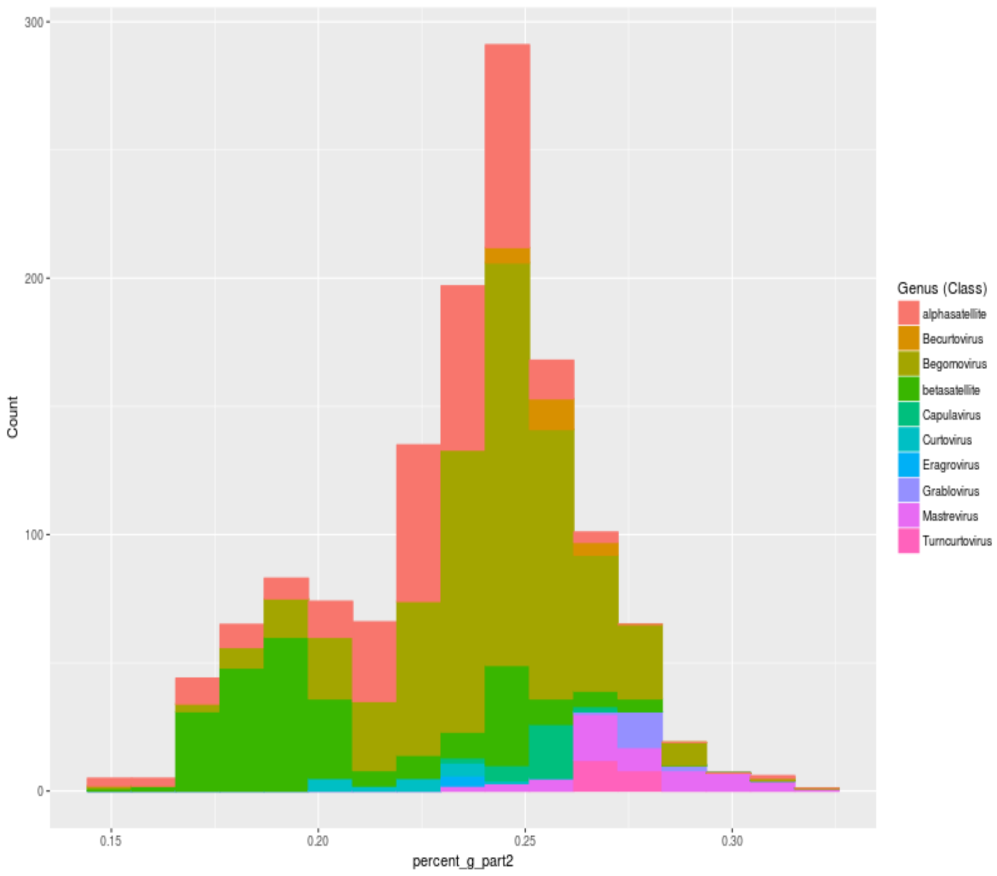

Density

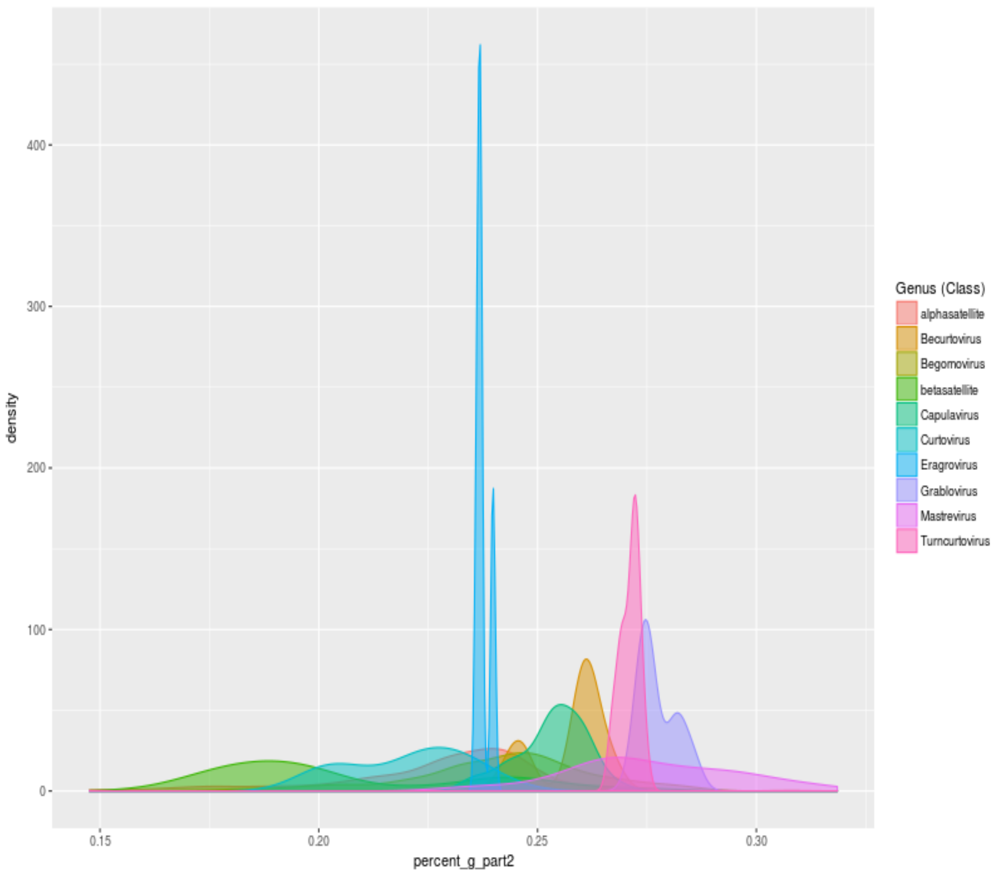

Boxplots

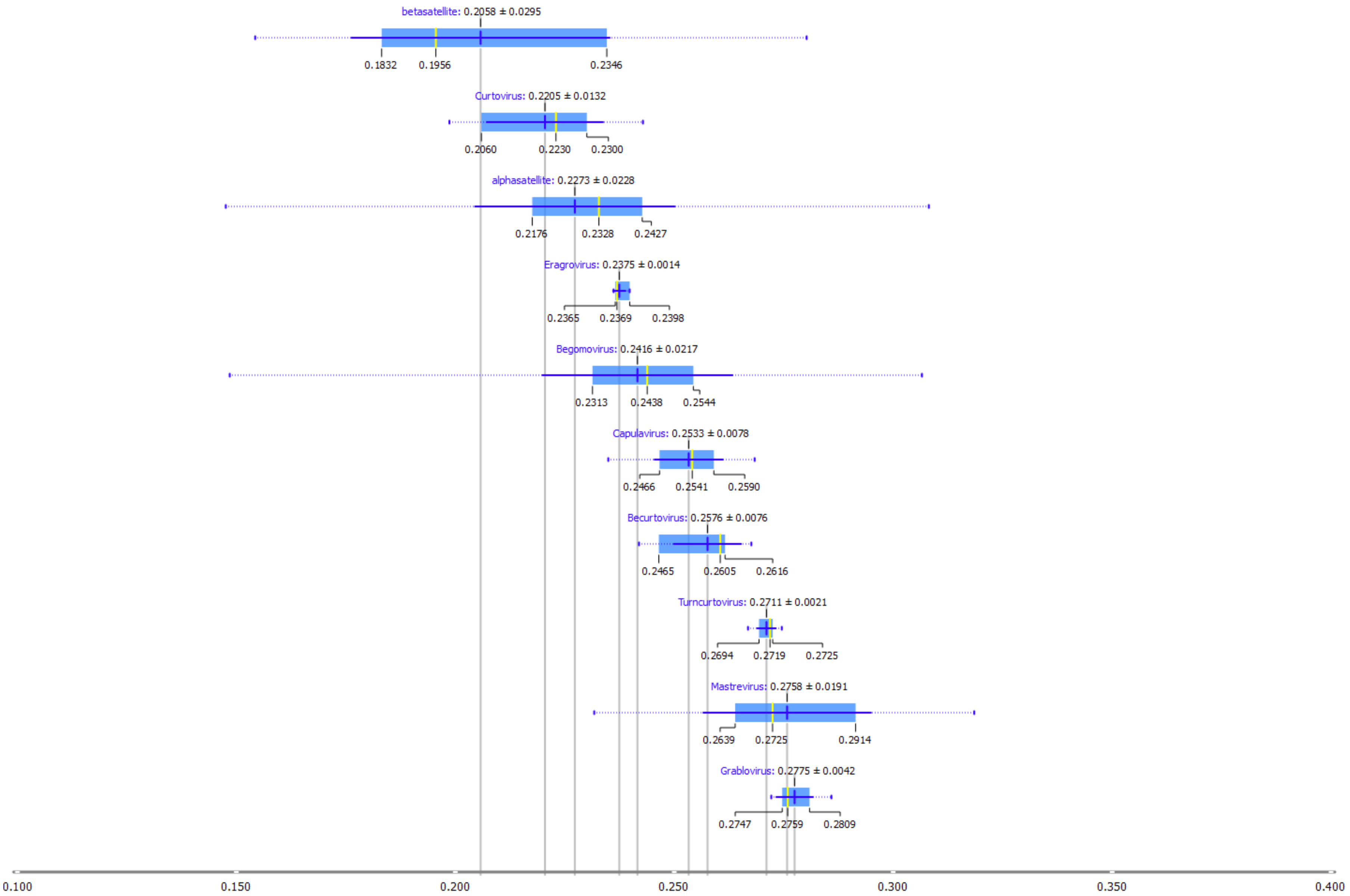

betasatellite: 0.2058 ± 0.0295
0.1832
0.1956
0.2346
Curtovirus: 0.2205 ± 0.0132
0.2060
0.2230
0.2300
alphasatellite: 0.2273 ± 0.0228
0.2176
0.2328
0.2427
Eragrovirus: 0.2375 ± 0.0014
0.2365
0.2369
0.2398
Begomovirus: 0.2416 ± 0.0217
0.2313
0.2438
0.2544
Capulavirus: 0.2533 ± 0.0078
0.2466
0.2541
0.2590
Becurtovirus: 0.2576 ± 0.0076
0.2465
0.2605
0.2616
Turncurtovirus: 0.2711 ± 0.0021
0.2694
0.2719
0.2725
Mastrevirus: 0.2758 ± 0.0191
0.2639
0.2725
0.2914
Grablovirus: 0.2775 ± 0.0042
0.2747
0.2759
0.2809
0.100
0.150
0.200
0.250
0.300
0.350
0.400

Proportion of Guanine in region 3

Histogram

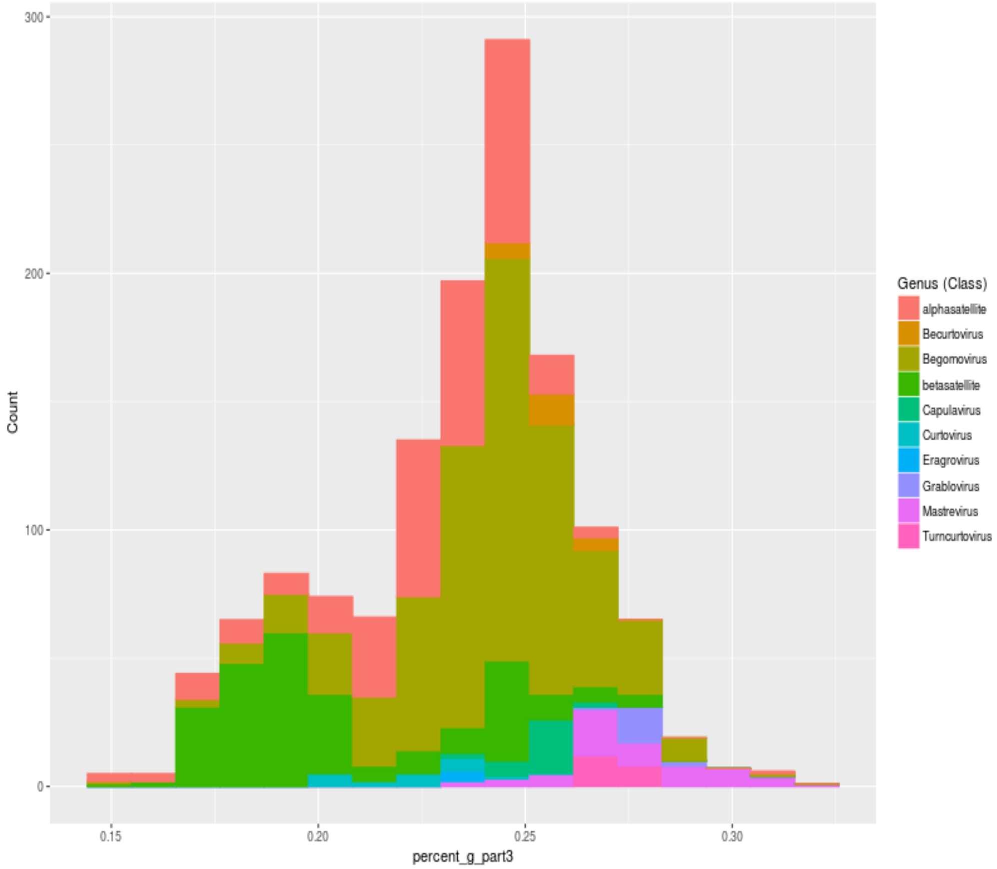

Density

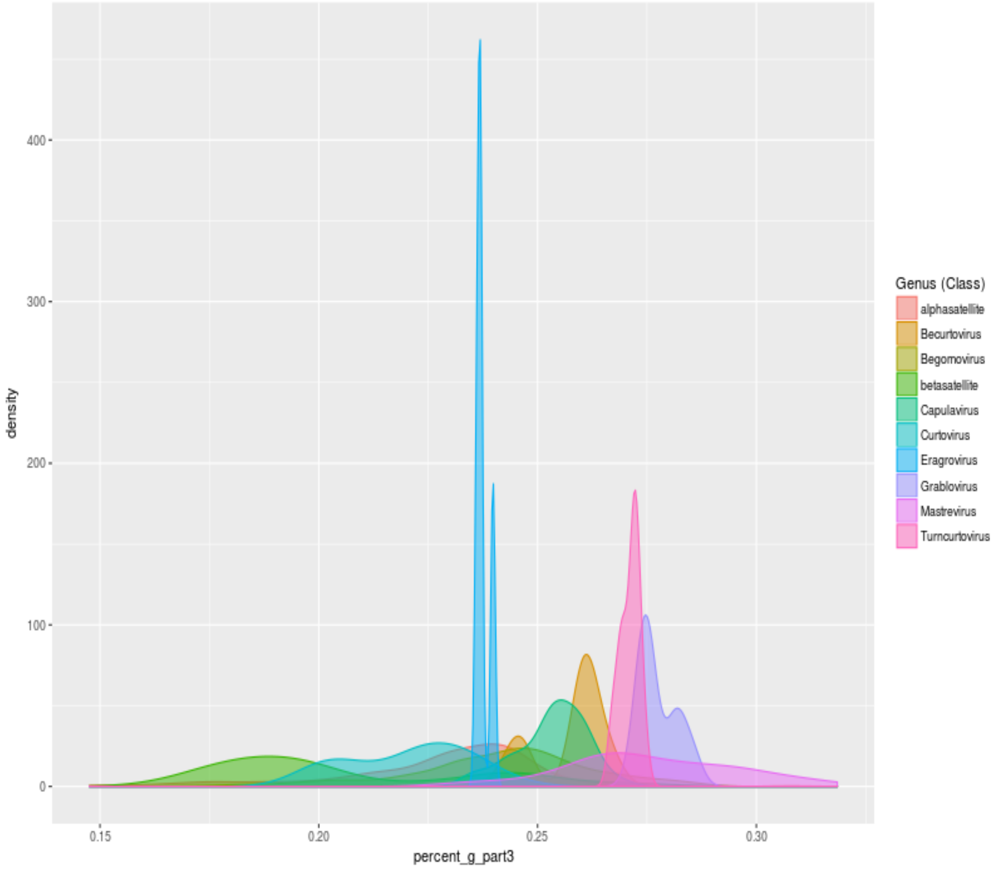

Boxplots

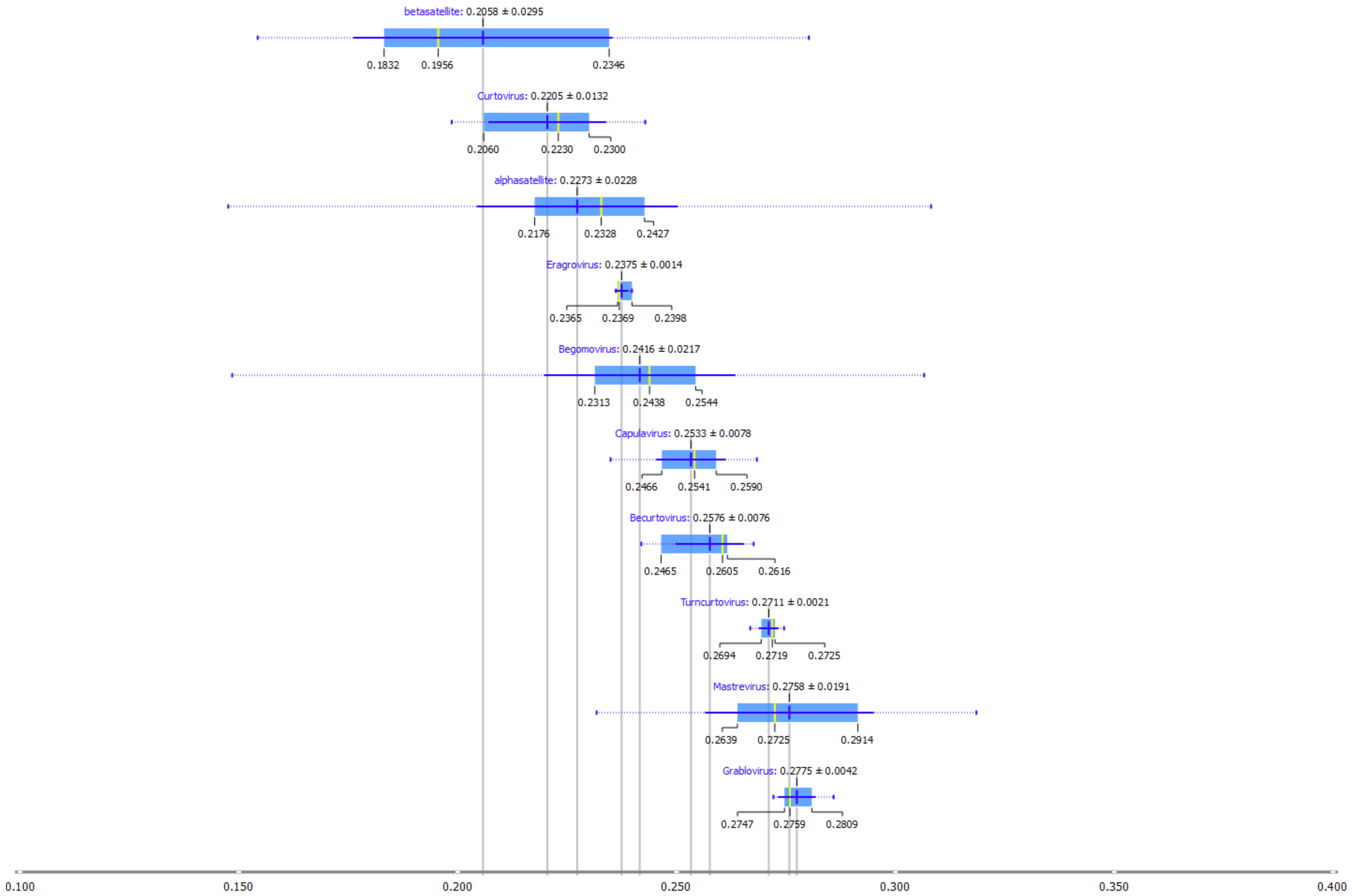

betasatellite: 0.2058 ± 0.0295
0.1832
0.1956
0.2346
Curtovirus: 0.2205 ± 0.0132
0.2060
0.2230
0.2300
alphasatellite: 0.2273 ± 0.0228
0.2176
0.2328
0.2427
Eragrovirus: 0.2375 ± 0.0014
0.2365
0.2369
0.2398
Begomovirus: 0.2416 ± 0.0217
0.2313
0.2438
0.2544
Capulavirus: 0.2533 ± 0.0078
0.2466
0.2541
0.2590
Becurtovirus: 0.2576 ± 0.0076
0.2465
0.2605
0.2616
Turncurtovirus: 0.2711 ± 0.0021
0.2694
0.2719
0.2725
Mastrevirus: 0.2758 ± 0.0191
0.2639
0.2725
0.2914
Grablovirus: 0.2775 ± 0.0042
0.2747
0.2759
0.2809
0.100
0.150
0.200
0.250
0.300
0.350
0.400

Proportion of Guanine in region 4

Histogram

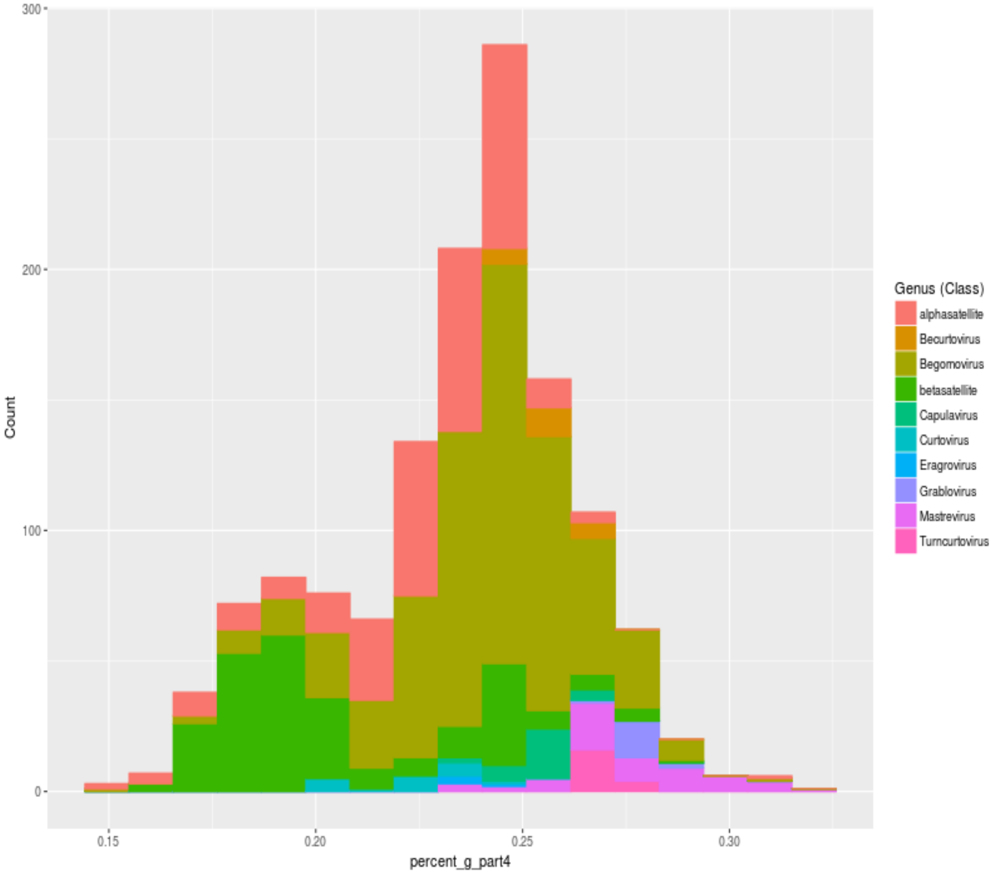

Density

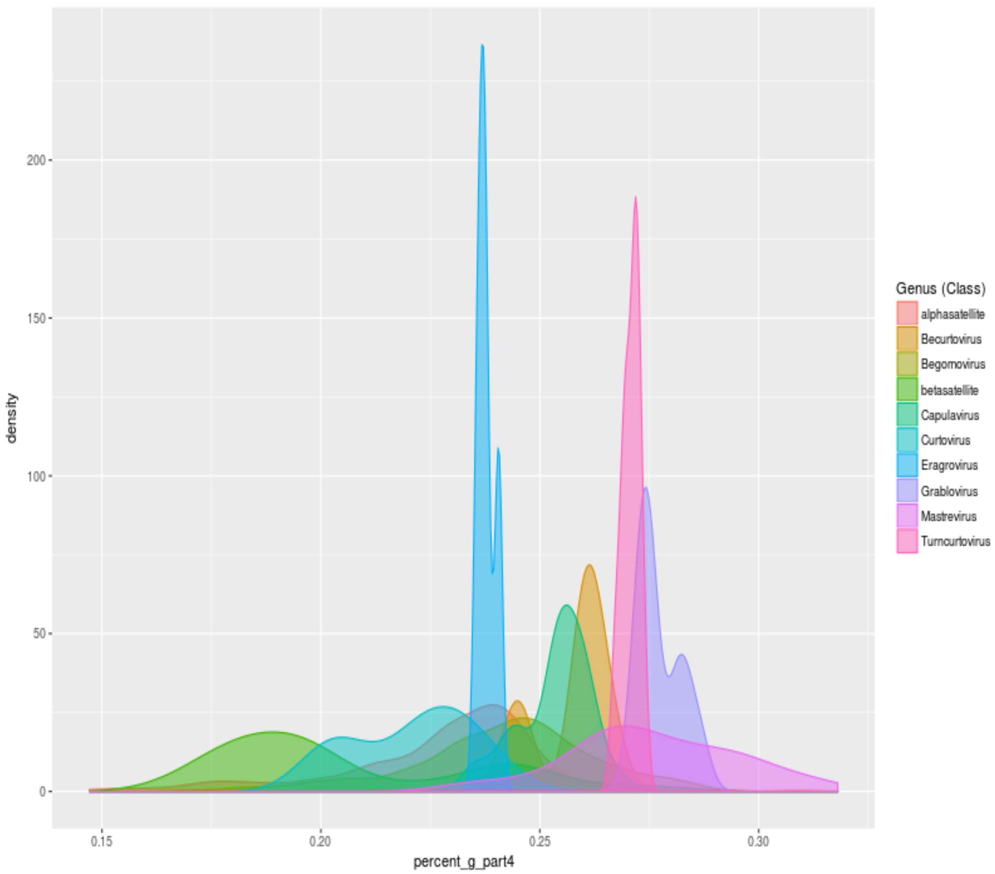

Boxplots

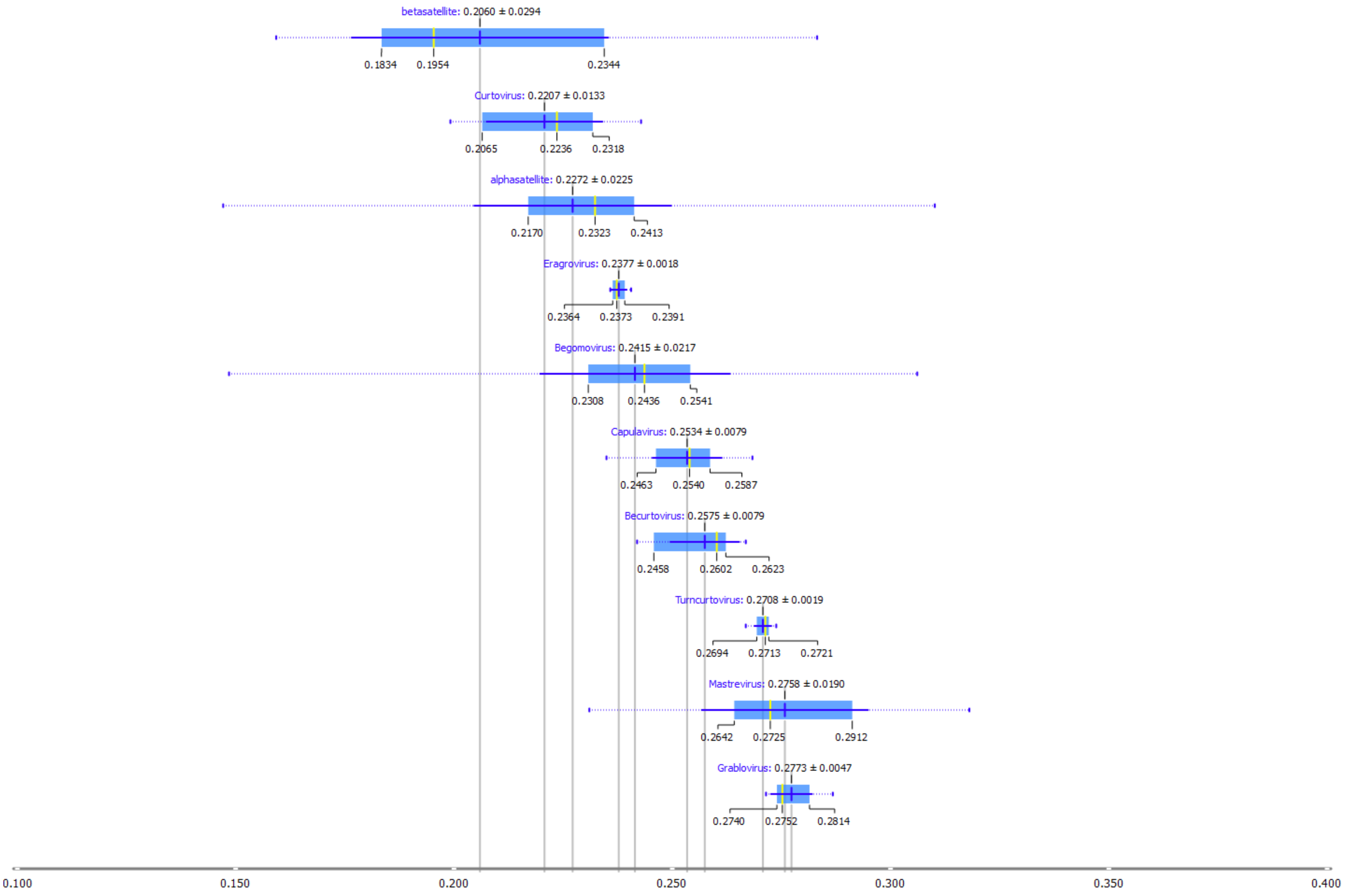

betasatellite: 0.2060 ± 0.0294
0.1834
0.1954
0.2344
Curtovirus: 0.2207 ± 0.0133
0.2065
0.2236
0.2318
alphasatellite: 0.2272 ± 0.0225
0.2170
0.2323
0.2413
Eragrovirus: 0.2377 ± 0.0018
0.2364
0.2373
0.2391
Begomovirus: 0.2415 ± 0.0217
0.2308
0.2436
0.2541
Capulavirus: 0.2534 ± 0.0079
0.2463
0.2540
0.2587
Becurtovirus: 0.2575 ± 0.0079
0.2458
0.2602
0.2623
Turncurtovirus: 0.2708 ± 0.0019
0.2694
0.2713
0.2721
Mastrevirus: 0.2758 ± 0.0190
0.2642
0.2725
0.2912
Grablovirus: 0.2773 ± 0.0047
0.2740
0.2752
0.2814
0.100
0.150
0.200
0.250
0.300
0.350
0.400

Proportion of Adenine in the genome

Histogram

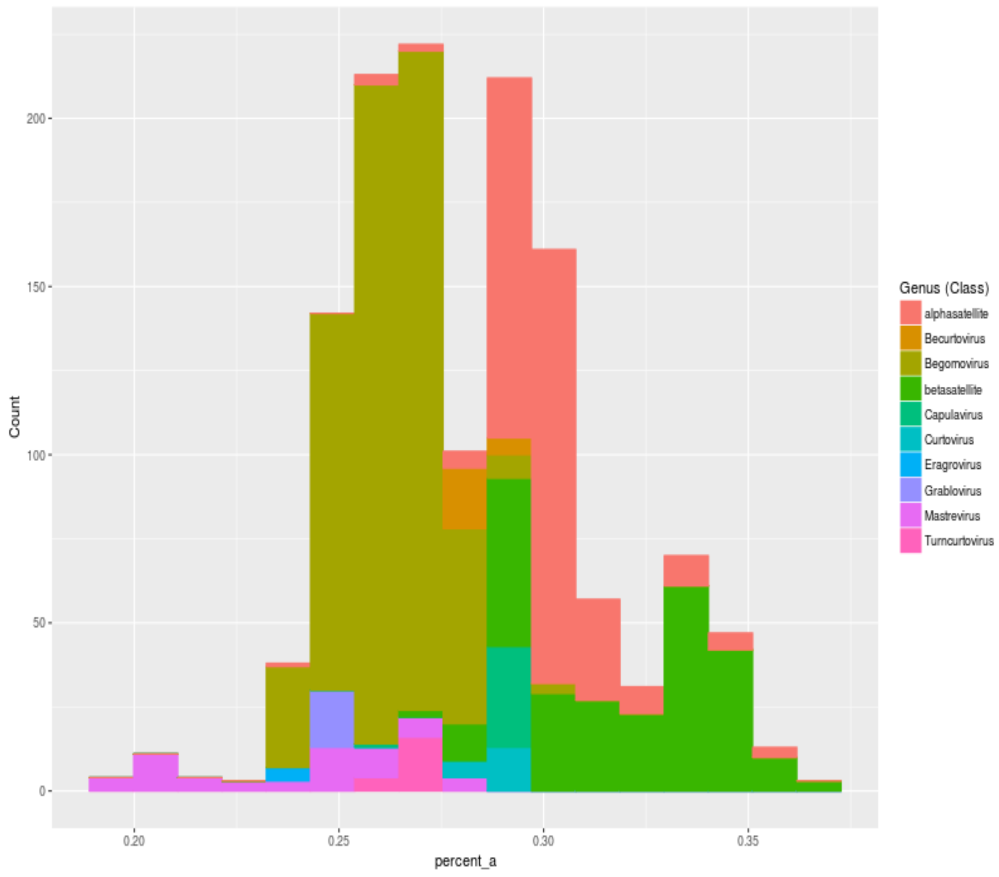

Density

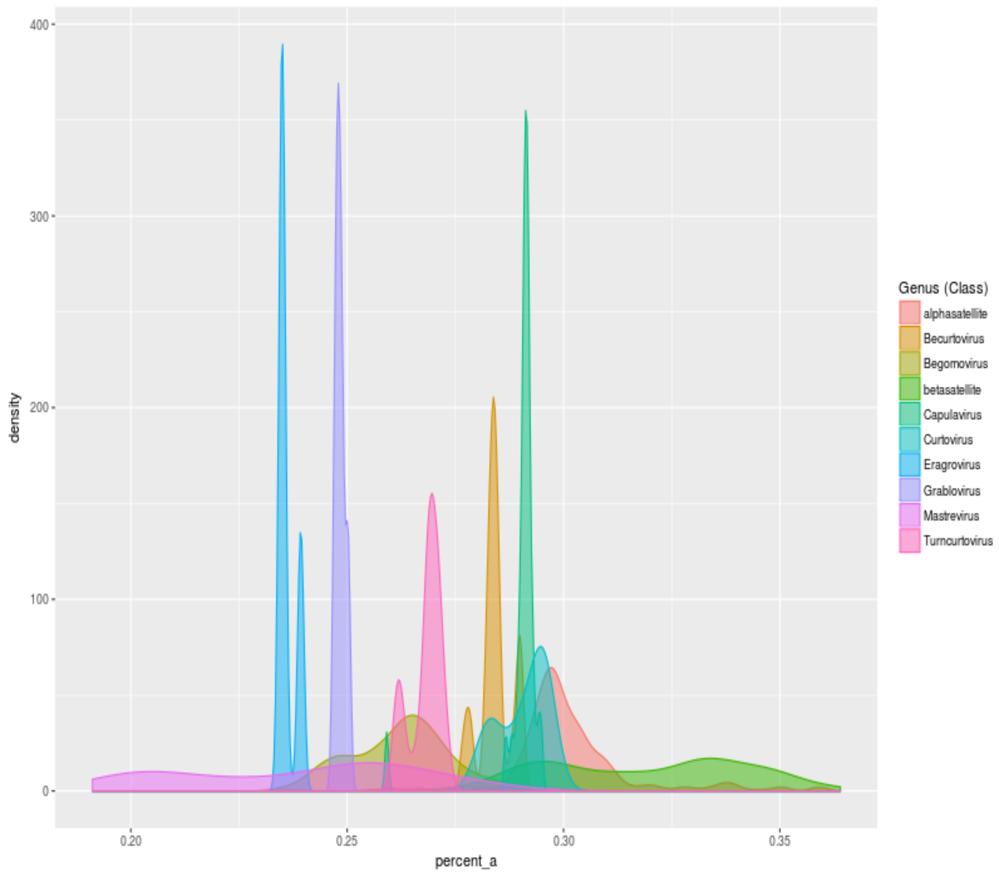

Boxplots

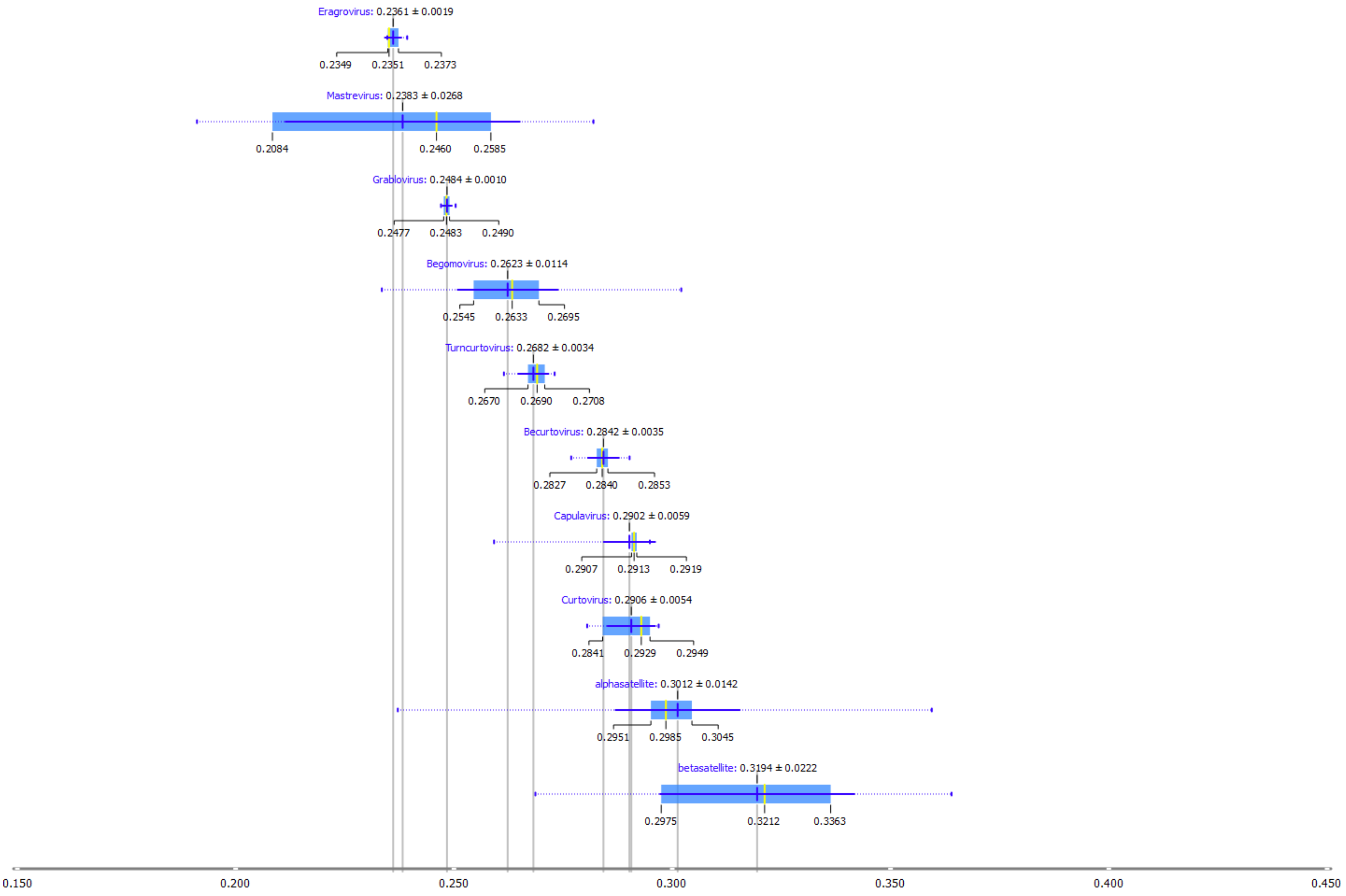

Eragrovirus: 0.2361 ± 0.0019
0.2349 0.2351 0.2373
Mastrevirus: 0.2383 ± 0.0268
0.2084 0.2460 0.2585
Grablovirus: 0.2484 ± 0.0010
0.2477 0.2483 0.2490
Begomovirus: 0.2623 ± 0.0114
0.2545 0.2633 0.2695
Turncurtovirus: 0.2682 ± 0.0034
0.2670 0.2690 0.2708
Becurtovirus: 0.2842 ± 0.0035
0.2827 0.2840 0.2853
Capulavirus: 0.2902 ± 0.0059
0.2907 0.2913 0.2919
Curtovirus: 0.2906 ± 0.0054
0.2841 0.2929 0.2949
alphasatellite: 0.3012 ± 0.0142
0.2951 0.2985 0.3045
betasatellite: 0.3194 ± 0.0222
0.2975 0.3212 0.3363
0.150 0.200 0.250 0.300 0.350 0.400 0.450

Proportion of Adenine in region 1

Histogram

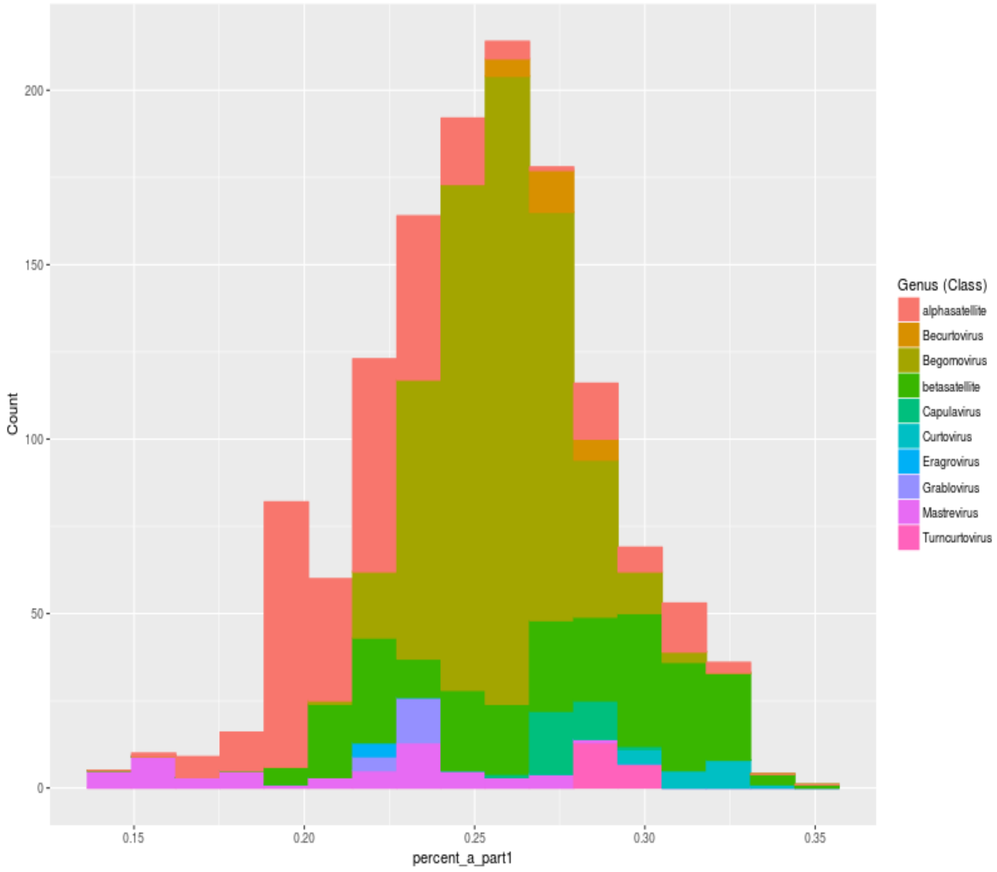

Density

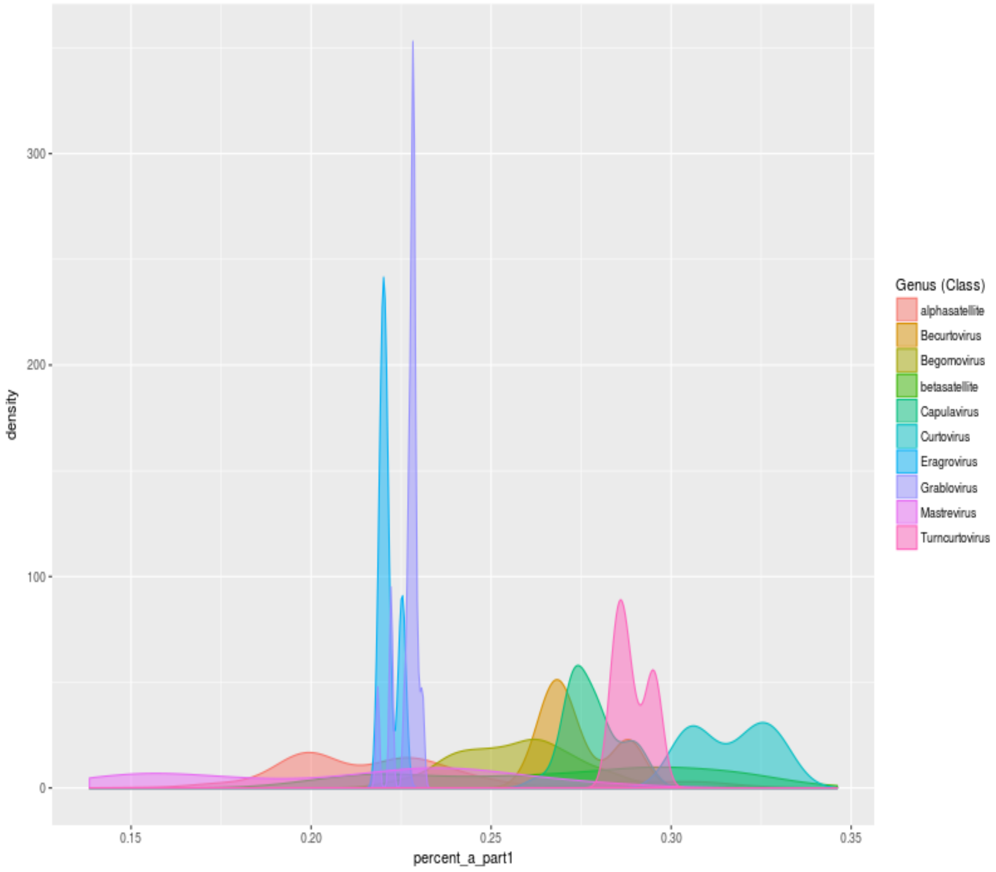

Boxplots

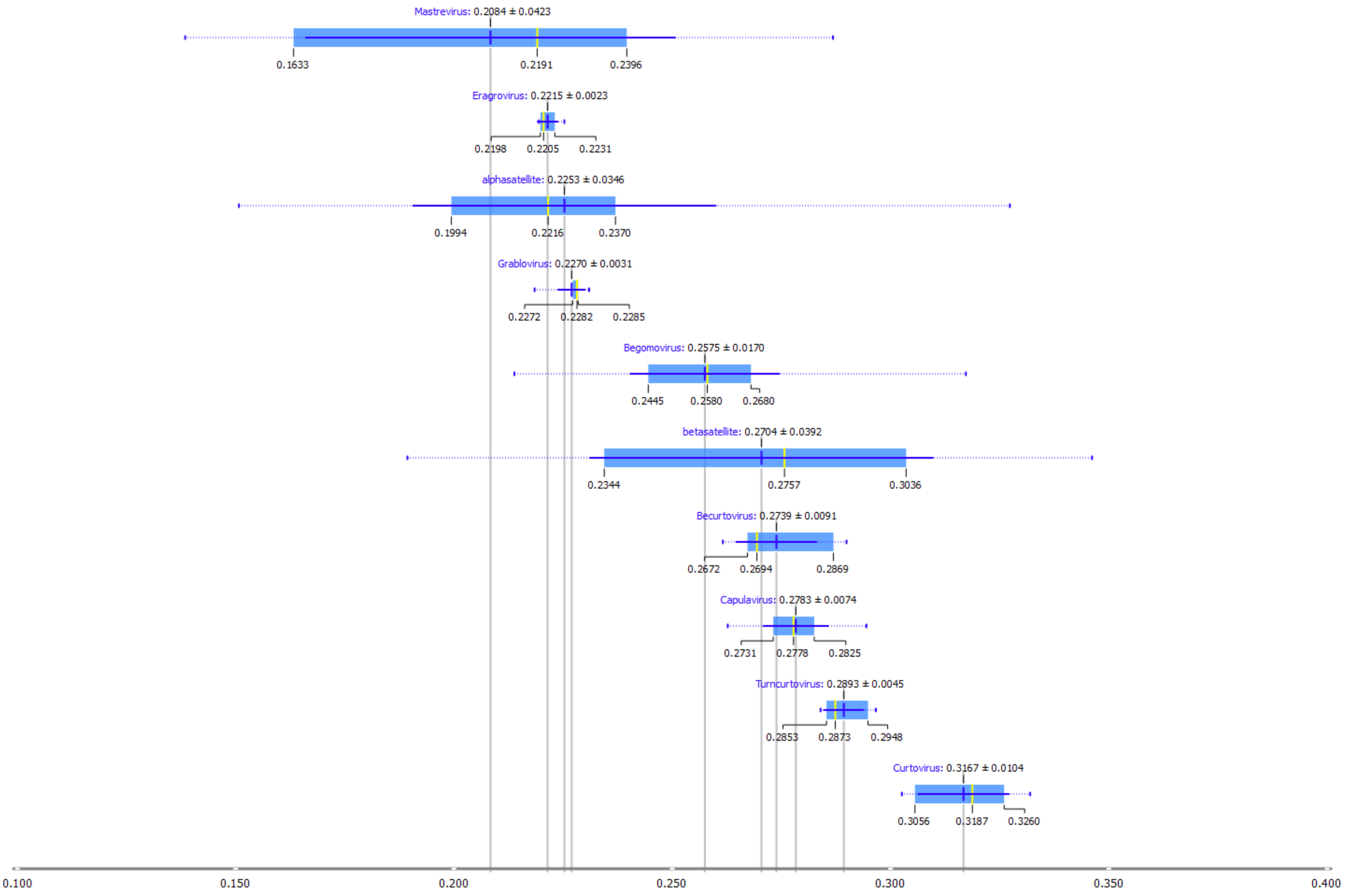
Mastrevirus: 0.2084 ± 0.0423
0.1633
0.2191
0.2396
Eragrovirus: 0.2215 ± 0.0023
0.2198
0.2205
0.2231
alphasatellite: 0.2253 ± 0.0346
0.1994
0.2216
0.2370
Grablovirus: 0.2270 ± 0.0031
0.2272
0.2282
0.2285
Begomovirus: 0.2575 ± 0.0170
0.2445
0.2580
0.2680
betasatellite: 0.2704 ± 0.0392
0.2344
0.2757
0.3036
Becurtovirus: 0.2739 ± 0.0091
0.2672
0.2694
0.2869
Capulavirus: 0.2783 ± 0.0074
0.2731
0.2778
0.2825
Turncurtovirus: 0.2893 ± 0.0045
0.2853
0.2873
0.2948
Curtovirus: 0.3167 ± 0.0104
0.3056
0.3187
0.3260
0.100
0.150
0.200
0.250
0.300
0.350
0.400

Proportion of Adenine in region 2

Histogram

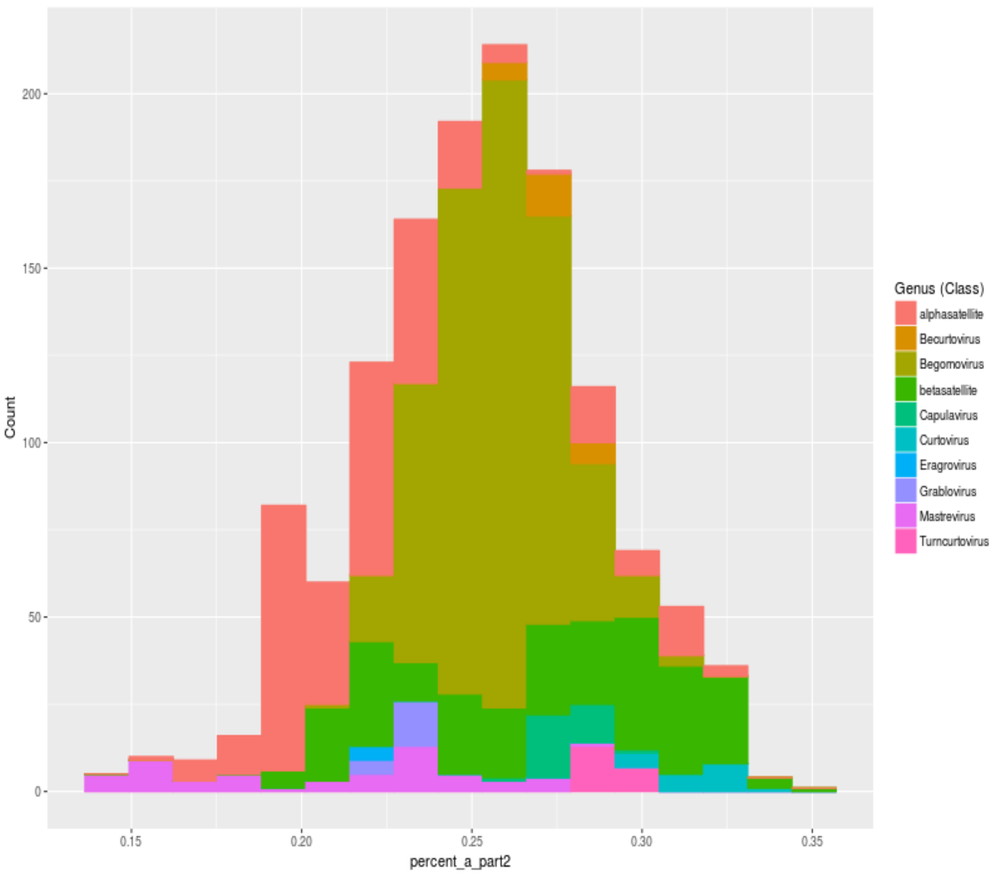

Density

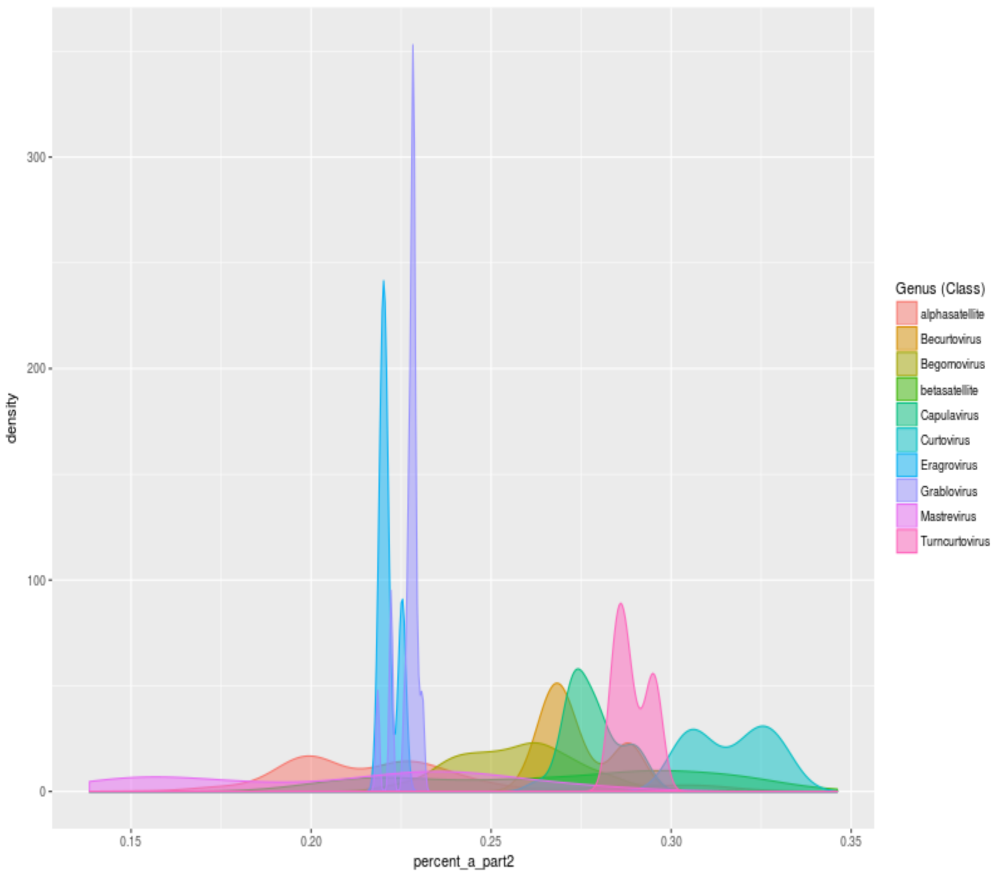

Boxplots

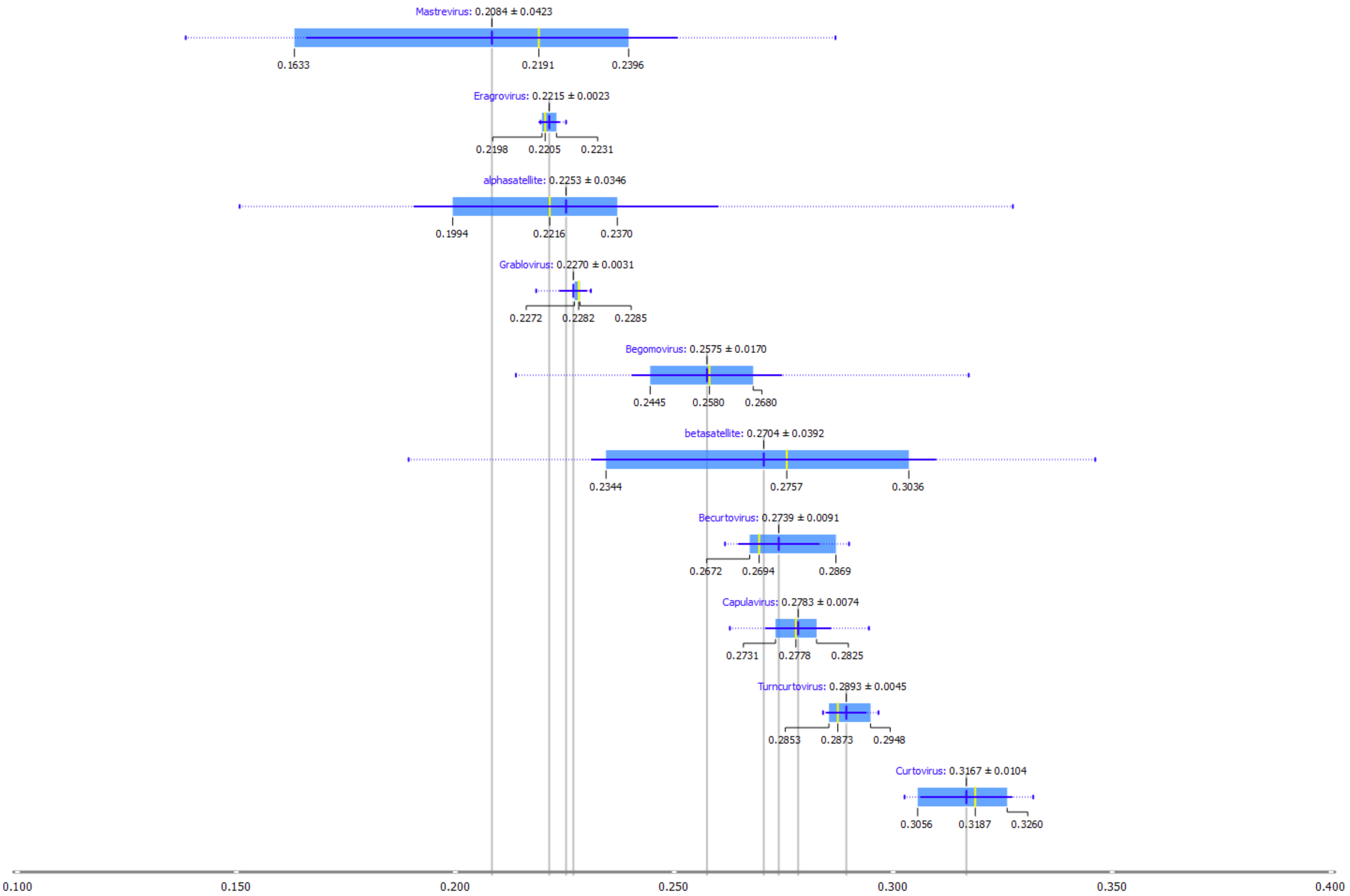

Mastrevirus: 0.2084 ± 0.0423
0.1633
0.2191
0.2396
Eragrovirus: 0.2215 ± 0.0023
0.2198
0.2205
0.2231
alphasatellite: 0.2253 ± 0.0346
0.1994
0.2216
0.2370
Grablovirus: 0.2270 ± 0.0031
0.2272
0.2282
0.2285
Begomovirus: 0.2575 ± 0.0170
0.2445
0.2580
0.2680
betasatellite: 0.2704 ± 0.0392
0.2344
0.2757
0.3036
Becurtovirus: 0.2739 ± 0.0091
0.2672
0.2694
0.2869
Capulavirus: 0.2783 ± 0.0074
0.2731
0.2778
0.2825
Turncurtovirus: 0.2893 ± 0.0045
0.2853
0.2873
0.2948
Curtovirus: 0.3167 ± 0.0104
0.3056
0.3187
0.3260
0.100
0.150
0.200
0.250
0.300
0.350
0.400

Proportion of Adenine in region 3

Histogram

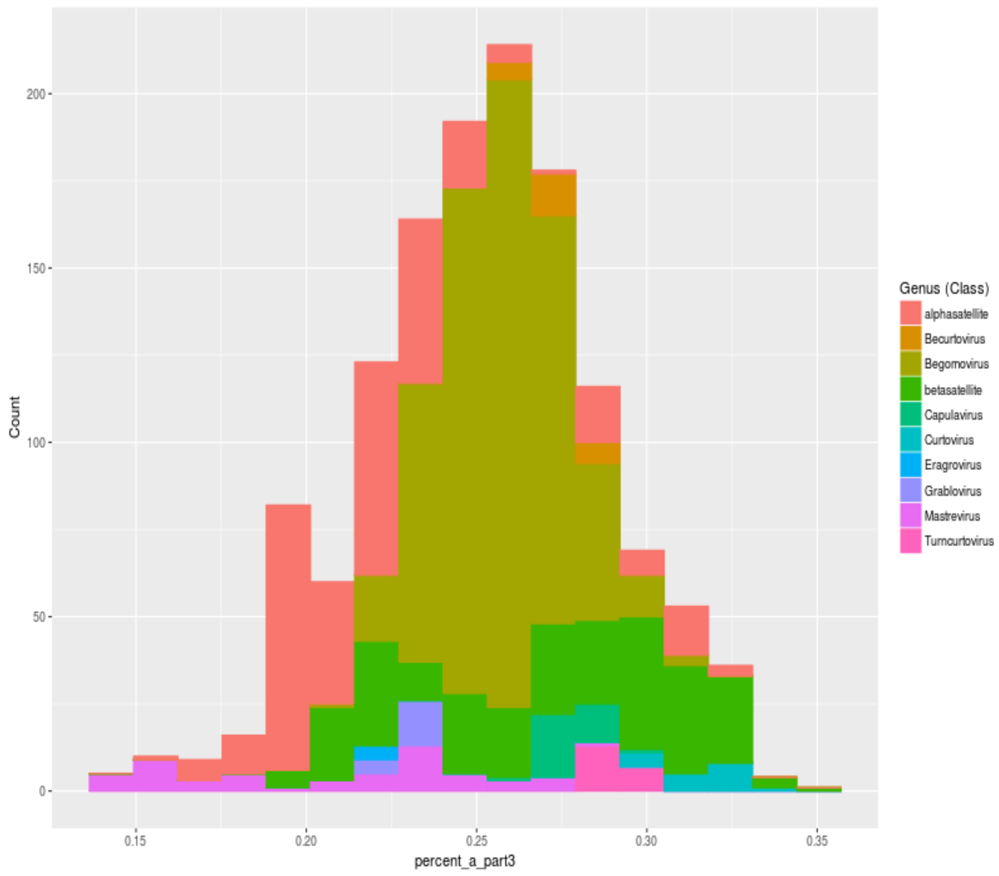

Density

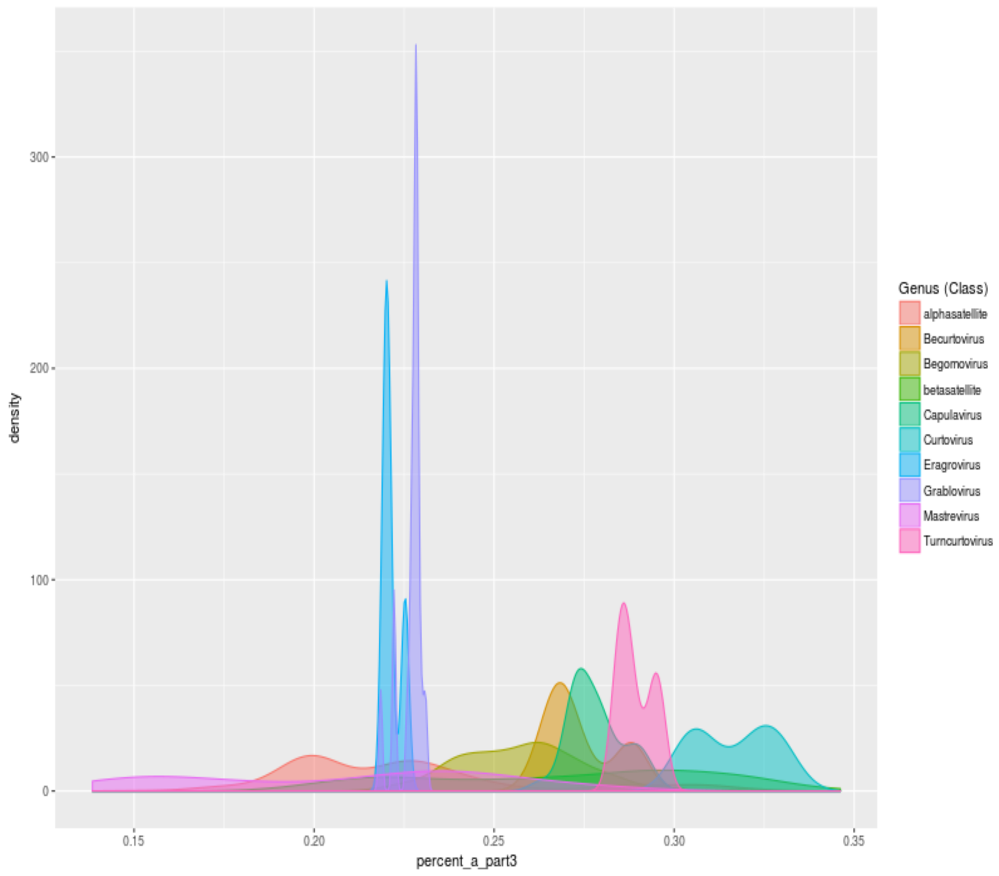

Boxplots

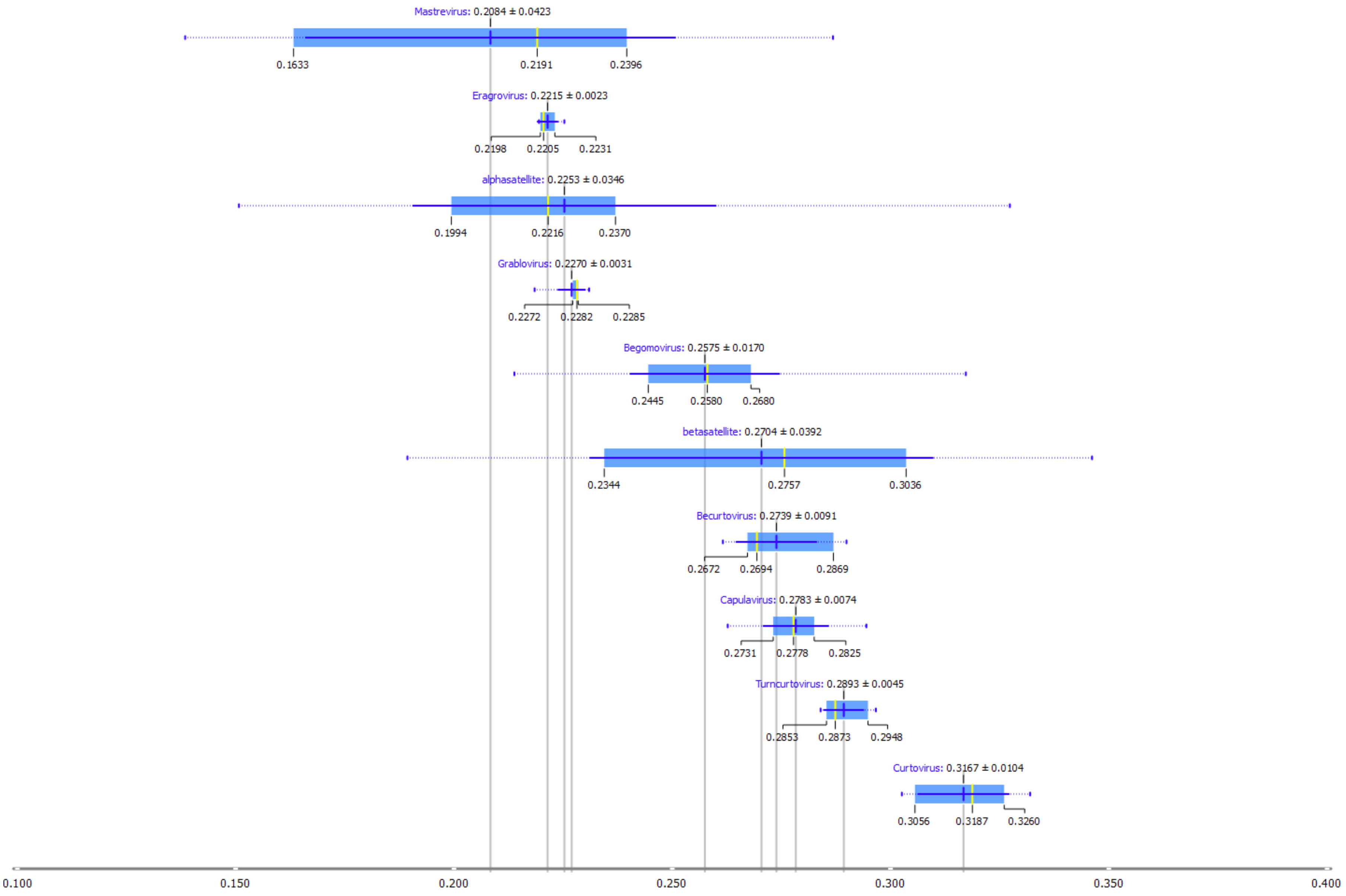

Mastrevirus: 0.2084 ± 0.0423
0.1633
0.2191
0.2396
Eragrovirus: 0.2215 ± 0.0023
0.2198
0.2205
0.2231
alphasatellite: 0.2253 ± 0.0346
0.1994
0.2216
0.2370
Grablovirus: 0.2270 ± 0.0031
0.2272
0.2282
0.2285
Begomovirus: 0.2575 ± 0.0170
0.2445
0.2580
0.2680
betasatellite: 0.2704 ± 0.0392
0.2344
0.2757
0.3036
Becurtovirus: 0.2739 ± 0.0091
0.2672
0.2694
0.2869
Capulavirus: 0.2783 ± 0.0074
0.2731
0.2778
0.2825
Turncurtovirus: 0.2893 ± 0.0045
0.2853
0.2873
0.2948
Curtovirus: 0.3167 ± 0.0104
0.3056
0.3187
0.3260
0.100
0.150
0.200
0.250
0.300
0.350
0.400

Proportion of Adenine in region 4

Histogram

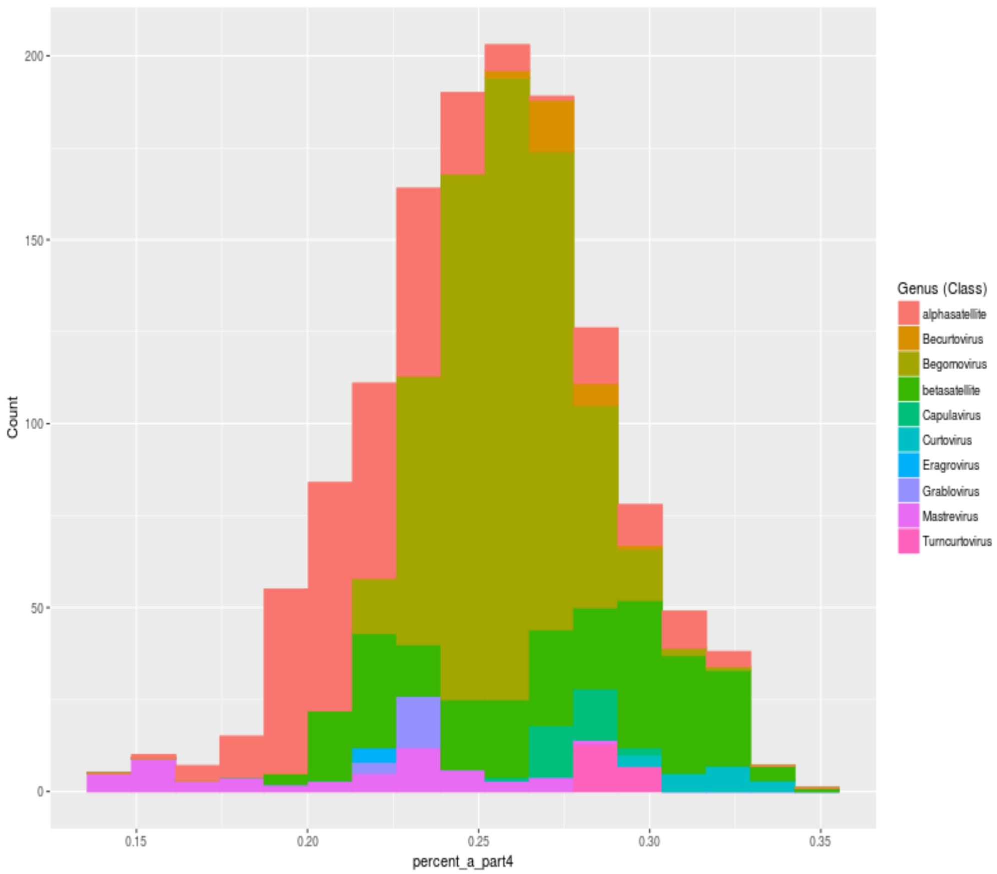

Density

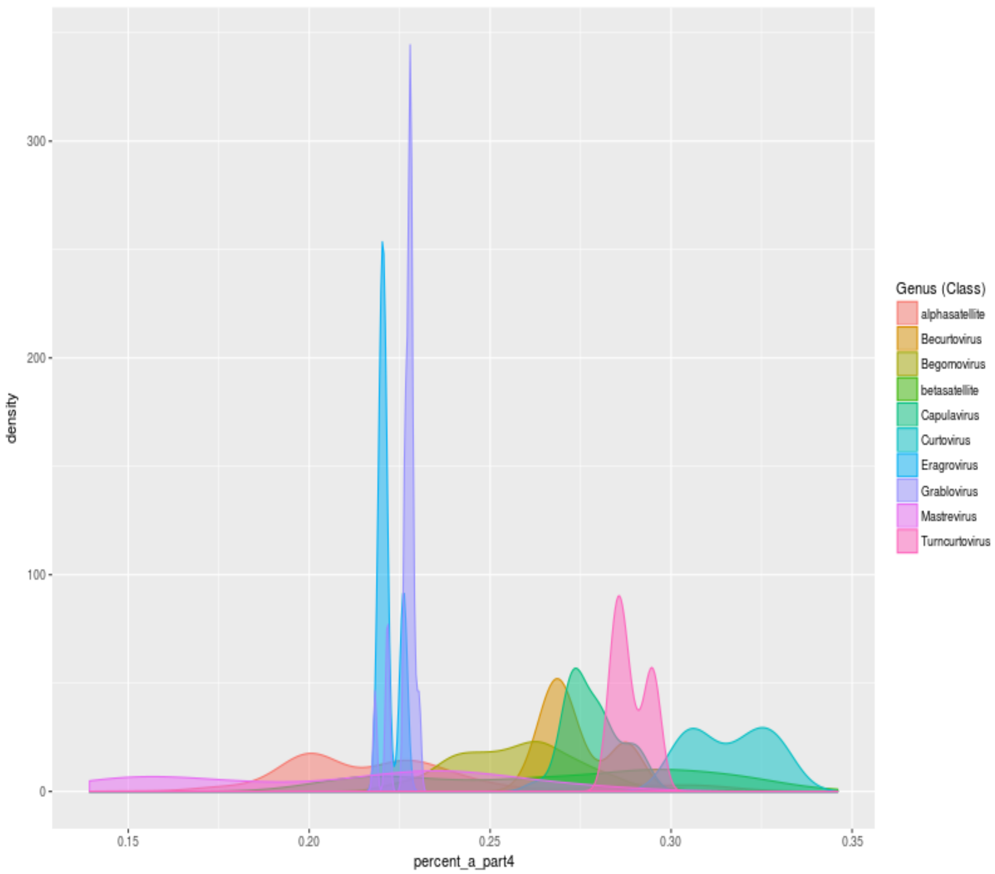

Boxplots

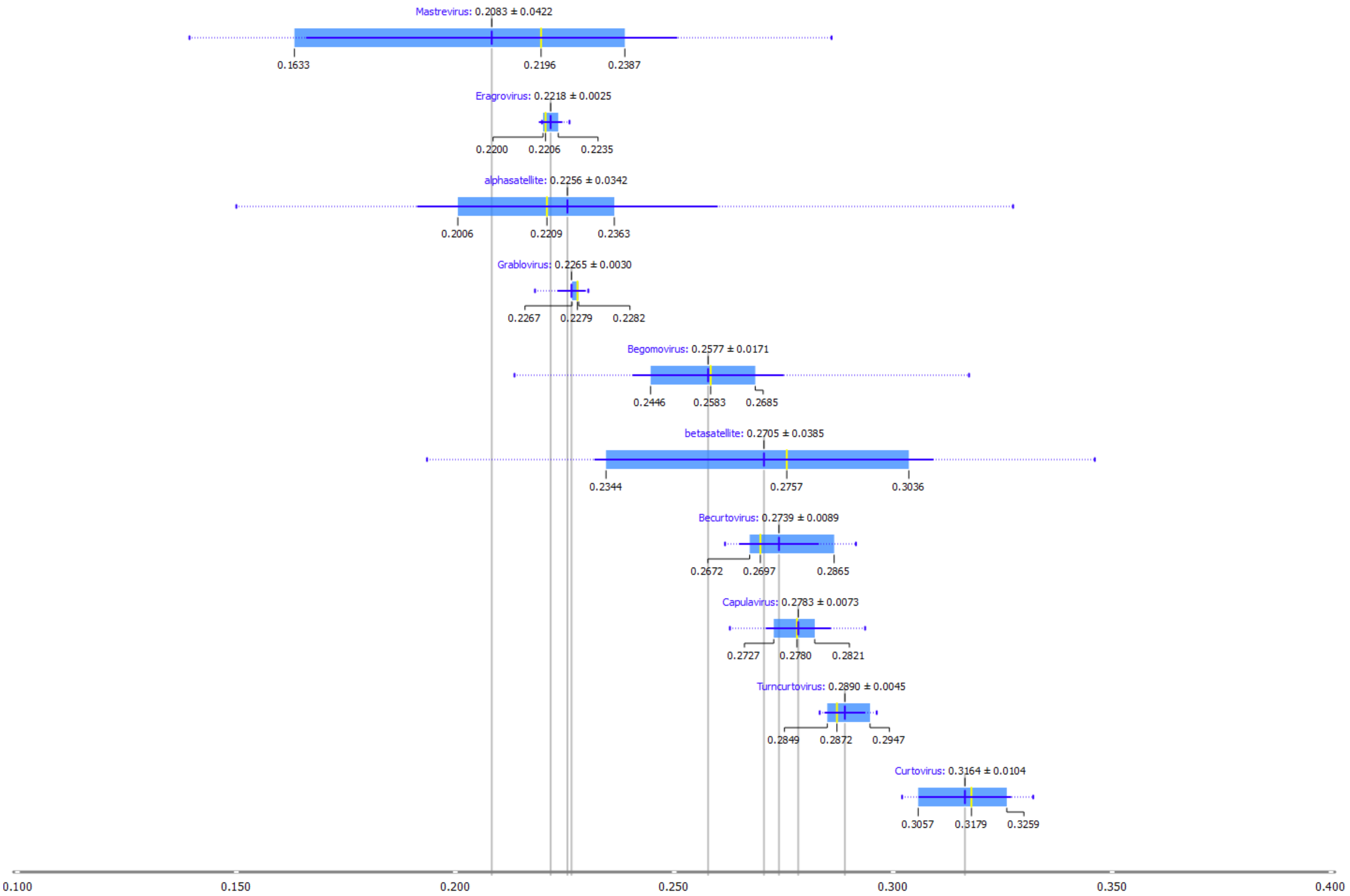

Mastrevirus: 0.2083 ± 0.0422
0.1633
0.2196
0.2387
Eragrovirus: 0.2218 ± 0.0025
0.2200
0.2206
0.2235
alphasatellite: 0.2256 ± 0.0342
0.2006
0.2209
0.2363
Grablovirus: 0.2265 ± 0.0030
0.2267
0.2279
0.2282
Begomovirus: 0.2577 ± 0.0171
0.2446
0.2583
0.2685
betasatellite: 0.2705 ± 0.0385
0.2344
0.2757
0.3036
Becurtovirus: 0.2739 ± 0.0089
0.2672
0.2697
0.2865
Capulavirus: 0.2783 ± 0.0073
0.2727
0.2780
0.2821
Turncurtovirus: 0.2890 ± 0.0045
0.2849
0.2872
0.2947
Curtovirus: 0.3164 ± 0.0104
0.3057
0.3179
0.3259
0.100
0.150
0.200
0.250
0.300
0.350
0.400

Proportion of Thymine in the genome

Histogram

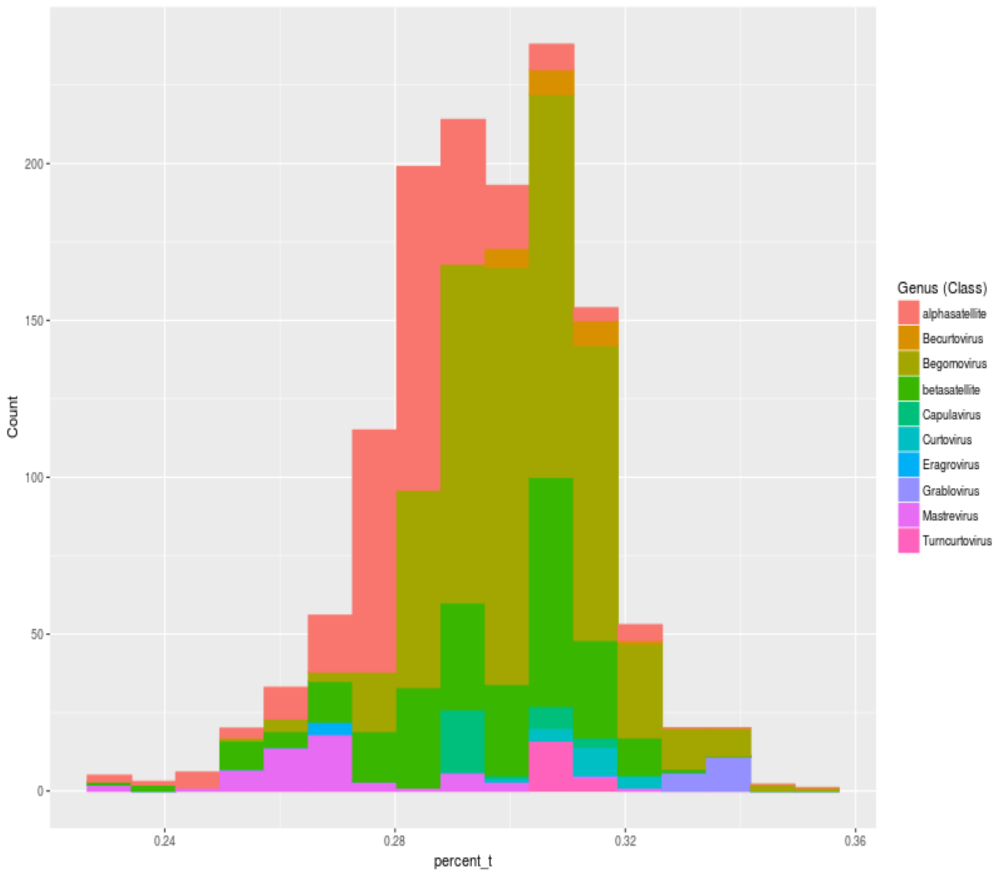

Density

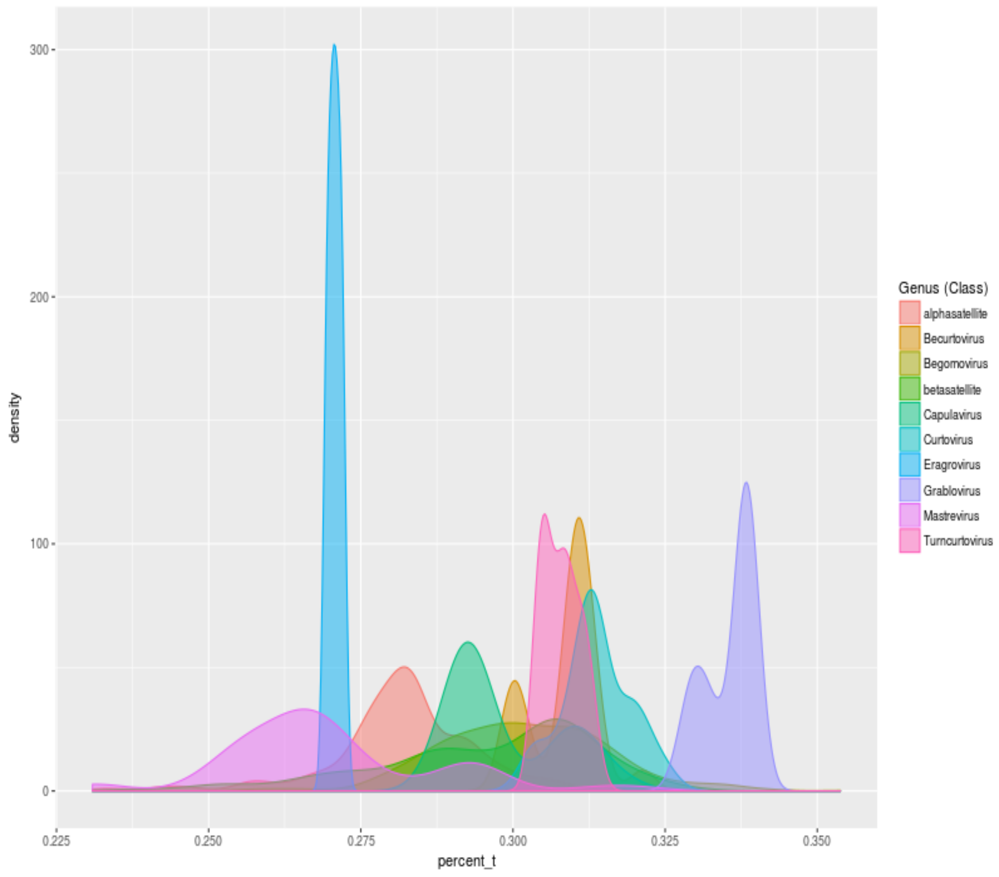

Boxplots

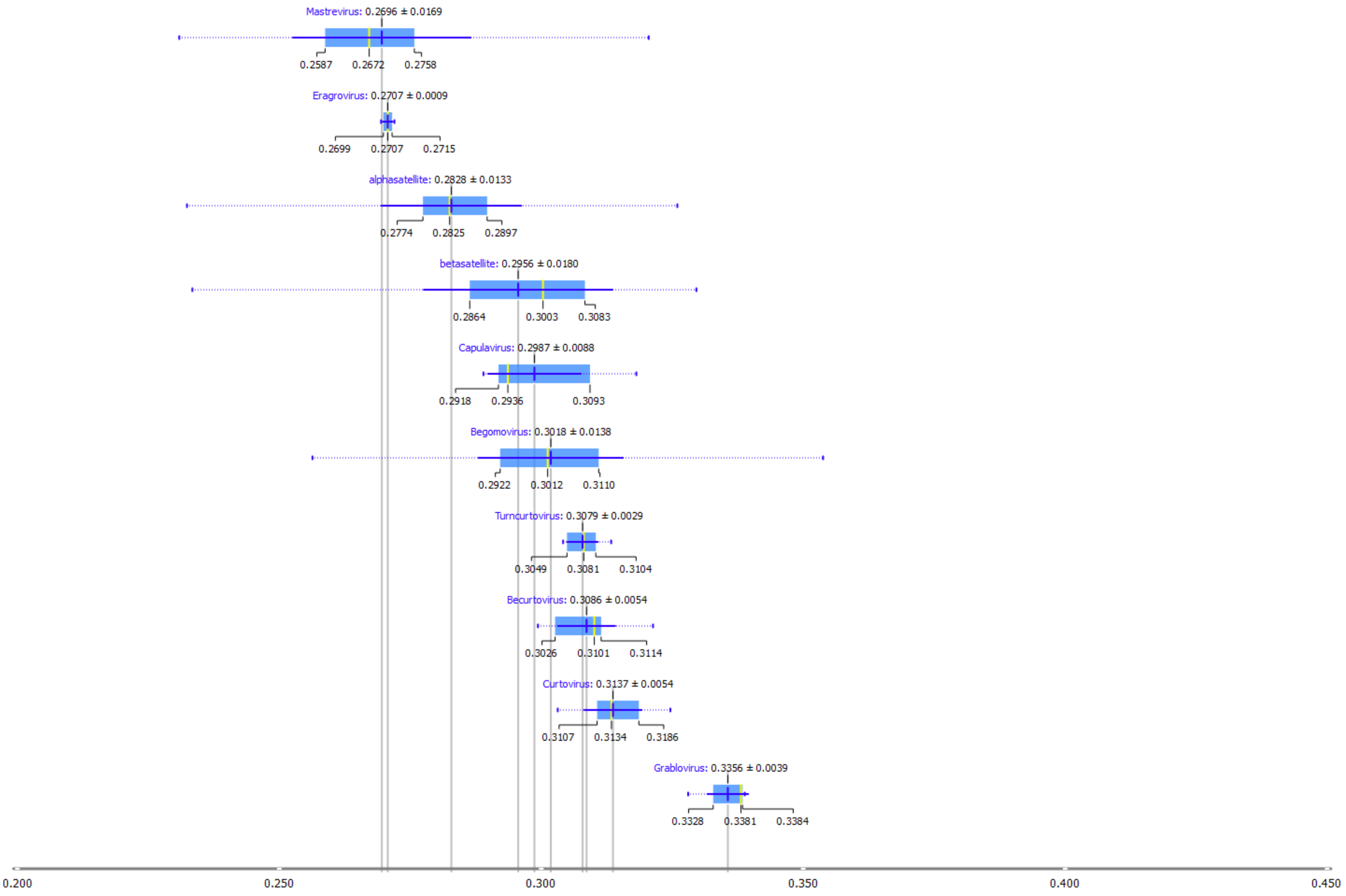

Mastrevirus: 0.2696 ± 0.0169
0.2587 0.2672 0.2758
Eragrovirus: 0.2707 ± 0.0009
0.2699 0.2707 0.2715
alphasatellite: 0.2828 ± 0.0133
0.2774 0.2825 0.2897
betasatellite: 0.2956 ± 0.0180
0.2864 0.3003 0.3083
Capulavirus: 0.2987 ± 0.0088
0.2918 0.2936 0.3093
Begomovirus: 0.3018 ± 0.0138
0.2922 0.3012 0.3110
Turncurtovirus: 0.3079 ± 0.0029
0.3049 0.3081 0.3104
Becurtovirus: 0.3086 ± 0.0054
0.3026 0.3101 0.3114
Curtovirus: 0.3137 ± 0.0054
0.3107 0.3134 0.3186
Grablovirus: 0.3356 ± 0.0039
0.3328 0.3381 0.3384
0.200 0.250 0.300 0.350 0.400 0.450

Proportion of Thymine in region 1

Histogram

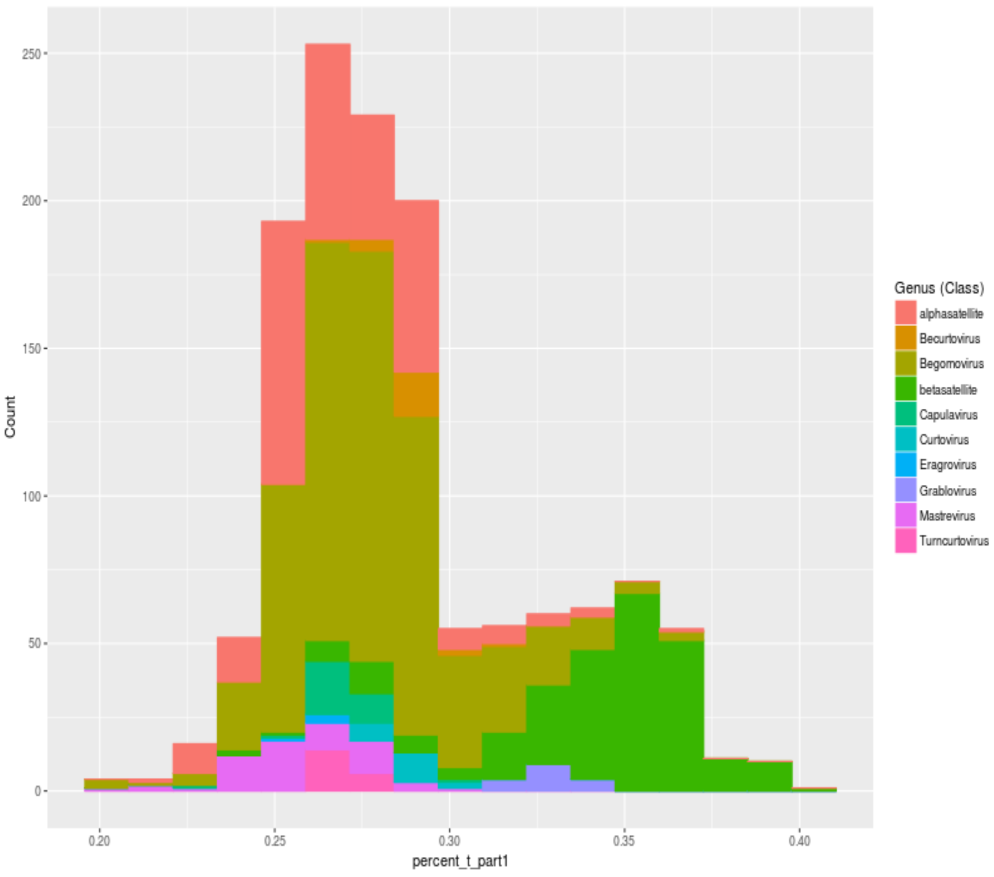

Density

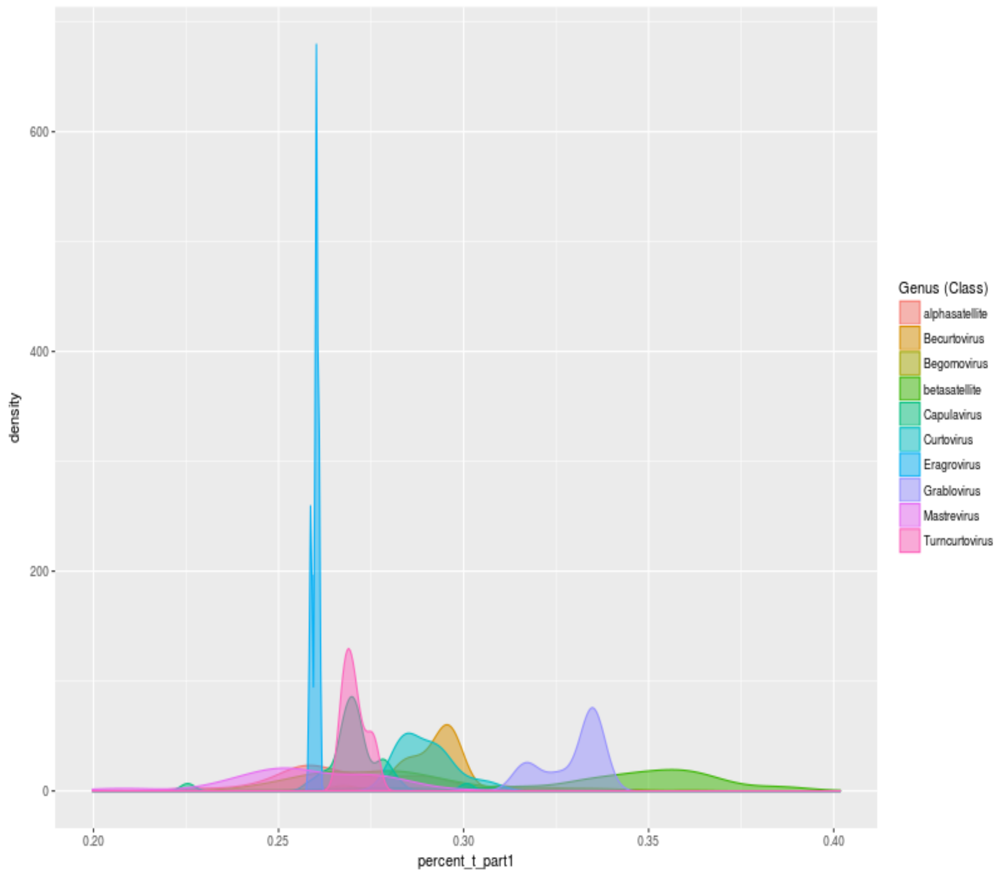

Boxplots

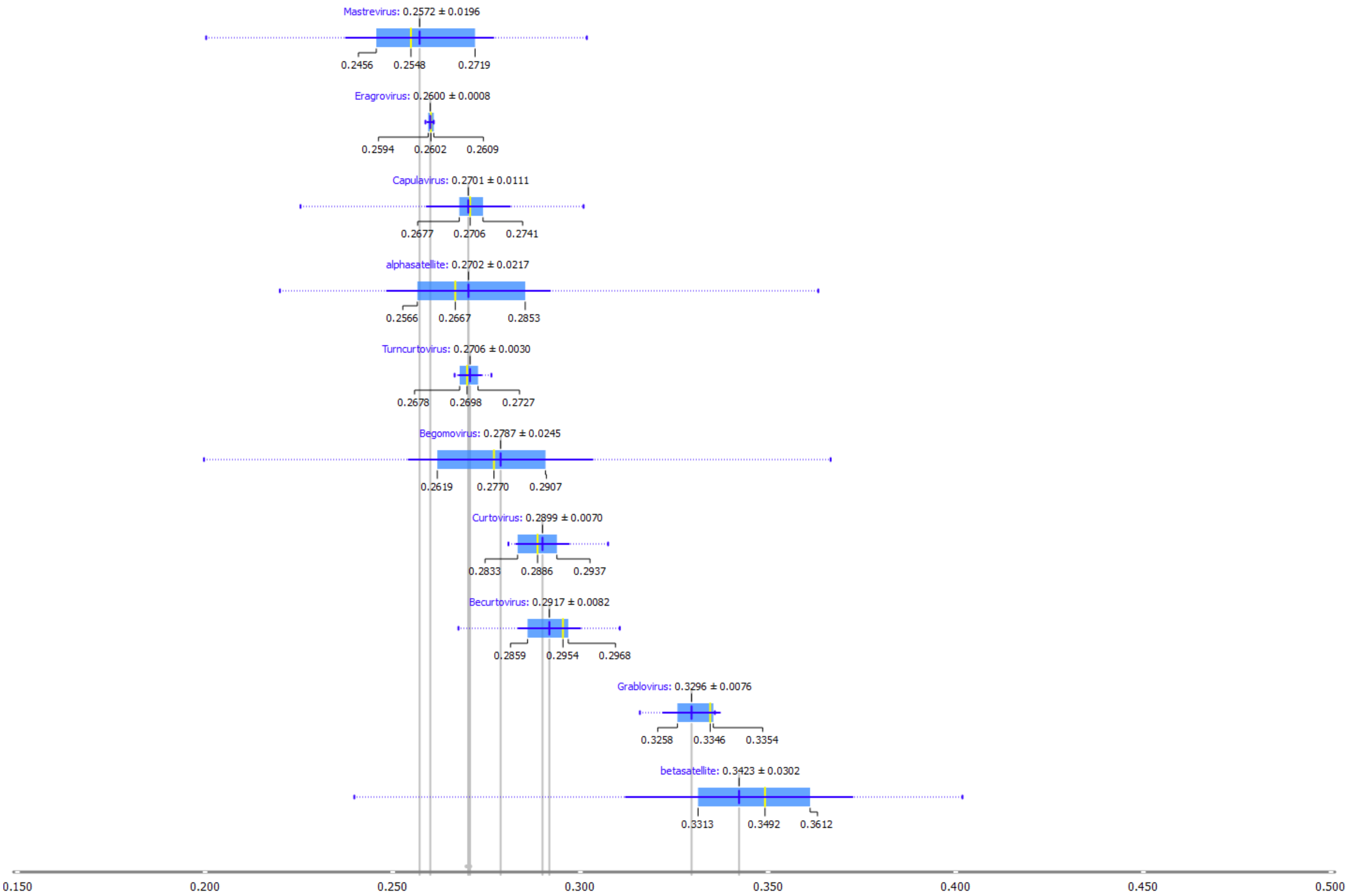

Mastrevirus: 0.2572 ± 0.0196
0.2456 0.2548 0.2719
Eragrovirus: 0.2600 ± 0.0008
0.2594 0.2602 0.2609
Capulavirus: 0.2701 ± 0.0111
0.2677 0.2706 0.2741
alphasatellite: 0.2702 ± 0.0217
0.2566 0.2667 0.2853
Turncurtovirus: 0.2706 ± 0.0030
0.2678 0.2698 0.2727
Begomovirus: 0.2787 ± 0.0245
0.2619 0.2770 0.2907
Curtovirus: 0.2899 ± 0.0070
0.2833 0.2886 0.2937
Becurtovirus: 0.2917 ± 0.0082
0.2859 0.2954 0.2968
Grablovirus: 0.3296 ± 0.0076
0.3258 0.3346 0.3354
betasatellite: 0.3423 ± 0.0302
0.3313 0.3492 0.3612
0.150 0.200 0.250 0.300 0.350 0.400 0.450 0.500

Proportion of Thymine in region 2

Histogram

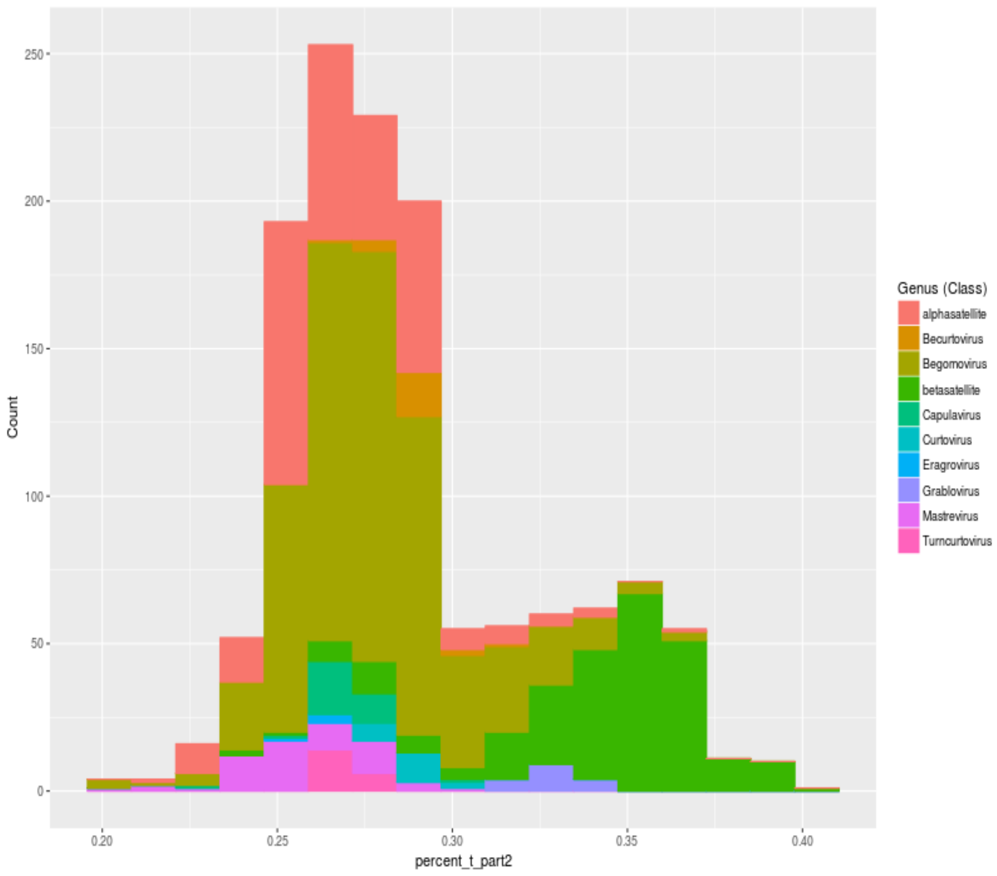

Density

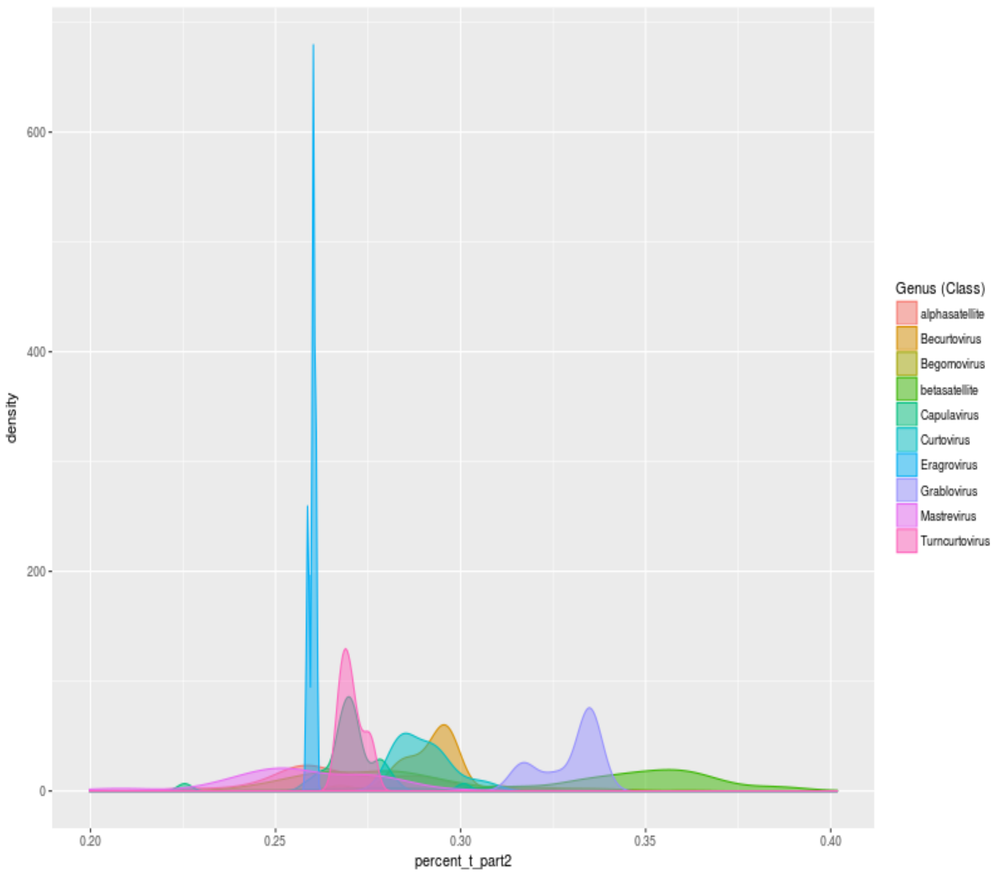

Boxplots

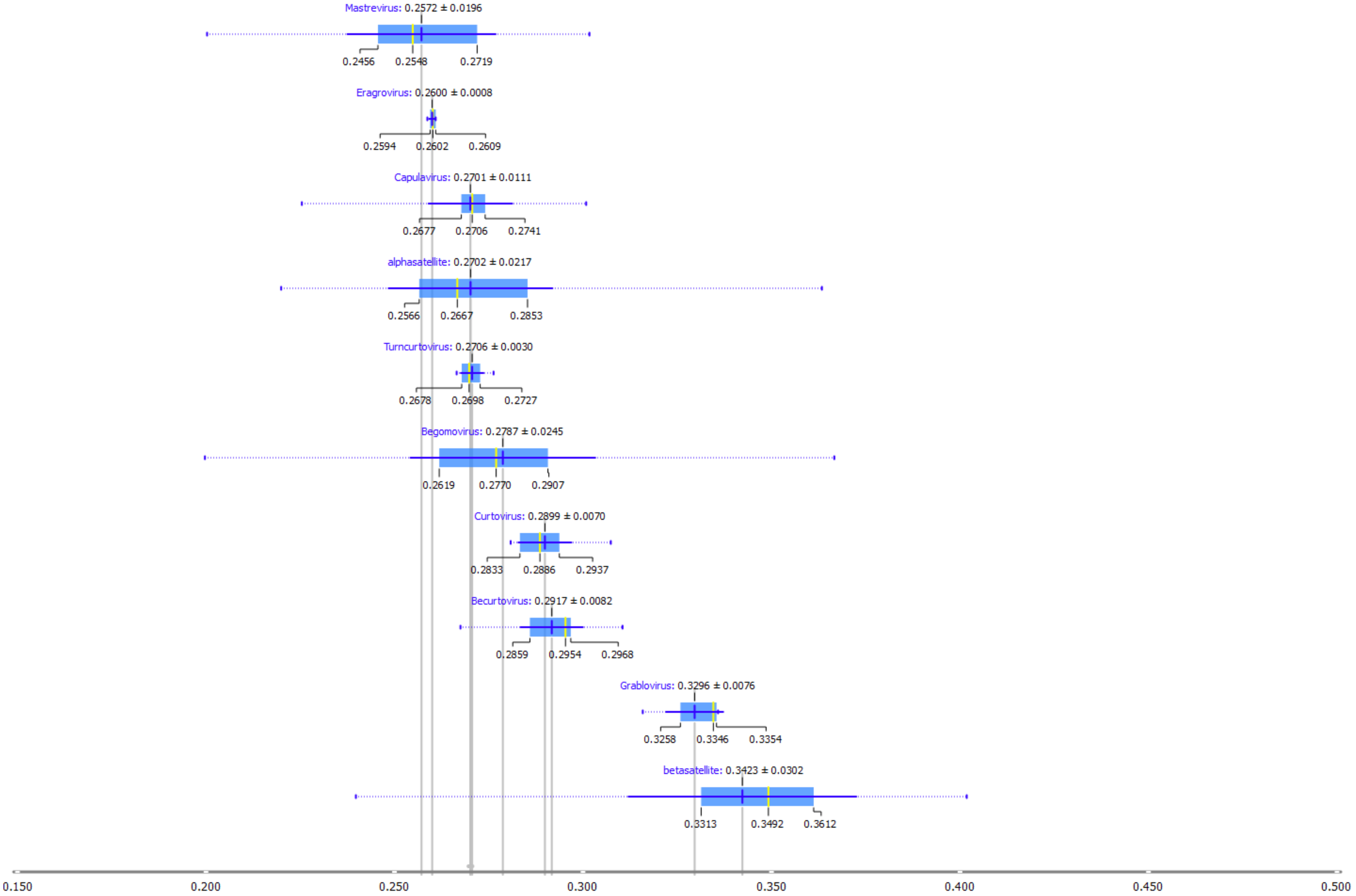

Mastrevirus: 0.2572 ± 0.0196
0.2456 0.2548 0.2719
Eragrovirus: 0.2600 ± 0.0008
0.2594 0.2602 0.2609
Capulavirus: 0.2701 ± 0.0111
0.2677 0.2706 0.2741
alphasatellite: 0.2702 ± 0.0217
0.2566 0.2667 0.2853
Turncurtovirus: 0.2706 ± 0.0030
0.2678 0.2698 0.2727
Begomovirus: 0.2787 ± 0.0245
0.2619 0.2770 0.2907
Curtovirus: 0.2899 ± 0.0070
0.2833 0.2886 0.2937
Becurtovirus: 0.2917 ± 0.0082
0.2859 0.2954 0.2968
Grablovirus: 0.3296 ± 0.0076
0.3258 0.3346 0.3354
betasatellite: 0.3423 ± 0.0302
0.3313 0.3492 0.3612
0.150 0.200 0.250 0.300 0.350 0.400 0.450 0.500

Proportion of Thymine in region 3

Histogram

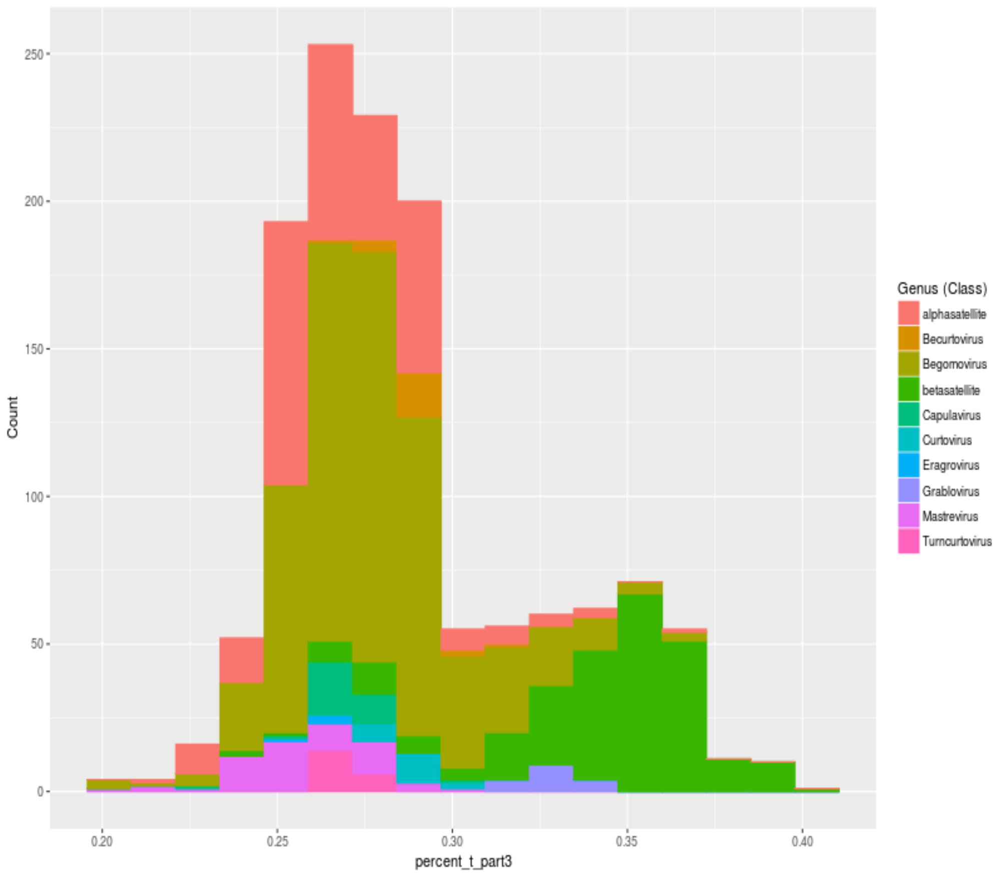

Density

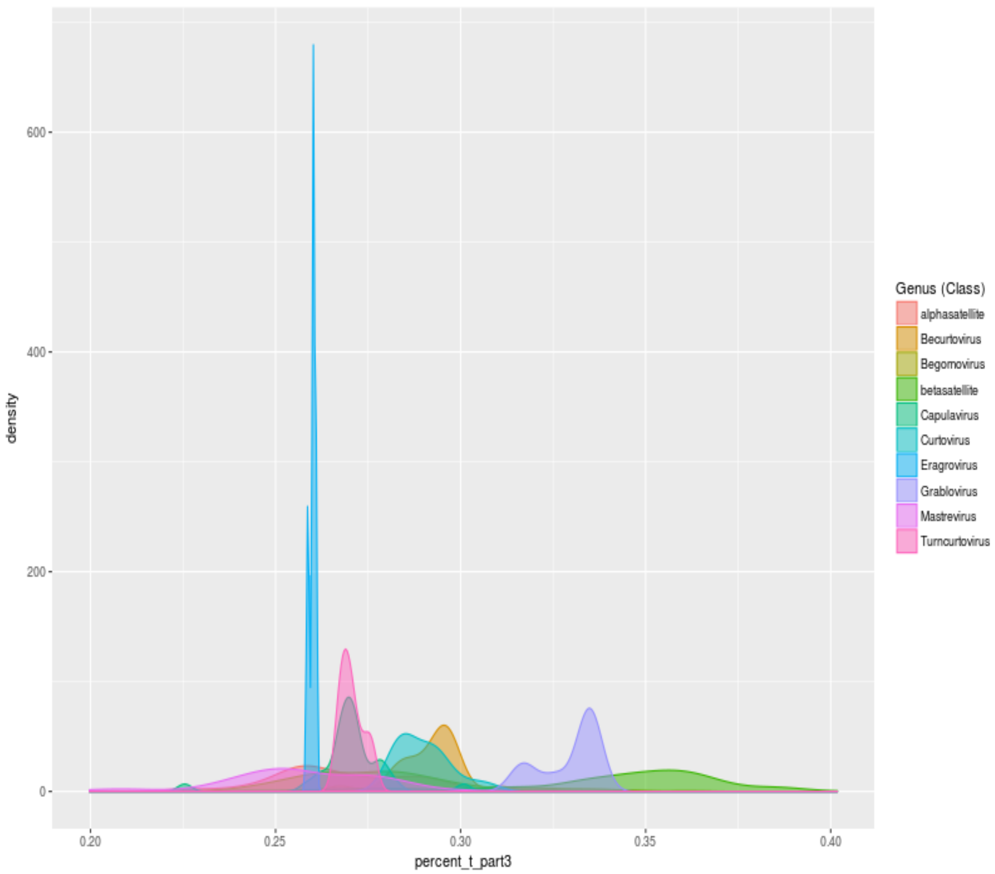

Boxplots

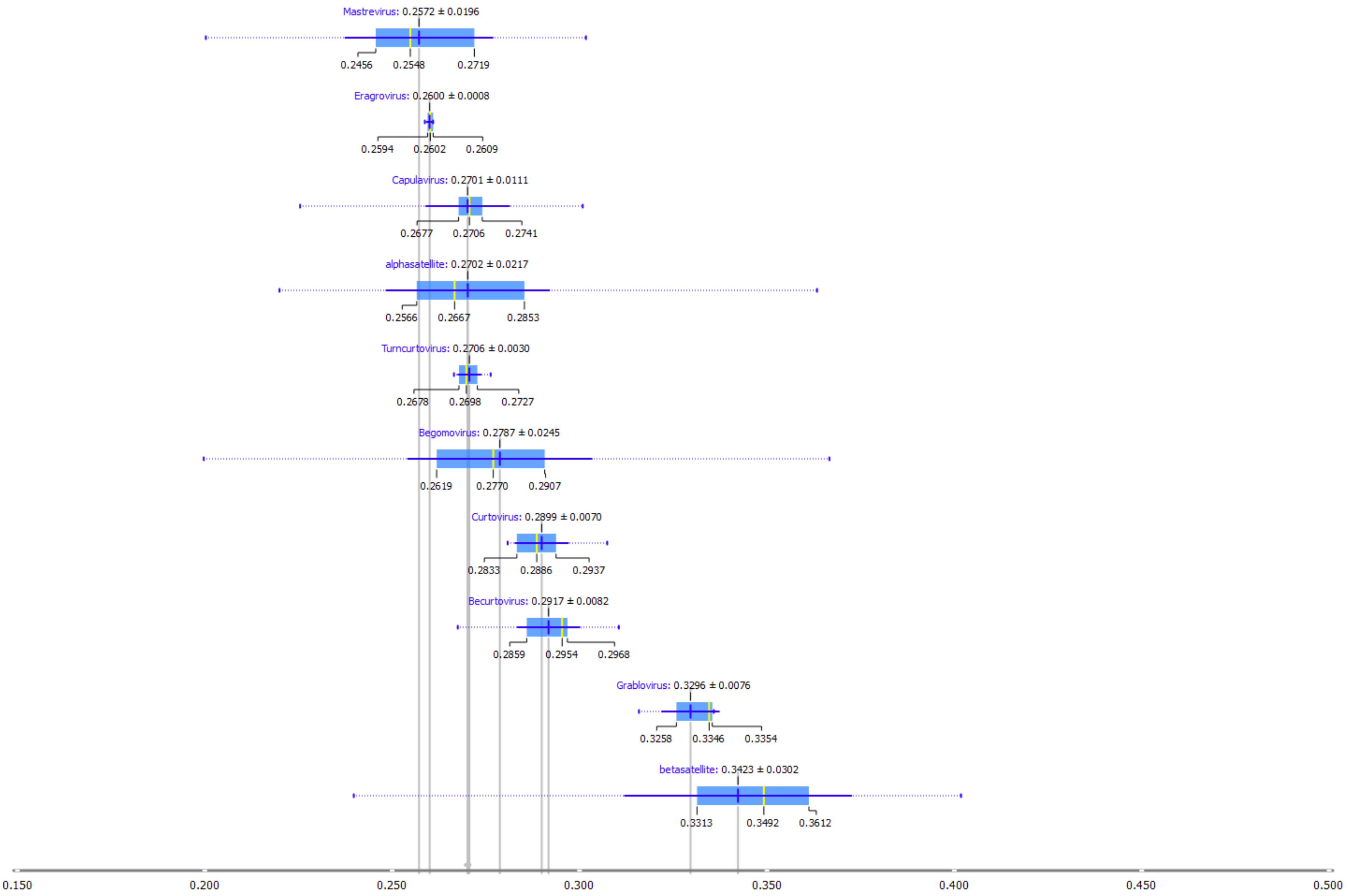

Mastrevirus: 0.2572 ± 0.0196
0.2456
0.2548
0.2719
Eragrovirus: 0.2600 ± 0.0008
0.2594
0.2602
0.2609
Capulavirus: 0.2701 ± 0.0111
0.2677
0.2706
0.2741
alphasatellite: 0.2702 ± 0.0217
0.2566
0.2667
0.2853
Turncurtovirus: 0.2706 ± 0.0030
0.2678
0.2698
0.2727
Begomovirus: 0.2787 ± 0.0245
0.2619
0.2770
0.2907
Curtovirus: 0.2899 ± 0.0070
0.2833
0.2886
0.2937
Becurtovirus: 0.2917 ± 0.0082
0.2859
0.2954
0.2968
Grablovirus: 0.3296 ± 0.0076
0.3258
0.3346
0.3354
betasatellite: 0.3423 ± 0.0302
0.3313
0.3492
0.3612
0.150
0.200
0.250
0.300
0.350
0.400
0.450
0.500

Proportion of Thymine in region 4

Histogram

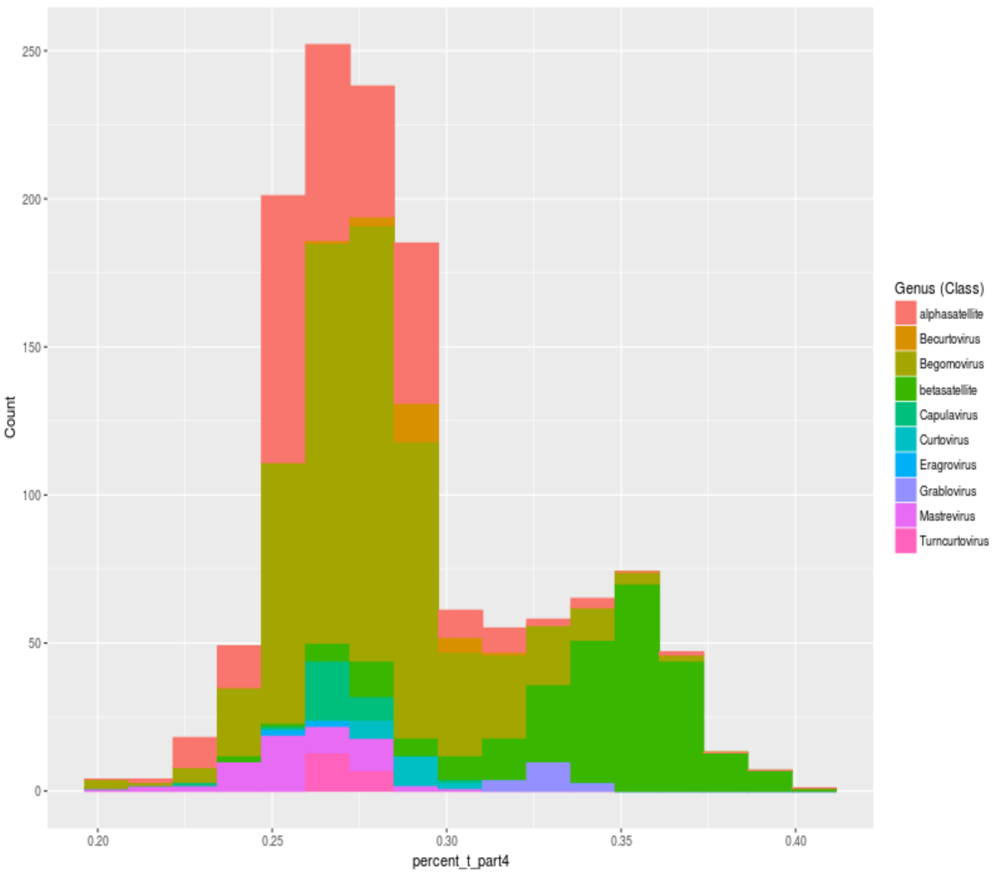

Density

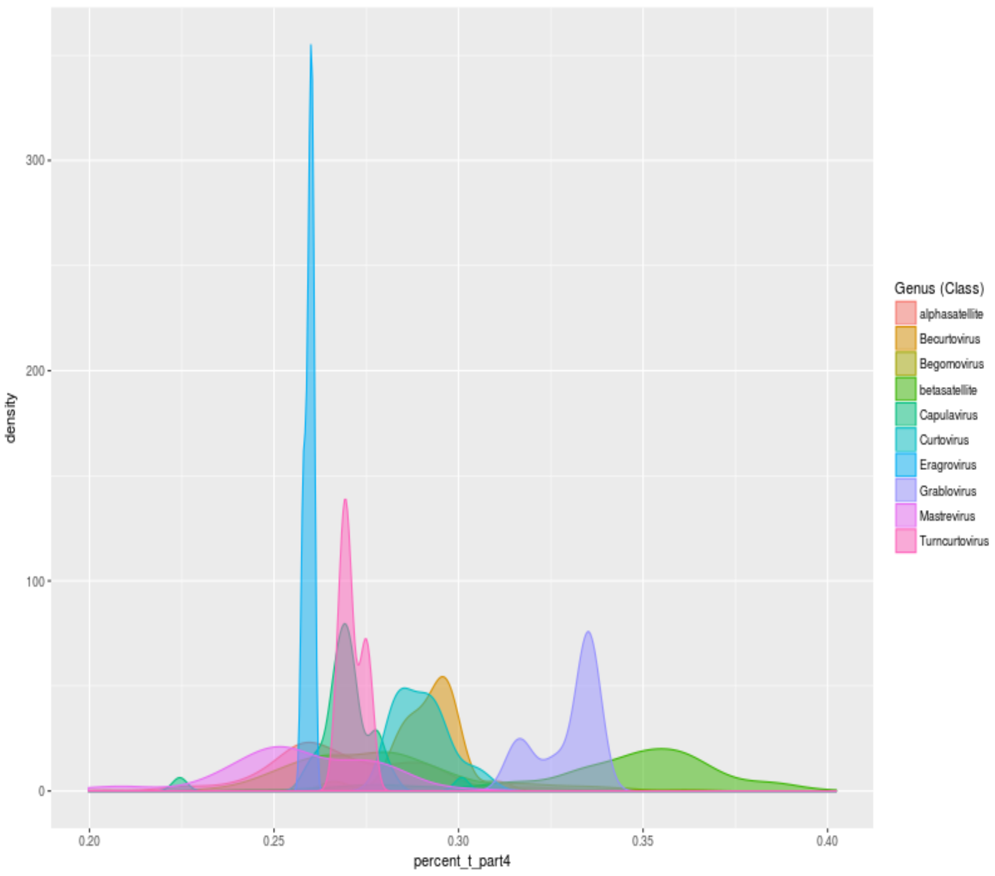

Boxplots

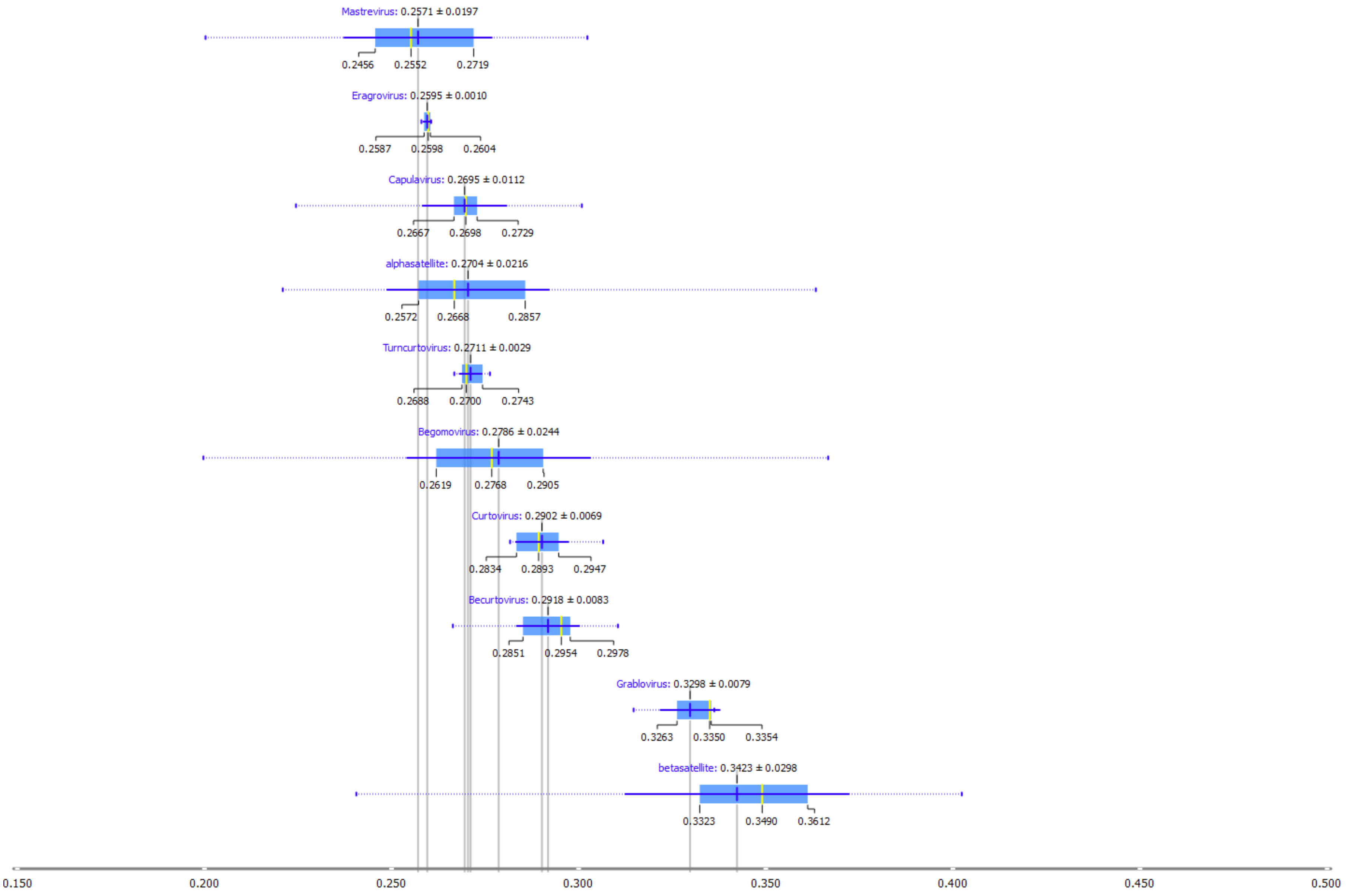

Mastrevirus: 0.2571 ± 0.0197
0.2456
0.2552
0.2719
Eragrovirus: 0.2595 ± 0.0010
0.2587
0.2598
0.2604
Capulavirus: 0.2695 ± 0.0112
0.2667
0.2698
0.2729
alphasatellite: 0.2704 ± 0.0216
0.2572
0.2668
0.2857
Turncurtovirus: 0.2711 ± 0.0029
0.2688
0.2700
0.2743
Begomovirus: 0.2786 ± 0.0244
0.2619
0.2768
0.2905
Curtovirus: 0.2902 ± 0.0069
0.2834
0.2893
0.2947
Becurtovirus: 0.2918 ± 0.0083
0.2851
0.2954
0.2978
Grablovirus: 0.3298 ± 0.0079
0.3263
0.3350
0.3354
betasatellite: 0.3423 ± 0.0298
0.3323
0.3490
0.3612
0.150
0.200
0.250
0.300
0.350
0.400
0.450
0.500

Proportion of Cytosine in the genome

Histogram

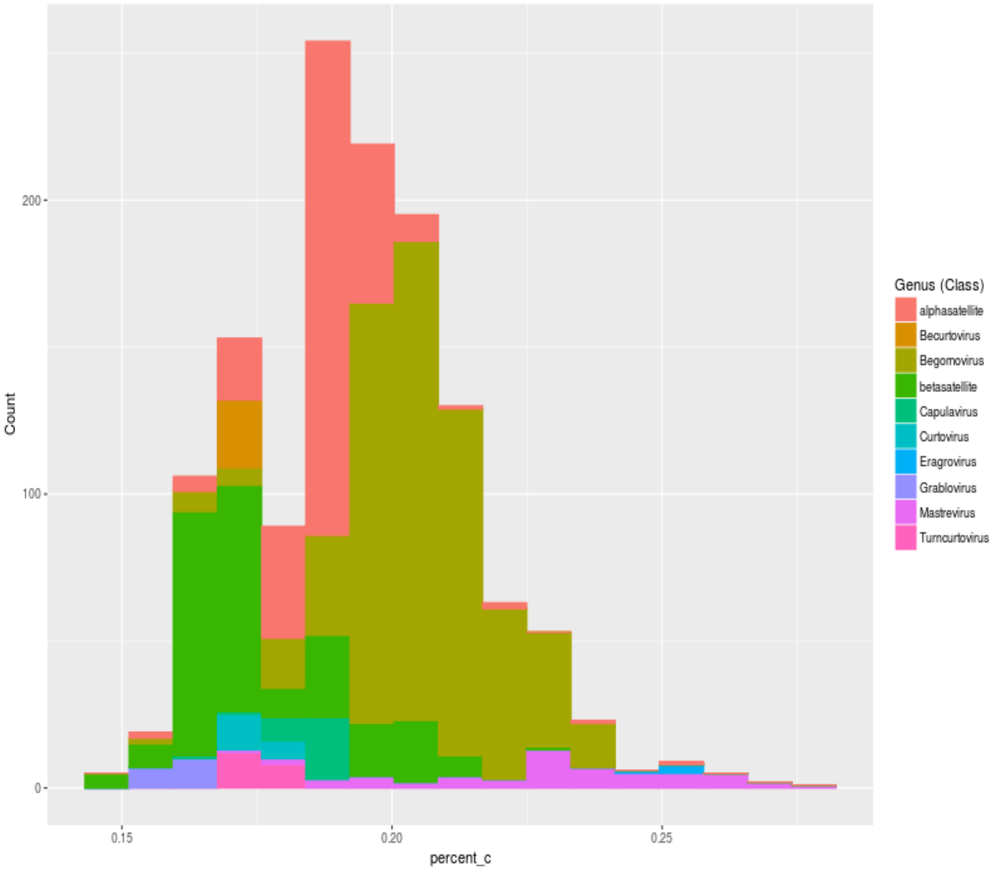

Density

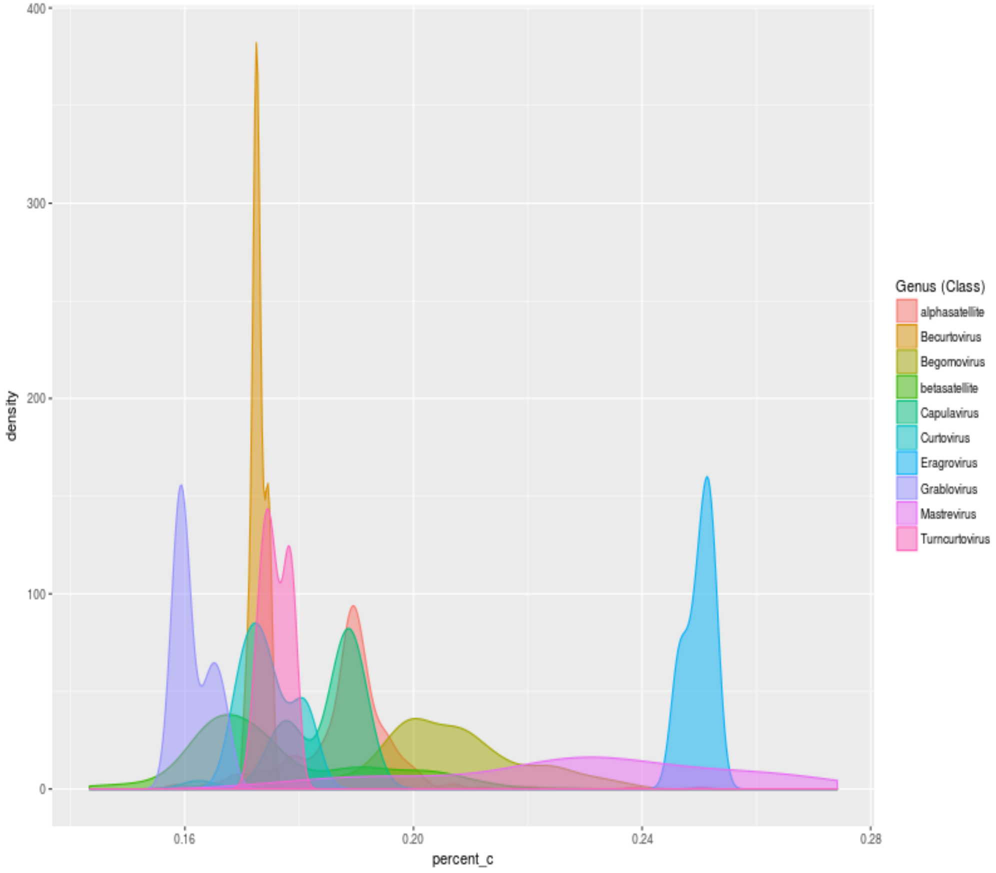

Boxplots

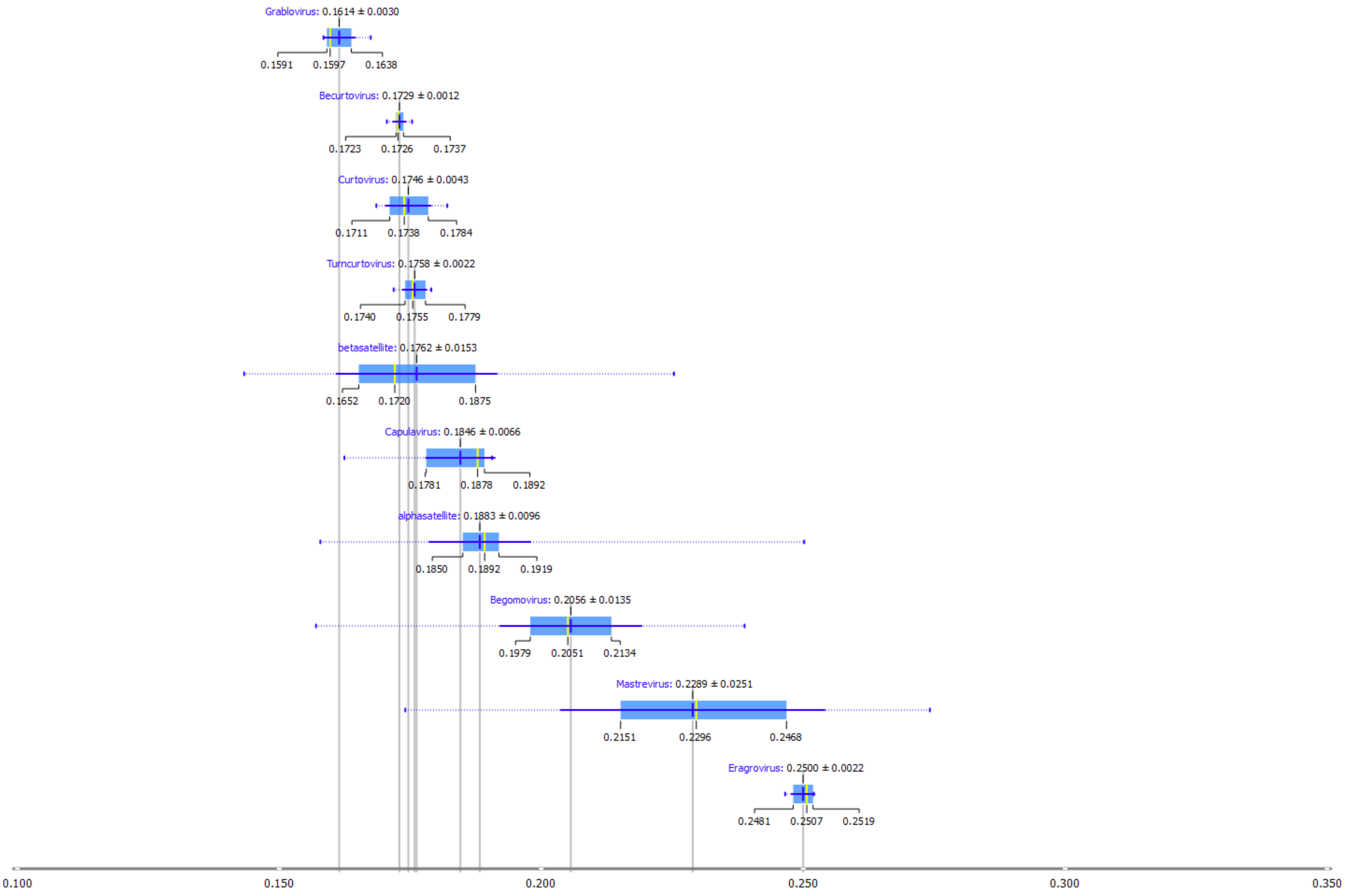

Grablovirus: 0.1614 ± 0.0030
0.1591 0.1597 0.1638
Becurtovirus: 0.1729 ± 0.0012
0.1723 0.1726 0.1737
Curtovirus: 0.1746 ± 0.0043
0.1711 0.1738 0.1784
Turncurtovirus: 0.1758 ± 0.0022
0.1740 0.1755 0.1779
betasatellite: 0.1762 ± 0.0153
0.1652 0.1720 0.1875
Capulavirus: 0.1846 ± 0.0066
0.1781 0.1878 0.1892
alphasatellite: 0.1883 ± 0.0096
0.1850 0.1892 0.1919
Begomovirus: 0.2056 ± 0.0135
0.1979 0.2051 0.2134
Mastrevirus: 0.2289 ± 0.0251
0.2151 0.2296 0.2468
Eragrovirus: 0.2500 ± 0.0022
0.2481 0.2507 0.2519
0.100
0.150
0.200
0.250
0.300
0.350

Proportion of Cytosine in region 1

Histogram

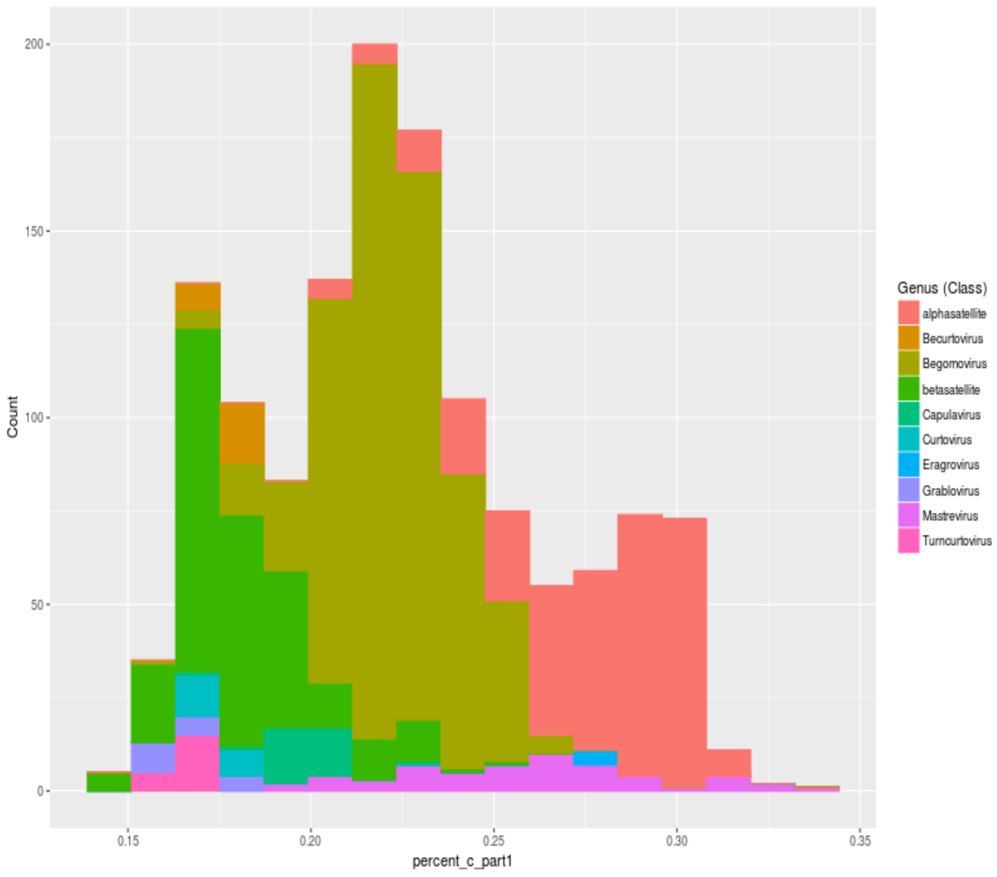

Density

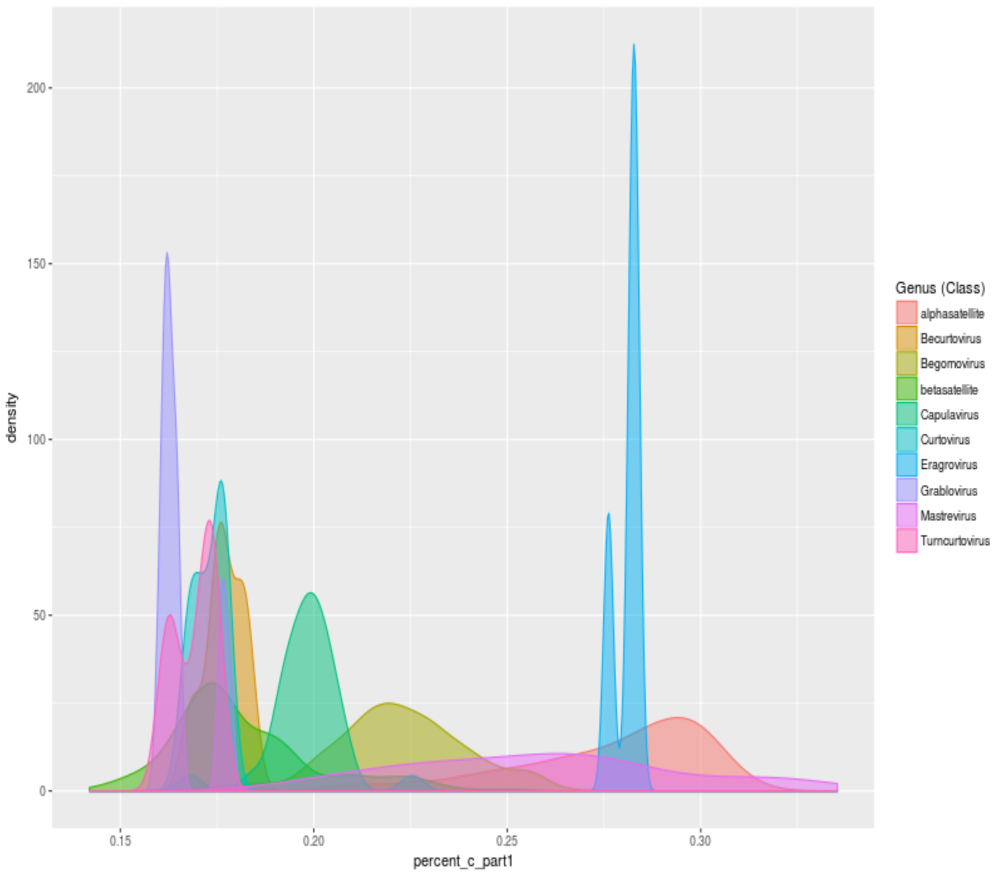

Boxplots

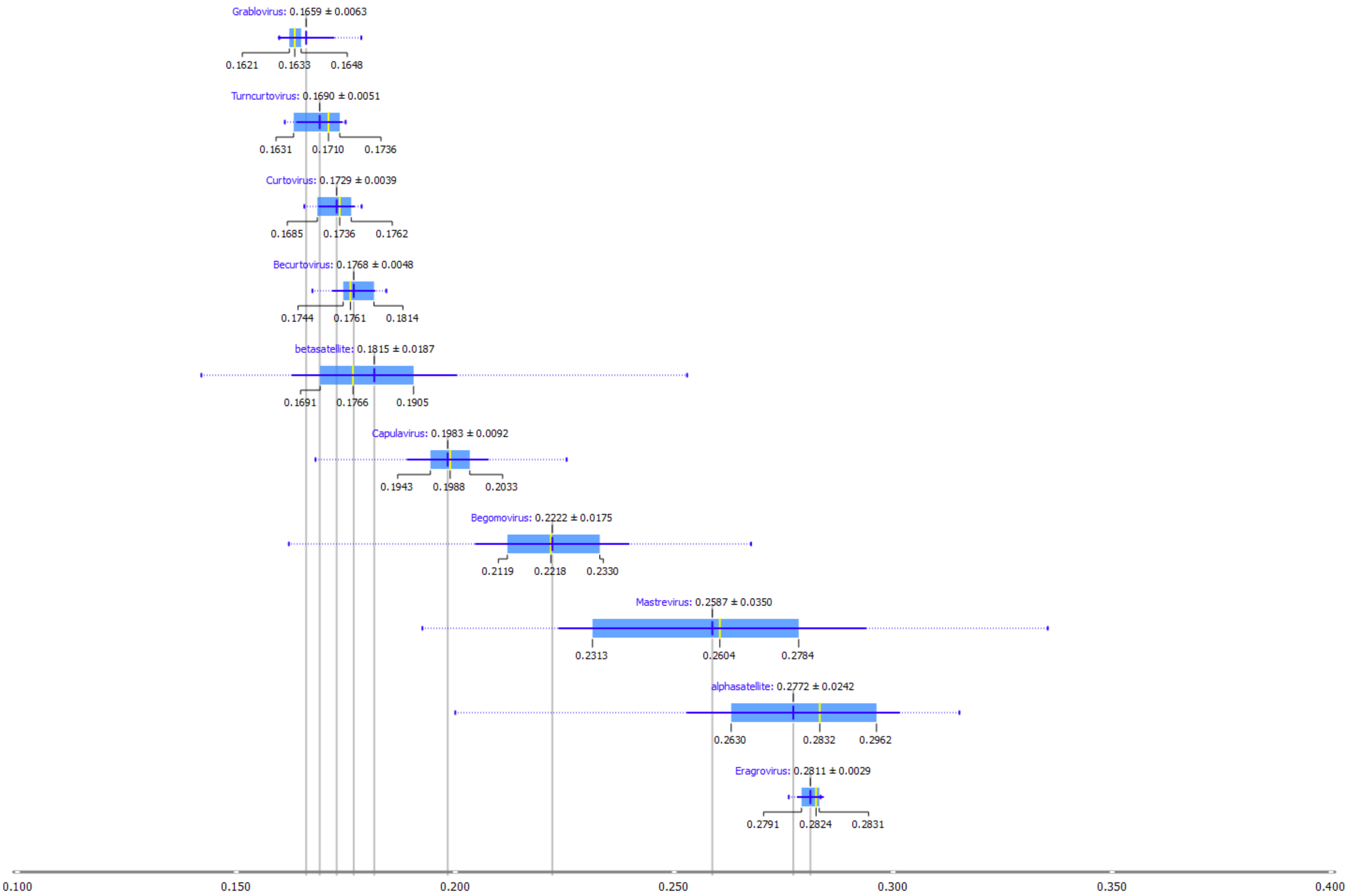

Grablovirus: 0.1659 ± 0.0063
0.1621
0.1633
0.1648
Turncurtovirus: 0.1690 ± 0.0051
0.1631
0.1710
0.1736
Curtovirus: 0.1729 ± 0.0039
0.1685
0.1736
0.1762
Becurtovirus: 0.1768 ± 0.0048
0.1744
0.1761
0.1814
betasatellite: 0.1815 ± 0.0187
0.1691
0.1766
0.1905
Capulavirus: 0.1983 ± 0.0092
0.1943
0.1988
0.2033
Begomovirus: 0.2222 ± 0.0175
0.2119
0.2218
0.2330
Mastrevirus: 0.2587 ± 0.0350
0.2313
0.2604
0.2784
alphasatellite: 0.2772 ± 0.0242
0.2630
0.2832
0.2962
Eragrovirus: 0.2811 ± 0.0029
0.2791
0.2824
0.2831
0.100
0.150
0.200
0.250
0.300
0.350
0.400

Proportion of Cytosine in region 2

Histogram

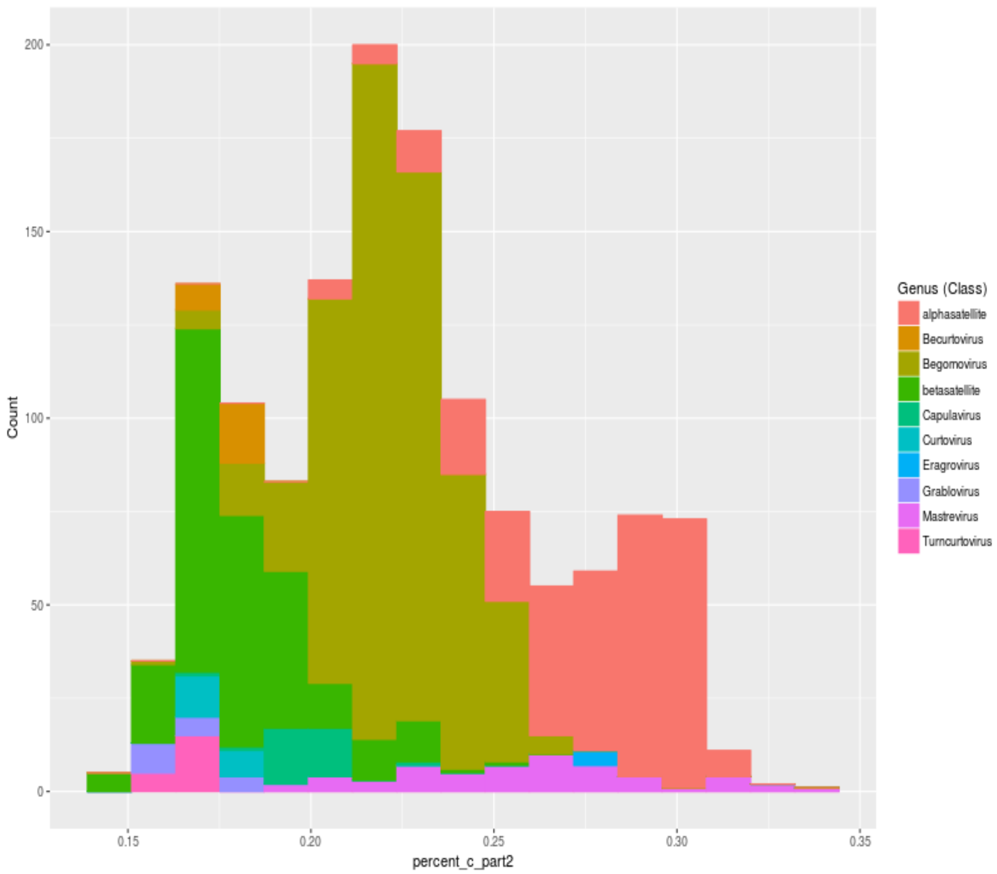

Density

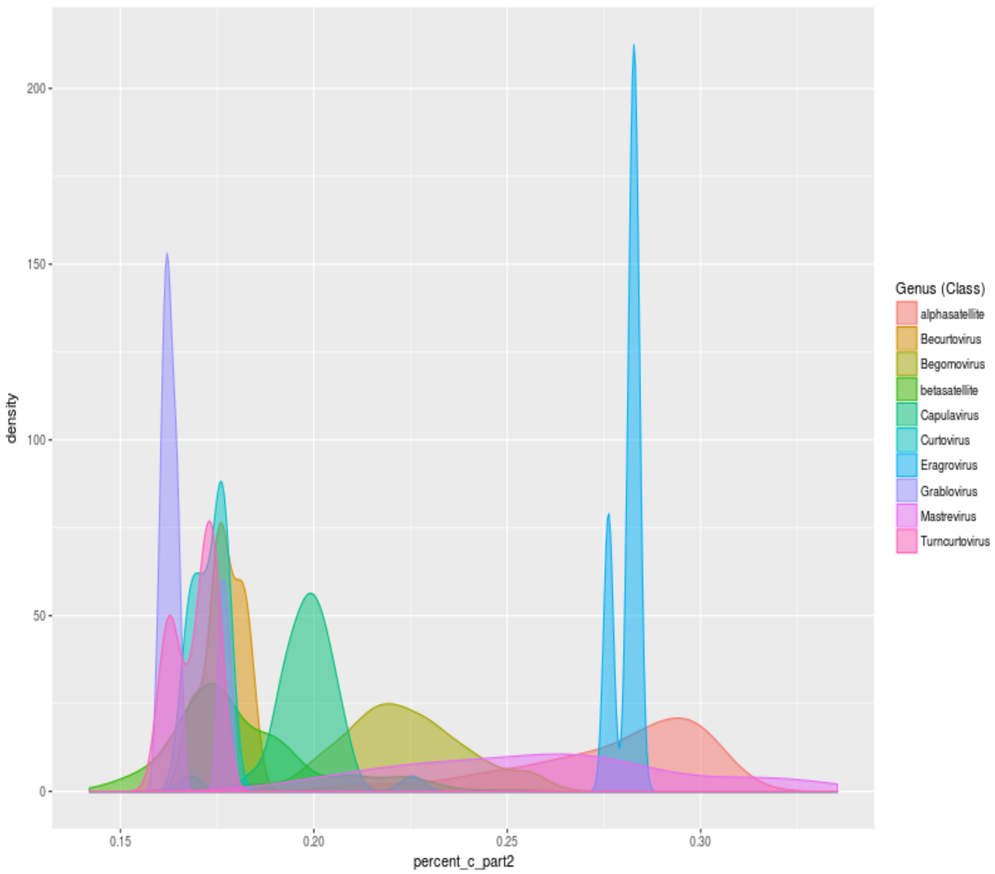

Boxplots

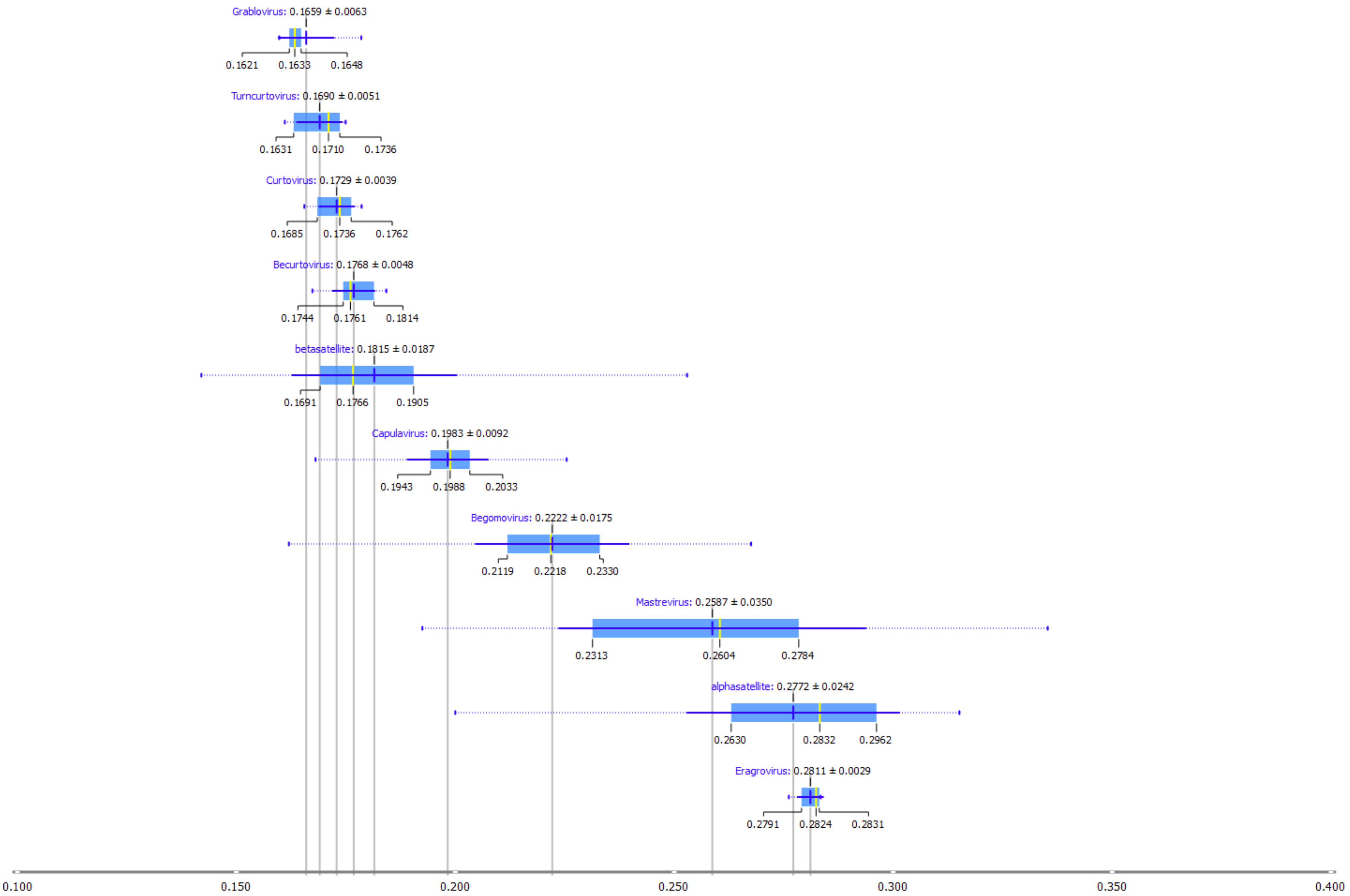

Grablovirus: 0.1659 ± 0.0063
0.1621
0.1633
0.1648
Turncurtovirus: 0.1690 ± 0.0051
0.1631
0.1710
0.1736
Curtovirus: 0.1729 ± 0.0039
0.1685
0.1736
0.1762
Becurtovirus: 0.1768 ± 0.0048
0.1744
0.1761
0.1814
betasatellite: 0.1815 ± 0.0187
0.1691
0.1766
0.1905
Capulavirus: 0.1983 ± 0.0092
0.1943
0.1988
0.2033
Begomovirus: 0.2222 ± 0.0175
0.2119
0.2218
0.2330
Mastrevirus: 0.2587 ± 0.0350
0.2313
0.2604
0.2784
alphasatellite: 0.2772 ± 0.0242
0.2630
0.2832
0.2962
Eragrovirus: 0.2811 ± 0.0029
0.2791
0.2824
0.2831
0.100
0.150
0.200
0.250
0.300
0.350
0.400

Proportion of Cytosine in region 3

Histogram

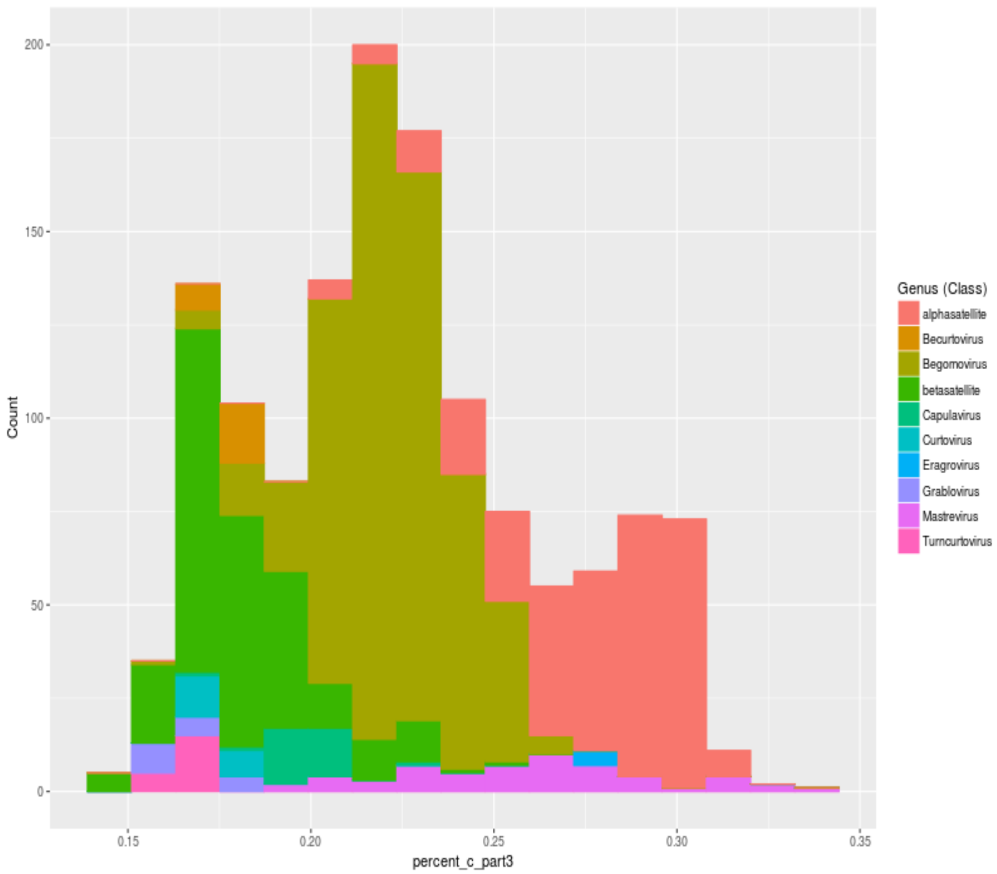

Density

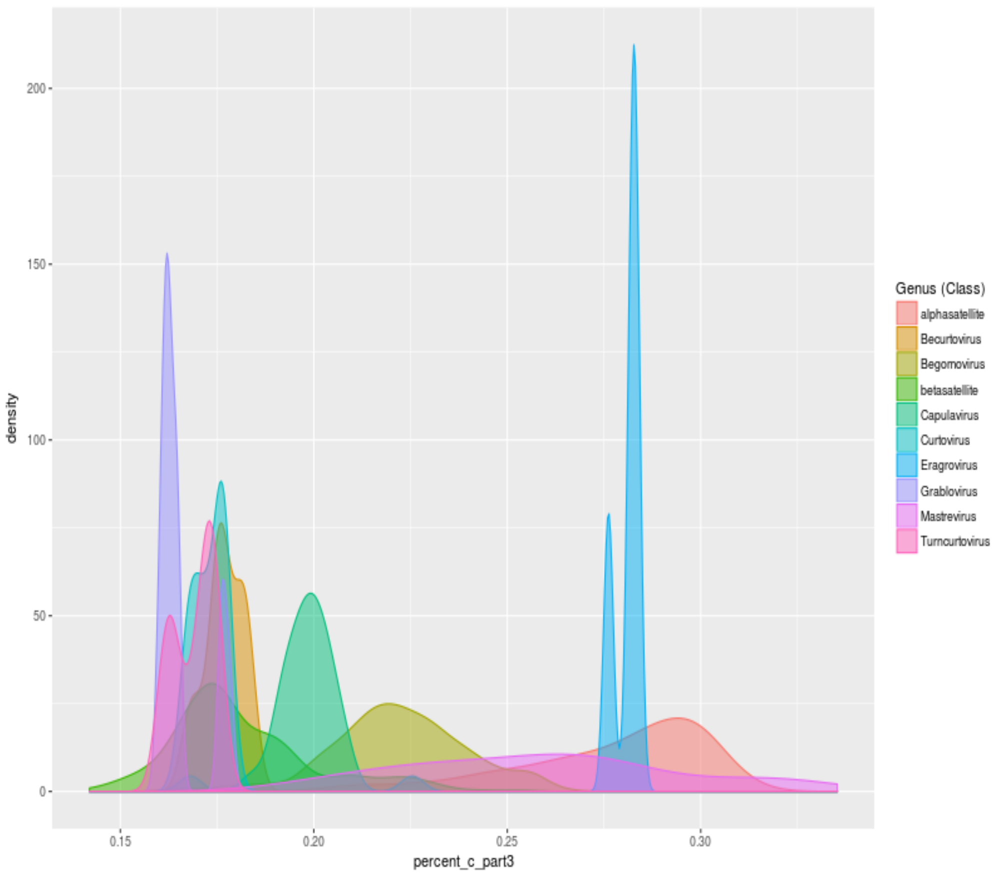

Boxplots

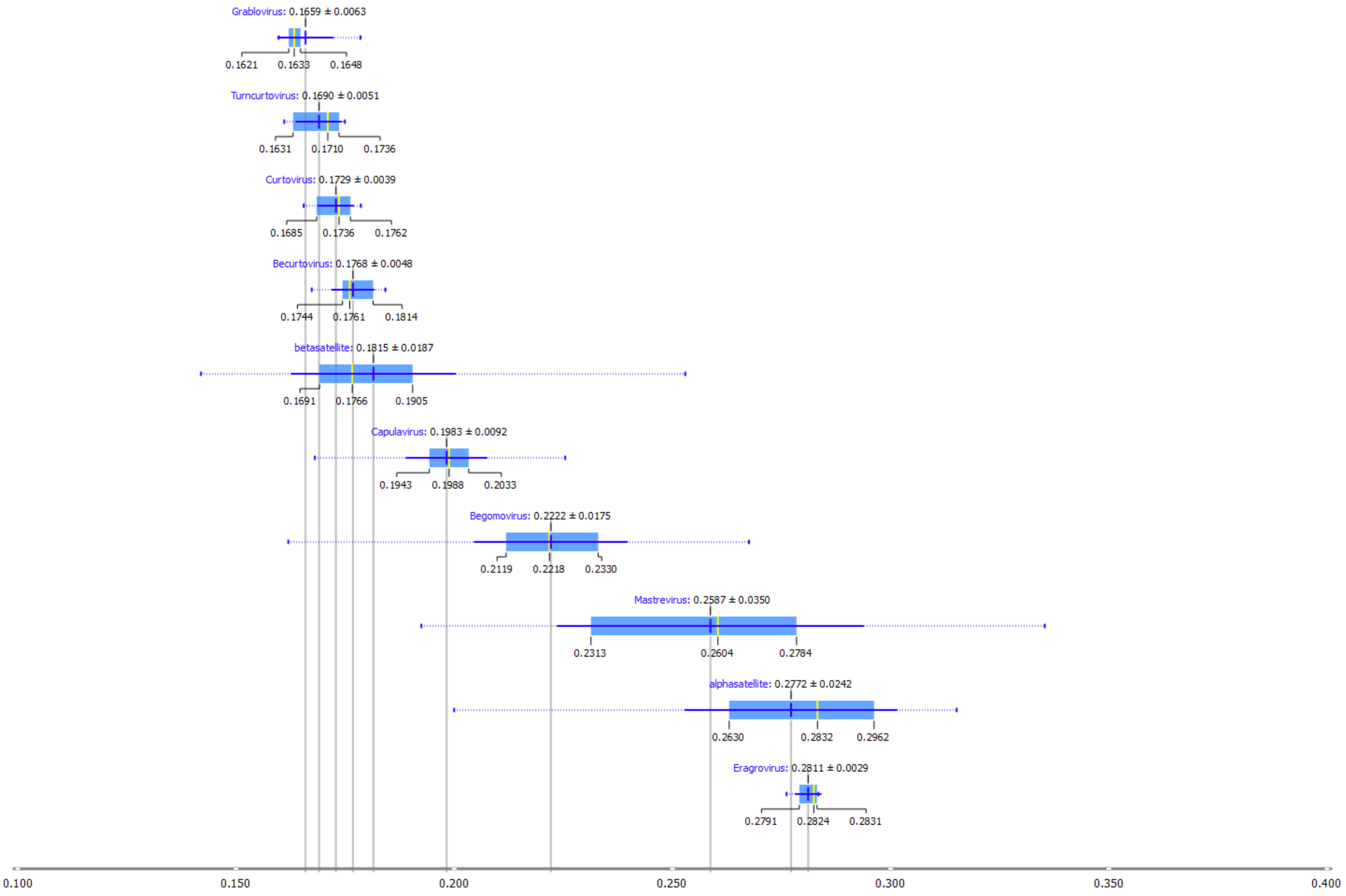

Grablovirus: 0.1659 ± 0.0063
0.1621
0.1633
0.1648
Turncurtovirus: 0.1690 ± 0.0051
0.1631
0.1710
0.1736
Curtovirus: 0.1729 ± 0.0039
0.1685
0.1736
0.1762
Becurtovirus: 0.1768 ± 0.0048
0.1744
0.1761
0.1814
betasatellite: 0.1815 ± 0.0187
0.1691
0.1766
0.1905
Capulavirus: 0.1983 ± 0.0092
0.1943
0.1988
0.2033
Begomovirus: 0.2222 ± 0.0175
0.2119
0.2218
0.2330
Mastrevirus: 0.2587 ± 0.0350
0.2313
0.2604
0.2784
alphasatellite: 0.2772 ± 0.0242
0.2630
0.2832
0.2962
Eragrovirus: 0.2811 ± 0.0029
0.2791
0.2824
0.2831
0.100
0.150
0.200
0.250
0.300
0.350
0.400

Proportion of Cytosine in region 4

Histogram

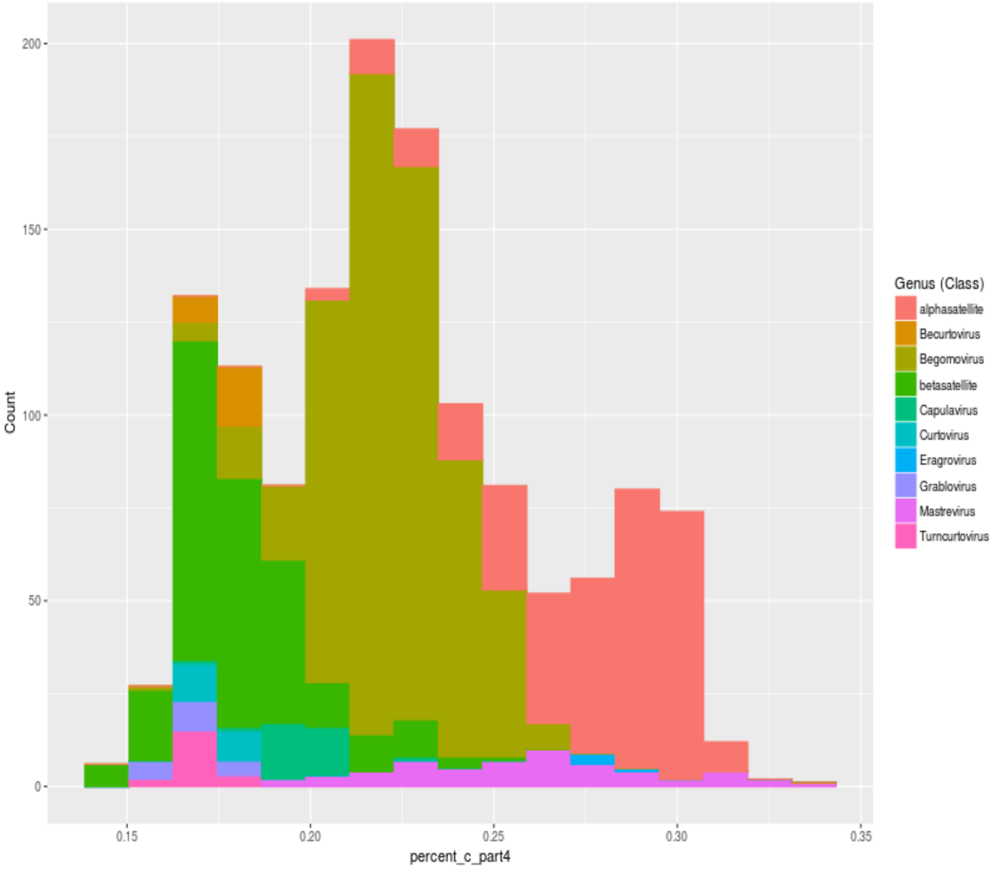

Density

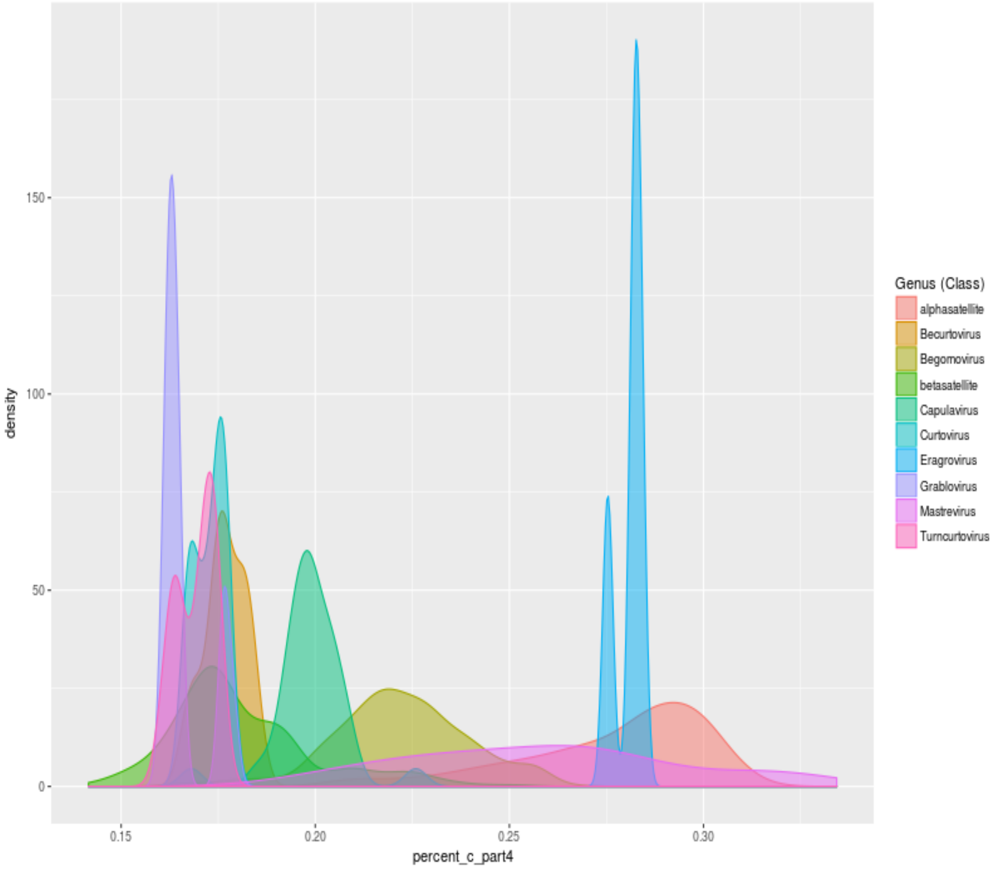

Boxplots

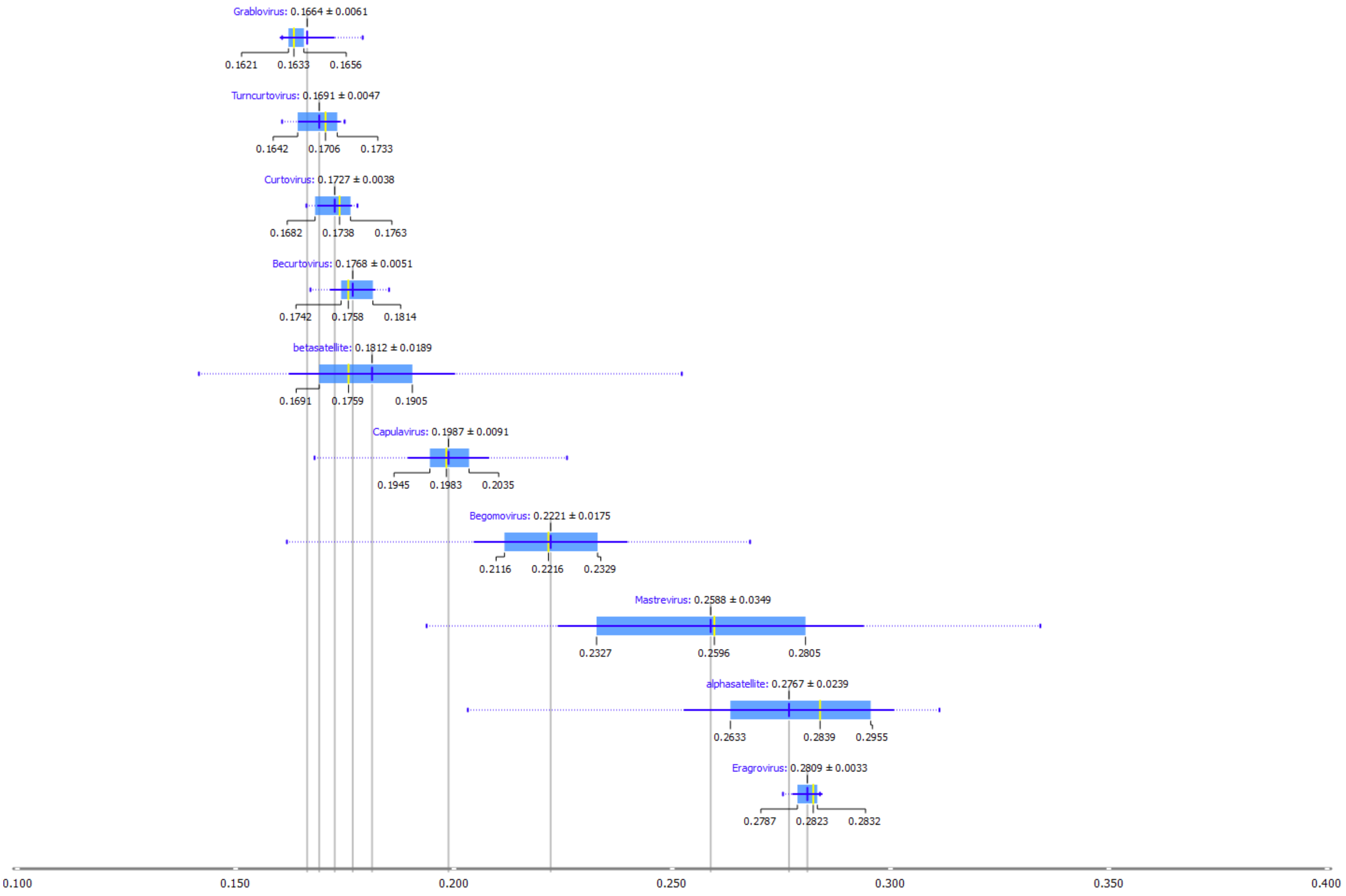

Grablovirus: 0.1664 ± 0.0061
0.1621
0.1633
0.1656
Turncurtovirus: 0.1691 ± 0.0047
0.1642
0.1706
0.1733
Curtovirus: 0.1727 ± 0.0038
0.1682
0.1738
0.1763
Becurtovirus: 0.1768 ± 0.0051
0.1742
0.1758
0.1814
betasatellite: 0.1812 ± 0.0189
0.1691
0.1759
0.1905
Capulavirus: 0.1987 ± 0.0091
0.1945
0.1983
0.2035
Begomovirus: 0.2221 ± 0.0175
0.2116
0.2216
0.2329
Mastrevirus: 0.2588 ± 0.0349
0.2327
0.2596
0.2805
alphasatellite: 0.2767 ± 0.0239
0.2633
0.2839
0.2955
Eragrovirus: 0.2809 ± 0.0033
0.2787
0.2823
0.2832
0.100
0.150
0.200
0.250
0.300
0.350
0.400

Proportion of Guanine and Cytosine in region 1

Histogram

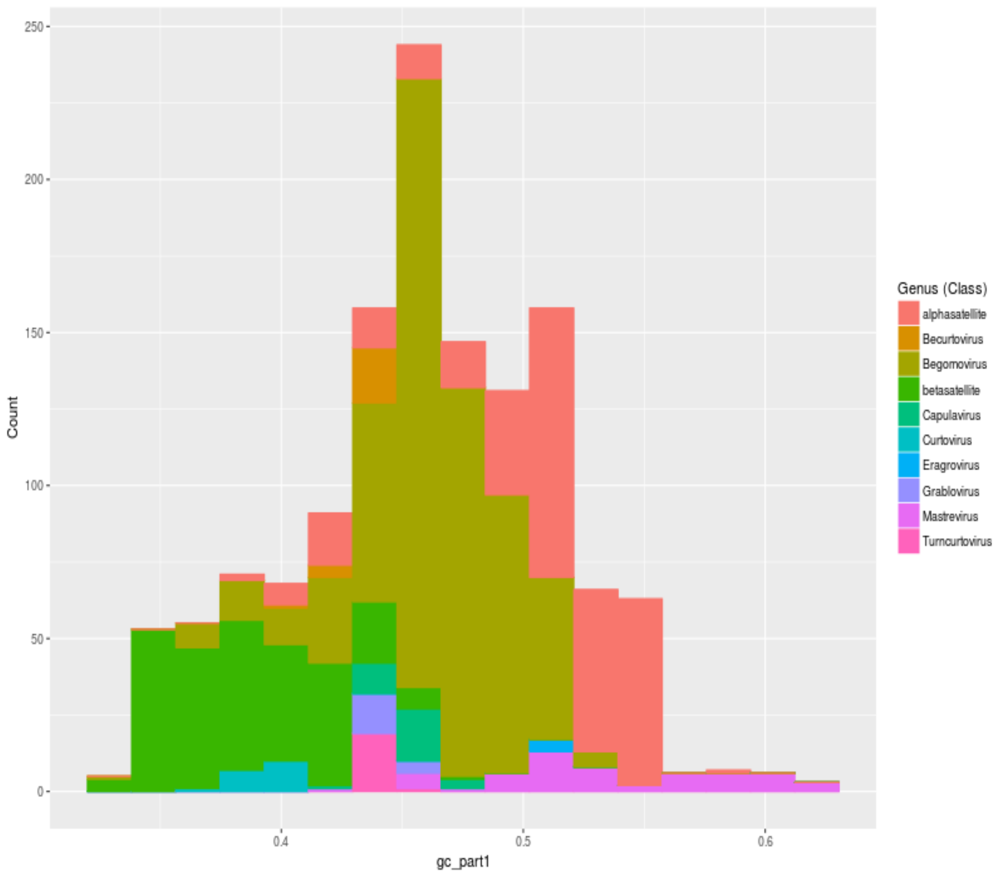

Density

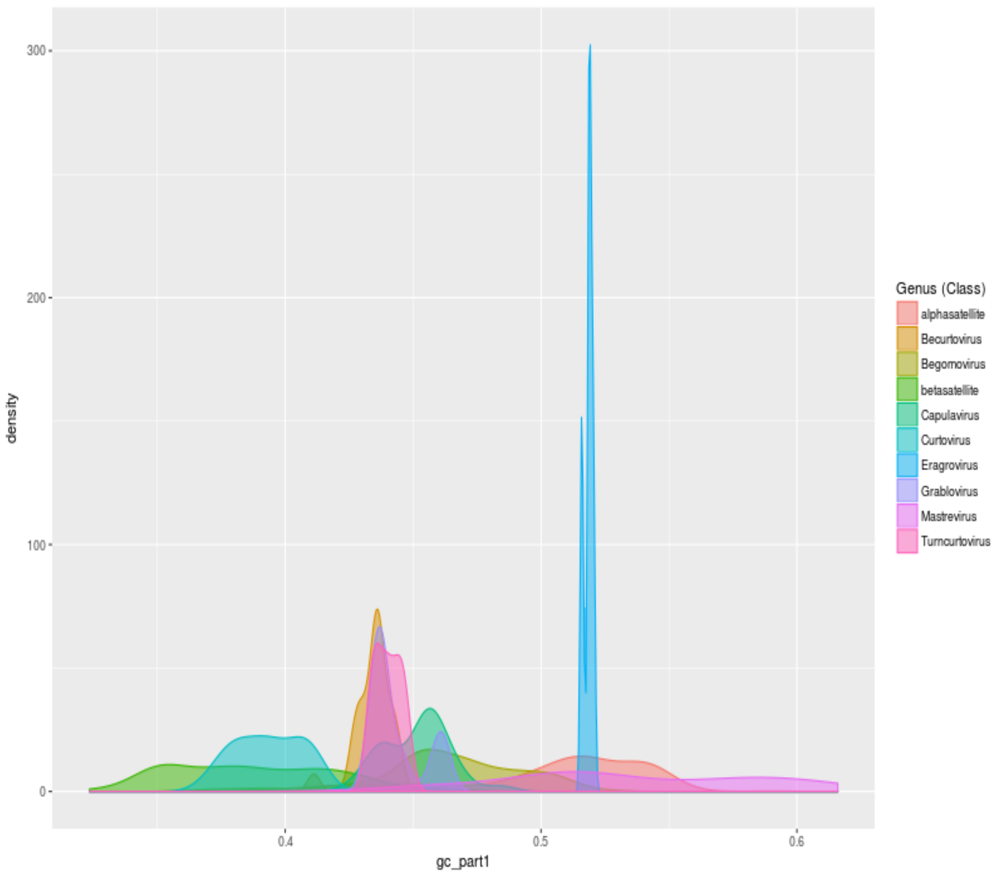

Boxplots

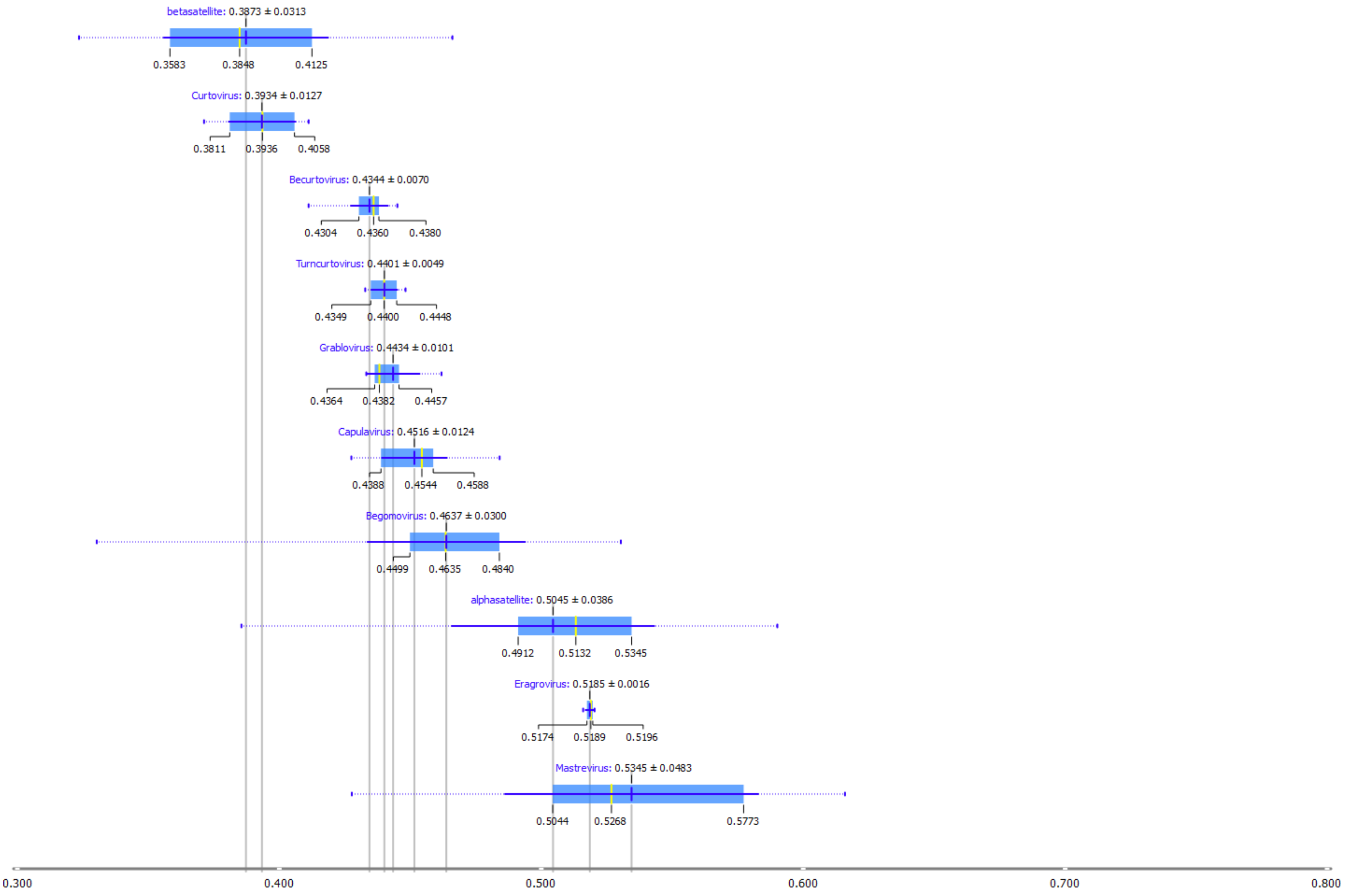

betasatellite: 0.3873 ± 0.0313
0.3583
0.3848
0.4125
Curtovirus: 0.3934 ± 0.0127
0.3811
0.3936
0.4058
Becurtovirus: 0.4344 ± 0.0070
0.4304
0.4360
0.4380
Turncurtovirus: 0.4401 ± 0.0049
0.4349
0.4400
0.4448
Grablovirus: 0.4434 ± 0.0101
0.4364
0.4382
0.4457
Capulavirus: 0.4516 ± 0.0124
0.4388
0.4544
0.4588
Begomovirus: 0.4637 ± 0.0300
0.4499
0.4635
0.4840
alphasatellite: 0.5045 ± 0.0386
0.4912
0.5132
0.5345
Eragrovirus: 0.5185 ± 0.0016
0.5174
0.5189
0.5196
Mastrevirus: 0.5345 ± 0.0483
0.5044
0.5268
0.5773
0.300
0.400
0.500
0.600
0.700
0.800

Proportion of Guanine and Cytosine in region 2

Histogram

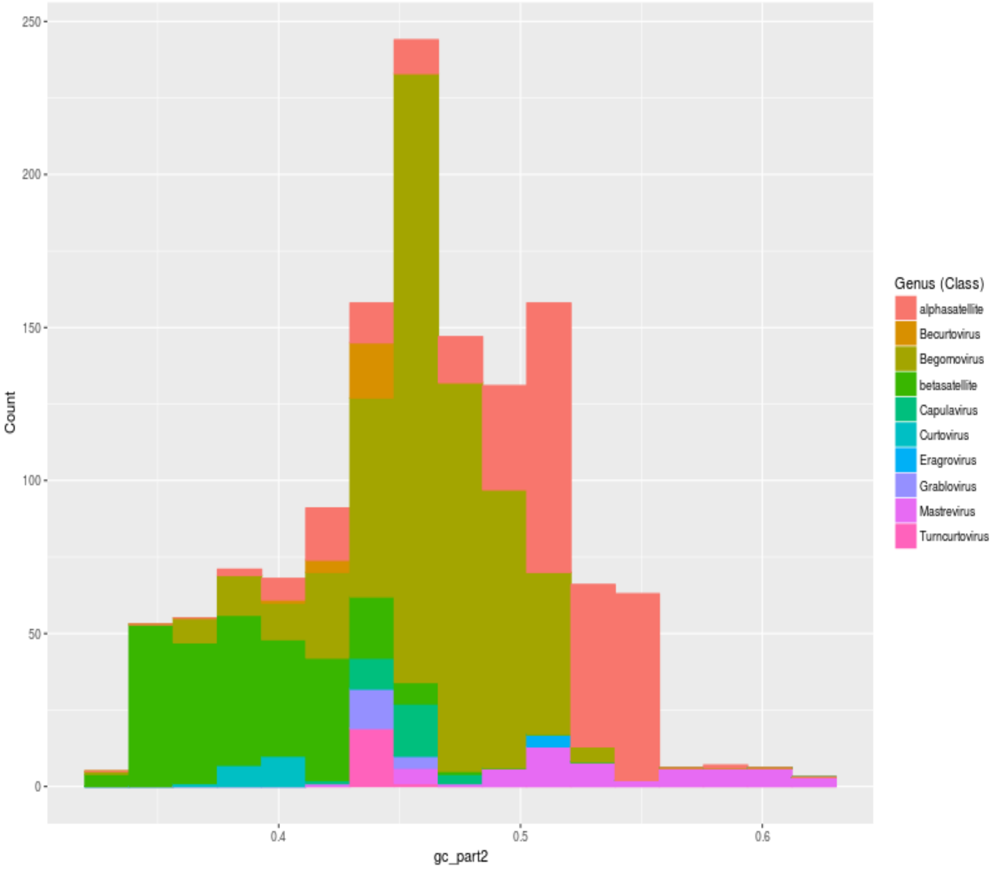

Density

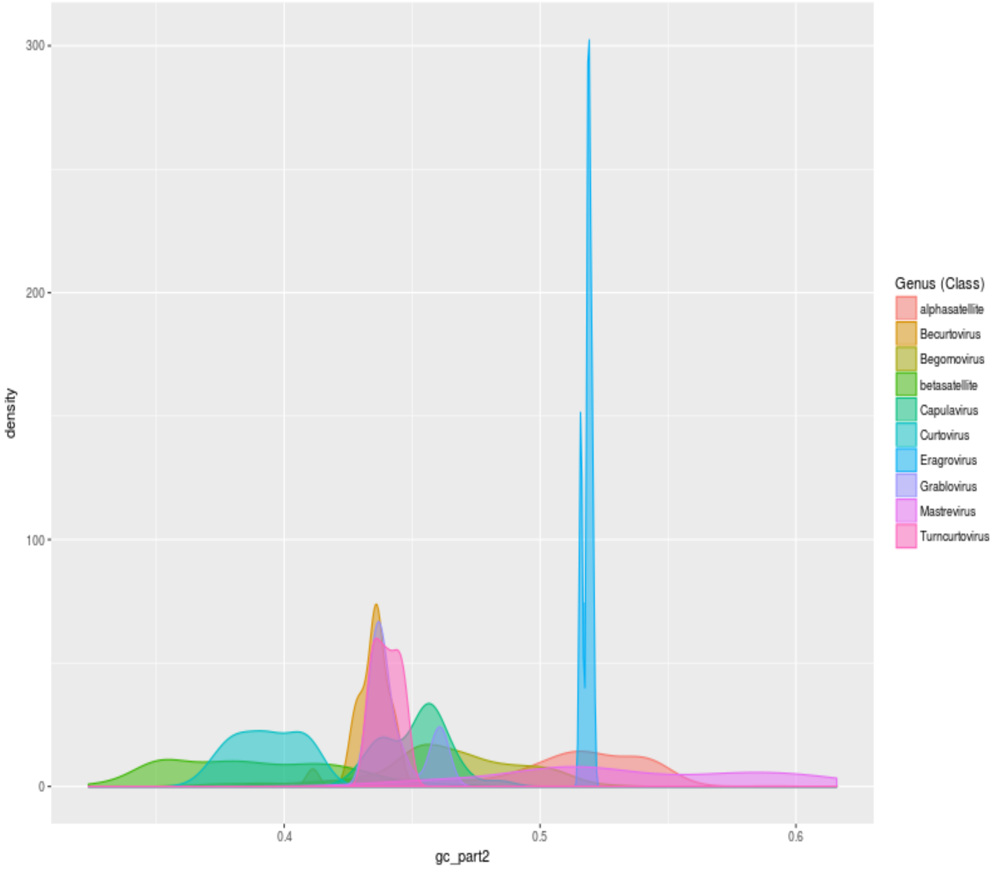

Boxplots

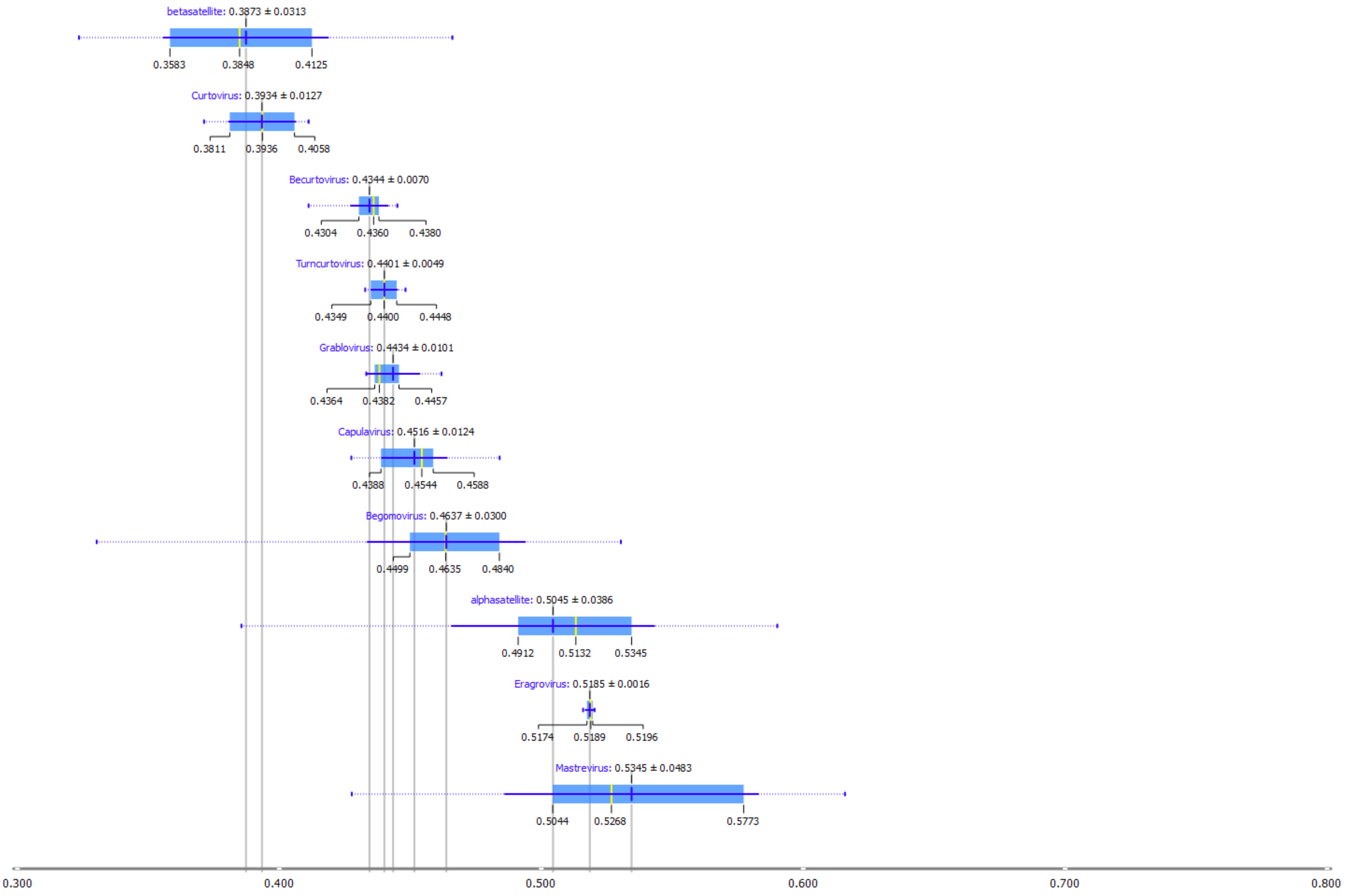

betasatellite: 0.3873 ± 0.0313
0.3583
0.3848
0.4125
Curtovirus: 0.3934 ± 0.0127
0.3811
0.3936
0.4058
Becurtovirus: 0.4344 ± 0.0070
0.4304
0.4360
0.4380
Turncurtovirus: 0.4401 ± 0.0049
0.4349
0.4400
0.4448
Grablovirus: 0.4434 ± 0.0101
0.4364
0.4382
0.4457
Capulavirus: 0.4516 ± 0.0124
0.4388
0.4544
0.4588
Begomovirus: 0.4637 ± 0.0300
0.4499
0.4635
0.4840
alphasatellite: 0.5045 ± 0.0386
0.4912
0.5132
0.5345
Eragrovirus: 0.5185 ± 0.0016
0.5174
0.5189
0.5196
Mastrevirus: 0.5345 ± 0.0483
0.5044
0.5268
0.5773
0.300
0.400
0.500
0.600
0.700
0.800

Proportion of Guanine and Cytosine in region 3

Histogram

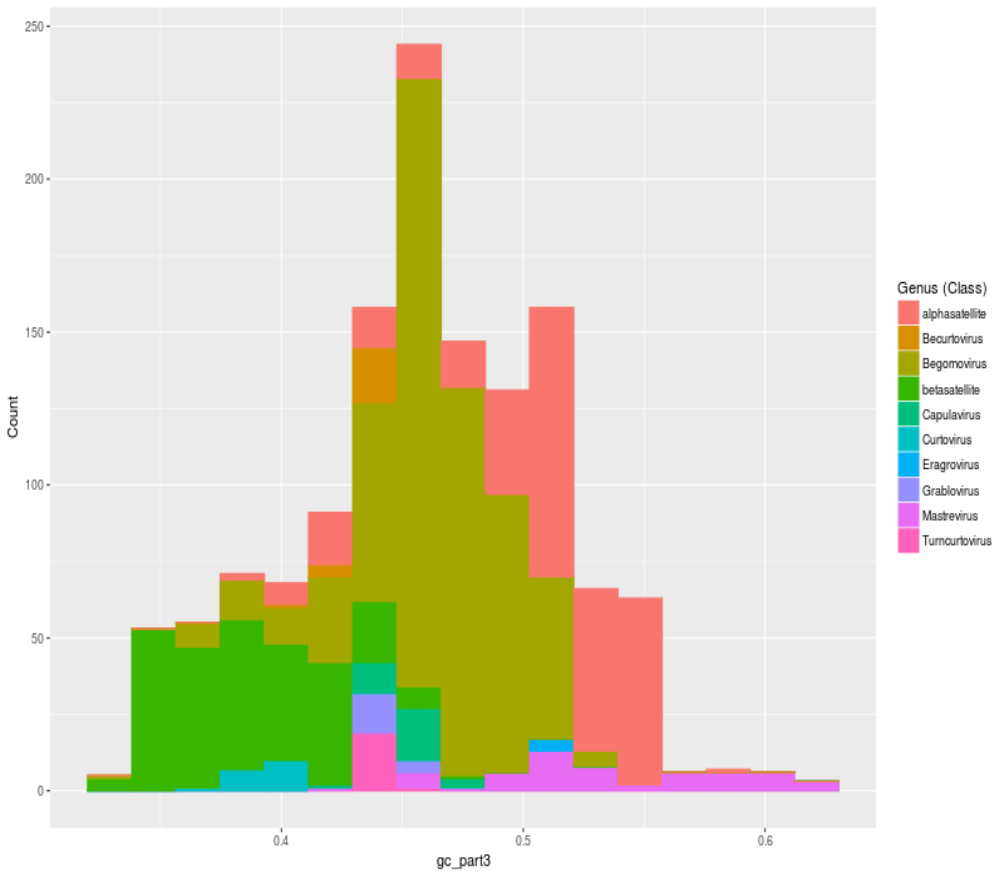

Density

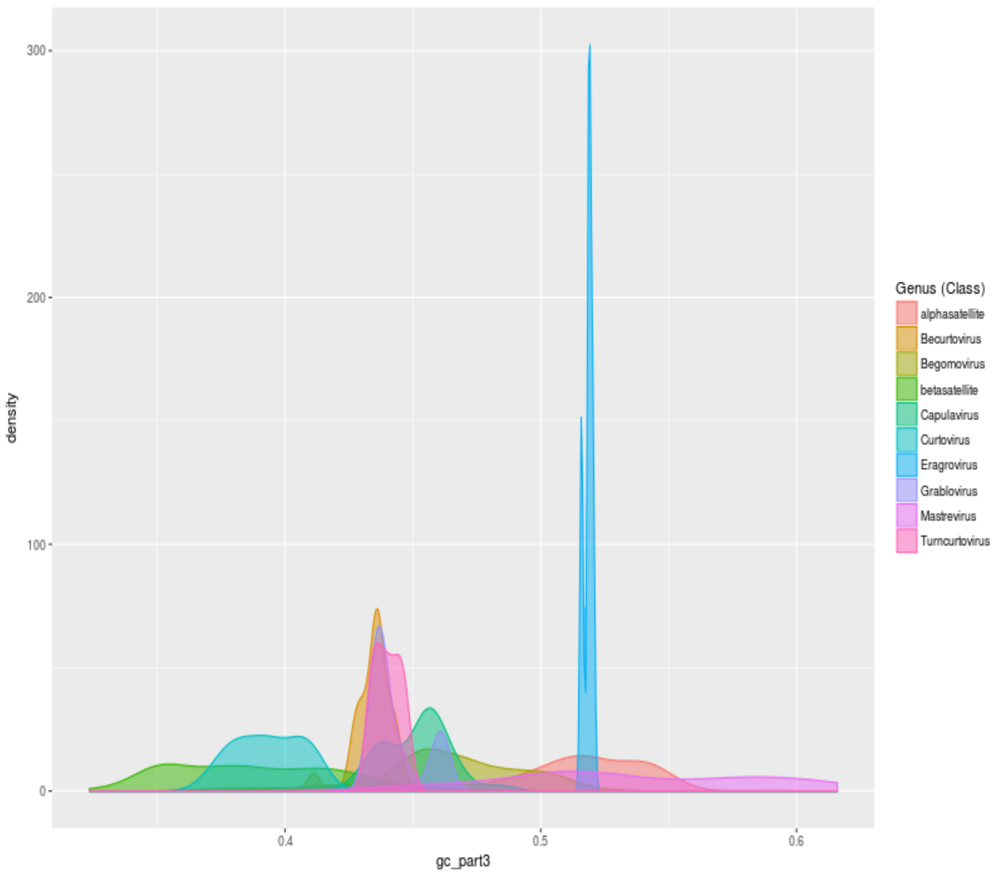

Boxplots

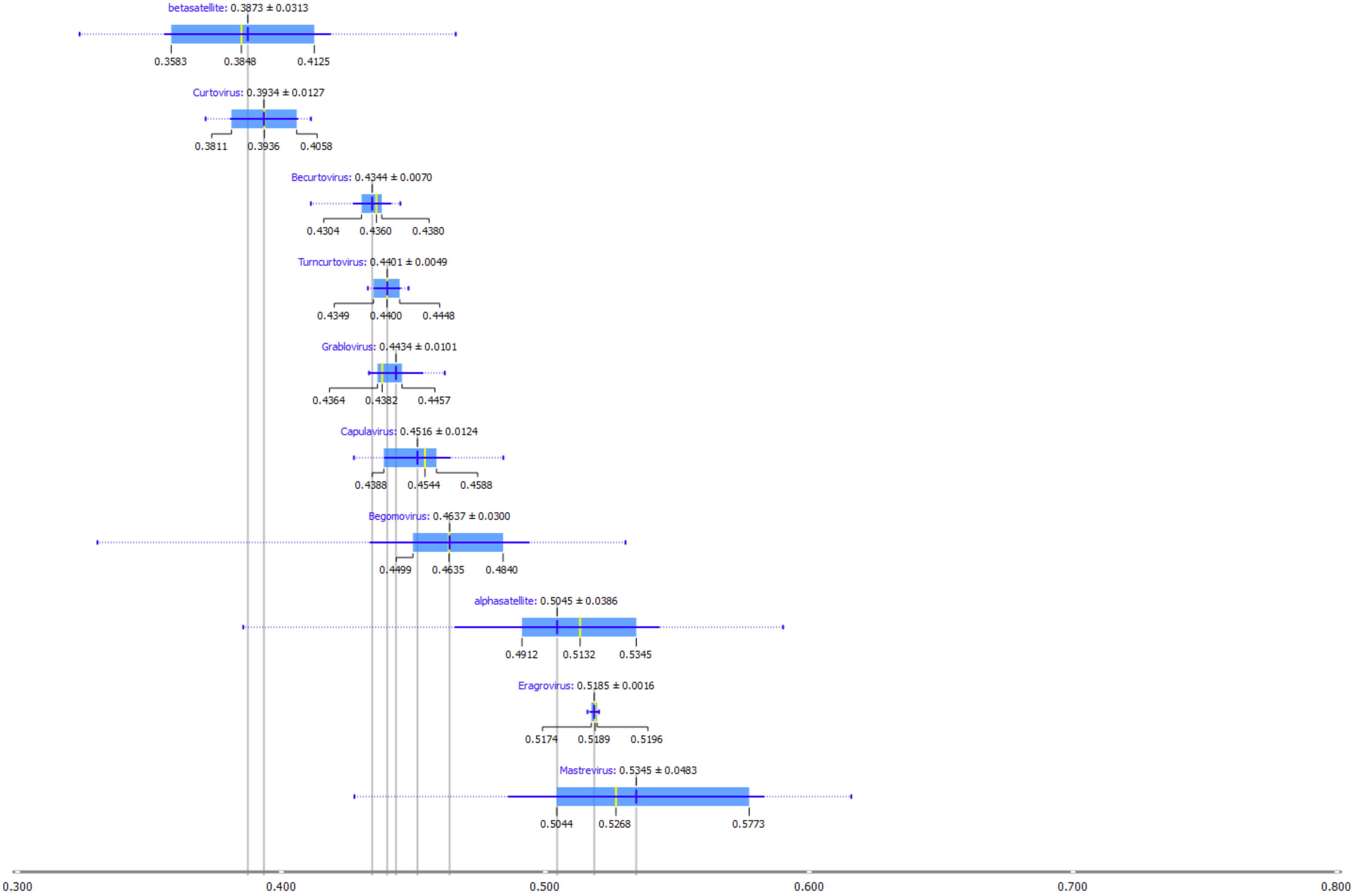

betasatellite: 0.3873 ± 0.0313
0.3583
0.3848
0.4125
Curtovirus: 0.3934 ± 0.0127
0.3811
0.3936
0.4058
Becurtovirus: 0.4344 ± 0.0070
0.4304
0.4360
0.4380
Turncurtovirus: 0.4401 ± 0.0049
0.4349
0.4400
0.4448
Grablovirus: 0.4434 ± 0.0101
0.4364
0.4382
0.4457
Capulavirus: 0.4516 ± 0.0124
0.4388
0.4544
0.4588
Begomovirus: 0.4637 ± 0.0300
0.4499
0.4635
0.4840
alphasatellite: 0.5045 ± 0.0386
0.4912
0.5132
0.5345
Eragrovirus: 0.5185 ± 0.0016
0.5174
0.5189
0.5196
Mastrevirus: 0.5345 ± 0.0483
0.5044
0.5268
0.5773
0.300
0.400
0.500
0.600
0.700
0.800

Proportion of Guanine and Cytosine in region 4

Histogram

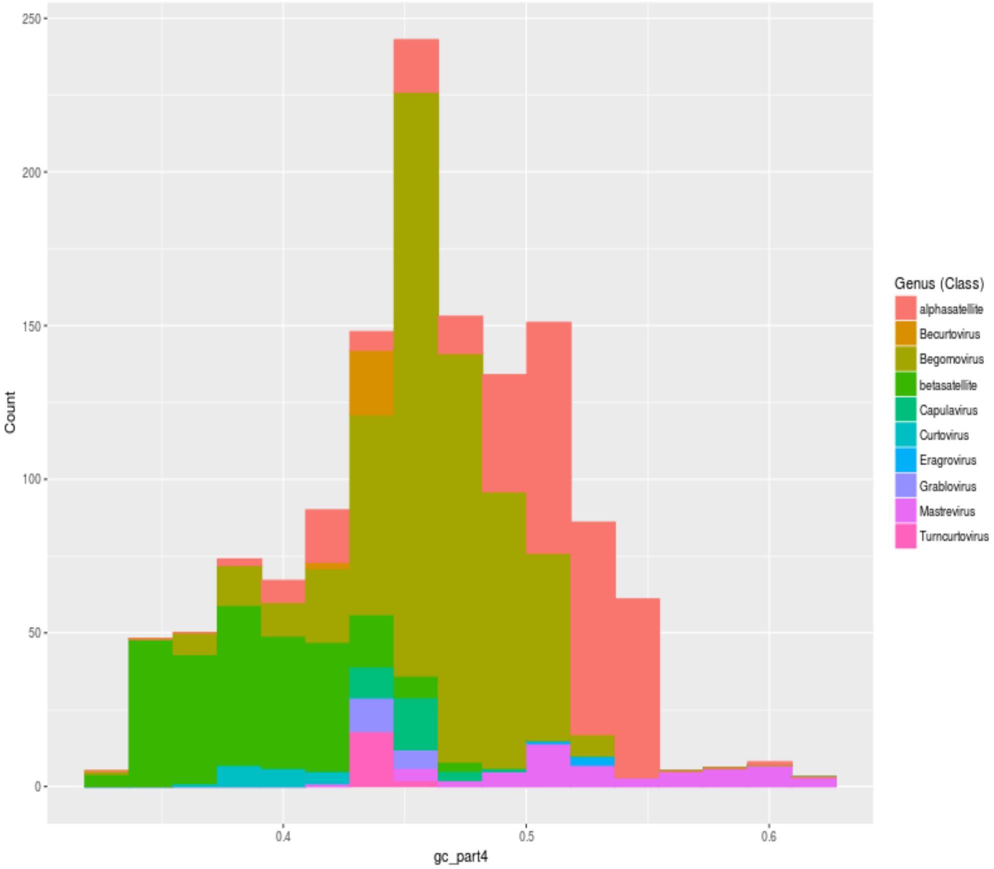

Density

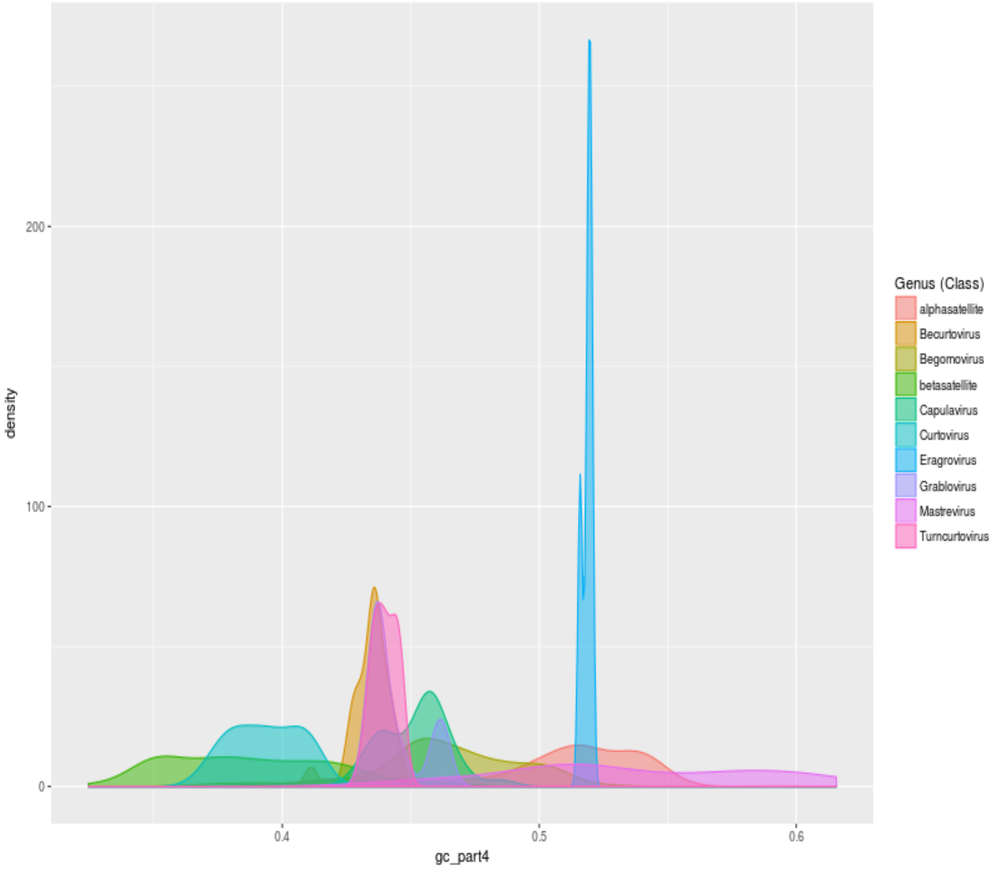

Boxplots

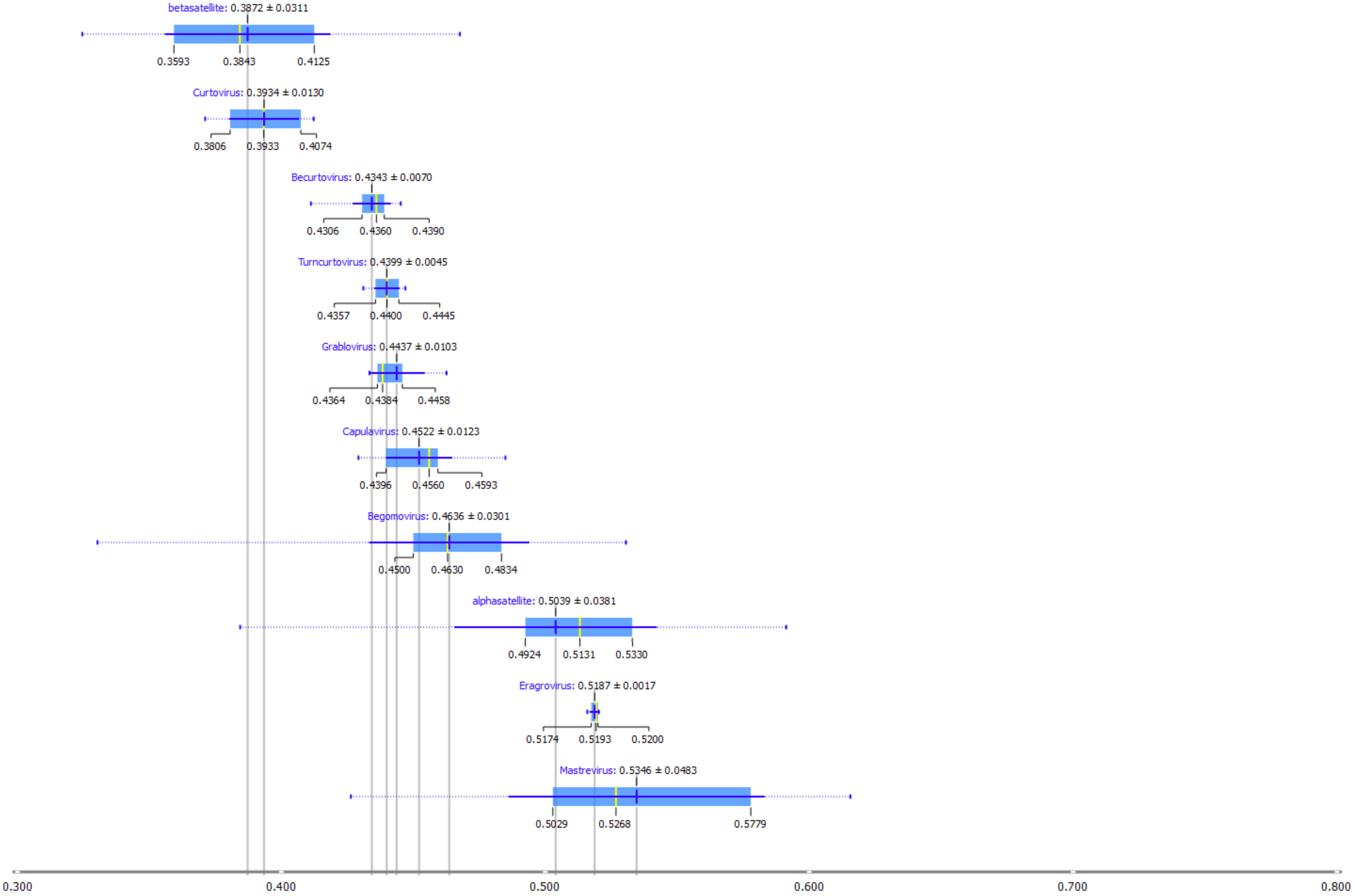

betasatellite: 0.3872 ± 0.0311
0.3593
0.3843
0.4125
Curtovirus: 0.3934 ± 0.0130
0.3806
0.3933
0.4074
Becurtovirus: 0.4343 ± 0.0070
0.4306
0.4360
0.4390
Turncurtovirus: 0.4399 ± 0.0045
0.4357
0.4400
0.4445
Grablovirus: 0.4437 ± 0.0103
0.4364
0.4384
0.4458
Capulavirus: 0.4522 ± 0.0123
0.4396
0.4560
0.4593
Begomovirus: 0.4636 ± 0.0301
0.4500
0.4630
0.4834
alphasatellite: 0.5039 ± 0.0381
0.4924
0.5131
0.5330
Eragrovirus: 0.5187 ± 0.0017
0.5174
0.5193
0.5200
Mastrevirus: 0.5346 ± 0.0483
0.5029
0.5268
0.5779
0.300
0.400
0.500
0.600
0.700
0.800

Charts of ORF attributes

Proportion of Arginine

Histogram

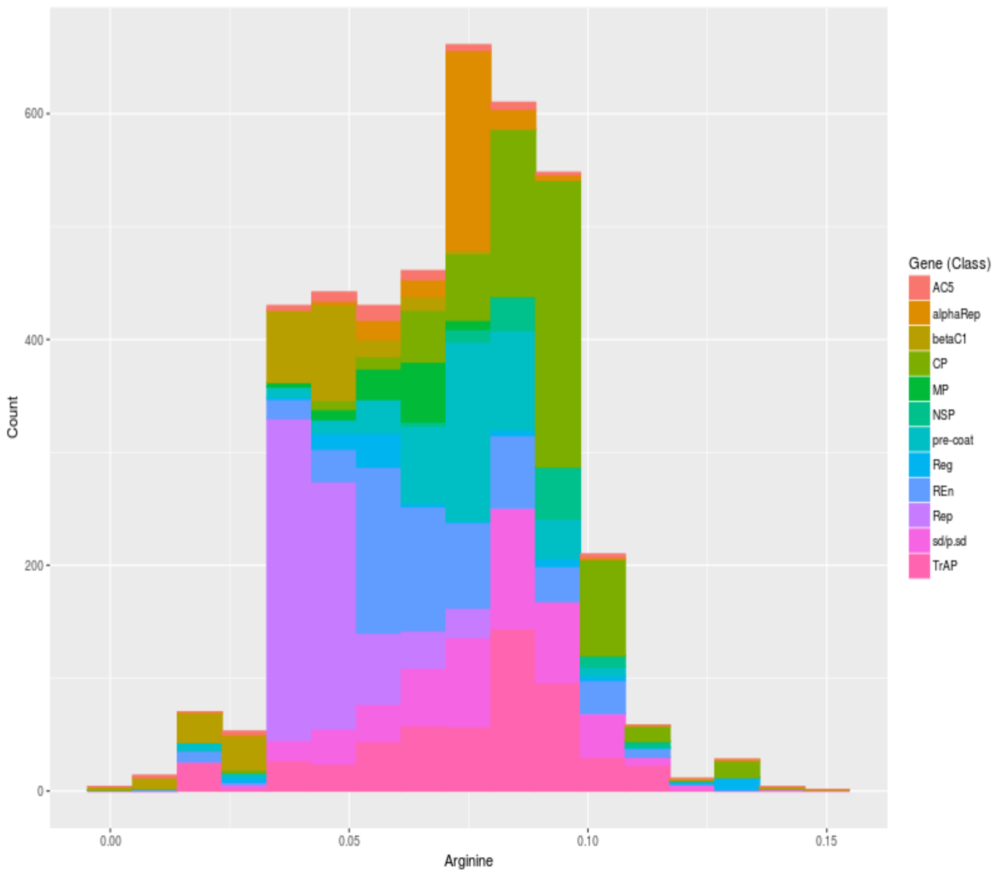

Density

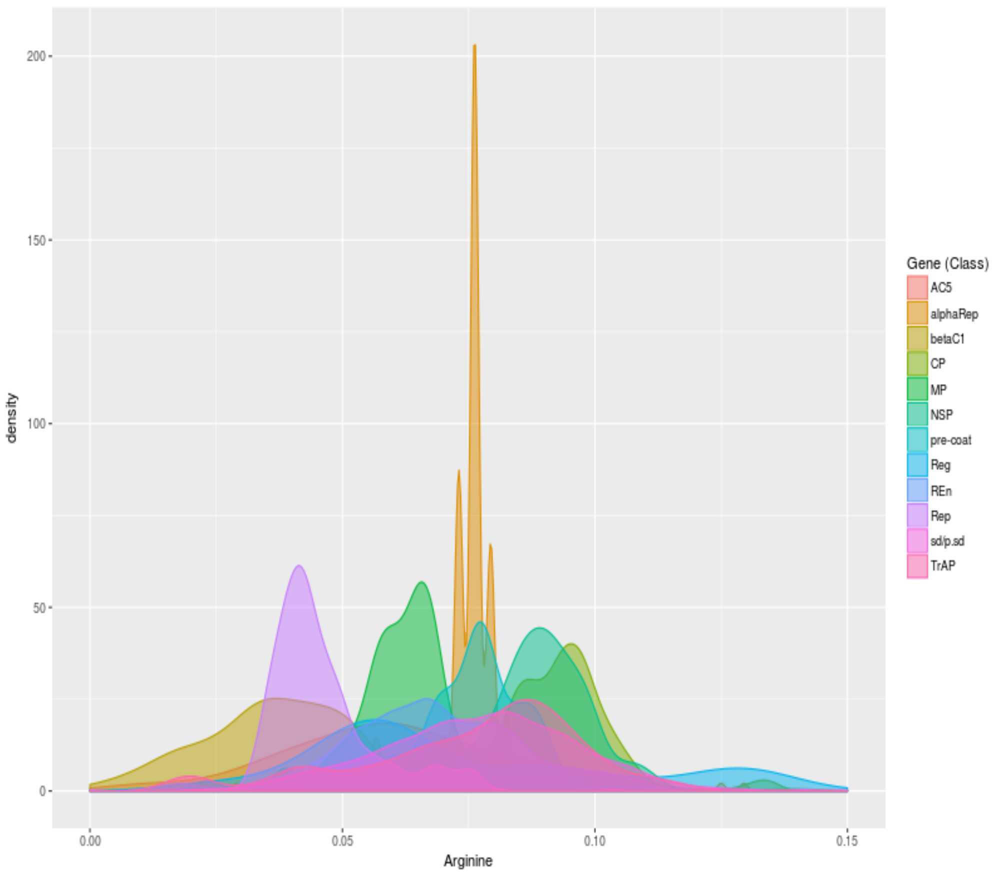

Boxplots

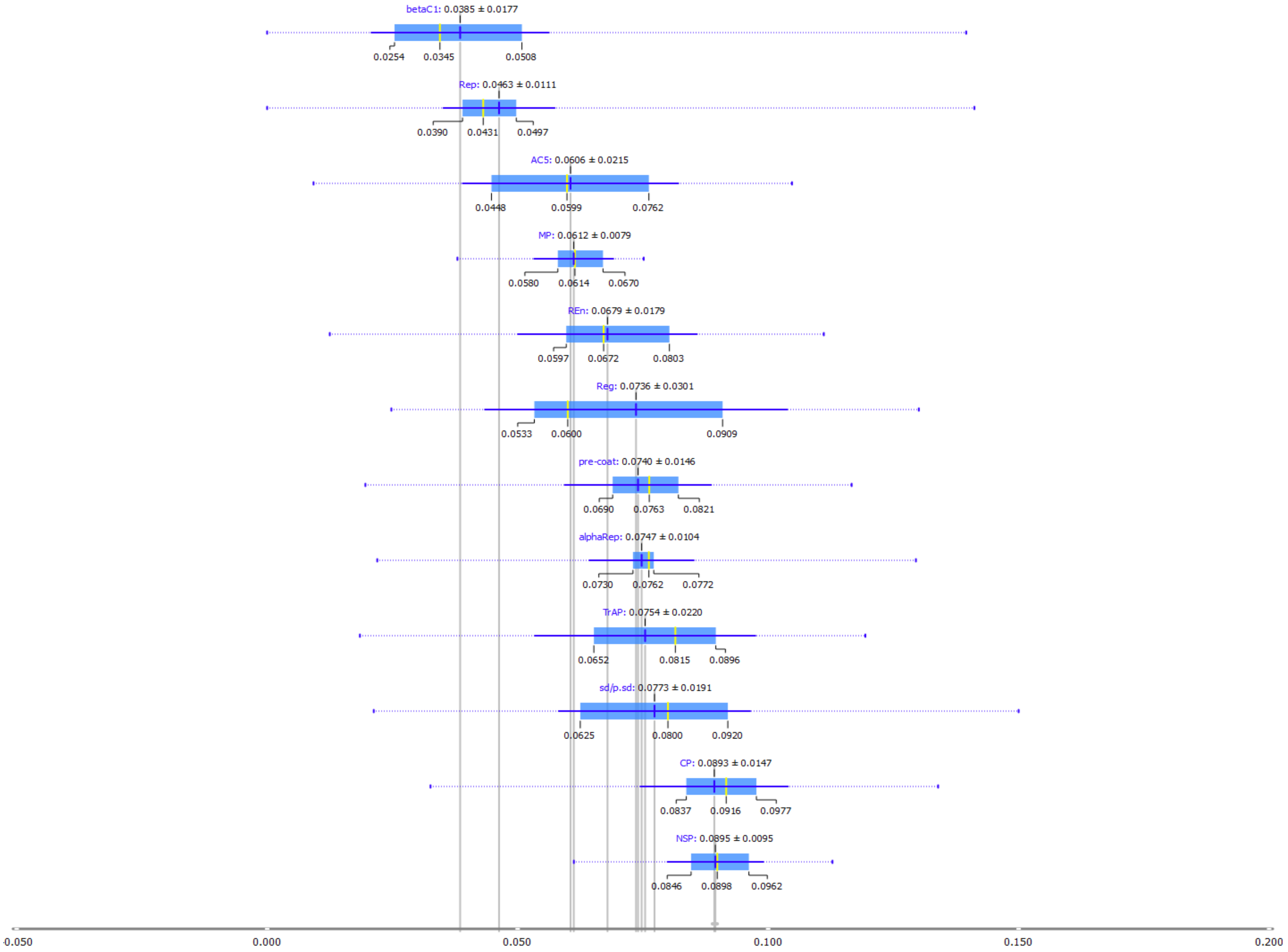
betaC1: 0.0385 ± 0.0177
0.0254
0.0345
0.0508
Rep: 0.0463 ± 0.0111
0.0390
0.0431
0.0497
AC5: 0.0606 ± 0.0215
0.0448
0.0599
0.0762
MP: 0.0612 ± 0.0079
0.0580
0.0614
0.0670
REn: 0.0679 ± 0.0179
0.0597
0.0672
0.0803
Reg: 0.0736 ± 0.0301
0.0533
0.0600
0.0909
pre-coat: 0.0740 ± 0.0146
0.0690
0.0763
0.0821
alphaRep: 0.0747 ± 0.0104
0.0730
0.0762
0.0772
TrAP: 0.0754 ± 0.0220
0.0652
0.0815
0.0896
sd/p.sd: 0.0773 ± 0.0191
0.0625
0.0800
0.0920
CP: 0.0893 ± 0.0147
0.0837
0.0916
0.0977
NSP: 0.0895 ± 0.0095
0.0846
0.0898
0.0962
-0.050
0.000
0.050
0.100
0.150
0.200

Proportion of Alanine

Histogram

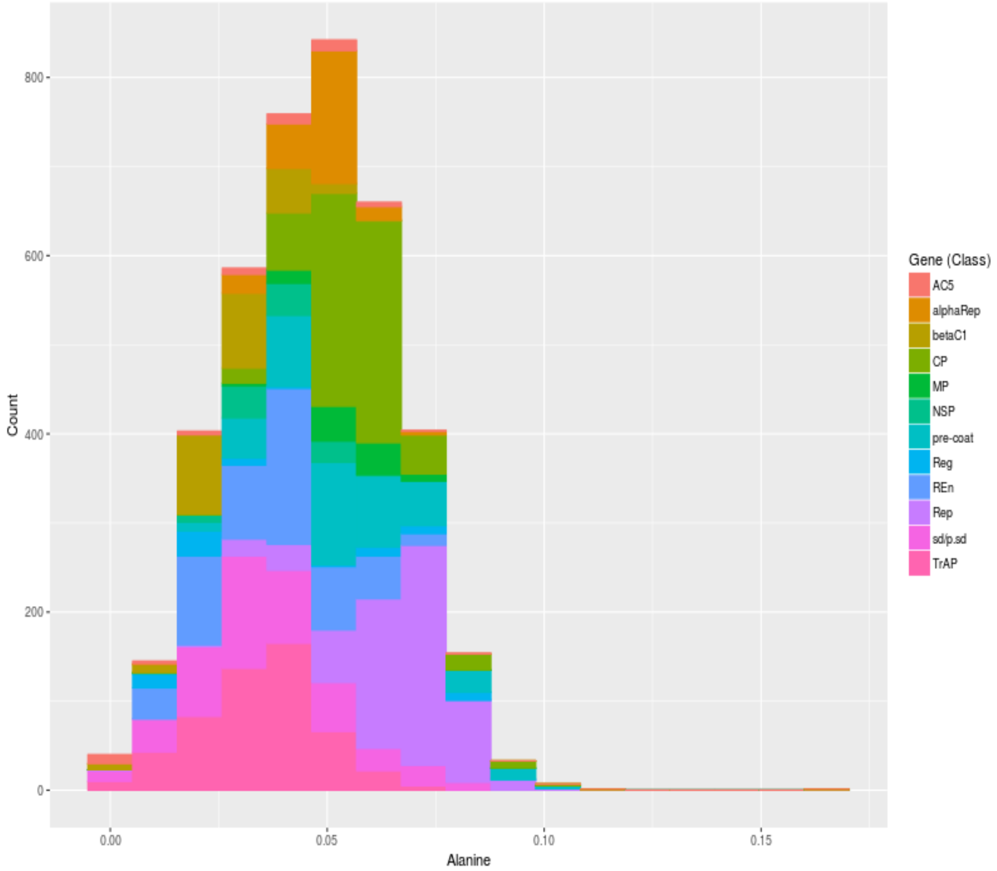

Density

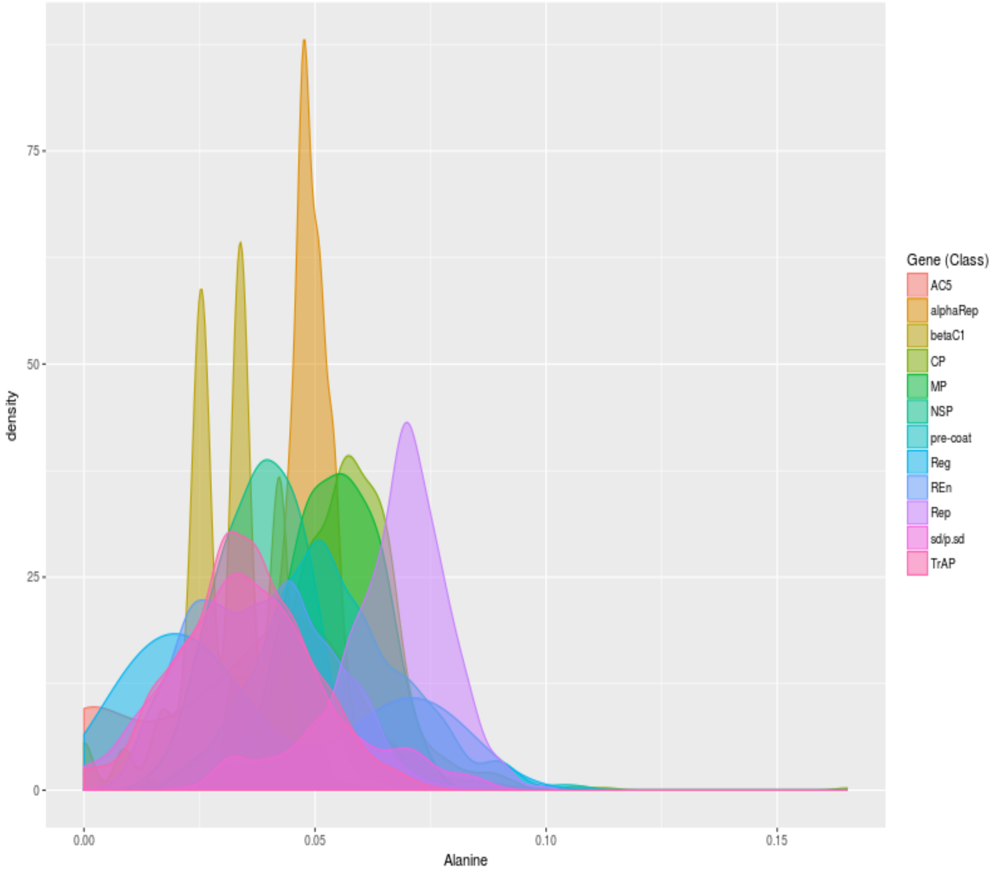

Boxplots

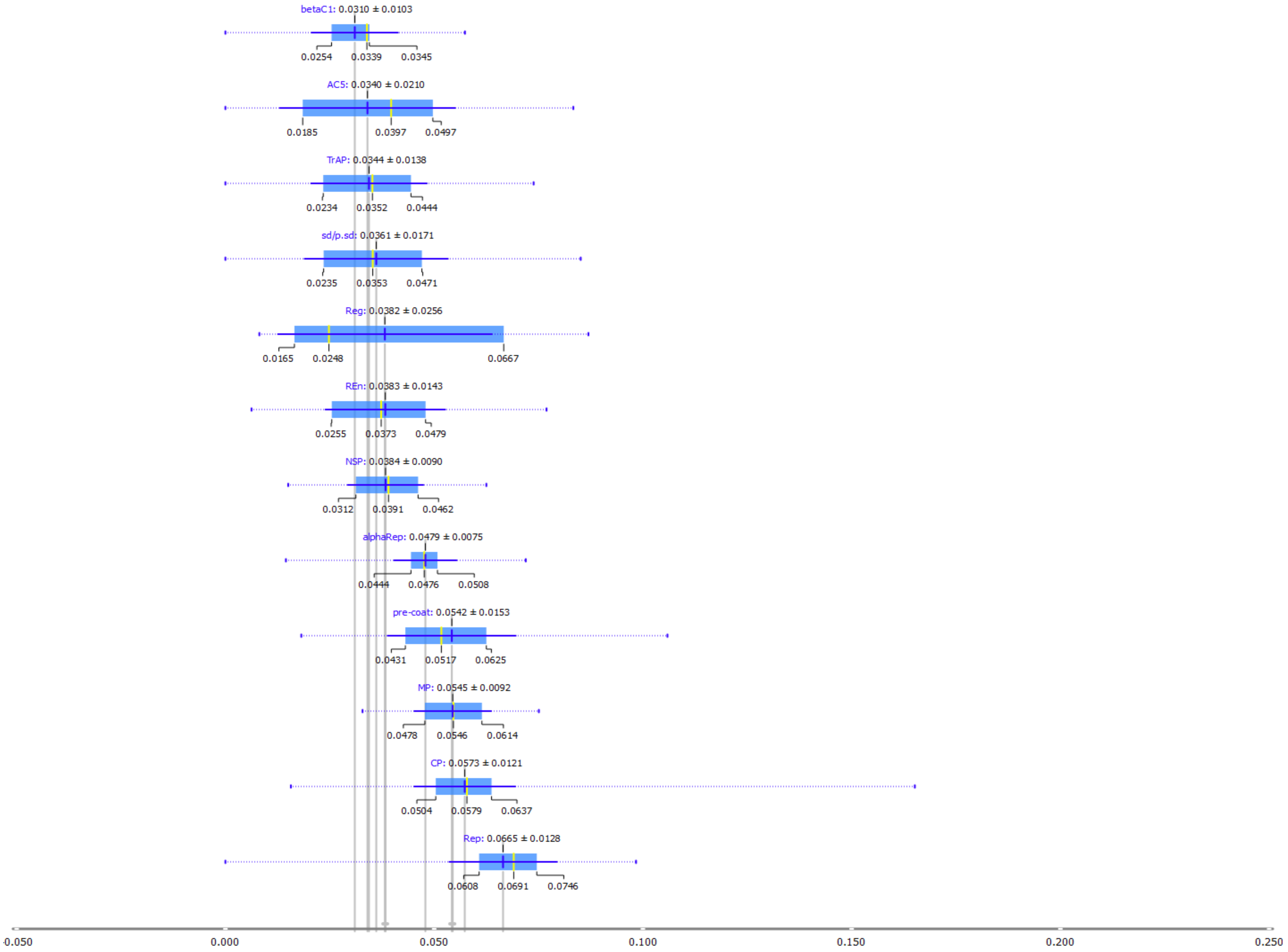

betaC1: 0.0310 ± 0.0103
0.0254
0.0339
0.0345
AC5: 0.0340 ± 0.0210
0.0185
0.0397
0.0497
TrAP: 0.0344 ± 0.0138
0.0234
0.0352
0.0444
sd/p.sd: 0.0361 ± 0.0171
0.0235
0.0353
0.0471
Reg: 0.0382 ± 0.0256
0.0165
0.0248
0.0667
REn: 0.0383 ± 0.0143
0.0255
0.0373
0.0479
NSP: 0.0384 ± 0.0090
0.0312
0.0391
0.0462
alphaRep: 0.0479 ± 0.0075
0.0444
0.0476
0.0508
pre-coat: 0.0542 ± 0.0153
0.0431
0.0517
0.0625
MP: 0.0545 ± 0.0092
0.0478
0.0546
0.0614
CP: 0.0573 ± 0.0121
0.0504
0.0579
0.0637
Rep: 0.0665 ± 0.0128
0.0608
0.0691
0.0746
-0.050
0.000
0.050
0.100
0.150
0.200
0.250

Proportion of Asparagine

Histogram

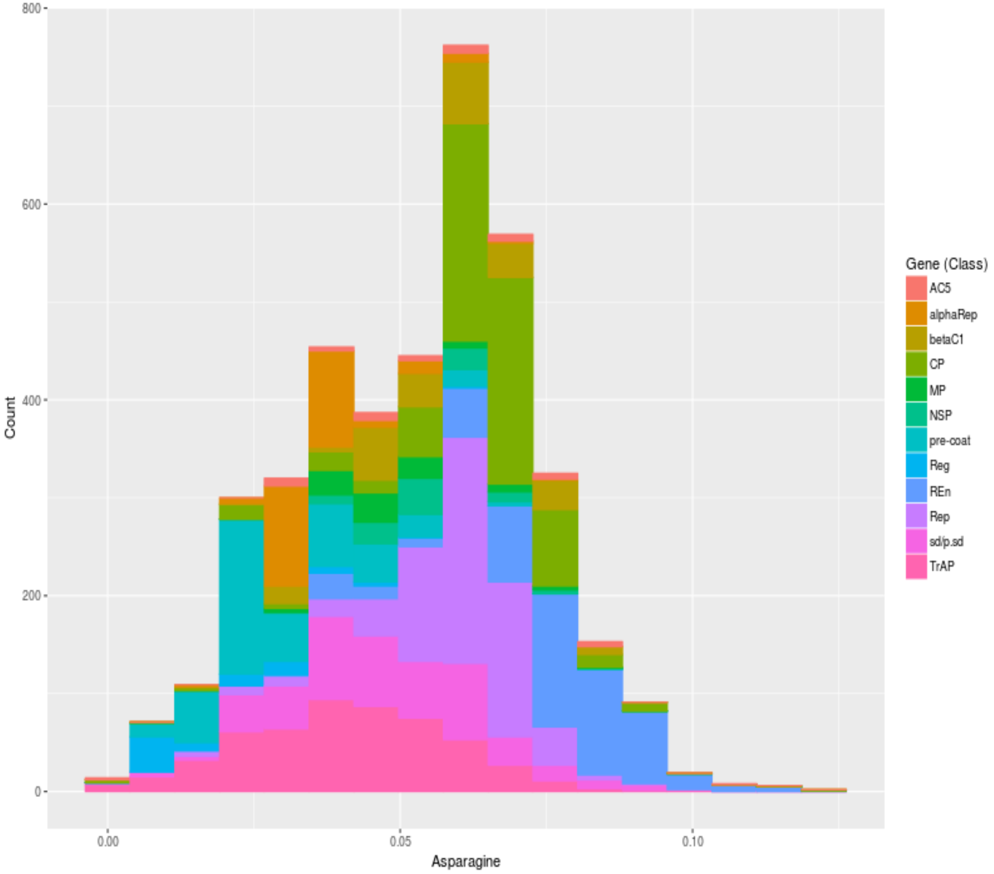

Density

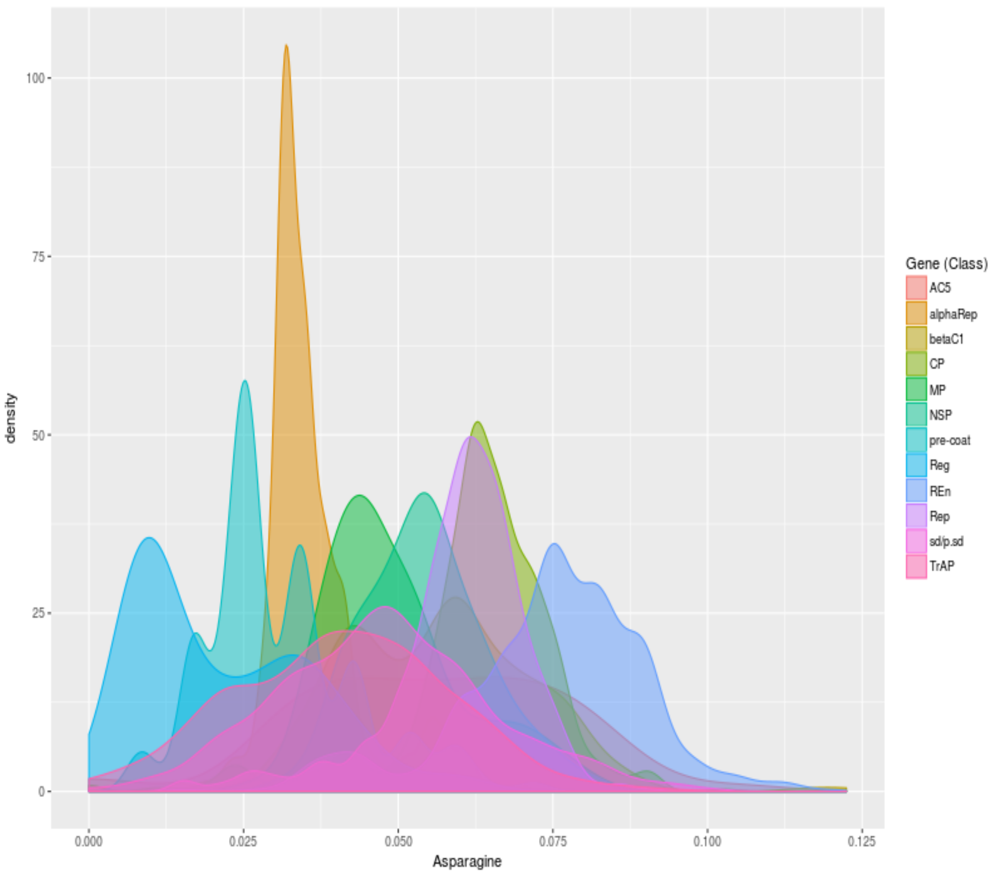

Boxplots

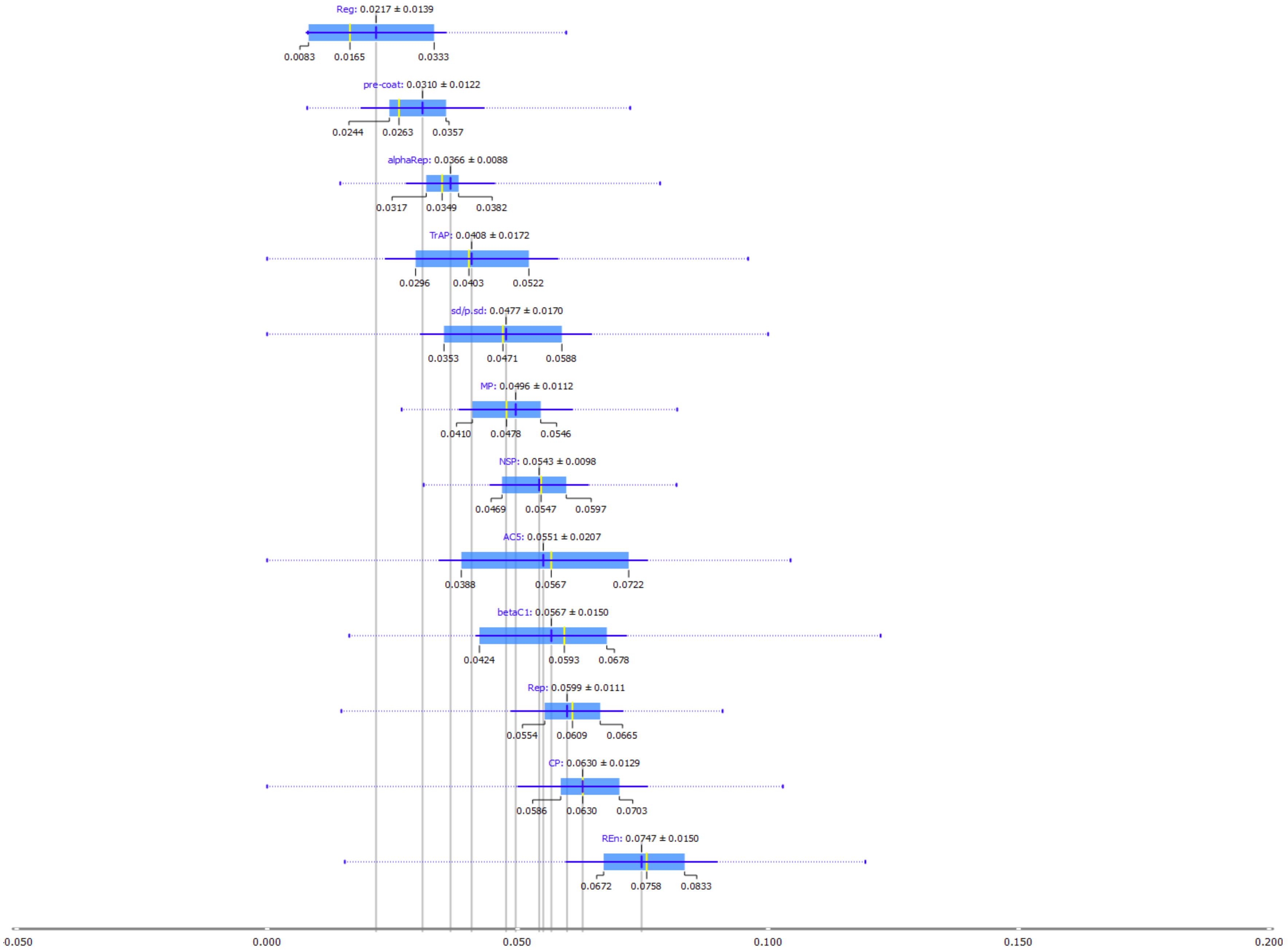

Reg: 0.0217 ± 0.0139
0.0083
0.0165
0.0333
pre-coat: 0.0310 ± 0.0122
0.0244
0.0263
0.0357
alphaRep: 0.0366 ± 0.0088
0.0317
0.0349
0.0382
TrAP: 0.0408 ± 0.0172
0.0296
0.0403
0.0522
sd/p.sd: 0.0477 ± 0.0170
0.0353
0.0471
0.0588
MP: 0.0496 ± 0.0112
0.0410
0.0478
0.0546
NSP: 0.0543 ± 0.0098
0.0469
0.0547
0.0597
AC5: 0.0551 ± 0.0207
0.0388
0.0567
0.0722
betaC1: 0.0567 ± 0.0150
0.0424
0.0593
0.0678
Rep: 0.0599 ± 0.0111
0.0554
0.0609
0.0665
CP: 0.0630 ± 0.0129
0.0586
0.0630
0.0703
REn: 0.0747 ± 0.0150
0.0672
0.0758
0.0833
-0.050
0.000
0.050
0.100
0.150
0.200

Proportion of Aspartic Acid

Histogram

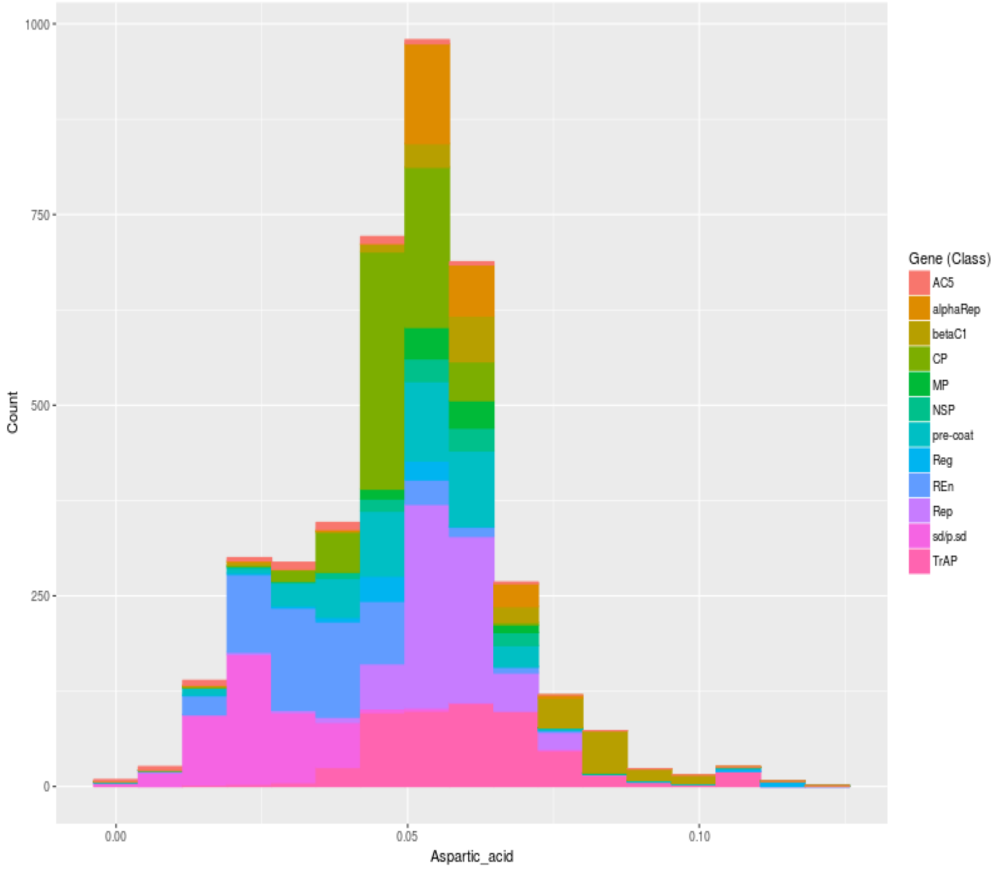

Density

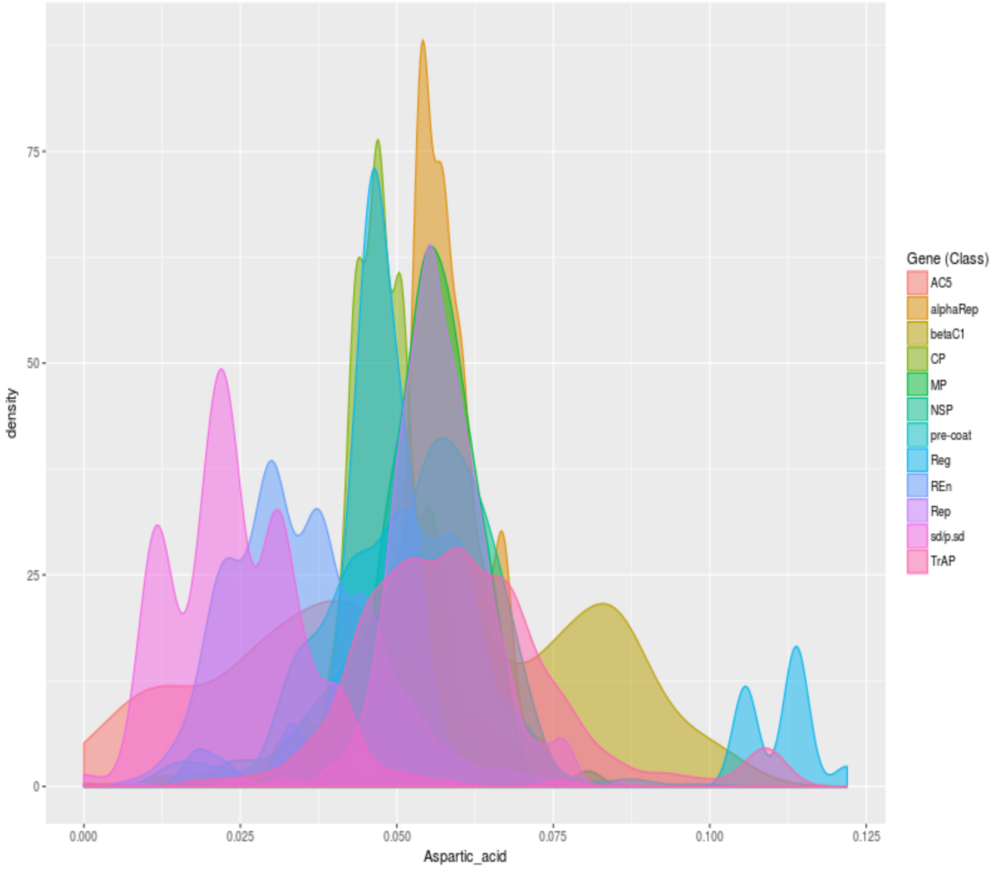

Boxplots

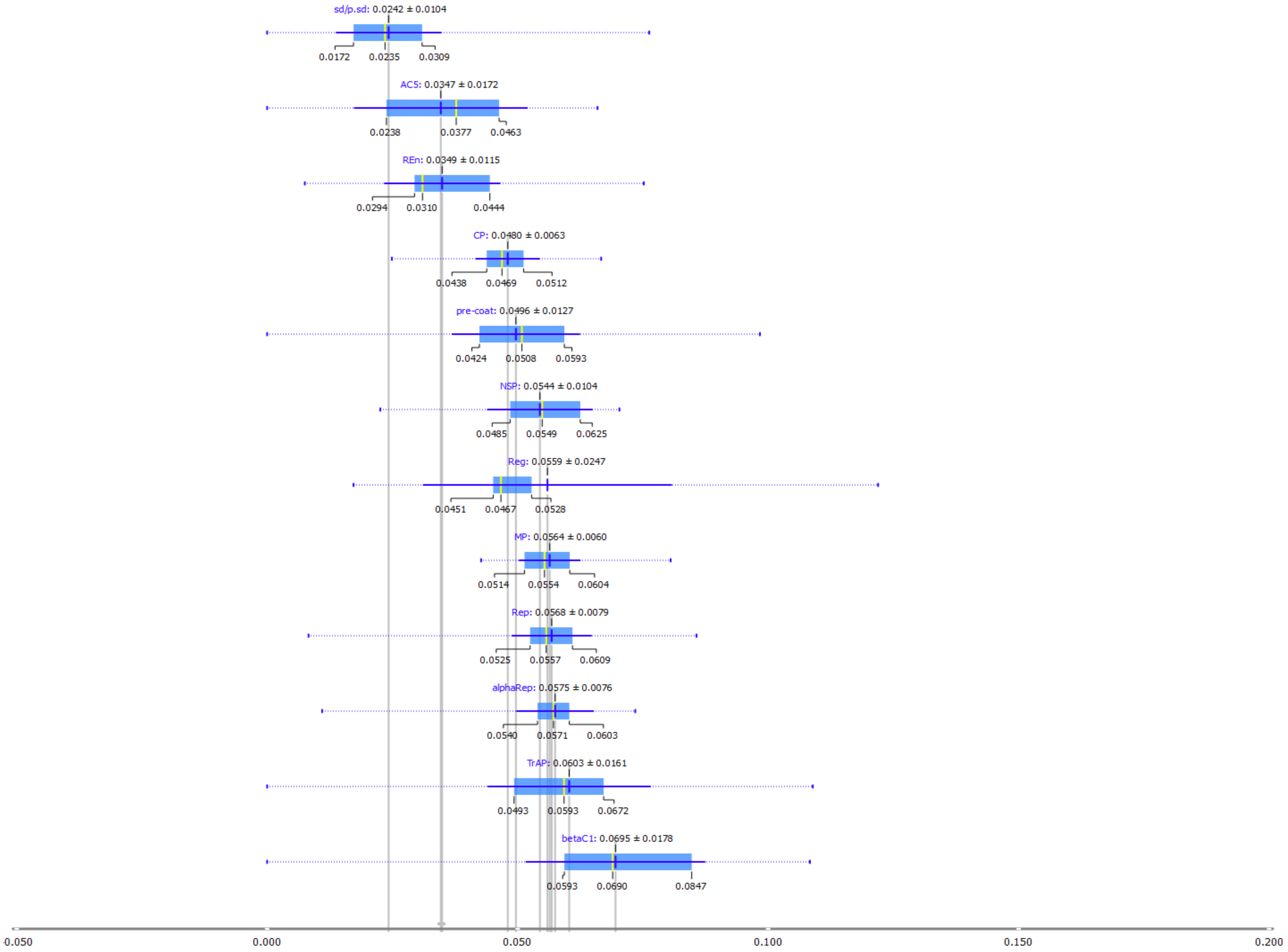

sd/p.sd: 0.0242 ± 0.0104
0.0172 0.0235 0.0309
AC5: 0.0347 ± 0.0172
0.0238 0.0377 0.0463
REn: 0.0349 ± 0.0115
0.0294 0.0310 0.0444
CP: 0.0480 ± 0.0063
0.0438 0.0469 0.0512
pre-coat: 0.0496 ± 0.0127
0.0424 0.0508 0.0593
NSP: 0.0544 ± 0.0104
0.0485 0.0549 0.0625
Reg: 0.0559 ± 0.0247
0.0451 0.0467 0.0528
MP: 0.0564 ± 0.0060
0.0514 0.0554 0.0604
Rep: 0.0568 ± 0.0079
0.0525 0.0557 0.0609
alphaRep: 0.0575 ± 0.0076
0.0540 0.0571 0.0603
TrAP: 0.0603 ± 0.0161
0.0493 0.0593 0.0672
betaC1: 0.0695 ± 0.0178
0.0593 0.0690 0.0847
-0.050 0.000 0.050 0.100 0.150 0.200

Proportion of Cysteine

Histogram

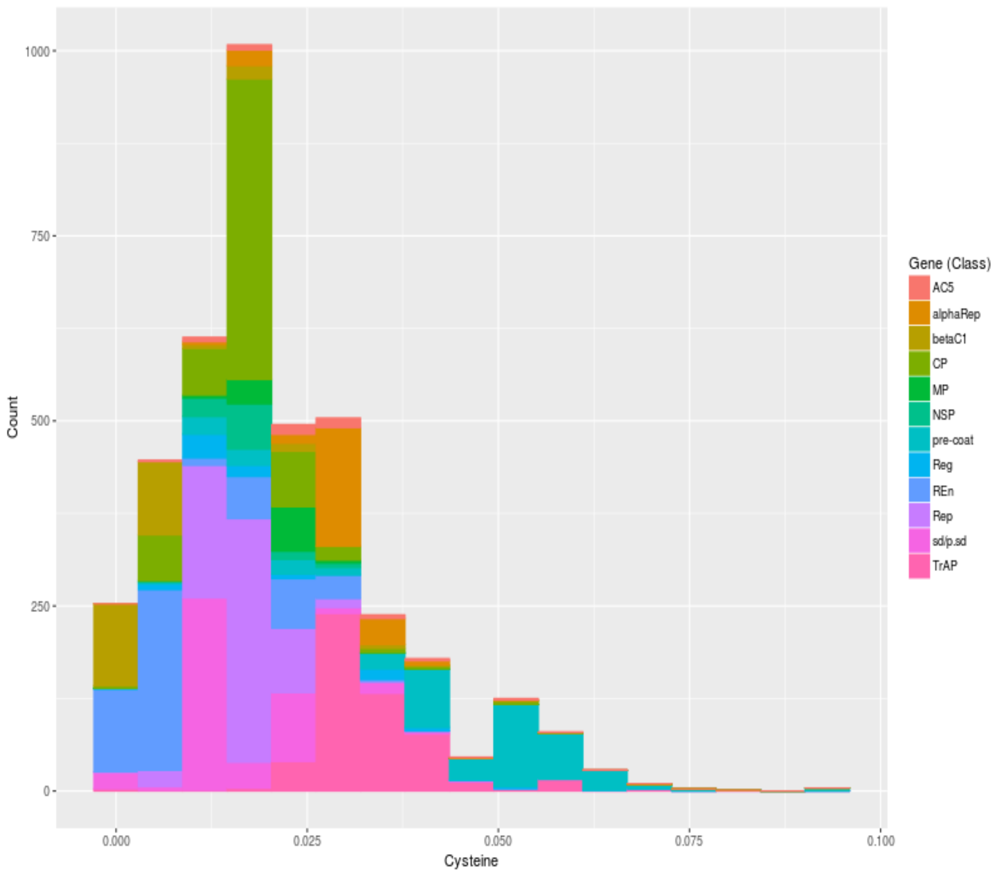

Density

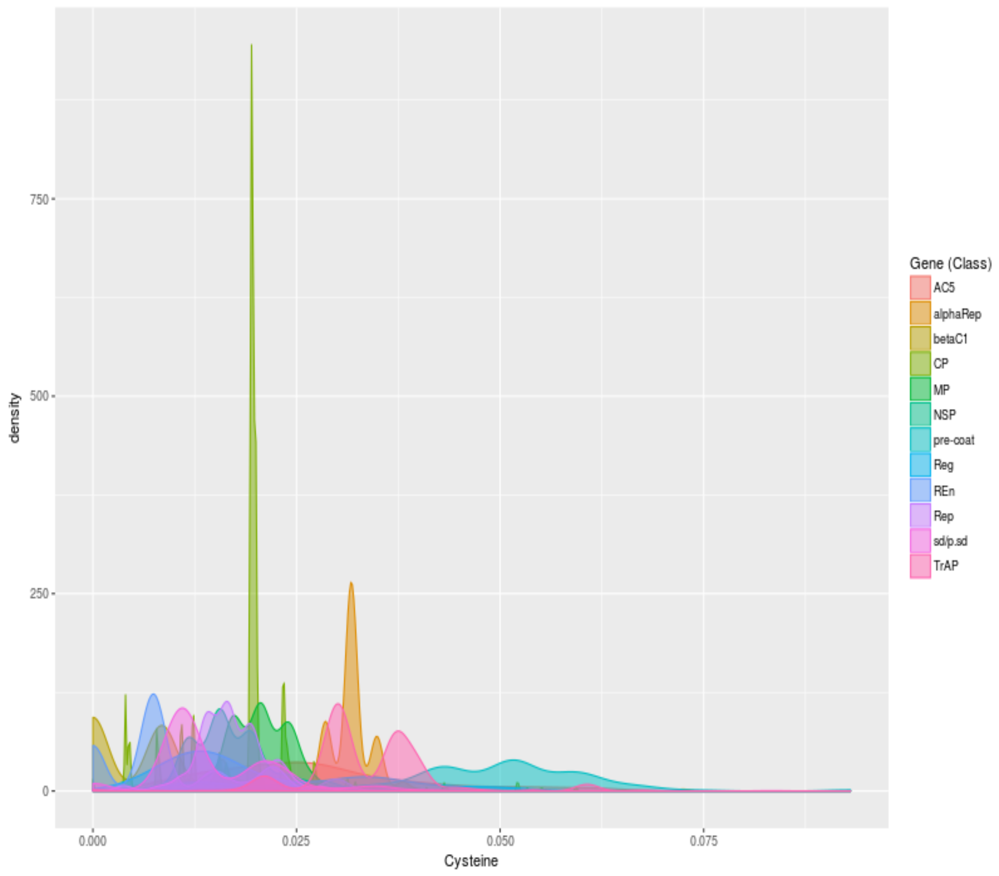

Boxplots

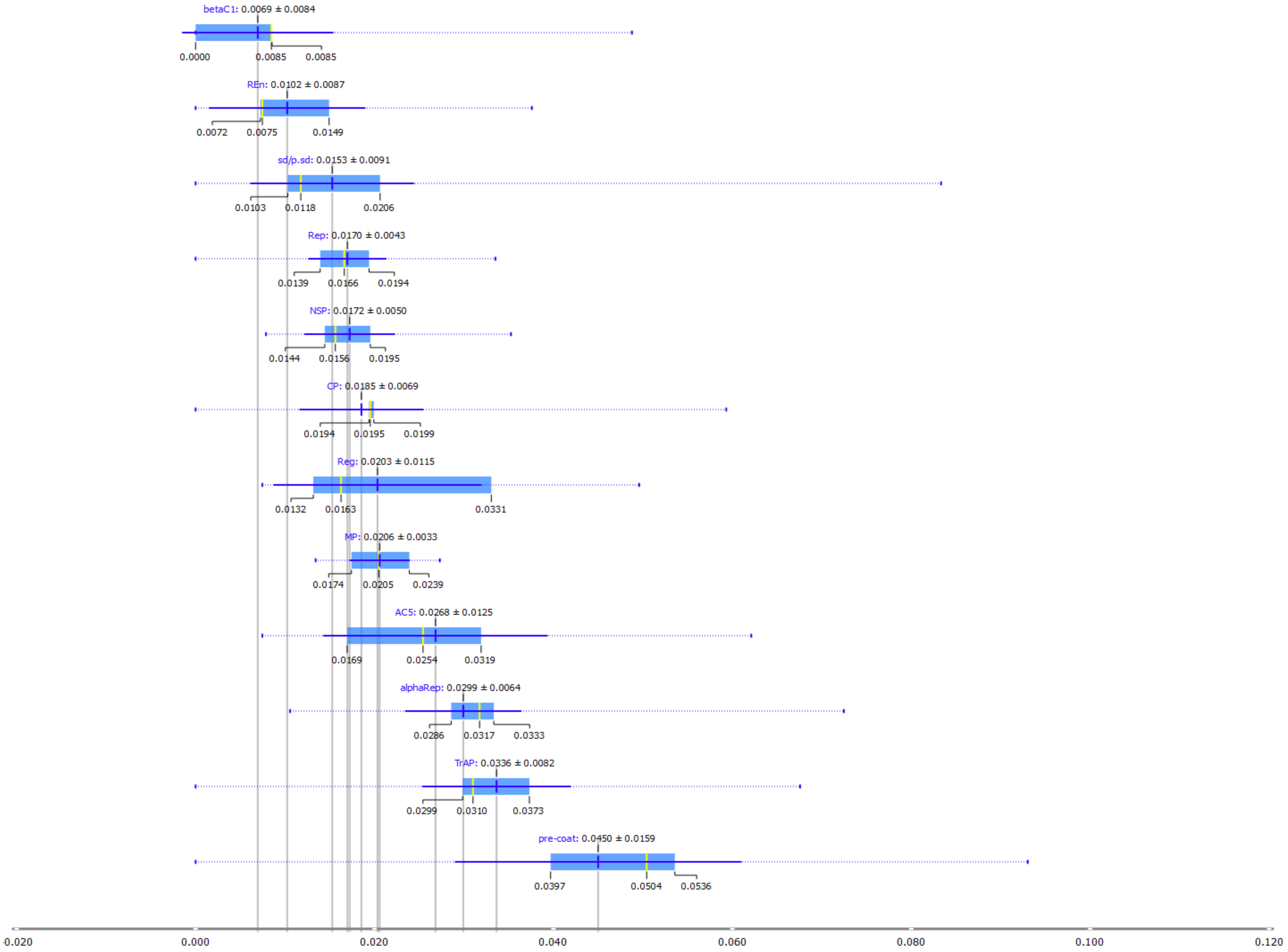

betaC1: 0.0069 ± 0.0084
0.0000
0.0085
0.0085
REn: 0.0102 ± 0.0087
0.0072
0.0075
0.0149
sd/p.sd: 0.0153 ± 0.0091
0.0103
0.0118
0.0206
Rep: 0.0170 ± 0.0043
0.0139
0.0166
0.0194
NSP: 0.0172 ± 0.0050
0.0144
0.0156
0.0195
CP: 0.0185 ± 0.0069
0.0194
0.0195
0.0199
Reg: 0.0203 ± 0.0115
0.0132
0.0163
0.0331
MP: 0.0206 ± 0.0033
0.0174
0.0205
0.0239
AC5: 0.0268 ± 0.0125
0.0169
0.0254
0.0319
alphaRep: 0.0299 ± 0.0064
0.0286
0.0317
0.0333
TrAP: 0.0336 ± 0.0082
0.0299
0.0310
0.0373
pre-coat: 0.0450 ± 0.0159
0.0397
0.0504
0.0536
-0.020
0.000
0.020
0.040
0.060
0.080
0.100
0.120

Proportion of Glutamic Acid

Histogram

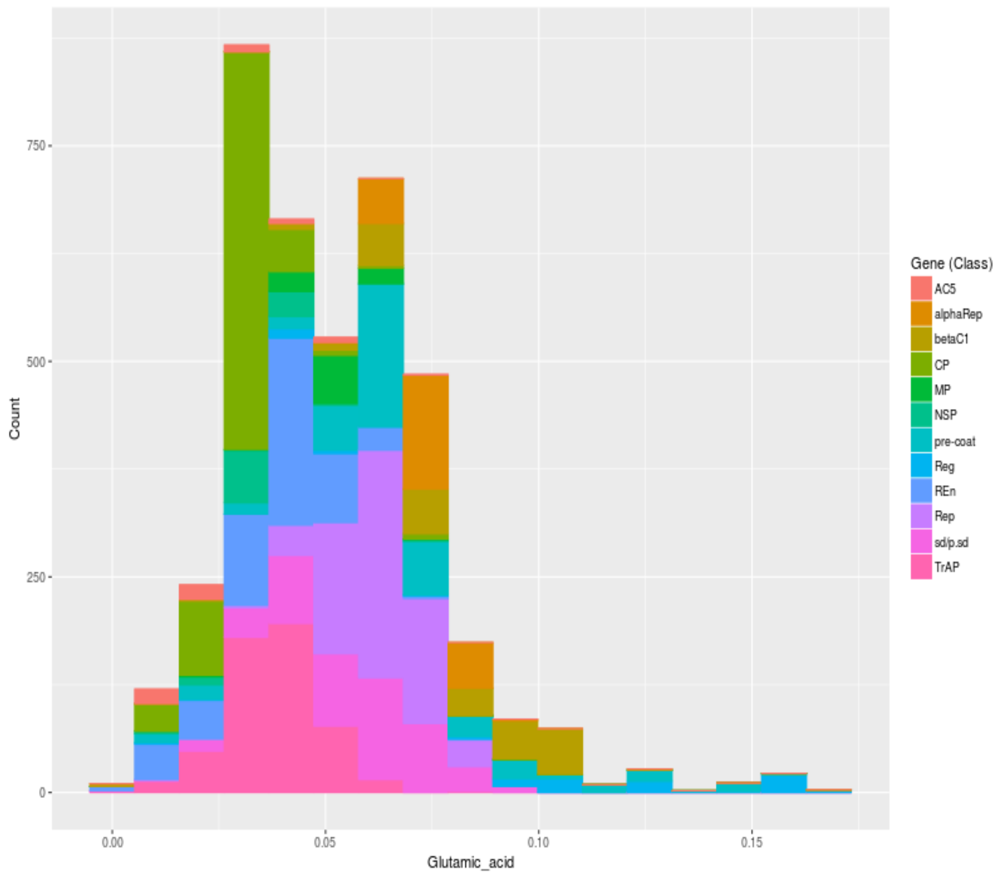

Density

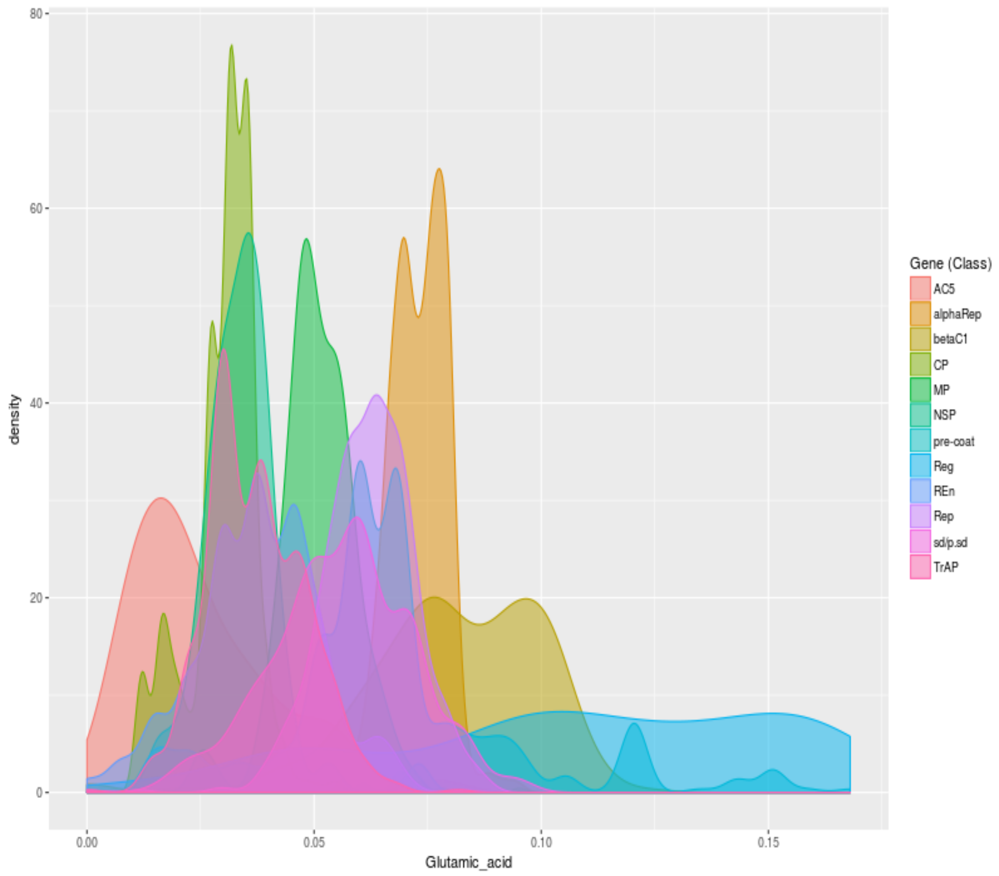

Boxplots

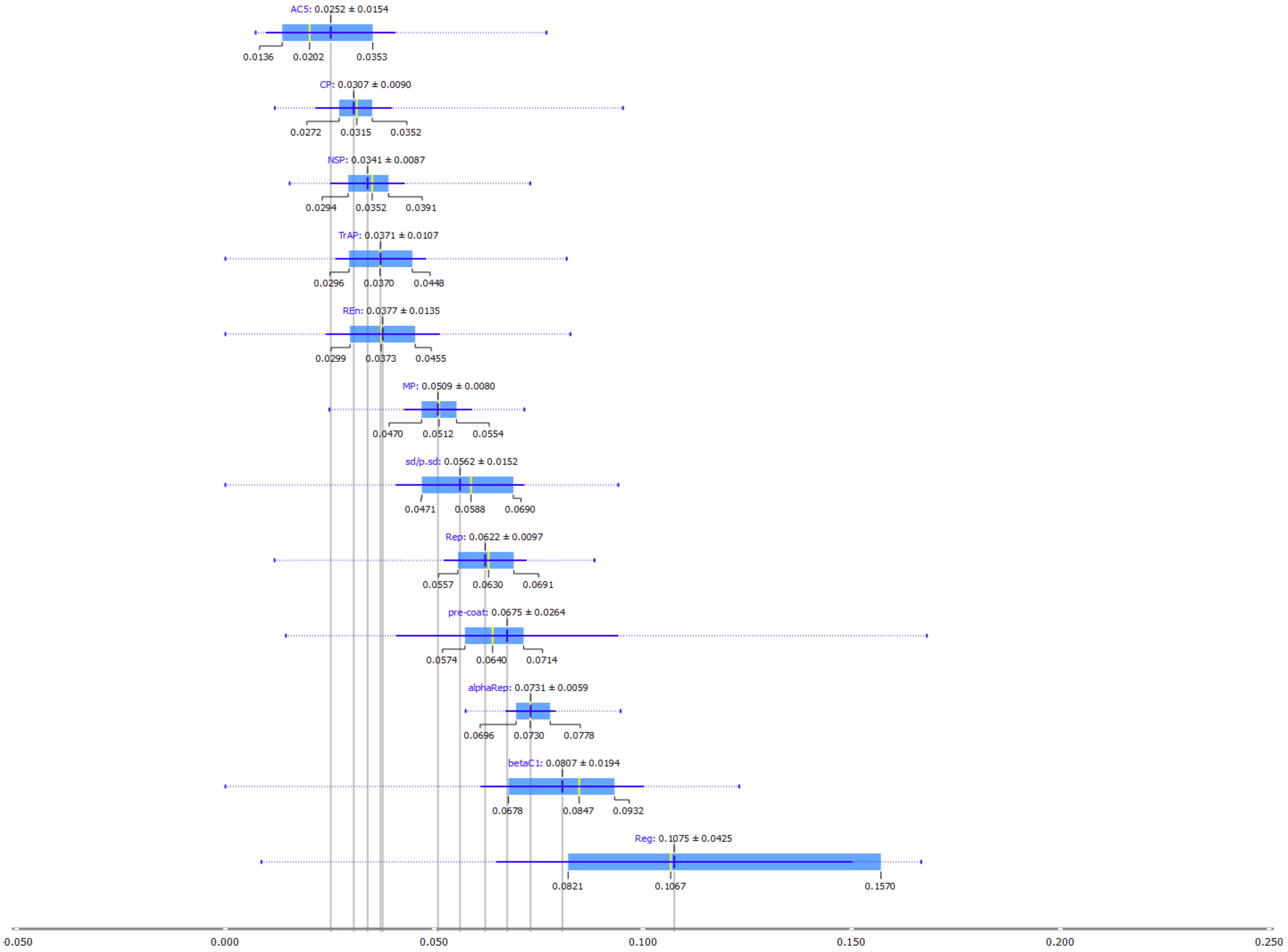

AC5: 0.0252 ± 0.0154
0.0136
0.0202
0.0353
CP: 0.0307 ± 0.0090
0.0272
0.0315
0.0352
NSP: 0.0341 ± 0.0087
0.0294
0.0352
0.0391
TrAP: 0.0371 ± 0.0107
0.0296
0.0370
0.0448
REn: 0.0377 ± 0.0135
0.0299
0.0373
0.0455
MP: 0.0509 ± 0.0080
0.0470
0.0512
0.0554
sd/p.sd: 0.0562 ± 0.0152
0.0471
0.0588
0.0690
Rep: 0.0622 ± 0.0097
0.0557
0.0630
0.0691
pre-coat: 0.0675 ± 0.0264
0.0574
0.0640
0.0714
alphaRep: 0.0731 ± 0.0059
0.0696
0.0730
0.0778
betaC1: 0.0807 ± 0.0194
0.0678
0.0847
0.0932
Reg: 0.1075 ± 0.0425
0.0821
0.1067
0.1570
-0.050
0.000
0.050
0.100
0.150
0.200
0.250

Proportion of Glutamine

Histogram

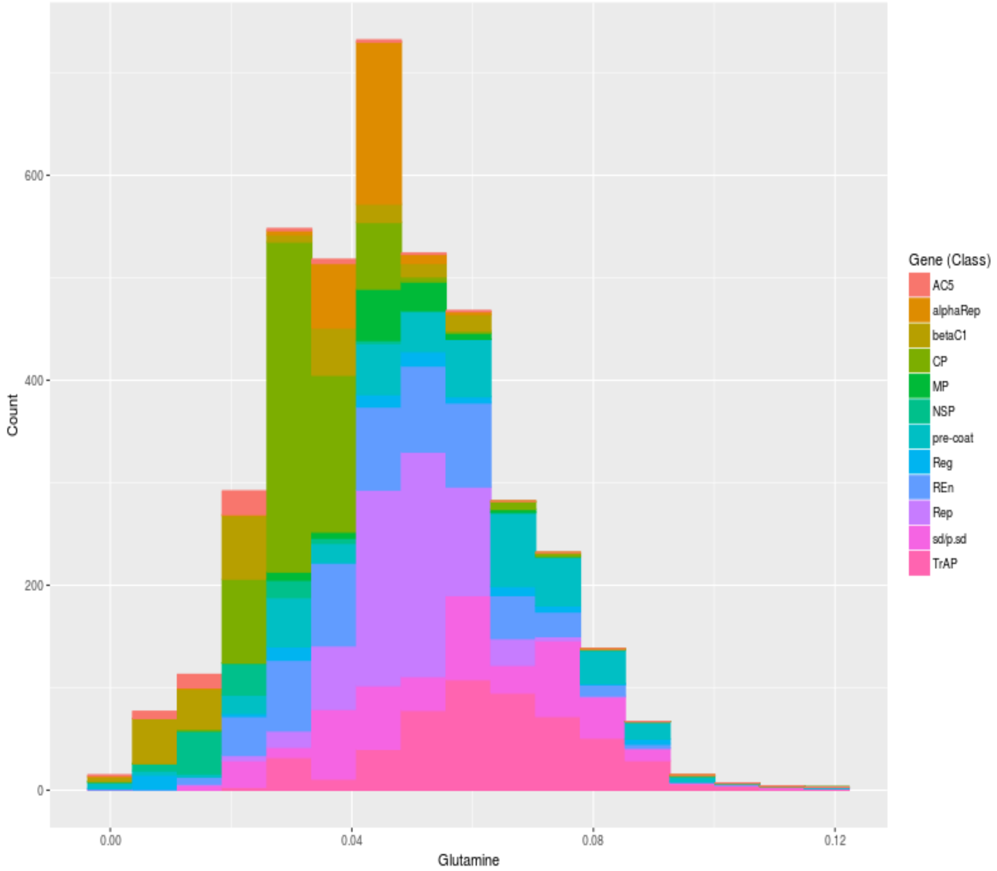

Density

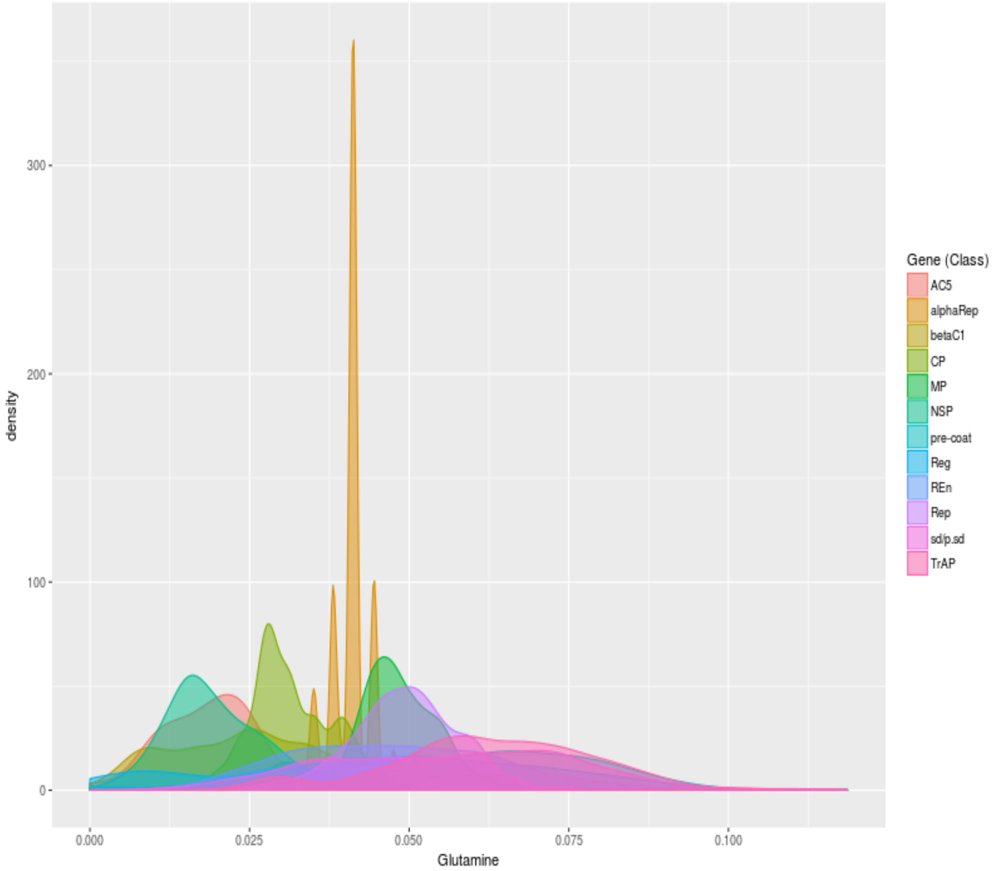

Boxplots

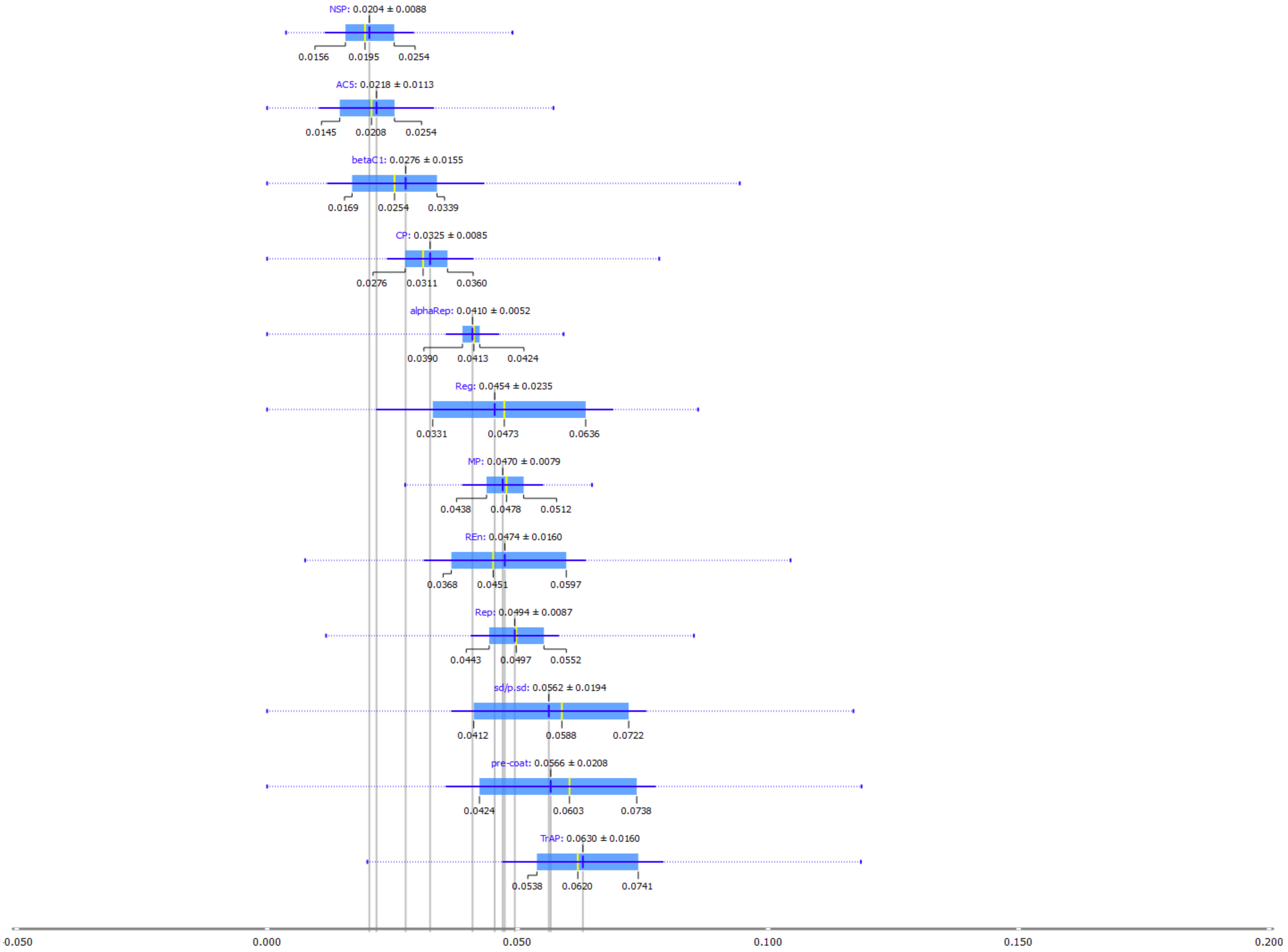

NSP: 0.0204 ± 0.0088
0.0156
0.0195
0.0254
AC5: 0.0218 ± 0.0113
0.0145
0.0208
0.0254
betaC1: 0.0276 ± 0.0155
0.0169
0.0254
0.0339
CP: 0.0325 ± 0.0085
0.0276
0.0311
0.0360
alphaRep: 0.0410 ± 0.0052
0.0390
0.0413
0.0424
Reg: 0.0454 ± 0.0235
0.0331
0.0473
0.0636
MP: 0.0470 ± 0.0079
0.0438
0.0478
0.0512
REn: 0.0474 ± 0.0160
0.0368
0.0451
0.0597
Rep: 0.0494 ± 0.0087
0.0443
0.0497
0.0552
sd/p.sd: 0.0562 ± 0.0194
0.0412
0.0588
0.0722
pre-coat: 0.0566 ± 0.0208
0.0424
0.0603
0.0738
TrAP: 0.0630 ± 0.0160
0.0538
0.0620
0.0741
-0.050
0.000
0.050
0.100
0.150
0.200

Proportion of Glycine

Histogram

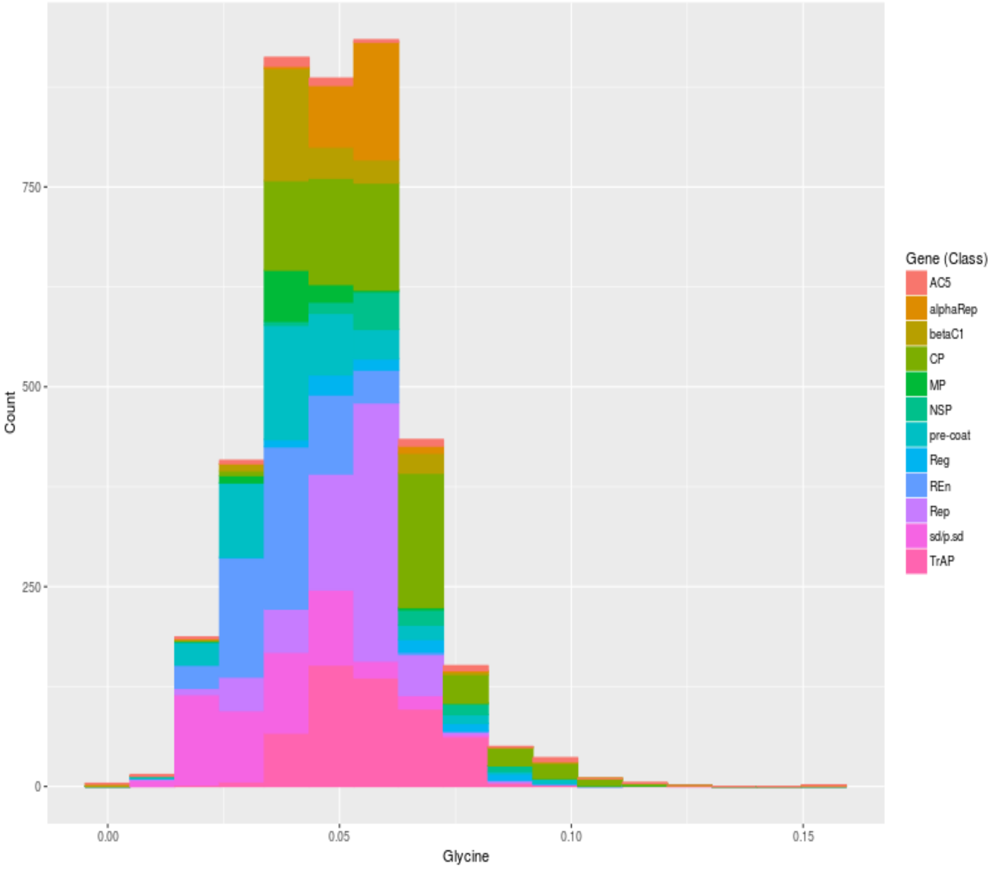

Density

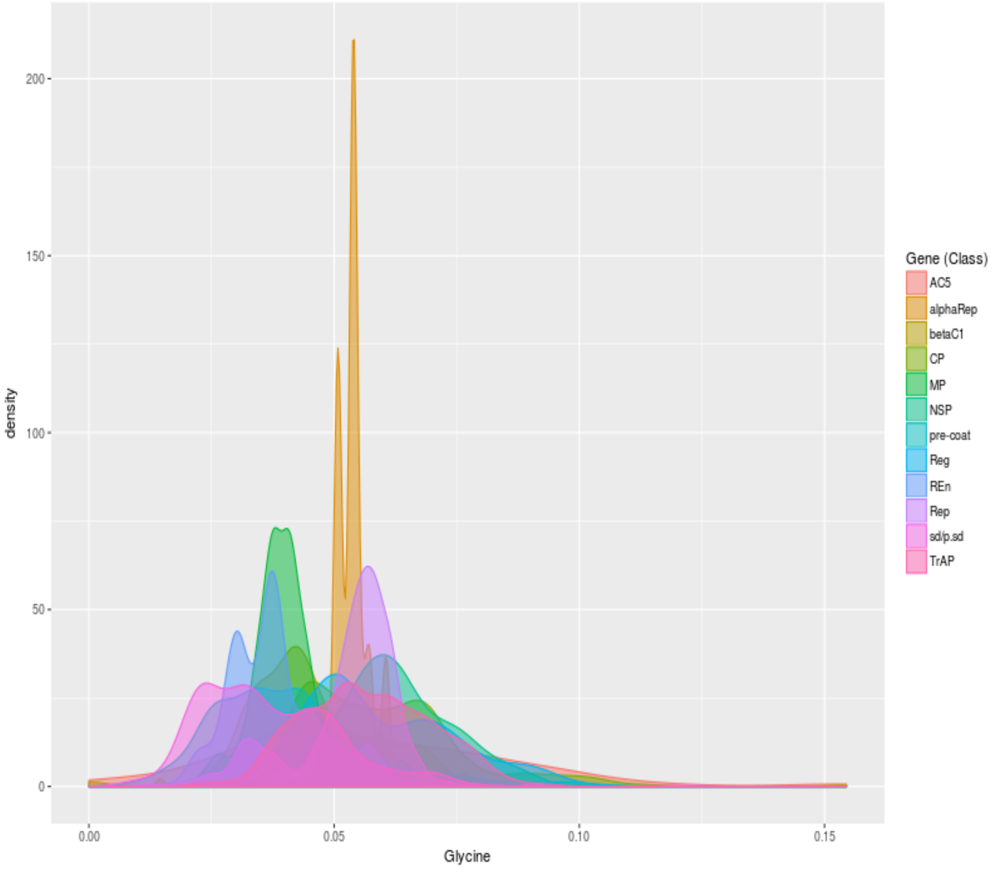

Boxplots

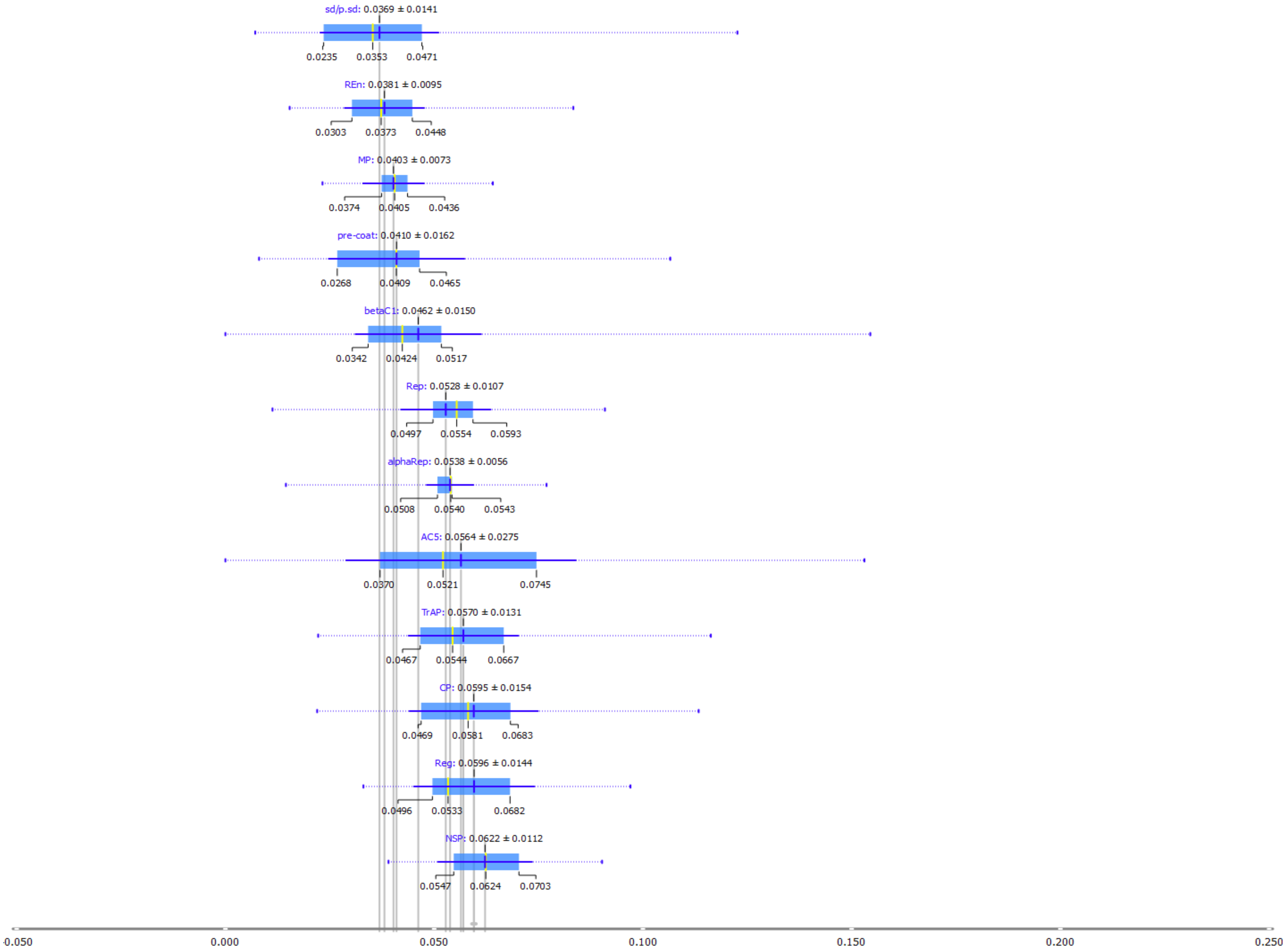

sd/p.sd: 0.0369 ± 0.0141
0.0235 0.0353 0.0471
REn: 0.0381 ± 0.0095
0.0303 0.0373 0.0448
MP: 0.0403 ± 0.0073
0.0374 0.0405 0.0436
pre-coat: 0.0410 ± 0.0162
0.0268 0.0409 0.0465
betaC1: 0.0462 ± 0.0150
0.0342 0.0424 0.0517
Rep: 0.0528 ± 0.0107
0.0497 0.0554 0.0593
alphaRep: 0.0538 ± 0.0056
0.0508 0.0540 0.0543
AC5: 0.0564 ± 0.0275
0.0370 0.0521 0.0745
TrAP: 0.0570 ± 0.0131
0.0467 0.0544 0.0667
CP: 0.0595 ± 0.0154
0.0469 0.0581 0.0683
Reg: 0.0596 ± 0.0144
0.0496 0.0533 0.0682
NSP: 0.0622 ± 0.0112
0.0547 0.0624 0.0703
-0.050
0.000
0.050
0.100
0.150
0.200
0.250

Proportion of Histidine

Histogram

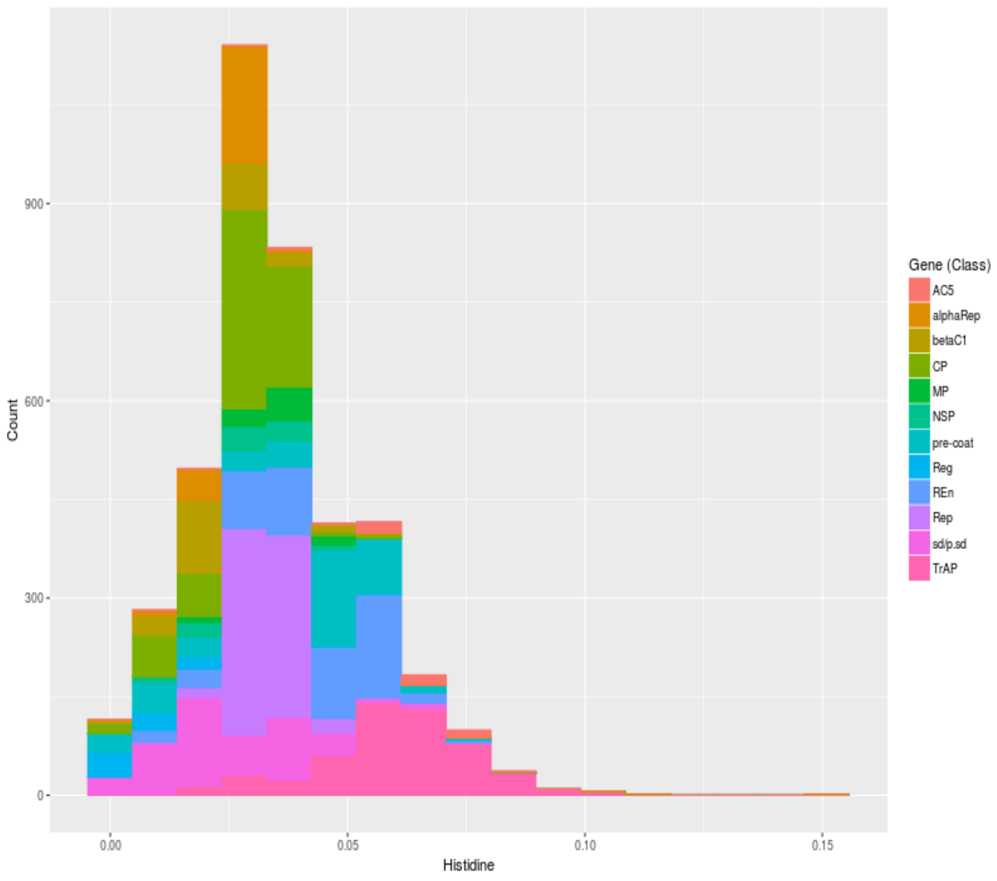

Density

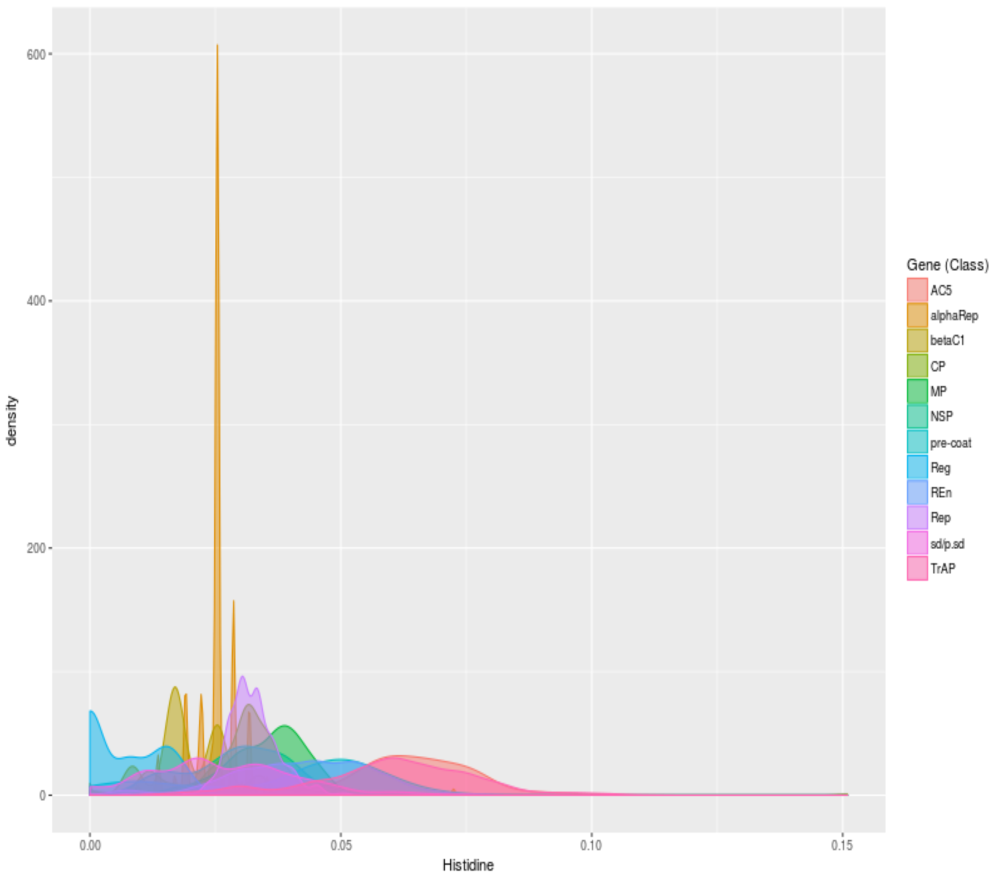

Boxplots

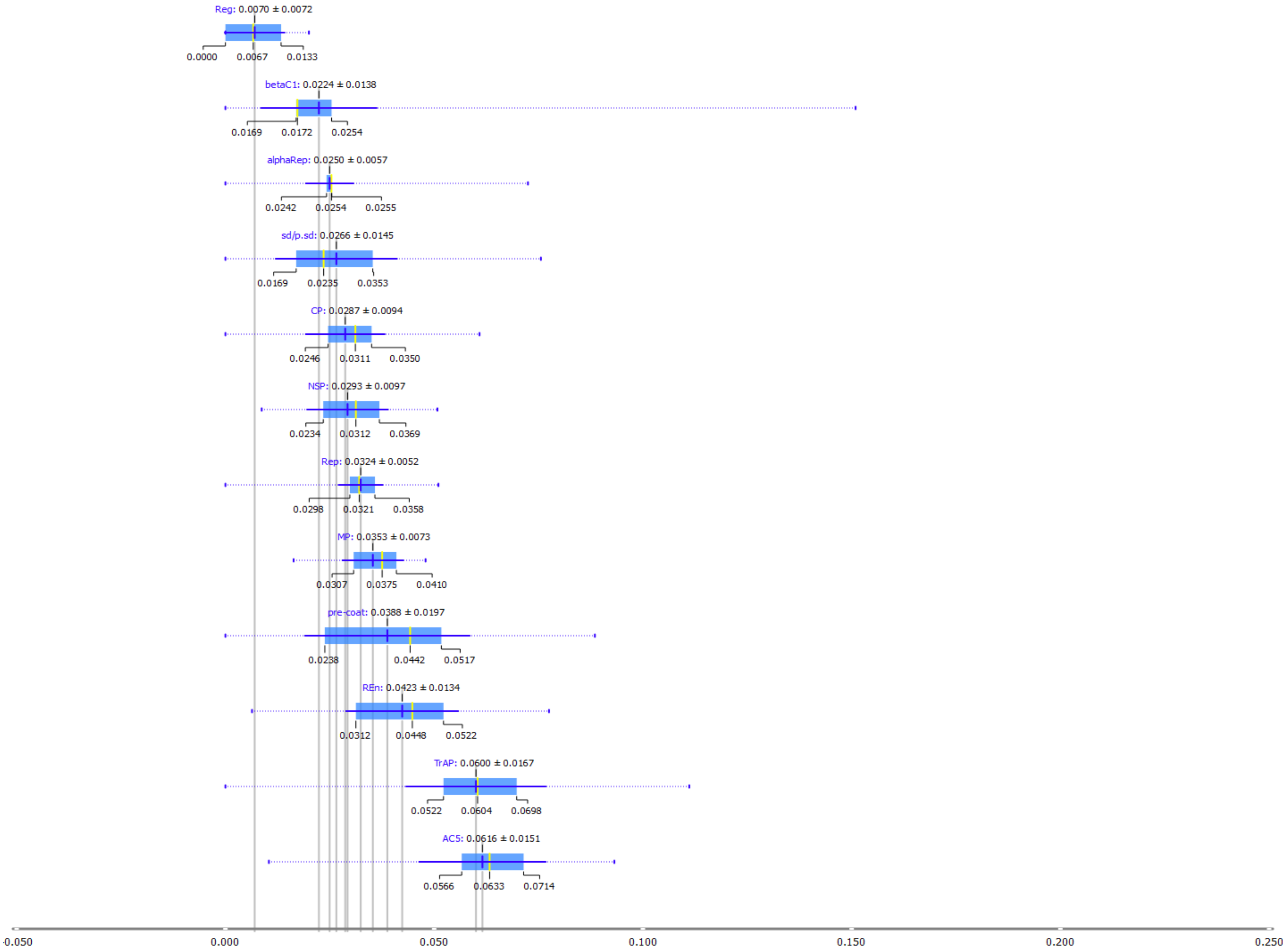

Reg: 0.0070 ± 0.0072
0.0000
0.0067
0.0133
betaC1: 0.0224 ± 0.0138
0.0169
0.0172
0.0254
alphaRep: 0.0250 ± 0.0057
0.0242
0.0254
0.0255
sd/p.sd: 0.0266 ± 0.0145
0.0169
0.0235
0.0353
CP: 0.0287 ± 0.0094
0.0246
0.0311
0.0350
NSP: 0.0293 ± 0.0097
0.0234
0.0312
0.0369
Rep: 0.0324 ± 0.0052
0.0298
0.0321
0.0358
MP: 0.0353 ± 0.0073
0.0307
0.0375
0.0410
pre-coat: 0.0388 ± 0.0197
0.0238
0.0442
0.0517
REn: 0.0423 ± 0.0134
0.0312
0.0448
0.0522
TrAP: 0.0600 ± 0.0167
0.0522
0.0604
0.0698
AC5: 0.0616 ± 0.0151
0.0566
0.0633
0.0714
-0.050
0.000
0.050
0.100
0.150
0.200
0.250

Proportion of Isoleucine

Histogram

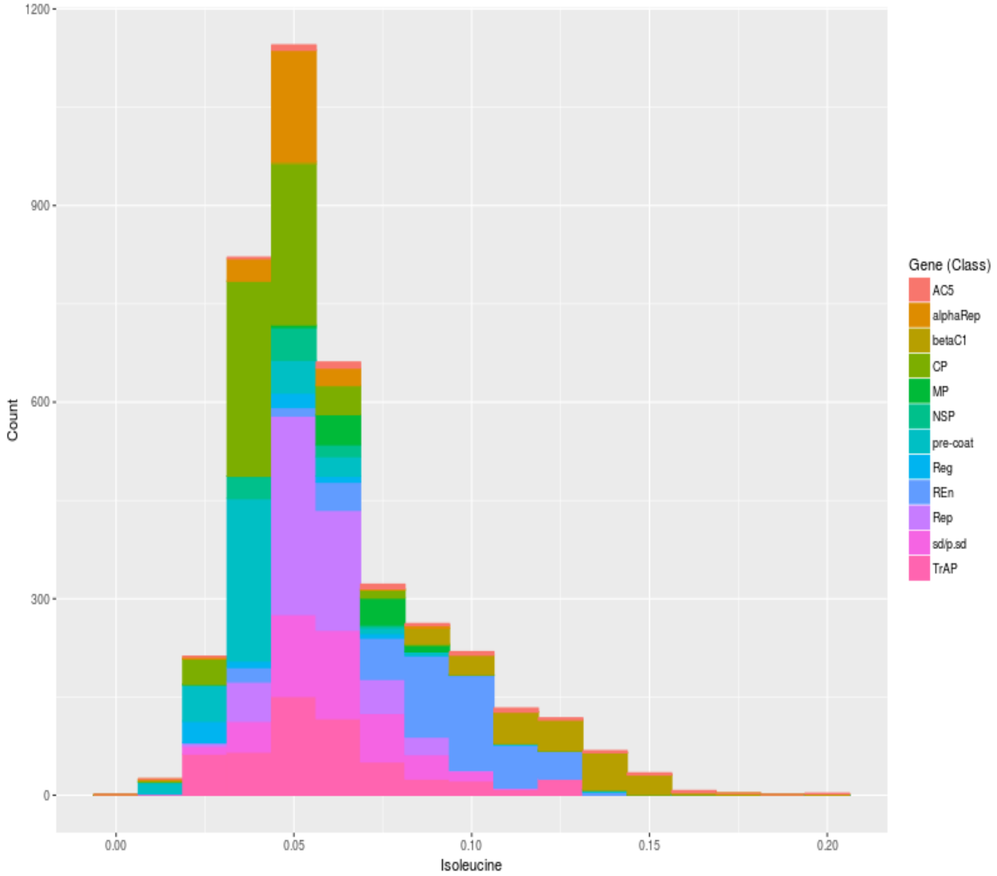

Density

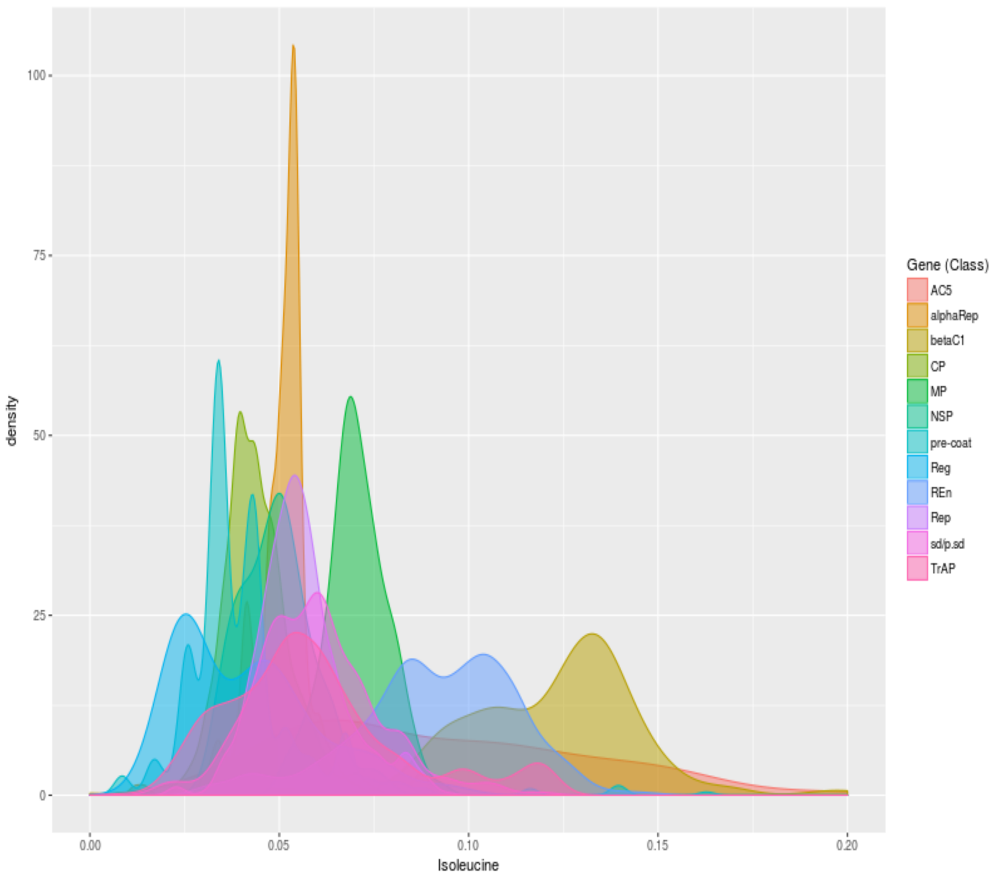

Boxplots

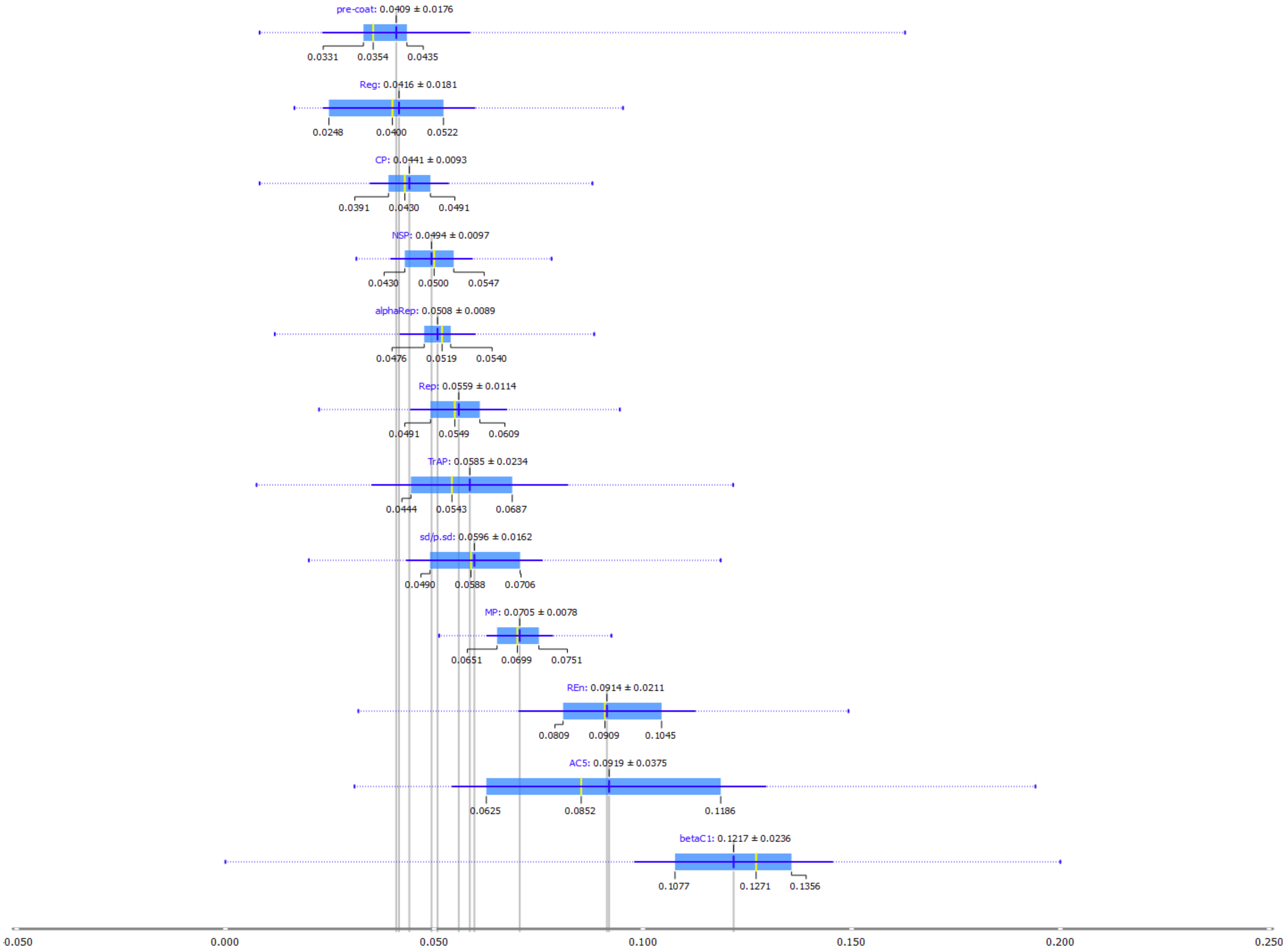

pre-coat: 0.0409 ± 0.0176
0.0331 0.0354 0.0435
Reg: 0.0416 ± 0.0181
0.0248 0.0400 0.0522
CP: 0.0441 ± 0.0093
0.0391 0.0430 0.0491
NSP: 0.0494 ± 0.0097
0.0430 0.0500 0.0547
alphaRep: 0.0508 ± 0.0089
0.0476 0.0519 0.0540
Rep: 0.0559 ± 0.0114
0.0491 0.0549 0.0609
TrAP: 0.0585 ± 0.0234
0.0444 0.0543 0.0687
sd/p.sd: 0.0596 ± 0.0162
0.0490 0.0588 0.0706
MP: 0.0705 ± 0.0078
0.0651 0.0699 0.0751
REn: 0.0914 ± 0.0211
0.0809 0.0909 0.1045
AC5: 0.0919 ± 0.0375
0.0625 0.0852 0.1186
betaC1: 0.1217 ± 0.0236
0.1077 0.1271 0.1356
-0.050 0.000 0.050 0.100 0.150 0.200 0.250

Length

Histogram

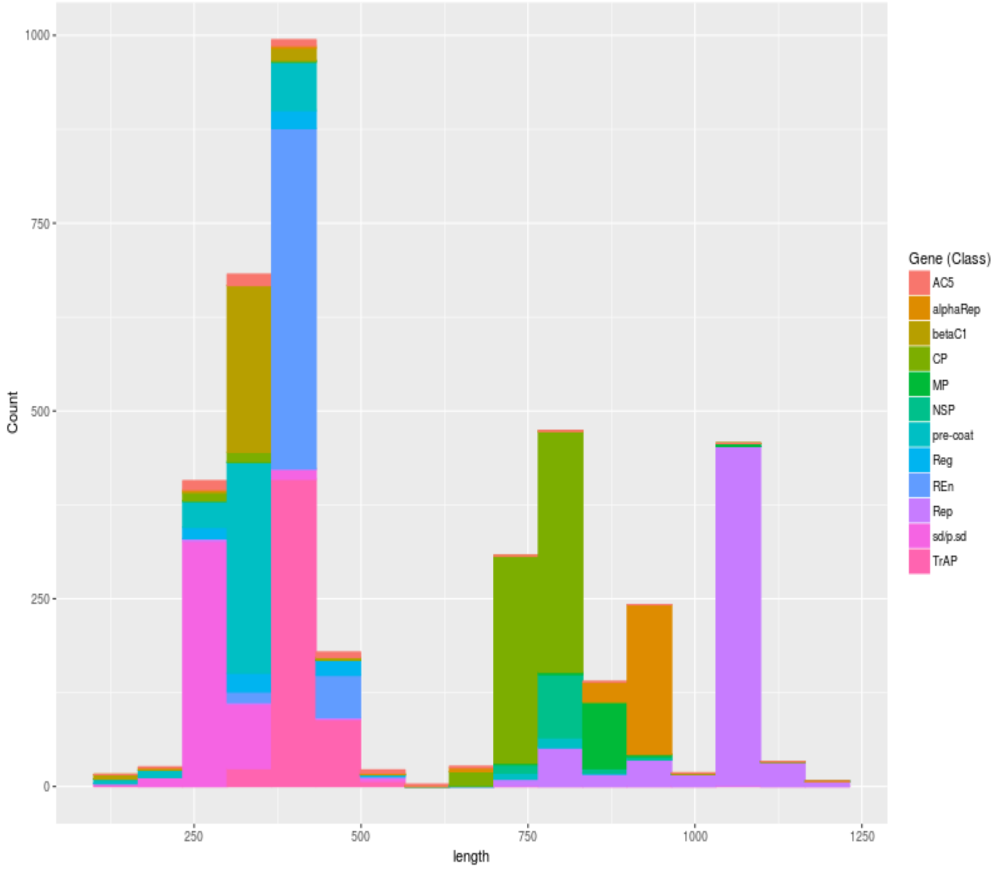

Density

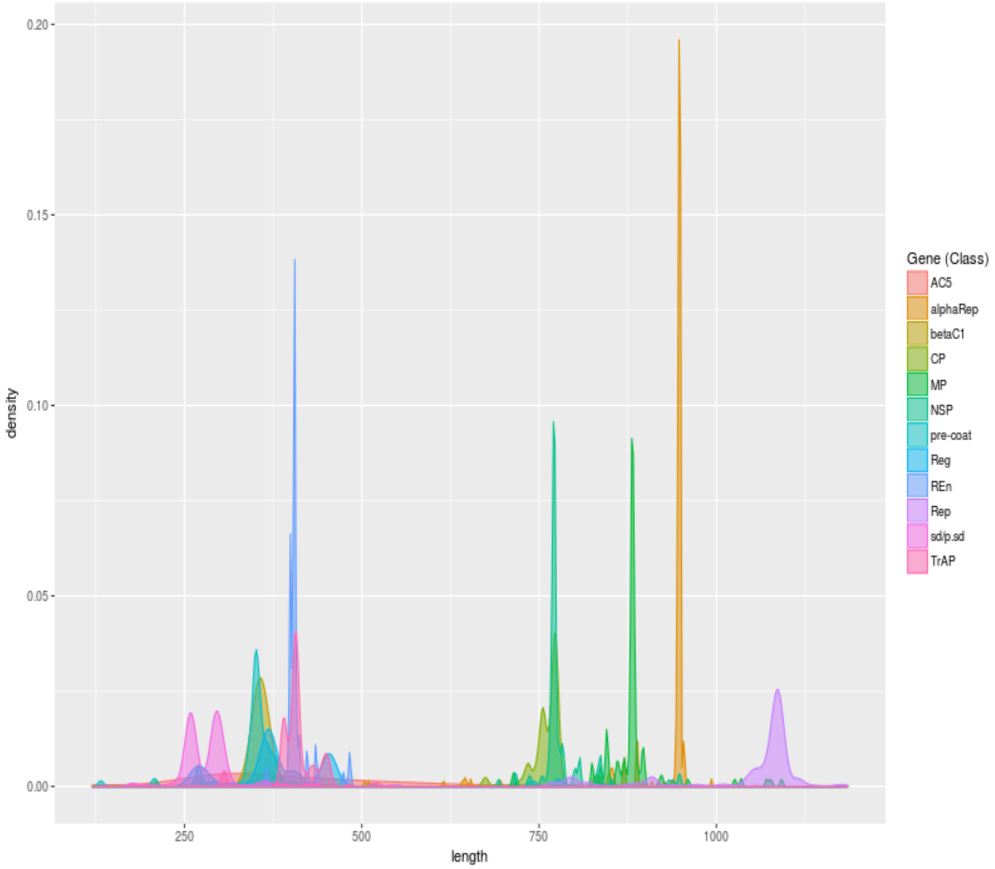

Boxplots

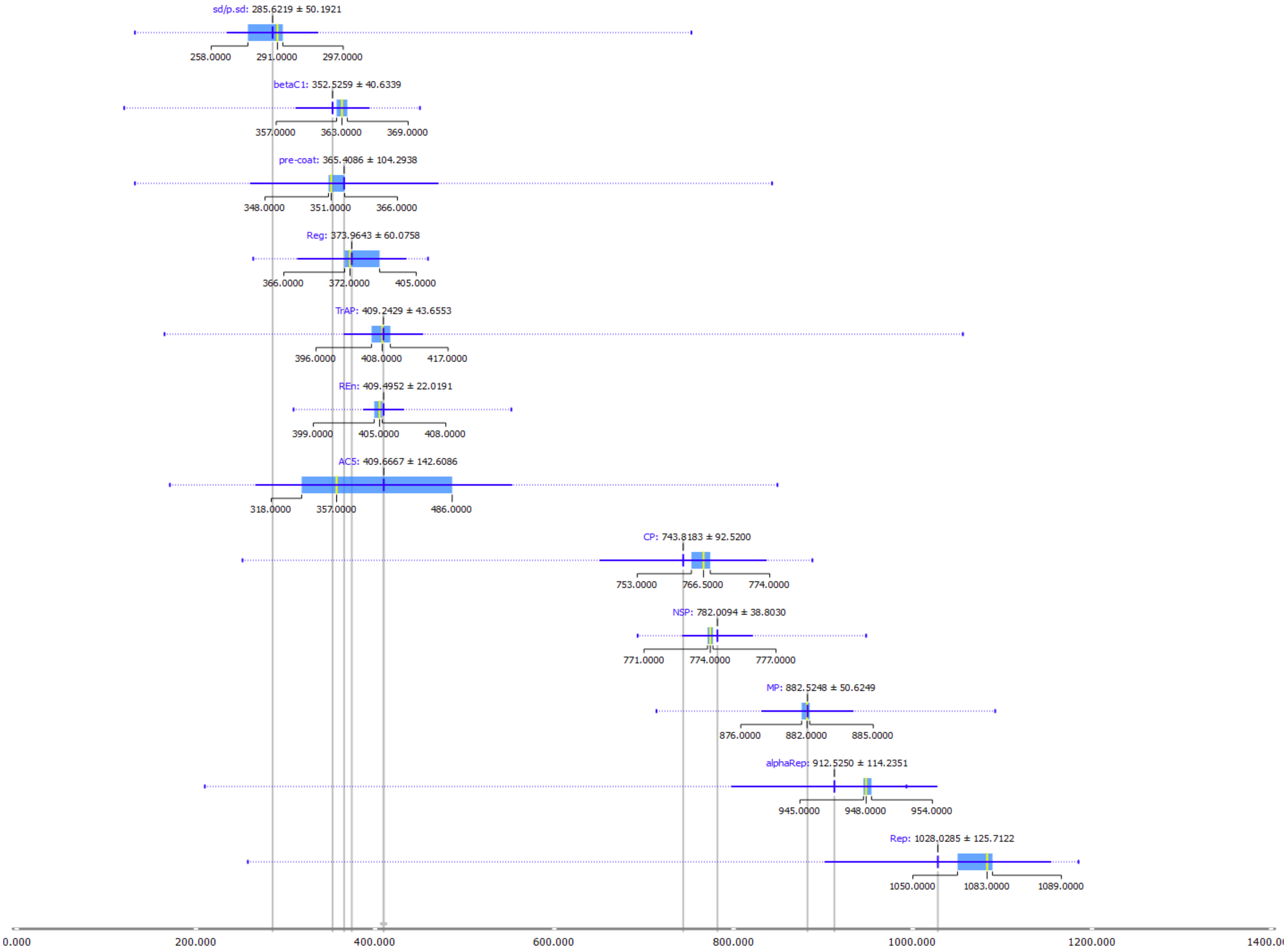

sd/p.sd: 285.6219 ± 50.1921
258.0000
291.0000
297.0000
betaC1: 352.5259 ± 40.6339
357.0000
363.0000
369.0000
pre-coat: 365.4086 ± 104.2938
348.0000
351.0000
366.0000
Reg: 373.9643 ± 60.0758
366.0000
372.0000
405.0000
TrAP: 409.2429 ± 43.6553
396.0000
408.0000
417.0000
REn: 409.4952 ± 22.0191
399.0000
405.0000
408.0000
AC5: 409.6667 ± 142.6086
318.0000
357.0000
486.0000
CP: 743.8183 ± 92.5200
753.0000
766.5000
774.0000
NSP: 782.0094 ± 38.8030
771.0000
774.0000
777.0000
MP: 882.5248 ± 50.6249
876.0000
882.0000
885.0000
alphaRep: 912.5250 ± 114.2351
945.0000
948.0000
954.0000
Rep: 1028.0285 ± 125.7122
1050.0000
1083.0000
1089.0000
0.000
200.000
400.000
600.000
800.000
1000.000
1200.000

Sense

Histogram

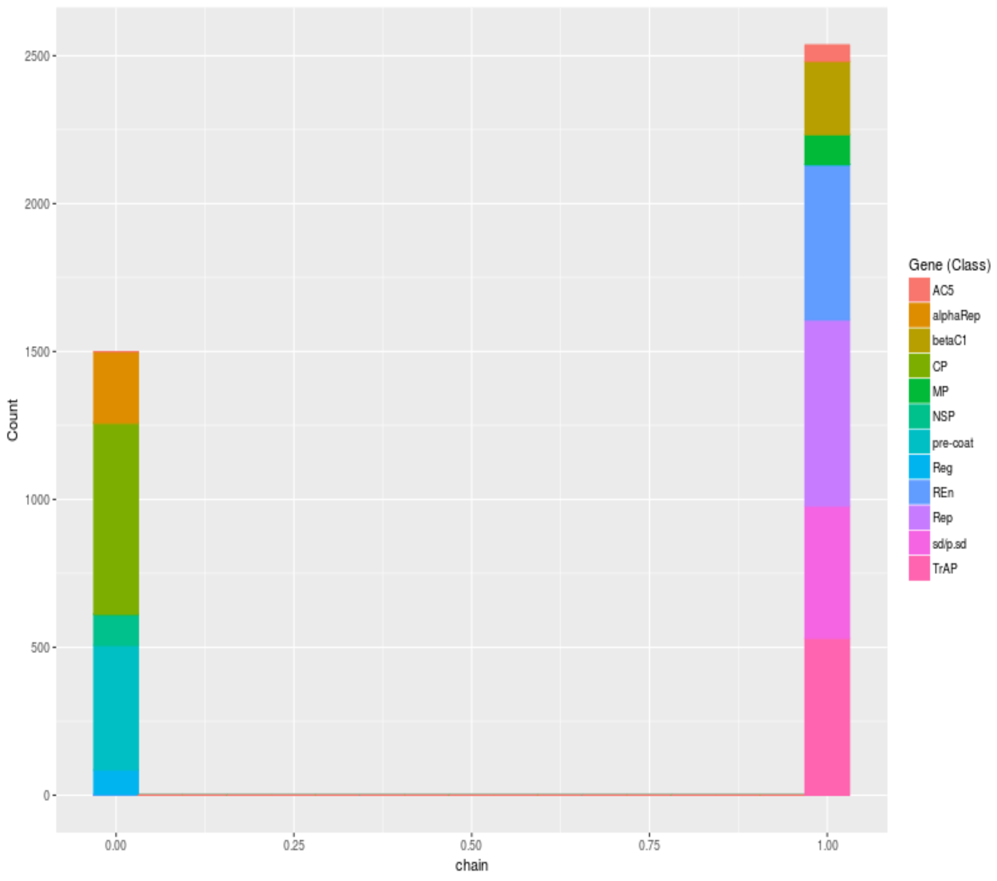

Density

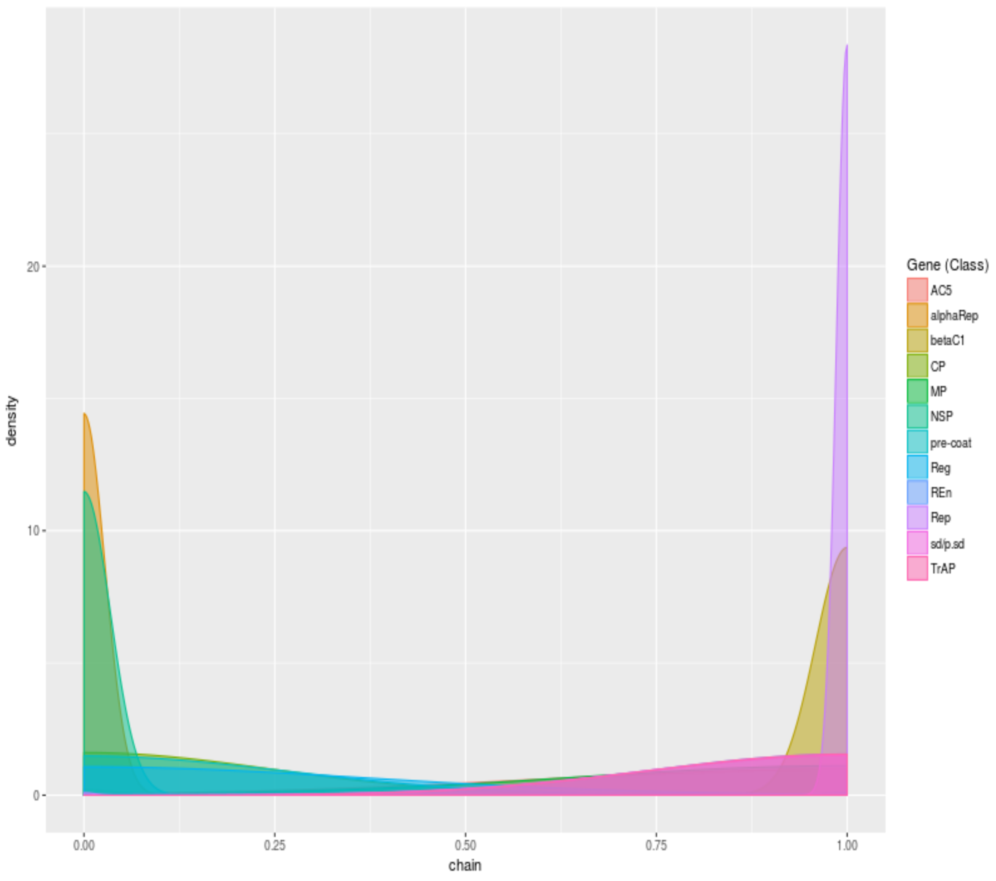

Proportion of Leucine

Histogram

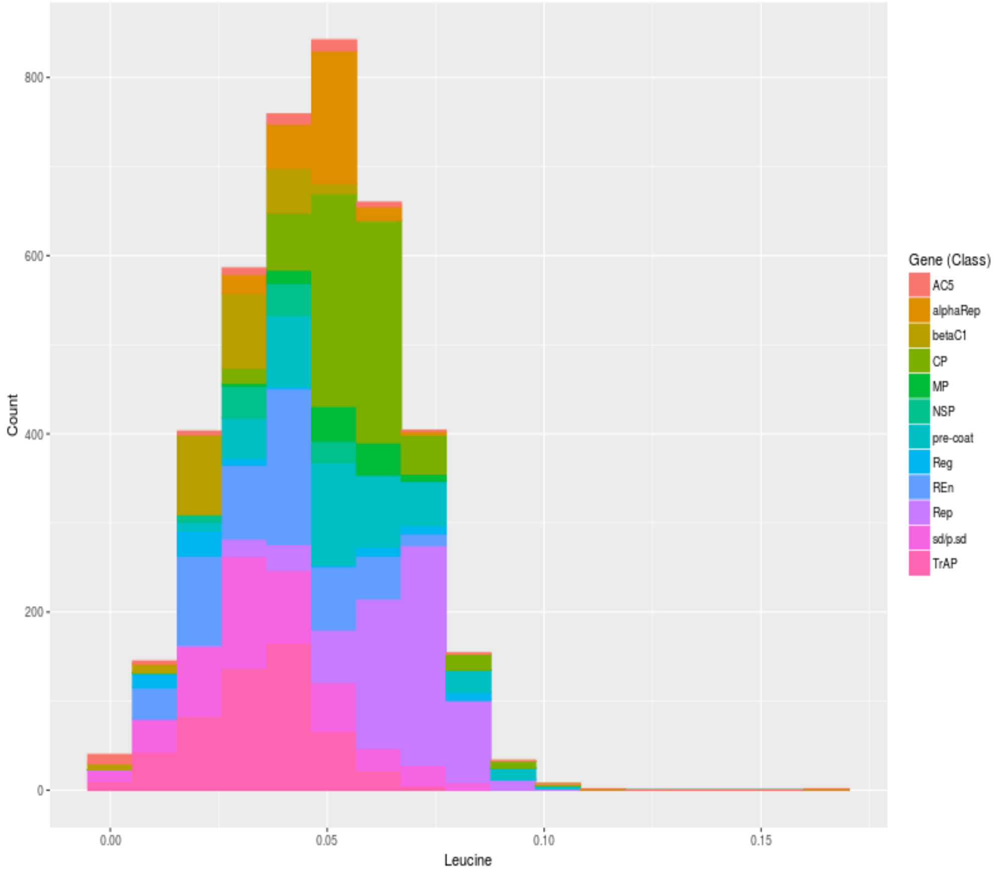

Density

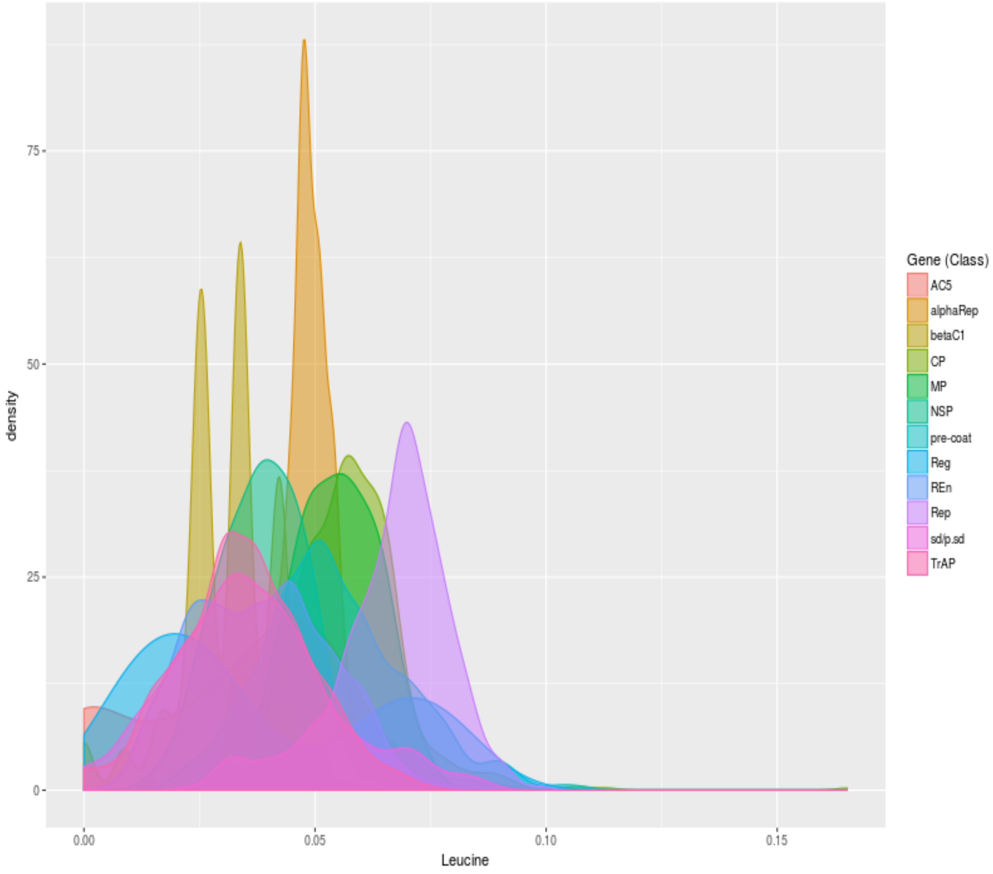

Boxplots

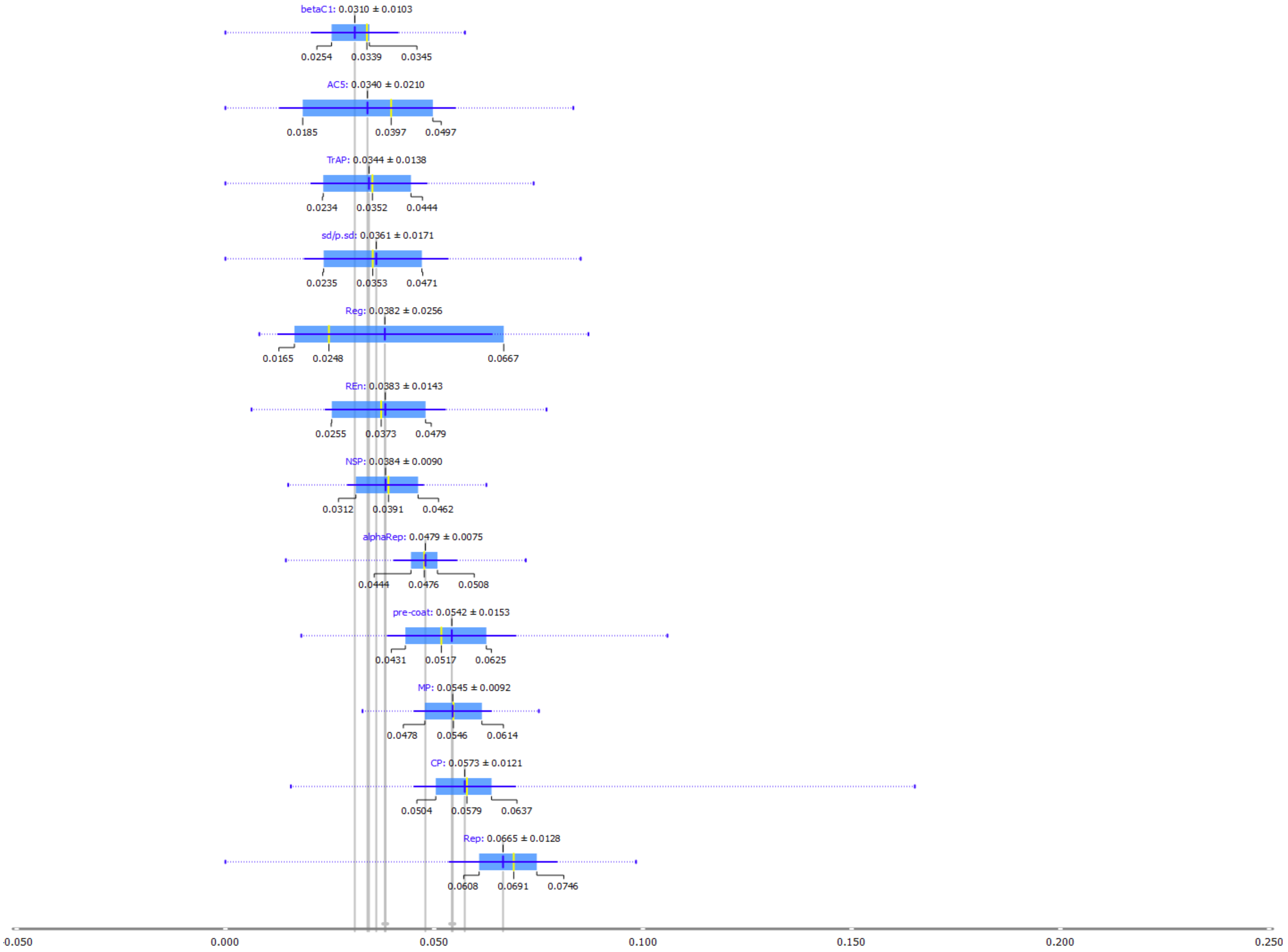

betaC1: 0.0310 ± 0.0103
0.0254 0.0339 0.0345
AC5: 0.0340 ± 0.0210
0.0185 0.0397 0.0497
TrAP: 0.0344 ± 0.0138
0.0234 0.0352 0.0444
sd/p.sd: 0.0361 ± 0.0171
0.0235 0.0353 0.0471
Reg: 0.0382 ± 0.0256
0.0165 0.0248 0.0667
REn: 0.0383 ± 0.0143
0.0255 0.0373 0.0479
NSP: 0.0384 ± 0.0090
0.0312 0.0391 0.0462
alphaRep: 0.0479 ± 0.0075
0.0444 0.0476 0.0508
pre-coat: 0.0542 ± 0.0153
0.0431 0.0517 0.0625
MP: 0.0545 ± 0.0092
0.0478 0.0546 0.0614
CP: 0.0573 ± 0.0121
0.0504 0.0579 0.0637
Rep: 0.0665 ± 0.0128
0.0608 0.0691 0.0746
-0.050 0.000 0.050 0.100 0.150 0.200 0.250

Proportion of Lysine

Histogram

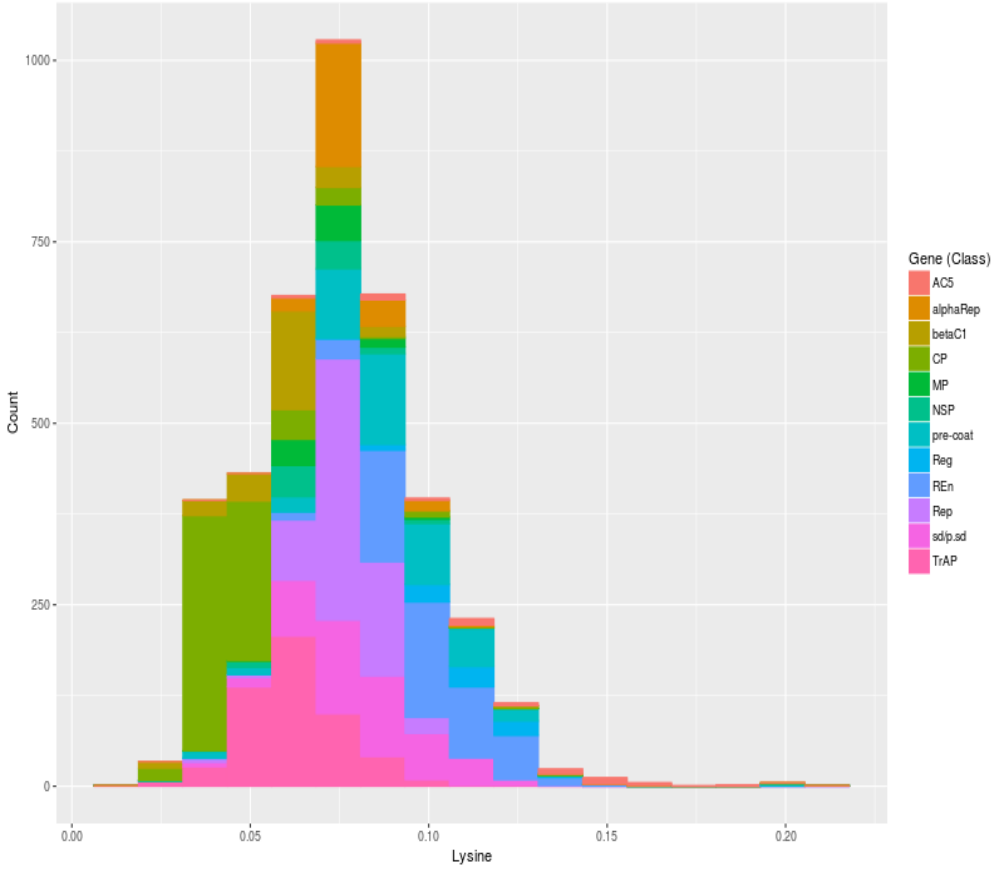

Density

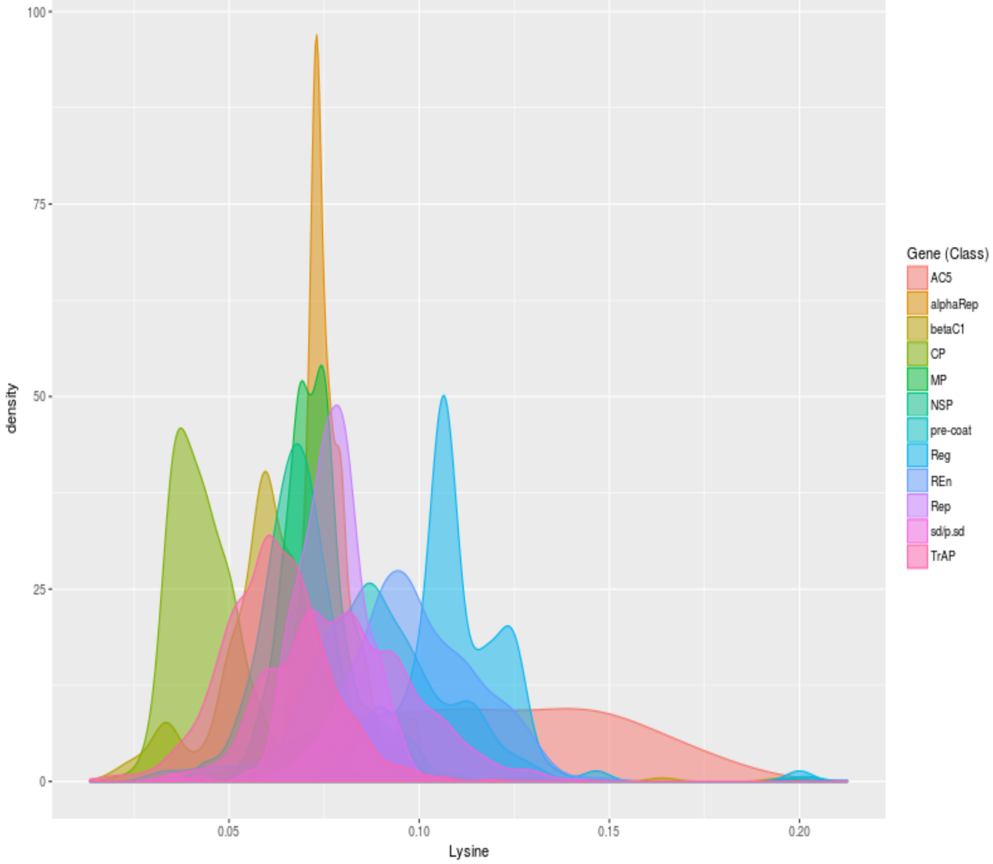

Boxplots

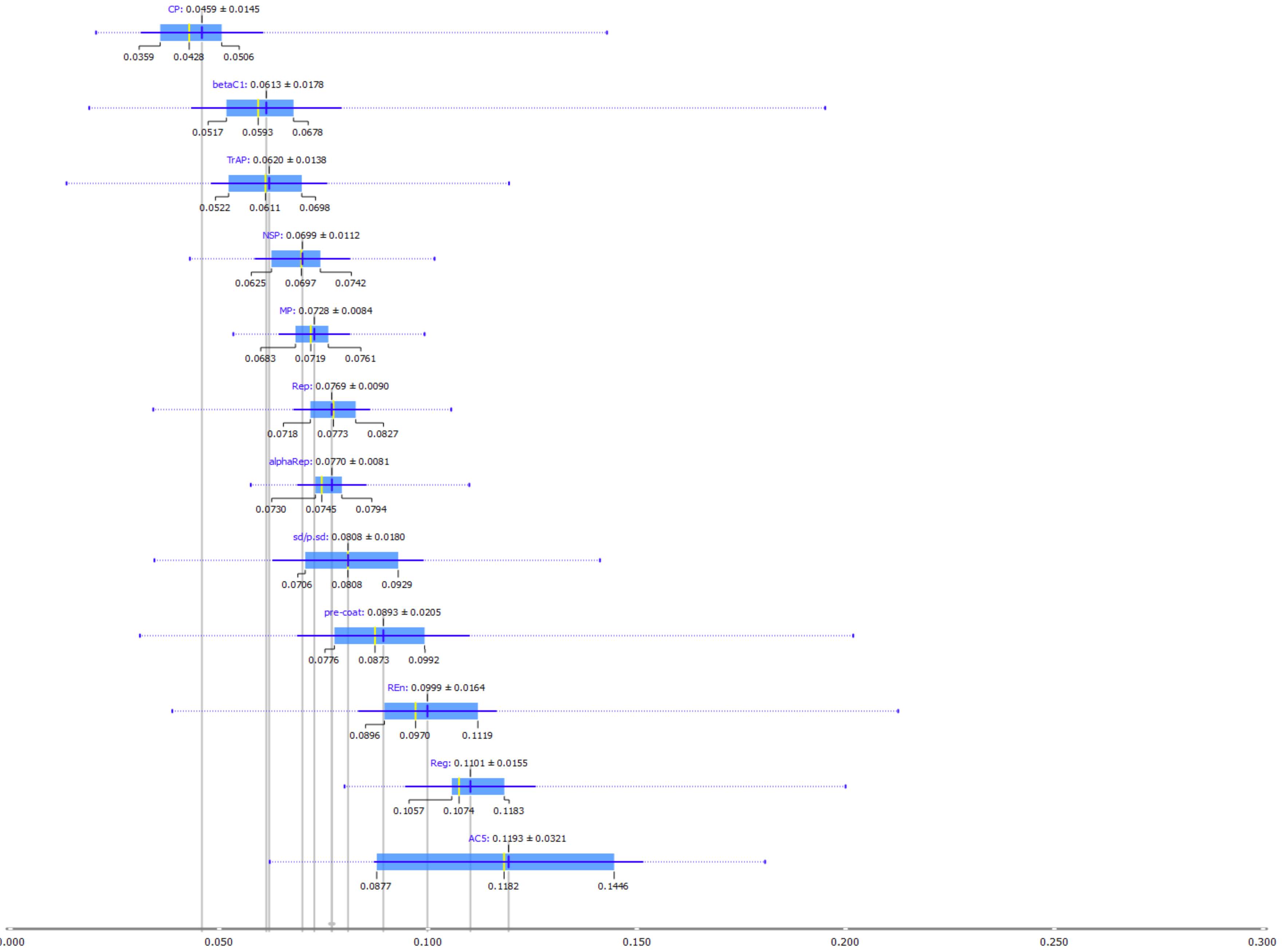

CP: 0.0459 ± 0.0145
0.0359
0.0428
0.0506
betaC1: 0.0613 ± 0.0178
0.0517
0.0593
0.0678
TrAP: 0.0620 ± 0.0138
0.0522
0.0611
0.0698
NSP: 0.0699 ± 0.0112
0.0625
0.0697
0.0742
MP: 0.0728 ± 0.0084
0.0683
0.0719
0.0761
Rep: 0.0769 ± 0.0090
0.0718
0.0773
0.0827
alphaRep: 0.0770 ± 0.0081
0.0730
0.0745
0.0794
sd/p.sd: 0.0808 ± 0.0180
0.0706
0.0808
0.0929
pre-coat: 0.0893 ± 0.0205
0.0776
0.0873
0.0992
REn: 0.0999 ± 0.0164
0.0896
0.0970
0.1119
Reg: 0.1101 ± 0.0155
0.1057
0.1074
0.1183
AC5: 0.1193 ± 0.0321
0.0877
0.1182
0.1446
0.000
0.050
0.100
0.150
0.200
0.250
0.300

Proportion of Methionine

Histogram

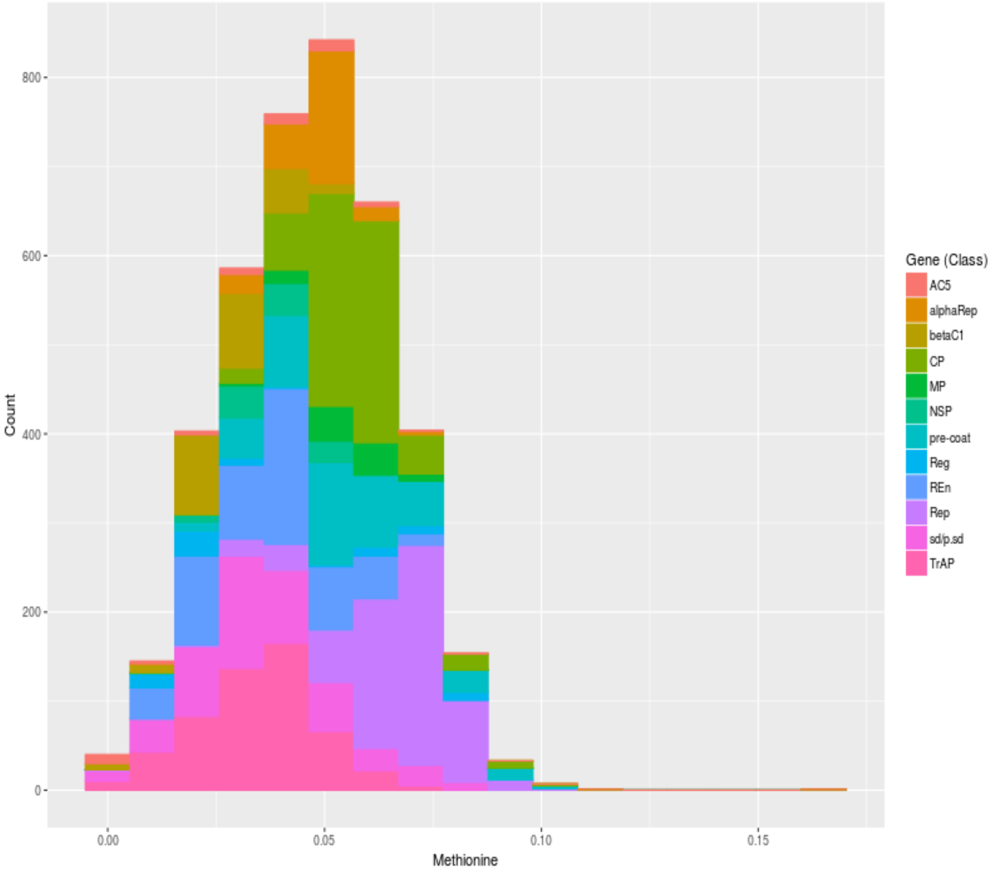

Density

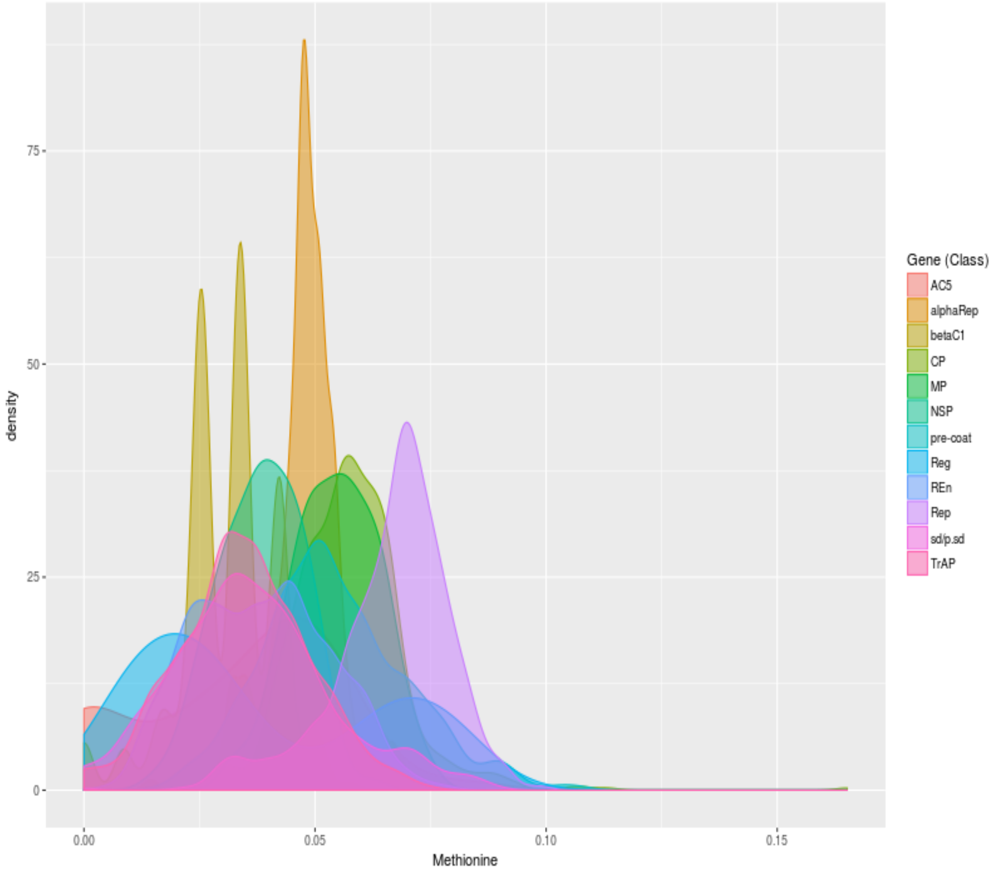

Boxplots

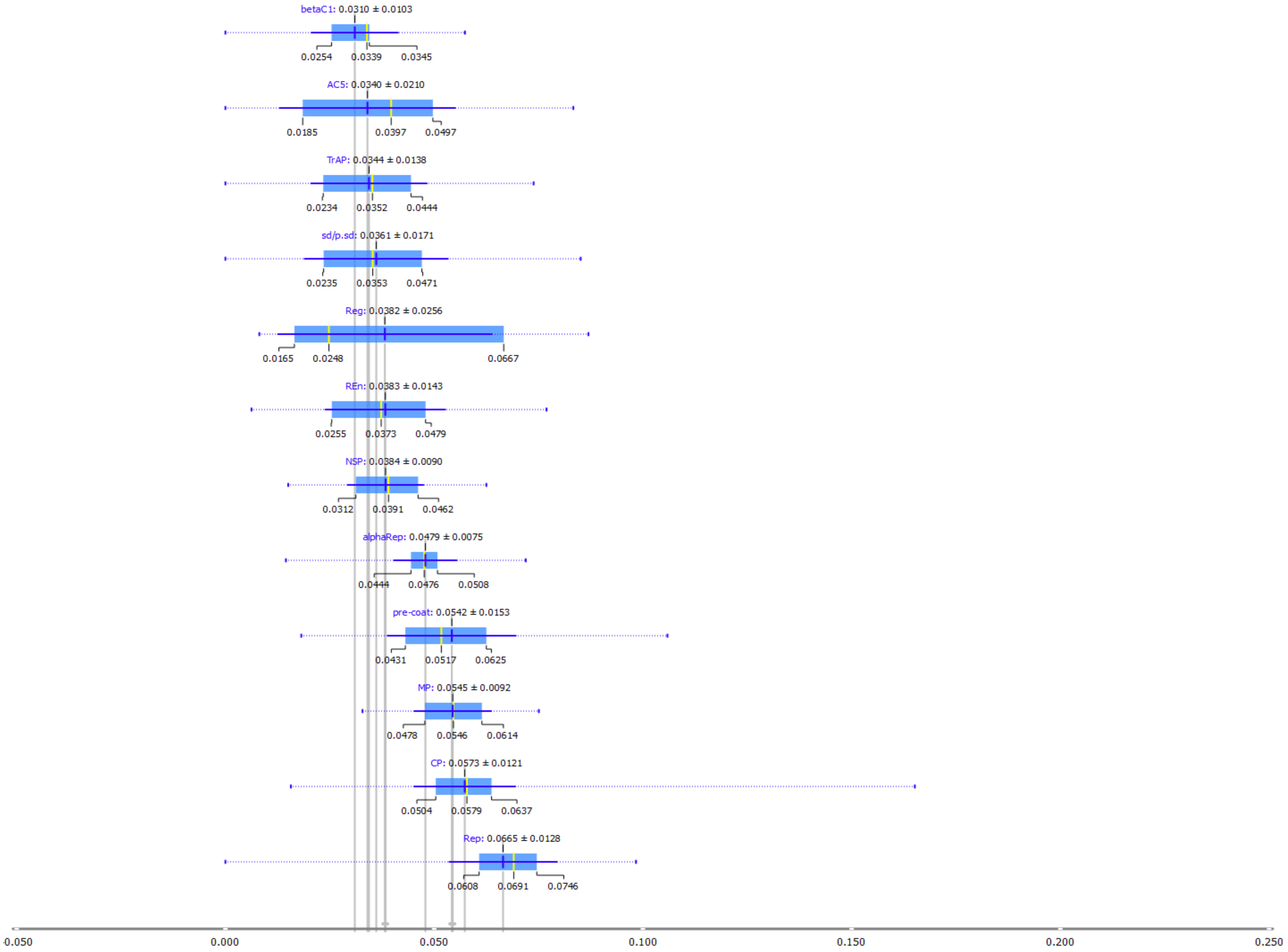

betaC1: 0.0310 ± 0.0103
0.0254 0.0339 0.0345
AC5: 0.0340 ± 0.0210
0.0185 0.0397 0.0497
TrAP: 0.0344 ± 0.0138
0.0234 0.0352 0.0444
sd/p.sd: 0.0361 ± 0.0171
0.0235 0.0353 0.0471
Reg: 0.0382 ± 0.0256
0.0165 0.0248 0.0667
REn: 0.0383 ± 0.0143
0.0255 0.0373 0.0479
NSP: 0.0384 ± 0.0090
0.0312 0.0391 0.0462
alphaRep: 0.0479 ± 0.0075
0.0444 0.0476 0.0508
pre-coat: 0.0542 ± 0.0153
0.0431 0.0517 0.0625
MP: 0.0545 ± 0.0092
0.0478 0.0546 0.0614
CP: 0.0573 ± 0.0121
0.0504 0.0579 0.0637
Rep: 0.0665 ± 0.0128
0.0608 0.0691 0.0746
-0.050 0.000 0.050 0.100 0.150 0.200 0.250

Proportion of Adenine

Histogram

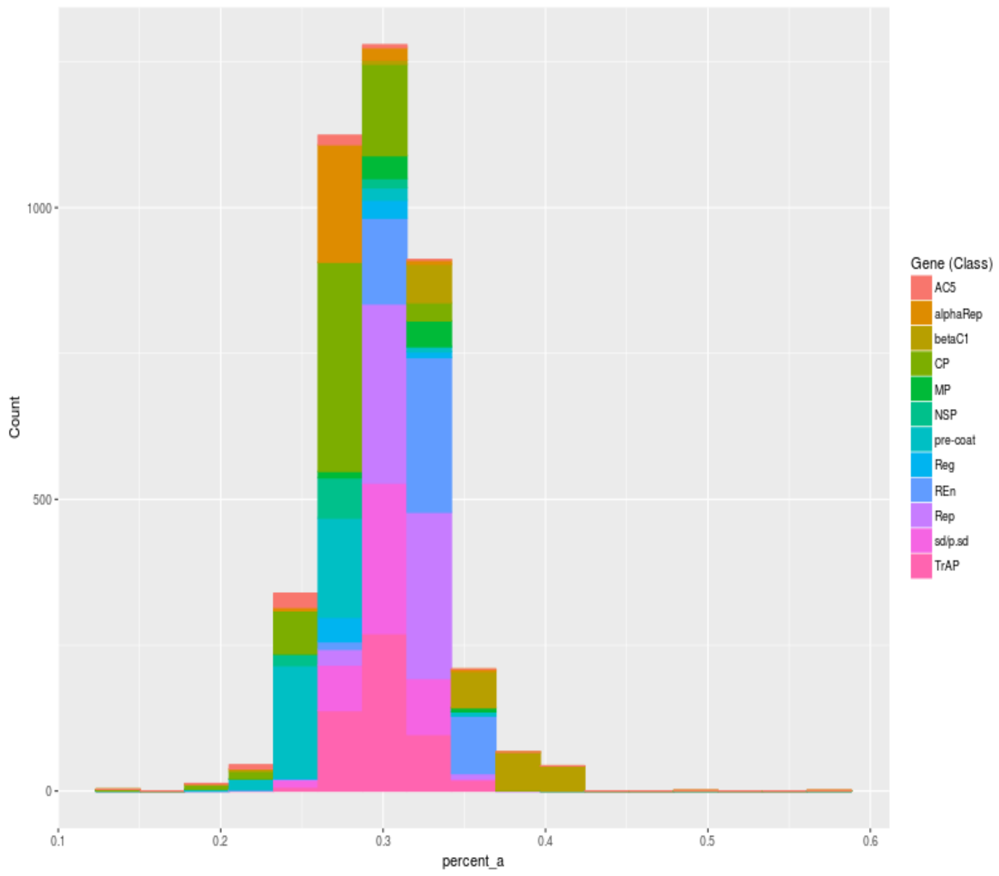

Density

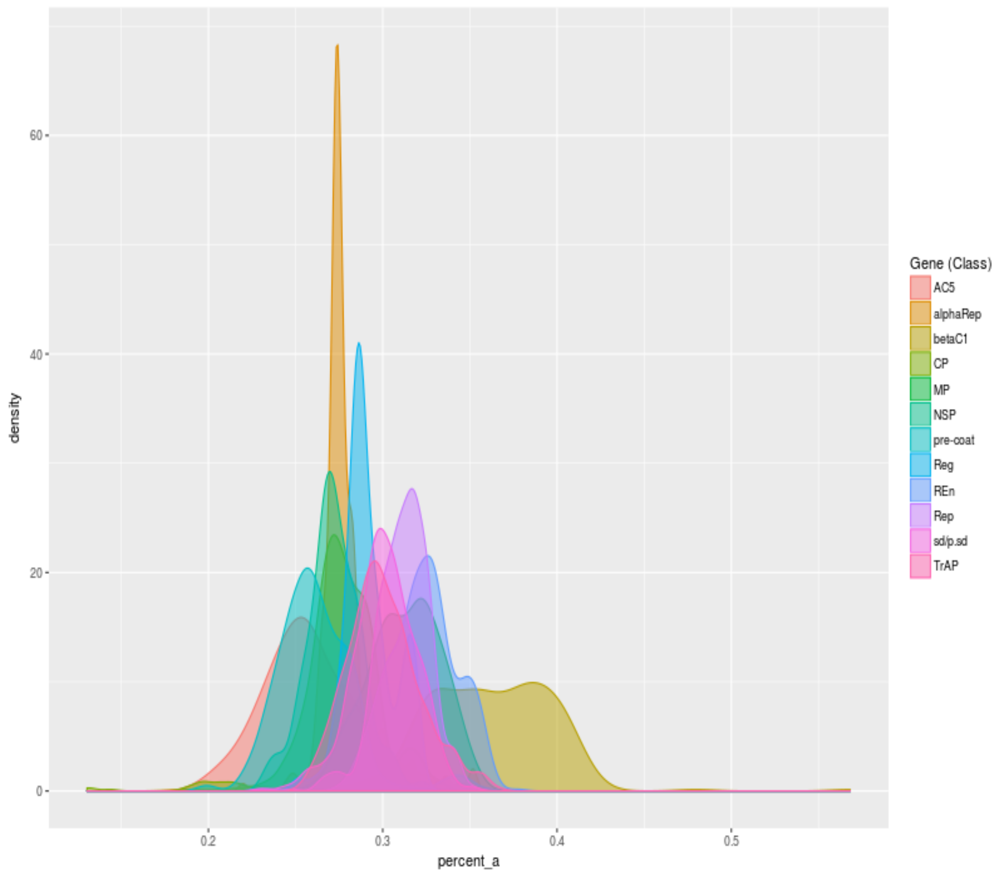

Boxplots

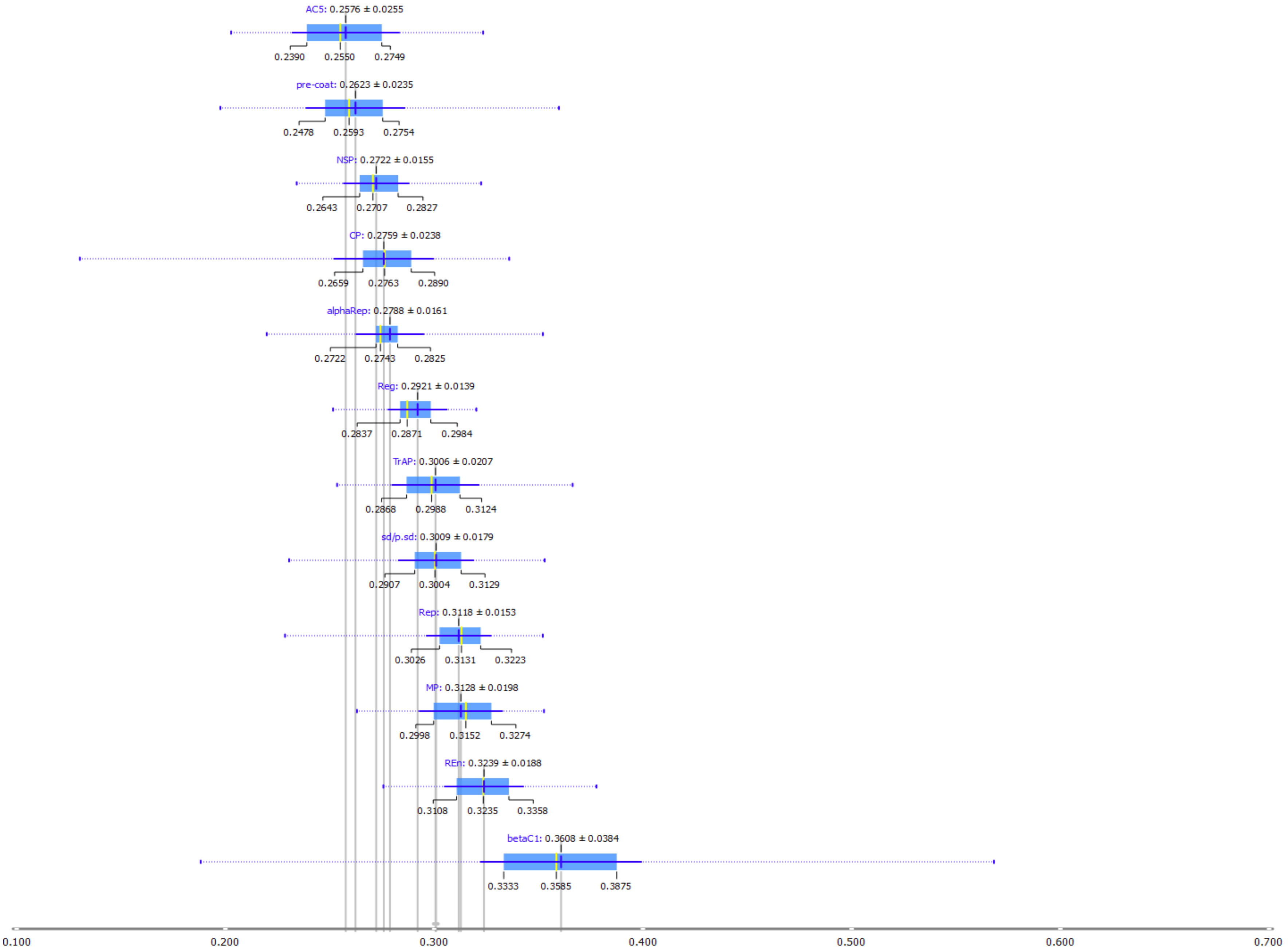

AC5: 0.2576 ± 0.0255
0.2390 0.2550 0.2749
pre-coat: 0.2623 ± 0.0235
0.2478 0.2593 0.2754
NSP: 0.2722 ± 0.0155
0.2643 0.2707 0.2827
CP: 0.2759 ± 0.0238
0.2659 0.2763 0.2890
alphaRep: 0.2788 ± 0.0161
0.2722 0.2743 0.2825
Reg: 0.2921 ± 0.0139
0.2837 0.2871 0.2984
TrAP: 0.3006 ± 0.0207
0.2868 0.2988 0.3124
sd/p.sd: 0.3009 ± 0.0179
0.2907 0.3004 0.3129
Rep: 0.3118 ± 0.0153
0.3026 0.3131 0.3223
MP: 0.3128 ± 0.0198
0.2998 0.3152 0.3274
REn: 0.3239 ± 0.0188
0.3108 0.3235 0.3358
betaC1: 0.3608 ± 0.0384
0.3333 0.3585 0.3875
0.100 0.200 0.300 0.400 0.500 0.600 0.700

Proportion of Adenine in region 1

Histogram

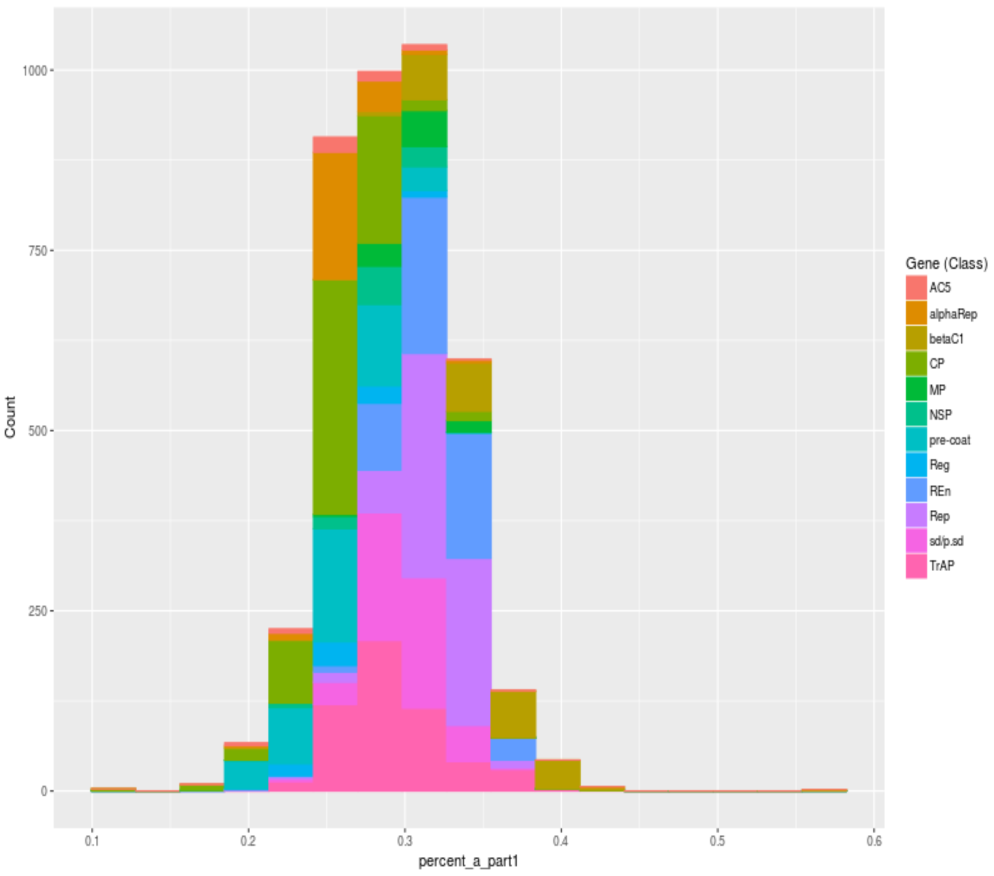

Density

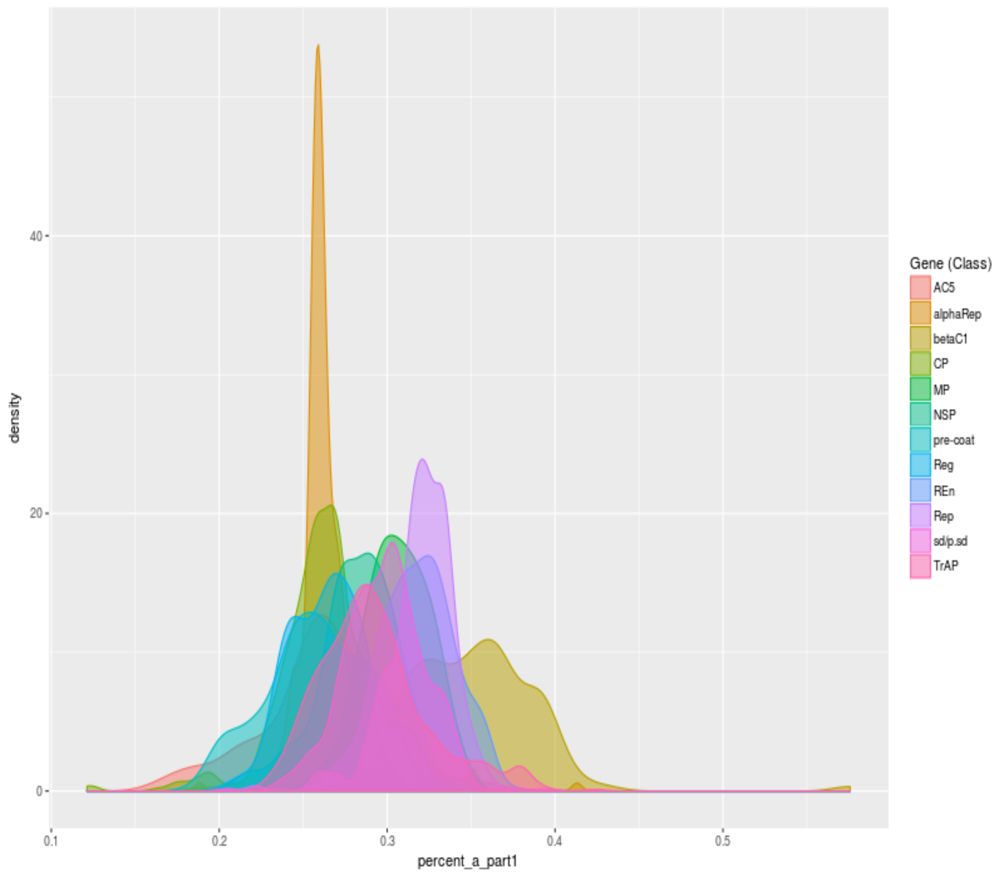

Boxplots

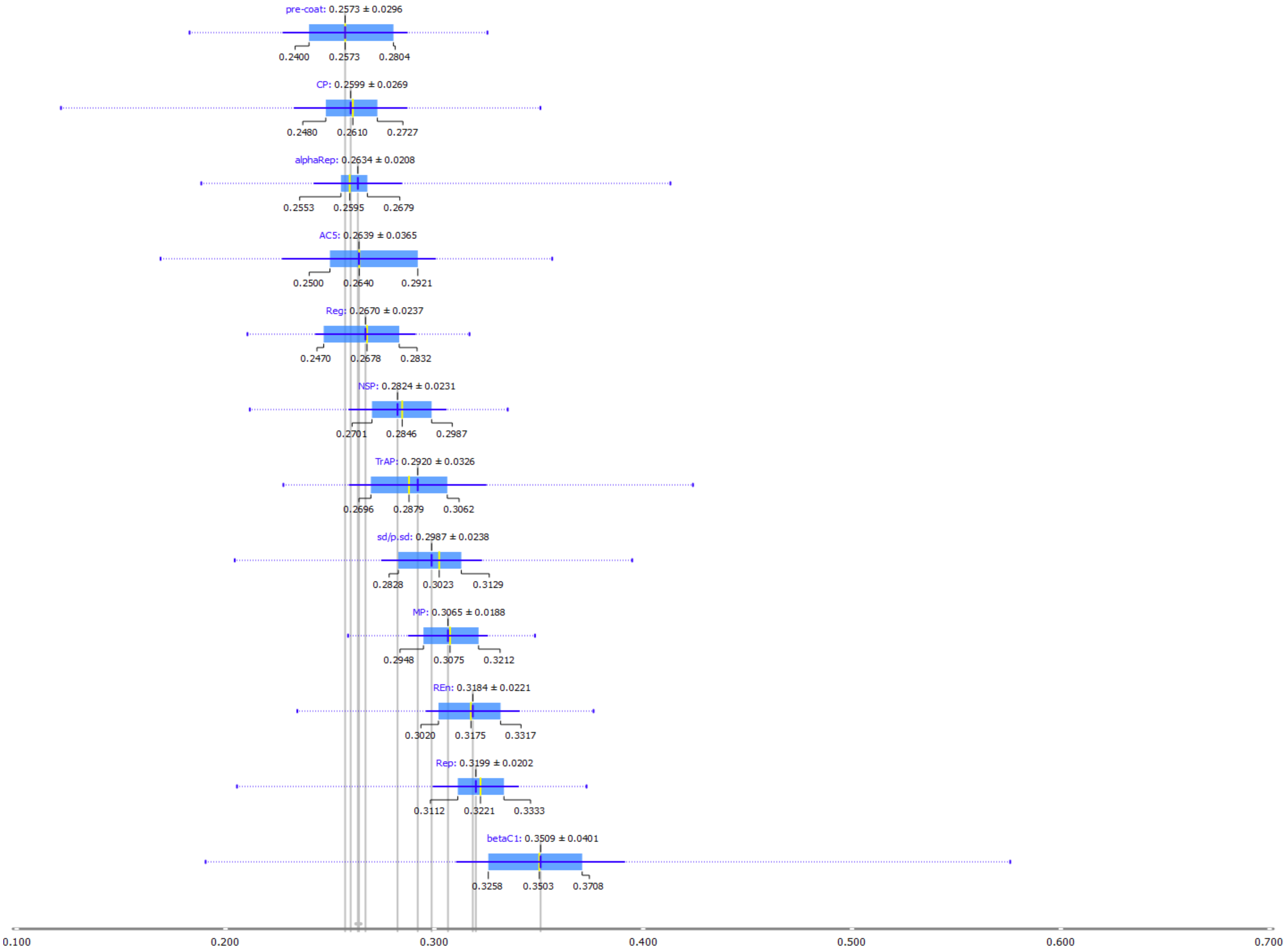

pre-coat: 0.2573 ± 0.0296
0.2400 0.2573 0.2804
CP: 0.2599 ± 0.0269
0.2480 0.2610 0.2727
alphaRep: 0.2634 ± 0.0208
0.2553 0.2595 0.2679
AC5: 0.2639 ± 0.0365
0.2500 0.2640 0.2921
Reg: 0.2670 ± 0.0237
0.2470 0.2678 0.2832
NSP: 0.2824 ± 0.0231
0.2701 0.2846 0.2987
TrAP: 0.2920 ± 0.0326
0.2696 0.2879 0.3062
sd/p.sd: 0.2987 ± 0.0238
0.2828 0.3023 0.3129
MP: 0.3065 ± 0.0188
0.2948 0.3075 0.3212
REn: 0.3184 ± 0.0221
0.3020 0.3175 0.3317
Rep: 0.3199 ± 0.0202
0.3112 0.3221 0.3333
betaC1: 0.3509 ± 0.0401
0.3258 0.3503 0.3708
0.100 0.200 0.300 0.400 0.500 0.600 0.700

Proportion of Adenine in region 2

Histogram

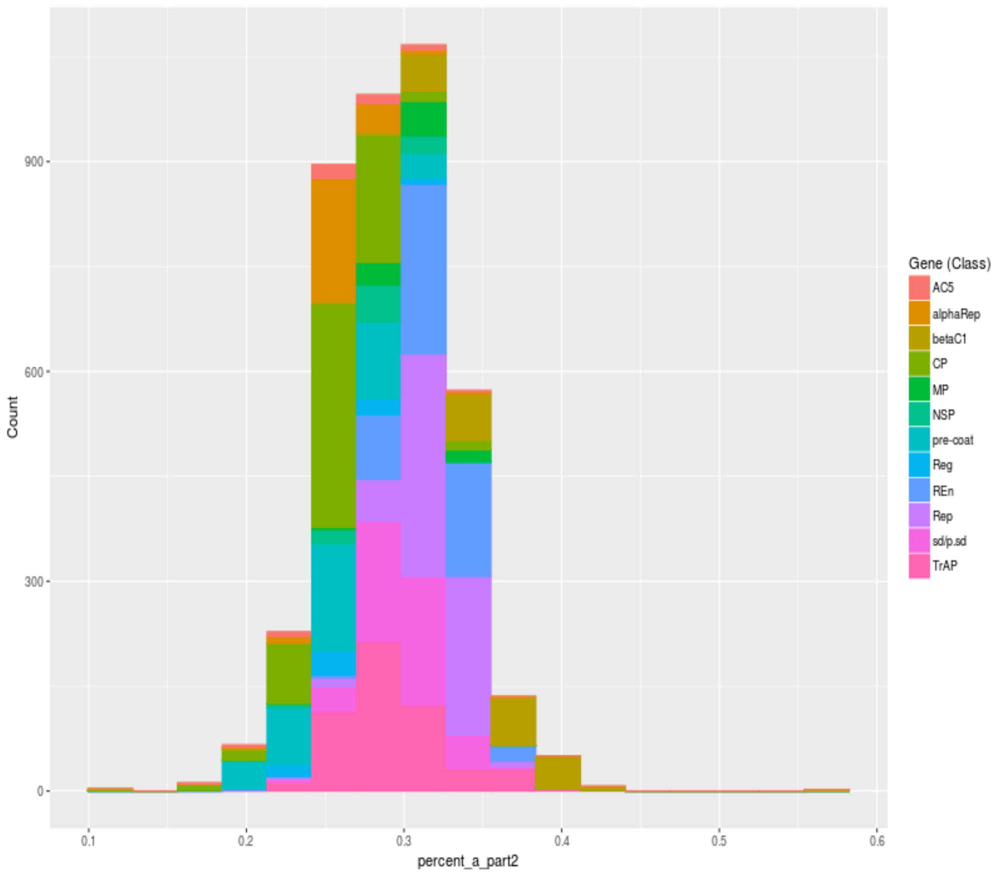

Density

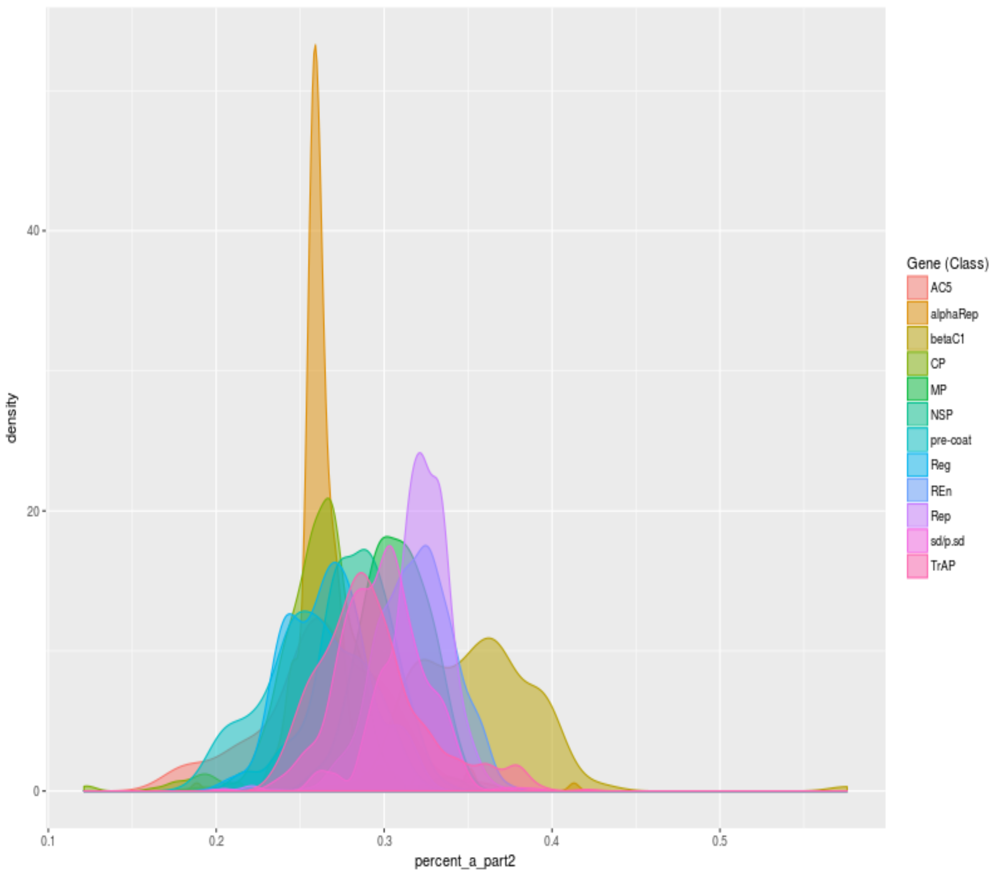

Boxplots

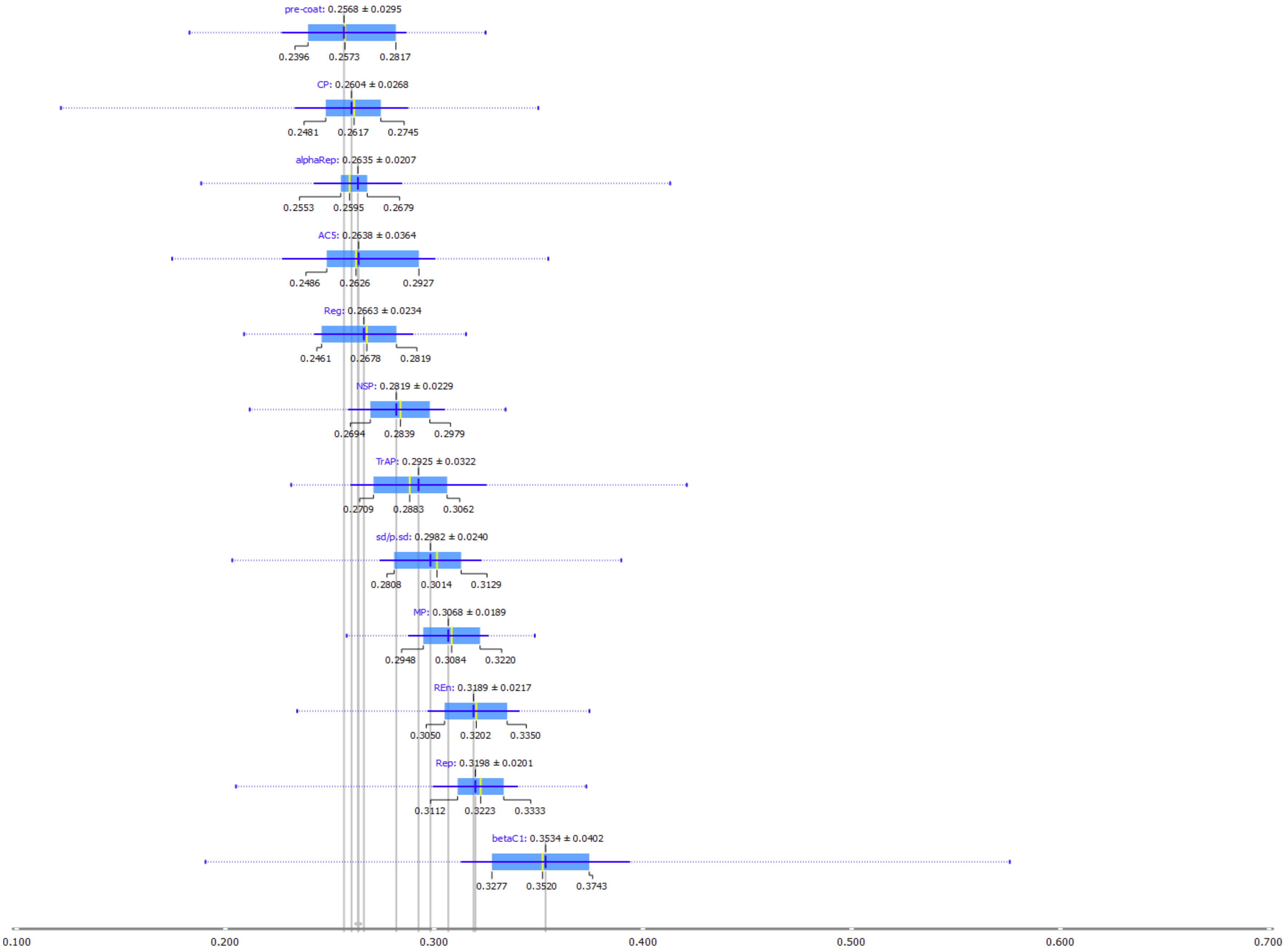
pre-coat: 0.2568 ± 0.0295
0.2396 0.2573 0.2817
CP: 0.2604 ± 0.0268
0.2481 0.2617 0.2745
alphaRep: 0.2635 ± 0.0207
0.2553 0.2595 0.2679
AC5: 0.2638 ± 0.0364
0.2486 0.2626 0.2927
Reg: 0.2663 ± 0.0234
0.2461 0.2678 0.2819
NSP: 0.2819 ± 0.0229
0.2694 0.2839 0.2979
TrAP: 0.2925 ± 0.0322
0.2709 0.2883 0.3062
sd/p.sd: 0.2982 ± 0.0240
0.2808 0.3014 0.3129
MP: 0.3068 ± 0.0189
0.2948 0.3084 0.3220
REn: 0.3189 ± 0.0217
0.3050 0.3202 0.3350
Rep: 0.3198 ± 0.0201
0.3112 0.3223 0.3333
betaC1: 0.3534 ± 0.0402
0.3277 0.3520 0.3743
0.100 0.200 0.300 0.400 0.500 0.600 0.700

Proportion of Cytosine

Histogram

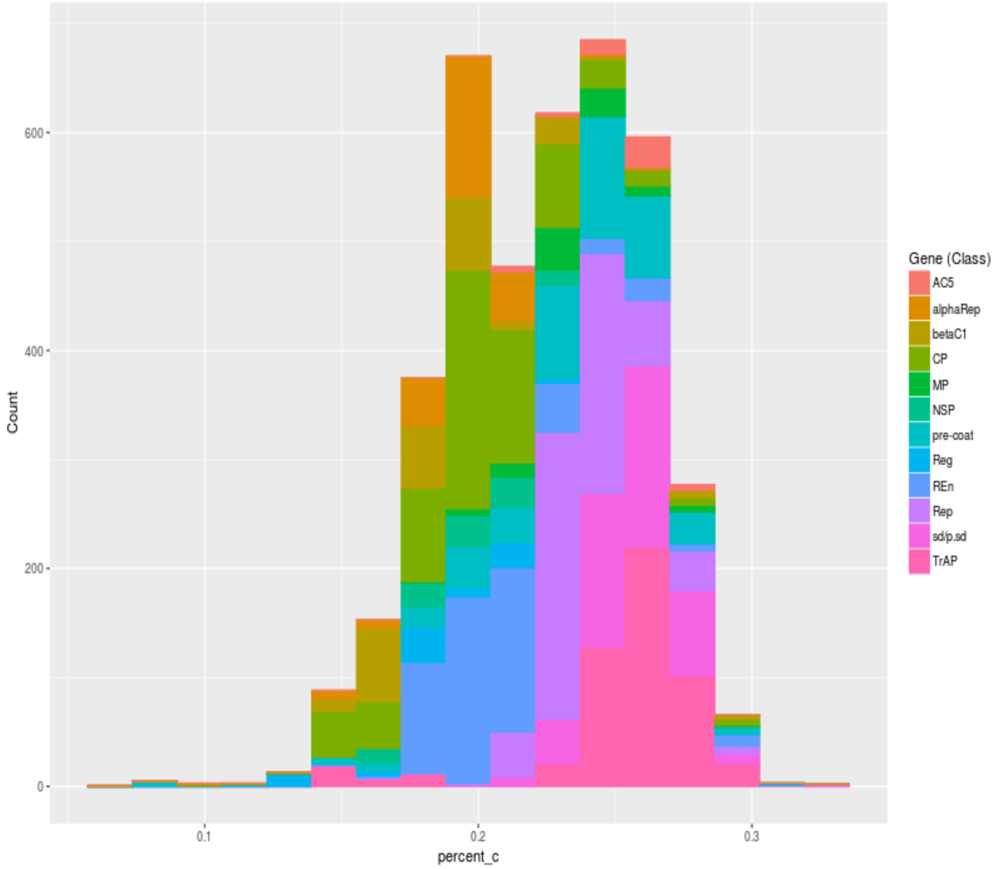

Density

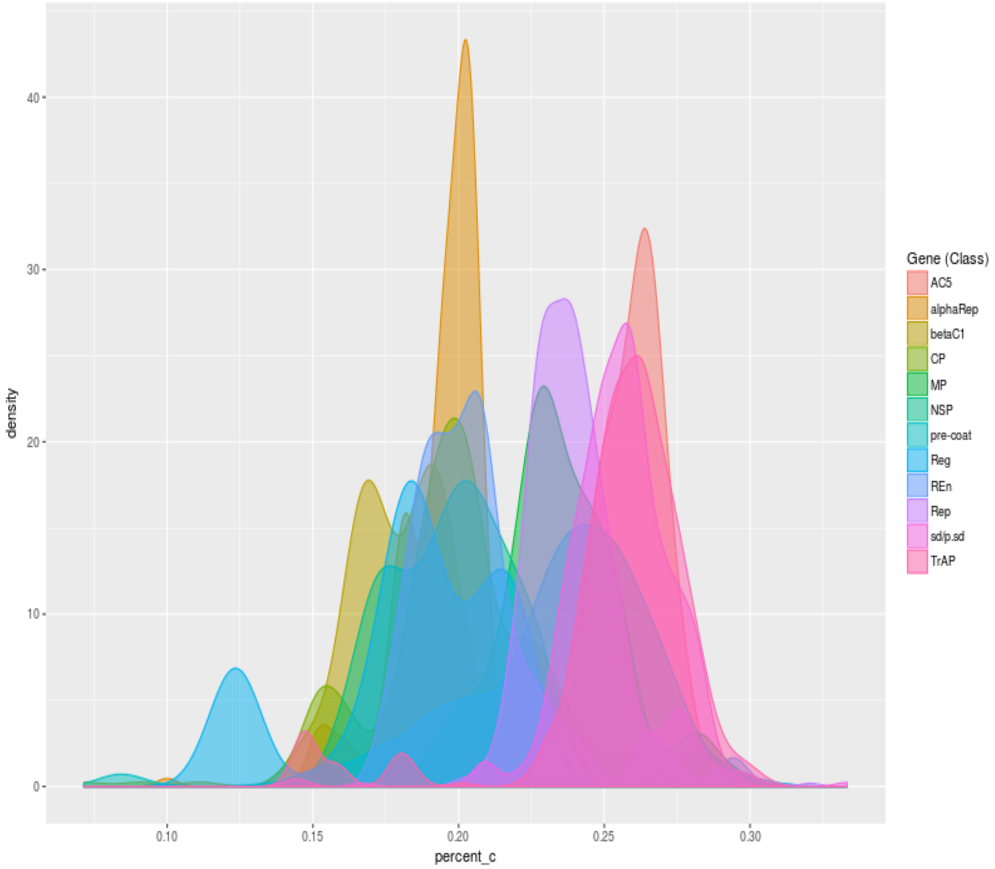

Boxplots

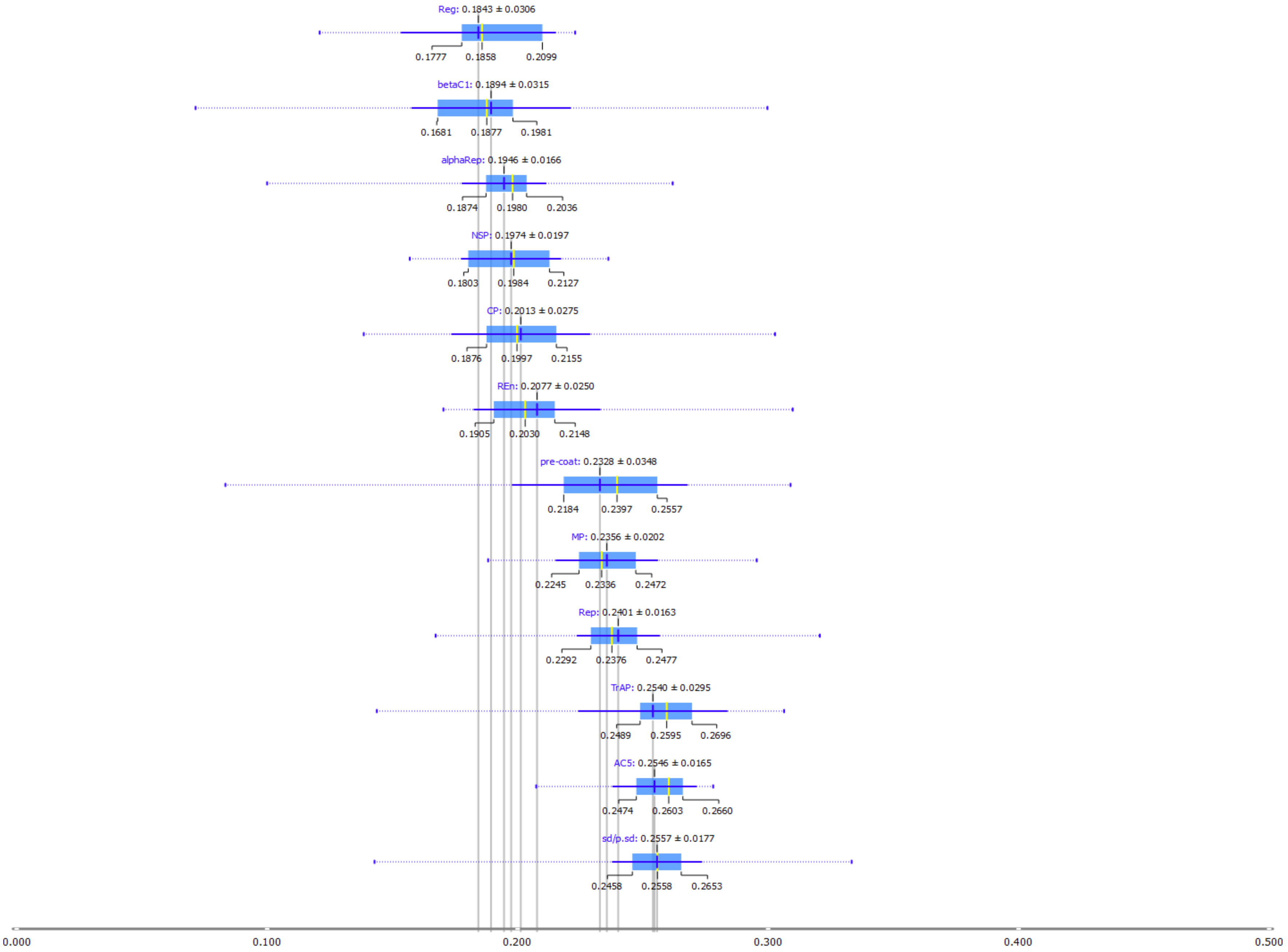

Reg: 0.1843 ± 0.0306
0.1777 0.1858 0.2099
betaC1: 0.1894 ± 0.0315
0.1681 0.1877 0.1981
alphaRep: 0.1946 ± 0.0166
0.1874 0.1980 0.2036
NSP: 0.1974 ± 0.0197
0.1803 0.1984 0.2127
CP: 0.2013 ± 0.0275
0.1876 0.1997 0.2155
REn: 0.2077 ± 0.0250
0.1905 0.2030 0.2148
pre-coat: 0.2328 ± 0.0348
0.2184 0.2397 0.2557
MP: 0.2356 ± 0.0202
0.2245 0.2336 0.2472
Rep: 0.2401 ± 0.0163
0.2292 0.2376 0.2477
TrAP: 0.2540 ± 0.0295
0.2489 0.2595 0.2696
AC5: 0.2546 ± 0.0165
0.2474 0.2603 0.2660
sd/p.sd: 0.2557 ± 0.0177
0.2458 0.2558 0.2653
0.000 0.100 0.200 0.300 0.400 0.500

Proportion of Cytosine in region 1

Histogram

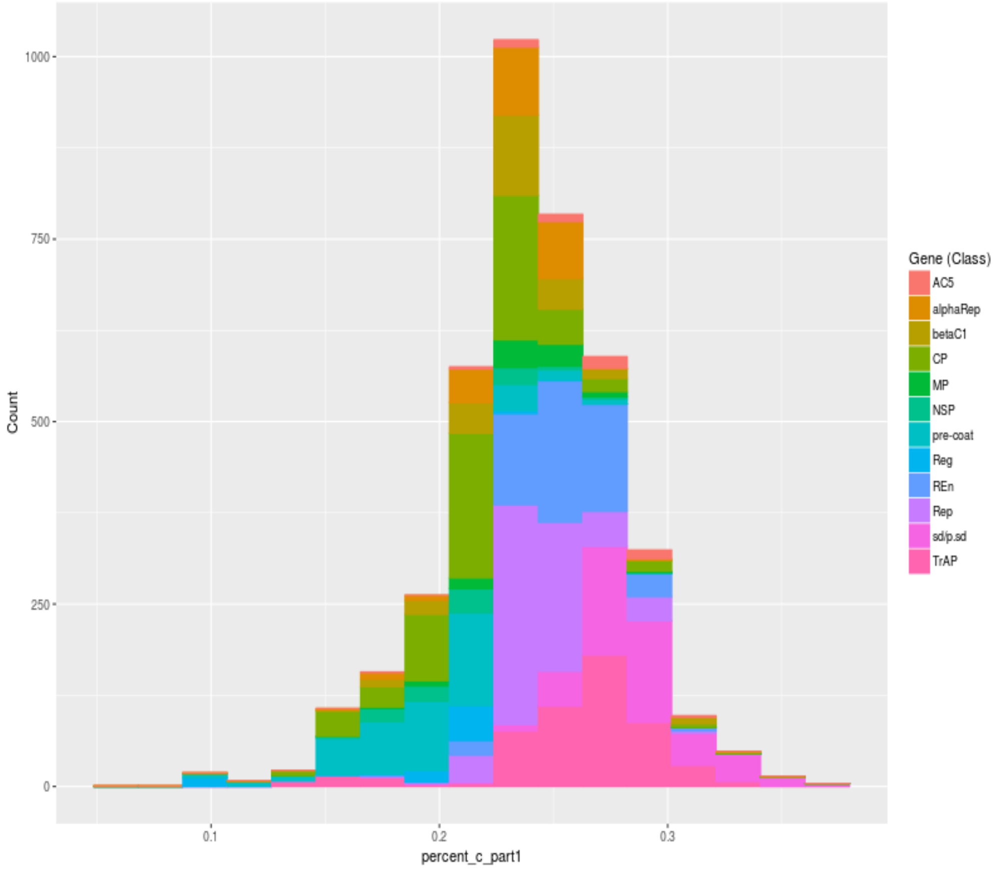

Density

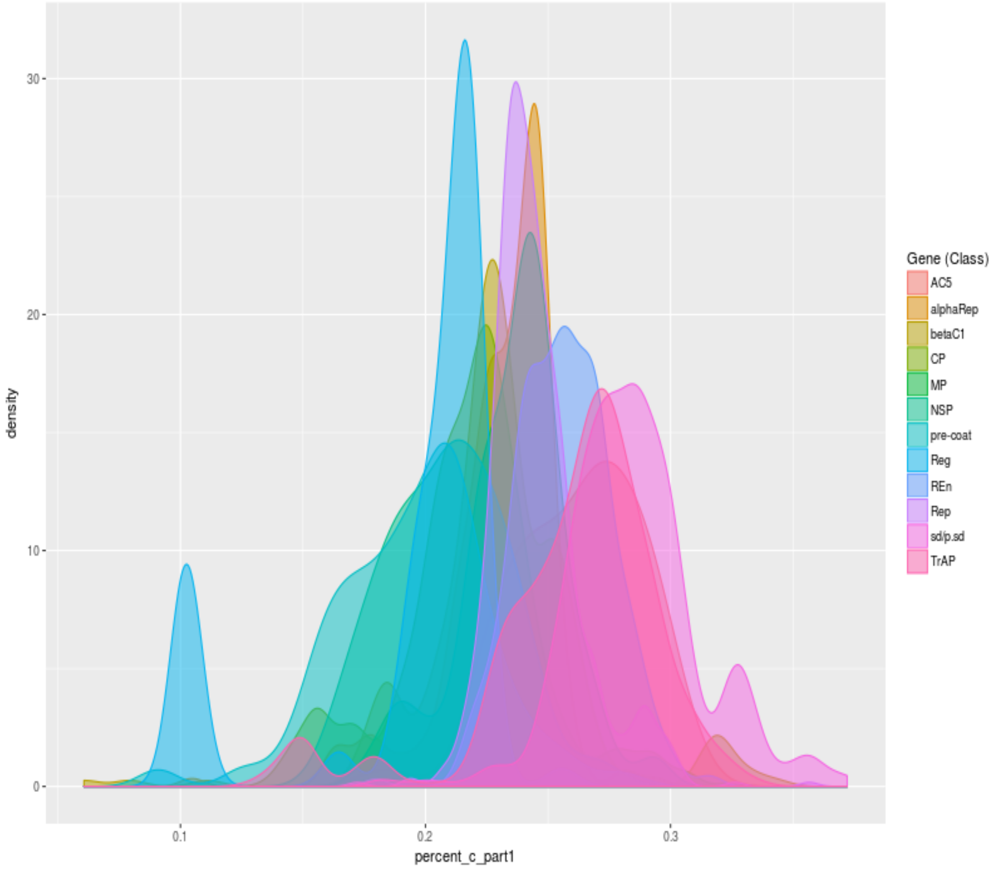

Boxplots

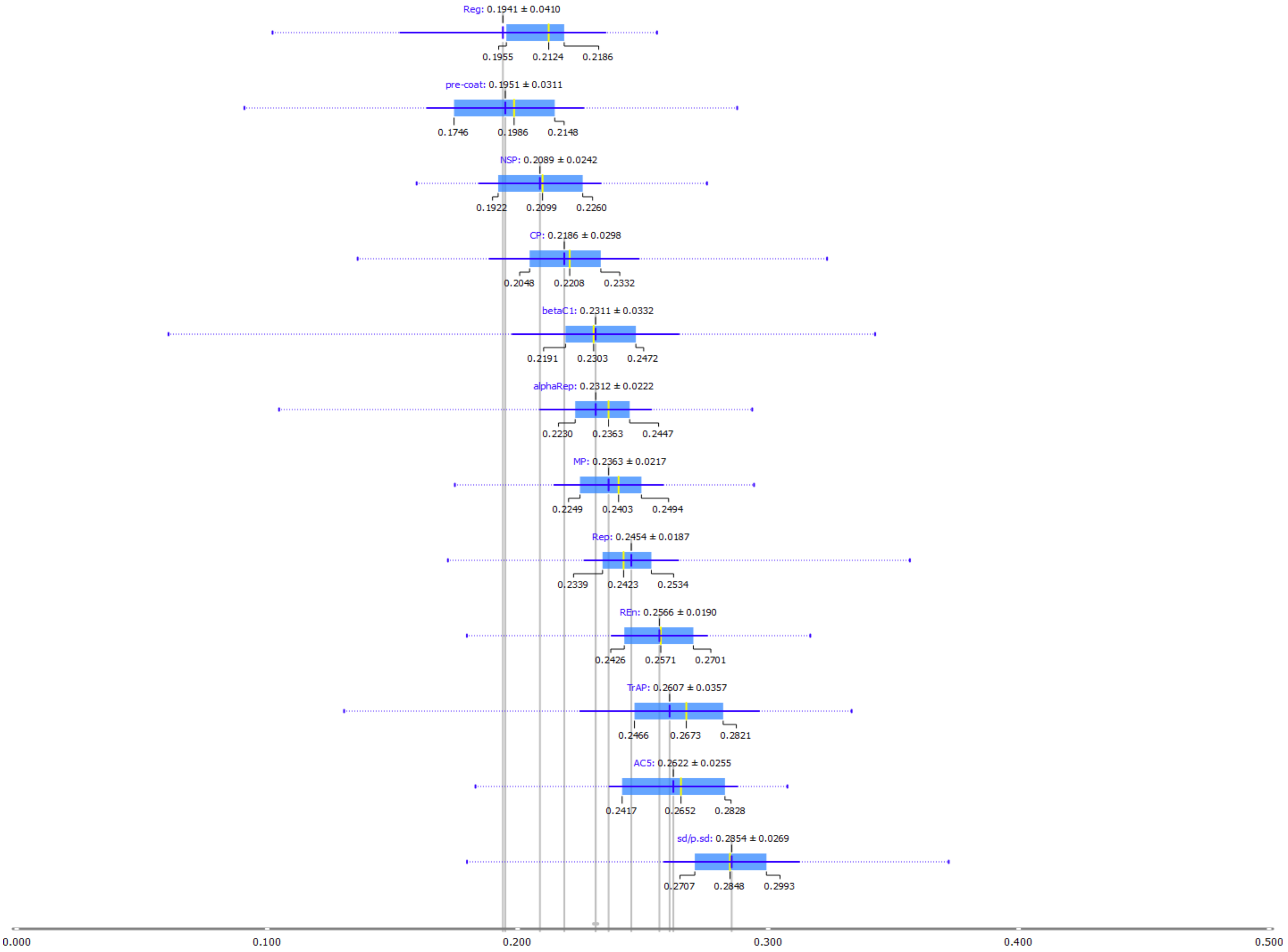
Reg: 0.1941 ± 0.0410
0.1955
0.2124
0.2186
pre-coat: 0.1951 ± 0.0311
0.1746
0.1986
0.2148
NSP: 0.2089 ± 0.0242
0.1922
0.2099
0.2260
CP: 0.2186 ± 0.0298
0.2048
0.2208
0.2332
betaC1: 0.2311 ± 0.0332
0.2191
0.2303
0.2472
alphaRep: 0.2312 ± 0.0222
0.2230
0.2363
0.2447
MP: 0.2363 ± 0.0217
0.2249
0.2403
0.2494
Rep: 0.2454 ± 0.0187
0.2339
0.2423
0.2534
REn: 0.2566 ± 0.0190
0.2426
0.2571
0.2701
TrAP: 0.2607 ± 0.0357
0.2466
0.2673
0.2821
AC5: 0.2622 ± 0.0255
0.2417
0.2652
0.2828
sd/p.sd: 0.2854 ± 0.0269
0.2707
0.2848
0.2993
0.000
0.100
0.200
0.300
0.400
0.500

Proportion of Cytosine in region 2

Histogram

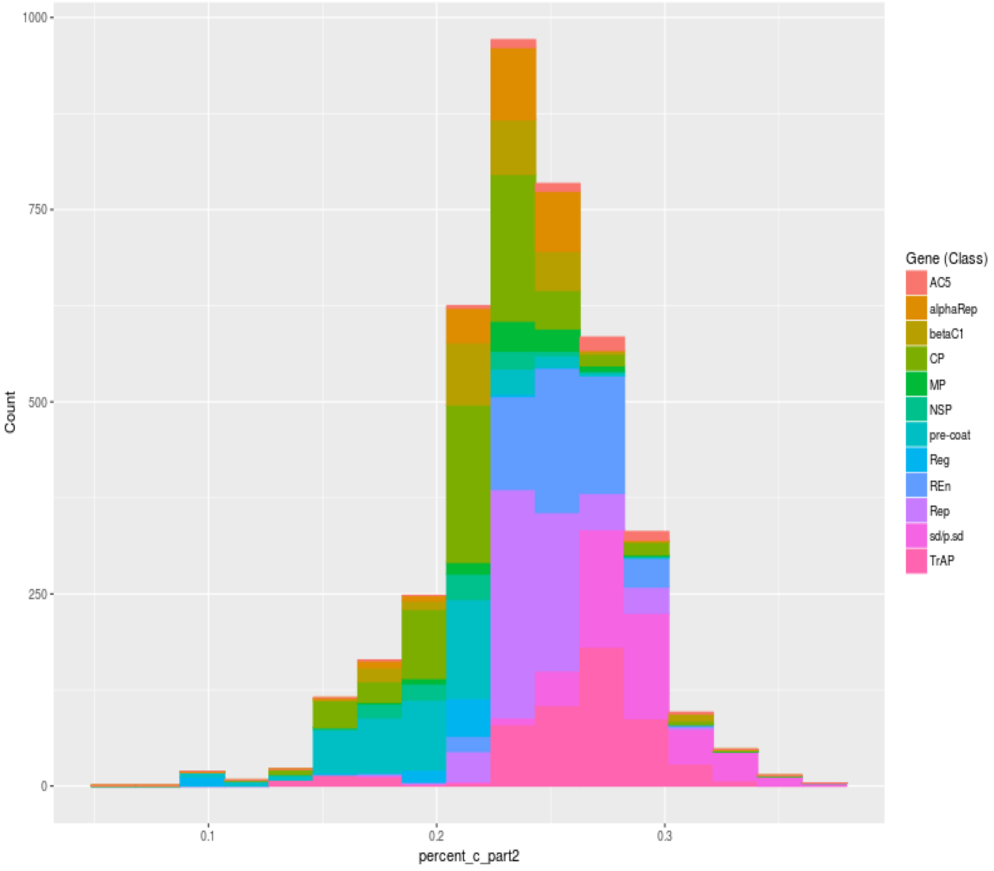

Density

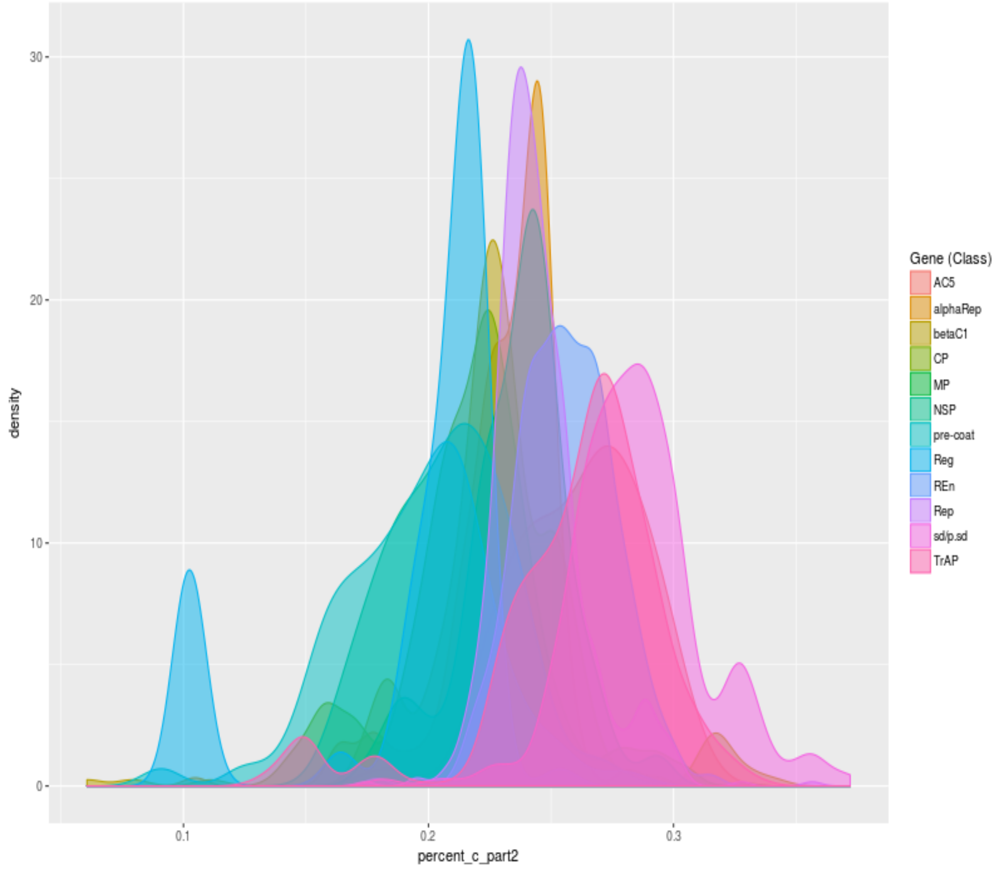

Boxplots

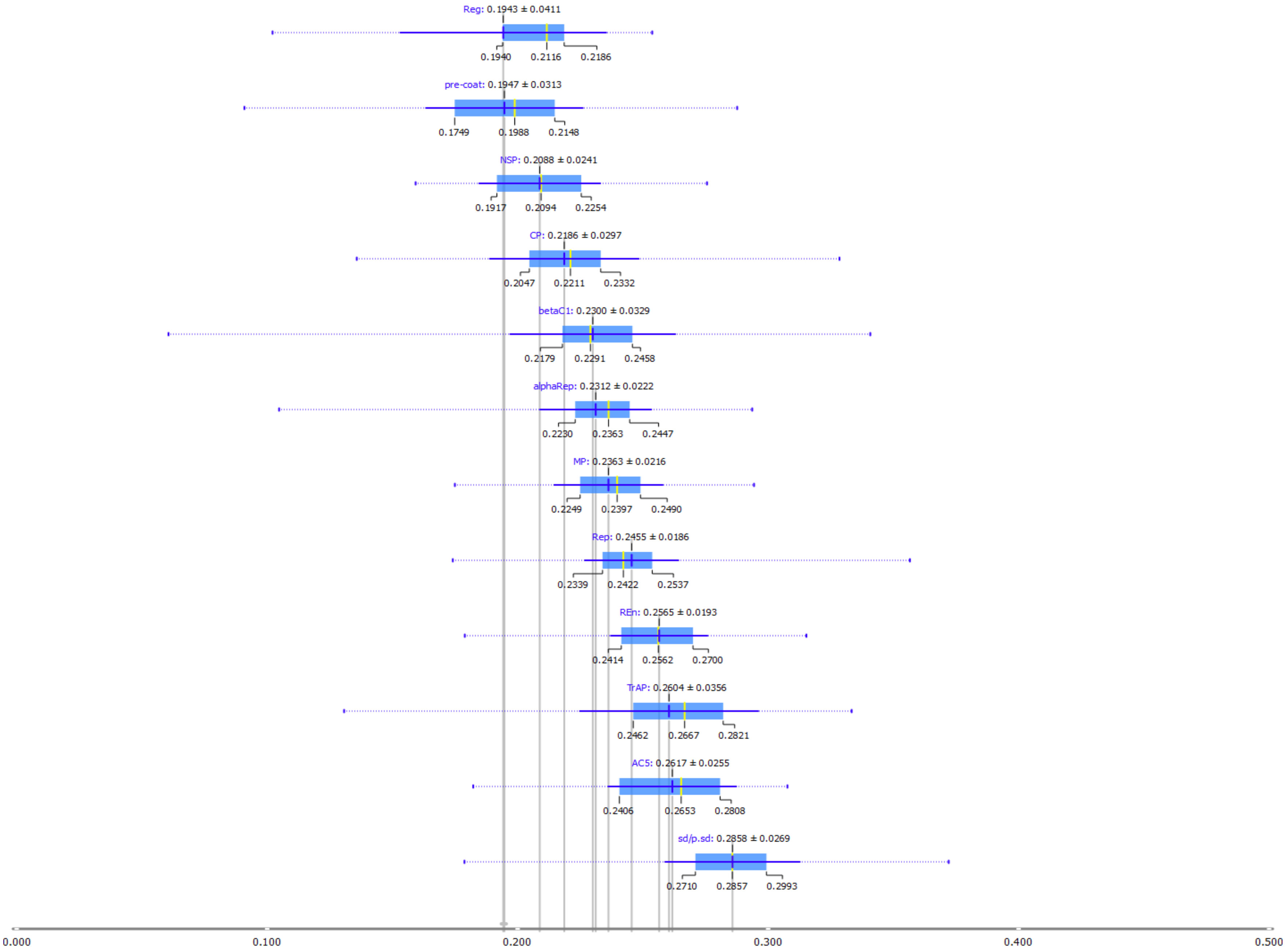

Reg: 0.1943 ± 0.0411
0.1940
0.2116
0.2186
pre-coat: 0.1947 ± 0.0313
0.1749
0.1988
0.2148
NSP: 0.2088 ± 0.0241
0.1917
0.2094
0.2254
CP: 0.2186 ± 0.0297
0.2047
0.2211
0.2332
betaC1: 0.2300 ± 0.0329
0.2179
0.2291
0.2458
alphaRep: 0.2312 ± 0.0222
0.2230
0.2363
0.2447
MP: 0.2363 ± 0.0216
0.2249
0.2397
0.2490
Rep: 0.2455 ± 0.0186
0.2339
0.2422
0.2537
REn: 0.2565 ± 0.0193
0.2414
0.2562
0.2700
TrAP: 0.2604 ± 0.0356
0.2462
0.2667
0.2821
AC5: 0.2617 ± 0.0255
0.2406
0.2653
0.2808
sd/p.sd: 0.2858 ± 0.0269
0.2710
0.2857
0.2993
0.000
0.100
0.200
0.300
0.400
0.500

Proportion of Guanine

Histogram

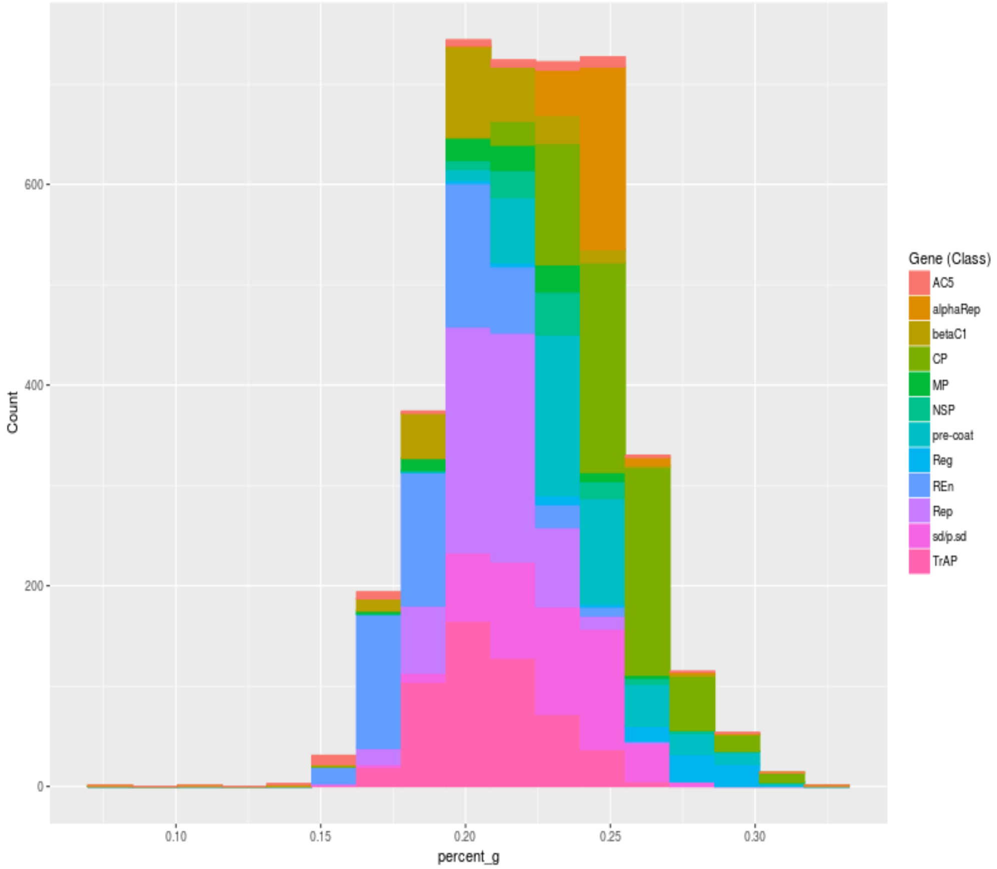

Density

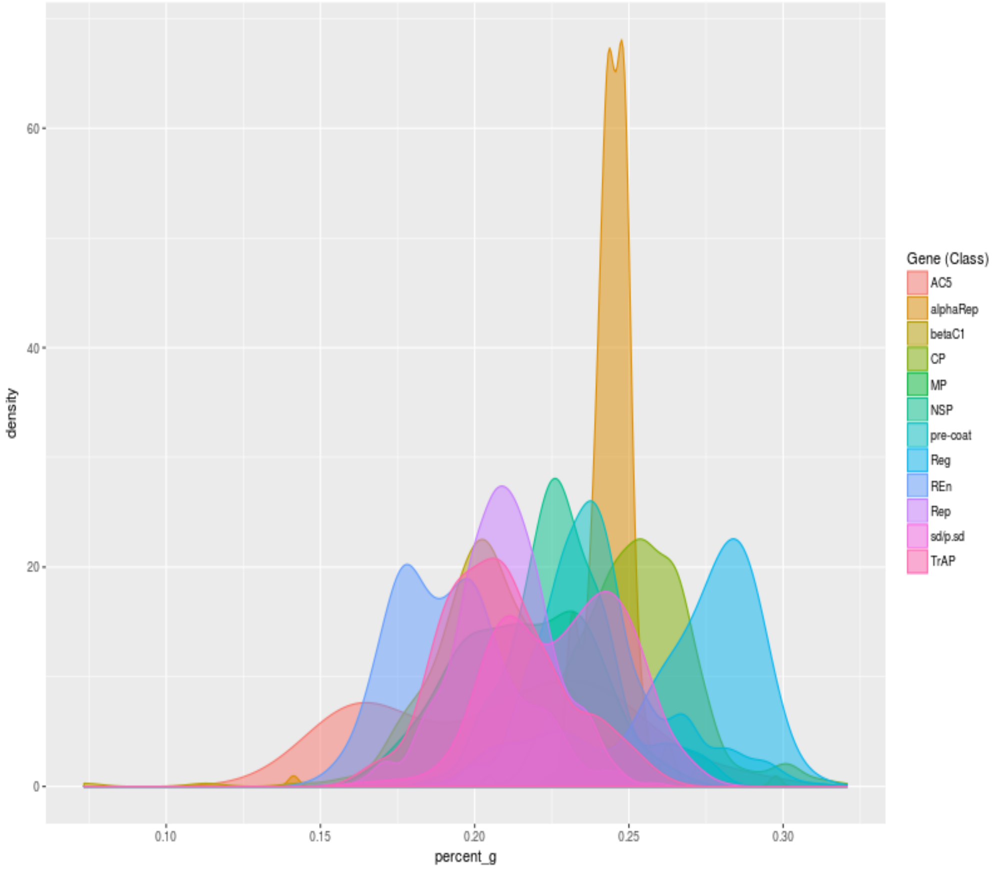

Boxplots

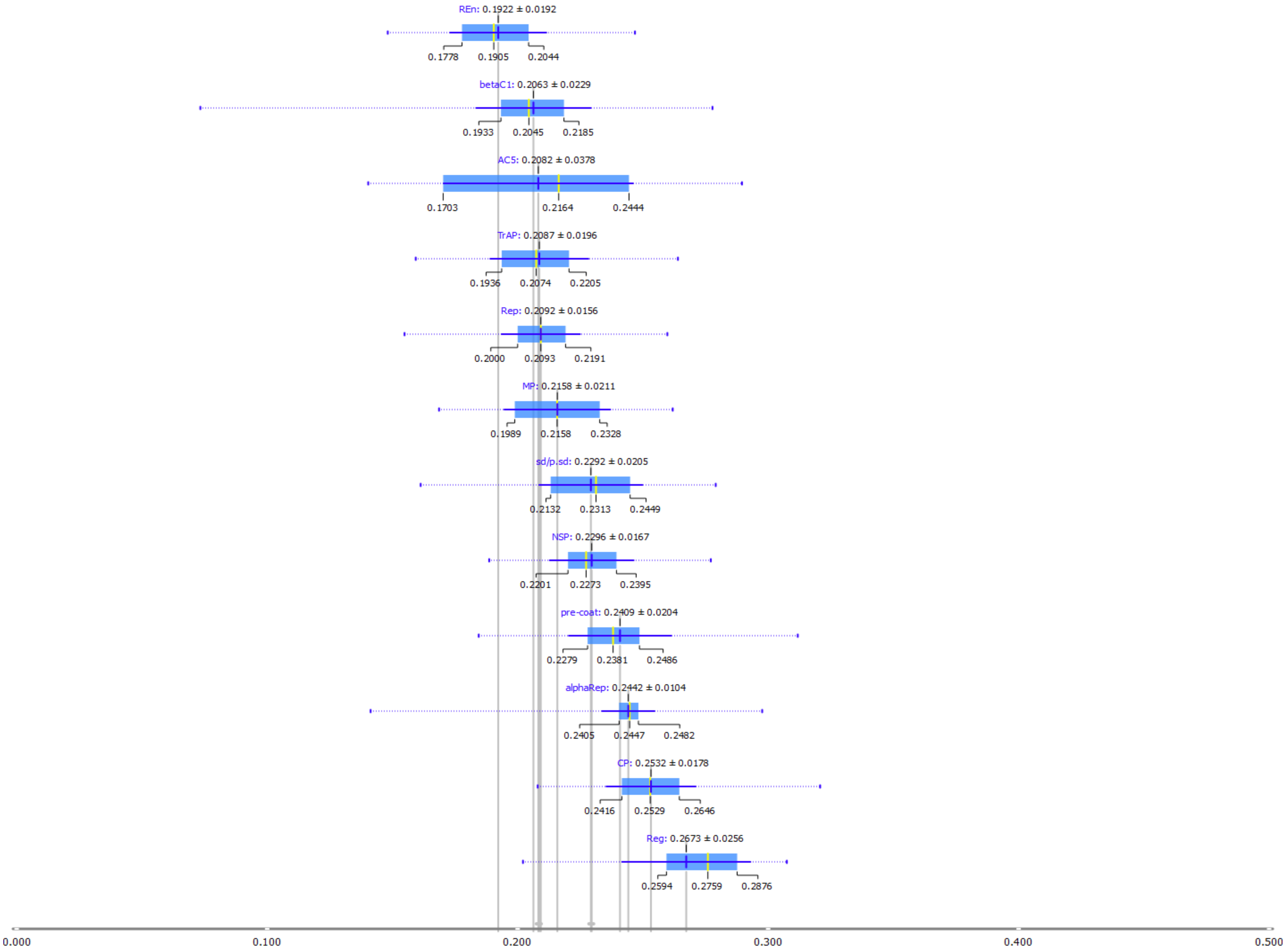
REn: 0.1922 ± 0.0192
0.1778
0.1905
0.2044
betaC1: 0.2063 ± 0.0229
0.1933
0.2045
0.2185
AC5: 0.2082 ± 0.0378
0.1703
0.2164
0.2444
TrAP: 0.2087 ± 0.0196
0.1936
0.2074
0.2205
Rep: 0.2092 ± 0.0156
0.2000
0.2093
0.2191
MP: 0.2158 ± 0.0211
0.1989
0.2158
0.2328
sd/p.sd: 0.2292 ± 0.0205
0.2132
0.2313
0.2449
NSP: 0.2296 ± 0.0167
0.2201
0.2273
0.2395
pre-coat: 0.2409 ± 0.0204
0.2279
0.2381
0.2486
alphaRep: 0.2442 ± 0.0104
0.2405
0.2447
0.2482
CP: 0.2532 ± 0.0178
0.2416
0.2529
0.2646
Reg: 0.2673 ± 0.0256
0.2594
0.2759
0.2876
0.000
0.100
0.200
0.300
0.400
0.500

Proportion of Guanine in region 1

Histogram

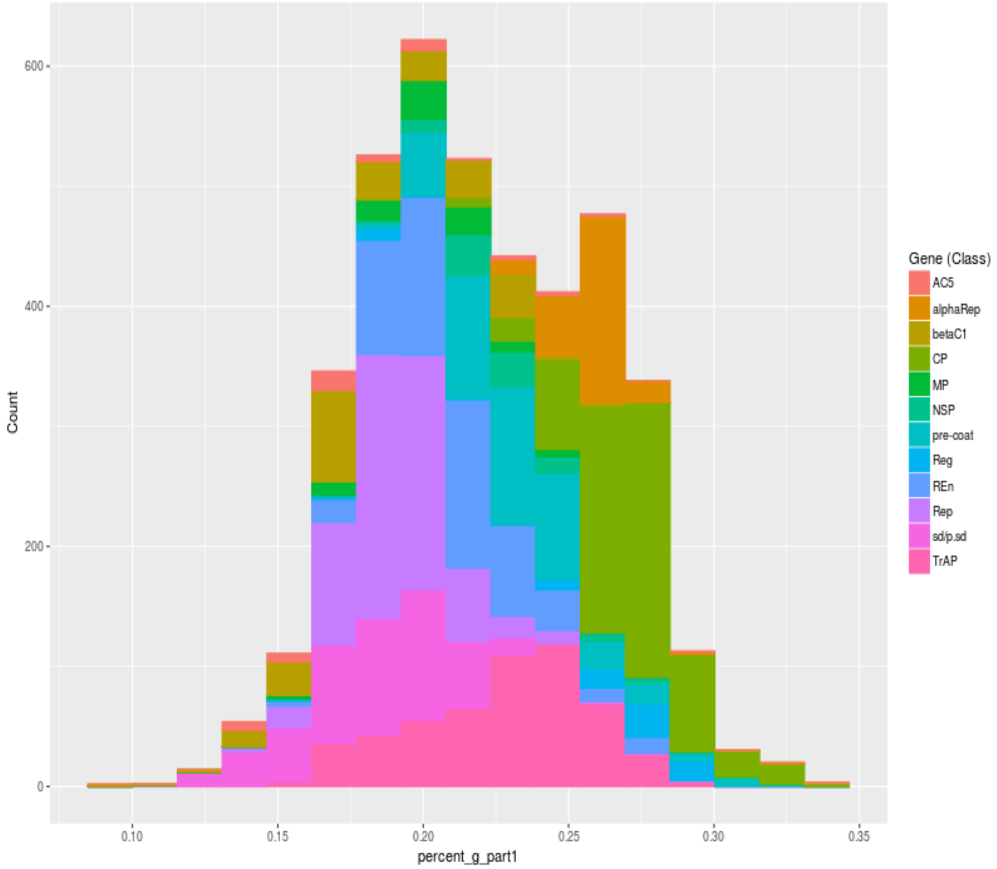

Density

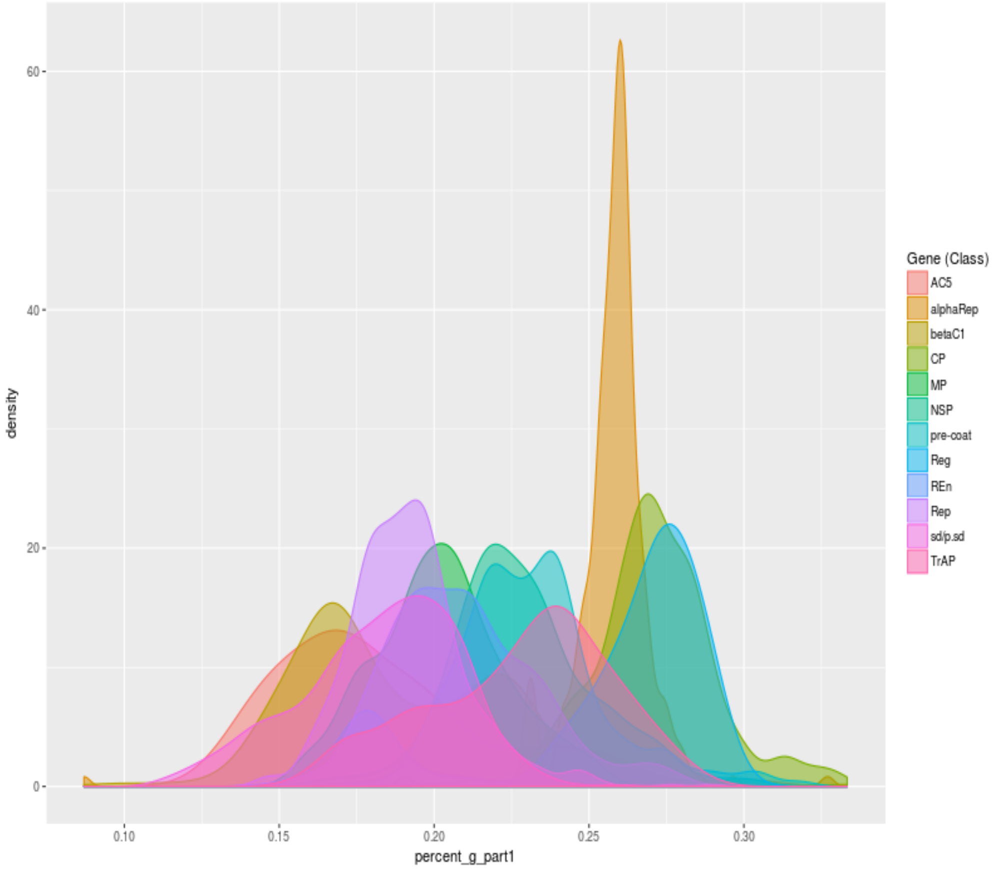

Boxplots

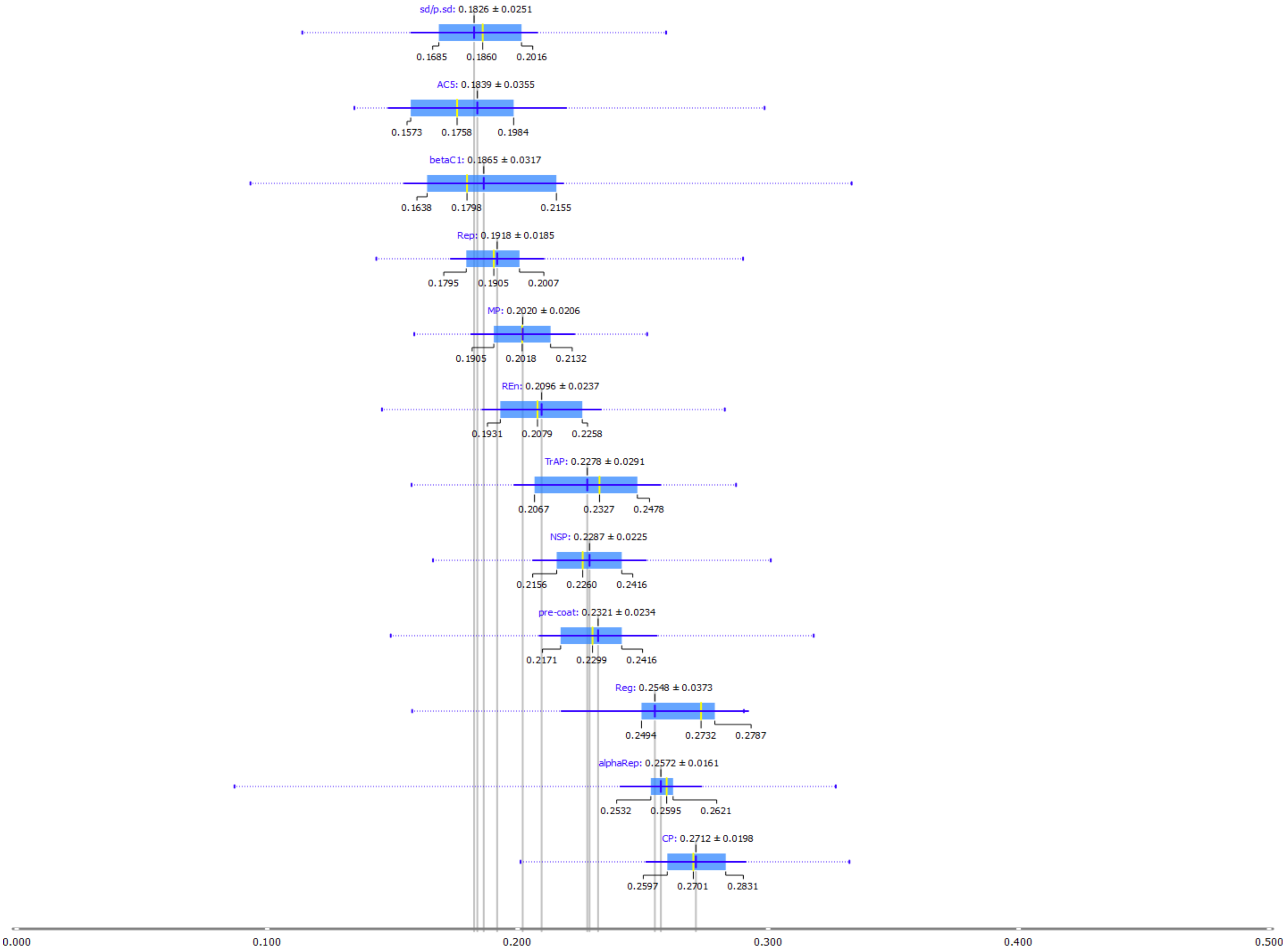

sd/p.sd: 0.1826 ± 0.0251
0.1685 0.1860 0.2016
AC5: 0.1839 ± 0.0355
0.1573 0.1758 0.1984
betaC1: 0.1865 ± 0.0317
0.1638 0.1798 0.2155
Rep: 0.1918 ± 0.0185
0.1795 0.1905 0.2007
MP: 0.2020 ± 0.0206
0.1905 0.2018 0.2132
REn: 0.2096 ± 0.0237
0.1931 0.2079 0.2258
TrAP: 0.2278 ± 0.0291
0.2067 0.2327 0.2478
NSP: 0.2287 ± 0.0225
0.2156 0.2260 0.2416
pre-coat: 0.2321 ± 0.0234
0.2171 0.2299 0.2416
Reg: 0.2548 ± 0.0373
0.2494 0.2732 0.2787
alphaRep: 0.2572 ± 0.0161
0.2532 0.2595 0.2621
CP: 0.2712 ± 0.0198
0.2597 0.2701 0.2831
0.000 0.100 0.200 0.300 0.400 0.500

Proportion of Guanine in region 2

Histogram

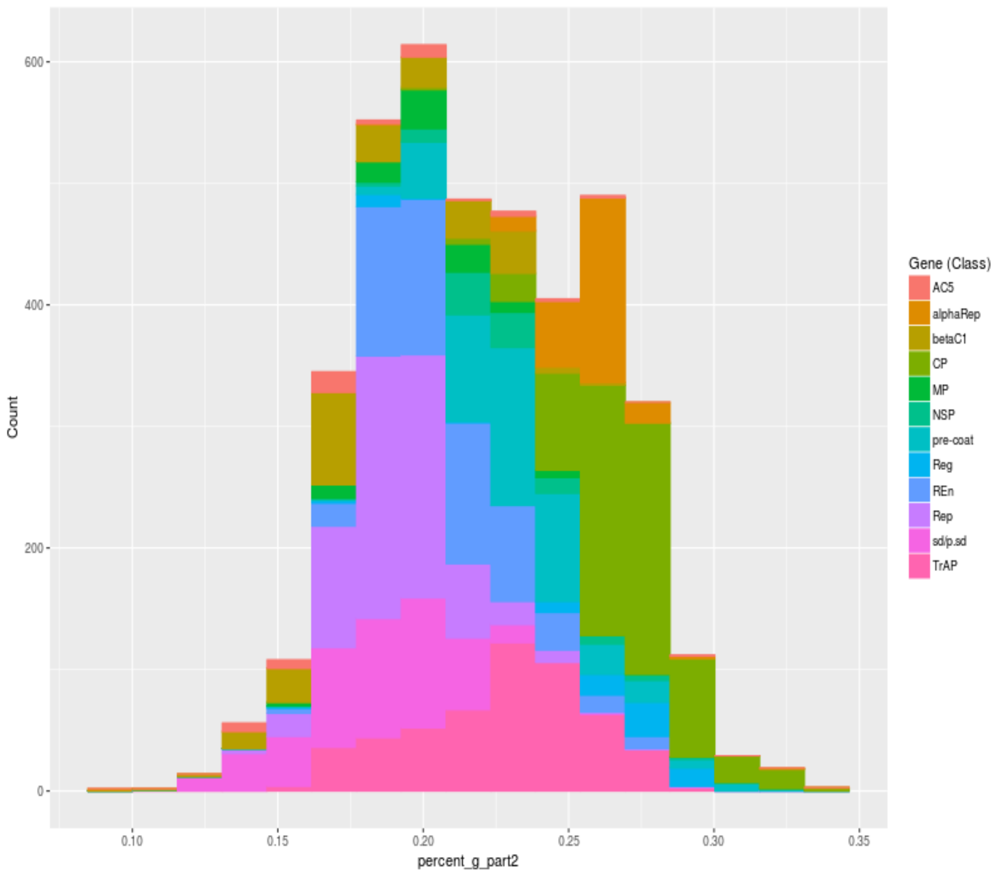

Density

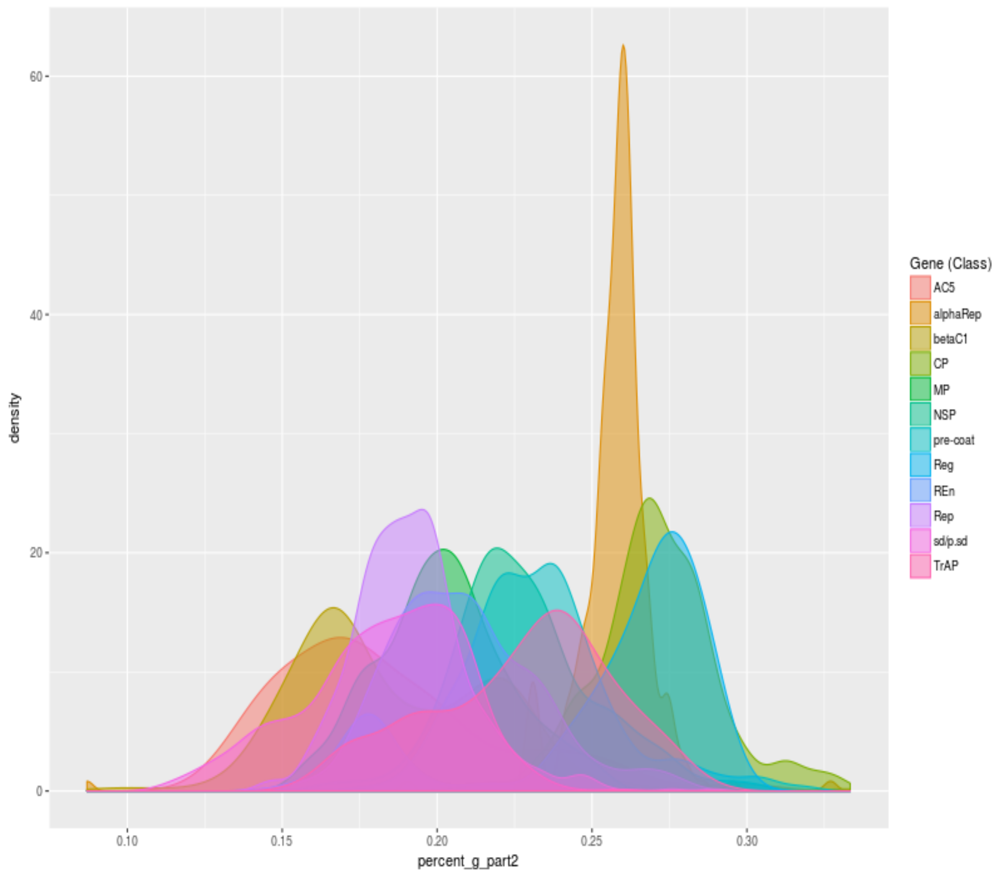

Boxplots

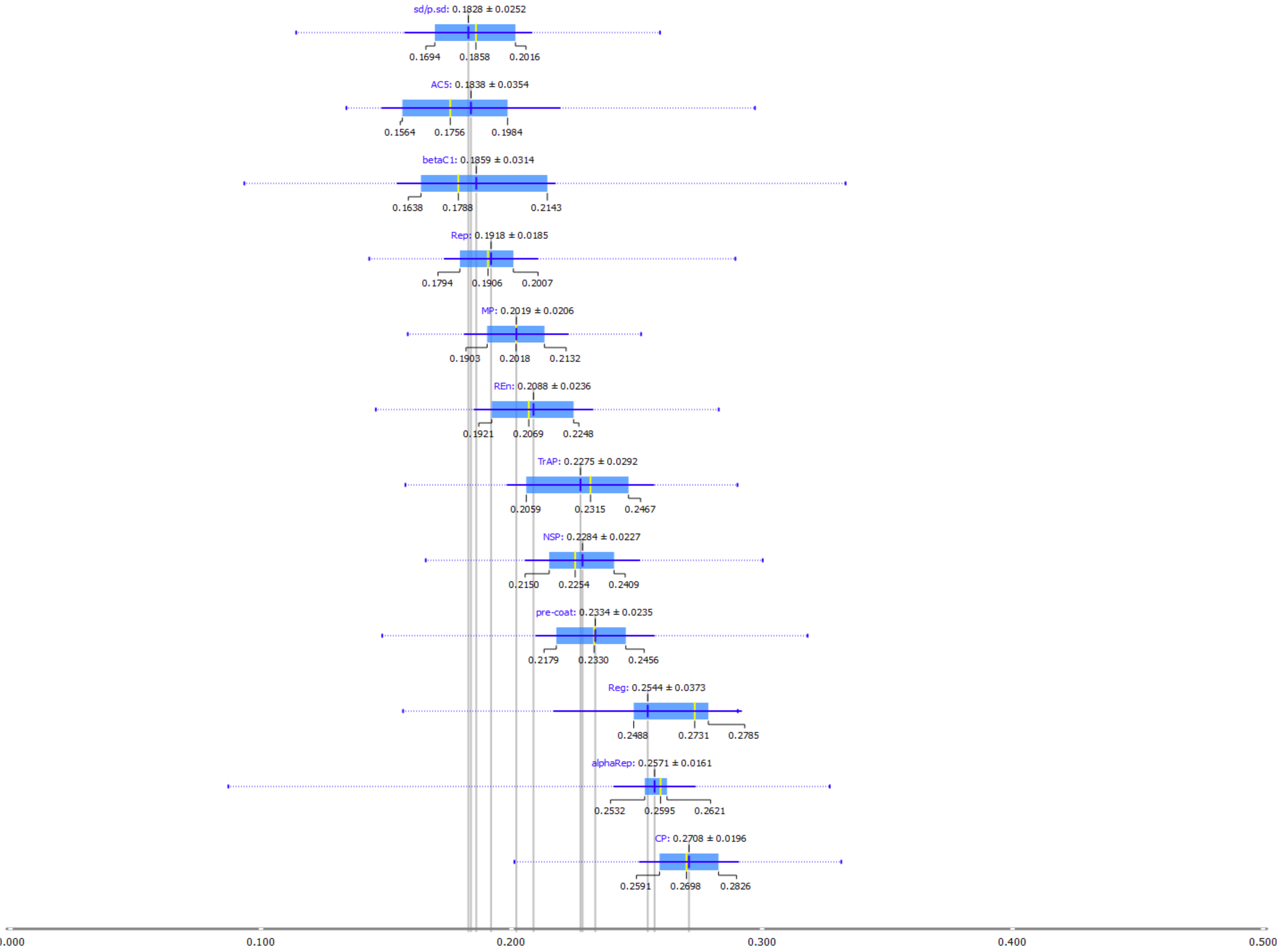

sd/p.sd: 0.1828 ± 0.0252
0.1694
0.1858
0.2016
AC5: 0.1838 ± 0.0354
0.1564
0.1756
0.1984
betaC1: 0.1859 ± 0.0314
0.1638
0.1788
0.2143
Rep: 0.1918 ± 0.0185
0.1794
0.1906
0.2007
MP: 0.2019 ± 0.0206
0.1903
0.2018
0.2132
REn: 0.2088 ± 0.0236
0.1921
0.2069
0.2248
TrAP: 0.2275 ± 0.0292
0.2059
0.2315
0.2467
NSP: 0.2284 ± 0.0227
0.2150
0.2254
0.2409
pre-coat: 0.2334 ± 0.0235
0.2179
0.2330
0.2456
Reg: 0.2544 ± 0.0373
0.2488
0.2731
0.2785
alphaRep: 0.2571 ± 0.0161
0.2532
0.2595
0.2621
CP: 0.2708 ± 0.0196
0.2591
0.2698
0.2826
0.000
0.100
0.200
0.300
0.400
0.500

Proportion of nucleotides regarding the genome (ORF_size / genome_size)

Histogram

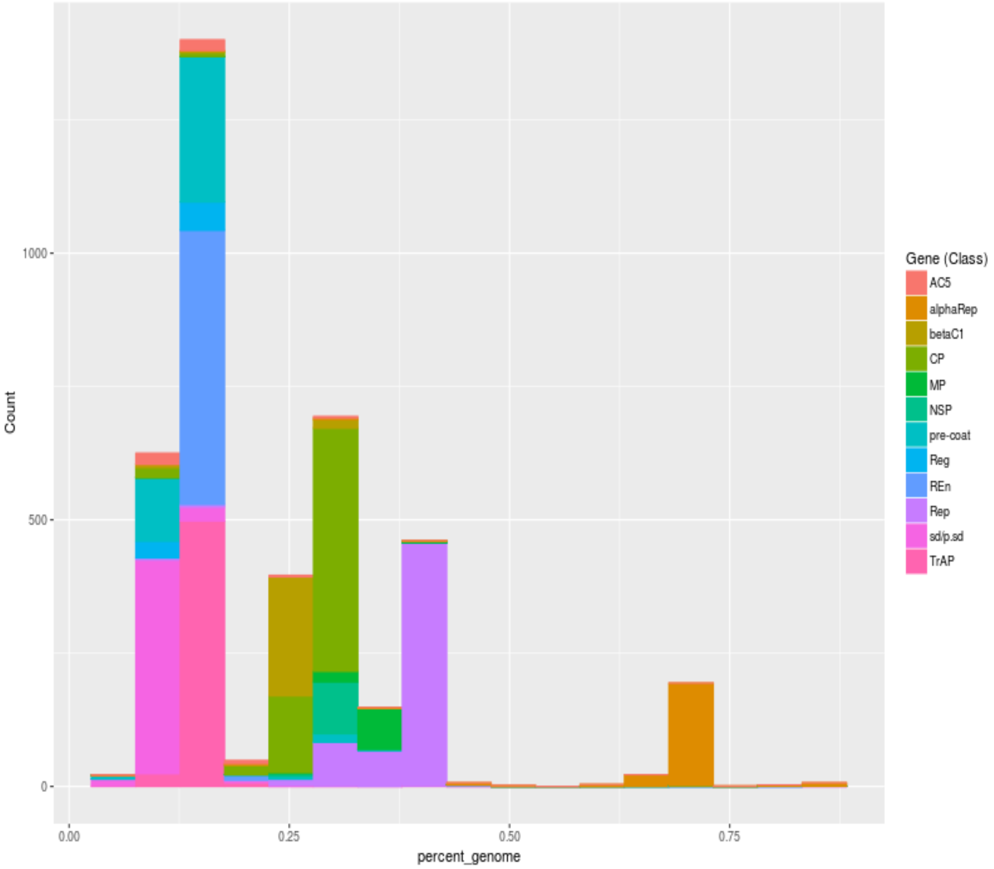

Density

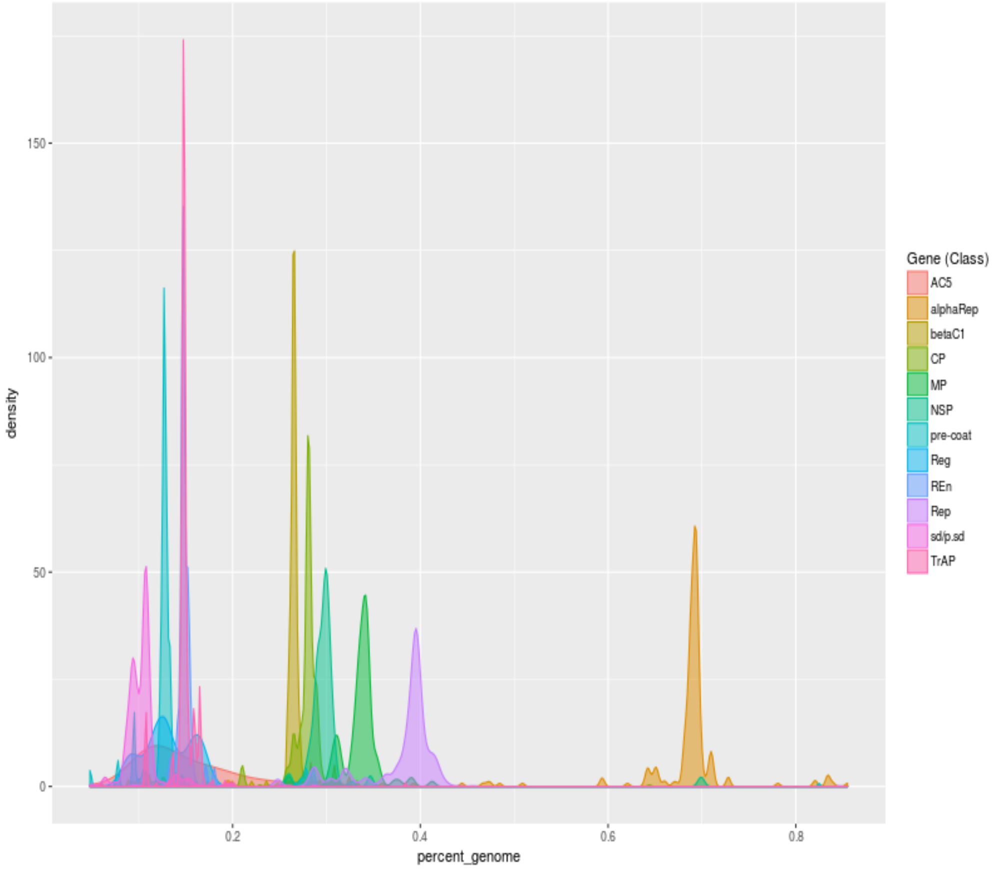

Boxplots

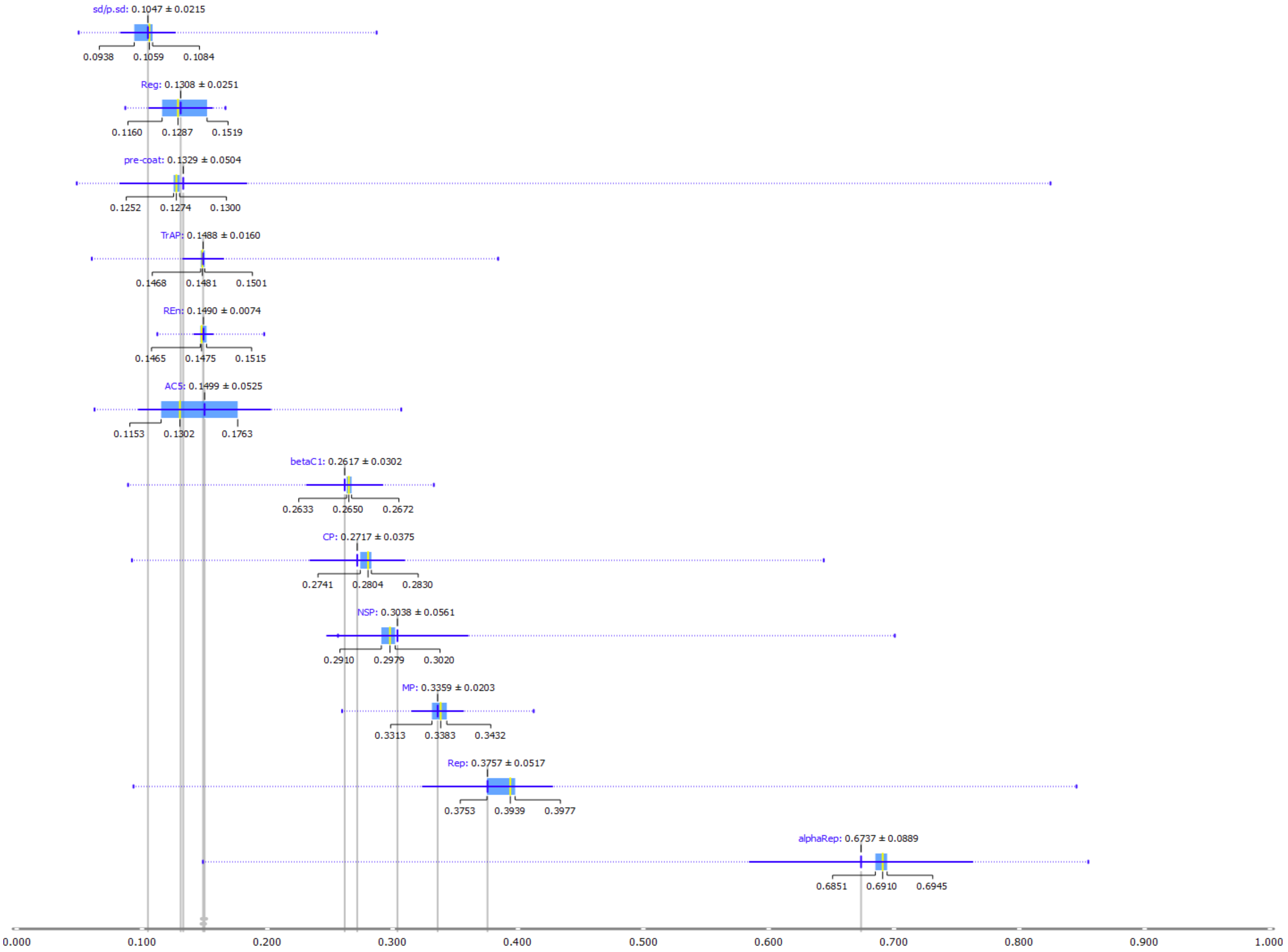

sd/p.sd: 0.1047 ± 0.0215
0.0938
0.1059
0.1084
Reg: 0.1308 ± 0.0251
0.1160
0.1287
0.1519
pre-coat: 0.1329 ± 0.0504
0.1252
0.1274
0.1300
TrAP: 0.1488 ± 0.0160
0.1468
0.1481
0.1501
REn: 0.1490 ± 0.0074
0.1465
0.1475
0.1515
ACS: 0.1499 ± 0.0525
0.1153
0.1302
0.1763
betaC1: 0.2617 ± 0.0302
0.2633
0.2650
0.2672
CP: 0.2717 ± 0.0375
0.2741
0.2804
0.2830
NSP: 0.3038 ± 0.0561
0.2910
0.2979
0.3020
MP: 0.3359 ± 0.0203
0.3313
0.3383
0.3432
Rep: 0.3757 ± 0.0517
0.3753
0.3939
0.3977
alphaRep: 0.6737 ± 0.0889
0.6851
0.6910
0.6945
0.000
0.100
0.200
0.300
0.400
0.500
0.600
0.700
0.800
0.900
1.000

Proportion of Thymine

Histogram

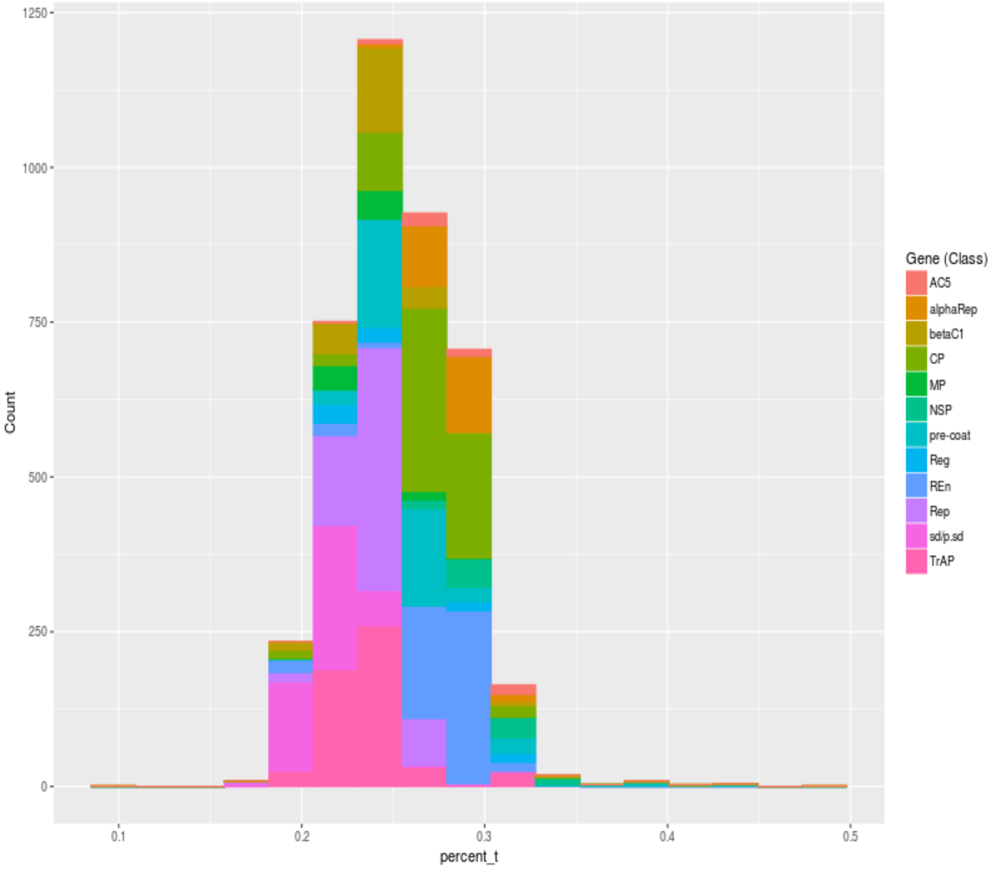

Density

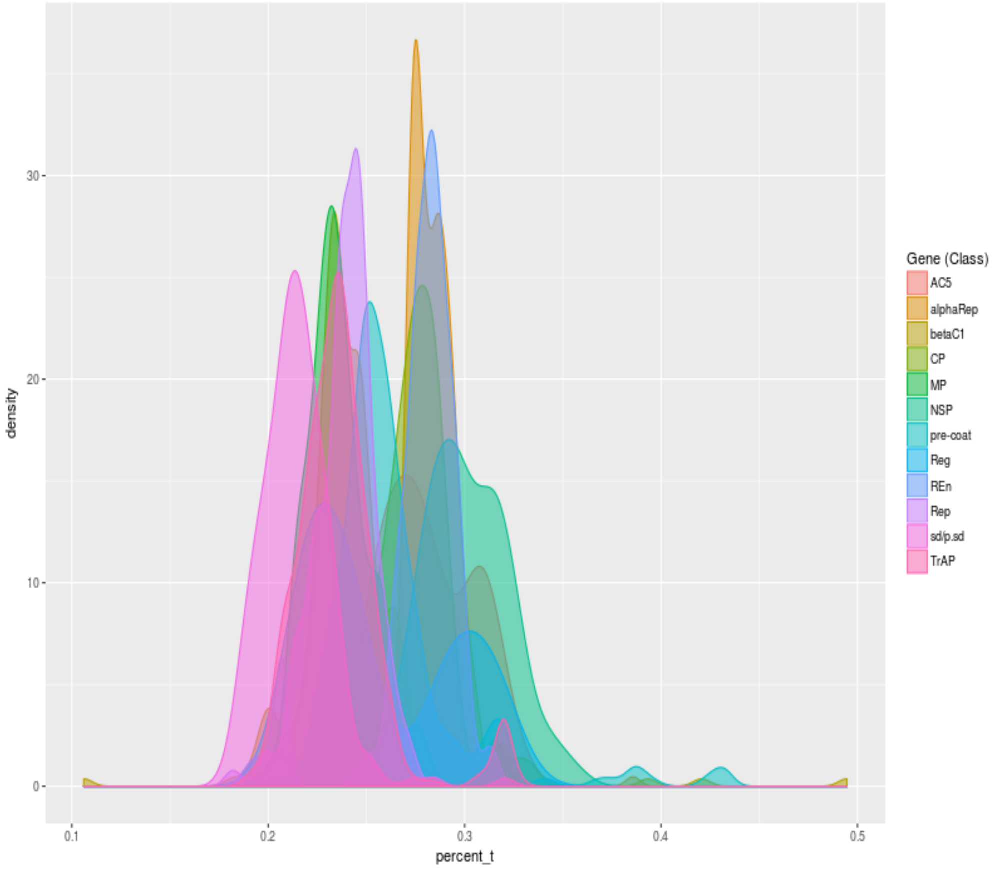

Boxplots

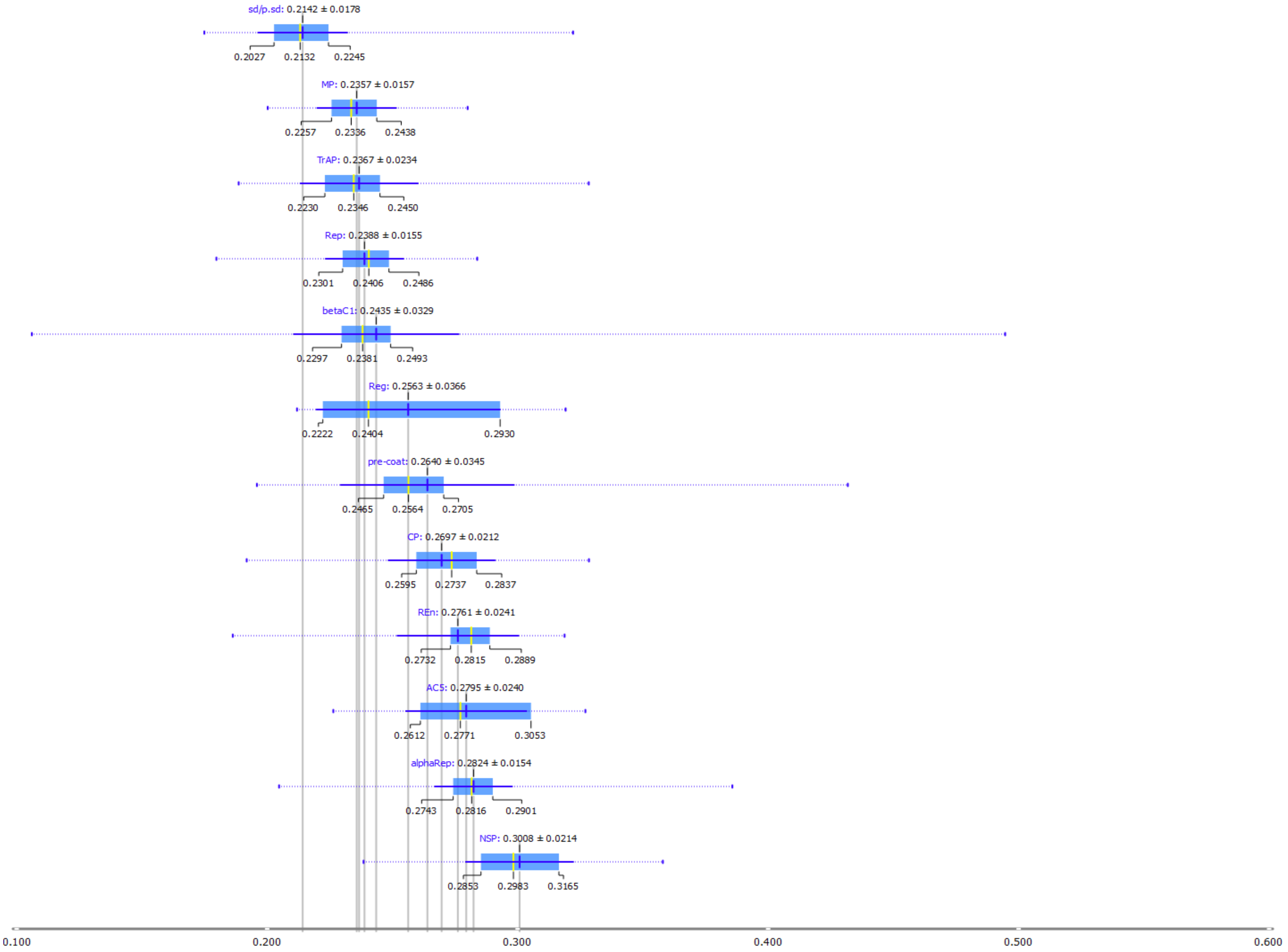

sd/p.sd: 0.2142 ± 0.0178
0.2027 0.2132 0.2245
MP: 0.2357 ± 0.0157
0.2257 0.2336 0.2438
TrAP: 0.2367 ± 0.0234
0.2230 0.2346 0.2450
Rep: 0.2388 ± 0.0155
0.2301 0.2406 0.2486
betaC1: 0.2435 ± 0.0329
0.2297 0.2381 0.2493
Reg: 0.2563 ± 0.0366
0.2222 0.2404 0.2930
pre-coat: 0.2640 ± 0.0345
0.2465 0.2564 0.2705
CP: 0.2697 ± 0.0212
0.2595 0.2737 0.2837
REn: 0.2761 ± 0.0241
0.2732 0.2815 0.2889
AC5: 0.2795 ± 0.0240
0.2612 0.2771 0.3053
alphaRep: 0.2824 ± 0.0154
0.2743 0.2816 0.2901
NSP: 0.3008 ± 0.0214
0.2853 0.2983 0.3165
0.100 0.200 0.300 0.400 0.500 0.600

Proportion of Thymine in region 1

Histogram

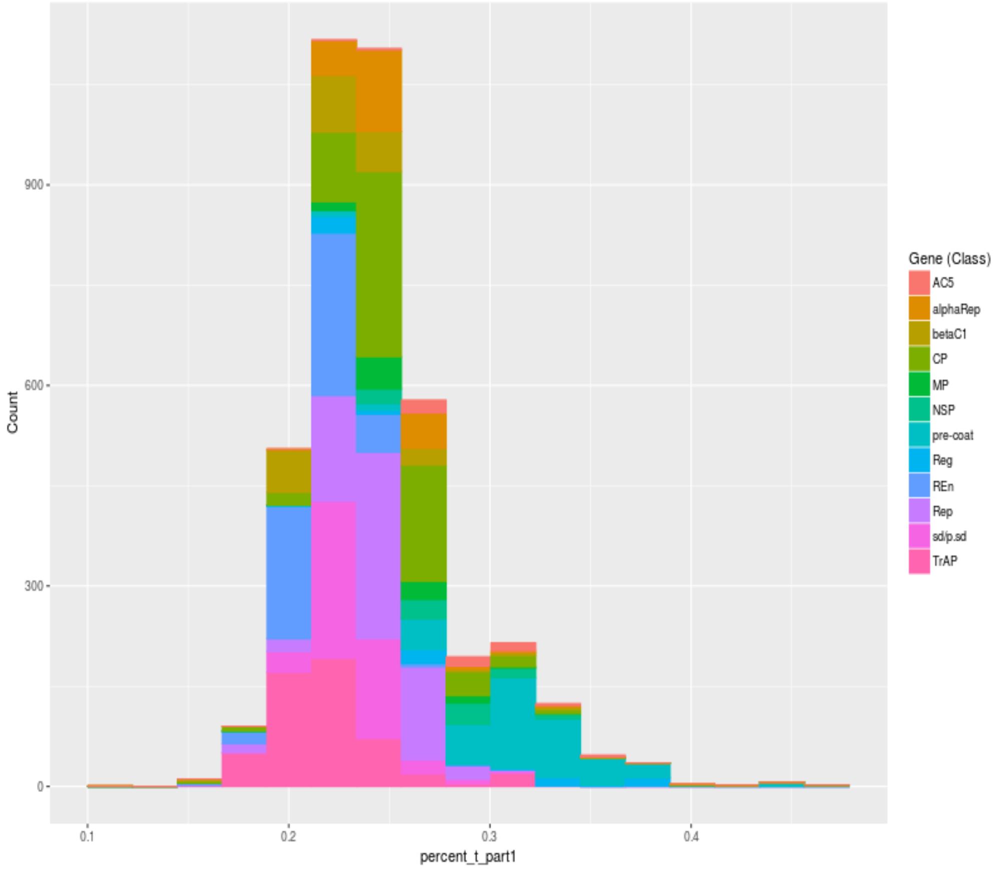

Density

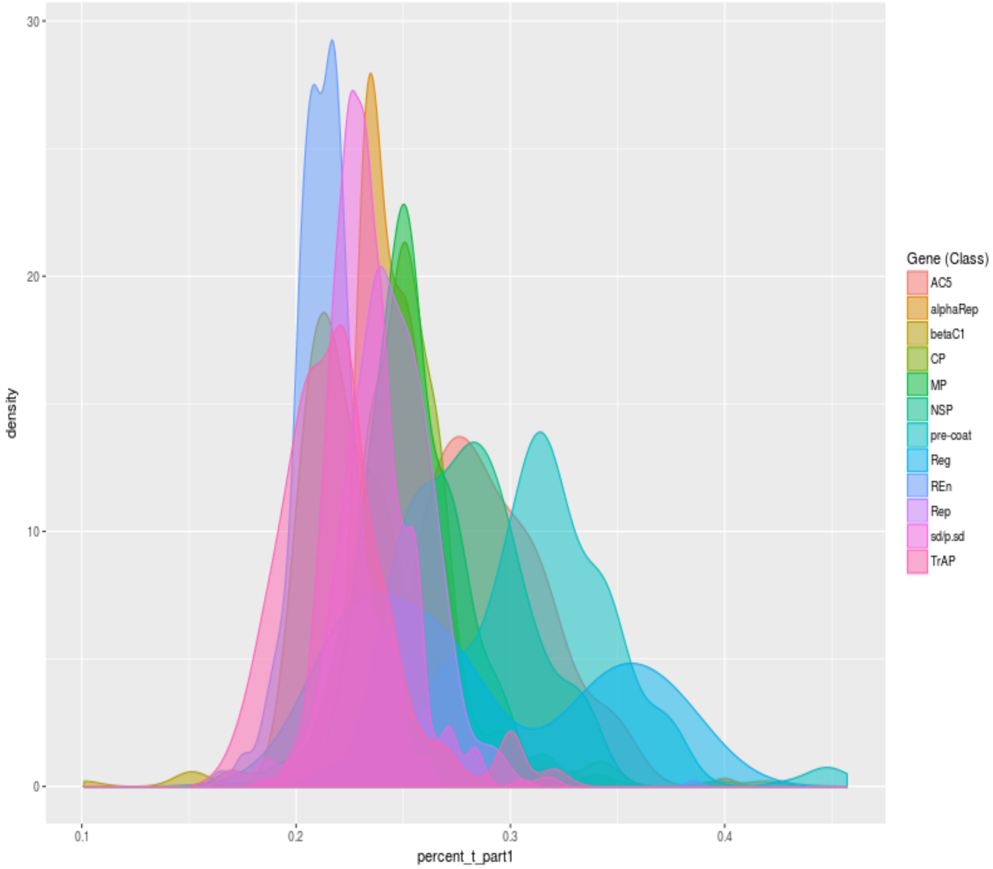

Boxplots

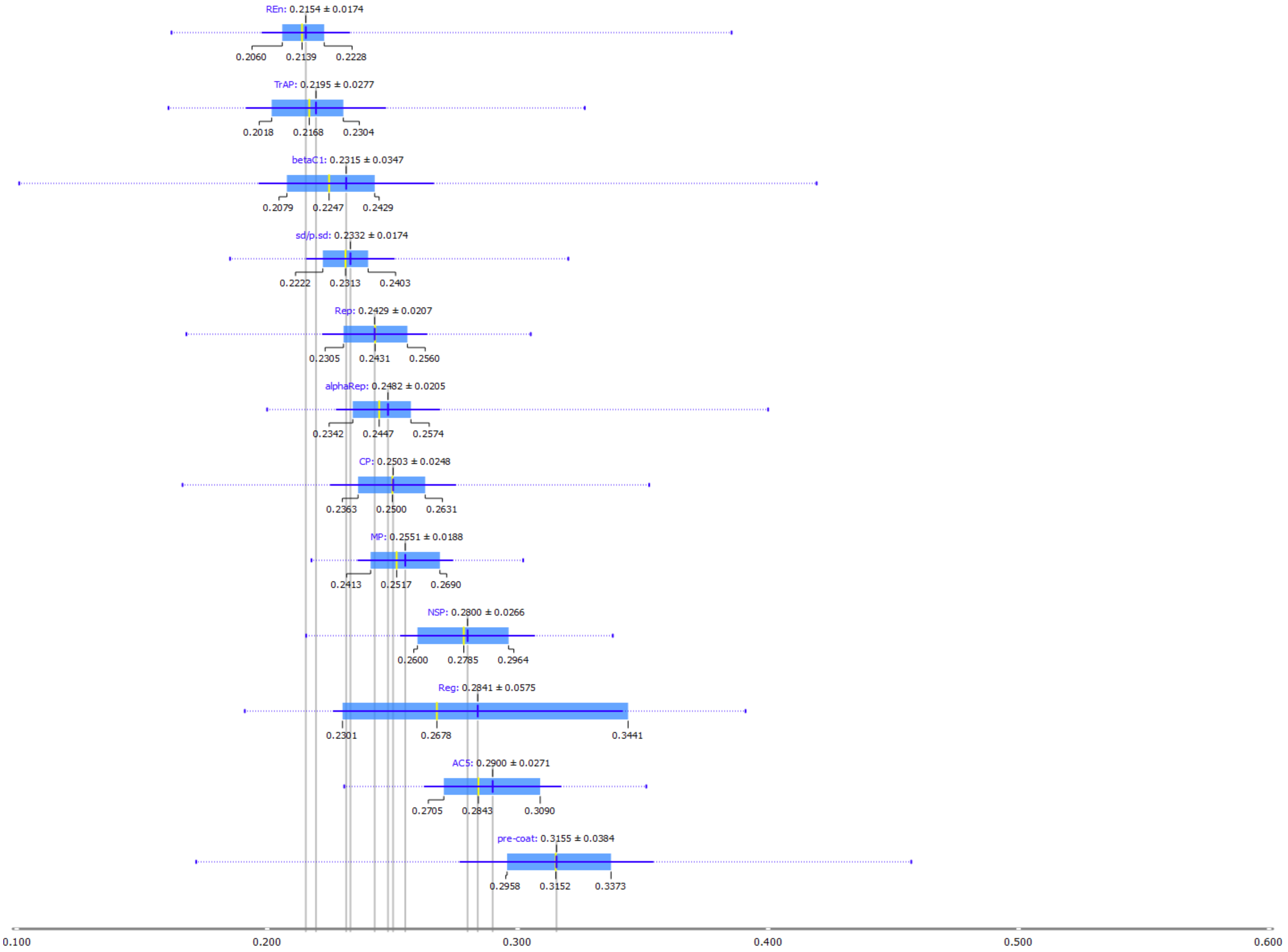

REn: 0.2154 ± 0.0174
0.2060 0.2139 0.2228
TrAP: 0.2195 ± 0.0277
0.2018 0.2168 0.2304
betaC1: 0.2315 ± 0.0347
0.2079 0.2247 0.2429
sd/p.sd: 0.2332 ± 0.0174
0.2222 0.2313 0.2403
Rep: 0.2429 ± 0.0207
0.2305 0.2431 0.2560
alphaRep: 0.2482 ± 0.0205
0.2342 0.2447 0.2574
CP: 0.2503 ± 0.0248
0.2363 0.2500 0.2631
MP: 0.2551 ± 0.0188
0.2413 0.2517 0.2690
NSP: 0.2800 ± 0.0266
0.2600 0.2785 0.2964
Reg: 0.2841 ± 0.0575
0.2301 0.2678 0.3441
AC5: 0.2900 ± 0.0271
0.2705 0.2843 0.3090
pre-coat: 0.3155 ± 0.0384
0.2958 0.3152 0.3373
0.100
0.200
0.300
0.400
0.500
0.600

Proportion of Thymine in region 2

Histogram

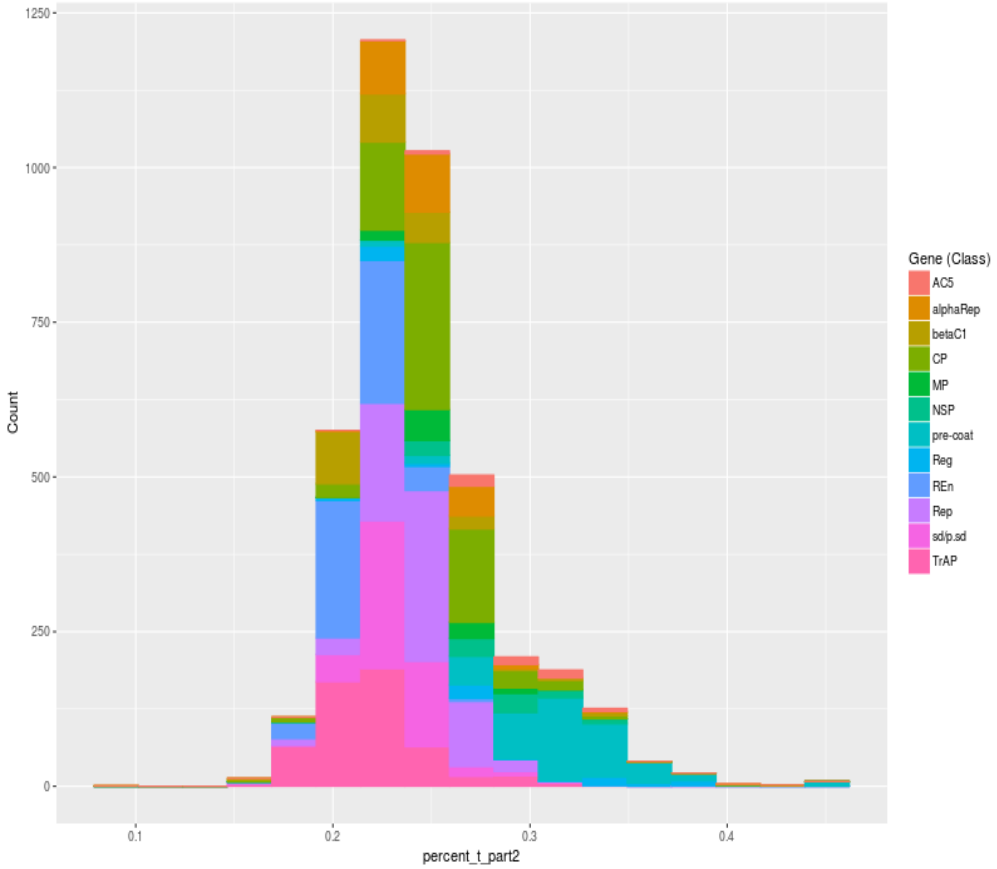

Density

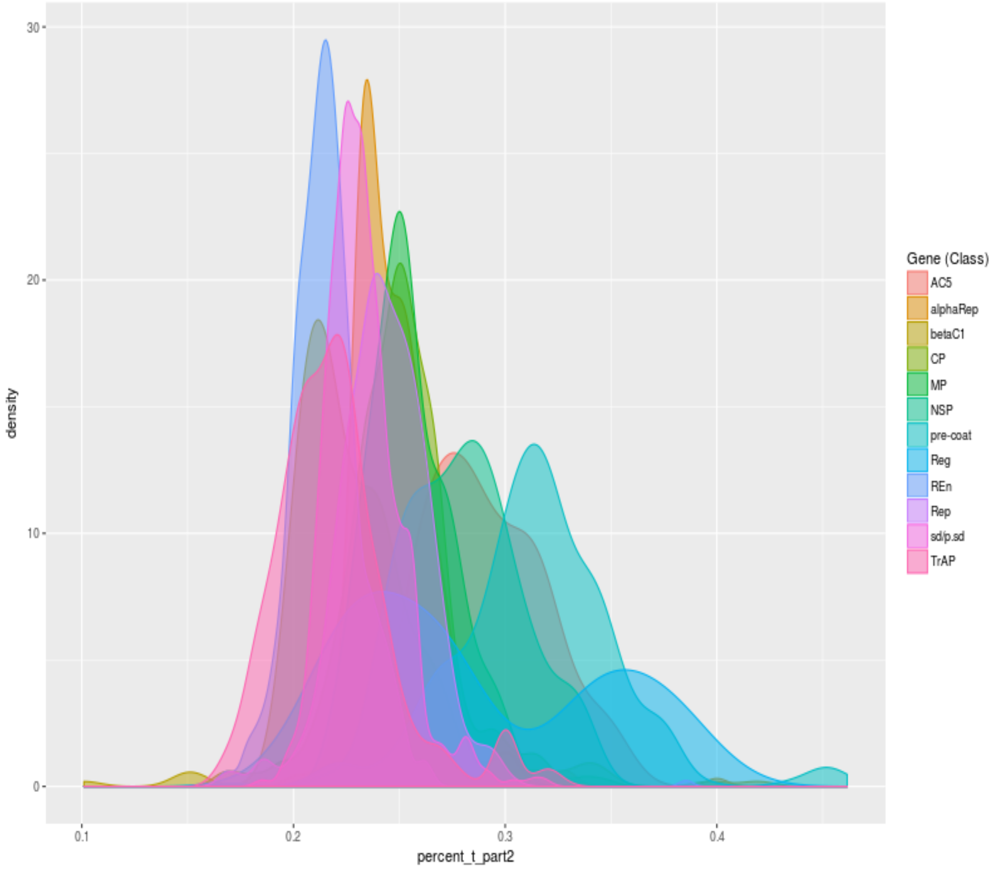

Boxplots

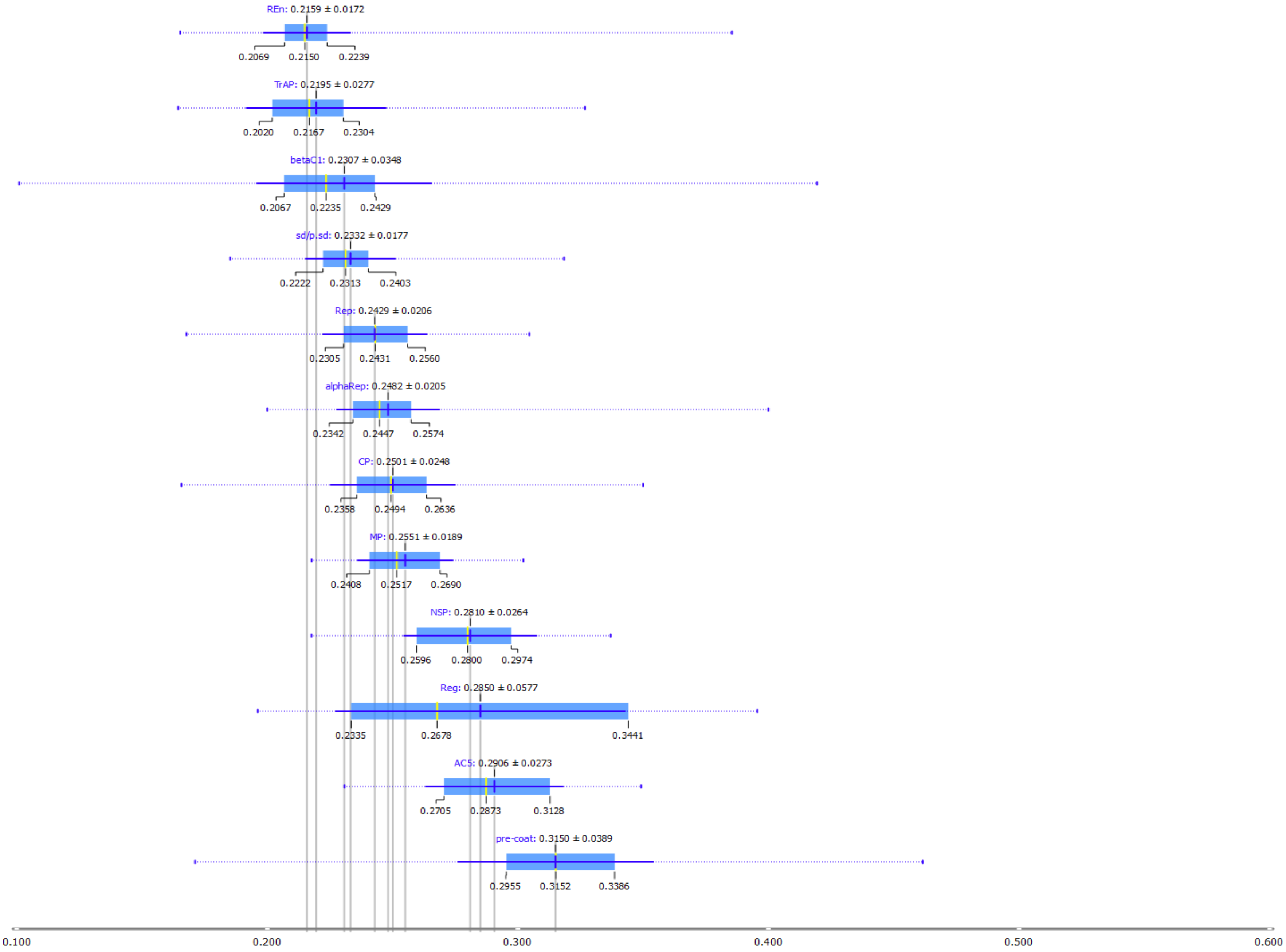

REn: 0.2159 ± 0.0172
0.2069
0.2150
0.2239
TrAP: 0.2195 ± 0.0277
0.2020
0.2167
0.2304
betaC1: 0.2307 ± 0.0348
0.2067
0.2235
0.2429
sd/p.sd: 0.2332 ± 0.0177
0.2222
0.2313
0.2403
Rep: 0.2429 ± 0.0206
0.2305
0.2431
0.2560
alphaRep: 0.2482 ± 0.0205
0.2342
0.2447
0.2574
CP: 0.2501 ± 0.0248
0.2358
0.2494
0.2636
MP: 0.2551 ± 0.0189
0.2408
0.2517
0.2690
NSP: 0.2810 ± 0.0264
0.2596
0.2800
0.2974
Reg: 0.2850 ± 0.0577
0.2335
0.2678
0.3441
AC5: 0.2906 ± 0.0273
0.2705
0.2873
0.3128
pre-coat: 0.3150 ± 0.0389
0.2955
0.3152
0.3386
0.100
0.200
0.300
0.400
0.500
0.600

Proportion of Phenylalanine

Histogram

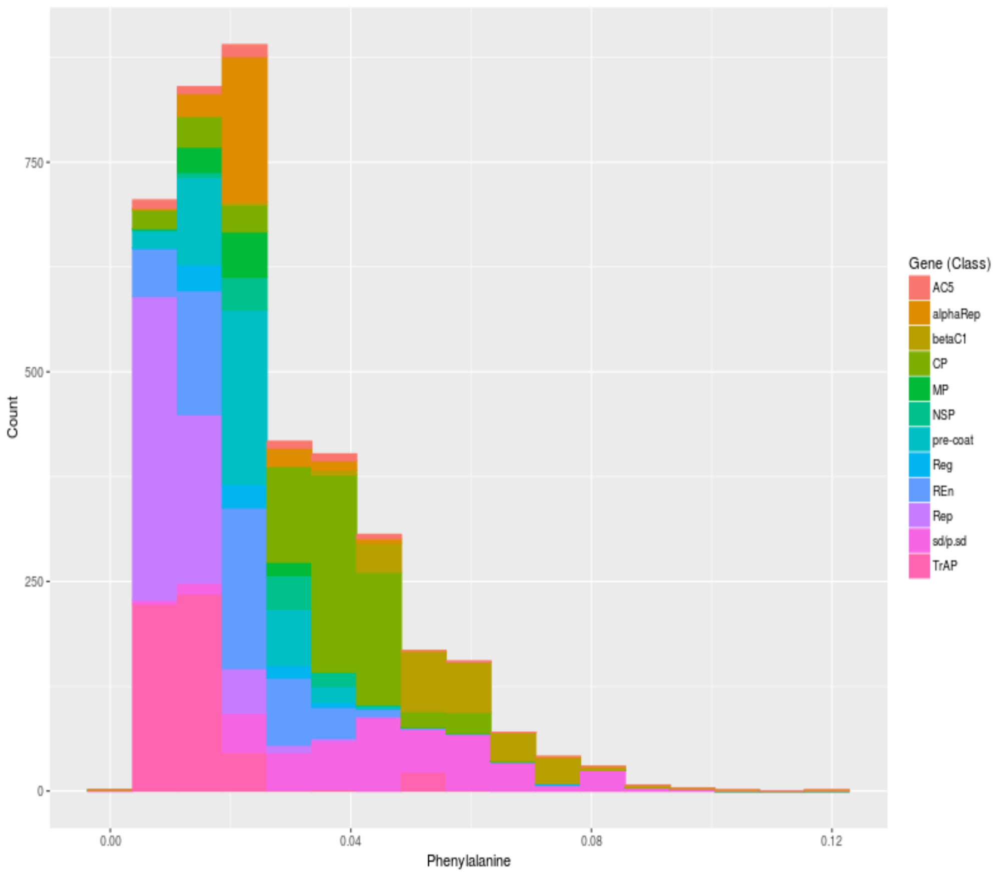

Density

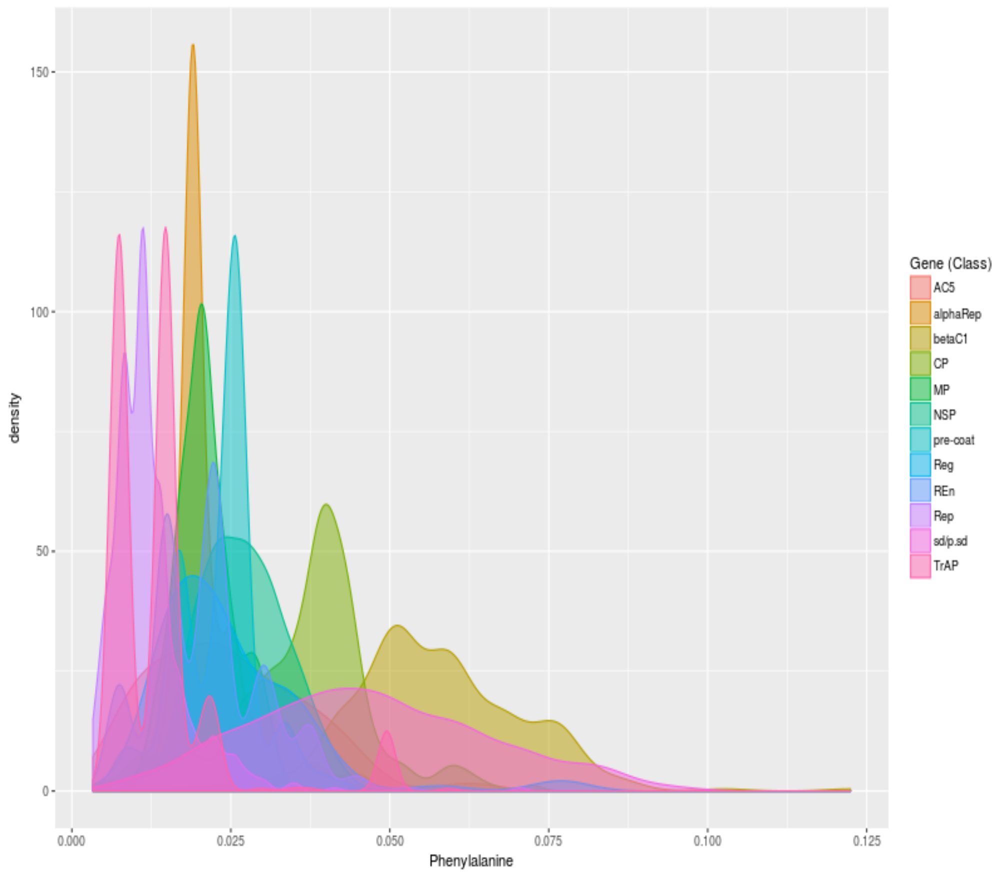

Boxplots

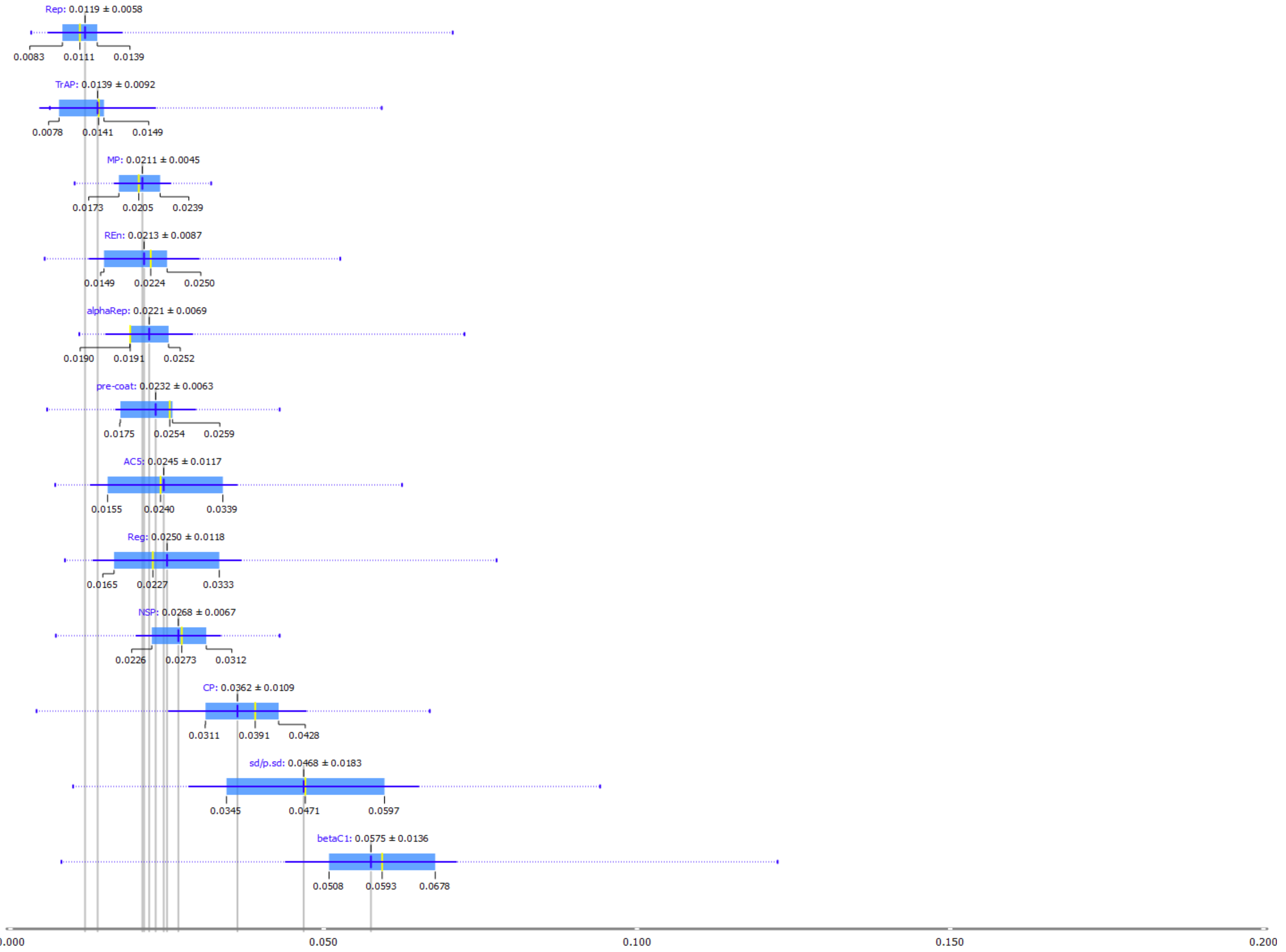
Rep: 0.0119 ± 0.0058
0.0083
0.0111
0.0139
TrAP: 0.0139 ± 0.0092
0.0078
0.0141
0.0149
MP: 0.0211 ± 0.0045
0.0173
0.0205
0.0239
REn: 0.0213 ± 0.0087
0.0149
0.0224
0.0250
alphaRep: 0.0221 ± 0.0069
0.0190
0.0191
0.0252
pre-coat: 0.0232 ± 0.0063
0.0175
0.0254
0.0259
AC5: 0.0245 ± 0.0117
0.0155
0.0240
0.0339
Reg: 0.0250 ± 0.0118
0.0165
0.0227
0.0333
NSP: 0.0268 ± 0.0067
0.0226
0.0273
0.0312
CP: 0.0362 ± 0.0109
0.0311
0.0391
0.0428
sd/p.sd: 0.0468 ± 0.0183
0.0345
0.0471
0.0597
betaC1: 0.0575 ± 0.0136
0.0508
0.0593
0.0678
0.000
0.050
0.100
0.150
0.200

Proportion of Proline

Histogram

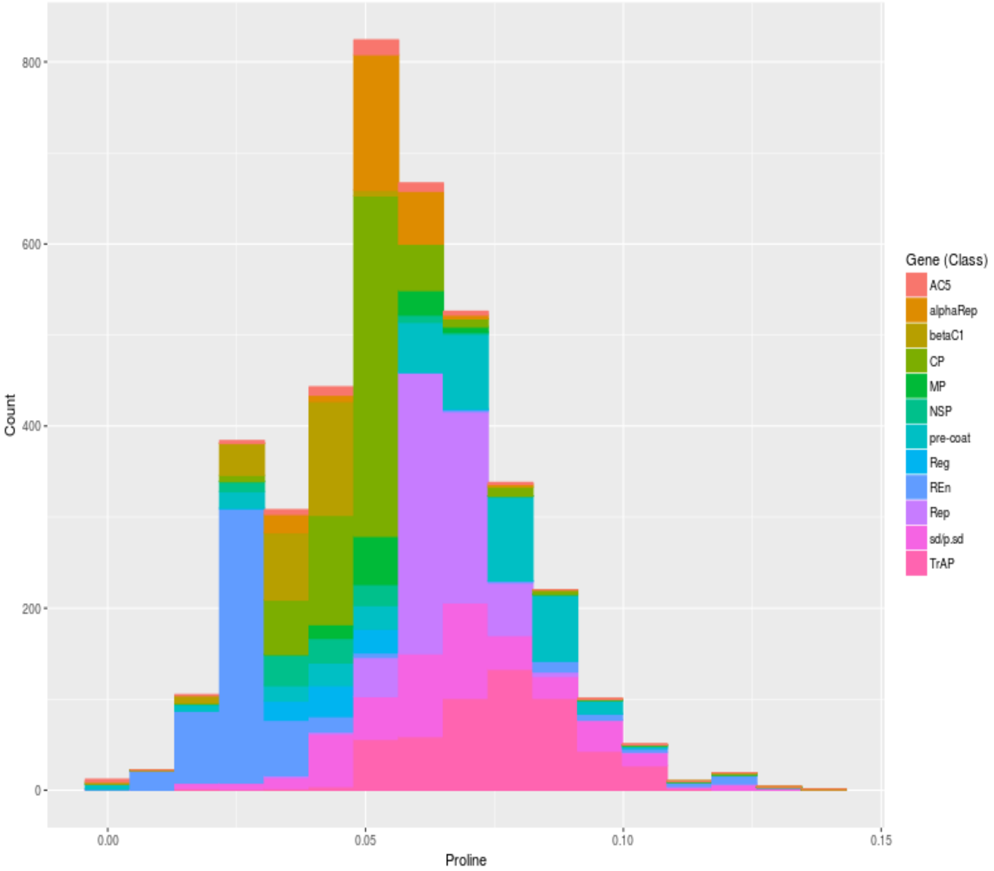

Density

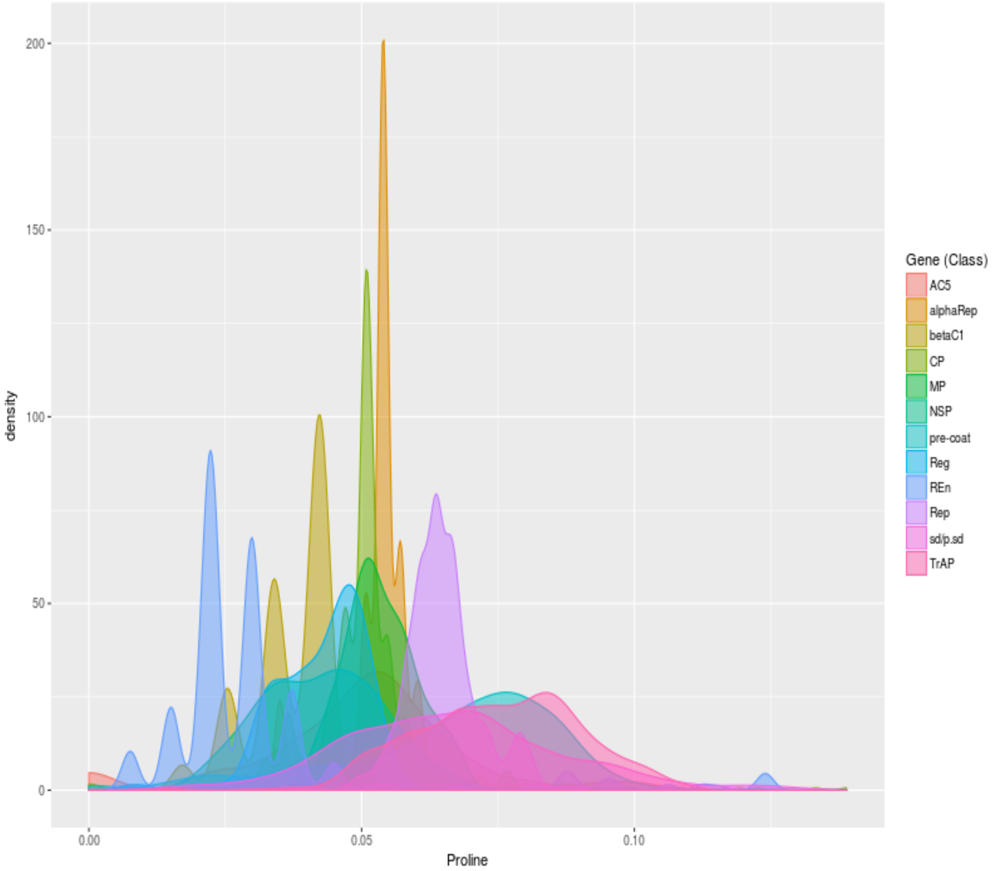

Boxplots

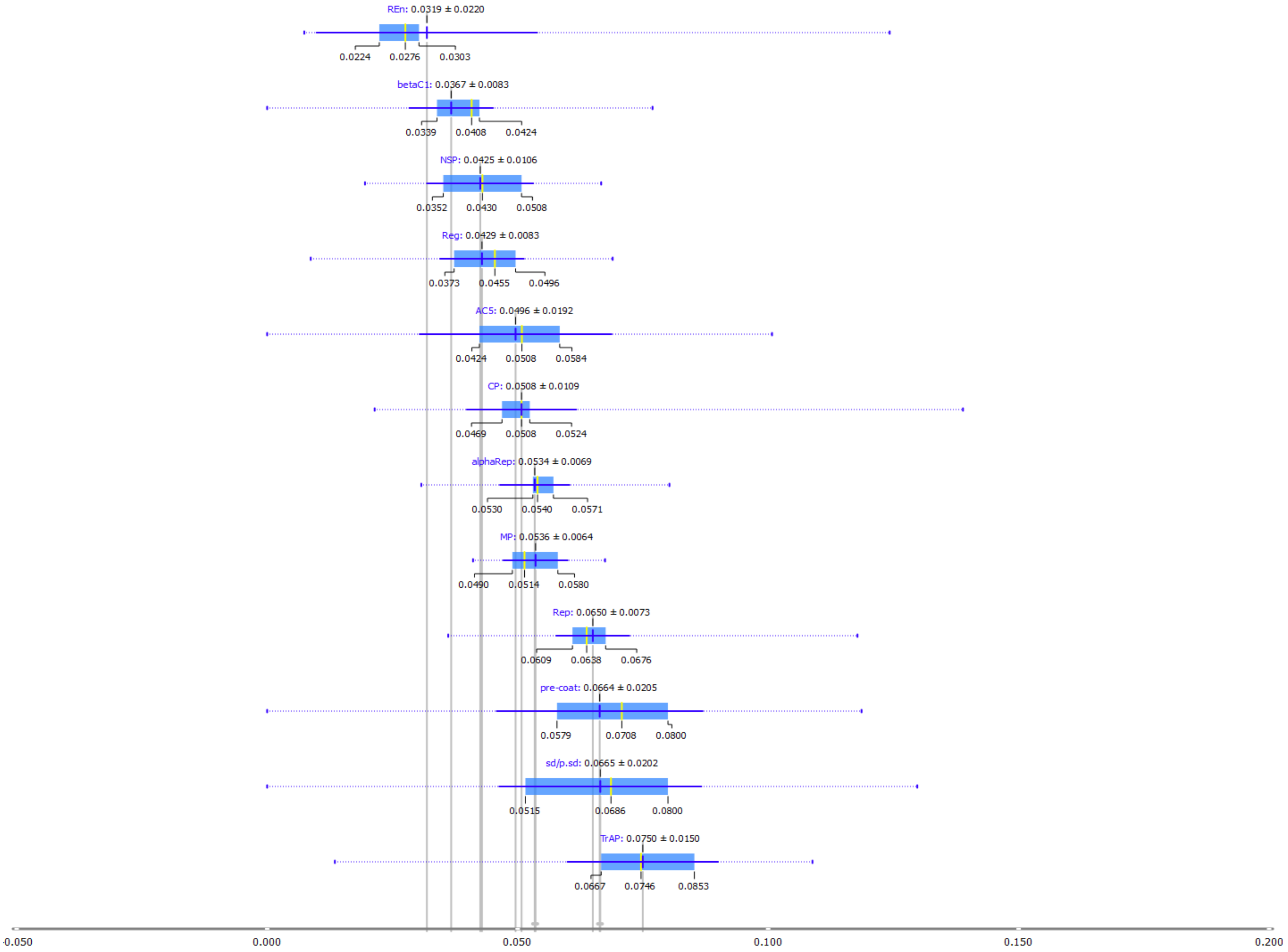

REn: 0.0319 ± 0.0220
0.0224
0.0276
0.0303
betaC1: 0.0367 ± 0.0083
0.0339
0.0408
0.0424
NSP: 0.0425 ± 0.0106
0.0352
0.0430
0.0508
Reg: 0.0429 ± 0.0083
0.0373
0.0455
0.0496
AC5: 0.0496 ± 0.0192
0.0424
0.0508
0.0584
CP: 0.0508 ± 0.0109
0.0469
0.0508
0.0524
alphaRep: 0.0534 ± 0.0069
0.0530
0.0540
0.0571
MP: 0.0536 ± 0.0064
0.0490
0.0514
0.0580
Rep: 0.0650 ± 0.0073
0.0609
0.0638
0.0676
pre-coat: 0.0664 ± 0.0205
0.0579
0.0708
0.0800
sd/p.sd: 0.0665 ± 0.0202
0.0515
0.0686
0.0800
TrAP: 0.0750 ± 0.0150
0.0667
0.0746
0.0853
-0.050
0.000
0.050
0.100
0.150
0.200

Proportion of Serine

Histogram

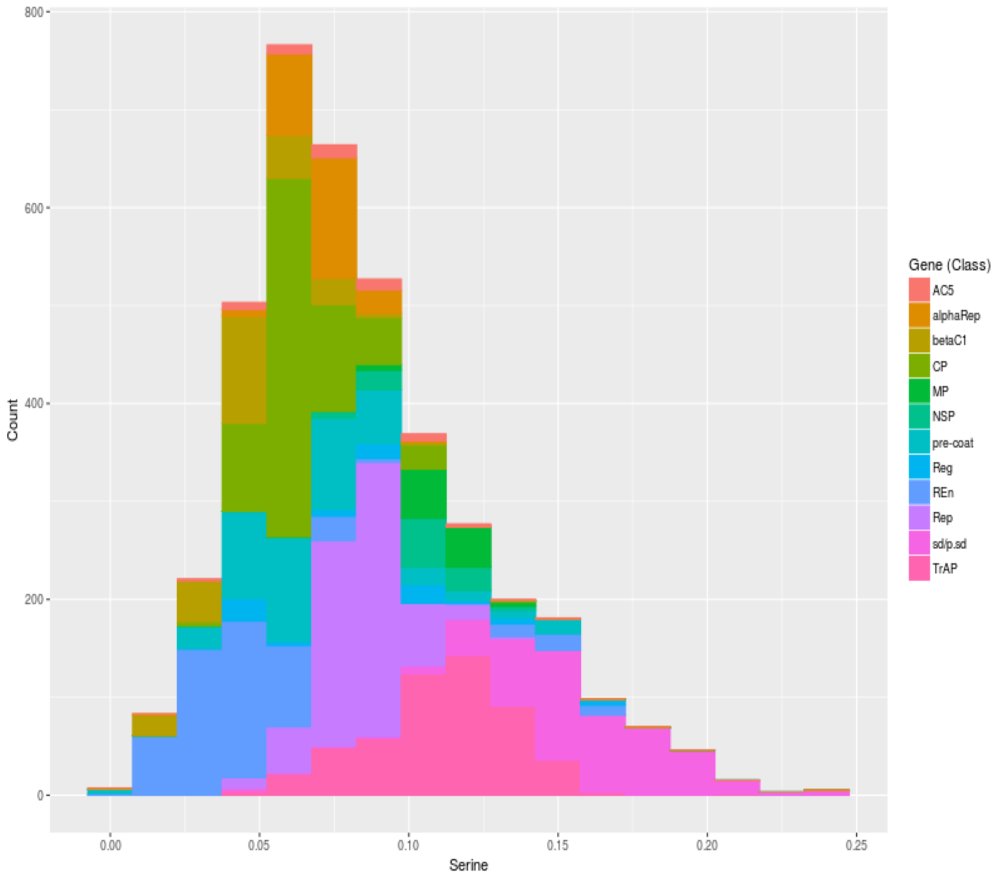

Density

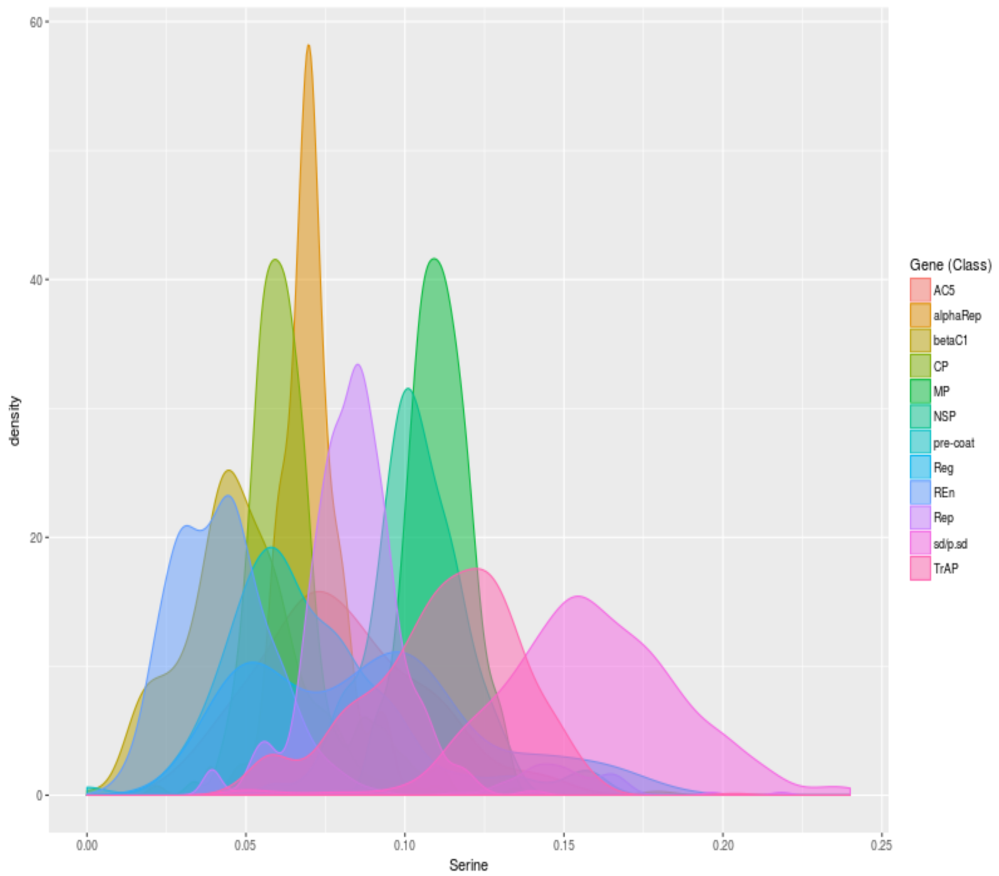

Boxplots

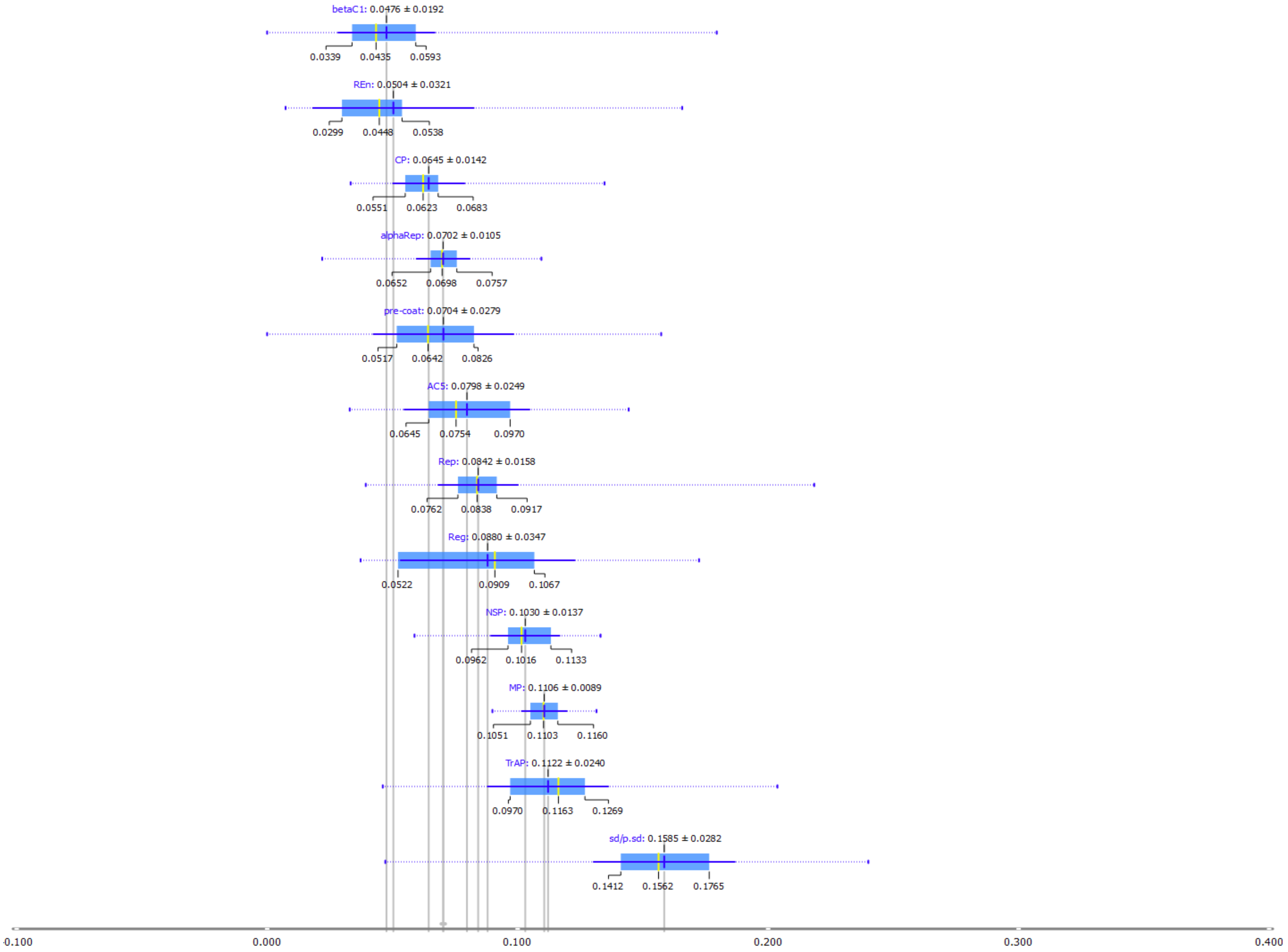
betaC1: 0.0476 ± 0.0192
0.0339
0.0435
0.0593
REn: 0.0504 ± 0.0321
0.0299
0.0448
0.0538
CP: 0.0645 ± 0.0142
0.0551
0.0623
0.0683
alphaRep: 0.0702 ± 0.0105
0.0652
0.0698
0.0757
pre-coat: 0.0704 ± 0.0279
0.0517
0.0642
0.0826
AC5: 0.0798 ± 0.0249
0.0645
0.0754
0.0970
Rep: 0.0842 ± 0.0158
0.0762
0.0838
0.0917
Reg: 0.0880 ± 0.0347
0.0522
0.0909
0.1067
NSP: 0.1030 ± 0.0137
0.0962
0.1016
0.1133
MP: 0.1106 ± 0.0089
0.1051
0.1103
0.1160
TrAP: 0.1122 ± 0.0240
0.0970
0.1163
0.1269
sd/p.sd: 0.1585 ± 0.0282
0.1412
0.1562
0.1765
-0.100
0.000
0.100
0.200
0.300
0.400

Proportion of Threonine

Histogram

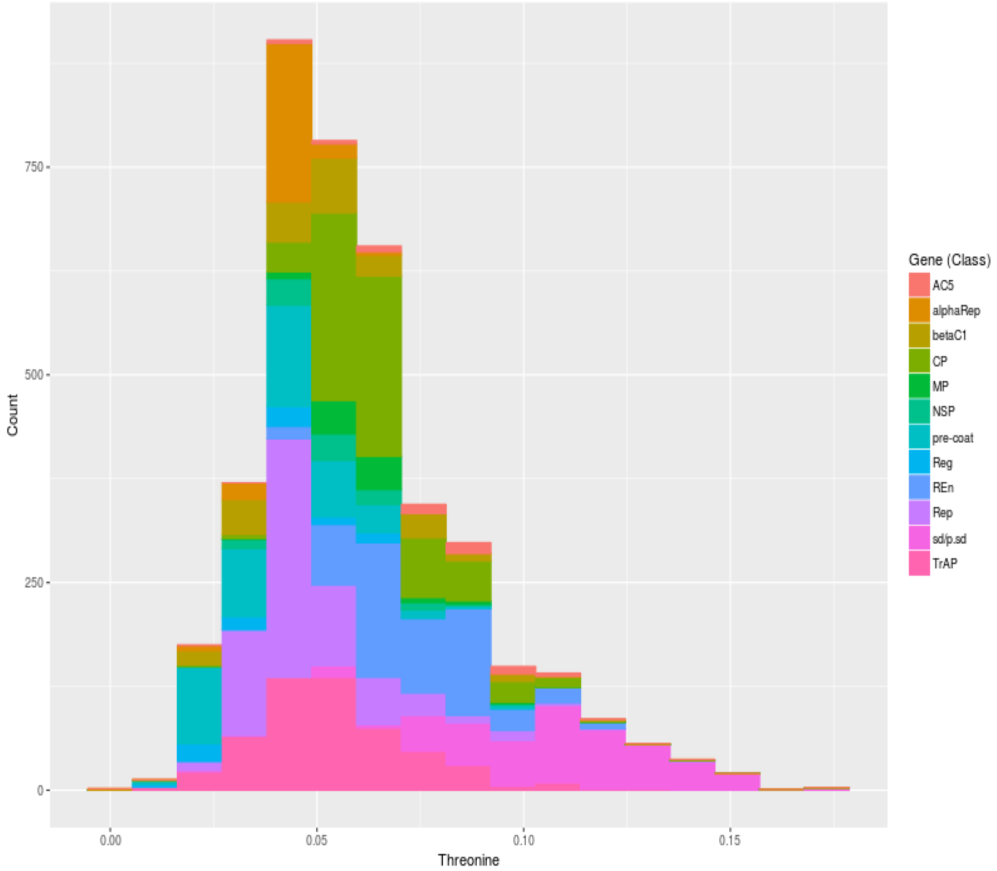

Density

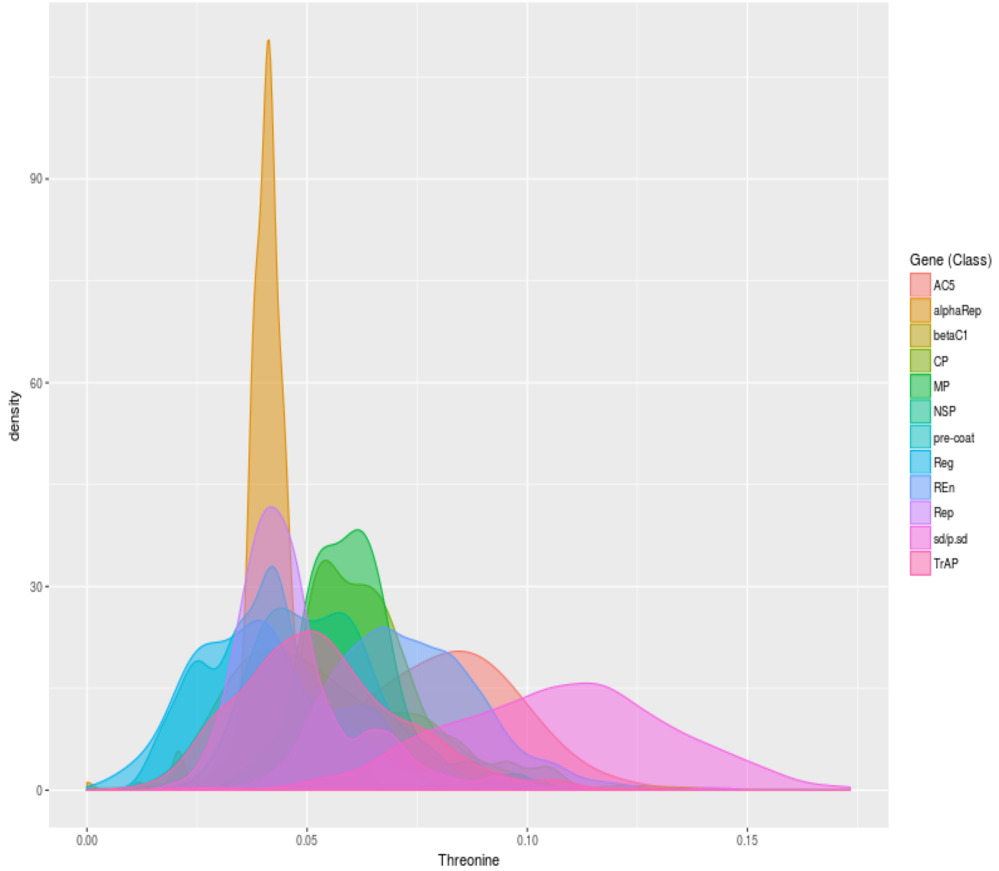

Boxplots

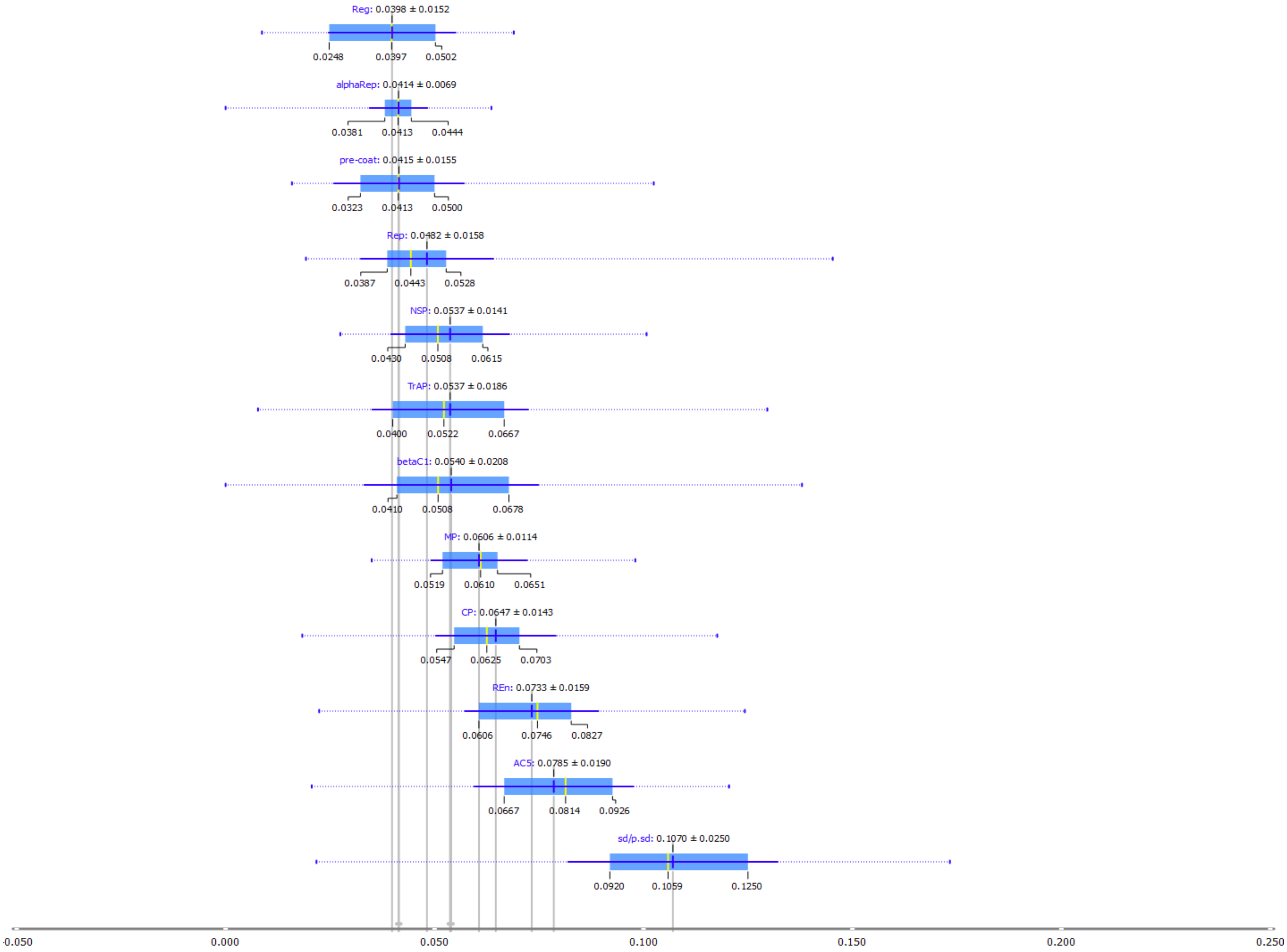

Reg: 0.0398 ± 0.0152
0.0248
0.0397
0.0502
alphaRep: 0.0414 ± 0.0069
0.0381
0.0413
0.0444
pre-coat: 0.0415 ± 0.0155
0.0323
0.0413
0.0500
Rep: 0.0482 ± 0.0158
0.0387
0.0443
0.0528
NSP: 0.0537 ± 0.0141
0.0430
0.0508
0.0615
TrAP: 0.0537 ± 0.0186
0.0400
0.0522
0.0667
betaC1: 0.0540 ± 0.0208
0.0410
0.0508
0.0678
MP: 0.0606 ± 0.0114
0.0519
0.0610
0.0651
CP: 0.0647 ± 0.0143
0.0547
0.0625
0.0703
REn: 0.0733 ± 0.0159
0.0606
0.0746
0.0827
AC5: 0.0785 ± 0.0190
0.0667
0.0814
0.0926
sd/p.sd: 0.1070 ± 0.0250
0.0920
0.1059
0.1250
-0.050
0.000
0.050
0.100
0.150
0.200
0.250

Proportion of Tryptophan

Histogram

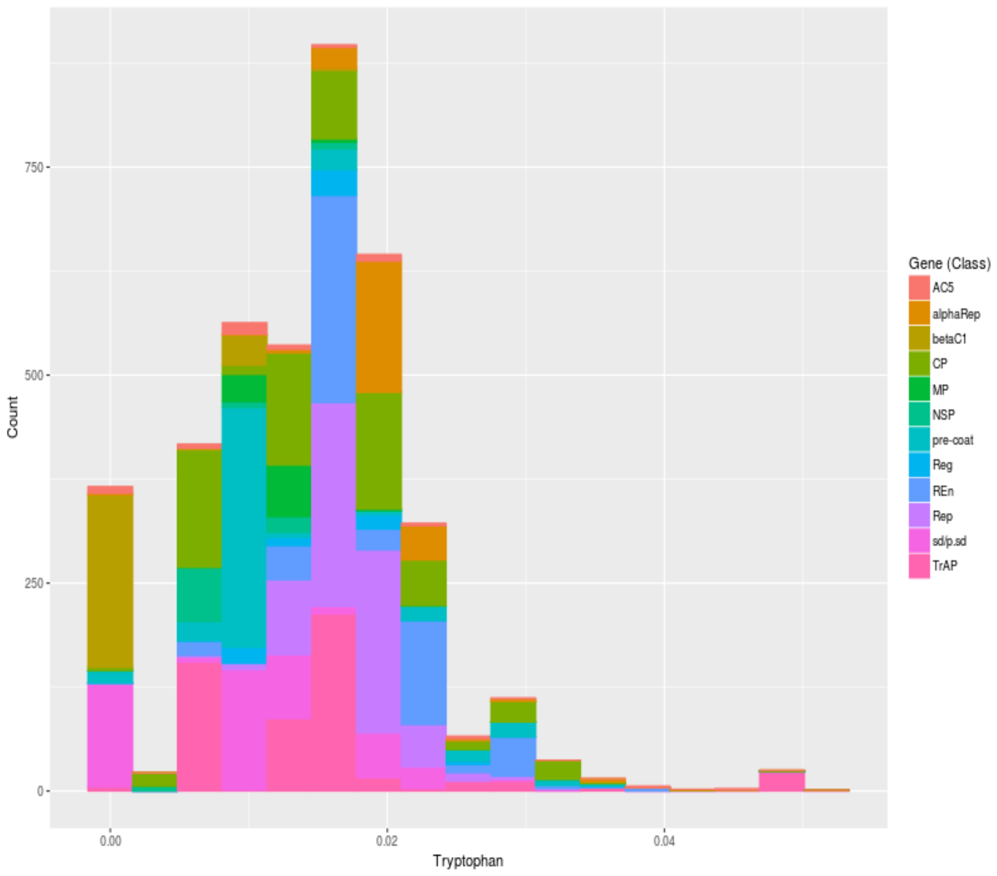

Density

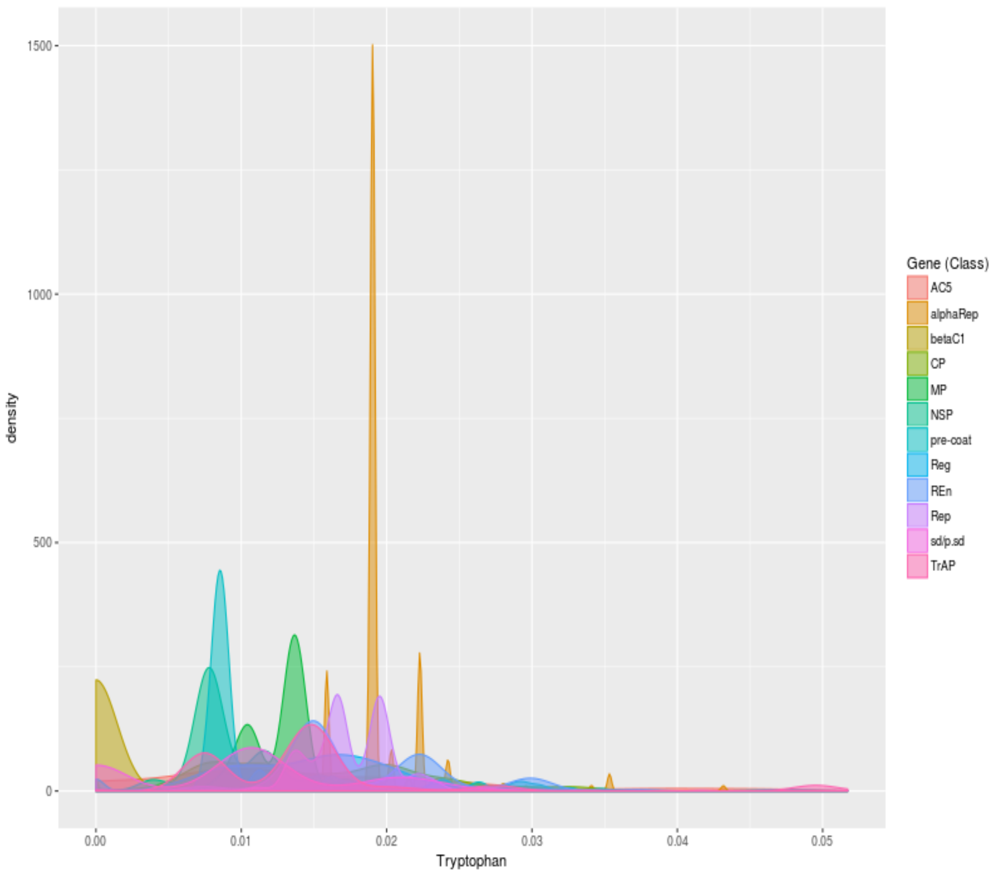

Boxplots

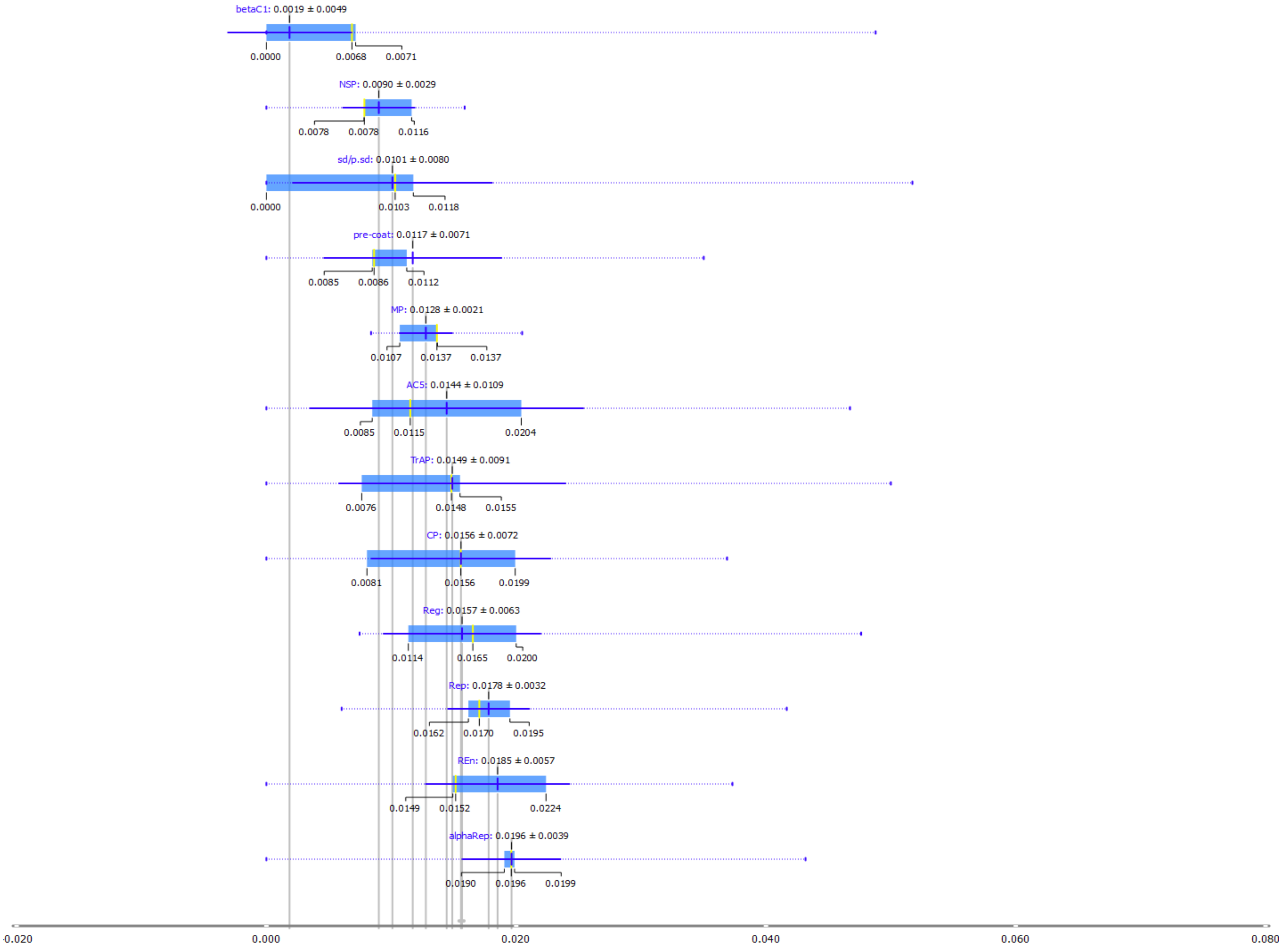

betaC1: 0.0019 ± 0.0049
0.0000
0.0068
0.0071
NSP: 0.0090 ± 0.0029
0.0078
0.0078
0.0116
sd/p.sd: 0.0101 ± 0.0080
0.0000
0.0103
0.0118
pre-coat: 0.0117 ± 0.0071
0.0085
0.0086
0.0112
MP: 0.0128 ± 0.0021
0.0107
0.0137
0.0137
AC5: 0.0144 ± 0.0109
0.0085
0.0115
0.0204
TrAP: 0.0149 ± 0.0091
0.0076
0.0148
0.0155
CP: 0.0156 ± 0.0072
0.0081
0.0156
0.0199
Reg: 0.0157 ± 0.0063
0.0114
0.0165
0.0200
Rep: 0.0178 ± 0.0032
0.0162
0.0170
0.0195
REn: 0.0185 ± 0.0057
0.0149
0.0152
0.0224
alphaRep: 0.0196 ± 0.0039
0.0190
0.0196
0.0199
-0.020
0.000
0.020
0.040
0.060
0.080

Proportion of Tyrosine

Histogram

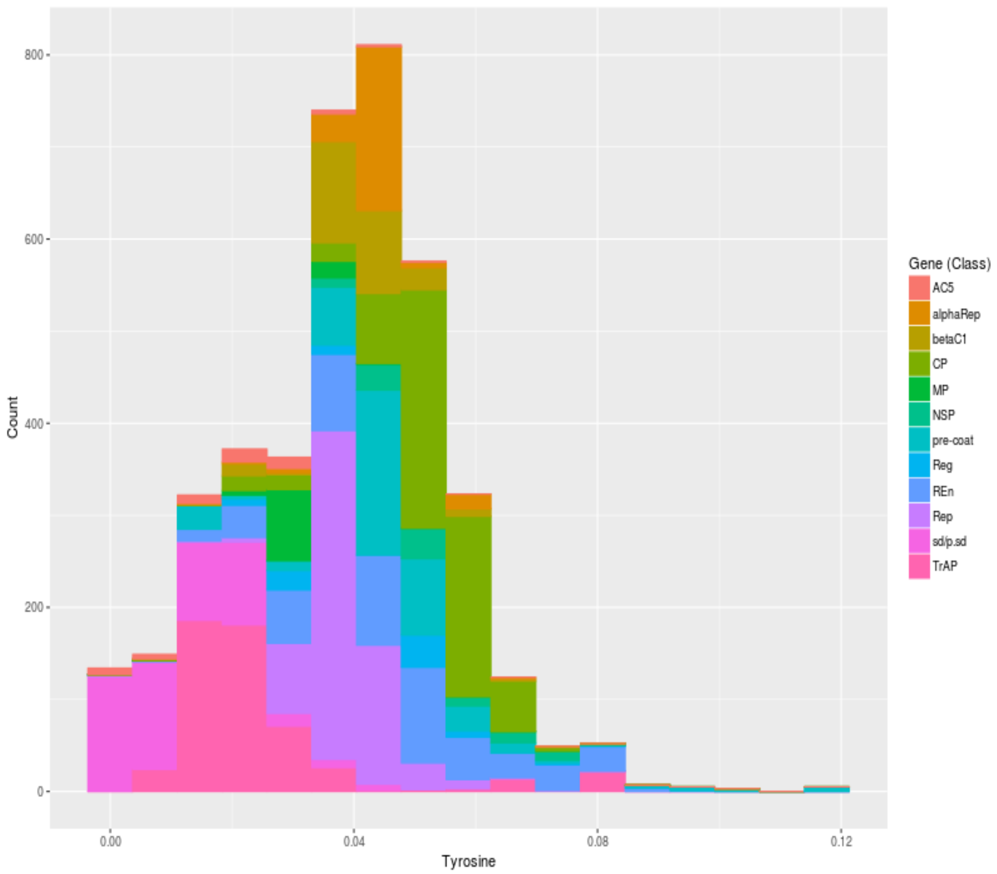

Density

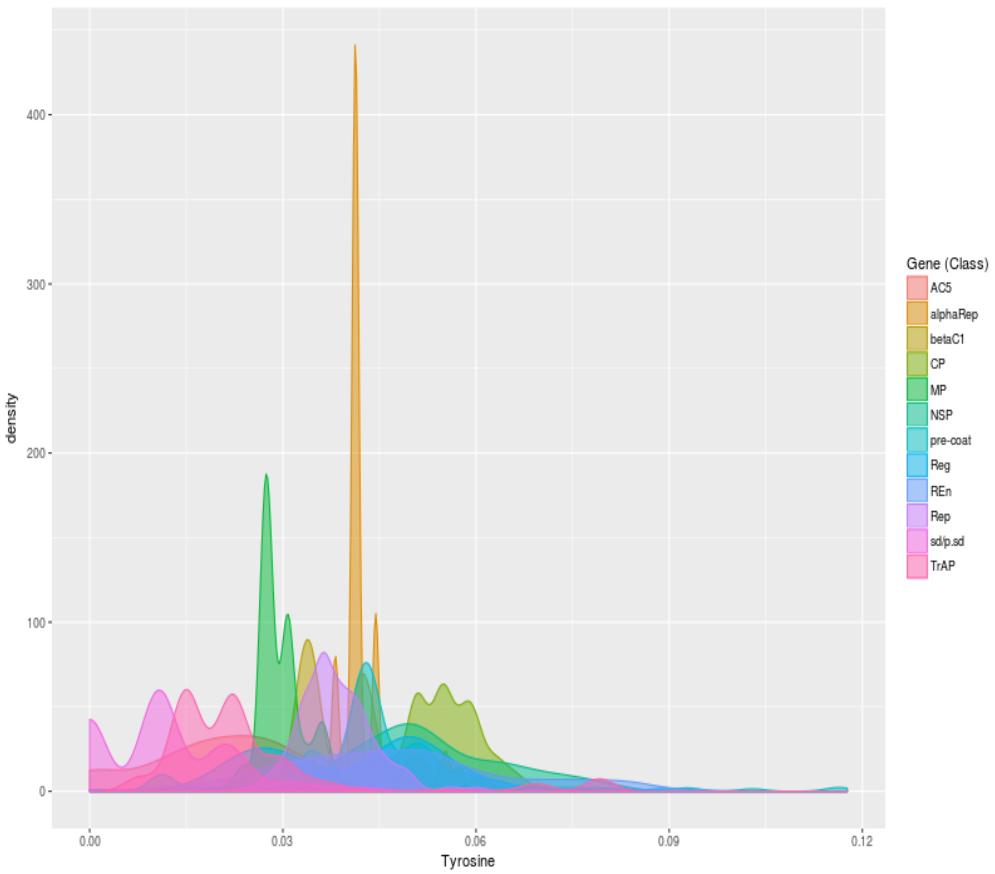

Boxplots

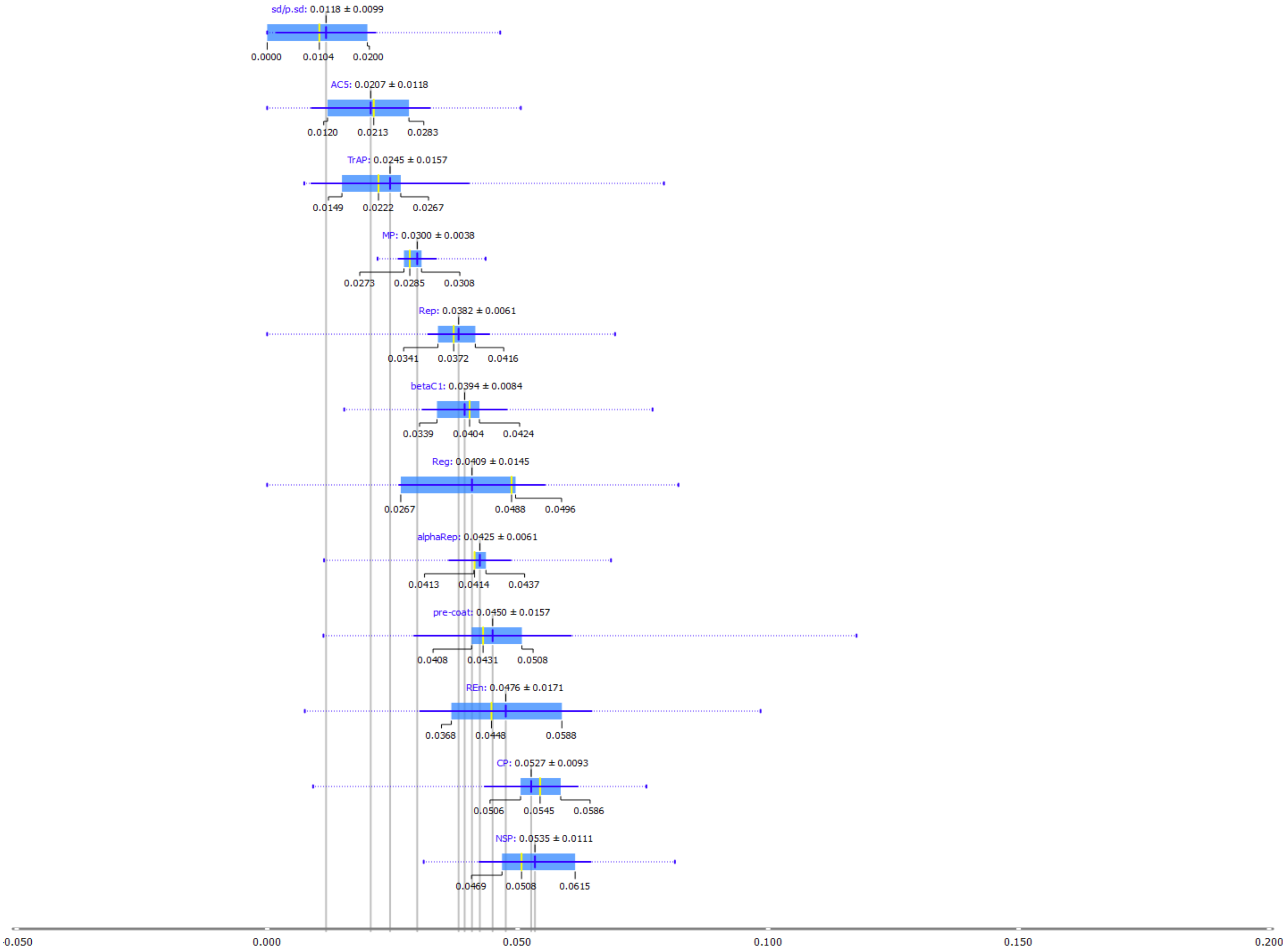
sd/p.sd: 0.0118 ± 0.0099
0.0000
0.0104
0.0200
AC5: 0.0207 ± 0.0118
0.0120
0.0213
0.0283
TrAP: 0.0245 ± 0.0157
0.0149
0.0222
0.0267
MP: 0.0300 ± 0.0038
0.0273
0.0285
0.0308
Rep: 0.0382 ± 0.0061
0.0341
0.0372
0.0416
betaC1: 0.0394 ± 0.0084
0.0339
0.0404
0.0424
Reg: 0.0409 ± 0.0145
0.0267
0.0488
0.0496
alphaRep: 0.0425 ± 0.0061
0.0413
0.0414
0.0437
pre-coat: 0.0450 ± 0.0157
0.0408
0.0431
0.0508
REn: 0.0476 ± 0.0171
0.0368
0.0448
0.0588
CP: 0.0527 ± 0.0093
0.0506
0.0545
0.0586
NSP: 0.0535 ± 0.0111
0.0469
0.0508
0.0615
-0.050
0.000
0.050
0.100
0.150
0.200

Proportion of Valine

Histogram

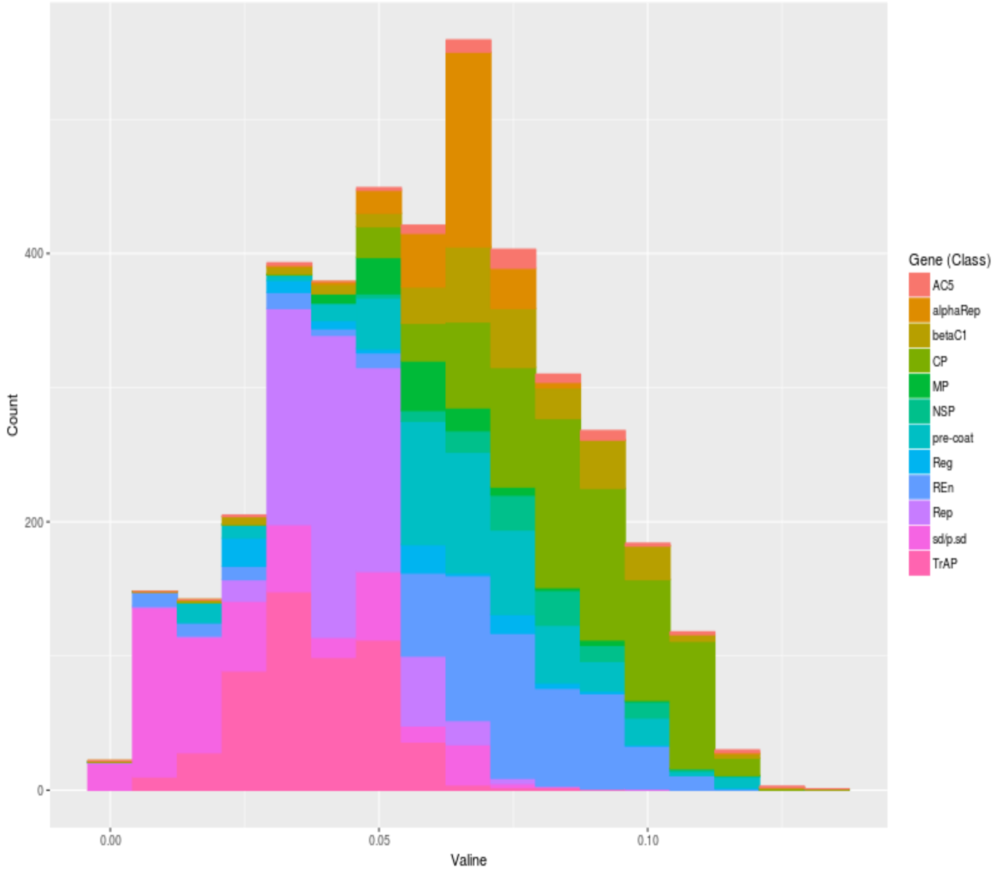

Density

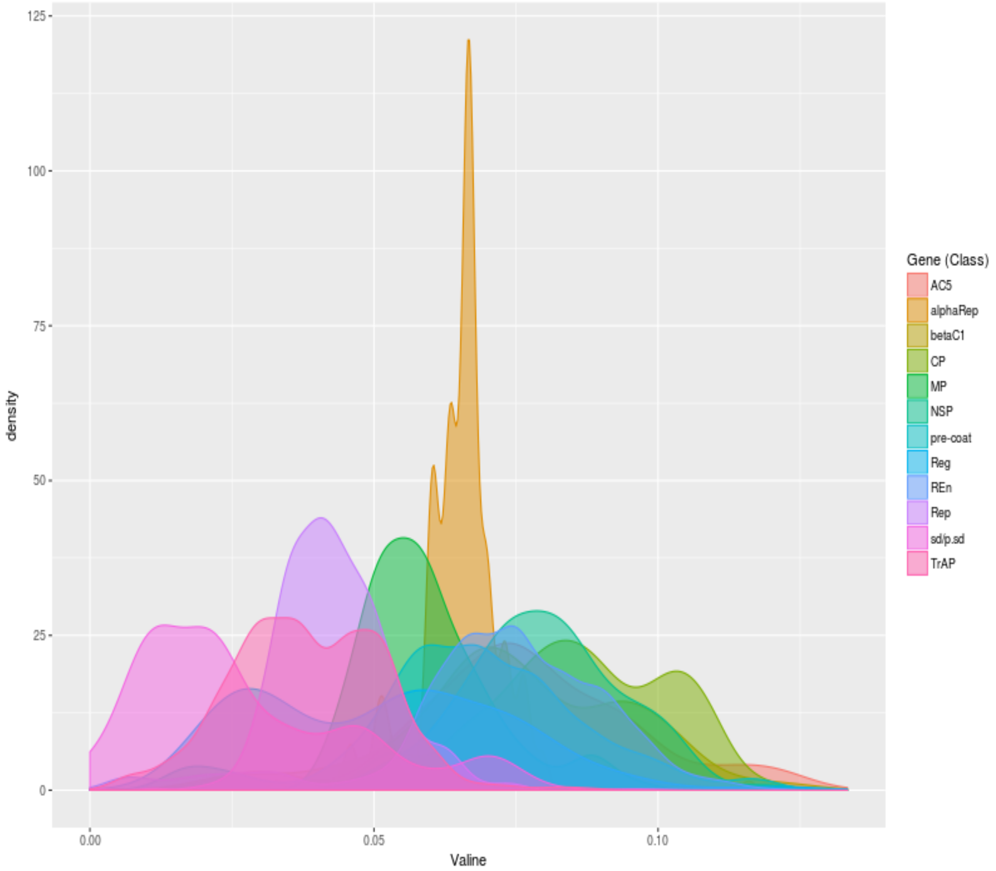

Boxplots

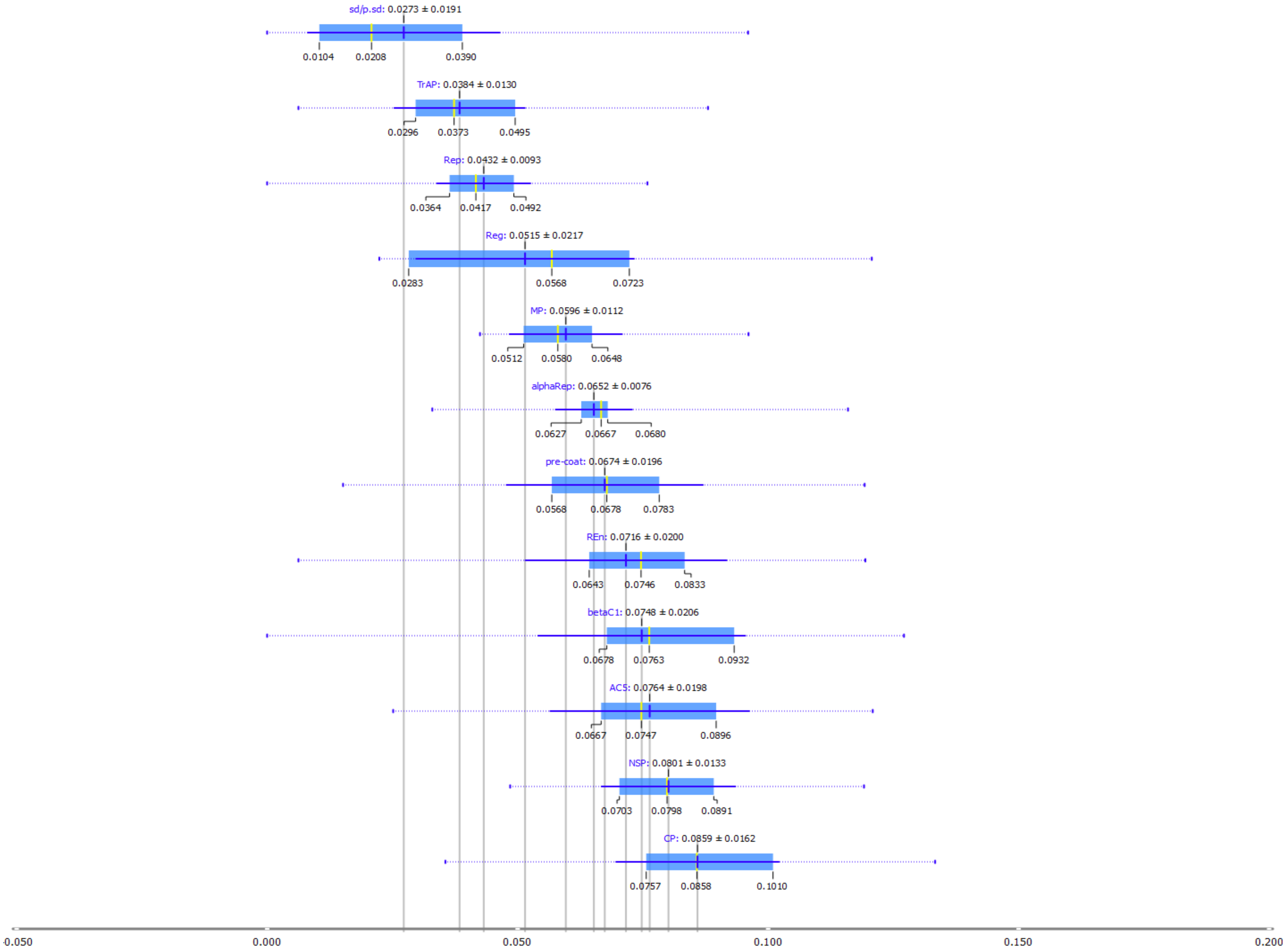

sd/p.sd: 0.0273 ± 0.0191
0.0104 0.0208 0.0390
TrAP: 0.0384 ± 0.0130
0.0296 0.0373 0.0495
Rep: 0.0432 ± 0.0093
0.0364 0.0417 0.0492
Reg: 0.0515 ± 0.0217
0.0283 0.0568 0.0723
MP: 0.0596 ± 0.0112
0.0512 0.0580 0.0648
alphaRep: 0.0652 ± 0.0076
0.0627 0.0667 0.0680
pre-coat: 0.0674 ± 0.0196
0.0568 0.0678 0.0783
REn: 0.0716 ± 0.0200
0.0643 0.0746 0.0833
betaC1: 0.0748 ± 0.0206
0.0678 0.0763 0.0932
AC5: 0.0764 ± 0.0198
0.0667 0.0747 0.0896
NSP: 0.0801 ± 0.0133
0.0703 0.0798 0.0891
CP: 0.0859 ± 0.0162
0.0757 0.0858 0.1010
-0.050 0.000 0.050 0.100 0.150 0.200
