# Supplementary material for: Fangorn Forest (F2): a machine learning approach to classify genes and genera in the family Geminiviridae
Source: BMC Bioinformatics. 2017 Sep 30;18:431. doi: 10.1186/s12859-017-1839-x (PMC5622471; doi:10.1186/s12859-017-1839-x)
Supplement: Supplementary file 7 — Equations S1. Model assessment measures. (DOCX 16 kb) [file 12859_2017_1839_MOESM7_ESM.docx]

# **Supplementary Equations S1. Model assessment measures.**

$$Accuracy= \frac{TP+TN}{\left( TP+FN \right)+\left( FP+TN \right)}$$

$$Precision=\frac{TP}{TP+FP}$$

$$Recall=\frac{TP}{TP+FN}$$

$$F measure=\frac{2TP}{2TP+FP+FN}$$

$$MCC=\frac{TP\times TN-FP\times FN}{\sqrt{(TP+FP)(TP+FN)(TN+FP)(TN+FN)}}$$

$TP :$ no. true positives

$TN :$ no. true negatives

$FP :$ no. false positives

$FN :$ no. false negatives
