# Supplementary material for: Fangorn Forest (F2): a machine learning approach to classify genes and genera in the family Geminiviridae
Source: BMC Bioinformatics. 2017 Sep 30;18:431. doi: 10.1186/s12859-017-1839-x (PMC5622471; doi:10.1186/s12859-017-1839-x)
Supplement: Supplementary file 8 — Performance results of the tests (performed with the Weka) for the models of family, genus and gene classification. (DOC 147 kb) [file 12859_2017_1839_MOESM8_ESM.doc]

**Supplementary Table S5. Performance results of the tests (performed with the Weka) for the models of classification of family, genus and gene.**

| **Type of evaluation** | **ML algorithm** | **Class** | **Detailed accuracy by class** |
| --- | --- | --- | --- |
| Using a test set | MLP | class 0 = non-geminivirus  class 1 = geminivirus | TP Rate FP Rate Precision Recall F-Measure MCC ROC Area PRC Area Class  0,910 0,022 0,976 0,910 0,942 0,891 0,969 0,978 0  0,978 0,090 0,917 0,978 0,947 0,891 0,969 0,939 1  Weighted Avg. 0,944 0,056 0,946 0,944 0,944 0,891 0,969 0,958  === Confusion Matrix ===  a b <-- classified as  8188 812 | a = 0  198 8990 | b = 1 |
| Using a test set | SMO | class 0 = non-geminivirus  class 1 = geminivirus | TP Rate FP Rate Precision Recall F-Measure MCC ROC Area PRC Area Class  0,750 0,130 0,850 0,750 0,797 0,625 0,810 0,761 0  0,870 0,250 0,780 0,870 0,823 0,625 0,810 0,745 1  Weighted Avg. 0,811 0,190 0,815 0,811 0,810 0,625 0,810 0,753  === Confusion Matrix ===  a b <-- classified as  6748 2252 | a = 0  1190 7998 | b = 1 |
| Using a test set | RF | class 0 = non-geminivirus  class 1 = geminivirus | TP Rate FP Rate Precision Recall F-Measure MCC ROC Area PRC Area Class  0,938 0,030 0,968 0,938 0,953 0,909 0,988 0,990 0  0,970 0,062 0,941 0,970 0,955 0,909 0,988 0,986 1  Weighted Avg. 0,954 0,046 0,955 0,954 0,954 0,909 0,988 0,988  === Confusion Matrix ===  a b <-- classified as  8442 558 | a = 0  275 8913 | b = 1 |
| 10-fold cross validation | MLP | class 0 = non-geminivirus  class 1 = geminivirus | TP Rate FP Rate Precision Recall F-Measure MCC ROC Area PRC Area Class  0,903 0,038 0,948 0,903 0,925 0,871 0,972 0,972 0  0,962 0,097 0,930 0,962 0,946 0,871 0,972 0,970 1  Weighted Avg. 0,937 0,072 0,937 0,937 0,937 0,871 0,972 0,971  === Confusion Matrix ===  a b <-- classified as  903 97 | a = 0  50 1283 | b = 1 |
| 10-fold cross validation | SMO | class 0 = non-geminivirus  class 1 = geminivirus | TP Rate FP Rate Precision Recall F-Measure MCC ROC Area PRC Area Class  0,754 0,066 0,895 0,754 0,819 0,709 0,844 0,781 0  0,934 0,246 0,835 0,934 0,882 0,709 0,844 0,818 1  Weighted Avg. 0,857 0,169 0,861 0,857 0,855 0,709 0,844 0,802  === Confusion Matrix ===  a b <-- classified as  754 246 | a = 0  88 1245 | b = 1 |
| 10-fold cross validation | RF | class 0 = non-geminivirus  class 1 = geminivirus | TP Rate FP Rate Precision Recall F-Measure MCC ROC Area PRC Area Class  0,937 0,023 0,969 0,937 0,953 0,919 0,992 0,991 0  0,977 0,063 0,954 0,977 0,966 0,919 0,992 0,993 1  Weighted Avg. 0,960 0,046 0,960 0,960 0,960 0,919 0,992 0,992  === Confusion Matrix ===  a b <-- classified as  937 63 | a = 0  30 1303 | b = 1 |
| Leave-one-out | MLP | class 0 = non-geminivirus  class 1 = geminivirus | TP Rate FP Rate Precision Recall F-Measure MCC ROC Area PRC Area Class  0,920 0,038 0,948 0,920 0,934 0,886 0,975 0,976 0  0,962 0,080 0,941 0,962 0,952 0,886 0,975 0,971 1  Weighted Avg. 0,944 0,062 0,944 0,944 0,944 0,886 0,975 0,973  === Confusion Matrix ===  a b <-- classified as  920 80 | a = 0  50 1283 | b = 1 |
| Leave-one-out | SMO | class 0 = non-geminivirus  class 1 = geminivirus | TP Rate FP Rate Precision Recall F-Measure MCC ROC Area PRC Area Class  0,754 0,068 0,893 0,754 0,818 0,707 0,843 0,779 0  0,932 0,246 0,835 0,932 0,881 0,707 0,843 0,817 1  Weighted Avg. 0,856 0,169 0,860 0,856 0,854 0,707 0,843 0,801  === Confusion Matrix ===  a b <-- classified as  754 246 | a = 0  90 1243 | b = 1 |
| Leave-one-out | RF | class 0 = non-geminivirus  class 1 = geminivirus | TP Rate FP Rate Precision Recall F-Measure MCC ROC Area PRC Area Class  0,937 0,019 0,974 0,937 0,955 0,923 0,992 0,991 0  0,981 0,063 0,954 0,981 0,967 0,923 0,992 0,992 1  Weighted Avg. 0,962 0,044 0,963 0,962 0,962 0,923 0,992 0,991  === Confusion Matrix ===  a b <-- classified as  937 63 | a = 0  25 1308 | b = 1 |
| Using a test set | MLP | class 0 = betasatellite  class 1 = alphasatellite  class 2 = Becurtovirus  class 3 = Curtovirus  class 4 = Eragrovirus  class 5 = Mastrevirus  class 7 = Turncurtovirus  class 8 = Begomovirus /  DNA-B  class 9 = Grablovirus  class 10 = Capulavirus | TP Rate FP Rate Precision Recall F-Measure MCC ROC Area PRC Area Class  0,930 0,009 0,929 0,930 0,930 0,921 0,988 0,966 0  0,980 0,024 0,583 0,980 0,732 0,747 0,988 0,942 1  1,000 0,006 0,161 1,000 0,278 0,400 0,999 0,804 2  0,989 0,004 0,712 0,989 0,828 0,838 1,000 0,986 3  0,000 0,000 0,000 0,000 0,000 0,000 0,990 0,016 4  0,944 0,004 0,977 0,944 0,960 0,952 0,991 0,983 5  0,741 0,002 0,526 0,741 0,615 0,623 0,998 0,457 7  0,941 0,036 0,982 0,941 0,961 0,886 0,958 0,984 8  1,000 0,000 1,000 1,000 1,000 1,000 1,000 1,000 9  0,938 0,001 0,682 0,938 0,789 0,799 0,970 0,636 10  Weighted Avg. 0,941 0,027 0,957 0,941 0,946 0,894 0,968 0,978  === Confusion Matrix ===  a b c d e f g h i j <-- classified as  917 14 5 0 0 0 14 31 0 5 | a = 0  3 297 1 0 0 0 0 2 0 0 | b = 1  0 0 10 0 0 0 0 0 0 0 | c = 2  0 0 0 94 0 0 0 1 0 0 | d = 3  0 0 0 0 0 2 0 0 0 0 | e = 4  0 14 4 0 0 1438 0 67 0 1 | f = 5  0 0 0 0 0 0 20 7 0 0 | g = 7  67 184 42 38 0 32 3 5849 0 1 | h = 8  0 0 0 0 0 0 0 0 9 0 | i = 9  0 0 0 0 0 0 1 0 0 15 | j = 10 |
| Using a test set | SMO | class 0 = betasatellite  class 1 = alphasatellite  class 2 = Becurtovirus  class 3 = Curtovirus  class 4 = Eragrovirus  class 5 = Mastrevirus  class 7 = Turncurtovirus  class 8 = Begomovirus /  DNA-B  class 9 = Grablovirus  class 10 = Capulavirus | TP Rate FP Rate Precision Recall F-Measure MCC ROC Area PRC Area Class  0,919 0,002 0,983 0,919 0,950 0,945 0,978 0,941 0  0,921 0,003 0,924 0,921 0,922 0,920 0,976 0,865 1  0,900 0,007 0,120 0,900 0,212 0,327 0,984 0,095 2  0,937 0,003 0,767 0,937 0,844 0,846 0,998 0,741 3  0,000 0,000 0,000 0,000 0,000 0,000 0,915 0,001 4  0,159 0,000 0,992 0,159 0,274 0,367 0,862 0,483 5  0,926 0,003 0,500 0,926 0,649 0,679 0,998 0,491 7  0,984 0,455 0,819 0,984 0,894 0,634 0,766 0,818 8  1,000 0,000 0,900 1,000 0,947 0,949 1,000 0,900 9  0,000 0,000 0,000 0,000 0,000 -0,001 0,829 0,006 10  Weighted Avg. 0,835 0,308 0,865 0,835 0,794 0,634 0,815 0,773  === Confusion Matrix ===  a b c d e f g h i j <-- classified as  906 23 0 9 0 1 16 29 0 2 | a = 0  6 279 1 1 0 1 0 15 0 0 | b = 1  0 0 9 0 0 0 0 1 0 0 | c = 2  0 0 0 89 0 0 0 6 0 0 | d = 3  0 0 0 0 0 0 0 2 0 0 | e = 4  0 0 0 0 0 242 0 1282 0 0 | f = 5  0 0 0 0 0 0 25 2 0 0 | g = 7  10 0 64 17 0 0 9 6115 1 0 | h = 8  0 0 0 0 0 0 0 0 9 0 | i = 9  0 0 1 0 0 0 0 15 0 0 | j = 10 |
| Using a test set | RF | class 0 = betasatellite  class 1 = alphasatellite  class 2 = Becurtovirus  class 3 = Curtovirus  class 4 = Eragrovirus  class 5 = Mastrevirus  class 7 = Turncurtovirus  class 8 = Begomovirus /  DNA-B  class 9 = Grablovirus  class 10 = Capulavirus | TP Rate FP Rate Precision Recall F-Measure MCC ROC Area PRC Area Class  0,925 0,005 0,961 0,925 0,943 0,936 0,994 0,973 0  0,970 0,009 0,788 0,970 0,870 0,870 0,993 0,976 1  0,800 0,005 0,138 0,800 0,235 0,331 0,995 0,808 2  0,989 0,003 0,764 0,989 0,862 0,868 1,000 0,977 3  1,000 0,000 0,667 1,000 0,800 0,816 1,000 1,000 4  0,834 0,010 0,942 0,834 0,885 0,866 0,995 0,977 5  0,926 0,002 0,610 0,926 0,735 0,751 1,000 0,974 7  0,961 0,095 0,955 0,961 0,958 0,870 0,988 0,994 8  1,000 0,000 1,000 1,000 1,000 1,000 1,000 1,000 9  0,938 0,001 0,625 0,938 0,750 0,765 1,000 0,963 10  Weighted Avg. 0,937 0,067 0,943 0,937 0,939 0,875 0,990 0,988  === Confusion Matrix ===  a b c d e f g h i j <-- classified as  912 28 1 3 0 1 14 24 0 3 | a = 0  4 294 1 0 0 1 0 3 0 0 | b = 1  0 0 8 0 0 0 0 1 0 1 | c = 2  0 0 1 94 0 0 0 0 0 0 | d = 3  0 0 0 0 2 0 0 0 0 0 | e = 4  0 0 0 0 0 1271 0 253 0 0 | f = 5  0 0 0 0 0 0 25 2 0 0 | g = 7  33 51 46 26 1 76 2 5976 0 5 | h = 8  0 0 0 0 0 0 0 0 9 0 | i = 9  0 0 1 0 0 0 0 0 0 15 | j = 10 |
| 10-fold cross validation | MLP | class 0 = betasatellite  class 1 = alphasatellite  class 2 = Becurtovirus  class 3 = Curtovirus  class 4 = Eragrovirus  class 5 = Mastrevirus  class 7 = Turncurtovirus  class 8 = Begomovirus /  DNA-B  class 9 = Grablovirus  class 10 = Capulavirus | TP Rate FP Rate Precision Recall F-Measure MCC ROC Area PRC Area Class  0,981 0,005 0,981 0,981 0,981 0,976 1,000 0,999 0  0,983 0,009 0,971 0,983 0,977 0,970 0,999 0,997 1  0,913 0,002 0,875 0,913 0,894 0,892 0,999 0,860 2  1,000 0,001 0,947 1,000 0,973 0,973 1,000 0,991 3  1,000 0,000 1,000 1,000 1,000 1,000 1,000 1,000 4  0,877 0,005 0,893 0,877 0,885 0,880 0,969 0,868 5  1,000 0,001 0,952 1,000 0,976 0,976 1,000 1,000 7  0,973 0,014 0,983 0,973 0,978 0,961 0,992 0,993 8  1,000 0,002 0,895 1,000 0,944 0,945 1,000 0,993 9  0,903 0,001 0,966 0,903 0,933 0,932 0,989 0,963 10  Weighted Avg. 0,971 0,009 0,972 0,971 0,971 0,961 0,994 0,987  === Confusion Matrix ===  a b c d e f g h i j <-- classified as  253 4 0 0 0 0 0 1 0 0 | a = 0  2 297 0 0 0 1 0 2 0 0 | b = 1  0 0 21 1 0 0 1 0 0 0 | c = 2  0 0 0 18 0 0 0 0 0 0 | d = 3  0 0 0 0 4 0 0 0 0 0 | e = 4  0 0 0 0 0 50 0 6 0 1 | f = 5  0 0 0 0 0 0 20 0 0 0 | g = 7  3 5 2 0 0 5 0 586 1 0 | h = 8  0 0 0 0 0 0 0 0 17 0 | i = 9  0 0 1 0 0 0 0 1 1 28 | j = 10 |
| 10-fold cross validation | SMO | class 0 = betasatellite  class 1 = alphasatellite  class 2 = Becurtovirus  class 3 = Curtovirus  class 4 = Eragrovirus  class 5 = Mastrevirus  class 7 = Turncurtovirus  class 8 = Begomovirus /  DNA-B  class 9 = Grablovirus  class 10 = Capulavirus | TP Rate FP Rate Precision Recall F-Measure MCC ROC Area PRC Area Class  0,981 0,010 0,958 0,981 0,969 0,962 0,995 0,962 0  0,921 0,001 0,996 0,921 0,957 0,946 0,991 0,967 1  0,783 0,004 0,783 0,783 0,783 0,779 0,989 0,687 2  1,000 0,004 0,783 1,000 0,878 0,883 0,998 0,783 3  0,000 0,000 0,000 0,000 0,000 0,000 0,876 0,012 4  0,579 0,002 0,943 0,579 0,717 0,731 0,892 0,589 5  1,000 0,002 0,909 1,000 0,952 0,953 0,999 0,909 7  0,988 0,099 0,892 0,988 0,938 0,886 0,946 0,889 8  1,000 0,000 1,000 1,000 1,000 1,000 1,000 1,000 9  0,000 0,002 0,000 0,000 0,000 -0,006 0,895 0,116 10  Weighted Avg. 0,925 0,047 0,906 0,925 0,912 0,885 0,965 0,884  === Confusion Matrix ===  a b c d e f g h i j <-- classified as  253 1 0 3 0 0 0 1 0 0 | a = 0  10 278 0 0 0 2 0 10 0 2 | b = 1  0 0 18 0 0 0 1 4 0 0 | c = 2  0 0 0 18 0 0 0 0 0 0 | d = 3  0 0 0 0 0 0 0 4 0 0 | e = 4  0 0 0 0 0 33 0 24 0 0 | f = 5  0 0 0 0 0 0 20 0 0 0 | g = 7  1 0 3 2 0 0 1 595 0 0 | h = 8  0 0 0 0 0 0 0 0 17 0 | i = 9  0 0 2 0 0 0 0 29 0 0 | j = 10 |
| 10-fold cross validation | RF | class 0 = betasatellite  class 1 = alphasatellite  class 2 = Becurtovirus  class 3 = Curtovirus  class 4 = Eragrovirus  class 5 = Mastrevirus  class 7 = Turncurtovirus  class 8 = Begomovirus /  DNA-B  class 9 = Grablovirus  class 10 = Capulavirus | TP Rate FP Rate Precision Recall F-Measure MCC ROC Area PRC Area Class  0,981 0,008 0,966 0,981 0,973 0,967 1,000 0,998 0  0,974 0,005 0,983 0,974 0,978 0,972 0,999 0,997 1  0,957 0,002 0,880 0,957 0,917 0,916 0,999 0,973 2  0,944 0,001 0,944 0,944 0,944 0,944 1,000 0,994 3  1,000 0,002 0,667 1,000 0,800 0,816 1,000 0,950 4  0,719 0,002 0,953 0,719 0,820 0,822 0,992 0,902 5  1,000 0,001 0,952 1,000 0,976 0,976 1,000 1,000 7  0,980 0,032 0,962 0,980 0,971 0,947 0,998 0,997 8  1,000 0,000 1,000 1,000 1,000 1,000 1,000 1,000 9  0,903 0,000 1,000 0,903 0,949 0,949 0,982 0,967 10  Weighted Avg. 0,965 0,017 0,966 0,965 0,965 0,951 0,998 0,992  === Confusion Matrix ===  a b c d e f g h i j <-- classified as  253 3 0 0 0 0 0 2 0 0 | a = 0  3 294 0 0 1 1 0 3 0 0 | b = 1  0 0 22 0 0 0 0 1 0 0 | c = 2  0 0 0 17 0 0 0 1 0 0 | d = 3  0 0 0 0 4 0 0 0 0 0 | e = 4  0 0 0 0 1 41 0 15 0 0 | f = 5  0 0 0 0 0 0 20 0 0 0 | g = 7  6 1 2 1 0 1 1 590 0 0 | h = 8  0 0 0 0 0 0 0 0 17 0 | i = 9  0 1 1 0 0 0 0 1 0 28 | j = 10 |
| Leave-one-out | MLP | class 0 = betasatellite  class 1 = alphasatellite  class 2 = Becurtovirus  class 3 = Curtovirus  class 4 = Eragrovirus  class 5 = Mastrevirus  class 7 = Turncurtovirus  class 8 = Begomovirus /  DNA-B  class 9 = Grablovirus  class 10 = Capulavirus | TP Rate FP Rate Precision Recall F-Measure MCC ROC Area PRC Area Class  0,984 0,004 0,984 0,984 0,984 0,981 1,000 0,999 0  0,980 0,005 0,983 0,980 0,982 0,976 0,996 0,993 1  0,957 0,002 0,917 0,957 0,936 0,935 0,999 0,896 2  1,000 0,000 1,000 1,000 1,000 1,000 1,000 1,000 3  1,000 0,001 0,800 1,000 0,889 0,894 1,000 0,950 4  0,842 0,008 0,828 0,842 0,835 0,827 0,945 0,848 5  1,000 0,001 0,952 1,000 0,976 0,976 1,000 1,000 7  0,973 0,018 0,978 0,973 0,976 0,956 0,989 0,989 8  1,000 0,000 1,000 1,000 1,000 1,000 1,000 1,000 9  0,935 0,002 0,935 0,935 0,935 0,934 0,983 0,955 10  Weighted Avg. 0,971 0,010 0,972 0,971 0,972 0,960 0,992 0,984  === Confusion Matrix ===  a b c d e f g h i j <-- classified as  254 1 0 0 0 0 0 2 0 1 | a = 0  1 296 0 0 0 2 0 3 0 0 | b = 1  0 0 22 0 0 0 1 0 0 0 | c = 2  0 0 0 18 0 0 0 0 0 0 | d = 3  0 0 0 0 4 0 0 0 0 0 | e = 4  0 1 0 0 0 48 0 7 0 1 | f = 5  0 0 0 0 0 0 20 0 0 0 | g = 7  3 3 1 0 1 8 0 586 0 0 | h = 8  0 0 0 0 0 0 0 0 17 0 | i = 9  0 0 1 0 0 0 0 1 0 29 | j = 10 |
| Leave-one-out | SMO | class 0 = betasatellite  class 1 = alphasatellite  class 2 = Becurtovirus  class 3 = Curtovirus  class 4 = Eragrovirus  class 5 = Mastrevirus  class 7 = Turncurtovirus  class 8 = Begomovirus /  DNA-B  class 9 = Grablovirus  class 10 = Capulavirus | TP Rate FP Rate Precision Recall F-Measure MCC ROC Area PRC Area Class  0,981 0,011 0,955 0,981 0,967 0,960 0,995 0,959 0  0,921 0,001 0,996 0,921 0,957 0,946 0,991 0,967 1  0,739 0,004 0,773 0,739 0,756 0,752 0,988 0,655 2  1,000 0,004 0,783 1,000 0,878 0,883 0,998 0,783 3  0,000 0,000 0,000 0,000 0,000 0,000 0,875 0,012 4  0,561 0,002 0,941 0,561 0,703 0,718 0,884 0,573 5  1,000 0,002 0,909 1,000 0,952 0,953 0,999 0,909 7  0,987 0,101 0,889 0,987 0,935 0,881 0,944 0,886 8  1,000 0,000 1,000 1,000 1,000 1,000 1,000 1,000 9  0,000 0,002 0,000 0,000 0,000 -0,006 0,896 0,117 10  Weighted Avg. 0,923 0,048 0,903 0,923 0,909 0,881 0,964 0,881  === Confusion Matrix ===  a b c d e f g h i j <-- classified as  253 1 0 3 0 0 0 1 0 0 | a = 0  10 278 0 0 0 2 0 10 0 2 | b = 1  0 0 17 0 0 0 1 5 0 0 | c = 2  0 0 0 18 0 0 0 0 0 0 | d = 3  0 0 0 0 0 0 0 4 0 0 | e = 4  0 0 0 0 0 32 0 25 0 0 | f = 5  0 0 0 0 0 0 20 0 0 0 | g = 7  2 0 3 2 0 0 1 594 0 0 | h = 8  0 0 0 0 0 0 0 0 17 0 | i = 9  0 0 2 0 0 0 0 29 0 0 | j = 10 |
| Leave-one-out | RF | class 0 = betasatellite  class 1 = alphasatellite  class 2 = Becurtovirus  class 3 = Curtovirus  class 4 = Eragrovirus  class 5 = Mastrevirus  class 7 = Turncurtovirus  class 8 = Begomovirus /  DNA-B  class 9 = Grablovirus  class 10 = Capulavirus | TP Rate FP Rate Precision Recall F-Measure MCC ROC Area PRC Area Class  0,981 0,006 0,977 0,981 0,979 0,974 1,000 0,998 0  0,983 0,005 0,983 0,983 0,983 0,979 0,999 0,998 1  0,957 0,002 0,917 0,957 0,936 0,935 0,998 0,962 2  1,000 0,002 0,900 1,000 0,947 0,948 1,000 0,989 3  1,000 0,001 0,800 1,000 0,889 0,894 1,000 1,000 4  0,719 0,003 0,911 0,719 0,804 0,802 0,993 0,907 5  1,000 0,001 0,952 1,000 0,976 0,976 1,000 1,000 7  0,980 0,029 0,966 0,980 0,973 0,950 0,998 0,997 8  1,000 0,000 1,000 1,000 1,000 1,000 1,000 1,000 9  0,903 0,000 1,000 0,903 0,949 0,949 0,982 0,964 10  Weighted Avg. 0,968 0,015 0,968 0,968 0,968 0,955 0,998 0,992  === Confusion Matrix ===  a b c d e f g h i j <-- classified as  253 3 0 0 0 0 0 2 0 0 | a = 0  1 297 0 0 0 2 0 2 0 0 | b = 1  0 0 22 0 0 0 0 1 0 0 | c = 2  0 0 0 18 0 0 0 0 0 0 | d = 3  0 0 0 0 4 0 0 0 0 0 | e = 4  0 0 0 0 1 41 0 15 0 0 | f = 5  0 0 0 0 0 0 20 0 0 0 | g = 7  5 1 1 2 0 2 1 590 0 0 | h = 8  0 0 0 0 0 0 0 0 17 0 | i = 9  0 1 1 0 0 0 0 1 0 28 | j = 10 |
| Using a test set | MLP | class 0 = betaC1  class 1 = alphaRep  class 2 = Rep  class 3 = TrAP  class 4 = REn  class 5 = sd/p.sd  class 6 = AC5  class 7 = CP  class 8 = pre-coat  class 9 = Reg  class 10 = MP  class 11 = NSP | TP Rate FP Rate Precision Recall F-Measure MCC ROC Area PRC Area Class  0,994 0,000 0,990 0,994 0,992 0,992 0,997 0,995 0  0,948 0,001 0,929 0,948 0,938 0,938 0,975 0,948 1  0,977 0,005 0,978 0,977 0,978 0,973 0,998 0,995 2  0,987 0,005 0,968 0,987 0,977 0,974 0,998 0,994 3  0,995 0,001 0,992 0,995 0,994 0,993 0,998 0,989 4  0,993 0,002 0,988 0,993 0,991 0,989 0,999 0,998 5  0,975 0,001 0,970 0,975 0,972 0,972 0,999 0,983 6  0,985 0,012 0,950 0,985 0,967 0,960 0,996 0,986 7  0,913 0,004 0,965 0,913 0,938 0,931 0,990 0,946 8  0,737 0,001 0,846 0,737 0,788 0,788 0,876 0,692 9  0,868 0,000 0,988 0,868 0,924 0,924 0,993 0,984 10  0,973 0,001 0,981 0,973 0,977 0,976 0,999 0,994 11  Weighted Avg. 0,973 0,005 0,973 0,973 0,972 0,968 0,996 0,985  === Confusion Matrix ===  a b c d e f g h i j k l <-- classified as  867 0 0 0 1 0 1 1 0 0 0 2 | a = 0  1 236 0 0 1 0 0 4 5 2 0 0 | b = 1  3 2 6289 112 6 10 2 4 0 0 7 0 | c = 2  1 0 5 4369 17 26 6 0 2 0 2 0 | d = 3  2 0 2 16 4468 0 2 1 0 0 0 0 | e = 4  1 0 7 10 1 4348 10 0 0 0 0 0 | f = 5  0 0 0 2 2 13 706 1 0 0 0 0 | g = 6  0 9 5 0 1 0 0 6233 72 1 0 9 | h = 7  0 5 0 5 0 0 0 297 3507 20 0 6 | i = 8  0 0 0 0 5 0 0 0 40 126 0 0 | j = 9  1 0 120 0 0 1 1 1 6 0 870 2 | k = 10  0 2 0 0 0 2 0 19 2 0 2 972 | l = 11 |
| Using a test set | SMO | class 0 = betaC1  class 1 = alphaRep  class 2 = Rep  class 3 = TrAP  class 4 = REn  class 5 = sd/p.sd  class 6 = AC5  class 7 = CP  class 8 = pre-coat  class 9 = Reg  class 10 = MP  class 11 = NSP | TP Rate FP Rate Precision Recall F-Measure MCC ROC Area PRC Area Class  0,994 0,000 0,993 0,994 0,994 0,994 0,999 0,989 0  0,944 0,000 0,983 0,944 0,963 0,963 0,988 0,935 1  0,977 0,001 0,996 0,977 0,986 0,983 0,998 0,991 2  0,988 0,006 0,960 0,988 0,974 0,970 0,994 0,955 3  0,996 0,002 0,990 0,996 0,993 0,992 0,998 0,987 4  0,991 0,001 0,992 0,991 0,992 0,991 0,999 0,989 5  0,981 0,000 0,982 0,981 0,981 0,981 0,997 0,968 6  0,987 0,013 0,947 0,987 0,967 0,959 0,992 0,942 7  0,922 0,004 0,965 0,922 0,943 0,936 0,992 0,932 8  0,731 0,000 0,933 0,731 0,820 0,825 0,965 0,695 9  0,976 0,000 0,994 0,976 0,985 0,984 0,990 0,972 10  0,939 0,000 0,996 0,939 0,967 0,966 0,995 0,947 11  Weighted Avg. 0,976 0,004 0,977 0,976 0,976 0,973 0,995 0,966  === Confusion Matrix ===  a b c d e f g h i j k l <-- classified as  867 0 0 0 2 0 0 0 3 0 0 0 | a = 0  1 235 0 4 1 0 0 0 6 2 0 0 | b = 1  0 1 6285 130 4 6 1 4 0 0 3 1 | c = 2  0 0 3 4376 26 20 1 0 2 0 0 0 | d = 3  0 0 2 16 4471 0 1 1 0 0 0 0 | e = 4  1 0 1 23 3 4339 9 0 0 1 0 0 | f = 5  0 0 0 2 4 7 710 0 1 0 0 0 | g = 6  1 1 5 0 0 0 0 6249 71 1 1 1 | h = 7  0 0 0 5 0 0 0 289 3541 5 0 0 | i = 8  2 0 0 0 5 0 0 0 39 125 0 0 | j = 9  1 0 12 0 0 1 1 1 6 0 978 2 | k = 10  0 2 0 0 0 0 0 57 0 0 2 938 | l = 11 |
| Using a test set | RF | class 0 = betaC1  class 1 = alphaRep  class 2 = Rep  class 3 = TrAP  class 4 = REn  class 5 = sd/p.sd  class 6 = AC5  class 7 = CP  class 8 = pre-coat  class 9 = Reg  class 10 = MP  class 11 = NSP | TP Rate FP Rate Precision Recall F-Measure MCC ROC Area PRC Area Class  0,993 0,000 0,998 0,993 0,995 0,995 1,000 0,998 0  0,992 0,000 0,988 0,992 0,990 0,990 1,000 0,990 1  0,977 0,002 0,993 0,977 0,985 0,982 0,998 0,995 2  0,984 0,005 0,965 0,984 0,974 0,971 0,998 0,992 3  0,995 0,001 0,995 0,995 0,995 0,994 0,998 0,993 4  0,997 0,003 0,980 0,997 0,989 0,987 1,000 0,998 5  0,970 0,000 0,985 0,970 0,977 0,977 0,999 0,989 6  0,971 0,003 0,987 0,971 0,979 0,974 0,998 0,990 7  0,980 0,007 0,949 0,980 0,964 0,960 0,996 0,984 8  0,725 0,000 0,992 0,725 0,838 0,848 0,986 0,848 9  0,977 0,000 0,997 0,977 0,987 0,987 0,994 0,986 10  0,990 0,000 0,994 0,990 0,992 0,992 0,999 0,990 11  Weighted Avg. 0,982 0,003 0,982 0,982 0,982 0,979 0,998 0,991  === Confusion Matrix ===  a b c d e f g h i j k l <-- classified as  866 0 0 0 0 3 0 0 2 0 0 1 | a = 0  1 247 0 1 0 0 0 0 0 0 0 0 | b = 1  0 0 6290 109 4 25 1 4 1 0 1 0 | c = 2  0 0 20 4356 14 36 1 0 1 0 0 0 | d = 3  0 0 2 11 4467 9 2 0 0 0 0 0 | e = 4  1 0 2 3 0 4366 5 0 0 0 0 0 | f = 5  0 0 4 2 0 16 702 0 0 0 0 0 | g = 6  0 1 5 0 0 0 0 6147 175 1 0 1 | h = 7  0 0 0 4 0 0 0 72 3764 0 0 0 | i = 8  0 0 0 25 4 0 1 0 17 124 0 0 | j = 9  0 0 10 1 0 1 1 0 6 0 979 4 | k = 10  0 2 0 0 0 0 0 4 2 0 2 989 | l = 11 |
| 10-fold cross validation | MLP | class 0 = betaC1  class 1 = alphaRep  class 2 = Rep  class 3 = TrAP  class 4 = REn  class 5 = sd/p.sd  class 6 = AC5  class 7 = CP  class 8 = pre-coat  class 9 = Reg  class 10 = MP  class 11 = NSP | TP Rate FP Rate Precision Recall F-Measure MCC ROC Area PRC Area Class  0,968 0,001 0,992 0,968 0,980 0,979 0,991 0,969 0  0,971 0,002 0,975 0,971 0,973 0,971 0,988 0,961 1  0,983 0,002 0,989 0,983 0,986 0,983 0,996 0,993 2  0,981 0,003 0,977 0,981 0,979 0,976 0,996 0,990 3  0,987 0,003 0,981 0,987 0,984 0,981 0,996 0,988 4  0,975 0,003 0,980 0,975 0,978 0,975 0,994 0,985 5  0,907 0,001 0,907 0,907 0,907 0,906 0,993 0,901 6  0,970 0,009 0,954 0,970 0,962 0,955 0,996 0,981 7  0,931 0,006 0,945 0,931 0,938 0,931 0,989 0,959 8  0,964 0,002 0,910 0,964 0,936 0,935 0,999 0,927 9  0,980 0,001 0,952 0,980 0,966 0,965 1,000 0,993 10  0,943 0,000 0,990 0,943 0,966 0,966 0,994 0,976 11  Weighted Avg. 0,971 0,004 0,971 0,971 0,971 0,967 0,994 0,979  === Confusion Matrix ===  a b c d e f g h i j k l <-- classified as  243 0 0 2 0 1 1 0 2 2 0 0 | a = 0  0 233 1 0 0 1 0 2 1 2 0 0 | b = 1  0 1 620 2 1 3 0 1 0 0 3 0 | c = 2  0 0 3 521 5 2 0 0 0 0 0 0 | d = 3  0 0 1 5 518 0 1 0 0 0 0 0 | e = 4  2 0 0 3 3 436 3 0 0 0 0 0 | f = 5  0 0 0 0 1 2 49 0 1 0 1 0 | g = 6  0 2 0 0 0 0 0 625 17 0 0 0 | h = 7  0 1 0 0 0 0 0 23 392 4 0 1 | i = 8  0 0 0 0 0 0 0 1 2 81 0 0 | j = 9  0 0 2 0 0 0 0 0 0 0 99 0 | k = 10  0 2 0 0 0 0 0 3 0 0 1 100 | l = 11 |
| 10-fold cross validation | SMO | class 0 = betaC1  class 1 = alphaRep  class 2 = Rep  class 3 = TrAP  class 4 = REn  class 5 = sd/p.sd  class 6 = AC5  class 7 = CP  class 8 = pre-coat  class 9 = Reg  class 10 = MP  class 11 = NSP | TP Rate FP Rate Precision Recall F-Measure MCC ROC Area PRC Area Class  0,972 0,001 0,988 0,972 0,980 0,979 0,992 0,964 0  0,971 0,001 0,979 0,971 0,975 0,973 0,989 0,958 1  0,979 0,001 0,997 0,979 0,988 0,986 0,994 0,988 2  0,983 0,005 0,970 0,983 0,977 0,973 0,994 0,960 3  0,989 0,004 0,976 0,989 0,982 0,979 0,996 0,970 4  0,978 0,001 0,989 0,978 0,983 0,981 0,995 0,975 5  0,907 0,001 0,907 0,907 0,907 0,906 0,992 0,843 6  0,974 0,008 0,959 0,974 0,966 0,960 0,994 0,950 7  0,943 0,007 0,941 0,943 0,942 0,935 0,991 0,913 8  0,929 0,001 0,940 0,929 0,934 0,933 0,998 0,903 9  1,000 0,001 0,962 1,000 0,981 0,980 0,999 0,962 10  0,943 0,000 1,000 0,943 0,971 0,971 0,988 0,951 11  Weighted Avg. 0,973 0,004 0,973 0,973 0,973 0,969 0,994 0,958  === Confusion Matrix ===  a b c d e f g h i j k l <-- classified as  244 0 0 2 1 0 1 0 2 1 0 0 | a = 0  1 233 1 0 0 0 0 1 2 2 0 0 | b = 1  0 1 618 4 1 3 0 1 0 0 3 0 | c = 2  0 0 1 522 6 2 0 0 0 0 0 0 | d = 3  0 0 0 5 519 0 1 0 0 0 0 0 | e = 4  2 0 0 2 3 437 3 0 0 0 0 0 | f = 5  0 0 0 3 2 0 49 0 0 0 0 0 | g = 6  0 1 0 0 0 0 0 627 16 0 0 0 | h = 7  0 1 0 0 0 0 0 21 397 2 0 0 | i = 8  0 0 0 0 0 0 0 1 5 78 0 0 | j = 9  0 0 0 0 0 0 0 0 0 0 101 0 | k = 10  0 2 0 0 0 0 0 3 0 0 1 100 | l = 11 |
| 10-fold cross validation | RF | class 0 = betaC1  class 1 = alphaRep  class 2 = Rep  class 3 = TrAP  class 4 = REn  class 5 = sd/p.sd  class 6 = AC5  class 7 = CP  class 8 = pre-coat  class 9 = Reg  class 10 = MP  class 11 = NSP | TP Rate FP Rate Precision Recall F-Measure MCC ROC Area PRC Area Class  0,972 0,001 0,992 0,972 0,982 0,981 1,000 0,997 0  0,975 0,001 0,983 0,975 0,979 0,978 0,997 0,993 1  0,983 0,001 0,997 0,983 0,990 0,988 0,998 0,995 2  0,985 0,003 0,981 0,985 0,983 0,981 0,997 0,992 3  0,994 0,002 0,987 0,994 0,991 0,989 0,999 0,996 4  0,984 0,003 0,980 0,984 0,982 0,980 0,997 0,992 5  0,926 0,002 0,893 0,926 0,909 0,908 0,999 0,922 6  0,972 0,006 0,971 0,972 0,971 0,966 0,995 0,982 7  0,952 0,007 0,941 0,952 0,947 0,941 0,995 0,960 8  0,929 0,002 0,929 0,929 0,929 0,927 1,000 0,996 9  1,000 0,000 0,990 1,000 0,995 0,995 1,000 0,989 10  0,972 0,001 0,981 0,972 0,976 0,976 1,000 0,995 11  Weighted Avg. 0,977 0,003 0,977 0,977 0,977 0,974 0,997 0,988  === Confusion Matrix ===  a b c d e f g h i j k l <-- classified as  244 0 0 1 0 1 2 0 2 1 0 0 | a = 0  0 234 0 1 0 0 0 1 2 2 0 0 | b = 1  0 1 620 4 0 4 0 1 0 0 1 0 | c = 2  0 0 1 523 5 2 0 0 0 0 0 0 | d = 3  0 0 0 2 522 0 1 0 0 0 0 0 | e = 4  2 0 0 0 2 440 3 0 0 0 0 0 | f = 5  0 0 0 2 0 2 50 0 0 0 0 0 | g = 6  0 1 0 0 0 0 0 626 15 0 0 2 | h = 7  0 1 0 0 0 0 0 16 401 3 0 0 | i = 8  0 0 0 0 0 0 0 0 6 78 0 0 | j = 9  0 0 0 0 0 0 0 0 0 0 101 0 | k = 10  0 1 1 0 0 0 0 1 0 0 0 103 | l = 11 |
| Leave-one-out | MLP | class 0 = betaC1  class 1 = alphaRep  class 2 = Rep  class 3 = TrAP  class 4 = REn  class 5 = sd/p.sd  class 6 = AC5  class 7 = CP  class 8 = pre-coat  class 9 = Reg  class 10 = MP  class 11 = NSP | TP Rate FP Rate Precision Recall F-Measure MCC ROC Area PRC Area Class  0,964 0,001 0,992 0,964 0,978 0,976 0,991 0,975 0  0,967 0,001 0,979 0,967 0,973 0,971 0,990 0,965 1  0,981 0,002 0,989 0,981 0,985 0,982 0,996 0,992 2  0,979 0,004 0,974 0,979 0,977 0,973 0,994 0,989 3  0,987 0,004 0,974 0,987 0,980 0,977 0,996 0,989 4  0,975 0,002 0,982 0,975 0,979 0,976 0,995 0,986 5  0,889 0,002 0,889 0,889 0,889 0,887 0,992 0,913 6  0,970 0,008 0,960 0,970 0,965 0,959 0,994 0,976 7  0,936 0,007 0,940 0,936 0,938 0,931 0,984 0,953 8  0,964 0,002 0,900 0,964 0,931 0,930 1,000 0,985 9  0,960 0,001 0,960 0,960 0,960 0,959 1,000 0,981 10  0,962 0,000 0,990 0,962 0,976 0,976 0,994 0,976 11  Weighted Avg. 0,970 0,004 0,970 0,970 0,970 0,966 0,994 0,979  === Confusion Matrix ===  a b c d e f g h i j k l <-- classified as  242 0 0 1 1 0 2 0 2 3 0 0 | a = 0  0 232 1 0 0 0 0 2 3 2 0 0 | b = 1  0 1 619 3 1 3 0 1 0 0 3 0 | c = 2  0 0 1 520 8 2 0 0 0 0 0 0 | d = 3  0 0 1 5 518 0 1 0 0 0 0 0 | e = 4  2 0 0 4 2 436 3 0 0 0 0 0 | f = 5  0 0 0 1 2 3 48 0 0 0 0 0 | g = 6  0 1 0 0 0 0 0 625 17 0 0 1 | h = 7  0 1 0 0 0 0 0 22 394 4 0 0 | i = 8  0 0 0 0 0 0 0 0 3 81 0 0 | j = 9  0 0 4 0 0 0 0 0 0 0 97 0 | k = 10  0 2 0 0 0 0 0 1 0 0 1 102 | l = 11 |
| Leave-one-out | SMO | class 0 = betaC1  class 1 = alphaRep  class 2 = Rep  class 3 = TrAP  class 4 = REn  class 5 = sd/p.sd  class 6 = AC5  class 7 = CP  class 8 = pre-coat  class 9 = Reg  class 10 = MP  class 11 = NSP | TP Rate FP Rate Precision Recall F-Measure MCC ROC Area PRC Area Class  0,976 0,001 0,992 0,976 0,984 0,983 0,992 0,970 0  0,971 0,001 0,979 0,971 0,975 0,973 0,991 0,960 1  0,981 0,001 0,995 0,981 0,988 0,986 0,994 0,988 2  0,981 0,005 0,967 0,981 0,974 0,970 0,993 0,956 3  0,989 0,003 0,981 0,989 0,985 0,983 0,997 0,974 4  0,978 0,001 0,989 0,978 0,983 0,981 0,994 0,975 5  0,926 0,001 0,909 0,926 0,917 0,916 0,992 0,855 6  0,974 0,008 0,957 0,974 0,965 0,959 0,994 0,950 7  0,941 0,007 0,938 0,941 0,940 0,932 0,991 0,910 8  0,917 0,001 0,939 0,917 0,928 0,926 0,998 0,897 9  1,000 0,001 0,971 1,000 0,985 0,985 1,000 0,971 10  0,943 0,000 1,000 0,943 0,971 0,971 0,988 0,951 11  Weighted Avg. 0,973 0,004 0,973 0,973 0,973 0,969 0,994 0,959  === Confusion Matrix ===  a b c d e f g h i j k l <-- classified as  245 0 0 2 0 0 1 0 2 1 0 0 | a = 0  0 233 1 1 0 0 0 1 2 2 0 0 | b = 1  0 1 619 5 0 3 0 1 0 0 2 0 | c = 2  0 0 2 521 6 2 0 0 0 0 0 0 | d = 3  0 0 0 5 519 0 1 0 0 0 0 0 | e = 4  2 0 0 2 3 437 3 0 0 0 0 0 | f = 5  0 0 0 3 1 0 50 0 0 0 0 0 | g = 6  0 1 0 0 0 0 0 627 16 0 0 0 | h = 7  0 1 0 0 0 0 0 22 396 2 0 0 | i = 8  0 0 0 0 0 0 0 1 6 77 0 0 | j = 9  0 0 0 0 0 0 0 0 0 0 101 0 | k = 10  0 2 0 0 0 0 0 3 0 0 1 100 | l = 11 |
| Leave-one-out | RF | class 0 = betaC1  class 1 = alphaRep  class 2 = Rep  class 3 = TrAP  class 4 = REn  class 5 = sd/p.sd  class 6 = AC5  class 7 = CP  class 8 = pre-coat  class 9 = Reg  class 10 = MP  class 11 = NSP | TP Rate FP Rate Precision Recall F-Measure MCC ROC Area PRC Area Class  0,968 0,001 0,992 0,968 0,980 0,979 1,000 0,996 0  0,975 0,001 0,983 0,975 0,979 0,978 0,995 0,991 1  0,983 0,001 0,994 0,983 0,988 0,986 0,998 0,995 2  0,985 0,003 0,981 0,985 0,983 0,981 0,997 0,994 3  0,994 0,002 0,985 0,994 0,990 0,988 0,999 0,994 4  0,984 0,003 0,978 0,984 0,981 0,979 0,998 0,992 5  0,870 0,002 0,887 0,870 0,879 0,877 0,999 0,939 6  0,967 0,006 0,969 0,967 0,968 0,962 0,995 0,981 7  0,960 0,007 0,937 0,960 0,948 0,942 0,993 0,956 8  0,952 0,000 0,988 0,952 0,970 0,969 1,000 0,998 9  0,990 0,000 0,990 0,990 0,990 0,990 1,000 0,985 10  0,962 0,001 0,962 0,962 0,962 0,961 1,000 0,995 11  Weighted Avg. 0,976 0,003 0,976 0,976 0,976 0,973 0,997 0,987  === Confusion Matrix ===  a b c d e f g h i j k l <-- classified as  243 0 0 0 0 1 2 0 4 1 0 0 | a = 0  0 234 0 1 0 0 0 2 2 0 0 1 | b = 1  0 1 620 4 0 5 0 0 0 0 1 0 | c = 2  0 0 1 523 5 2 0 0 0 0 0 0 | d = 3  0 0 0 2 522 0 1 0 0 0 0 0 | e = 4  2 0 0 0 2 440 3 0 0 0 0 0 | f = 5  0 0 1 3 1 2 47 0 0 0 0 0 | g = 6  0 1 0 0 0 0 0 623 17 0 0 3 | h = 7  0 1 0 0 0 0 0 16 404 0 0 0 | i = 8  0 0 0 0 0 0 0 0 4 80 0 0 | j = 9  0 0 1 0 0 0 0 0 0 0 100 0 | k = 10  0 1 1 0 0 0 0 2 0 0 0 102 | l = 11 |
